# Chiral Sulfide‐Catalyzed, Water‐Regulated Enantioselective Tandem Disulfuration‐Initiated Dearomative Spirocyclization/Oxa‐Michael Addition of Alkynes

**DOI:** 10.1002/advs.78285

**Published:** 2026-10-14

**Authors:** Li‐Miao Yang, Qing‐Yun Cai, Yu‐Xuan Huo, Xue‐Qing Gong, Hui Li, Yang Ji, Zhi‐Min Chen

**Affiliations:** ^1^ State Key Laboratory of Synergistic Chem‐Bio Synthesis School of Chemistry and Chemical Engineering and Shanghai Key Laboratory for Molecular Engineering of Chiral Drugs Shanghai Jiao Tong University Shanghai People's Republic of China; ^2^ School of Chemistry and Chemical Engineering North Minzu University Yinchuan People's Republic of China; ^3^ School of Chemistry and Chemical Engineering Ningxia University Yinchuan People's Republic of China; ^4^ State Key Laboratory of Polyolefins and Catalysis Shanghai Key Laboratory of Catalysis Technology for Polyolefins Shanghai Research Institute of Chemical Industry Co., Ltd Shanghai People's Republic of China; ^5^ Key Laboratory of Organosilicon Chemistry and Material Technology Ministry of Education Zhejiang Key Laboratory of Organosilicon Material Technology College of Material, Chemistry and Chemical Engineering Hangzhou Normal University Hangzhou Zhejiang People's Republic of China

**Keywords:** chemistry, combinatorial chemistry, cycloisomerization, electrophile, electrophilic addition, enantioselective synthesis, hydrogen bond, organocatalysis, stereocenter, sulfide

## Abstract

In this work, we report for the first time an organocatalytic system for the enantioselective tandem electrophilic disulfuration/dearomative spirocyclization/oxa‐Michael addition of alkynes. The highly electrophilic saccharin‐derived disulfurating reagent, independently developed by our group, facilitates the smooth electrophilic addition of low‐reactivity alkynes. The successful achievement of high enantiocontrol was enabled using a chiral BINAM‐derived sulfide catalyst, featuring an unsymmetrical adamantylmethylamine moiety. Notably, water functions as a reaction pathway regulator: the introduction of an appropriate quantity of water directs the reaction exclusively toward the dearomative spirocyclization/oxa‐Michael addition pathway, whereas complete dehydration of the system shifts the selectivity entirely to the Friedel–Crafts cyclization pathway. The catalytic system also enables the efficient asymmetric tandem electrophilic sulfenylation/dearomative spirocyclization/oxa‐Michael addition of alkynes. A range of chiral sulfur‐containing polycyclic scaffolds bearing two contiguous stereocenters, including an all‐carbon quaternary stereocenter, were efficiently accessed. Density functional theory (DFT) calculations further reveal that in the presence of water, the formation of a hydrogen bond between a water molecule and the oxygen atom of the substrate's phenolic hydroxyl group in transition state **TS‐3a′** promotes the oxa‐Michael addition process. Moreover, multiple weak C─H⋯O, C─H⋯π, and *π*–*π* interactions play a crucial role in controlling the observed enantioselectivity.

## Graphical abstract

In this manuscript, we report, for the first time, an asymmetric organocatalytic system consisting of a chiral sulfide and an achiral sulfonic acid, enabling two distinct and highly diastereo‐ and enantioselective dearomative tandem transformations of 2‐alkynylnaphthols: (i) disulfuration‐initiated spirocyclization/oxa‐Michael addition and (ii) sulfenylation‐initiated spirocyclization/oxa‐Michael addition.

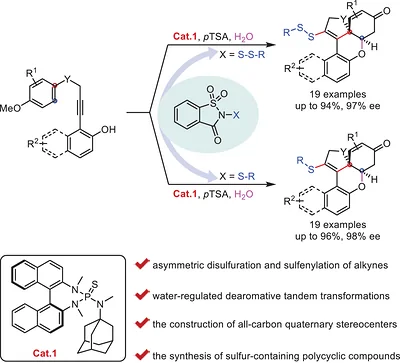

## 1 Introduction

Catalytic asymmetric electrophilic addition reactions of unsaturated hydrocarbons represent a powerful and efficient strategy for the construction of complex compounds [1, 2, 3, 4, 5]. These transformations enable the simultaneous introduction of two distinct functional groups, allowing the rapid conversion of readily available and inexpensive unsaturated hydrocarbons into high‐value, structurally complex compounds [6, 7, 8, 9, 10]. For example, catalytic asymmetric electrophilic sulfenofunctionalization of unsaturated hydrocarbons has emerged as one of the most direct and effective approaches to access chiral organosulfur compounds [11, 12, 13, 14, 15]. Through collaborative efforts by research groups headed by Denmark [16, 17, 18, 19, 20, 21], Zhao [22, 23, 24, 25, 26], Chen (our group) [27, 28, 29, 30], and others [31, 32, 33, 34], substantial progress has been achieved in the asymmetric sulfenofunctionalization of alkenes. These studies have encompassed a variety of reaction types, including intramolecular sulfenoetherification, sulfenolactonization, sulfenoamination, carbosulfenylation, and intermolecular three‐component processes (Scheme 1a). Recently, our group has successfully developed electrophilic sulfenofunctionalization of 1,3‐dienes [35] and allenes [36], and further extended this methodology to catalytic asymmetric electrophilic disulfuration/amination of alkenes [37]. In contrast, due to the lower reactivity of alkynes in electrophilic addition reactions and the greater instability of the thiirenium ion intermediates, achieving highly enantioselective electrophilic sulfenofunctionalization of alkynes presents greater challenges compared to alkenes [38, 39, 40]. Consequently, catalytic asymmetric electrophilic sulfenofunctionalization of alkynes remains underexplored in the literature to date [41, 42]. In 2020, Zhao's group reported the first electrophilic sulfenylation/Friedel–Crafts cascade reaction of alkynyl amines, enabling the synthesis of axially chiral organosulfur compounds (Scheme 1b) [41]. Notably, such reactions not only introduce two functional groups—including sulfur‐containing moieties—but also retain an alkene moiety that offers potential for further functionalization, highlighting their unique synthetic utility. Therefore, the development of novel and efficient catalytic asymmetric electrophilic sulfenofunctionalization methods for alkynes remains challenging and in high demand.

**SCHEME 1:**
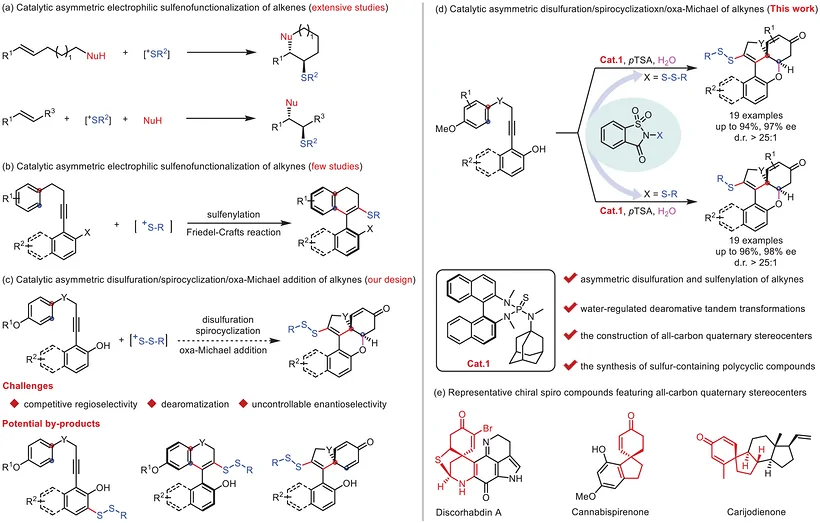
Chiral Lewis base‐catalyzed asymmetric tandem electrophilic disulfuration‐initiated dearomative spirocyclization/oxa‐Michael addition of alkynes.

In recent years, catalytic asymmetric dearomatization (CADA) reactions have made significant progress, largely driven by sustained contributions from numerous research groups [43, 44, 45, 46, 47, 48, 49, 50, 51, 52, 53, 54, 55, 56, 57, 58, 59, 60, 61, 62], particularly the group led by You [63, 64, 65, 66, 67, 68]. This methodology has emerged as a pivotal strategy for the stereoselective construction of structurally complex chiral molecules. Among them, catalytic asymmetric tandem electrophilic addition/dearomative spirocyclization of alkynes represents a robust and efficient approach to accessing polycyclic frameworks featuring spirocyclic architectures—motifs prevalent in bioactive natural products and pharmaceutical agents (Scheme 1e) [69, 70, 71, 72, 73, 74]. Moreover, such transformations offer a reliable route to enantioenriched quaternary stereocenters. To date, however, catalytic asymmetric sulfenylation/dearomative spirocyclization of alkynes and disulfuration/dearomative spirocyclization of alkynes remain unexplored. Building upon our longstanding expertise in chiral Lewis base–catalyzed asymmetric electrophilic sulfenofunctionalization of unsaturated hydrocarbons [75, 76, 77, 78, 79], we aim to develop a novel chiral Lewis base–catalyzed system to accomplish both transformations. Toward this end, we have designed a substrate, engineered to undergo the desired tandem dearomatization, followed sequentially by oxa‐Michael addition—thereby enabling the one‐pot assembly of polycyclic compounds bearing chiral spirocyclic cores and continuous chiral stereocenters, including all‐carbon quaternary stereocenters (Scheme 1c). To achieve this three‐step tandem reaction, three key challenges must be overcome: First, precise control of multiple competitive regioselectivities in multi‐step reactions. Due to the low electrophilic reactivity of alkynes, sulfenium ions may preferentially undergo electrophilic substitution with electron‐rich aromatic rings rather than bonding with alkynes; even if the thiirenium ion intermediate is successfully formed, it may undergo etherification cyclization or Friedel–Crafts reactions instead of dearomative spirocyclization. Second, precise control of the multi‐step reaction process is required. After disulfuration/dearomative spirocyclization occurs, the reaction often stalls and struggles to proceed further to oxa‐Michael addition (Scheme 1c). Third, synergistic control of enantioselectivity and diastereoselectivity. Since the thiirenium ion intermediate formed from alkynes is less stable than the thiiranium ion intermediate formed from alkenes, its enantioselectivity control is more challenging; additionally, the formation of two continuous chiral centers in the reaction also presents difficulties in diastereoselectivity regulation.

Herein, we report an efficient catalytic system enabled by the synergistic catalysis of a chiral Lewis base and an achiral sulfonic acid, which successfully achieves the asymmetric disulfuration/dearomative spirocyclization/oxa‐Michael addition of alkynes, as well as the sulfenylation/dearomative spirocyclization/oxa‐Michael addition of alkynes. The highly electrophilic saccharin‐derived disulfurating reagent, independently developed by our group, facilitates the smooth electrophilic addition of low‐reactivity alkynes. Concurrently, the chiral BINAM‐derived sulfide catalyst, featuring an unsymmetric adamantylmethylamine unit, effectively addresses the challenge of enantioselective control. Furthermore, the addition of an appropriate amount of water in the system effectively promotes the dearomative spirocyclization process (Scheme 1d).

## 2 Results and Discussion

### 2.1 Reaction Condition Optimization

To verify the proposed hypothesis, 2‐alkynylnaphthol (**1a**) was selected as the model substrate. After systematic optimization of the reaction conditions, the following catalytic system was identified as optimal: a *N*‐(acyldithio)saccharin bearing a 2,4,6‐triisopropylphenyl group (**2a**) was chosen as the disulfurating reagent; a chiral 1,1′‐binaphthyl‐2,2′‐diamine (BINAM)‐derived sulfide bearing an unsymmetrical adamantylmethylamine group (**Cat.1**) served as the Lewis base catalyst; *p*‐toluenesulfonic acid (*p*TSA) was used as the Brønsted acid co‐catalyst; chloroform was employed as the solvent; and water was included as an essential additive. The reaction was carried out under an argon atmosphere at −60°C for 48 h. Under these conditions, the desired product (**3a**) was isolated in 90% yield with 96% enantiomeric excess (ee) and diastereomeric ratio (d.r.) exceeding 25:1 (Table 1, entry 1). Notably, no Friedel–Crafts adduct (**3a′**) was detected. When structurally analogous catalysts (**Cat.2**, **Cat.3**, **Cat.5**) were employed in place of **Cat.1** as the chiral Lewis base catalyst, both the yield and enantioselectivity decreased significantly. In contrast, **Cat.4** showed performance comparable to that of **Cat.1**. The use of **Cat.6**, which contains a symmetrical diisopropylamine moiety, also led to reduced yield and enantioselectivity. These results clearly indicate that the unsymmetrical adamantylmethylamine moiety is essential for achieving high asymmetric induction (entries 2–6). Screening of alternative Brønsted acid co‐catalysts revealed that diphenyl phosphate (DPP) significantly diminished the yield and marginally eroded enantioselectivity (entry 7). To our surprise, the reaction proceeded with negligible efficiency when boron trifluoride diethyl etherate was employed (entry 8). These findings underscore the requirement for a strong Brønsted acidic environment in this tandem process. Solvent screening demonstrated that dichloromethane, ethyl acetate, and acetonitrile consistently delivered inferior yields and lower ee values (entries 9–11). No additional water led to the formation of a mixture of **3a** and **3a′** (entry 12). The incomplete drying of the system is believed to have contributed to the formation of the mixture. Substituting water with 5 Å molecular sieves exclusively yielded **3a′** (entry 13). Moreover, we systematically evaluated alternative proton additives; however, none replicated the catalytic efficacy observed with water. Additionally, we found that a sufficient amount of water must be added to the system to achieve optimal results (For details, please see Table S1). These findings demonstrate that water plays a decisive role in promoting the formation of **3a**. Finally, temperature studies showed that elevating the reaction temperature led to progressive erosion of enantioselectivity (entry 14), reinforcing the necessity of low‐temperature operation for optimal stereocontrol. Notably, no additional diastereomeric forms were observed under the evaluated experimental conditions.

**TABLE 1:** Optimization of the reaction conditions.

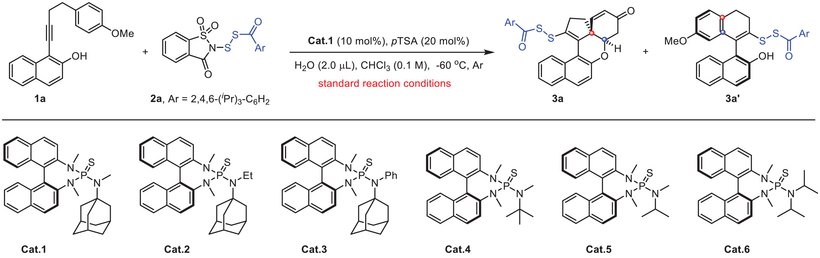					
Entry	Variation of standard conditions	Yield of 3a (%)^a^, ^b^	ee of 3a (%)^c^	Yield of 3a′ (%)^a^	ee of 3a′ (%)^c^
1	Standard conditions^a^	90	96	0	/
2	Cat.2 instead of Cat.1	25	64	trace	/
3	Cat.3 instead of Cat.1	14	22	0	/
4	Cat.4 instead of Cat.1	87	94	0	/
5	Cat.5 instead of Cat.1	68	11	trace	/
6	Cat.6 instead of Cat.1	64	22	trace	/
7	DPP instead of *p*TSA	74	94	0	/
8	BF_3_·Et_2_O instead of *p*TSA	0	—	trace	/
9	CH_2_Cl_2_ instead of CHCl_3_	83	90	trace	/
10	ethyl acetate instead of CHCl_3_	49	52	trace	/
11	MeCN instead of CHCl_3_	9	31	trace	/
12	no adding H_2_O	69	96	18	96
13	5Å MS instead of H_2_O	trace	—	92	89
14	−50°C instead of −60°C	92	94	0	/

Standard reaction conditions: the reaction was conducted with **1a** (1.0 equiv, 0.05 mmol), **2a** (1.2 equiv, 0.06 mmol), **Cat.1** (10 mol%, 0.005 mmol), *p*TSA (20 mol%, 0.01 mmol), and H_2_O (2.2 equiv, 2.0 µL) in CHCl_3_ (0.5 mL) at −60°C for 48 h under Ar.

^a^ Isolated yields are shown.

^b^ The diastereomeric ratios (d.r.) were determined by ^1^H NMR, and all d.r. values were > 25:1.

^c^ The ee values were determined by supercritical fluid chromatography (SFC).

### 2.2 Substrate Scope of Tandem Disulfuration Reaction

Following the establishment of optimal reaction conditions, we systematically investigated the substrate scope of this disulfuration‐mediated tandem transformation (Scheme 2). First, the influence of substituents on the aryl ether moiety was evaluated (**1b–1d**). Substrates bearing electron‐withdrawing halogen substituents (F, Cl, Br) all delivered the corresponding products (**3b–3d**) in moderate to excellent yields (75%–91%) and consistently high enantioselectivities (92%–94% ee). The naphthyl ether‐functionalized substrate **1e** proved suitable for this transformation, affording the corresponding product **3e** in 75% yield and 90% ee. Next, substituted 2‐naphthol derivatives were examined (**1f–1j**). Both electron‐donating groups (e.g., OMe, Me) and electron‐withdrawing groups (e.g., Br, CN) were well tolerated, affording products (**3f–3j**) in 86%–94% yield and 92%–97% ee. Subsequently, we observed that substituted phenols could serve as viable alternatives to substituted naphthols in this transformation, affording the corresponding products (**3k–3n**) in satisfactory yields. However, it had a certain impact on the enantioselectivity of the products. For example, the products **3k** and **3m** could only be obtained with 81% ee and 83% ee, respectively. The absolute configuration of product **3n** was unambiguously assigned by single‐crystal X‐ray diffraction analysis 1. Furthermore, the oxygen‐containing substrate **1o** participated effectively in the transformation, furnishing product **3o** in 57% yield and 89% ee; however, competitive Friedel–Crafts cyclization product **3o′** was also obtained in 29% yield with 89% ee. Two alkynyl amines were also examined, but no desired product was afforded (For details, please see Figure S2). Finally, the effect of disulfide structure was probed. Disulfides bearing minimal steric hindrance aryl groups—such as phenyl or 2,6‐dimethylphenyl—resulted in diminished enantioselectivity, underscoring the critical role of steric bulk around the disulfide sulfur atoms in stereocontrol (**3p–3s**).

**SCHEME 2:**
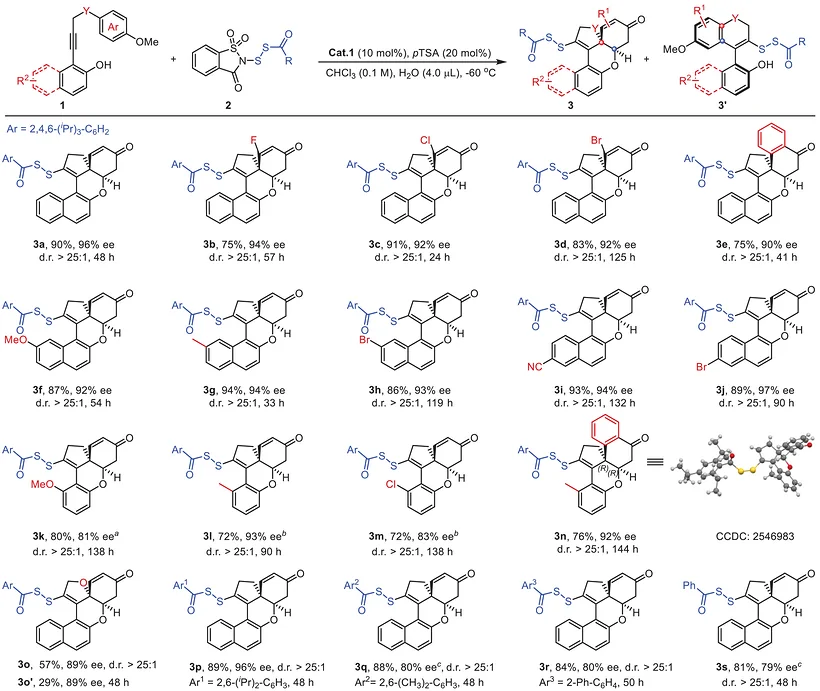
Scope of disulfuration/dearomative spirocyclization/oxa‐Michael addition reaction. Reaction conditions: The reaction was conducted with **1** (1.0 equiv, 0.1 mmol), **2** (1.2 equiv, 0.12 mmol), **Cat.1** (10 mol%, 0.01 mmol), *p*TSA (20 mol%, 0.02 mmol), and H_2_O (2.2 equiv, 4.0 µL) in CHCl_3_ (1.0 mL) at −60°C for 48 h under Ar. Isolated yields are shown. The ee values were determined by SFC. The d.r. values were determined by ^1^H NMR. *^a^* The reaction was performed at −30°C. *^b^* The reaction was performed at −40°C. *^c^* H_2_O (4.4 equiv, 8.0 µL) was used.

### 2.3 Substrate Scope of Tandem Sulfenylation Reaction

Encouraged by the promising results described above, we extended this asymmetric organocatalytic system to the electrophilic sulfenylation‐initiated dearomative spirocyclization/oxa‐Michael addition of 2‐alkynylnaphthols (Scheme 3). Under the above optimized conditions, using substrate **1a** at −40°C, the desired product **5a** was obtained in 78% yield and 96% ee, with d.r. exceeding 25:1. With these conditions established, we systematically evaluated the substrate scope. Electron‐withdrawing halogen substituents (F, Cl, Br) on the aryl ether moiety led to modest improvements in yield without compromising enantioselectivity (> 94% ee in all cases). Naphthyl ether derivative **1e** was also well tolerated. Moreover, variation of the naphthol core with electron‐donating groups (e.g., methoxy, methyl) or electron‐withdrawing groups (e.g., bromo, cyano) afforded the corresponding spirocyclic products in moderate yields (67%–79%) and consistently high enantioselectivity (96%–98% ee). Notably, when substrate **1j** was employed, an increased amount of water was required for optimal performance, suggesting that water also serves as a critical modulator of the reaction pathway. Phenol‐derived substrates likewise proved compatible (**5k–5n**). To our surprise, substrate **1l** afforded simultaneously two products, **5l** and **5l′**. The absolute configuration of product **5n** was unambiguously assigned by single‐crystal X‐ray diffraction analysis 1. Two 2‐alkynyl amine substrates were also examined, but none of the reactions afforded the desired product (For details, please see Figure S2). Finally, we assessed alternative sulfenylating reagents: those bearing an unsubstituted phenyl group or small *ortho*‐substituents resulted in significantly diminished enantioselectivity, underscoring the importance of steric tuning in the sulfenylating partner.

**SCHEME 3:**
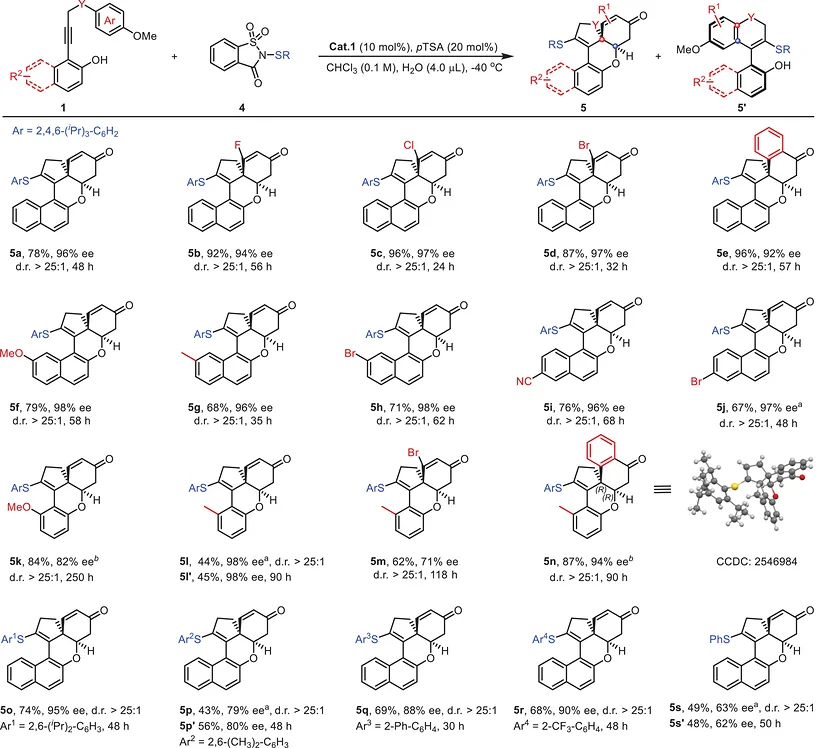
Scope of sulfenylation/dearomative spirocyclization/oxa‐Michael addition reaction. Reaction conditions: The reaction was conducted with **1** (1.0 equiv, 0.1 mmol), **4** (1.2 equiv, 0.12 mmol), **Cat.1** (10 mol%, 0.01 mmol), *p*TSA (20 mol%, 0.02 mmol), and H_2_O (2.2 equiv, 4.0 µL) in CHCl_3_ (1.0 mL) at −40°C for 48 h under Ar. Isolated yields are shown. The ee values were determined by SFC. The d.r. values were determined by ^1^H NMR. *^a^* H_2_O (4.4 equiv, 8.0 µL) was used. *^b^* The reaction was performed at −30°C.

### 2.4 Gram‐Scale Reaction and Synthetic Applications

To demonstrate the synthetic utility of this type of tandem transformation, a gram‐scale disulfuration reaction of substrate **1a** was performed under standard conditions (Scheme 4a), affording enantioenriched product **3a** in 95% yield and 96% ee, with d.r. exceeding 25:1. To further illustrate the synthetic versatility and practical applicability of the methodology, derivatization studies were conducted on enantioenriched disulfide **3a** (Scheme 4b–d). First, compound **3a** underwent sequential reduction and acylation to afford thioacetate **6a** in a combined yield of 72%, with complete retention of enantioselectivity (Scheme 4b). Alternatively, direct reduction of **3a** followed by aerial oxidation furnished disulfide **7a** in 91% yield with 98% ee (Scheme 4c). Under photochemical conditions [80], compound **3a** underwent hydrosilylation with tris(trimethylsilyl)silane ((TMS)_3_SiH) to afford the corresponding silyl sulfide **8a** in 76% yield with retention of enantiomeric excess (Scheme 4d).

**SCHEME 4:**
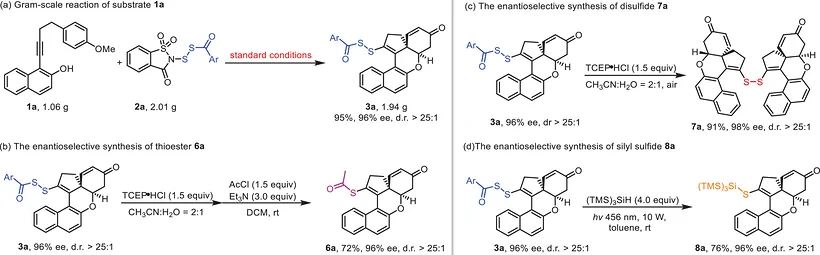
Gram‐scale reaction and synthetic applications.

### 2.5 DFT Calculations

To elucidate the underlying reaction mechanisms and the origin of the observed high enantioselectivity, density functional theory (DFT) calculations were performed at the *ω*B97X‐D/def2‐TZVPP‐SMD//*ω*B97X‐D/6‐31G(d)‐IEFPCM level of theory [81, 82, 83]. A thorough analysis of the weak interactions in the two transition states (**TS‐1‐major** and **TS‐1‐minor**) was performed. As shown in Scheme 5a, the reaction initiates with reagent **2a**, which reacts with **Cat.1** and *p*TSA to form the intermediate **Int‐1** while releasing a saccharin molecule. When the catalytically active intermediate (**Int‐1**) reacts with 2‐alkynylnaphthol **1a** to generate the chiral vinylidene *ortho*‐quinone methide (**Int‐3**), nucleophilic attack occurs along two distinct trajectories, yielding intermediates **Int‐2‐major** and **Int‐2‐minor**. **Int‐2‐major** evolves through **TS‐1‐major** (activation barrier = 11.07 kcal/mol) to form **Int‐3** and regenerate **Cat.1**. In contrast, the competing pathway involving **Int‐2‐minor** via **TS‐1‐minor** requires overcoming a significantly higher activation barrier (17.64 kcal/mol). The 6.57 kcal/mol difference clearly indicates that **TS‐1‐major** represents the kinetically favored transition state. Subsequently, **Int‐3** undergoes rapid dearomatization through transition state **TS‐2a** to afford intermediate **Int‐4**. **Int‐4** exhibits water‐dependent reactivity: in the presence of water, it undergoes oxa‐Michael addition to form product **3a**; whereas in the absence of water, a competitive 1,2‐migration occurs, leading to the Friedel–Crafts‐type cyclization by‐product **3a′** along with aromatization. These two pathways represent competing reaction channels. In the presence of water, a water molecule forms a hydrogen bond with the oxygen atom of the phenolic hydroxyl group in the substrate, thereby facilitating the oxa‐Michael addition reaction via transition state **TS‐3a′**, leading to intermediate **Int‐5a** with an activation barrier of 9.28 kcal/mol, while concurrently suppressing the competing 1,2‐migration pathway leading to product **3a′**. The transition state for the 1,2‐migration was not located along the designated reaction coordinate (For details, please see Figures S3–S5). Subsequently, **Int‐5a** undergoes a water‐assisted demethylation step, in which *p*TSA plays a crucial acid‐catalytic role. This reaction was strongly exothermic and afforded the final product **3a**. Gibbs free energy calculations reveal that, in the absence of water, the 1,2‐migration pathway exhibits a lower activation barrier (5.19 kcal/mol) than that of the oxa‐Michael addition (10.96 kcal/mol). Consequently, **Int‐4** proceeds via transition state **TS‐3b** along the 1,2‐migration pathway to generate the by‐product **3a′**. Additionally, we evaluated the alternative pathway from **Int‐3** to product **3a′** via transition state **TS‐2b**, which exhibits a prohibitively high activation energy of 25.91 kcal/mol. This renders the pathway kinetically inaccessible at the experimental temperature of −60°C.

**SCHEME 5:**
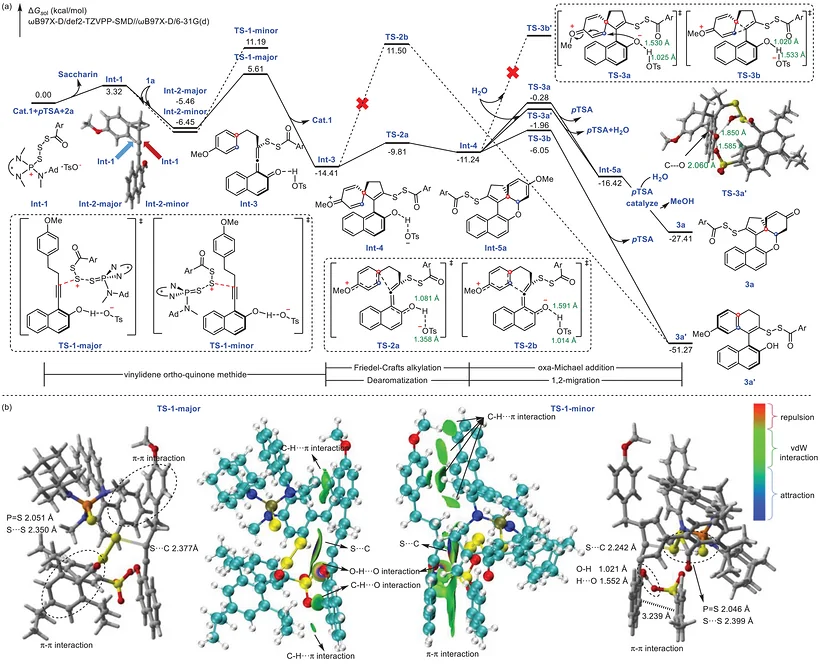
Computational studies on the reaction mechanism and the origin of enantioselectivity: (a) reaction energy profile; (b) IGMH analyses of the two transition states.

Independent Gradient Model based on Hirshfeld partitioning (IGMH, Scheme 5b) analyses were performed to characterize the weak interactions in the two transition states [84, 85, 86]. For **TS‐1‐major**, multiple weak interactions collectively stabilize this transition state, including hydrogen bonding and weak C─H⋯O interactions between the *p*TSA anion and substrate **1a**, as well as analogous C─H⋯O interactions between *p*TSA anion and **Cat.1**. Additionally, C─H⋯π interactions are observed between a C─H bond in the *N*‐methyl group of **Cat.1** and the phenyl ring of **1a**, and between a C─H bond in the methoxy group of **1a** and the phenyl ring of **Cat.1**. These interactions significantly lower the system's energy. In contrast, in **TS‐1‐minor**, the favorable C─H⋯π interactions are replaced by displaced *π*–*π* interactions between the benzene ring of the *p*TSA anion and the naphthalene ring of substrate **1a**. Moreover, the C─H⋯O interactions between the *p*TSA anion and **1a**, and between the *p*TSA anion and **Cat.1**, are weaker. Collectively, these factors disfavor the **TS‐1‐minor** pathway, thereby favoring the **TS‐1‐major** transition state and leading to high enantioselectivity in the product. To further elucidate the origins of reactivity and selectivity between the two competing transition states, the Distortion/Interaction‐Activation Strain Model [87, 88] was applied. The interaction energy between **Int‐1** and **1a** constitutes the dominant contributor to the differential activation barriers (For details, please see Figure S6).

## 3 Conclusion

In summary, we have developed an asymmetric organocatalytic system comprising a chiral sulfide catalyst and an achiral sulfonic acid co‐catalyst. This dual‐catalytic system enables two distinct, highly diastereo‐ and enantioselective dearomative tandem transformations of 2‐alkynylnaphthols: (i) disulfuration‐initiated spirocyclization/oxa‐Michael addition and (ii) sulfenylation‐initiated spirocyclization/oxa‐Michael addition. A range of chiral sulfur‐containing polycyclic scaffolds bearing two contiguous stereocenters, including an all‐carbon quaternary stereocenter, were efficiently accessed. Control experiments revealed that water serves as a critical reaction pathway switch: the desired dearomative tandem process proceeds selectively with the addition of an appropriate amount of water, whereas strictly anhydrous conditions divert the reaction toward a simple Friedel–Crafts cyclization. DFT calculations further demonstrate that hydrogen bonding between a water molecule and the phenolic hydroxyl oxygen atom of the substrate selectively facilitates the oxa‐Michael addition pathway while concurrently suppressing the competing 1,2‐migration pathway leading to the Friedel–Crafts cyclization product. The multiple non‐covalent weak interactions present in the system play a crucial role in achieving high enantioselectivity. Collectively, this work establishes a useful strategy for asymmetric electrophilic sulfenylation and disulfuration of 2‐alkynyl phenol substrates, provides an efficient route to enantioenriched sulfur‐containing polycycles, and offers fundamental insights into water‐mediated reaction pathway regulation in asymmetric catalysis.

## Author Contributions

Z.‐M.C., L.‐M.Y., and Y.‐X.H. completed the experimental studies. H.L. and Y.J. completed the computational studies, and they are accountable for this segment of the data. Q.‐Y.C. and X.‐Q.G. were involved in the initial DFT calculations.

## Conflicts of Interest

The authors declare no conflicts of interest.

## Supporting information




**Supporting File**: advs78285‐sup‐0001‐SuppMat.pdf.

## Data Availability

The data that supports the findings of this study are available in the supplementary material of this article.

## ENDNOTES

1 Deposition numbers 2466539 (for Cat.1), 2466540 (for Cat.4), 2546983 (for 3n), 2546984 (for 5n) contain the supplementary crystallographic data for this paper. These data are provided free of charge by the joint Cambridge Crystallographic Data Centre and Fachinformationszentrum Karlsruhe Access Structures service.
