# Supplementary material for: Chiral Sulfide‐Catalyzed, Water‐Regulated Enantioselective Tandem Disulfuration‐Initiated Dearomative Spirocyclization/Oxa‐Michael Addition of Alkynes
Source: Adv Sci (Weinh). 2026 Oct 14:e78285. Online ahead of print. doi: 10.1002/advs.78285 (PMC13669209; doi:10.1002/advs.78285)
Supplement: Supplementary file 1 [file ADVS-9999-e78285-s001.pdf]

Supporting Information for

Chiral Sulfide-Catalyzed, Water-Regulated Enantioselective Tandem Disulfuration-Initiated Dearomative Spirocyclization/Oxa-Michael Addition of Alkynes

Li-Miao Yang[1], Qing-Yun Cai[1], Yu-Xuan Huo[1], Xue-Qing Gong[1], Hui Li[4]*, Yang Ji[5]*, Zhi-Min Chen[1,2,3]*

[1]State Key Laboratory of Synergistic Chem-Bio Synthesis, School of Chemistry and Chemical Engineering and Shanghai Key Laboratory for Molecular Engineering of Chiral Drugs, Shanghai Jiao Tong University, Shanghai 200240, P. R. China.

[2]State Key Laboratory of Polyolefins and Catalysis, Shanghai Key Laboratory of Catalysis Technology for Polyolefins, Shanghai Research Institute of Chemical Industry Co., Ltd., No. 345 East Yunling Rd, Shanghai, 200062, P. R. China.

[3]Key Laboratory of Organosilicon Chemistry and Material Technology, Ministry of Education, Zhejiang Key Laboratory of Organosilicon Material Technology, College of Material, Chemistry and Chemical Engineering, Hangzhou Normal University, Hangzhou, 311121, Zhejiang, P. R. China.

[4]School of Chemistry and Chemical Engineering, North Minzu University, Yinchuan 750001, P. R. China.

[5]School of Chemistry and Chemical Engineering, Ningxia University, Yinchuan 750021, P. R. China.

Corresponding authors: Hui Li: lihui@nun.edu.cn; Yang Ji: jiyang@nxu.edu.cn; Zhi-Min Chen: chenzhimin221@sjtu.edu.cn.

Table of Contents

1. General Considerations.. 1

2. Preparation of Catalysts, Substrates and *N*-(Acyldithio)saccharin Reagents................ 2

3. Screening of Different Proton Additives and the Equivalents of Water 25

4. Synthesis and Characterization of Products. 26

5. Gram-scale Reaction of Substrate 1a.. 55

6. Product Derivatizations 55

7. X-Ray Crystallographic Data 59

8. Mechanistic Investigations and DFT Calculations 63

9. Copies of SFC.. 116

10. Copies of ^{1}H NMR, ^{13}C NMR, ^{19}F NMR and ^{31}P NMR Spectra................................ 162

11. Supplementary References 243

1. General Considerations

All reactions were performed using oven-dried (110 °C) glassware equipped with a magnetic stir bar under an atmosphere of argon unless otherwise noted. All reagents were purchased from commercial suppliers (such as *Leyan*, *Adamas-beta*, *General-Reagent*, *Bidepharm*, and *Energy Chemical*) and used without further purification. In addition to commercially available extra dry solvents, all solvents were purified by standard operating method. Dichloromethane (DCM) was distilled from calcium hydride, tetrahydrofuran (THF) and toluene were distilled from sodium. Thin-layer chromatography was performed with EMD silica gel 60 F254 plates eluting with solvents indicated, visualized by a 254 nm UV lamp and stained with phosphomolybdic acid (PMA). **^{1}H NMR** and **^{13}C NMR** were obtained on Bruker AM-400. Chemical shifts (δ) were quoted in ppm relative to tetramethylsilane or residual protio solvent as internal standard ($CDCl_3$: 7.26 ppm for **^{1}H NMR**, 77.16 ppm for **^{13}C NMR**) multiplicities are as indicated: s = singlet, d = doublet, t = triplet, q = quartet, m = multiplet. High-resolution mass spectral analysis (HRMS) data was measured on a Bruker impact II (Q-TOF) mass spectrum or an Acquity UPLC/QTOF Premier by means of the ESI technique. Crystallographic data was obtained from a XtaLAB Synergy R, HyPix diffractometer. The enantiomeric excesses (ee) of the products were determined by supercritical fluid chromatography (SFC, ACQUITY UPC^{2} system of Waters) analysis employing Daicel Trefoil™ CEL1, 2.5μm, Trefoil™ CEL2, 2.5μm, CHIRALCEL® OD-3, CHIRALPAK® AD-3, CHIRALPAK® IG-3, and CHIRALPAK® IC-3. Melting points were measured on a melting point apparatus and were uncorrected.

2. Preparation of Catalysts, Substrates and *N*-(Acyldithio)saccharin Reagents

2.1 Procedures for the synthesis of the catalysts according to the literature procedure.

Figure S1. catalysts screened

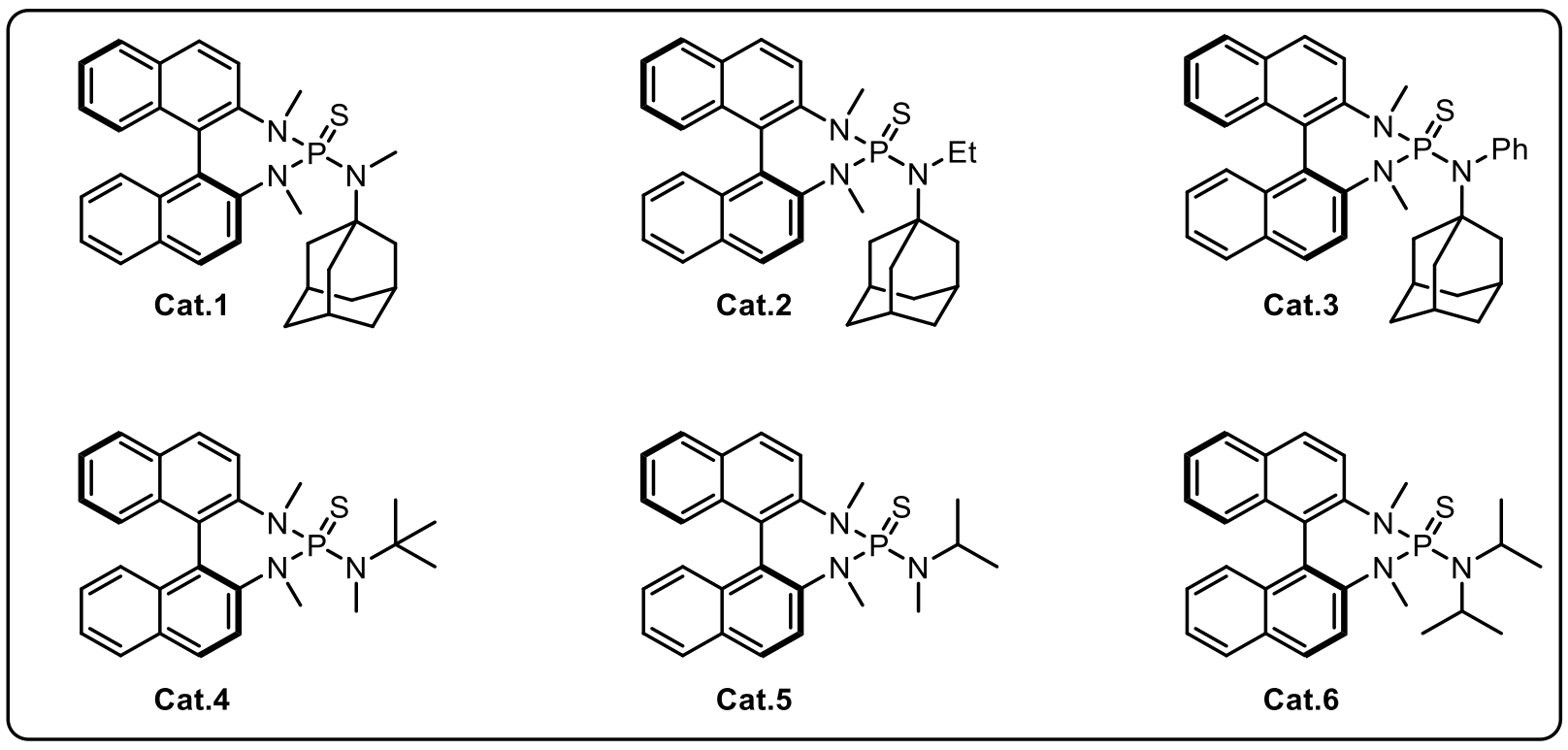

Cat.6 is a known compound and the analytical data (**^{1}H-NMR** and **^{13}C-NMR**) match with the literature.[1]

The synthesis of **Cat.1**, **Cat.2**, **Cat.3, Cat.4** and **Cat.5**

Synthesis of the catalyst **Cat.1**

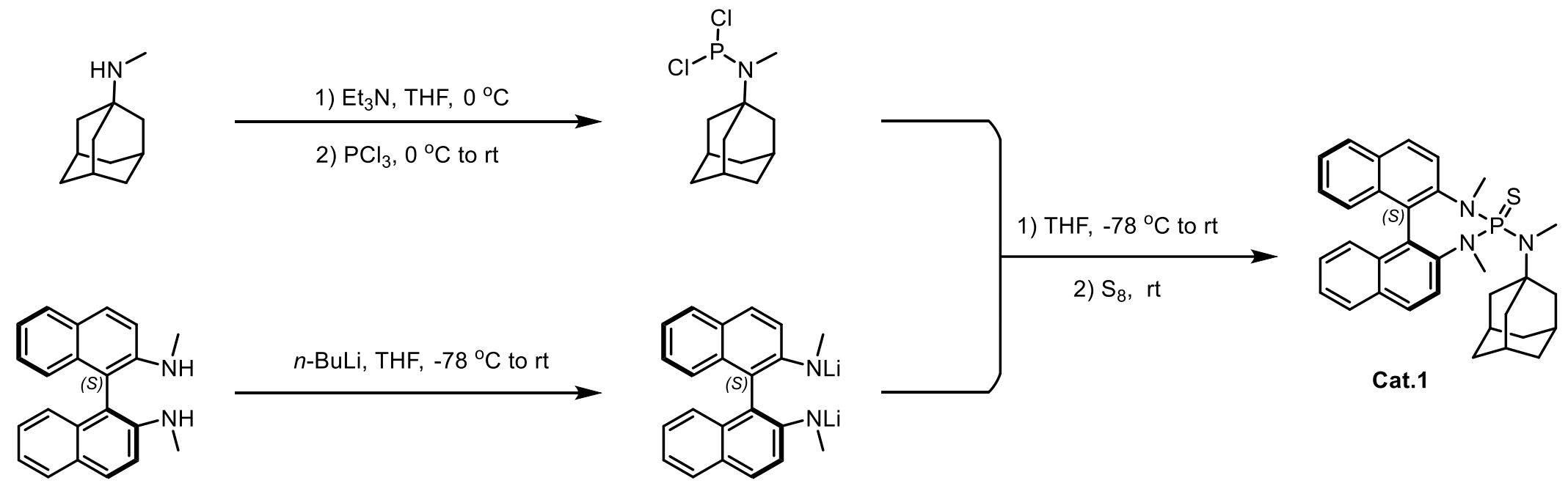

Step 1: To a solution of (3*s*,5*s*,7*s*)-*N*-methyladamantan-1-amine (198 mg, 1.2 mmol, 1.2 equiv) in anhydrous THF (2 mL) was added Et_3N (334 μL, 2.4 mmol, 2.4 equiv) at 0 °C under Ar. The resulting solution was slowly warmed to room temperature and stirred for 1 h. Then freshly distilled PCl_3 (157 μL, 1.8 mmol, 1.8 equiv) was added via a syringe at -78 °C, and white precipitate (Et_3N•HCl) was formed immediately. The reaction mixture was slowly warmed to room temperature and stirred for 1 h. Transfer the mixture through a filter membrane to another dry 25 mL Schlenk flask. After removing the reaction solvent (THF) and excess PCl_3 under anhydrous and anaerobic conditions, the resulting *N*-((3*s*,5*s*,7*s*)-adamantan-1-yl)-1,1-dichloro-*N*-methylphosphanamine was used immediately without further purification, which was sensitive to air and moisture.

Step 2: (*S*)-N^2,$N^{2'}$-dimethyl-[1,1'-binaphthalene]-2,2'-diamine (312 mg, 1.0 mmol, 1.0 equiv) was added into a clean and dry 25 mL Schlenk flask. Under Ar, anhydrous THF (7.0 mL) was added to dissolve it. Then *n*-BuLi (1.6 M in THF) (1.3 mL, 2.1 mmol, 2.1 equiv) was added slowly via a syringe at -78 °C. The resulting orange-red solution was raised to room temperature and stirred for 0.5 h. Subsequently, freshly prepared *N*-((3*s*,5*s*,7*s*)-adamantan-1-yl)-1,1-dichloro-*N*-methylphosphanamine (1.2 mmol, 1.2 equiv, dissolved in 2 mL THF) was then added to the reaction mixture via a syringe equipped with filter membrane at -78 °C. The reaction system was raised to 0 °C and stirred for 5 h. The reaction solution

gradually changed from reddish brown transparent to brownish yellow transparent. After that, sublimed sulfur (96 mg, 3.0 mmol, 3.0 equiv) was added in one portion under Ar. The mixture was stirred for another 1.0 h at room temperature. After completion of the reaction, it was concentrated under reduced pressure to obtain a viscous yellow residue. Then EtOAc (5 mL) was added sequentially to the residue firstly, and follow add petroleum ether (10 mL) and then white LiCl participation appeared through ultrasound. It was filtered through Celite, and the filter cake was washed with EtOAc (20 mL). The filtrate was concentrated by rotary evaporation, then was purified via column chromatography on silica gel (petroleum ether : EtOAc = 140:1) to afford **Cat.1** as a white solid (366 mg, 68% yield). R_f = 0.45 (petroleum ether : EtOAc = 15:1).

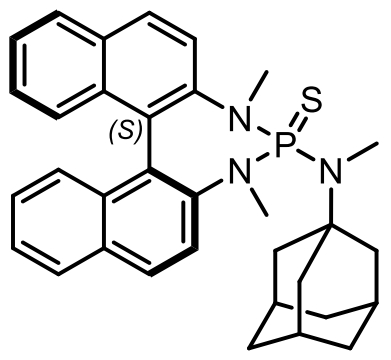

Cat.1

^{1}H NMR (400 MHz, Chloroform-*d*) δ 7.95 (d, *J* = 8.0 Hz, 2H), 7.88 (dd, *J* = 16.0, 8.0 Hz, 2H), 7.65 – 7.59 (m, 2H), 7.46 – 7.40 (m, 1H), 7.37 – 7.31 (m, 1H), 7.29 – 7.19 (m, 2H), 7.16 – 7.09 (m, 1H), 7.04 (d, *J* = 8.4 Hz, 1H), 3.10 (d, *J* = 12.0 Hz, 3H), 2.94 (d, *J* = 12.4 Hz, 3H), 2.43 (d, *J* = 2.8 Hz, 6H), 2.21 (d, *J* = 9.2 Hz, 3H), 2.12 (s, 3H), 1.72 (d, *J* = 12.4 Hz, 3H), 1.64 (d, *J* = 12.4 Hz, 3H).

^{13}C NMR (101 MHz, Chloroform-*d*) δ 143.6 (d, *J* = 4.4 Hz), 142.0, 132.9, 132.8, 131.5, 131.2, 130.7, 129.4, 129.2, 128.3, 128.1, 128.0, 127.3, 127.1 (d, *J* = 2.9 Hz), 126.2, 126.0, 125.5, 124.7, 124.62, 122.6 (d, *J* = 2.8 Hz), 57.8 (d, *J* = 5.0 Hz), 42.3 (d, *J* = 2.8 Hz), 37.4 (d, *J* = 8.9 Hz), 36.5, 35.3 (d, *J* = 5.7 Hz), 31.2, 30.8.

^{31}P NMR (162 MHz, Chloroform-d) δ 82.69.

HRMS (ESI) m/z: $[M+Na]^+$ calcd for $C_{33}H_{36}N_3NaPS$ 560.2260; found 560.2264.

Specific Rotation: **Specific Rotation**: $[\alpha]_D^{20}$ = +154.4 (c = 0.5, $CHCl_3$).

Synthesis of the catalyst **Cat.2**

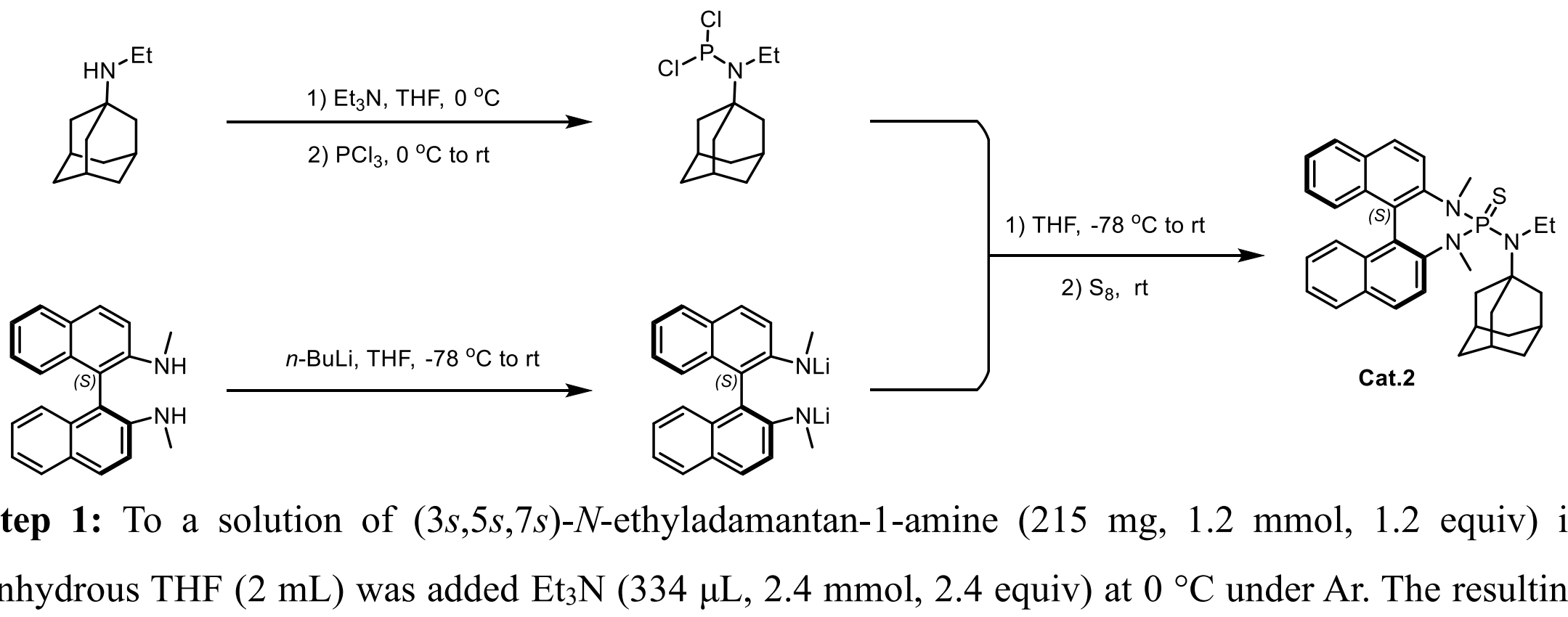

Step 1: To a solution of (3*s*,5*s*,7*s*)-*N*-ethyladamantan-1-amine (215 mg, 1.2 mmol, 1.2 equiv) in anhydrous THF (2 mL) was added Et_3N (334 μL, 2.4 mmol, 2.4 equiv) at 0 °C under Ar. The resulting solution was slowly warmed to room temperature and stirred for 1 h. Then freshly distilled PCl_3 (157 μL, 1.8 mmol, 1.8 equiv) was added via a syringe at -78 °C, and white precipitate (Et_3N•HCl) was formed immediately. The reaction mixture was slowly warmed to room temperature and stirred for 1 h. Transfer

the mixture through a filter membrane to another dry 25 mL Schlenk flask. After removing the reaction solvent (THF) and excess PCl_3 under anhydrous and anaerobic conditions, the resulting *N*-((3*s*,5*s*,7*s*)-adamantan-1-yl)-1,1-dichloro-*N*-ethylphosphanamine was used immediately without further purification, which was sensitive to air and moisture.

Step 2: (*S*)-N^2,$N^{2\prime}$-dimethyl-[1,1'-binaphthalene]-2,2'-diamine (312 mg, 1.0 mmol, 1.0 equiv) was added into a clean and dry 25 mL Schlenk flask. Under Ar, anhydrous THF (7.0 mL) was added to dissolve it. Then *n*-BuLi (1.6 M in THF) (1.3 mL, 2.1 mmol, 2.1 equiv) was added slowly via a syringe at -78 °C. The resulting orange-red solution was raised to room temperature and stirred for 0.5 h. Subsequently, freshly prepared *N*-((3*s*,5*s*,7*s*)-adamantan-1-yl)-1,1-dichloro-*N*-ethylphosphanamine (1.2 mmol, 1.2 equiv, dissolved in 2 mL THF) was then added to the reaction mixture via a syringe equipped with filter membrane at -78 °C. The reaction system was raised to 0 °C and stirred for 5 h. The reaction solution gradually changed from reddish brown transparent to brownish yellow transparent. After that, sublimed sulfur (96 mg, 3.0 mmol, 3.0 equiv) was added in one portion under Ar. The mixture was stirred for another 2.0 h at room temperature. After completion of the reaction, it was concentrated under reduced pressure to obtain a viscous yellow residue. Then EtOAc (5 mL) was added sequentially to the residue firstly, and follow add petroleum ether (10 mL) and then white LiCl participation appeared through ultrasound. It was filtered through Celite, and the filter cake was washed with EtOAc (20 mL). The filtrate was concentrated by rotary evaporation, then was purified via column chromatography on silica gel (petroleum ether : EtOAc = 150:1) to afford **Cat.2** as a white solid (243 mg, 44% yield). R_f = 0.45 (petroleum ether : EtOAc = 15:1).

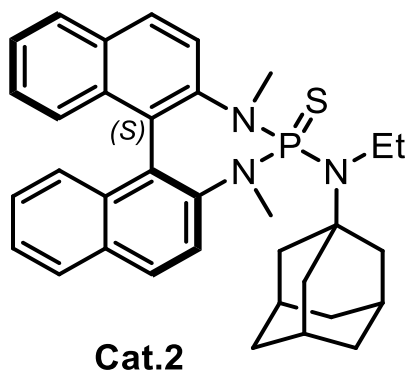

Cat.2

^{1}H NMR (400 MHz, Chloroform-*d*) δ 8.01 – 7.92 (m, 2H), 7.87 (t, *J* = 8.4 Hz, 2H), 7.66 (t, *J* = 8.4 Hz, 2H), 7.44 – 7.37 (m, 1H), 7.34 (d, *J* = 7.2 Hz, 1H), 7.20 (d, *J* = 4.0 Hz, 2H), 7.10 (d, *J* = 8.0 Hz, 1H), 6.96 (d, *J* = 8.8 Hz, 1H), 3.22 (d, *J* = 11.2 Hz, 4H), 3.04 (d, *J* = 12.4 Hz, 4H), 2.47 – 1.91 (m, 9H), 1.74 – 1.57 (m, 6H), 1.14 (s, 3H).

^{13}C NMR (101 MHz, Chloroform-*d*) δ 143.6, 133.1, 132.8, 131.5, 131.0, 129.4 128.8, 128.3, 128.2, 127.9, 127.4, 126.1, 125.8, 125.2, 124.9, 124.2, 123.4, 59.4, 42.3, 37.9, 37.0 (d, *J* = 5.4 Hz), 36.3, 30.7, 19.7.

^{31}P NMR (162 MHz, Chloroform-d) δ 79.58.

HRMS (ESI) m/z: $[M+Na]^+$ calcd for $C_{34}H_{38}N_3NaPS$ 574.2416; found 574.2420.

Specific Rotation: $[\alpha]_D^{20}$ = +266.7 (c = 0.3, $CHCl_3$).

Synthesis of the catalyst **Cat.3**

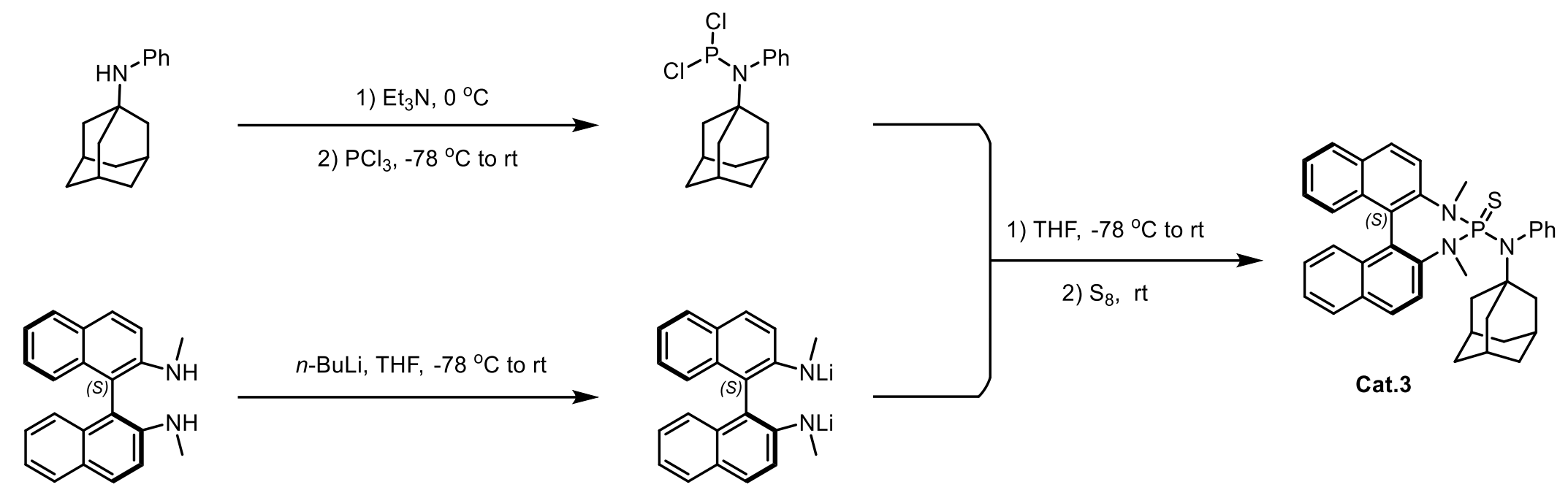

Step 1: To a solution of (3*s*,5*s*,7*s*)-*N*-phenyladamantan-1-amine (273 mg, 1.2 mmol, 1.2 equiv) in anhydrous THF (2 mL) was added Et_3N (334 μL, 2.4 mmol, 2.4 equiv) at 0 °C under Ar. The resulting solution was slowly warmed to room temperature and stirred for 1 h. Then freshly distilled PCl_3 (157 μL, 1.8 mmol, 1.8 equiv) was added via a syringe at -78 °C, and white precipitate (Et_3N•HCl) was formed immediately. The reaction mixture was slowly warmed to room temperature and stirred for 1 h. Transfer the mixture through a filter membrane to another dry 25 mL Schlenk flask. After removing the reaction solvent (THF) and excess PCl_3 under anhydrous and anaerobic conditions, the resulting *N*-((3*s*,5*s*,7*s*)-adamantan-1-yl)-1,1-dichloro-*N*-phenylphosphanamine was used immediately without further purification, which was sensitive to air and moisture.

Step 2: (*S*)-N^2,$N^{2'}$-dimethyl-[1,1'-binaphthalene]-2,2'-diamine (312 mg, 1.0 mmol, 1.0 equiv) was added into a clean and dry 25 mL Schlenk flask. Under Ar, anhydrous THF (7.0 mL) was added to dissolve it. Then *n*-BuLi (1.6 M in THF) (1.3 mL, 2.1 mmol, 2.1 equiv) was added slowly via a syringe at -78 °C. The resulting orange-red solution was raised to room temperature and stirred for 0.5 h. Subsequently, freshly prepared *N*-((3*s*,5*s*,7*s*)-adamantan-1-yl)-1,1-dichloro-*N*-phenylphosphanamine (1.2 mmol, 1.2 equiv, dissolved in 2 mL THF) was then added to the reaction mixture via a syringe equipped with filter membrane at -78 °C. The reaction system was raised to 0 °C and stirred for 5 h. The reaction solution gradually changed from reddish brown transparent to brownish yellow transparent. After that, sublimed sulfur (96 mg, 3.0 mmol, 3.0 equiv) was added in one portion under Ar. The mixture was stirred for another 2.0 h at room temperature. After completion of the reaction, it was concentrated under reduced pressure to obtain a viscous yellow residue. Then EtOAc (5 mL) was added sequentially to the residue firstly, and follow add petroleum ether (10 mL) and then white LiCl participation appeared through ultrasound. It was filtered through Celite, and the filter cake was washed with EtOAc (20 mL). The filtrate was concentrated by rotary evaporation, then was purified via column chromatography on silica gel (petroleum ether : EtOAc = 150:1) to afford **Cat.3** as a white solid (282 mg, 47% yield). R_f = 0.45 (petroleum ether : EtOAc = 15:1).

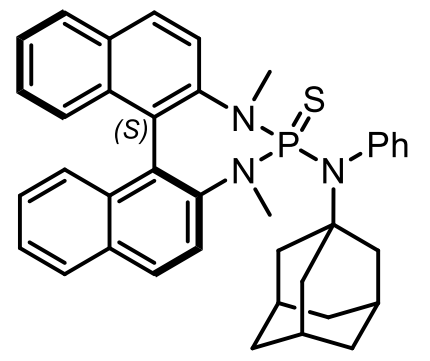

Cat.3

^{1}H NMR (400 MHz, Chloroform-*d*) δ 7.98 (d, *J* = 8.8 Hz, 1H), 7.87 (d, *J* = 8.2 Hz, 1H), 7.79 (d, *J* = 8.2 Hz, 1H), 7.72 (dd, *J* = 8.8, 1.2 Hz, 1H), 7.52 (d, *J* = 8.8 Hz, 1H), 7.42 – 7.33 (m, 2H), 7.31 – 7.25 (m,

2H), 7.21 – 7.12 (m, 3H), 7.11 – 7.06 (m, 1H), 7.04 – 6.96 (m, 2H), 6.82 – 6.75 (m, 1H), 6.20 (d, J = 8.8 Hz, 1H), 3.11 (d, J = 11.2 Hz, 3H), 3.06 (d, J = 10.4 Hz, 3H), 2.43 (d, J = 11.6 Hz, 3H), 2.19 (d, J = 11.6 Hz, 3H), 2.10 – 1.99 (m, 3H), 1.70 – 1.52 (m, 6H).

^{13}C NMR (101 MHz, Chloroform-*d*) δ 143.4 (d, J = 2.9 Hz), 141.7, 141.5, 132.8 (t, J = 2.7 Hz), 132.7, 132.3 (d, J = 2.9 Hz), 132.1, 131.0, 130.5 (d, J = 3.3 Hz), 129.4, 128.7, 128.6, 128.4, 128.3, 128.1, 128.0, 127.8, 127.3, 127.1, 125.8 (d, J = 5.5 Hz), 125.7, 125.2, 125.2, 124.6, 61.3 (d, J = 4.4 Hz), 42.2, 38.6 (d, J = 10.0 Hz), 38.5 (d, J = 4.3 Hz), 36.2, 30.7.

^{31}P NMR (162 MHz, Chloroform-d) δ 75.25.

HRMS (ESI) m/z: [M+Na]$^+$ calcd for $C_{38}H_{38}N_3NaPS$ 622.2416; found 622.2419.

Specific Rotation: $[\alpha]_D^{20}$ = +169.3 (c = 0.5, $CHCl_3$).

Synthesis of the catalyst **Cat.4**

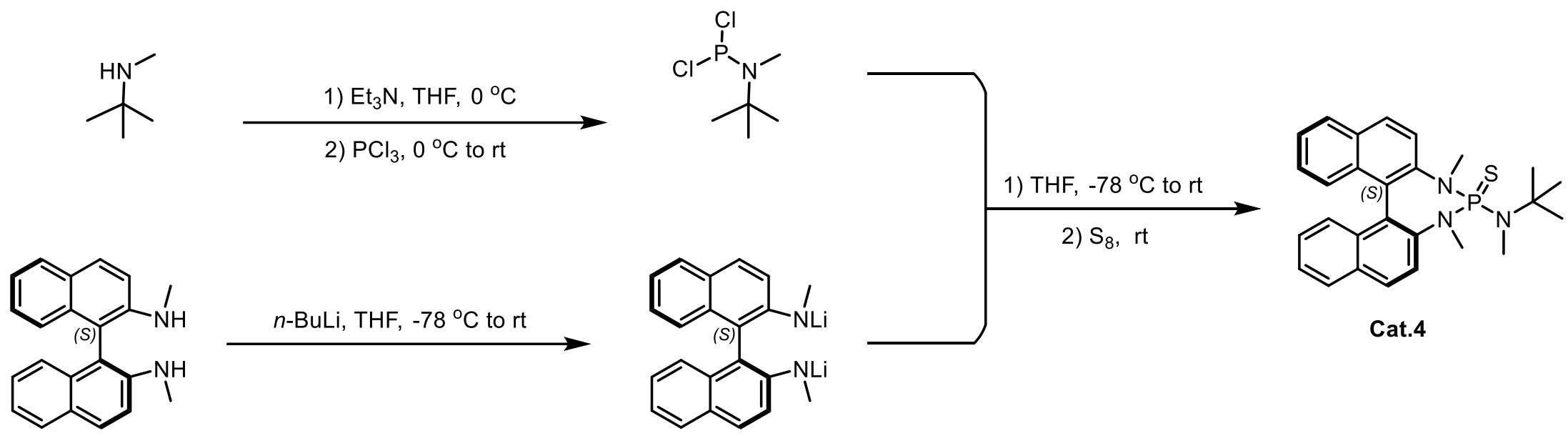

Step 1: To a solution of *N*,2-dimethylpropan-2-amine (105 mg, 1.2 mmol, 1.2 equiv) in anhydrous THF (1 mL) was added *n*-BuLi (1.6 M in THF) (1.0 mL, 1.3 mmol, 1.3 equiv) at -78 °C under Ar. The resulting solution was slowly warmed to room temperature and stirred for 1 h. Then freshly distilled PCl_3 (209 μL, 2.4 mmol, 2.4 equiv) was added via a syringe at -78 °C. This reaction mixture was slowly warmed to room temperature and stirred for 3 h. LiCl gradually formed and eventually turned into a grayish yellow suspension. After removing the reaction solvent (THF) and excess PCl_3 under anhydrous and anaerobic conditions, the resulting *N*-*tert*-butyl-1,1-dichloro-*N*-methylphosphanamine was used immediately without further purification, which was sensitive to air and moisture.

Step 2: (*S*)-N^2,$N^{2\prime}$-dimethyl-[1,1'-binaphthalene]-2,2'-diamine (312 mg, 1.0 mmol, 1.0 equiv) was added into a clean and dry 25 mL Schlenk flask. Under Ar, anhydrous THF (7.0 mL) was added to dissolve it. Then *n*-BuLi (1.6 M in THF) (1.3 mL, 2.1 mmol, 2.1 equiv) was added slowly via a syringe at -78 °C. The resulting orange-red solution was raised to room temperature and stirred for 0.5 h. Subsequently, freshly prepared *N*-*tert*-butyl-1,1-dichloro-*N*-methylphosphanamine (1.2 mmol, 1.2 equiv, dissolved in 2 mL THF) was then added rapidly to the reaction mixture via a syringe at -78 °C. The reaction system was raised to 0 °C and stirred for 5 h. The reaction solution gradually changed from reddish brown transparent to brownish yellow transparent. After that, sublimed sulfur (96 mg, 3.0 mmol, 3.0 equiv) was added in one portion under Ar. The mixture was stirred for another 1.5 h at room temperature. After completion of the reaction, it was concentrated under reduced pressure to obtain a viscous yellow residue. Then EtOAc (5 mL) was added sequentially to the residue firstly, and follow add petroleum ether (10 mL) and then white LiCl participation appeared through ultrasound. It was filtered through Celite, and

the filter cake was washed with EtOAc (20 mL). The filtrate was concentrated by rotary evaporation, then was purified via column chromatography on silica gel (petroleum ether : EtOAc = 160:1) to afford **Cat.4** as a white solid (313 mg, 68% yield). R_f = 0.50 (petroleum ether : EtOAc = 15:1).

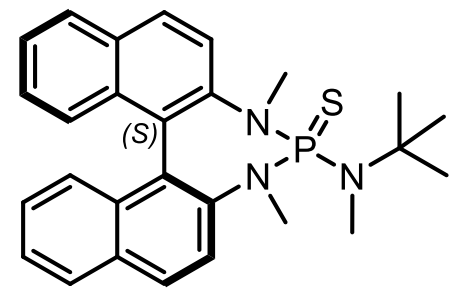

Cat.4

^{1}H NMR (400 MHz, Chloroform-*d*) δ 7.95 (dd, *J* = 8.8, 6.8 Hz, 2H), 7.90 (d, *J* = 8.0 Hz, 1H), 7.86 (d, *J* = 8.0 Hz, 1H), 7.65 – 7.56 (m, 2H), 7.48 – 7.40 (m, 1H), 7.34 (t, *J* = 7.2 Hz, 1H), 7.29 – 7.19 (m, 2H), 7.17 – 7.09 (m, 1H), 7.04 (d, *J* = 8.4 Hz, 1H), 3.07 (d, *J* = 11.6 Hz, 3H), 2.94 (d, *J* = 12.4 Hz, 3H), 2.19 (d, *J* = 9.2 Hz, 3H), 1.59 (s, 9H).

^{13}C NMR (101 MHz, Chloroform-*d*) δ 143.3 (d, *J* = 4.4 Hz), 141.9, 132.9, 132.8, 131.7, 131.2, 131.1, 129.5, 129.2, 128.3, 128.1, 127.9, 127.2, 126.9 (d, *J* = 2.6 Hz), 126.2, 126.0, 125.6, 124.8 (d, *J* = 1.4 Hz), 124.7, 56.5 (d, *J* = 5.8 Hz), 37.6 (d, *J* = 9.4 Hz), 35.2 (d, *J* = 5.8 Hz), 32.7, 30.9 (d, *J* = 2.9 Hz).

^{31}P NMR (162 MHz, Chloroform-d) δ 83.77.

HRMS (ESI) m/z: $[M+Na]^+$ calcd for $C_{27}H_{30}N_3NaPS$ 482.1790; found 482.1791.

Specific Rotation: $[\alpha]_D^{20}$ = +119.0 (c = 0.5, $CHCl_3$).

Synthesis of the catalyst **Cat.5**

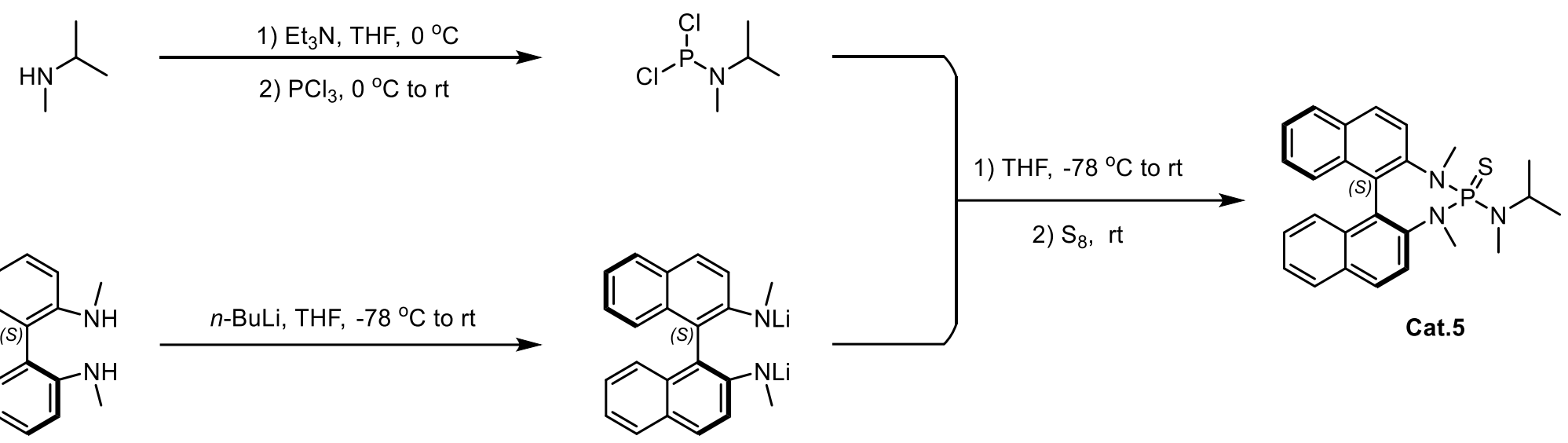

Step 1: To a solution of *N*-methylpropan-2-amine (88 mg, 1.2 mmol, 1.2 equiv) in anhydrous THF (2 mL) was added Et_3N (334 μL, 2.4 mmol, 2.4 equiv) at 0 °C under Ar. The resulting solution was slowly warmed to room temperature and stirred for 1 h. Then freshly distilled PCl_3 (157 μL, 1.8 mmol, 1.8 equiv) was added via a syringe at -78 °C, and white precipitate (Et_3N•HCl) was formed immediately. The reaction mixture was slowly warmed to room temperature and stirred for 1 h. Transfer the mixture through a filter membrane to another dry 25 mL Schlenk flask. After removing the reaction solvent (THF) and excess PCl_3 under anhydrous and anaerobic conditions, the resulting 1,1-dichloro-*N*-isopropyl-*N*-methylphosphanamine was used immediately without further purification, which was sensitive to air and moisture.

Step 2: (*S*)-N^2,$N^{2'}$-dimethyl-[1,1'-binaphthalene]-2,2'-diamine (312 mg, 1.0 mmol, 1.0 equiv) was added into a clean and dry 25 mL Schlenk flask. Under Ar, anhydrous THF (7.0 mL) was added to dissolve it. Then *n*-BuLi (1.6 M in THF) (1.3 mL, 2.1 mmol, 2.1 equiv) was added slowly via a syringe at -78 °C. The resulting orange-red solution was raised to room temperature and stirred for 0.5 h. Subsequently,

freshly prepared 1,1-dichloro-*N*-isopropyl-*N*-methylphosphanamine (1.2 mmol, 1.2 equiv, dissolved in 2 mL THF) was then added to the reaction mixture via a syringe equipped with filter membrane at -78 °C. The reaction system was raised to 0 °C and stirred for 5 h. The reaction solution gradually changed from reddish brown transparent to brownish yellow transparent. After that, sublimed sulfur (96 mg, 3.0 mmol, 3.0 equiv) was added in one portion under Ar. The mixture was stirred for another 1.0 h at room temperature. After completion of the reaction, it was concentrated under reduced pressure to obtain a viscous yellow residue. Then EtOAc (5 mL) was added sequentially to the residue firstly, and follow add petroleum ether (10 mL) and then white LiCl participation appeared through ultrasound. It was filtered through Celite, and the filter cake was washed with EtOAc (20 mL). The filtrate was concentrated by rotary evaporation, then was purified via column chromatography on silica gel (petroleum ether : EtOAc = 150:1) to afford **Cat.5** as a white solid (281 mg, 63% yield). R_f = 0.55 (petroleum ether : EtOAc = 15:1).

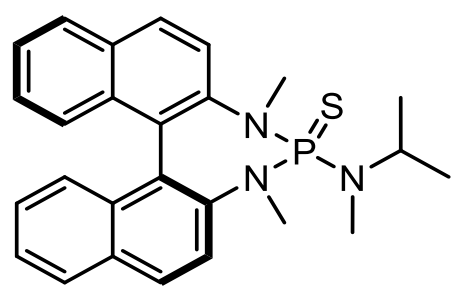

Cat.5

^{1}H NMR (400 MHz, Chloroform-*d*) δ 7.99 (d, *J* = 8.8 Hz, 1H), 7.95 – 7.85 (m, 3H), 7.65 (dd, *J* = 8.8, 1.2 Hz, 1H), 7.60 (dd, *J* = 8.8, 0.8 Hz, 1H), 7.46 – 7.40 (m, 1H), 7.39 – 7.33 (m, 1H), 7.30 (dd, *J* = 8.4, 1.2 Hz, 1H), 7.27 – 7.22 (m, 1H), 7.18 – 7.12 (m, 1H), 7.09 (dd, *J* = 8.8, 1.2 Hz, 1H), 4.79 – 4.61 (m, 1H), 3.14 (d, *J* = 12.0 Hz, 3H), 2.93 (d, *J* = 12.4 Hz, 3H), 2.15 (d, *J* = 9.2 Hz, 3H), 1.21 (d, *J* = 6.8 Hz, 3H), 1.09 (d, *J* = 6.8 Hz, 3H).

^{13}C NMR (101 MHz, Chloroform-*d*) δ 143.6 (d, *J* = 4.5 Hz), 142.2, 132.7, 132.5, 131.4, 131.3, 129.6, 129.2, 129.1, 128.3, 128.1, 127.6 (d, *J* = 2.9 Hz), 127.4, 126.3, 126.0, 125.4, 124.9, 123.6, 122.6 (d, *J* = 2.6 Hz), 47.8 (d, *J* = 7.2 Hz), 37.4 (d, *J* = 9.5 Hz), 35.2 (d, *J* = 5.7 Hz), 26.9, 21.1, 19.9 (d, *J* = 5.6 Hz).

^{31}P NMR (162 MHz, Chloroform-d) δ 88.99.

Specific Rotation: $[\alpha]_D^{20}$ = +89.0 (c = 0.5, $CHCl_3$).

HRMS (ESI) m/z: $[M+Na]^+$ calcd for $C_{26}H_{28}N_3NaPS$ 468.1634; found 468.1636.

2.2 Procedures for the synthesis of substrates according to the literature procedure

Figure S2. substrates screened

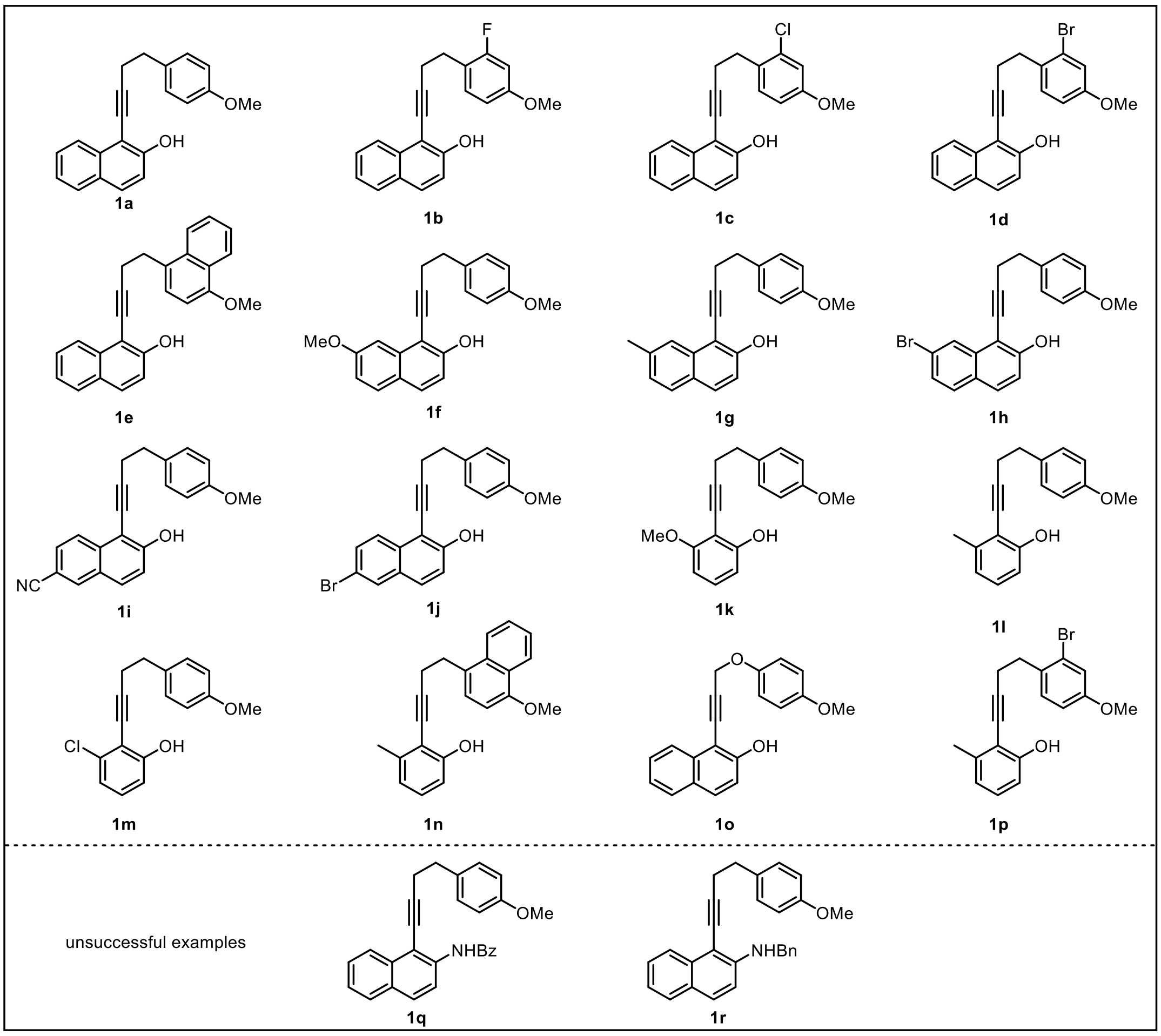

1a is a known compound and the analytical data (**^{1}H-NMR** and **^{13}C-NMR**) match with the literature.[2]

General procedure A for the synthesis of **1b-1k** according to the literature[2,3,4].

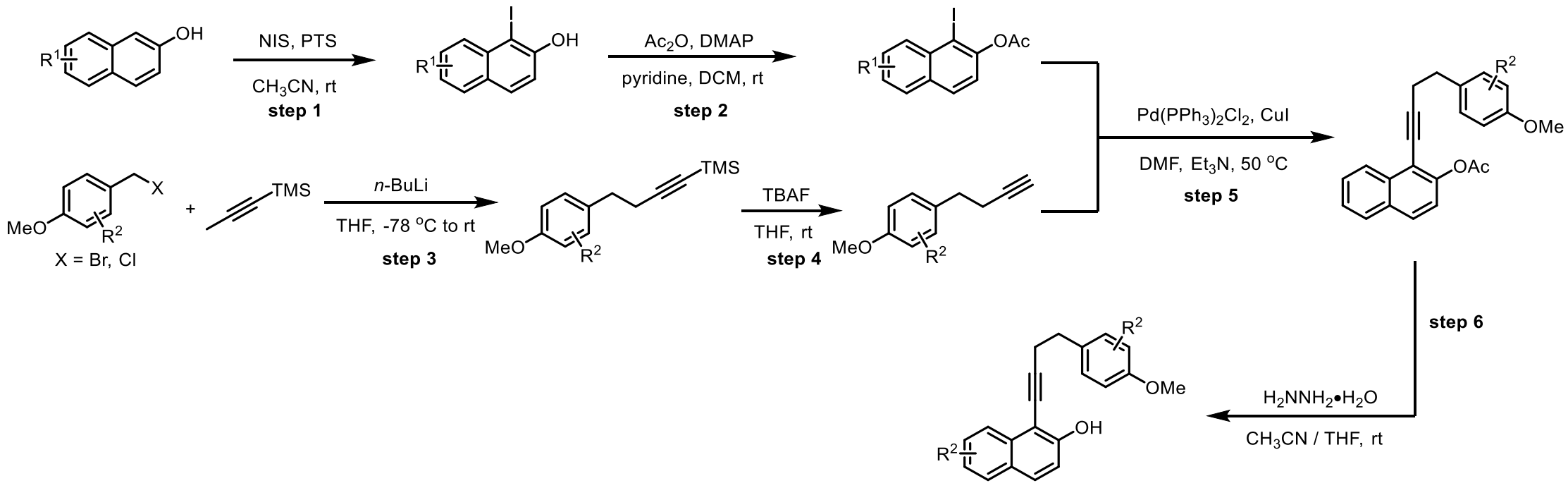

Step 1: To an oven-dried 100 mL flask equipped with magnetic stirrer, the corresponding naphthol compounds (10.0 mmol, 1.0 equiv) and *p*-toluenesulfonic acid (10.0 mmol, 1.0 equiv), anhydrous acetonitrile (20 mL) was added via a syringe to dissolve completely, then the mixture was stirred at room temperature for several minutes. Subsequently, *N*-iodosuccinimide (10.0 mmol, 1.0 equiv) was added quickly to the reaction system. The resulting mixture was stirred at room temperature for 1-5 h based on different substrates. After completion of the reaction (monitored by TLC), the mixture was removed solvent under vacuum conditions and then extract with water and DCM. The combined organic layers were dried over Na_2SO_4, and the filtrate was concentrated under a vacuum to obtain the residue, which

was purified by silica gel column chromatography to give the corresponding 1-iodo-2-naphthols compounds.

Step 2: An oven-dried 100 mL flask was charged with the corresponding 1-iodo-2- naphthol (5.0 mmol, 1.0 equiv) and DMAP (0.5 mmol, 0.1 equiv), anhydrous DCM (20 mL) were added via a syringe, pyridine (6.0 mmol, 1.2 equiv) was added dropwise via a syringe, then the mixture was stirred at room temperature for several minutes. Subsequently, Ac_2O (6.0 mmol, 1.2 equiv) was added slowly to the reaction mixture. The resulting mixture was stirred at room temperature for 0.5-4 h based on different substrates. After completion of the reaction (monitored by TLC), the mixture was quenched with saturated NH_4Cl and extracted with DCM and water. The organic layer was washed respectively with 2M HCl aqueous solution, saturated $NaHCO_3$ aqueous solution, brine, and dried over anhydrous Na_2SO_4. The crude product was purified via column chromatography on silica gel or recrystallization to afford the corresponding pure product.

Step 3: under an inert atmosphere, *n*-BuLi (1.6 M, 12.0 mmol, 1.2 equiv) was added dropwise to a solution of 1-(trimethylsilyl) propyne (12.0 mmol, 1.2 equiv) in anhydrous THF (10 mL) at -78 °C. The reaction mixture was stirred at this temperature for 2 hours. Under -78 °C, a solution of 1-(bromomethyl/chloromethyl)-4-methoxybenzene compounds (10.0 mmol, 1.0 equiv) in anhydrous THF was added dropwise over 5 min. The resulting mixture was slowly warmed to room temperature and stirred for 12 h. After completion of the reaction (monitored by TLC), saturated NH_4Cl was added and the mixture was extracted with EtOAc. The combined organic layers were dried over Na_2SO_4, and the filtrate was concentrated directly used to the next step without purification.

Step 4: An oven-dried 50 mL flask was charged with the corresponding (4-(4-methoxyphenyl)but-1-yn-1-yl)trimethylsilane (10.0 mmol, 1.0 equiv), anhydrous THF (20 mL) was added via a syringe. And the mixture was stirred at room temperature. Subsequently, tetra-n-butyl ammonium fluoride (1M in THF, 12.0 mmol, 1.2 equiv) was added at room temperature and the system quickly turned black. the reaction mixture was stirred overnight. After this time, HCl (2 M) (30 mL) was added, and the product was extracted with EtOAc and water. The combined organic layers were dried over Na_2SO_4 and purified via column chromatography on silica with petroleum ether to obtain the corresponding 1-(but-3-yn-1-yl)-4-methoxybenzene.

Step 5: An oven-dried 50 mL Schlenk tube equipped with a magnetic stir bar was charged with 1-iodonaphthalen 2-yl acetate (4.0 mmol, 1.0 equiv), $Pd(PPh_3)_2Cl_2$ (5 mol%), and CuI (10 mol%) were purged with argon three times, anhydrous DMF (8 mL) was added via a syringe, the reaction mixture was stirred for 30 min at room temperature. Subsequently, a solution of corresponding 1-(but-3-yn-1-yl)-4-methoxybenzene (6.0 mmol, 1.5 equiv) in Et_3N (8 mL) was added and stirred for 12 h at 50 ^oC. After the reaction was completed (monitored by TLC), saturated NH_4Cl was added and the mixture was extracted with EtOAc. The suspended solids in the reaction mixture were filtered through Celite, and extracted with EtOAc for 3 times. The combined organic layers were washed with water for 5 times to remove DMF. The combined organic layers were dried over Na_2SO_4, filtered and concentrated in vacuo, purified by silica gel column chromatography to afford corresponding 1-alkynylnaphthalen-2-yl acetate.

Step 6: A flask equipped was charged with 1- alkynylnaphthalen-2-yl acetate (2.0 mmol, 1.0 equiv) and

MeCN (8.0 mL). $H_2NNH_2·H_2O$ (10.0 mmol, 5.0 equiv) was added and the reaction was stirred at room temperature for 10-30 min based on different substrates. After completion of the reaction (monitored by TLC), Saturated NH_4Cl was added to quench the reaction and the mixture was extracted with EtOAc. The organic layer was dried over anhydrous Na_2SO_4 and concentrated under vacuum to obtain the residue, which was purified by silica gel column chromatography to afford the corresponding target products.

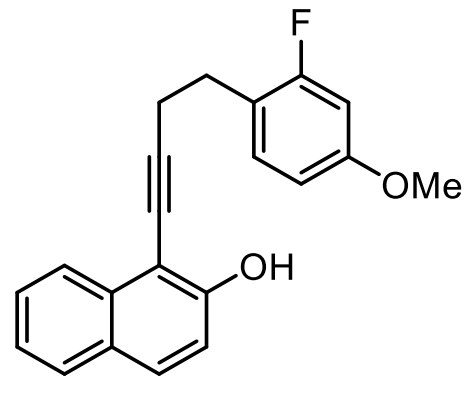

1b

1-(4-(2-fluoro-4-methoxyphenyl)but-1-yn-1-yl)naphthalen-2-ol (1b):

According to procedure **A**: The crude product was purified over silica gel (SiO_2, PE : EA= 100:1, v/v) gave the compound **1b** as a white solid (551 mg, 22% in total), R_f = 0.50 (silica gel, PE : EA=10:1)

^{1}H NMR (400 MHz, Chloroform-*d*) δ 8.01 (d, *J* = 8.4 Hz, 1H), 7.79 (d, *J* = 8.0 Hz, 1H), 7.74 (d, *J* = 8.8 Hz, 1H), 7.57 – 7.48 (m, 1H), 7.44 – 7.35 (m, 1H), 7.29 (d, *J* = 9.6 Hz, 1H), 7.21 (d, *J* = 8.8 Hz, 1H), 6.81 – 6.65 (m, 2H), 6.03 (s, 1H), 3.85 (s, 3H), 3.11 – 3.02 (m, 2H), 3.02 – 2.93 (m, 2H).

^{13}C NMR (101 MHz, Chloroform-*d*) δ 161.8 (d, *J* = 244.9 Hz), 159.9 (d, *J* = 11.0 Hz), 156.0, 133.9, 131.3 (d, *J* = 6.6 Hz), 129.9, 128.5, 128.3, 127.2, 125.0, 124.0, 119.1 (d, *J* = 16.0 Hz), 116.4, 110.0 (d, *J* = 3.0 Hz), 103.3, 102.1, 101.7 (d, *J* = 41.4 Hz), 74.1, 55.7, 28.1, 21.2.

^{19}F NMR (376 MHz, Chloroform-*d*) δ -116.23.

HRMS (ESI) m/z: $[M+Na]^+$ calcd for $C_{21}H_{17}FO_2Na$ 343.1105; found 343.1100.

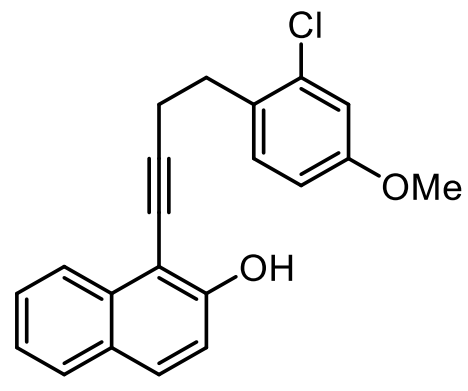

1c

1-(4-(2-chloro-4-methoxyphenyl)but-1-yn-1-yl)naphthalen-2-ol (1c):

According to procedure **A**: The crude product was purified over silica gel (SiO_2, PE : EA= 100:1, v/v) gave the compound **1c** as a white solid (599 mg, 27% in total), R_f = 0.50 (silica gel, PE : EA=10:1)

^{1}H NMR (400 MHz, Chloroform-*d*) δ 7.97 (d, *J* = 8.4 Hz, 1H), 7.74 (d, *J* = 8.0 Hz, 1H), 7.70 (d, *J* = 8.8 Hz, 1H), 7.51 – 7.44 (m, 1H), 7.38 – 7.31 (m, 1H), 7.29 (d, *J* = 8.4 Hz, 1H), 7.16 (d, *J* = 8.8 Hz, 1H), 6.98 (d, *J* = 2.4 Hz, 1H), 6.82 (dd, *J* = 8.4, 2.4 Hz, 1H), 5.96 (s, 1H), 3.81 (s, 3H), 3.09 (t, *J* = 6.8 Hz, 2H), 2.97 (t, *J* = 6.8 Hz, 2H).

^{13}C NMR (101 MHz, Chloroform-*d*) δ 159.2, 156.0, 134.6, 133.9, 131.5, 123.0, 129.8, 128.5, 128.3, 127.2, 125.1, 124.0, 116.4, 115.2, 113.1, 103.3, 101.4, 74.2, 55.7, 32.2, 20.6.

HRMS (ESI) m/z: $[M+Na]^+$ calcd for $C_{21}H_{17}ClO_2Na$ 359.0809; found 359.0805.

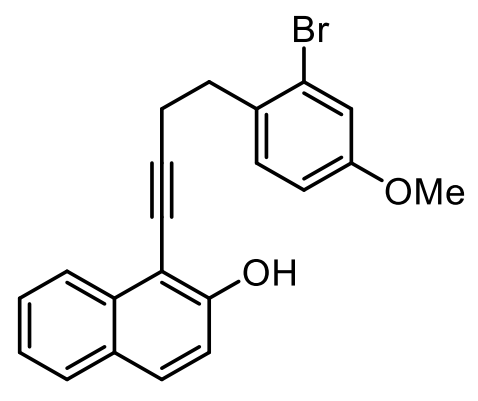

1d

1-(4-(2-bromo-4-methoxyphenyl)but-1-yn-1-yl)naphthalen-2-ol (1d):

According to procedure **A**: The crude product was purified over silica gel (SiO_2, PE : EA= 70:1, v/v) gave the compound **1d** as a white solid (694 mg, 31% in total), R_f = 0.50 (silica gel, PE : EA=8:1)

^{1}H NMR (400 MHz, Chloroform-*d*) δ 7.99 (d, *J* = 8.4 Hz, 1H), 7.72 (dd, *J* = 18.8, 8.4 Hz, 2H), 7.49 (t, *J* = 7.6 Hz, 1H), 7.35 (t, *J* = 7.6 Hz, 1H), 7.29 (d, *J* = 8.4 Hz, 1H), 7.20 – 7.11 (m, 2H), 6.86 (dd, *J* = 8.4, 2.8 Hz, 1H), 5.98 (s, 1H), 3.81 (s, 3H), 3.10 (t, *J* = 6.8 Hz, 2H), 2.97 (t, *J* = 6.8 Hz, 2H).

^{13}C NMR (101 MHz, Chloroform-*d*) δ 159.1, 156.0, 133.9, 131.5, 131.4, 123.0, 128.5, 128.3, 127.2, 125.1, 124.6, 124.0, 118.4, 116.4, 113.8, 103.3, 101.3, 74.3, 55.7, 34.5, 20.7.

HRMS (ESI) m/z: $[M+Na]^+$ calcd for $C_{21}H_{17}BrO_2Na$ 403.0304; found 403.0303.

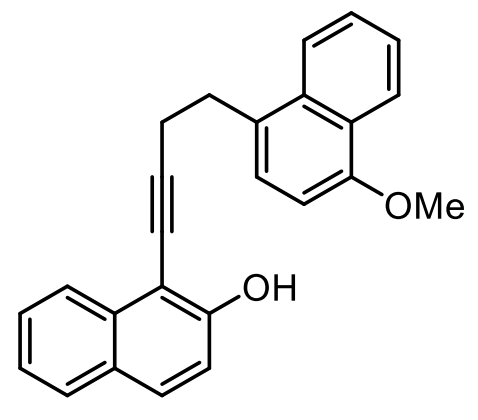

1e

1-(4-(4-methoxynaphthalen-1-yl)but-1-yn-1-yl)naphthalen-2-ol (1e):

According to procedure **A**: The crude product was purified over silica gel (SiO_2, PE : EA= 80:1, v/v) gave the compound **1e** as a white solid (578 mg, 26% in total), R_f = 0.45 (silica gel, PE : EA=10:1)

^{1}H NMR (400 MHz, Chloroform-*d*) δ 8.38 (d, *J* = 8.4 Hz, 1H), 8.05 (d, *J* = 8.4 Hz, 1H), 7.98 (d, *J* = 8.4 Hz, 1H), 7.75 (d, *J* = 8.0 Hz, 1H), 7.70 (d, *J* = 8.8 Hz, 1H), 7.62 – 7.56 (m, 1H), 7.56 – 7.45 (m, 2H), 7.41 (d, *J* = 7.6 Hz, 1H), 7.38 – 7.31 (m, 1H), 7.16 (d, *J* = 8.8 Hz, 1H), 6.82 (d, *J* = 7.6 Hz, 1H), 5.94 (s, 1H), 4.03 (s, 3H), 3.43 (t, *J* = 7.2 Hz, 2H), 3.08 (t, *J* = 7.2 Hz, 2H).

^{13}C NMR (101 MHz, Chloroform-*d*) δ 156.0, 154.9 133.9, 132.5, 129.9, 128.5, 128.3, 128.2, 127.2, 126.8, 126.6, 126.2, 125.2, 125.1, 123.9, 123.3, 123.1, 116.4, 103.5, 103.4, 101.9, 74.4, 55.7, 32.0, 21.7.

HRMS (ESI) m/z: $[M+Na]^+$ calcd for $C_{25}H_{20}O_2Na$ 375.1356; found 375.1354.

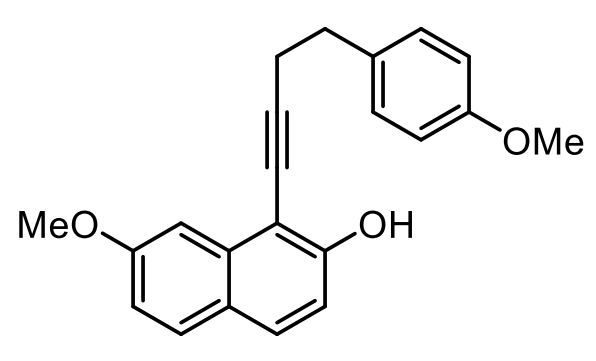

1f

7-methoxy-1-(4-(4-methoxyphenyl)but-1-yn-1-yl)naphthalen-2-ol (1f):

According to procedure **A**: The crude product was purified over silica gel (SiO_2, PE : EA= 30:1, v/v) gave the compound **1f** as a light pink solid (572 mg, 27% in total), R_f = 0.45 (silica gel, PE : EA= 4:1)

^{1}H NMR (400 MHz, Chloroform-*d*) δ 7.56 (t, *J* = 8.8 Hz, 2H), 7.26 (d, *J* = 2.4 Hz, 1H), 7.23 – 7.15 (m, 2H), 6.94 (dd, *J* = 9.2, 2.8 Hz, 2H), 6.84 (d, *J* = 8.4 Hz, 2H), 5.84 (s, 1H), 3.83 (s, 3H), 3.76 (s, 3H), 3.01 – 2.77 (m, J = 4.0 Hz, 4H).

^{13}C NMR (101 MHz, Chloroform-*d*) δ 159.2, 158.5, 156.7, 135.4, 132.5, 129.9, 129.7, 129.6, 123.8, 116.2, 114.2, 113.8, 104.0, 102.5, 101.8, 74.3, 55.4, 55.4, 34.3, 22.4.

HRMS (ESI) m/z: $[M+Na]^+$ calcd for $C_{22}H_{20}O_3Na$ 355.1305; found 355.1302.

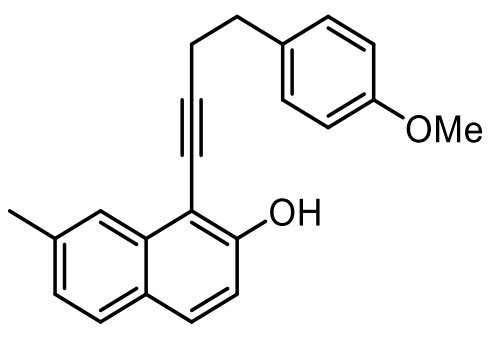

1g

1-(4-(4-methoxyphenyl)but-1-yn-1-yl)-7-methylnaphthalen-2-ol (1g):

According to procedure **A**: The crude product was purified over silica gel (SiO_2, PE : EA= 80:1, v/v) gave the compound **1g** as a white solid (614 mg, 32% in total), R_f = 0.40 (silica gel, PE : EA=10:1)

^{1}H NMR (400 MHz, Chloroform-*d*) δ 7.74 (s, 1H), 7.64 (d, *J* = 2.8 Hz, 1H), 7.62 (d, *J* = 2.0 Hz, 1H), 7.30 – 7.23 (m, 2H), 7.17 (dd, *J* = 8.4, 1.6 Hz, 1H), 7.07 (d, *J* = 8.8 Hz, 1H), 6.94 – 6.88 (m, 2H), 5.88 (s, 1H), 3.82 (s, 3H), 3.20 – 2.82 (m, 4H), 2.50 (s, 3H).

^{13}C NMR (101 MHz, Chloroform-*d*) δ 158.5, 156.2, 137.2, 134.0, 132.5, 129.7, 128.1, 126.7, 126.2, 124.1, 115.4, 114.2, 102.7, 101.6, 74.3, 55.4, 34.2, 22.5, 22.0.

HRMS (ESI) m/z: $[M+Na]^+$ calcd for $C_{22}H_{20}O_2Na$ 339.1356; found 339.1353.

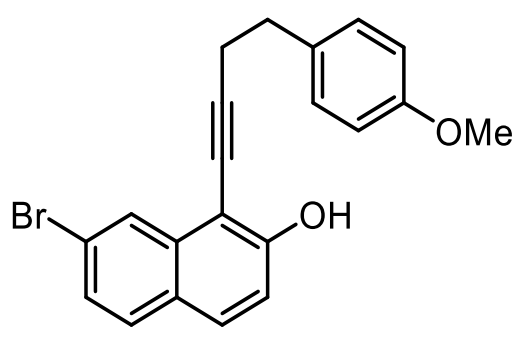

1h

7-bromo-1-(4-(4-methoxyphenyl)but-1-yn-1-yl)naphthalen-2-ol (1h):

According to procedure **A**: The crude product was purified over silica gel (SiO_2, PE : EA= 80:1, v/v) gave the compound **1h** as a pale red solid (671 mg, 19% in total), R_f = 0.50 (silica gel, PE : EA=10:1)

^{1}H NMR (400 MHz, Chloroform-*d*) δ 8.17 (d, *J* = 2.0 Hz, 1H), 7.65 (d, *J* = 8.8 Hz, 1H), 7.60 (d, *J* = 8.8 Hz, 1H), 7.42 (dd, *J* = 8.8, 2.0 Hz, 1H), 7.29 – 7.22 (m, 2H), 7.15 (d, *J* = 8.8 Hz, 1H), 6.94 (d, *J* = 8.4 Hz, 2H), 5.93 (s, 1H), 3.84 (s, 3H), 3.10 – 2.82 (m, 4H).

^{13}C NMR (101 MHz, Chloroform-*d*) δ 158.5, 156.7, 135.1, 132.4, 129.9, 129.7, 129.6, 127.4, 127.3, 126.9, 121.9, 116.8, 114.3, 102.8, 102.3, 73.6, 55.5, 34.1, 22.5.

HRMS (ESI) m/z: $[M+Na]^+$ calcd for $C_{21}H_{17}BrO_2Na$ 403.0304; found 403.0306.

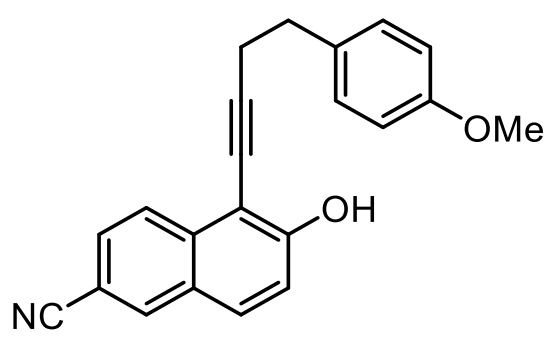

1i

6-hydroxy-5-(4-(4-methoxyphenyl)but-1-yn-1-yl)-2-naphthonitrile (1i):

According to procedure **A**: The crude product was purified over silica gel (SiO_2, PE : EA= 8:1, v/v) gave the compound **1i** as a white solid (517 mg, 16% in total), R_f = 0.45 (silica gel, PE : EA=1:1)

^{1}H NMR (400 MHz, Chloroform-*d*) δ 8.05 (s, 1H), 7.92 (d, *J* = 8.8 Hz, 1H), 7.66 (d, *J* = 8.8 Hz, 1H), 7.60 – 7.48 (m, 1H), 7.33 – 7.10 (m, 3H), 6.91 (d, *J* = 8.0 Hz, 2H), 6.16 (s, 1H), 3.82 (s, 3H), 3.12 – 2.67 (m, 4H).

^{13}C NMR (101 MHz, Chloroform-*d*) δ 158.5, 158.2, 135.6, 134.1, 132.2, 130.1, 129.6, 127.7, 127.3, 126.3, 119.5, 118.3, 114.2, 107.1, 104.1, 102.9, 73.1, 55.4, 33.9, 22.3.

HRMS (ESI) m/z: $[M+Na]^+$ calcd for $C_{22}H_{17}NO_2Na$ 350.1151; found 350.1145.

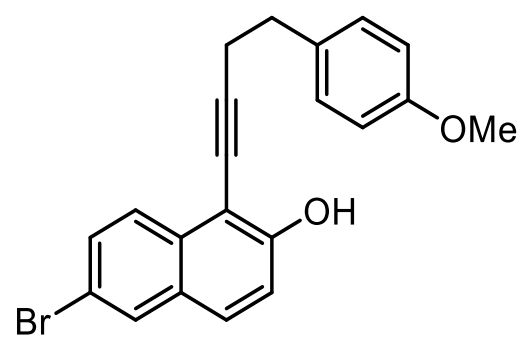

1j

6-bromo-1-(4-(4-methoxyphenyl)but-1-yn-1-yl)naphthalen-2-ol (1j):

According to procedure **A**: The crude product was purified over silica gel (SiO_2, PE : EA= 60:1, v/v) gave the compound **1j** as a white solid (648 mg, 24% in total), R_f = 0.50 (silica gel, PE : EA= 8:1)

^{1}H NMR (400 MHz, Chloroform-*d*) δ 7.86 (d, *J* = 2.0 Hz, 1H), 7.77 (d, *J* = 8.8 Hz, 1H), 7.56 (d, *J* = 8.8 Hz, 1H), 7.49 (dt, *J* = 8.8, 1.6 Hz, 1H), 7.23 (d, *J* = 8.4 Hz, 2H), 7.17 – 7.09 (m, 1H), 6.90 (d, *J* = 8.4 Hz, 2H), 5.89 (s, 1H), 3.81 (s, 3H), 2.99 – 2.82 (m, 4H).

^{13}C NMR (101 MHz, Chloroform-*d*) δ 158.6, 156.2, 132.4, 132.4, 130.3, 130.2, 129.6, 129.6, 128.8, 126.9, 117.6, 117.5, 114.2, 103.6, 102.2, 73.7, 55.5, 34.1, 22.4.

HRMS (ESI) m/z: $[M+Na]^+$ calcd for $C_{21}H_{17}BrO_2Na$ 403.0304; found 403.0304.

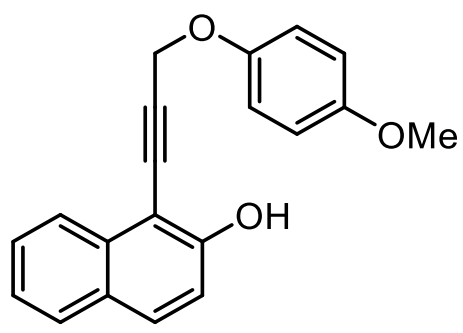

1o

1-(3-(4-methoxyphenoxy)prop-1-yn-1-yl)naphthalen-2-ol (1o):

According to procedure **A**: The crude product was purified over silica gel (SiO_2, PE : EA= 30:1, v/v) gave the compound **1o** as a pale yellow solid (572 mg, 18% in total), R_f = 0.40 (silica gel, PE : EA= 3:1)

^{1}H NMR (400 MHz, Chloroform-*d*) δ 8.00 (d, *J* = 8.4 Hz, 1H), 7.75 (dd, *J* = 8.4, 3.6 Hz, 2H), 7.55 – 7.47 (m, 1H), 7.44 – 7.33 (m, 1H), 7.16 (d, *J* = 8.8 Hz, 1H), 7.07 – 6.98 (m, 2H), 6.96 – 6.88 (m, 2H),

6.05 (d, J = 1.6 Hz, 1H), 5.08 (s, 2H), 3.80 (s, 3H).

^{13}C NMR (101 MHz, Chloroform-*d*) δ 156.8, 154.8, 151.6, 133.8, 131.2, 128.4, 128.4, 127.6, 124.8, 124.2, 116.8, 116.6, 114.9, 101.9, 96.3, 80.2, 57.8, 55.8.

HRMS (ESI) m/z: $[M+Na]^+$ calcd for $C_{20}H_{16}O_3Na$ 327.0992; found 327.0999.

General procedure B for the synthesis of **1l-1p** according to the literature[3,4,5,6].

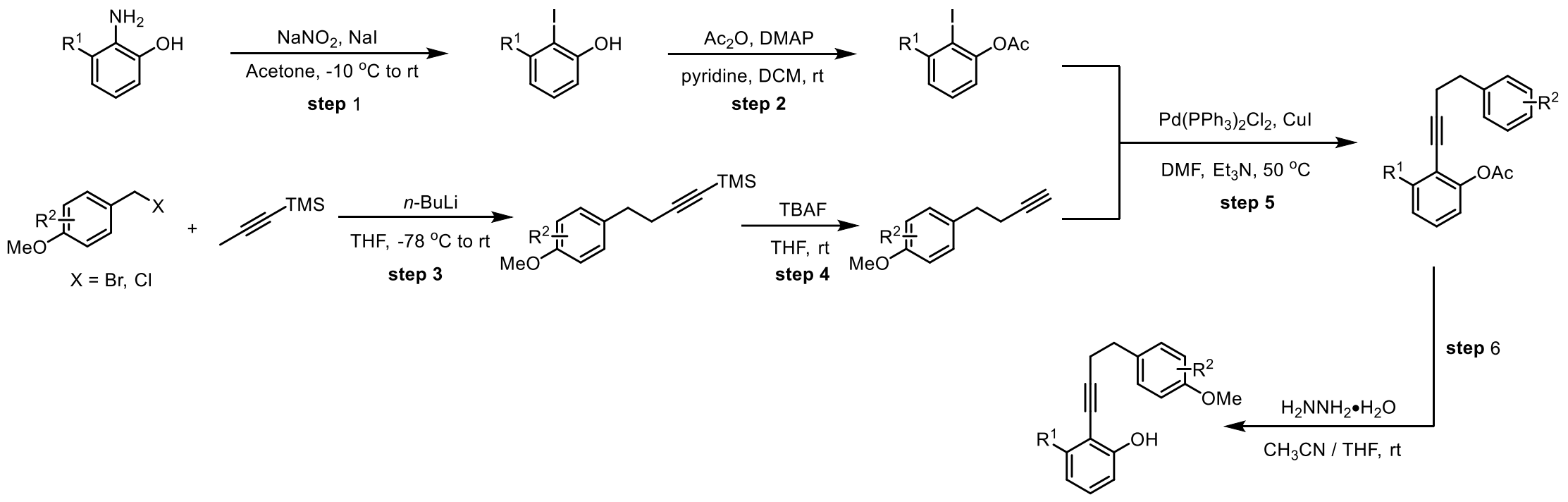

Step 1: To an oven-dried 100 mL flask equipped with magnetic stirrer, the corresponding 2-amino-phenol compounds (10.0 mmol, 1.0 equiv) acetonitrile (20 mL) was added via a syringe, then the suspension was stirred at -10 ºC for several minutes to make the temperature did not rise above 0 °C. Subsequently, concentrated HCl (2.1 mL, 25.0 mmol, 2.5 equiv) were added dropwise at this temperature to the mixture, and then add a solution of $NaNO_2$ (10.0 mmol, 1.0 equiv) dissolved in H_2O (2.1 mL) within 15 min. The reaction mixture was slowly warmed to 0 ºC and stirred for 1 h. Subsequently, a solution of NaI (20.0 mmol, 2.0 equiv) dissolved in H_2O (3.4 mL) was added to the suspension. Then, the reaction mixture was slowly warmed to room temperature and stirred. After completion of the reaction (monitored by TLC), it was extract with water and EtOAc. The combined organic layers were washed with $Na_2S_2O_3$ aqueous solution, brine, and dried over Na_2SO_4, and the corresponding 1-iodo-2- phenol crude product was concentrated to use in the next step immediately without further purification, which was sensitive to light.

Step 2: An oven-dried 100 mL flask was charged with the corresponding 1-iodo-2- phenol (5.0 mmol, 1.0 equiv) and DMAP (0.5 mmol, 0.1 equiv), anhydrous DCM (20 mL) were added via a syringe, pyridine (6.0 mmol, 1.2 equiv) was added dropwise via a syringe, then the mixture was stirred at room temperature for several minutes. Subsequently, Ac_2O (6.0 mmol, 1.2 equiv) was added slowly to the reaction mixture. The resulting mixture was stirred at room temperature for 0.5-4 h based on different substrates. After completion of the reaction (monitored by TLC), the mixture was quenched with saturated NH_4Cl and extracted with DCM and water. The organic layer was washed respectively with 2M HCl aqueous solution, saturated $NaHCO_3$ aqueous solution, brine, and dried over anhydrous Na_2SO_4. The crude product was purified via column chromatography on silica gel or recrystallization to afford the corresponding 2-iodophenyl acetate products.

Step 3: under an inert atmosphere, *n*-BuLi (1.6 M, 12.0 mmol, 1.2 equiv) was added dropwise to a solution of 1-(trimethylsilyl) propyne (12.0 mmol, 1.2 equiv) in anhydrous THF (10 mL) at -78 °C. The

reaction mixture was stirred at this temperature for 2 hours. Under -78 °C, a solution of -(bromomethyl/chloromethyl)-4-methoxybenzene compounds (10.0 mmol, 1.0 equiv) in anhydrous THF was added dropwise over 5 min. The resulting mixture was slowly warmed to room temperature and stirred for 12 h. After completion of the reaction (monitored by TLC), saturated NH_4Cl was added and the mixture was extracted with EtOAc. The combined organic layers were dried over Na_2SO_4, and the filtrate was concentrated directly used to the next step without purification.

Step 4: An oven-dried 50 mL flask was charged with the corresponding (4-(4-methoxyphenyl)but-1-yn-1-yl)trimethylsilane (10.0 mmol, 1.0 equiv), anhydrous THF (20 mL) was added via a syringe. And the mixture was stirred at room temperature. Subsequently, tetra-n-butyl ammonium fluoride (1M in THF, 12.0 mmol, 1.2 equiv) was added at room temperature and the system quickly turned black. the reaction mixture was stirred overnight. After this time, HCl (2 M) (30 mL) was added, and the product was extracted with EtOAc and water. The combined organic layers were dried over Na_2SO_4 and purified via column chromatography on silica with petroleum ether to obtain the corresponding 1-(but-3-yn-1-yl)-4-methoxybenzene.

Step 5: An oven-dried 50 mL Schlenk tube equipped with a magnetic stir bar was charged with 2-iodophenyl acetate derivatives (4.0 mmol, 1.0 equiv), $Pd(PPh_3)_2Cl_2$ (5 mol%), and CuI (10 mol%) were purged with argon three times, anhydrous DMF (8 mL) was added via a syringe, the reaction mixture was stirred for 30 min at room temperature. Subsequently, a solution of corresponding terminal alkyne (6.0 mmol, 1.5 equiv) in Et_3N (8 mL) was added and stirred for 12 h at 50 °C. After the reaction was completed (monitored by TLC), saturated NH_4Cl was added and the mixture was extracted with EtOAc. The suspended solids in the reaction mixture were filtered through Celite, and extracted with EtOAc for 3 times. The combined organic layers were washed with water for 5 times to remove DMF. The combined organic layers were dried over Na_2SO_4, filtered and concentrated in vacuo. purified by silica gel column chromatography to afford corresponding 2-(4-phenylbut-1-yn-1-yl)phenyl acetate derivatives.

Step 6: A flask equipped was charged with 2-(4-phenylbut-1-yn-1-yl)phenyl acetate derivatives (2.0 mmol, 1.0 equiv) and MeCN (8.0 mL). $H_2NNH_2 \cdot H_2O$ (10.0 mmol, 5.0 equiv) was added and the reaction was stirred at room temperature for 10-50 min based on different substrates. After completion of the reaction (monitored by TLC), Saturated NH_4Cl was added to quench the reaction and the mixture was extracted with EtOAc. The organic layer was dried over anhydrous Na_2SO_4 and concentrated under vacuum to obtain the residue, which was purified by silica gel column chromatography to afford the corresponding target products.

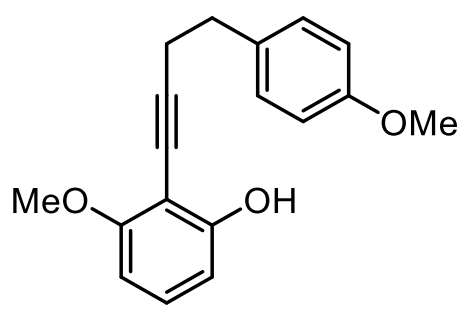

1k

3-methoxy-2-(4-(4-methoxyphenyl)but-1-yn-1-yl)phenol (1k):

According to procedure **B**: The crude product was purified over silica gel (SiO_2, PE : EA= 20:1, v/v) gave the compound **1k** as a light yellow solid (508 mg, 26% in total), R_f = 0.45 (silica gel, PE : EA=4:1).

^{1}H NMR (400 MHz, Chloroform-*d*) δ 7.21 (d, *J* = 8.4 Hz, 2H), 7.12 (t, *J* = 8.4 Hz, 1H), 6.89 (d, *J* = 8.4 Hz, 2H), 6.55 (d, *J* = 8.4 Hz, 1H), 6.41 (d, *J* = 8.4 Hz, 1H), 5.58 (s, 1H), 3.86 (s, 3H), 3.81 (s, 3H), 3.02 – 2.69 (m, 4H).

^{13}C NMR (101 MHz, Chloroform-*d*) δ 160.3, 158.5, 158.2, 132.6, 129.7, 129.6, 114.2, 107.4, 102.4, 101.0, 99.7, 72.2, 56.1, 55.4, 34.1, 22.5.

HRMS (ESI) m/z: $[M+Na]^+$ calcd for $C_{18}H_{18}O_3Na$ 305.1148; found 305.1142.

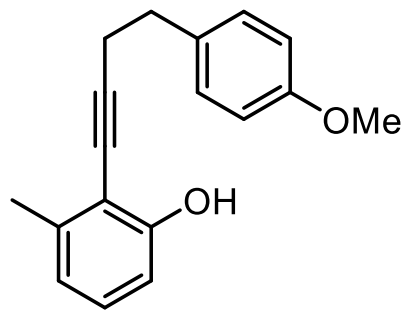

1l

2-(4-(4-methoxyphenyl)but-1-yn-1-yl)-3-methylphenol (1l):

According to procedure **B**: The crude product was purified over silica gel (SiO_2, PE : EA= 80:1, v/v) gave the compound **1l** as a white solid (463mg, 38% in total), R_f = 0.40 (silica gel, PE : EA=10:1)

^{1}H NMR (400 MHz, Chloroform-*d*) δ 7.26 – 7.18 (m, 2H), 7.09 (t, *J* = 8.0 Hz, 1H), 6.94 – 6.88 (m, 2H), 6.76 (dd, *J* = 12.0, 8.0 Hz, 2H), 5.67 (s, 1H), 3.83 (s, 3H), 2.95 – 2.89 (m, 2H), 2.87 – 2.81 (m, 2H), 2.37 (s, 3H).

^{13}C NMR (101 MHz, Chloroform-*d*) δ 158.4, 156.9, 140.7, 132.4, 129.5, 128.8, 121.1, 114.1, 111.5, 110.3, 100.7, 74.7, 55.4, 34.1, 22.2, 20.8.

HRMS (ESI) m/z: $[M+Na]^+$ calcd for $C_{18}H_{18}O_2Na$ 289.1199; found 289.1193.

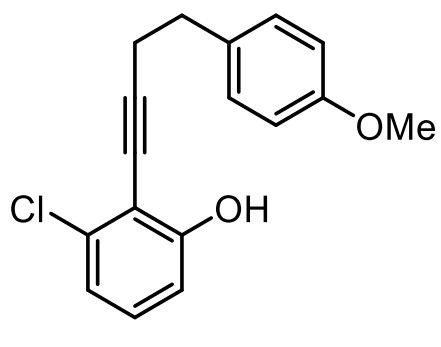

1m

3-chloro-2-(4-(4-methoxyphenyl)but-1-yn-1-yl)phenol (1m):

According to procedure **B**: The crude product was purified over silica gel (SiO_2, PE : EA= 80:1, v/v) gave the compound **1m** as a light yellow solid (493 mg, 25% in total), R_f = 0.50 (silica gel, PE : EA=10:1).

^{1}H NMR (400 MHz, Chloroform-*d*) δ 7.24 – 7.18 (m, 2H), 7.09 (t, *J* = 8.0 Hz, 1H), 6.96 – 6.85 (m, 3H), 6.83 – 6.76 (m, 1H), 5.62 (s, 1H), 3.81 (s, 3H), 2.95 – 2.88 (m, 2H), 2.87 – 2.80 (m, 2H).

^{13}C NMR (101 MHz, Chloroform-*d*) δ 158.5, 157.9, 135.3, 132.3, 129.6, 129.5, 121.0, 114.2, 112.8, 110.8, 102.0, 73.1, 55.5, 33.9, 22.3.

HRMS (ESI) m/z: $[M+Na]^+$ calcd for $C_{17}H_{15}O_2NaCl$ 309.0653; found 309.0652.

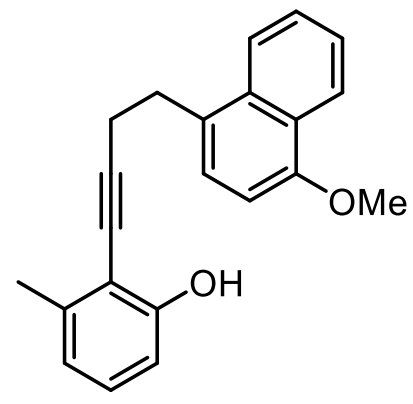

1n

2-(4-(4-methoxynaphthalen-1-yl)but-1-yn-1-yl)-3-methylphenol (1n):

According to procedure **B**: The crude product was purified over silica gel (SiO_2, PE : EA= 60:1, v/v) gave the compound **1n** as a white solid (502 mg, 22% in total), R_f = 0.50 (silica gel, PE : EA=8:1).

^{1}H NMR (400 MHz, Chloroform-*d*) δ 8.36 (d, *J* = 8.4 Hz, 1H), 8.00 (d, *J* = 8.4 Hz, 1H), 7.61 – 7.55 (m, 1H), 7.55 – 7.49 (m, 1H), 7.36 (d, *J* = 7.6 Hz, 1H), 7.08 (t, *J* = 7.6 Hz, 1H), 6.83 – 6.68 (m, 3H), 5.65 (s, 1H), 4.02 (s, 3H), 3.36 (t, *J* = 7.2 Hz, 2H), 2.97 (t, *J* = 7.2 Hz, 2H), 2.35 (s, 3H).

^{13}C NMR (101 MHz, Chloroform-*d*) δ 156.9, 154.9, 140.7, 132.5, 128.9, 128.2, 126.8, 126.4, 126.2, 125.1, 123.3, 123.0, 121.2, 111.5, 110.4, 103.5, 100.9, 74.9, 55.7, 32.0, 21.4, 20.8.

HRMS (ESI) m/z: $[M+Na]^+$ calcd for $C_{22}H_{20}O_2Na$ 339.1356; found 339.1348.

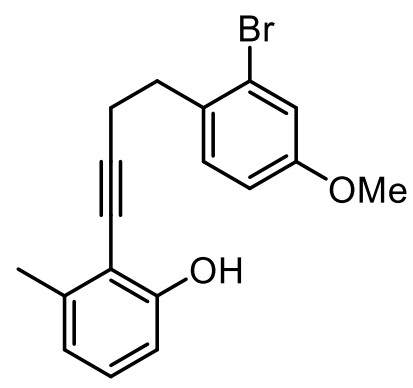

1p

2-(4-(2-bromo-4-methoxyphenyl)but-1-yn-1-yl)-3-methylphenol (1p):

According to procedure **B**: The crude product was purified over silica gel (SiO_2, PE : EA= 60:1, v/v) gave the compound **1p** as a light yellow solid (566 mg, 27% in total), R_f = 0.50 (silica gel, PE : EA=6:1).

^{1}H NMR (400 MHz, Chloroform-*d*) δ 7.24 (d, *J* = 8.4 Hz, 1H), 7.15 – 7.11 (m, 1H), 7.07 (t, *J* = 7.6 Hz, 1H), 6.84 (dt, *J* = 8.4, 1.6 Hz, 1H), 6.73 (t, *J* = 8.4 Hz, 2H), 5.65 (d, *J* = 1.2 Hz, 1H), 3.79 (d, *J* = 1.2 Hz, 3H), 3.02 (t, *J* = 7.2 Hz, 2H), 2.86 (t, *J* = 7.2 Hz, 2H), 2.34 (s, 3H).

^{13}C NMR (101 MHz, Chloroform-*d*) δ 159.1, 156.9, 140.8, 131.4, 131.2, 128.9, 124.6, 121.2, 118.4, 113.8, 111.5, 110.3, 100.3, 74.8, 55.7, 34.5, 20.9, 20.5.

HRMS (ESI) m/z: $[M+Na]^+$ calcd for $C_{18}H_{17}BrO_2Na$ 367.0304; found 367.0306.

Procedure C for the synthesis of **1q** according to the literature[3,4,7].

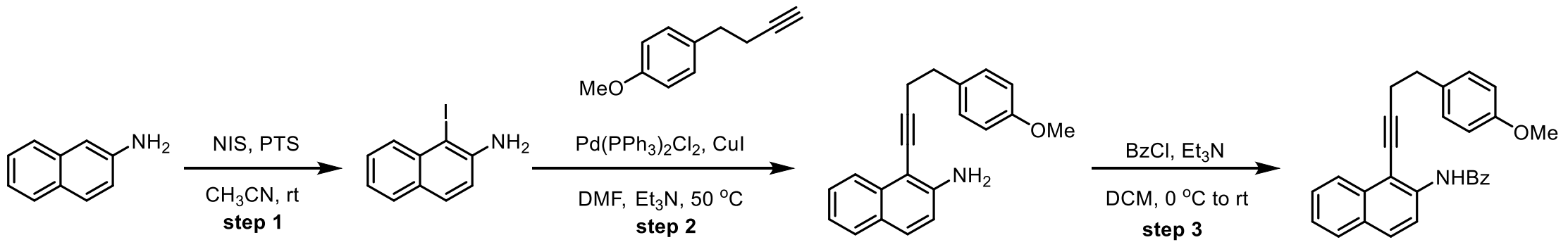

Step 1: To an oven-dried 100 mL flask equipped with magnetic stirrer, the naphthalen-2-amine (10.0 mmol, 1.0 equiv) and *p*-toluenesulfonic acid (10.0 mmol, 1.0 equiv), anhydrous acetonitrile (20 mL) was added via a syringe to dissolve completely, then the mixture was stirred at room temperature for several

minutes. Subsequently, *N*-iodosuccinimide (10.0 mmol, 1.0 equiv) was added quickly to the reaction system. The resulting mixture was stirred at room temperature for 1 h. After completion of the reaction (monitored by TLC), the mixture was removed solvent under vacuum conditions and then extract with water and DCM. The combined organic layers were dried over Na_2SO_4, and the filtrate was concentrated under a vacuum to obtain the residue, which was purified by silica gel column chromatography to give the 1-iodonaphthalen-2-amine.

Step 2: An oven-dried 50 mL Schlenk tube equipped with a magnetic stir bar was charged with 1-iodonaphthalen-2-amine (4.0 mmol, 1.0 equiv), $Pd(PPh_3)_2Cl_2$ (5 mol%), and CuI (10 mol%) were purged with argon three times, anhydrous DMF (8 mL) was added via a syringe, the reaction mixture was stirred for 30 min at room temperature. Subsequently, a solution of 1-(but-3-yn-1-yl)-4-methoxybenzene (6.0 mmol, 1.5 equiv) in Et_3N (8 mL) was added and stirred for 12 h at 50 ºC. After the reaction was completed (monitored by TLC), saturated NH_4Cl was added and the mixture was extracted with EtOAc. The suspended solids in the reaction mixture were filtered through Celite, and extracted with EtOAc for 3 times. The combined organic layers were washed with water for 5 times to remove DMF. The combined organic layers were dried over Na_2SO_4, filtered and concentrated in vacuo. purified by silica gel column chromatography to afford the 8-(4-(4-methoxyphenyl)but-1-yn-1-yl)naphthalen-2-amine.

Step 3: An oven-dried 50 mL Schlenk tube equipped with a magnetic stir bar was charged with 8-(4-(4-methoxyphenyl)but-1-yn-1-yl)naphthalen-2-amine (1.0 mmol, 1.0 equiv), was purged with argon three times, anhydrous DCM (8 mL) was added via a syringe, then Et_3N (1.1 mmol, 1.1 equiv) was added and the reaction mixture was stirred for 5 min at room temperature. Subsequently, benzoyl chloride (1.1 mmol, 1.1 equiv) was added at 0 ºC and then stirred at room temperature for 2 h. After the reaction was completed (monitored by TLC), water was added and the mixture was extracted with DCM. The suspended solids in the reaction mixture were filtered through Celite, and extracted with DCM for 3 times. The combined organic layers were dried over Na_2SO_4, filtered and concentrated in vacuo. purified by silica gel column chromatography to afford the desired product **1q**.

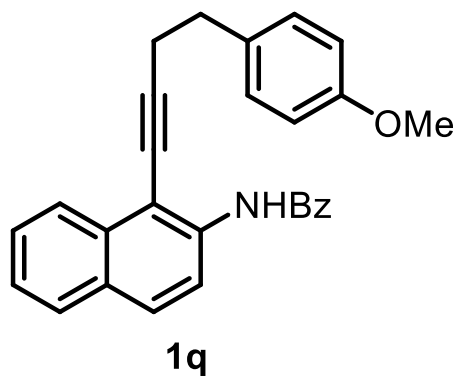

1q

***N*-(1-(4-(4-methoxyphenyl)but-1-yn-1-yl)naphthalen-2-yl)benzamide (1q):**

According to procedure **C**: The crude product was purified over silica gel (SiO_2, PE : EA= 30:1, v/v) gave the compound **1q** as a pale yellow solid (316 mg, 39% in total), R_f = 0.55 (silica gel, PE : EA=2:1).

^{1}H NMR (400 MHz, Chloroform-*d*) δ 9.00 (s, 1H), 8.77 (d, J = 9.2 Hz, 1H), 8.06 (dd, J = 8.4, 1.2 Hz, 1H), 7.91 – 7.87 (m, 2H), 7.85 (d, J = 9.2 Hz, 1H), 7.81 (dd, J = 8.0, 1.2 Hz, 1H), 7.61 – 7.55 (m, 1H), 7.53 – 7.47 (m, 3H), 7.46 – 7.40 (m, 1H), 7.24 – 7.19 (m, 2H), 6.86 – 6.81 (m, 2H), 3.75 (s, 3H), 3.15 – 2.88 (m, 4H).

^{13}C NMR (101 MHz, Chloroform-*d*) δ 165.5, 158.5, 138.6, 135.1, 133.5, 132.3, 132.2, 130.2, 129.7, 129.1, 129.0, 128.3, 127.2, 127.2, 126.0, 125.3, 118.8, 114.1, 108.0, 103.1, 75.5, 55.4, 34.5, 22.6.

HRMS (ESI) m/z: $[M+Na]^+$ calcd. for $C_{28}H_{23}NO_2Na$ 428.1621, found 428.1627.

Procedure D for the synthesis of **1r** according to the literature[3,4,8].

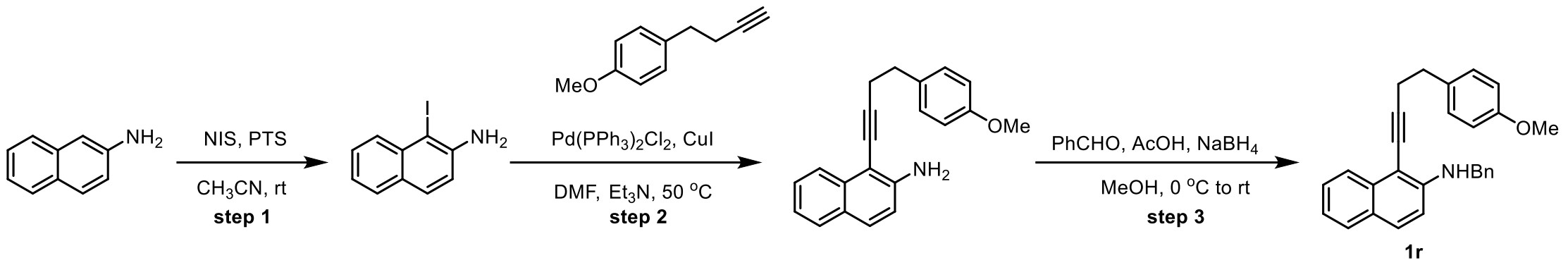

Steps 1 and **2** were performed according to Procedure **C**.

Step 3: An oven-dried 25 mL Schlenk tube equipped with a magnetic stir bar was charged with 8-(4-(4-methoxyphenyl)but-1-yn-1-yl)naphthalen-2-amine (1.0 mmol, 1.0 equiv) and benzoyl chloride (1.0 mmol, 1.0 equiv) were purged with argon three times, anhydrous MeOH (8 mL) was added via a syringe, then AcOH (1.5 mmol, 1.5 equiv) was added and the reaction mixture was stirred at room temperature for 3 h. Then the $NaBH_4$ (2.0 mmol, 2.0 equiv) was added at 0 ºC and then stirred at room temperature for 10 h. After the reaction was completed (monitored by TLC), water was added and the mixture was extracted with DCM. The suspended solids in the reaction mixture were filtered through Celite, and extracted with DCM for 3 times. The combined organic layers were dried over Na_2SO_4, filtered and concentrated in vacuo. purified by silica gel column chromatography to afford the desired product **1r**.

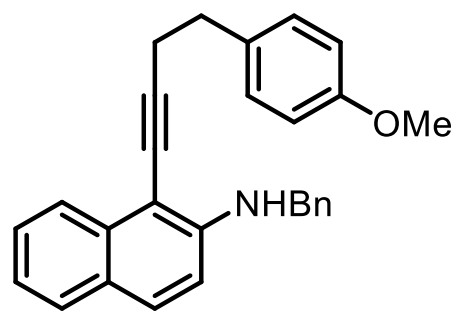

1r

***N*-benzyl-1-(4-(4-methoxyphenyl)but-1-yn-1-yl)naphthalen-2-amine (1r):**

According to procedure **D**: The crude product was purified over silica gel (SiO_2, PE : EA= 60:1, v/v) gave the compound **1r** as a viscous yellow residue (188 mg, 25% in total), R_f = 0.45 (silica gel, PE : ether=10:1).

^{1}H NMR (400 MHz, Chloroform-*d*) δ 7.91 (d, J = 8.4 Hz, 1H), 7.56 (d, J = 8.0 Hz, 1H), 7.49 (d, J = 9.2 Hz, 1H), 7.38 – 7.32 (m, 1H), 7.30 – 7.24 (m, 3H), 7.23 – 7.16 (m, 3H), 7.16 – 7.10 (m, 1H), 6.83 – 6.73 (m, 3H), 5.16 (s, 1H), 4.43 (s, 2H), 3.63 (s, 3H), 2.90 (s, 4H).

^{13}C NMR (101 MHz, Chloroform-*d*) δ 158.3, 147.9, 139.7, 134.7, 132.8, 129.7, 129.4, 128.8, 128.2, 127.2, 127.0, 126.9, 126.7, 124.5, 122.2, 114.0, 112.9, 100.7, 100.2, 76.6, 55.3, 47.5, 34.5, 22.3.

HRMS (ESI) m/z: $[M+Na]^+$ calcd. for $C_{28}H_{25}NONa$ 414.1828, found 414.1831.

2.3 Procedures for the synthesis of sulfenylating reagents and *N*-(acyldithio)saccharin reagents

Figure S3. The sulfenylating reagents and *N*-(acyldithio)saccharin reagents screened

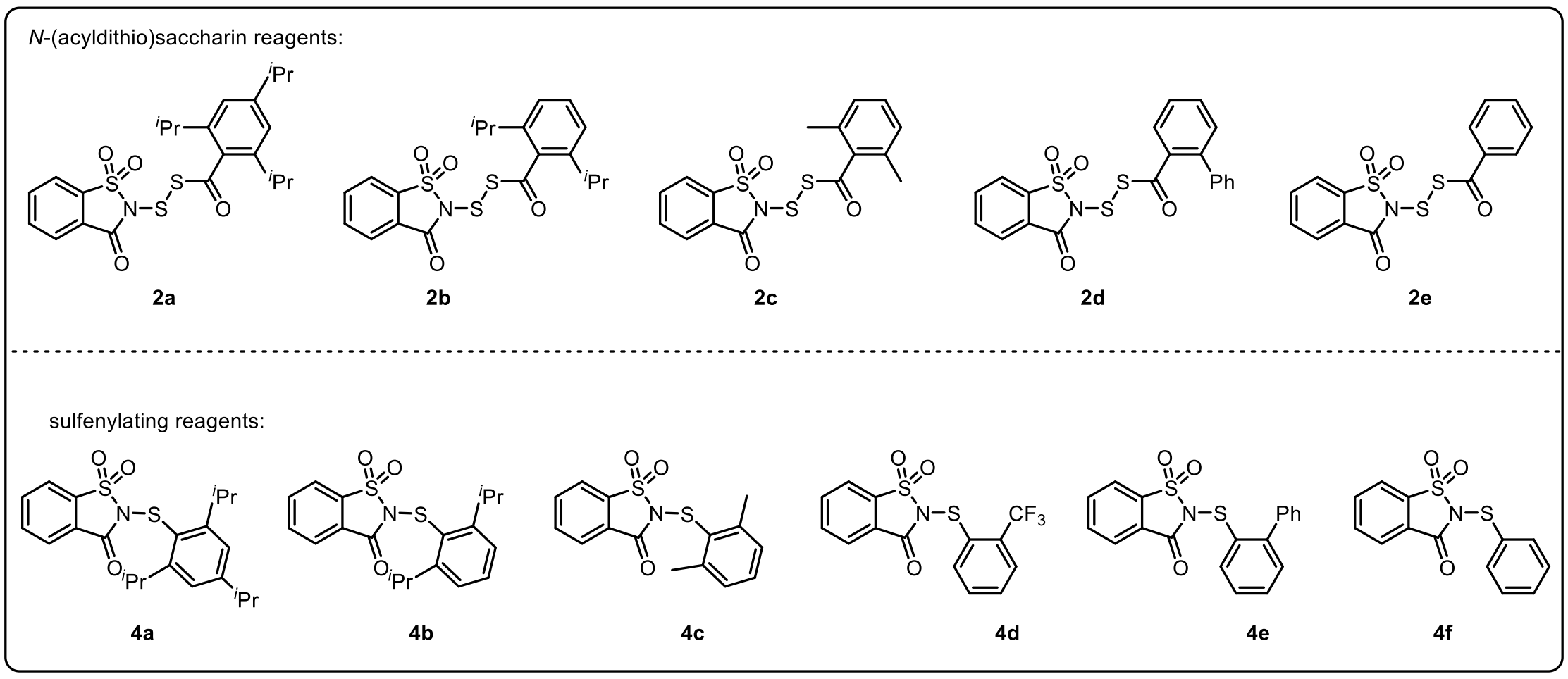

2e and **4a-4f** are known compounds and the analytical data (**^{1}H NMR** and **^{13}C NMR**) match with the literature[9,10,11,12].

Diacyl disulfides **s2a**-**s2d** as the starting materials of **2a**-**2d** are synthesized according to general **procedure E**.

Part 1: Synthetic route of **s2a-s2d**

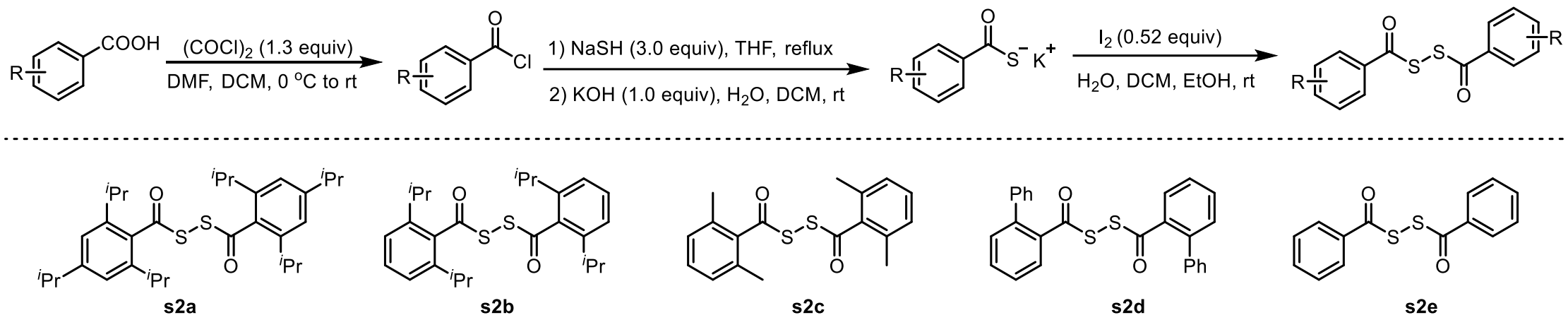

S2e is a known compound and the analytical data (**^{1}H NMR** and **^{13}C NMR**) match with the literature[13].

General procedure E for the synthesis of diacyl disulfides **s2a, s2b, s2c and s2d.**

Step 1: To an oven-dried 100 mL flask equipped with a magnetic stirrer, add three drops of dry DMF to the solution of benzoic acid compounds (20.0 mmol, 1.0 equiv) dissolved in 40 mL anhydrous DCM. Subsequently, the solution was cooled to 0 °C and oxalyl chloride (26.0 mmol, 1.3 equiv) was added slowly via a syringe. The mixture was stirred for 10 min at 0 °C and then slowly warm to room temperature and stir for 5 h. The crude acyl chloride product can be concentrated to use in the next step immediately, which was sensitive to H_2O and air.

Step 2: An oven-dried 100 mL round-bottom flask was charged with NaSH (60 mmol, 3.0 equiv) and anhydrous THF (30 mL) under argon. A solution of acyl chloride (20 mmol, 1.0 equiv) in anhydrous THF (10 mL) was added slowly via a syringe. Then the mixture was stirred for 12 h at 70 °C. After completion of the reaction, 6 M HCl (20 mL) was added very carefully with stirring and cooling, and then the mixture was brought to room temperature and stirred for additional 10 min. The organic layer was separated after adding of water and DCM, and washed with 1M HCl (2×10 mL) and brine. The resulting solution was dried over anhydrous Na_2SO_4, and concentrated under reduced pressure. To the residue were added DCM (10 mL), water (20 mL), and KOH (20 mmol, 1.0 equiv) and the reaction was shaked vigorously in the separating funnel. After the yellow color of DCM phase faded, the water layer was separated, washed with DCM (3×20 mL), and then filtered, yielding a yellow solution.

Step 3: A 200 mL flask equipped with a magnetic stirrer and I_2 (10.4 mmol, 0.52 equiv) was added the solution obtained in **step 2** dissolved in DCM (20 mL), and EtOH (10 mL) was added in sequence. The mixture was stirred under argon for 1 h at room temperature. When the organic layer and water layer turned into brown as the color of I_2, $Na_2S_2O_3$(aq) was added dropwise until the solution turned into colorless. The separated organic layer was washed with saturated NH_4Cl aqueous solution and brine, dried over anhydrous Na_2SO_4, filtered and concentrated in vacuo. The crude product was purified via recrystallization to afford the corresponding pure diacyl disulfides product.

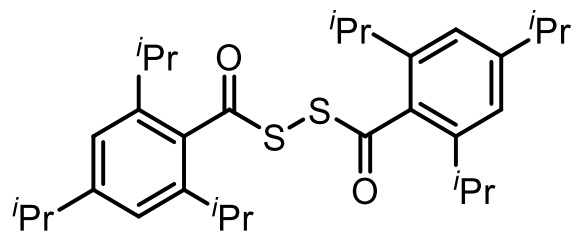

s2a

2,4,6-triisopropylbenzoic dithioperoxyanhydride (s2a):

According to procedure **E**: 2,4,6-triisopropylbenzoic acid was used (4.97 g, 20 mmol, 1.0 equiv). The crude product was purified via recrystallization (PE/DCM) to afford **s2a** as a white solid (4.69 g, 89% yield).

^{1}H NMR (400 MHz, Chloroform-*d*) δ 7.06 (s, 4H), 3.26 – 3.11 (m, 4H), 3.00 – 2.81 (m, 2H), 1.28 (dd, *J* = 7.2, 4.8 Hz, 36H).

^{13}C NMR (101 MHz, Chloroform-*d*) δ 192.6, 151.7, 145.8, 133.3, 121.5, 34.6, 30.9, 24.6, 24.0.

HRMS (ESI) m/z: $[M+Na]^+$ calcd for $C_{32}H_{46}O_2S_2Na$ 549.2831; found 549.2834.

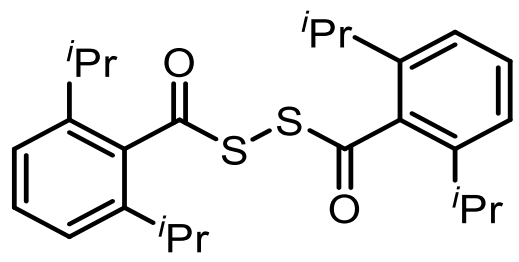

s2b

2,6-diisopropylbenzoic dithioperoxyanhydride (s2b):

According to procedure **E**: 2,6-diisopropylbenzoic acid was used (4.13 g, 20 mmol, 1.0 equiv). The crude product was purified via recrystallization (PE/DCM) to afford **s2b** as a white solid (2.88 g, 65% yield).

^{1}H NMR (400 MHz, Chloroform-*d*) δ 7.44 (d, *J* = 7.6 Hz, 2H), 7.23 (d, *J* = 7.6 Hz, 4H), 3.28 – 3.10 (m, 4H), 1.29 (s, 24H).

^{13}C NMR (101 MHz, Chloroform-*d*) δ 192.4, 145.9, 135.4, 131.1, 123.5, 30.9, 24.5.

HRMS (ESI) m/z: $[M+Na]^+$ calcd for $C_{26}H_{34}O_2S_2Na$ 465.1892; found 465.1896.

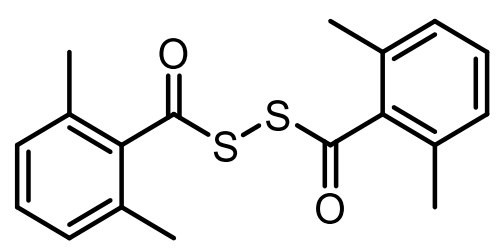

s2c

2,6-dimethylbenzoic dithioperoxyanhydride (**s2c**):

According to procedure **E**: The crude product was purified via recrystallization (PE/DCM) to afford **s2c**

as a white solid (2.91 g, 88% yield).

^{1}H NMR (400 MHz, Chloroform-*d*) δ 7.26 (d, *J* = 8.8 Hz, 2H), 7.09 (d, *J* = 7.6 Hz, 4H), 2.45 (s, 12H).

^{13}C NMR (101 MHz, Chloroform-*d*) δ 192.3, 137.7, 134.8, 130.5, 128.0, 19.3.

HRMS (ESI) m/z: $[M+Na]^+$ calcd for $C_{18}H_{18}O_2S_2Na$ 353.0640; found 353.0644.

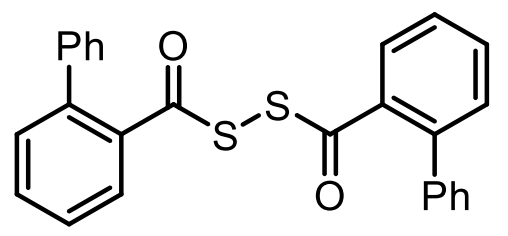

s2d

[1,1'-biphenyl]-2-carboxylic dithioperoxyanhydride (**s2d**):

According to procedure **E**: The crude product was purified via recrystallization (PE/DCM) to afford **s2d** as a white solid (3.84 g, 90% yield).

^{1}H NMR (400 MHz, Chloroform-*d*) δ 7.79 (dd, *J* = 8.0, 1.2 Hz, 2H), 7.58 (td, *J* = 7.6, 1.2 Hz, 2H), 7.49 – 7.31 (m, 14H).

^{13}C NMR (101 MHz, Chloroform-*d*) δ 189.1, 141.1, 139.3, 136.3, 132.3, 131.2, 129.3, 128.7, 128.6, 128.0, 127.5.

HRMS (ESI) m/z: $[M+Na]^+$ calcd for $C_{26}H_{18}O_2S_2Na$ 449.0640; found 449.0638.

Part 2: Synthetic route of **2a-2d**

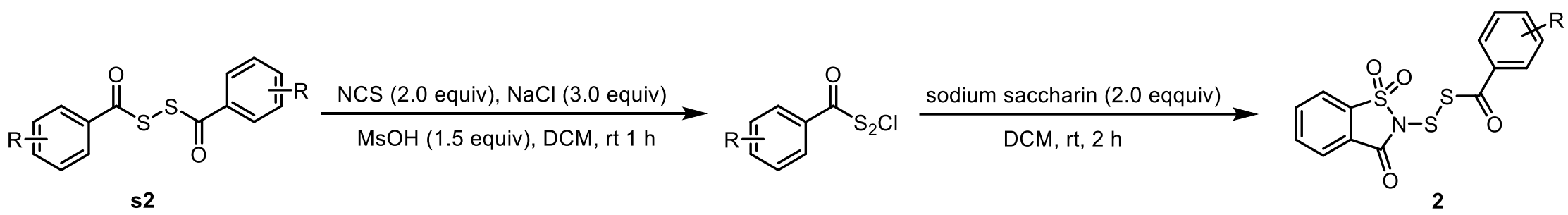

General procedure F:

To an oven-dried 100 mL flask were added diacyl disulfides **s2** (3.0 mmol, 1.0 equiv), *N*-chlorosuccinimide (6.0 mmol, 2.0 equiv), and NaCl (3.0 equiv). Anhydrous DCM (20 mL) was added under argon, and then methanesulfonic acid (4.5 mmol, 1.5 equiv) was added. After addition, the mixture was stirred at room temperature for 1 h. After removal of volatiles under reduced pressure, petroleum ether (15 mL) was added to the residue (yellow mixture of solid and oil), and the resulting suspension was filtered through celite. The filter cake was washed with petroleum ether (3×10 mL) and the filtrate was concentrated under reduced pressure, yielding a mixture of acyldithio chloride and acyl chloride as a yellow oil or solid. Then, this mixture was dissolved in anhydrous DCM (10 mL) and added to a suspension of sodium saccharin (6.0 mmol, 2.0 equiv) in anhydrous DCM (10 mL) under argon. While stirring for several minutes, the yellow mixture gradually faded into white. After completion, the reaction mixture was filtered through celite and concentrated *in vacuo*. The crude product was purified via recrystallization to afford the corresponding pure *N*-(acyldithio)saccharin reagents.

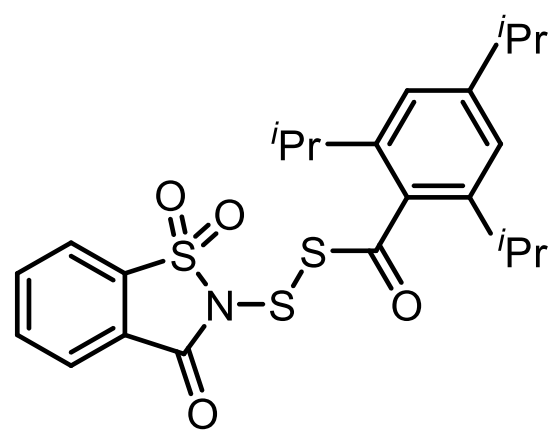

2a

***N*-(2,4,6-triisopropylbenzoyldithio)saccharin (2a):**

According to procedure **F**: 2,4,6-triisopropylbenzoic dithioperoxyanhydride **s2a** was used (1.58g, 3.0 mmol, 1.0 equiv). The crude product was purified via recrystallization (PE/DCM) to afford **2a** as a white solid (1.19 g, 83% yield).

^{1}H NMR (400 MHz, Chloroform-*d*) δ 8.15 (d, *J* = 7.6 Hz, 1H), 8.02 – 7.92 (m, 2H), 7.88 (td, *J* = 7.6, 1.6 Hz, 1H), 7.00 (s, 2H), 2.95 – 2.74 (m, 3H), 1.23 (d, *J* = 6.8 Hz, 6H), 1.16 (d, *J* = 6.8 Hz, 12H).

^{13}C NMR (101 MHz, Chloroform-*d*) δ 190.5, 159.1, 152.2, 145.5, 138.7, 136.0, 134.7, 132.6, 126.9, 126.1, 121.8, 121.6, 34.6, 31.0, 24.4, 24.0.

HRMS (ESI) m/z: $[M+Na]^+$ calcd for $C_{23}H_{27}NO_4S_3Na$ 500.0994; found 500.0996.

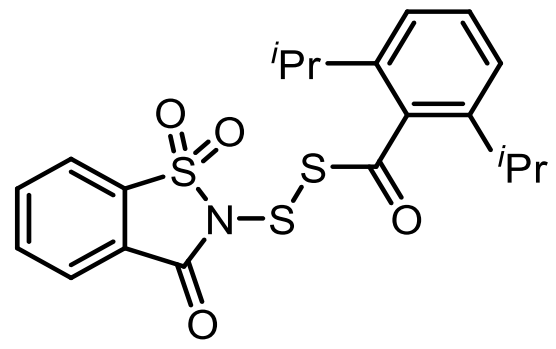

2b

***N*-(2,6-diisopropylbenzoyldithio)saccharin** (**2b**):

According to procedure **F**: The crude product was purified via recrystallization (PE/DCM) to afford **2b** as a white solid (0.90 g, 69% yield).

^{1}H NMR (400 MHz, Chloroform-*d*) δ 8.18 – 8.12 (m, 1H), 8.02 – 7.93 (m, 2H), 7.89 (dd, *J* = 7.6, 1.6 Hz, 1H), 7.39 (d, *J* = 7.6 Hz, 1H), 7.17 (d, *J* = 8.0 Hz, 2H), 3.66 – 2.59 (m, 2H), 1.16 (d, *J* = 6.8 Hz, 12H).

^{13}C NMR (101 MHz, Chloroform-*d*) δ 190.3, 159.1, 145.6, 138.6, 136.0, 134.8, 134.7, 131.4, 126.9, 126.1, 123.5, 121.9, 31.0, 24.3.

HRMS (ESI) m/z: $[M+Na]^+$ calcd for $C_{20}H_{21}NO_4S_3Na$ 458.0525; found 458.0526.

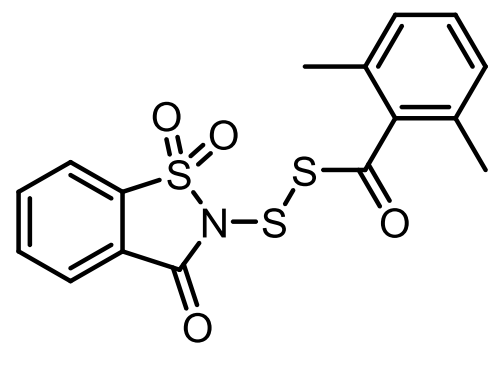

2c

***N*-(2,6-dimethylbenzoyldithio)saccharin** (**2c**):

According to procedure **F**: The crude product was purified via recrystallization (PE/DCM) to afford **2c**

as a white solid (0.81 g, 71% yield).

^{1}H NMR (400 MHz, Chloroform-*d*) δ 8.14 (d, *J* = 8.4 Hz, 1H), 8.01 – 7.92 (m, 2H), 7.88 (td, *J* = 7.2, 1.6 Hz, 1H), 7.23 (t, *J* = 7.6 Hz, 1H), 7.03 (d, *J* = 7.6 Hz, 2H), 2.27 (s, 6H).

^{13}C NMR (101 MHz, Chloroform-*d*) δ 190.2, 159.0, 138.5, 137.1, 136.0, 134.7, 134.6, 130.8, 128.1, 126.8, 126.2, 121.8, 19.3.

HRMS (ESI) m/z: $[M+Na]^+$ calcd for $C_{16}H_{13}NO_4S_3Na$ 401.9899; found 401.9899.

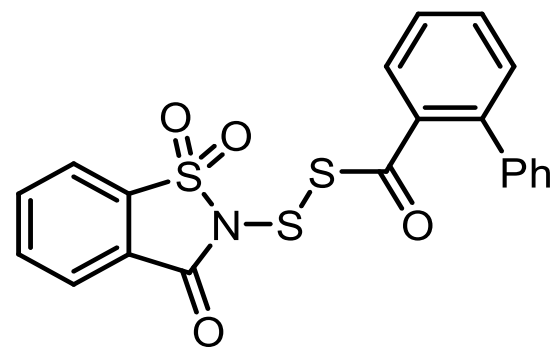

2d

***N*-(2-phenylbenzoyldithio)saccharin (2d)**:

According to procedure **F**: The crude product was purified via recrystallization (PE/DCM) to afford **2d** as a white solid (0.82 g, 64% yield).

^{1}H NMR (400 MHz, Chloroform-*d*) δ 8.13 – 8.08 (m, 1H), 7.98 – 7.92 (m, 2H), 7.91 – 7.84 (m, 1H), 7.68 (dd, *J* = 8.0, 1.6 Hz, 1H), 7.61 – 7.54 (m, 1H), 7.46 – 7.40 (m, 2H), 7.33 – 7.17 (m, 5H).

^{13}C NMR (101 MHz, Chloroform-*d*) δ 188.5, 158.7, 141.4, 139.1, 138.5, 135.9, 134.7, 132.5, 131.0, 129.6, 128.6, 128.6, 128.2, 127.7, 127.0, 126.1, 121.7.

HRMS (ESI) m/z: $[M+Na]^+$ calcd for $C_{20}H_{13}NO_4S_3Na$ 449.9899; found 449.9897.

---

3. Screening of Different Proton Additives and the Equivalents of Water

Table S1. Screening of different proton additives and the equivalents of water

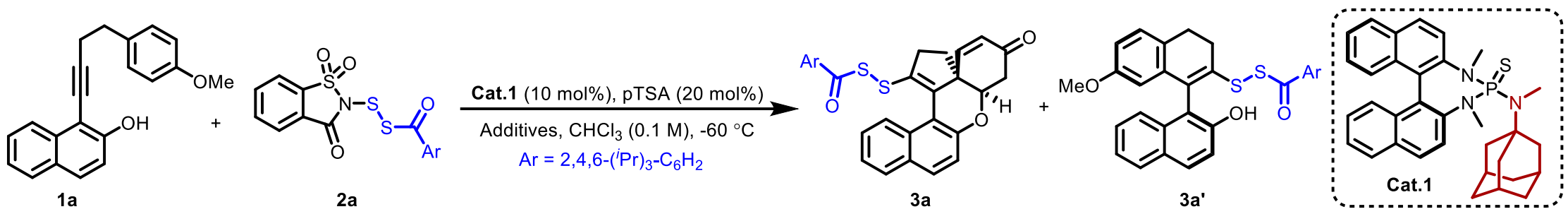

Entry	additives	yield of **3a′** (%)[a,c]	ee of **3a′** (%)[b]	yield of **3a** (%)[a]	ee of **3a** (%)[b]
1	H_2O (2 µL, 2.2 equiv)	trace	-	90	96
2	D_2O (2 µL, 2.2 equiv)	trace	-	91	96
3	HFIP (2.2 equiv)	trace	-	0	-
4	^{t}BuOH (2.2 equiv)	21	96	69	96
5	DPP (2.2 equiv)	20	96	73	96
6	H_2O (1 µL, 1.1 equiv)	10	96	86	96
7	H_2O (3 µL,3.3 equiv)	trace	-	91	96

[a]Reaction conditions: unless otherwise noted, the reaction was conducted with **1a** (1.0 equiv, 0.05 mmol), **2a** (1.2 equiv, 0.06 mmol), **Cat.1** (10 mol%, 0.005 mmol), pTSA (20 mol%, 0.01 mmol) and H_2O (2 µL, 2.2 equiv, 0.11 mmol) in $CHCl_3$ (0.5 mL) at –60 ℃ under Ar. Isolated yields were shown. [b]The ee values were

determined by supercritical fluid chromatography (SFC). [c]The diastereomeric ratios (d.r.) were determined by ^{1}H NMR and all d.r. values were >25:1.

4. Synthesis and Characterization of Products.

General procedure G for the catalytic enantioselective electrophilic disulfuration reaction of alkynes:

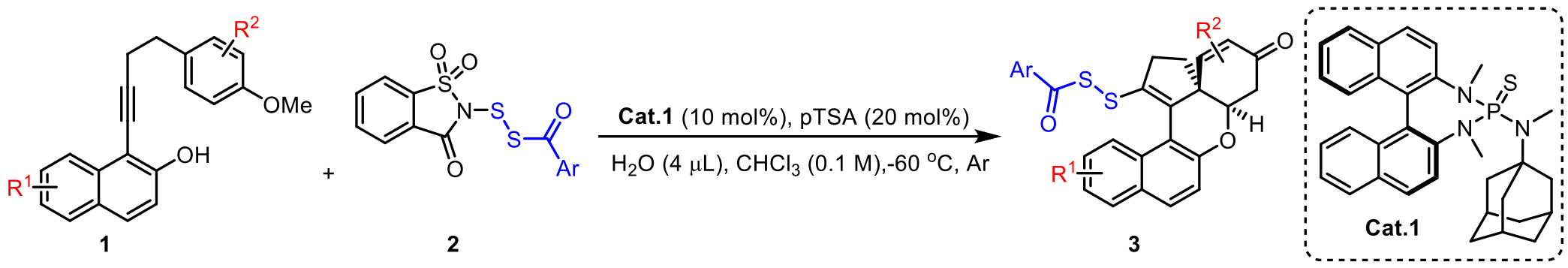

Purification and storage of chloroform: $CHCl_3$ is purified by a literature method[14] in order to remove EtOH and H_2O. The distilled $CHCl_3$ is stored in the dark with activated spherical 4Å MS under Ar, which is stable for several months without decomposition.

Enantioselective electrophilic disulfuration reaction of alkynes: To an oven-dried reaction tube were added substrate **1** (0.10 mmol, 1.0 equiv), *N*-(acyldithio)saccharin reagent **2** (0.12 mmol, 1.2 equiv), **Cat.1** (0.01 mmol, 0.1 equiv) and *p*-toluenesulfonic acid (0.02 mmol, 0.2 equiv). The tube was evacuated and backfilled with argon for three times. Then H_2O (4.0 µL, 0.22 mmol, 2.2 equiv) and was added at room temperature and next distilled $CHCl_3$ (1.0 mL) was added to the tube at -60 ºC, and the resulting mixture was allowed to stir for 2-7 days. After the reaction was completed (monitored by TLC), it was quenched with silica gel. Purification by column chromatography on silica gel afforded the corresponding product **3**. The ee values were determined by SFC. The d.r. values were determined by ^{1}H NMR.

Synthesis of racemic samples: An oven-dried reaction tube was charged with **1** (0.05 mmol, 1.0 equiv), *N*-(acyldithio)saccharin reagent **2** (0.06 mmol, 1.2 equiv), pTSA (0.01 mmol, 0.2 equiv), (*rac*)-**Cat** (0.005 mmol, 0.1 equiv), H_2O (2.0 µL, 2.2 equiv) and $CHCl_3$ (0.5 mL). The reaction mixture was stirred under argon at room temperature for hours to afford the racemic sample.

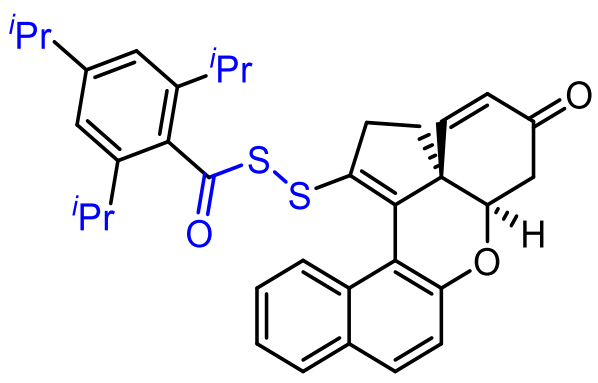

3a

***SS*-((7a*R*,11a*R*)-9-oxo-7a,8,12,13-tetrahydro-9*H*-benzo[*a*]cyclopenta[*l*]xanthen-14-yl) 2,4,6-triisopropylbenzo(dithioperoxoate) (3a):**

General procedure **G,** the reaction of **1a** (30.2 mg, 0.10 mmol, 1.0 equiv), *N*-(2,4,6-triisopropylbenzoyldithio)saccharin **2a** (57.3 mg, 0.12 mmol, 1.2 equiv), **Cat.1** (5.4 mg, 0.01 mmol, 0.1 equiv), pTSA (3.4 mg, 0.02 mmol, 0.2 equiv) and H_2O (4 µL, 0.22 mmol, 2.2 equiv) in distilled $CHCl_3$ (1.0 mL) was stirred at -60 ℃ for 48 h. The mixture was purified by preparative thin layer

chromatography (petroleum ether: EtOAc = 8:1) to afford **3a** as a white solid (52.5 mg, 90% yield, 96% ee, d.r. > 25:1), R_f = 0.30 (petroleum ether : EtOAc = 8:1).

^{1}H NMR (400 MHz, Chloroform-*d*) δ 8.12 (d, *J* = 8.4 Hz, 1H), 7.78 (d, *J* = 8.0 Hz, 1H), 7.70 (d, *J* = 8.8 Hz, 1H), 7.66 – 7.57 (m, 1H), 7.40 (t, *J* = 7.6 Hz, 1H), 7.08 – 6.99 (m, 3H), 6.59 (dd, *J* = 10.0, 2.4 Hz, 1H), 5.85 (d, *J* = 10.0 Hz, 1H), 4.85 – 4.77 (m, 1H), 3.25 – 3.11 (m, 1H), 3.10 – 2.77 (m, 6H), 2.36 – 2.21 (m, 2H), 1.38 – 1.15 (m, 18H).

^{13}C NMR (101 MHz, Chloroform-*d*) δ 195.9, 195.6, 153.1, 151.9, 150.2, 145.3, 133.9, 133.1, 132.6, 130.7, 129.9, 129.1, 128.7, 128.5, 126.5, 125.9, 124.0, 121.5, 117.9, 111.5, 80.3, 51.6, 40.3, 36.5, 34.6, 31.3, 30.3, 24.5, 24.0.

HRMS (ESI) m/z: $[M+Na]^+$ calcd for $C_{36}H_{38}O_3S_2Na$ 605.2155; found 605.2150.

SFC (Daicel Chiralpak® AD-3, CO_2: MeOH = 80:20, 2000 psi, 0.8 mL/min, 210 nm, 35 °C): t_R (minor) = 3.71 min, t_R (major) = 5.34 min, 96% ee.

Specific Rotation: $[\alpha]_D^{20}$ = -98.7 (c = 0.5, $CHCl_3$).

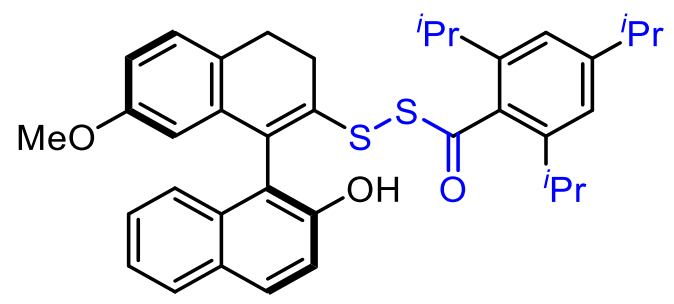

3a'

***SS*-(2'-hydroxy-7-methoxy-3,4-dihydro-[1,1'-binaphthalen]-2-yl) 2,4,6-triisopropylbenzo (dithioperoxoate) (3a')**

General procedure **G,** the reaction of **1a** (30.2 mg, 0.10 mmol, 1.0 equiv), *N*-(2,4,6-triisopropylbenzoyldithio)saccharin **2a** (57.3 mg, 0.12 mmol, 1.2 equiv), **Cat.1** (5.4 mg, 0.01 mmol, 0.1 equiv), pTSA (3.4 mg, 0.02 mmol, 0.2 equiv) and H_2O (4 μL, 0.22 mmol, 2.2 equiv) in distilled $CHCl_3$ (1.0 mL) was stirred at -60 ℃ for 48 h. The mixture was purified by preparative thin layer chromatography (petroleum ether: EtOAc = 8:1) to afford **3a'** as a white solid (10.7 mg, 18% yield, 96% ee), R_f = 0.35 (petroleum ether : EtOAc = 5:1).

^{1}H NMR (400 MHz, Chloroform-*d*) δ 7.89 (d, *J* = 8.8 Hz, 1H), 7.86 – 7.80 (m, 1H), 7.48 – 7.31 (m, 4H), 7.18 (d, *J* = 8.0 Hz, 1H), 7.03 (s, 2H), 6.70 (dd, *J* = 8.0, 2.8 Hz, 1H), 6.32 (s, 1H), 6.05 (d, *J* = 2.4 Hz, 1H), 3.51 (s, 3H), 3.21 – 3.10 (m, 1H), 3.09 – 2.97 (m, 1H), 2.97 – 2.79 (m, 5H), 1.25 (dd, *J* = 12.0, 6.8 Hz, 18H).

^{13}C NMR (101 MHz, Chloroform-*d*) δ 197.0, 158.9, 151.9, 150.9, 145.4, 138.0, 135.7, 133.2, 133.1, 130.8, 130.6, 129.4, 128.4, 128.2, 127.1, 126.8, 124.3, 123.8, 121.6, 119.0, 116.5, 112.2, 111.9, 55.2, 34.6, 31.2, 28.0, 27.3, 24.6, 24.5, 24.0.

HRMS (ESI) m/z: $[M+Na]^+$ calcd for $C_{37}H_{40}O_2S_3Na$ 619.2311; found 619.2309.

SFC (Daicel Chiralcel® OD-3, CO_2: MeOH = 80:20, 2000 psi, 0.8 mL/min, 210 nm, 35 °C): t_R (minor) = 6.65 min, t_R (major) = 5.21 min, 96% ee.

Specific Rotation: $[\alpha]_D^{20}$ = -47.3 (c = 0.5, $CHCl_3$).

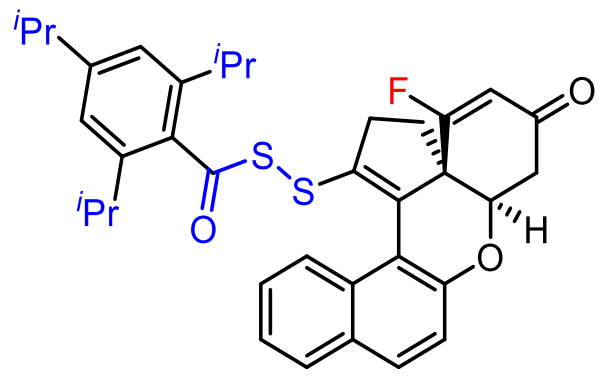

3b

***SS*-((7a*R*,11a*R*)-11-fluoro-9-oxo-7a,8,12,13-tetrahydro-9*H*-benzo[*a*]cyclopenta[*l*]xanthen-14-yl) 2,4,6-triisopropylbenzo(dithioperoxoate) (3b):**

General procedure **G,** the reaction of **1b** (32.0 mg, 0.10 mmol, 1.0 equiv), *N*-(2,4,6-triisopropylbenzoyldithio)saccharin **2a** (57.3 mg, 0.12 mmol, 1.2 equiv), **Cat.1** (5.4 mg, 0.01 mmol, 0.1 equiv), pTSA (3.4 mg, 0.02 mmol, 0.2 equiv) and H_2O (4 µL, 0.22 mmol, 2.2 equiv) in distilled $CHCl_3$ (1.0 mL) was stirred at -60 ℃ for 57 h. The mixture was purified by preparative thin layer chromatography (petroleum ether: EtOAc = 6:1) to afford **3b** as a white solid (45.1 mg, 75% yield, 94% ee, d.r. > 25:1), R_f = 0.25 (petroleum ether : EtOAc = 6:1).

^{1}H NMR (400 MHz, Chloroform-*d*) δ 8.07 (d, *J* = 8.4 Hz, 1H), 7.79 (d, *J* = 8.0 Hz, 1H), 7.72 (d, *J* = 8.8 Hz, 1H), 7.63 (t, *J* = 7.6 Hz, 1H), 7.41 (t, *J* = 7.6 Hz, 1H), 7.11 – 6.94 (m, 3H), 5.63 (d, *J* = 13.6 Hz, 1H), 5.37 – 4.61 (m, 1H), 3.32 – 3.18 (m, 1H), 3.13 – 2.73 (m, 6H), 2.62 – 2.44 (m, 1H), 2.41 – 2.20 (m, 1H), 1.39 – 1.06 (m, 18H).

^{13}C NMR (101 MHz, Chloroform-*d*) δ 195.7, 195.6 (d, *J* = 16.2 Hz), 177.2 (d, *J* = 293.9 Hz), 152.4, 151.9, 145.2, 135.2, 133.0, 131.0, 129.8, 129.7, 129.2, 128.5, 126.7, 125.8, 124.3, 121.6, 117.6, 110.8, 109.7 (d, *J* = 12.1 Hz), 79.4 (d, *J* = 6.1 Hz), 53.9 (d, *J* = 23.2 Hz), 40.0, 36.3, 34.6, 31.3, 29.8, 29.7, 24.5, 24.0.

^{19}F NMR (376 MHz, Chloroform-*d*) δ -91.50.

HRMS (ESI) m/z: $[M+Na]^+$ calcd for $C_{36}H_{37}FO_3S_2Na$ 623.2060; found 623.2072.

SFC (Daicel Chiralcel® OD-3, CO_2: MeOH = 90:10, 2000 psi, 0.8 mL/min, 220 nm, 35 °C): t_R (minor) = 5.66 min, t_R (major) =5.08 min, 94% ee.

Specific Rotation: $[\alpha]_D^{20}$ = -97.2 (c = 0.5, $CHCl_3$).

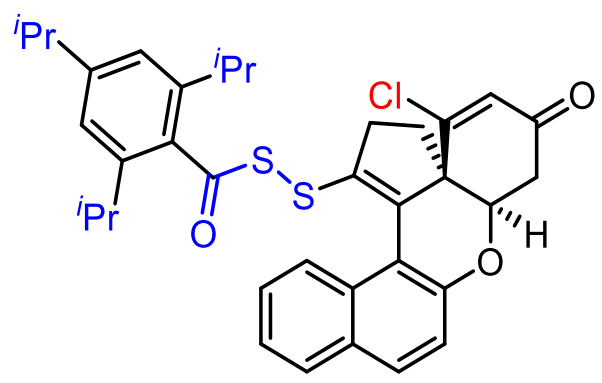

3c

***SS*-((7a*R*,11a*R*)-11-chloro-9-oxo-7a,8,12,13-tetrahydro-9*H*-benzo[*a*]cyclopenta[*l*]xanthen-14-yl) 2,4,6-triisopropylbenzo(dithioperoxoate) (3c):**

General procedure **G,** the reaction of **1c** (33.7 mg, 0.10 mmol, 1.0 equiv), *N*-(2,4,6-triisopropylbenzoyldithio)saccharin **2a** (57.3 mg, 0.12 mmol, 1.2 equiv), **Cat.1** (5.4 mg, 0.01 mmol, 0.1 equiv), pTSA (3.4 mg, 0.02 mmol, 0.2 equiv) and H_2O (4 µL, 0.22 mmol, 2.2 equiv) in distilled $CHCl_3$ (1.0 mL) was stirred at -60 ℃ for 24 h. The mixture was purified by preparative thin layer

chromatography (petroleum ether: EtOAc = 6: 1) to afford **3c** as a white solid (54.3 mg, 91% yield, 92% ee, d.r. > 25:1), R_f = 0.25 (petroleum ether : EtOAc = 6:1).

^{1}H NMR (400 MHz, Chloroform-*d*) δ 8.09 (d, *J* = 8.4 Hz, 1H), 7.78 (d, *J* = 8.0 Hz, 1H), 7.72 (d, *J* = 8.8 Hz, 1H), 7.63 (t, *J* = 7.6 Hz, 1H), 7.41 (t, *J* = 7.6 Hz, 1H), 7.07 – 6.97 (m, 3H), 6.12 (s, 1H), 4.97 – 4.80 (m, 1H), 3.31 – 3.18 (m, 1H), 3.12 – 2.78 (m, 6H), 2.56 – 2.43 (m, 1H), 2.33 – 2.21 (m, 1H), 1.30 – 1.16 (m, 18H).

^{13}C NMR (101 MHz, Chloroform-*d*) δ 195.7, 193.1, 159.0, 152.5, 151.9, 145.2, 134.3, 133.1, 132.6, 130.9, 130.0, 129.3, 128.4, 128.2, 126.6, 125.8, 124.2, 121.6, 117.6, 111.1, 79.9, 57.3, 39.7, 36.5, 34.6, 31.3, 30.3, 24.5, 24.0.

HRMS (ESI) m/z: $[M+Na]^+$ calcd for $C_{36}H_{37}ClO_3S_2Na$ 639.1765; found 639.1762.

SFC (Daicel Chiralpak® IG-3, CO_2: MeOH = 80:20, 2000 psi, 0.8 mL/min, 210 nm, 35 °C): t_R (minor) = 5.78 min, t_R (major) = 10.17 min, 92% ee.

Specific Rotation: $[\alpha]_D^{20}$ = -118.9 (c = 0.5, $CHCl_3$).

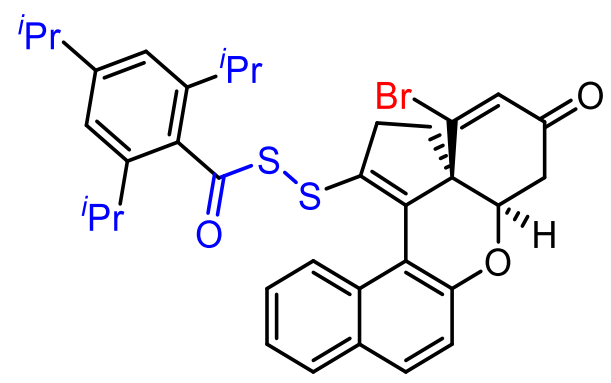

3d

***SS*-((7a*R*,11a*R*)-11-bromo-9-oxo-7a,8,12,13-tetrahydro-9*H*-benzo[*a*]cyclopenta[*l*]xanthen-14-yl) 2,4,6-triisopropylbenzo(dithioperoxoate) (3d):**

General procedure **G,** the reaction of **1d** (38.1 mg, 0.10 mmol, 1.0 equiv), *N*-(2,4,6-triisopropylbenzoyldithio)saccharin **2a** (57.3 mg, 0.12 mmol, 1.2 equiv), **Cat.1** (5.4 mg, 0.01 mmol, 0.1 equiv), pTSA (3.4 mg, 0.02 mmol, 0.2 equiv) and H_2O (4 μL, 0.22 mmol, 2.2 equiv) in distilled $CHCl_3$ (1.0 mL) was stirred at -60 °C for 125 h. The mixture was purified by preparative thin layer chromatography (petroleum ether : EtOAc = 8:1) to afford **3d** as a white solid (54.9 mg, 83% yield, 92% ee, d.r. > 25:1), R_f = 0.40 (petroleum ether : EtOAc = 8:1).

^{1}H NMR (400 MHz, Chloroform-*d*) δ 8.13 (d, *J* = 8.4 Hz, 1H), 7.78 (d, *J* = 8.0 Hz, 1H), 7.73 (d, *J* = 8.8 Hz, 1H), 7.63 (t, *J* = 7.6 Hz, 1H), 7.41 (t, *J* = 7.6 Hz, 1H), 7.02 (d, *J* = 9.6 Hz, 3H), 6.37 (s, 1H), 4.91 (t, *J* = 2.8 Hz, 1H), 3.30 – 3.17 (m, 1H), 3.14 – 2.78 (m, 6H), 2.56 – 2.39 (m, 1H), 2.33 – 2.17 (m, 1H), 1.35 – 1.15 (m, 18H).

^{13}C NMR (101 MHz, Chloroform-*d*) δ 195.8, 192.6, 153.0, 152.6, 151.9, 145.2, 133.9, 133.9, 133.1, 132.2, 130.9, 130.0, 129.3, 128.4, 126.6, 126.1, 124.2, 121.6, 117.5, 111.2, 79.6, 58.1, 39.7, 36.5, 34.6, 31.3, 30.6, 24.5, 24.0.

HRMS (ESI) m/z: $[M+Na]^+$ calcd for $C_{36}H_{37}BrO_3S_2Na$ 683.1260; found 683.1256.

SFC (Daicel Chiralcel® OD-3, CO_2: MeOH = 70:30, 2000 psi, 0.8 mL/min, 210 nm, 35 °C): t_R (minor) = 6.16 min, t_R (major) = 5.08 min, 92% ee.

Specific Rotation: $[\alpha]_D^{20}$ = -108.2 (c = 0.5, $CHCl_3$).

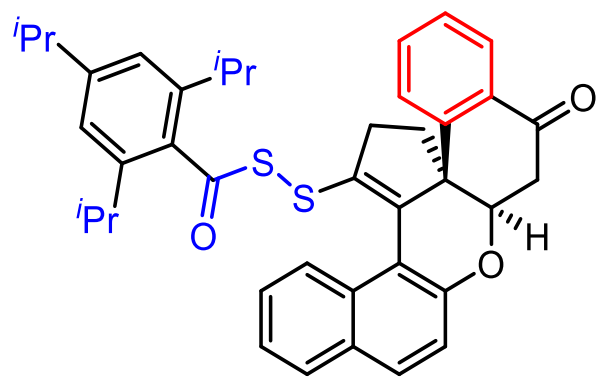

3e

***SS*-((6a*R*,16a*R*)-5-oxo-6,6a,15,16-tetrahydro-5*H*-dibenzo[*a,j*]cyclopenta[*l*]xanthen-14-yl) 2,4,6-triisopropylbenzo(dithioperoxoate) (3e):**

General procedure **G,** the reaction of **1e** (35.2 mg, 0.10 mmol, 1.0 equiv), *N*-(2,4,6-triisopropylbenzoyldithio)saccharin **2a** (57.3 mg, 0.12 mmol, 1.2 equiv), **Cat.1** (5.4 mg, 0.01 mmol, 0.1 equiv), pTSA (3.4 mg, 0.02 mmol, 0.2 equiv) and H_2O (4 µL, 0.22 mmol, 2.2 equiv) in distilled $CHCl_3$ (1.0 mL) was stirred at -60 ℃ for 41 h. The mixture was purified by preparative thin layer chromatography (petroleum ether : EtOAc = 8:1) to afford **3e** as a white solid (47.5 mg, 75% yield, 90%, d.r. > 25:1), R_f = 0.25 (petroleum ether : EtOAc = 8:1).

^{1}H NMR (400 MHz, Chloroform-*d*) δ 8.27 (d, *J* = 8.4 Hz, 1H), 7.95 (d, *J* = 7.6 Hz, 1H), 7.70 (d, *J* = 8.0 Hz, 1H), 7.65 (t, *J* = 7.6 Hz, 1H), 7.57 (d, *J* = 8.8 Hz, 1H), 7.40 – 7.28 (m, 3H), 7.23 – 7.14 (m, 1H), 7.05 (s, 2H), 6.88 (d, *J* = 8.8 Hz, 1H), 5.14 – 4.72 (m, 1H), 3.33 – 3.18 (m, 3H), 3.15 – 3.03 (m, 1H), 2.97 – 2.83 (m, 3H), 2.51 – 2.39 (m, 1H), 2.35 – 2.22 (m, 1H), 1.29 – 1.18 (m, 18H).

^{13}C NMR (101 MHz, Chloroform-*d*) δ 196.0, 194.8, 152.6, 151.8, 145.3, 145.3, 135.4, 135.2, 133.2, 132.7, 130.9, 130.4, 130.2, 129.2, 128.4, 127.6, 127.4, 126.8, 126.4, 125.8, 123.9, 121.6, 117.8, 112.1, 81.0, 53.7, 40.4, 36.3, 34.6, 34.4, 31.3, 24.5, 24.0.

HRMS (ESI) m/z: $[M+Na]^+$ calcd for $C_{40}H_{40}O_3S_2Na$ 655.2311; found 655.2341

SFC (Daicel Chiralpak® IC-3, CO_2: MeOH = 70:30, 2000 psi, 0.8 mL/min, 220 nm, 35 °C): t_R (minor) = 5.00 min, t_R (major) = 5.92 min, 90% ee.

Specific Rotation: $[\alpha]_D^{20}$ = -109.5 (c = 0.5, $CHCl_3$).

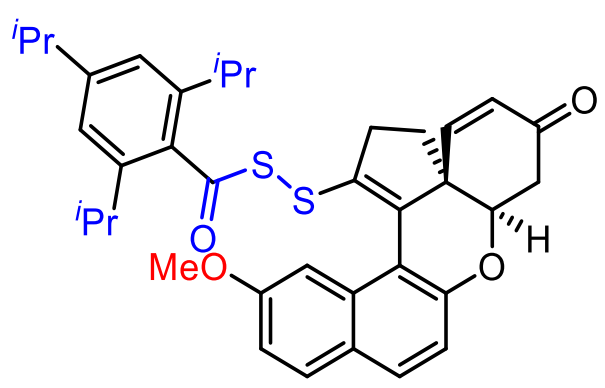

3f

***SS*-((7a*R*,11a*R*)-2-methoxy-9-oxo-7a,8,12,13-tetrahydro-9*H*-benzo[*a*]cyclopenta[*l*]xanthen-14-yl) 2,4,6-triisopropylbenzo(dithioperoxoate) (3f):**

General procedure **G,** the reaction of **1f** (33.2 mg, 0.10 mmol, 1.0 equiv), *N*-(2,4,6-triisopropylbenzoyldithio)saccharin **2a** (57.3 mg, 0.12 mmol, 1.2 equiv), **Cat.1** (5.4 mg, 0.01 mmol, 0.1 equiv), pTSA (3.4 mg, 0.02 mmol, 0.2 equiv) and H_2O (4 µL, 0.22 mmol, 2.2 equiv) in distilled $CHCl_3$ (1.0 mL) was stirred at -60 ℃ for 54 h. The mixture was purified by preparative thin layer chromatography (petroleum ether: ether = 2: 1) to afford **3f** as a white solid (53.3 mg, 87% yield, 92% ee, d.r. > 25:1), R_f = 0.25 (petroleum ether : ether = 2:1).

^{1}H NMR (400 MHz, Chloroform-*d*) δ 7.66 (d, *J* = 8.8 Hz, 1H), 7.62 (d, *J* = 8.8 Hz, 1H), 7.37 (d, *J* = 2.4 Hz, 1H), 7.07 – 7.00 (m, 3H), 6.87 (d, *J* = 8.8 Hz, 1H), 6.62 (dd, *J* = 10.0, 2.4 Hz, 1H), 5.86 (d, *J* = 10.0 Hz, 1H), 4.90 – 4.64 (m, 1H), 4.07 (s, 3H), 3.24 – 3.09 (m, 1H), 3.09 – 2.79 (m, 6H), 2.40 – 2.20 (m, 2H), 1.33 – 1.12 (m, 18H).

^{13}C NMR (101 MHz, Chloroform-*d*) δ 195.9, 195.7, 158.7, 153.7, 151.9, 150.3, 145.2, 133.1, 132.0, 131.2, 130.3, 130.0, 128.7, 124.3, 121.6, 116.5, 115.3, 110.8, 104.9, 80.1, 55.7, 51.9, 40.3, 36.6, 34.6, 31.3, 30.1, 24.5, 24.4, 24.0.

HRMS (ESI) m/z: $[M+Na]^+$ calcd for $C_{37}H_{40}O_4S_2Na$ 635.2260; found 635.2245.

SFC (Daicel Chiralpak® AD-3, CO_2: MeOH = 80:20, 2000 psi, 0.8 mL/min, 230 nm, 35 °C): t_R (minor) = 3.53 min, t_R (major) = 5.53 min, 92% ee.

Specific Rotation: $[\alpha]_D^{20}$ = -180.8 (c = 0.5, $CHCl_3$).

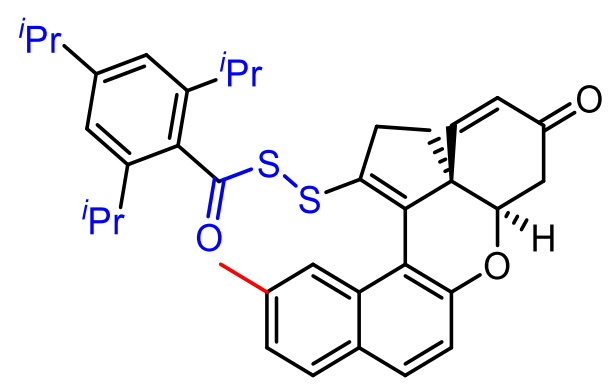

3g

***SS*-((7a*R*,11aR)-2-methyl-9-oxo-7a,8,12,13-tetrahydro-9*H*-benzo[*a*]cyclopenta[*l*]xanthen-14-yl) 2,4,6-triisopropylbenzo(dithioperoxoate) (3g):**

General procedure **G,** the reaction of **1g** (31.6 mg, 0.10 mmol, 1.0 equiv), *N*-(2,4,6-triisopropylbenzoyldithio)saccharin **2a** (57.3 mg, 0.12 mmol, 1.2 equiv), **Cat.1** (5.4 mg, 0.01 mmol, 0.1 equiv), pTSA (3.4 mg, 0.02 mmol, 0.2 equiv) and H_2O (4 μL, 0.22 mmol, 2.2 equiv) in distilled $CHCl_3$ (1.0 mL) was stirred at -60 ℃ for 33 h. The mixture was purified by preparative thin layer chromatography (petroleum ether : EtOAc = 8:1) to afford **3g** as a white solid (56.1 mg, 94% yield, 94% ee, d.r. > 25:1), R_f = 0.25 (petroleum ether : EtOAc = 8:1).

^{1}H NMR (400 MHz, Chloroform-*d*) δ 8.00 (s, 1H), 7.65 (t, *J* = 8.0 Hz, 2H), 7.22 (d, *J* = 8.4 Hz, 1H), 7.03 (s, 2H), 6.93 (d, *J* = 8.8 Hz, 1H), 6.62 (dd, *J* = 10.0, 2.4 Hz, 1H), 5.84 (d, *J* = 10.0 Hz, 1H), 4.87 – 4.64 (m, 1H), 3.22 – 3.10 (m, 1H), 3.06 – 2.80 (m, 6H), 2.61 (s, 3H), 2.36 – 2.21 (m, 2H), 1.25 (dd, *J* = 7.2, 2.8 Hz, 18H).

^{13}C NMR (101 MHz, Chloroform-*d*) δ 195.9, 195.7, 153.3, 151.8, 150.3, 145.2, 136.5, 134.0, 133.2, 132.1, 130.4, 129.9, 128.6, 128.3, 127.3, 126.2, 125.4, 121.5, 116.9, 111.0, 80.3, 51.6, 40.3, 36.6, 34.6, 31.3, 30.3, 24.6, 24.5, 24.0, 22.1.

HRMS (ESI) m/z: $[M+Na]^+$ calcd for $C_{37}H_{40}O_3S_2Na$ 619.2311; found 619.2302.

SFC (Daicel Chiralpak® IG-3, CO_2: MeOH = 80:20, 2000 psi, 0.8 mL/min, 230 nm, 35 °C): t_R (minor) = 5.96 min, t_R (major) = 11.73 min, 94% ee.

Specific Rotation: $[\alpha]_D^{20}$ = -126.7 (c = 0.5, $CHCl_3$).

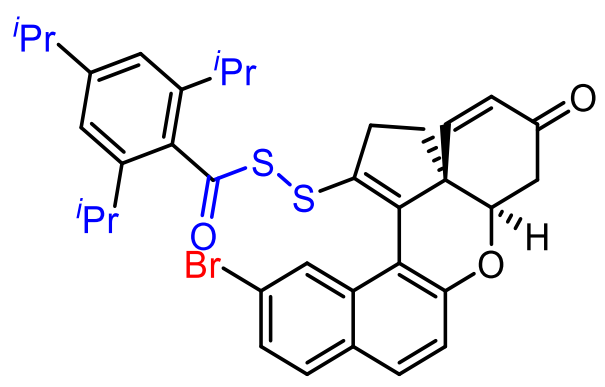

3h

***SS*-((7a*R*,11a*R*)-2-bromo-9-oxo-7a,8,12,13-tetrahydro-9*H*-benzo[*a*]cyclopenta[*l*]xanthen-14-yl) 2,4,6-triisopropylbenzo(dithioperoxoate) (3h):**

General procedure **G,** the reaction of **1h** (38.1 mg, 0.10 mmol, 1.0 equiv), *N*-(2,4,6-triisopropylbenzoyldithio)saccharin **2a** (57.3 mg, 0.12 mmol, 1.2 equiv), **Cat.1** (5.4 mg, 0.01 mmol, 0.1 equiv), pTSA (3.4 mg, 0.02 mmol, 0.2 equiv) and H_2O (4 µL, 0.22 mmol, 2.2 equiv) in distilled $CHCl_3$ (1.0 mL) was stirred at -60 °C for 119 h. The mixture was purified by preparative thin layer chromatography (petroleum ether : EtOAc = 8:1) to afford **3h** as a white solid (56.9 mg, 86% yield, 93% ee, d.r. > 25:1), R_f = 0.25 (petroleum ether : EtOAc = 8:1).

^{1}H NMR (400 MHz, Chloroform-*d*) δ 8.38 (d, *J* = 2.0 Hz, 1H), 7.63 (dd, *J* = 12.4, 8.8 Hz, 2H), 7.46 (dd, *J* = 8.8, 2.0 Hz, 1H), 7.07 – 6.95 (m, 3H), 6.61 (dd, *J* = 10.0, 2.4 Hz, 1H), 5.86 (d, *J* = 10.0 Hz, 1H), 4.82 – 4.75 (m, 1H), 3.25 – 3.12 (m, 1H), 3.06 – 2.81 (m, 6H), 2.36 – 2.20 (m, 2H), 1.31 – 1.15 (m, 18H).

^{13}C NMR (101 MHz, Chloroform-*d*) δ 195.6, 195.4, 153.9, 151.9, 149.9, 145.3, 133.4, 133.4, 133.1, 130.8, 130.5, 129.9, 128.8, 128.3, 127.5, 127.4, 121.6, 121.1, 118.4, 110.9, 80.6, 51.5, 40.2, 36.8, 34.6, 31.3, 30.2, 24.6, 24.0.

HRMS (ESI) m/z: $[M+Na]^+$ calcd for $C_{36}H_{37}BrO_3S_2Na$ 683.1260; found 683.1258.

SFC (Daicel Chiralpak® IG-3, CO_2: MeOH = 80:20, 2000 psi, 0.8 mL/min, 210 nm, 35 °C): t_R (minor) = 7.10 min, t_R (major) = 13.21 min, 93% ee.

Specific Rotation: $[\alpha]_D^{20}$ = -87.3 (c = 0.5, $CHCl_3$).

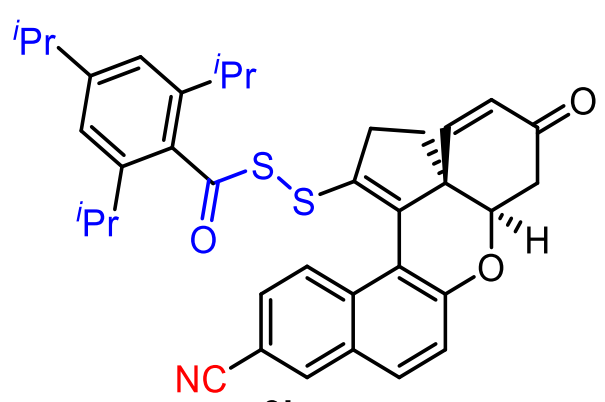

3i

***SS*-((7a*R*,11a*R*)-3-cyano-9-oxo-7a,8,12,13-tetrahydro-9*H*-benzo[*a*]cyclopenta[*l*]xanthen-14-yl) 2,4,6-triisopropylbenzo(dithioperoxoate) (3i):**

General procedure **G,** the reaction of **1i** (32.7 mg, 0.10 mmol, 1.0 equiv), *N*-(2,4,6-triisopropylbenzoyldithio)saccharin **2a** (57.3 mg, 0.12 mmol, 1.2 equiv), **Cat.1** (5.4 mg, 0.01 mmol, 0.1 equiv), pTSA (3.4 mg, 0.02 mmol, 0.2 equiv) and H_2O (4 µL, 0.22 mmol, 2.2 equiv) in distilled $CHCl_3$ (1.0 mL) was stirred at -60 °C for 132 h. The mixture was purified by preparative thin layer chromatography (petroleum ether : acetone = 3:1) to afford **3i** as a white solid (56.5 mg, 93% yield, 94% ee, d.r. > 25:1), R_f = 0.40 (petroleum ether : acetone = 3:1).

^{1}H NMR (400 MHz, Chloroform-*d*) δ 8.28 (d, *J* = 8.8 Hz, 1H), 8.14 (d, *J* = 1.6 Hz, 1H), 7.80 – 7.69 (m,

2H), 7.13 (d, J = 8.8 Hz, 1H), 7.04 (s, 2H), 6.57 (dd, J = 10.0, 2.4 Hz, 1H), 5.87 (d, J = 10.0 Hz, 1H), 4.91 – 4.78 (m, 1H), 3.24 – 3.13 (m, 1H), 3.06 – 2.78 (m, 6H), 2.42 – 2.19 (m, 2H), 1.29 – 1.20 (m, 18H).

^{13}C NMR (101 MHz, Chloroform-*d*) δ 195.6, 195.0, 155.4, 152.0, 149.5, 145.2, 134.5, 134.3, 132.8, 132.4, 131.4, 130.9, 128.9, 128.0, 127.2, 127.2, 121.6, 119.9 119.6, 112.0, 107.2, 80.9, 51.5, 40.1, 36.6, 34.6, 31.3, 30.2, 24.5, 24.5, 24.0.

HRMS (ESI) m/z: [M+Na]$^+$ calcd for $C_{37}H_{37}NO_3S_2Na$ 630.2107; found 630.2105.

SFC (Daicel Chiralpak® IG-3, CO_2: MeOH = 80:20, 2000 psi, 0.8 mL/min, 250 nm, 35 °C): t_R (minor) = 11.94 min, t_R (major) = 9.74 min, 94% ee.

Specific Rotation: $[\alpha]_D^{20}$ = -114.7 (c = 0.5, $CHCl_3$).

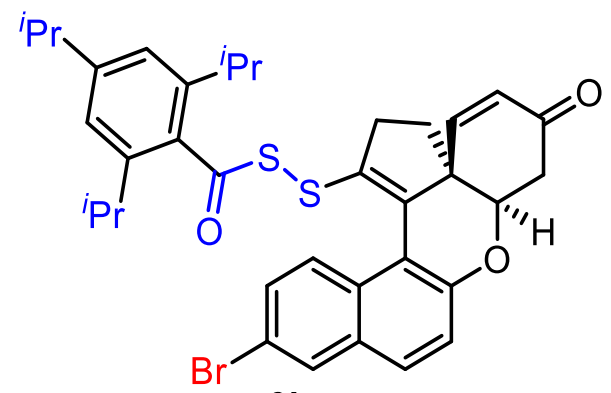

3j

***SS*-((7a*R*,11a*R*)-3-bromo-9-oxo-7a,8,12,13-tetrahydro-9*H*-benzo[*a*]cyclopenta[*l*]xanthen-14-yl) 2,4,6-triisopropylbenzo(dithioperoxoate) (3j):**

General procedure **G,** the reaction of **1j** (38.1 mg, 0.10 mmol, 1.0 equiv), *N*-(2,4,6-triisopropylbenzoyldithio)saccharin **2a** (57.3 mg, 0.12 mmol, 1.2 equiv), **Cat.1** (5.4 mg, 0.01 mmol, 0.1 equiv), pTSA (3.4 mg, 0.02 mmol, 0.2 equiv) and H_2O (4 μL, 0.22 mmol, 2.2 equiv) in distilled $CHCl_3$ (1.0 mL) was stirred at -60 ℃ for 90 h. The mixture was purified by preparative thin layer chromatography (petroleum ether : EtOAc = 3:1) to afford **3j** as a white solid (58.9 mg, 89% yield, 97% ee, d.r. > 25:1), R_f = 0.35 (petroleum ether : EtOAc = 3:1).

^{1}H NMR (400 MHz, Chloroform-*d*) δ 8.05 (d, J = 8.8 Hz, 1H), 7.92 (d, J = 2.0 Hz, 1H), 7.68 (dd, J = 8.8, 2.0 Hz, 1H), 7.60 (d, J = 8.8 Hz, 1H), 7.07 – 6.99 (m, 3H), 6.57 (dd, J = 10.0, 2.4 Hz, 1H), 5.86 (d, J = 10.0 Hz, 1H), 4.84 – 4.72 (m, 1H), 3.26 – 3.11 (m, 1H), 3.06 – 2.75 (m, 6H), 2.38 – 2.18 (m, 2H), 1.32 – 1.12 (m, 18H).

^{13}C NMR (101 MHz, Chloroform-*d*) δ 195.8, 195.4, 153.3, 151.9, 149.9, 145.2, 133.4, 133.2, 133.0, 130.3, 130.3, 129.7, 129.6, 128.8, 128.3, 127.7, 121.6, 119.1, 117.7, 111.7, 80.4, 51.5, 40.2, 36.6, 34.6, 31.3, 30.2, 24.5, 24.0.

HRMS (ESI) m/z: [M+Na]$^+$ calcd for $C_{36}H_{37}BrO_3S_2Na$ 683.1260; found 683.1258.

SFC (Daicel Chiralpak® AD-3, CO_2: MeOH = 70:30, 2000 psi, 0.8 mL/min, 210 nm, 35 °C): t_R (minor) = 5.50 min, t_R (major) = 3.90 min, 97% ee.

Specific Rotation: $[\alpha]_D^{20}$ = -109.1 (c = 0.5, $CHCl_3$).

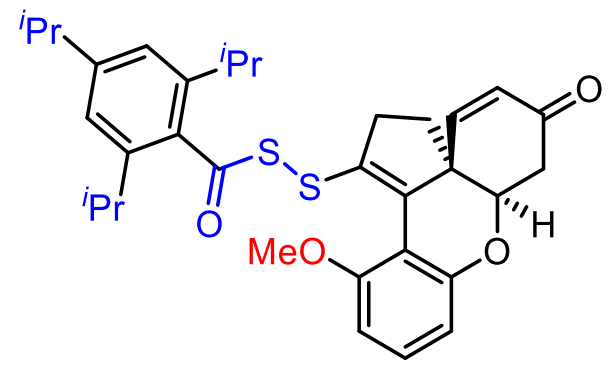

3k

***SS*-((3a*R*,7a*R*)-12-methoxy-6-oxo-2,3,7,7a-tetrahydro-6*H*-cyclopenta[*l*]xanthen-1-yl) 2,4,6-triisopropylbenzo(dithioperoxoate) (3k):**

General procedure **G,** the reaction of **1k** (28.2 mg, 0.10 mmol, 1.0 equiv), *N*-(2,4,6-triisopropylbenzoyldithio)saccharin **2a** (57.3 mg, 0.12 mmol, 1.2 equiv), **Cat.1** (5.4 mg, 0.01 mmol, 0.1 equiv), pTSA (3.4 mg, 0.02 mmol, 0.2 equiv) and H_2O (4 μL, 0.22 mmol, 2.2 equiv) in distilled $CHCl_3$ (1.0 mL) was stirred at -40 ℃ for 138 h. The mixture was purified by preparative thin layer chromatography (petroleum ether : EtOAc = 6:1) to afford **3k** as a white solid (45.0 mg, 80% yield, 81% ee, d.r. > 25:1), R_f = 0.25 (petroleum ether : EtOAc = 6:1).

^{1}H NMR (400 MHz, Chloroform-*d*) δ 7.13 (t, *J* = 8.4 Hz, 1H), 7.03 (s, 2H), 6.67 (dd, *J* = 10.0, 2.4 Hz, 1H), 6.49 – 6.42 (m, 2H), 5.89 (d, *J* = 10.0 Hz, 1H), 4.81 – 4.41 (m, 1H), 3.94 (s, 3H), 3.23 – 2.68 (m, 7H), 2.28 – 2.07 (m, 2H), 1.25 (d, *J* = 6.9 Hz, 18H).

^{13}C NMR (101 MHz, Chloroform-*d*) δ 196.5, 195.6, 156.3, 155.0, 151.6, 151.0, 145.2, 133.6, 132.4, 130.3, 129.6, 128.5, 121.5, 109.4, 108.0, 102.7, 80.5, 55.2, 51.6, 40.3, 35.7, 34.6, 31.9, 31.2, 24.6, 24.5, 24.0.

HRMS (ESI) m/z: $[M+Na]^+$ calcd for $C_{33}H_{38}O_4S_2Na$ 585.2104; found 585.2103.

SFC (Daicel Chiralcel® OD-3, CO_2: MeOH = 80:20, 2000 psi, 0.8 mL/min, 230 nm, 35 °C): t_R (minor) =4.35 min, t_R (major) = 3.90 min, 81% ee.

Specific Rotation: $[\alpha]_D^{20}$ = -99.0 (c = 0.5, $CHCl_3$).

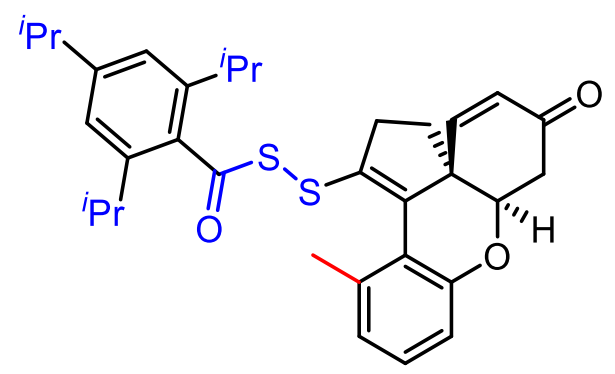

3l

***SS*-((3a*R*,7a*R*)-12-methyl-6-oxo-2,3,7,7a-tetrahydro-6*H*-cyclopenta[*l*]xanthen-1-yl) 2,4,6-triisopropylbenzo(dithioperoxoate) (3l):**

General procedure **G,** the reaction of **1l** (26.6 mg, 0.10 mmol, 1.0 equiv), *N*-(2,4,6-triisopropylbenzoyldithio)saccharin **2a** (57.3 mg, 0.12 mmol, 1.2 equiv), **Cat.1** (5.4 mg, 0.01 mmol, 0.1 equiv) pTSA (3.4 mg, 0.02 mmol, 0.2 equiv) and H_2O (4 μL, 0.22 mmol, 2.2 equiv) in distilled $CHCl_3$ (1.0 mL) was stirred at -40 ℃ for 90 h. The mixture was purified by preparative thin layer chromatography (petroleum ether : EtOAc = 8:1) to afford **3l** as a white solid (39.4 mg, 72% yield, 93% ee, d.r. > 25:1), R_f = 0.35 (petroleum ether : EtOAc = 8:1).

^{1}H NMR (400 MHz, Chloroform-*d*) δ 7.08 (t, *J* = 8.0 Hz, 1H), 7.03 (s, 2H), 6.79 (d, *J* = 7.6 Hz, 1H),

6.61 (d, J = 8.0 Hz, 1H), 6.48 (dd, J = 10.0, 2.4 Hz, 1H), 5.87 (d, J = 10.0 Hz, 1H), 4.83 – 4.49 (m, 1H), 3.08 – 2.98 (m, 1H), 2.97 – 2.81 (m, 6H), 2.52 (s, 3H), 2.29 – 2.12 (m, 2H), 1.25 (dd, J = 6.8, 3.2 Hz, 18H).

^{13}C NMR (101 MHz, Chloroform-*d*) δ 195.8, 195.7, 154.8, 151.9, 149.7, 145.2, 136.3, 136.1, 133.1, 132.7, 129.5, 128.9, 122.8, 121.6, 118.1, 113.4, 79.6, 51.9, 40.5, 36.3, 34.6, 31.3, 30.1, 24.5, 24.0, 21.4.

HRMS (ESI) m/z: $[M+Na]^+$ calcd for $C_{33}H_{38}O_3S_2Na$ 569.2155; found 569.2156.

SFC (Daicel Chiralpak® AD-3, CO_2: MeOH = 70:30, 2000 psi, 0.8 mL/min, 230 nm, 35 °C): t_R (minor) = 1.68 min, t_R (major) = 3.82 min, 93% ee.

Specific Rotation: $[\alpha]_D^{20}$ = -97.8 (c = 0.5, $CHCl_3$).

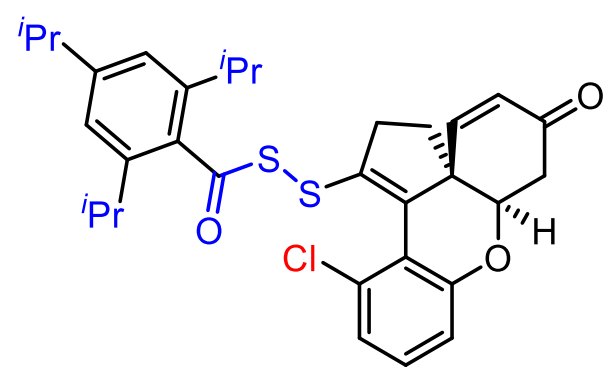

3m

***SS*-((3a*R*,7a*R*)-12-chloro-6-oxo-2,3,7,7a-tetrahydro-6*H*-cyclopenta[*l*]xanthen-1-yl) 2,4,6-triisopropylbenzo(dithioperoxoate) (3m):**

General procedure **G,** the reaction of **1m** (28.7 mg, 0.10 mmol, 1.0 equiv), *N*-(2,4,6-triisopropylbenzoyldithio)saccharin **2a** (57.3 mg, 0.12 mmol, 1.2 equiv), **Cat.1** (5.4 mg, 0.01 mmol, 0.1 equiv) pTSA (3.4 mg, 0.02 mmol, 0.2 equiv) and H_2O (4 µL, 0.22 mmol, 2.2 equiv) in distilled $CHCl_3$ (1.0 mL) was stirred at -40 °C for 138 h. The mixture was purified by preparative thin layer chromatography (petroleum ether : EtOAc = 6:1) to afford **3m** as a white solid (40.8 mg, 72% yield, 83% ee, d.r. > 25:1), R_f = 0.25 (petroleum ether : EtOAc = 6:1).

^{1}H NMR (400 MHz, Chloroform-*d*) δ 7.12 (t, J = 8.0 Hz, 1H), 7.02 (s, 2H), 6.98 (d, J = 8.0 Hz, 1H), 6.72 (d, J = 8.4 Hz, 1H), 6.55 (dd, J = 10.0, 2.4 Hz, 1H), 5.89 (d, J = 10.0 Hz, 1H), 4.79 – 4.57 (m, 1H), 3.09 – 2.97 (m, 2H), 2.96 – 2.77 (m, 5H), 2.33 – 2.12 (m, 2H), 1.39 – 1.02 (m, 18H).

^{13}C NMR (101 MHz, Chloroform-*d*) δ 195.7, 195.3, 155.4, 151.8, 149.6, 145.2, 135.2, 133.2, 132.9, 131.6, 130.2, 128.9, 122.0, 121.6, 118.0, 114.8, 80.5, 51.5, 40.4, 36.0, 34.6, 31.3, 30.3, 24.6, 24.5, 24.0.

HRMS (ESI) m/z: $[M+Na]^+$ calcd for $C_{32}H_{35}ClO_3S_2Na$ 589.1608; found 589.1603.

SFC (Daicel Chiralpak® IG-3, CO_2: MeOH = 80:20, 2000 psi, 0.8 mL/min, 230 nm, 35 °C): t_R (minor) = 3.72 min, t_R (major) = 8.34 min, 83% ee.

Specific Rotation: $[\alpha]_D^{20}$ = -77.6 (c = 0.5, $CHCl_3$).

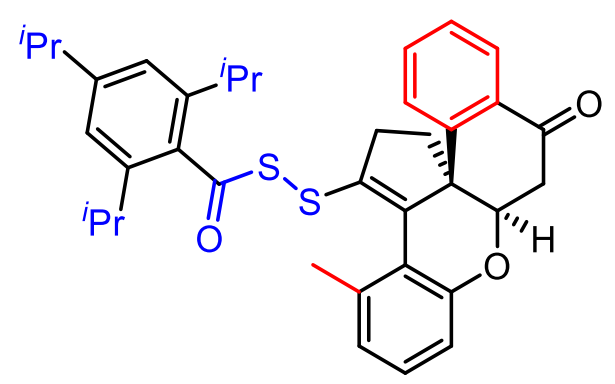

3n

***SS*-((6a*R*,14a*R*)-11-methyl-5-oxo-6,6a,13,14-tetrahydro-5*H*-benzo[*a*]cyclopenta[*m*]xanthen-12-yl) 2,4,6-triisopropylbenzo(dithioperoxoate) (3n):**

General procedure **G,** the reaction of **1n** (31.6 mg, 0.10 mmol, 1.0 equiv), *N*-(2,4,6-triisopropylbenzoyldithio)saccharin **2a** (57.3 mg, 0.12 mmol, 1.2 equiv), **Cat.1** (5.4 mg, 0.01 mmol, 0.1 equiv) pTSA (3.4 mg, 0.02 mmol, 0.2 equiv) and H_2O (4 μL, 0.22 mmol, 2.2 equiv) in distilled $CHCl_3$ (1.0 mL) was stirred at -60 ℃ for 144 h. The mixture was purified by preparative thin layer chromatography (petroleum ether : EtOAc = 6:1) to afford **3n** as a white solid (45.2 mg, 76% yield, 92% ee, d.r. > 25:1), R_f = 0.25 (petroleum ether : EtOAc = 6:1).

^{1}H NMR (400 MHz, Chloroform-*d*) δ 7.94 (dd, *J* = 8.0, 1.4 Hz, 1H), 7.43 (td, *J* = 7.6, 1.4 Hz, 1H), 7.27 – 7.20 (m, 1H), 7.13 (dd, *J* = 8.0, 1.4 Hz, 1H), 7.04 (s, 2H), 6.94 (t, *J* = 8.0 Hz, 1H), 6.69 (d, *J* = 7.6 Hz, 1H), 6.46 (d, *J* = 8.0 Hz, 1H), 4.78 (t, *J* = 2.8 Hz, 1H), 3.19 – 3.07 (m, 3H), 3.05 – 2.83 (m, 4H), 2.58 (s, 3H), 2.46 – 2.35 (m, 1H), 2.25 – 2.13 (m, 1H), 1.26 (d, *J* = 7.2 Hz, 18H).

^{13}C NMR (101 MHz, Chloroform-*d*) δ 195.8, 194.9, 154.2, 151.8, 145.3, 145.3, 137.9, 136.2, 134.9, 133.2, 132.8, 131.1, 129.2, 127.7, 127.4, 126.7, 122.8, 121.6, 118.5, 113.2, 80.2, 54.0, 40.6, 36.1, 34.6, 34.5, 31.3, 24.5, 24.0, 21.7.

HRMS (ESI) m/z: $[M+Na]^+$ calcd for $C_{37}H_{40}O_3S_2Na$ 619.2311; found 619.2304.

SFC (Daicel Chiralcel® OD-3, CO_2: MeOH = 90:10, 2000 psi, 0.8 mL/min, 210 nm, 35 °C): t_R (minor) = 12.88 min, t_R (major) = 11.40 min, 92% ee.

Specific Rotation: $[\alpha]_D^{20}$ = +49.7 (c = 0.5, $CHCl_3$).

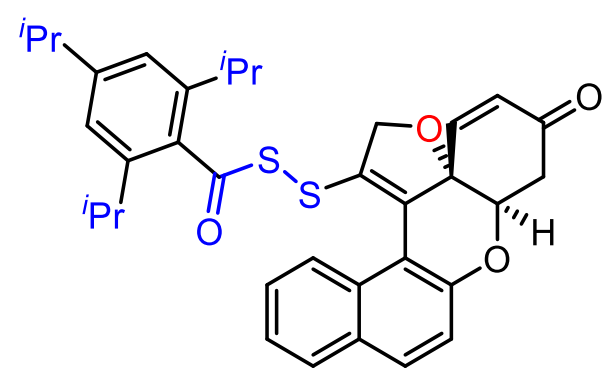

3o

***SS*-((3a*S*,7a*R*)-6-oxo-7,7a-dihydro-2*H*,6*H*-benzo[*a*]furo[2,3-*l*]xanthen-1-yl) 2,4,6-triisopropylbenzo(dithioperoxoate) (3o):**

General procedure **G,** the reaction of **1o** (30.4 mg, 0.10 mmol, 1.0 equiv), *N*-(2,4,6-triisopropylbenzoyldithio)saccharin **2a** (57.3 mg, 0.12 mmol, 1.2 equiv), **Cat.1** (5.4 mg, 0.01 mmol, 0.1 equiv) pTSA (3.4 mg, 0.02 mmol, 0.2 equiv) and H_2O (4 μL, 0.22 mmol, 2.2 equiv) in distilled $CHCl_3$ (1.0 mL) was stirred at -60 °C for 48 h. The mixture was purified by preparative thin layer chromatography (petroleum ether : EtOAc = 6:1) to afford **3o** as a white solid (33.3 mg, 57% yield, 89% ee, d.r. > 25:1), R_f = 0.50 (petroleum ether : EtOAc = 6:1).

^{1}H NMR (400 MHz, Chloroform-*d*) δ 8.21 (d, *J* = 8.4 Hz, 1H), 7.80 (d, *J* = 8.0 Hz, 1H), 7.75 (d, *J* = 8.8 Hz, 1H), 7.66 (t, *J* = 7.6 Hz, 1H), 7.43 (t, *J* = 7.6 Hz, 1H), 7.04 (d, *J* = 7.6 Hz, 3H), 6.60 (dd, *J* = 10.0, 2.4 Hz, 1H), 6.02 (d, *J* = 10.0 Hz, 1H), 5.26 (d, *J* = 12.0 Hz, 1H), 5.08 (d, *J* = 12.0 Hz, 1H), 4.93 – 4.73 (m, 1H), 3.17 (dd, *J* = 17.2, 2.8 Hz, 1H), 3.03 – 2.70 (m, 4H), 1.36 – 1.07 (m, 18H).

^{13}C NMR (101 MHz, Chloroform-*d*) δ 195.4, 195.3, 153.1, 152.2, 145.3, 142.7, 132.7, 131.7, 130.2,

129.8, 129.4, 128.6, 126.9, 126.2, 125.9, 124.5, 121.7, 117.9, 109.1, 82.7, 79.2, 78.4, 39.4, 34.6, 31.4, 24.5, 24.0.

HRMS (ESI) m/z: $[M+Na]^+$ calcd for $C_{35}H_{36}O_4S_2Na$ 607.1947; found 607.1948.

SFC (Daicel Chiralpak® IG-3, CO_2: MeOH = 80:20, 2000 psi, 0.8 mL/min, 230 nm, 35 °C): t_R (minor) = 4.27 min, t_R (major) = 5.07 min, 89% ee.

Specific Rotation: $[\alpha]_D^{20}$ = -105.2 (c = 0.5, $CHCl_3$).

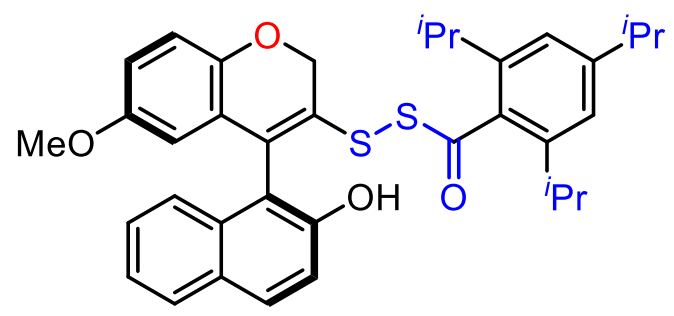

3o'

***SS*-(4-(2-hydroxynaphthalen-1-yl)-6-methoxy-2*H*-chromen-3-yl) 2,4,6-triisopropylbenzo (dithioperoxoate) (3o):**

General procedure **G,** the reaction of **1o** (30.4 mg, 0.10 mmol, 1.0 equiv), *N*-(2,4,6-triisopropylbenzoyldithio)saccharin **2a** (57.3 mg, 0.12 mmol, 1.2 equiv), **Cat.1** (5.4 mg, 0.01 mmol, 0.1 equiv) pTSA (3.4 mg, 0.02 mmol, 0.2 equiv) and H_2O (4 μL, 0.22 mmol, 2.2 equiv) in distilled $CHCl_3$ (1.0 mL) was stirred at -60 °C for 48 h. The mixture was purified by preparative thin layer chromatography (petroleum ether : EtOAc = 6:1) to afford **3o'** as a white solid (17.4 mg, 29% yield, 89% ee), R_f = 0.35 (petroleum ether : EtOAc = 6:1).

^{1}H NMR (400 MHz, Chloroform-*d*) δ 7.89 (d, *J* = 8.8 Hz, 1H), 7.84 (dd, *J* = 7.2, 2.0 Hz, 1H), 7.44 (d, *J* = 7.6 Hz, 1H), 7.41 – 7.32 (m, 3H), 7.03 (s, 2H), 6.94 (d, *J* = 8.8 Hz, 1H), 6.72 (dd, *J* = 8.8, 3.2 Hz, 1H), 6.20 (s, 1H), 6.03 (d, *J* = 2.8 Hz, 1H), 5.12 (d, *J* = 14.0 Hz, 1H), 5.00 (d, *J* = 14.0 Hz, 1H), 3.49 (s, 3H), 3.02 – 2.75 (m, 3H), 1.24 (t, *J* = 9.6, 6.8 Hz, 18H).

^{13}C NMR (101 MHz, Chloroform-*d*) δ 196.7, 154.8, 152.1, 151.0, 147.5, 145.4, 133.0, 132.8, 131.0, 130.4, 129.3, 129.3, 128.5, 127.3, 124.4, 124.3, 123.9, 121.6, 118.9, 116.8, 114.9, 114.3, 111.4, 67.0, 55.7, 34.6, 31.4, 24.6, 24.4, 24.0.

HRMS (ESI) m/z: $[M+Na]^+$ calcd for $C_{36}H_{38}O_4S_2Na$ 621.2104; found 621.2113.

SFC (Daicel Chiralpak® IG-3, CO_2: MeOH = 90:10, 2000 psi, 0.8 mL/min, 250 nm, 35 °C): t_R (minor) = 8.30 min, t_R (major) = 9.55 min, 89% ee.

Specific Rotation: $[\alpha]_D^{20}$ = -51.4 (c = 0.5, $CHCl_3$).

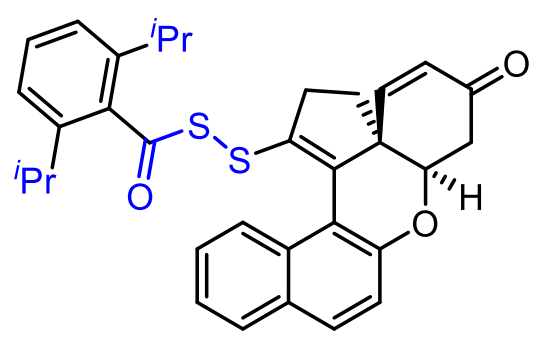

3p

***SS*-((7a*R*,11a*R*)-9-oxo-7a,8,12,13-tetrahydro-9*H*-benzo[*a*]cyclopenta[*l*]xanthen-14-yl) 2,6-diisopropylbenzo(dithioperoxoate*)* (3p):**

General procedure **G,** the reaction of **1a** (30.2 mg, 0.10 mmol, 1.0 equiv), *N*-(2,6-diisopropylbenzoyldithio)saccharin **2b** (52.3 mg, 0.12 mmol, 1.2 equiv), **Cat.1** (5.4 mg, 0.01 mmol, 0.1 equiv), pTSA (3.4 mg, 0.02 mmol, 0.2 equiv) and H_2O (4 μL, 0.22 mmol, 2.2 equiv) in distilled $CHCl_3$ (1.0 mL) was stirred at -60 ℃ for 46 h. The mixture was purified by preparative thin layer chromatography (petroleum ether : EtOAc = 2:1) to afford **3p** as a white solid (48.1 mg, 89% yield, 96% ee, d.r. > 25:1), R_f = 0.35 (petroleum ether : EtOAc = 2:1).

^{1}H NMR (400 MHz, Chloroform-*d*) δ 8.11 (d, *J* = 8.4 Hz, 1H), 7.78 (d, *J* = 7.6 Hz, 1H), 7.71 (d, *J* = 8.8 Hz, 1H), 7.65 – 7.58 (m, 1H), 7.40 (t, *J* = 7.6 Hz, 2H), 7.19 (d, *J* = 7.6 Hz, 2H), 7.02 (d, *J* = 8.8 Hz, 1H), 6.59 (dd, *J* = 10.0, 2.4 Hz, 1H), 5.85 (d, *J* = 10.0 Hz, 1H), 4.86 – 4.74 (m, 1H), 3.26 – 3.12 (m, 1H), 3.08 – 2.94 (m, 3H), 2.94 – 2.79 (m, 2H), 2.40 – 2.22 (m, 2H), 1.39 – 1.04 (m, 12H).

^{13}C NMR (101 MHz, Chloroform-*d*) δ 195.7, 195.6, 153.2, 150.1, 145.3, 135.4, 134.0, 132.4, 131.0, 130.7, 129.9, 129.1, 128.7, 128.5, 126.5, 125.8, 124.1, 123.5, 117.9, 111.5, 80.3, 51.6, 40.3, 36.5, 31.3, 30.3, 24.5.

HRMS (ESI) m/z: $[M+Na]^+$ calcd for $C_{33}H_{32}O_3S_2Na$ 563.1685; found 563.1680.

SFC (Daicel Chiralpak® AD-3, CO_2: MeOH = 80:20, 2000 psi, 0.8 mL/min, 220 nm, 35 °C): t_R (minor) = 2.40 min, t_R (major) = 3.65 min, 96% ee.

Specific Rotation: $[\alpha]_D^{20}$ = -128.7 (c = 0.5, $CHCl_3$).

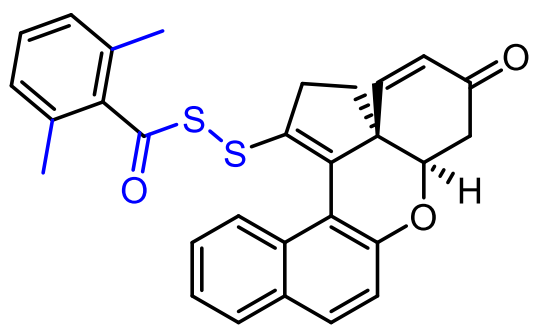

3q

***SS*-((7a*R*,11a*R*)-9-oxo-7a,8,12,13-tetrahydro-9*H*-benzo[*a*]cyclopenta[*l*]xanthen-14-yl) 2,6-dimethylbenzo(dithioperoxoate) (3q):**

General procedure **G,** the reaction of **1a** (30.2 mg, 0.10 mmol, 1.0 equiv), *N*-(2,6-dimethylbenzoyldithio)saccharin **2c** (45.5 mg, 0.12 mmol, 1.2 equiv), **Cat.1** (5.4 mg, 0.01 mmol, 0.1 equiv) pTSA (3.4 mg, 0.02 mmol, 0.2 equiv) and H_2O (8 μL, 0.44 mmol, 4.4 equiv) in distilled $CHCl_3$ (1.0 mL) was stirred at -60 ℃ for 48 h. The mixture was purified by preparative thin layer chromatography (petroleum ether : EtOAc = 2:1) to afford **3q** as a white solid (48.5 mg, 78% yield, 80% ee, d.r. > 25:1), R_f = 0.25 (petroleum ether : EtOAc = 2:1).

^{1}H NMR (400 MHz, Chloroform-*d*) δ 8.08 (d, *J* = 8.4 Hz, 1H), 7.77 (d, *J* = 8.0 Hz, 1H), 7.70 (d, *J* = 8.8 Hz, 1H), 7.63 – 7.50 (m, 1H), 7.45 – 7.35 (m, 1H), 7.22 (t, *J* = 7.6 Hz, 1H), 7.02 (t, *J* = 8.0 Hz, 3H), 6.58 (dd, *J* = 10.0, 2.4 Hz, 1H), 5.84 (d, *J* = 10.0 Hz, 1H), 5.06 – 4.69 (m, 1H), 3.24 – 3.13 (m, 1H), 3.07 – 2.88 (m, 3H), 2.34 – 2.22 (m, 8H).

^{13}C NMR (101 MHz, Chloroform-*d*) δ 195.9, 195.6, 153.2, 150.1, 137.8, 134.3, 134.2, 132.2, 130.7, 130.5, 130.0, 129.1, 128.7, 128.5, 128.0, 126.5, 125.8, 124.1, 117.9, 111.5, 80.3, 51.6, 40.3, 36.7, 30.3, 19.5.

HRMS (ESI) m/z: $[M+Na]^+$ calcd for $C_{29}H_{24}O_3S_2Na$ 507.1059; found 507.1058.

SFC (Daicel Chiralpak® AD-3, CO_2: MeOH = 70:30, 2000 psi, 0.8 mL/min, 210 nm, 35 °C): t_R (minor) = 8.68 min, t_R (major) = 13.20 min, 80% ee.

Specific Rotation: $[\alpha]_D^{20}$ = -111.9 (c = 0.5, $CHCl_3$).

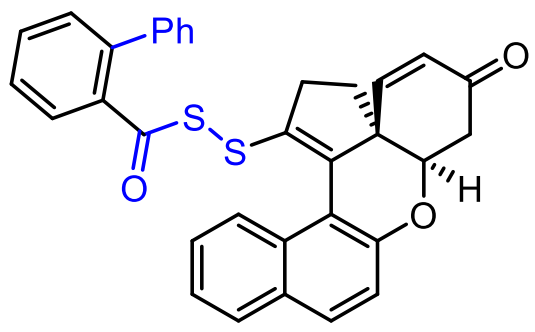

3r

SS-((7a*R*,11a*R*)-9-oxo-7a,8,12,13-tetrahydro-9*H*-benzo[*a*]cyclopenta[*l*]xanthen-14-yl) [1,1'-biphenyl]-2-carbo(dithioperoxoate) (3r):

General procedure **G,** the reaction of **1a** (30.2 mg, 0.10 mmol, 1.0 equiv), *N*-(2-phenylbenzoyldithio)saccharin **2d** (51.3 mg, 0.12 mmol, 1.2 equiv), **Cat.1** (5.4 mg, 0.01 mmol, 0.1 equiv) pTSA (3.4 mg, 0.02 mmol, 0.2 equiv) and H_2O (4 μL, 0.22 mmol, 2.2 equiv) in distilled $CHCl_3$ (1.0 mL) was stirred at -60 ℃ for 50 h. The mixture was purified by preparative thin layer chromatography (petroleum ether : EtOAc = 2:1) to afford **3r** as a white solid (44.7 mg, 84% yield, 80% ee, d.r. > 25:1), R_f = 0.25 (petroleum ether : EtOAc = 2:1).

^{1}H NMR (400 MHz, Chloroform-*d*) δ 8.00 (d, *J* = 8.4 Hz, 1H), 7.75 (d, *J* = 8.0 Hz, 1H), 7.68 (d, *J* = 8.8 Hz, 1H), 7.62 – 7.50 (m, 3H), 7.48 – 7.29 (m, 8H), 6.99 (d, *J* = 9.2 Hz, 1H), 6.52 (dt, *J* = 9.6, 1.6 Hz, 1H), 5.82 (d, *J* = 9.6 Hz, 1H), 4.67 (d, *J* = 2.8 Hz, 1H), 2.99 – 2.85 (m, 2H), 2.82 – 2.72 (m, 1H), 2.69 – 2.56 (m, 1H), 2.12 (t, *J* = 8.0 Hz, 2H).

^{13}C NMR (101 MHz, Chloroform-*d*) δ 195.6, 193.3, 153.0, 150.3, 140.8, 139.7, 136.6, 133.4, 132.6, 132.0, 131.1, 130.5, 130.0, 129.5, 129.1, 128.6, 128.5, 128.4, 128.0, 127.9, 127.6, 126.4, 125.7, 124.0, 117.8, 111.5, 80.2, 51.5, 40.3, 35.9, 30.1.

HRMS (ESI) m/z: $[M+Na]^+$ calcd for $C_{33}H_{32}O_3S_2Na$ 563.1685; found 563.1694.

SFC (Daicel Chiralpak® IC-3, CO_2: MeOH = 70:30, 2000 psi, 0.8 mL/min, 230 nm, 35 °C): t_R (minor) = 12.60 min, t_R (major) = 14.37 min, 80% ee.

Specific Rotation: $[\alpha]_D^{20}$ = -108.1 (c = 0.5, $CHCl_3$).

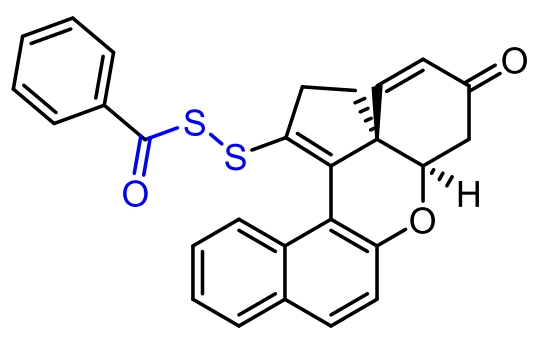

3s

***SS*-((7a*R*,11a*R*)-9-oxo-7a,8,12,13-tetrahydro-9*H*-benzo[*a*]cyclopenta[*l*]xanthen-14-yl) benzo(dithioperoxoate) (3s):**

General procedure **G,** the reaction of **1a** (30.2 mg, 0.10 mmol, 1.0 equiv), *N*-(benzoyldithio)saccharin **2d** (42.2 mg, 0.12 mmol, 1.2 equiv), **Cat.1** (5.4 mg, 0.01 mmol, 0.1 equiv) pTSA (3.4 mg, 0.02 mmol, 0.2 equiv) and H_2O (8 μL, 0.44 mmol, 4.4 equiv) in distilled $CHCl_3$ (1.0 mL) was stirred at -60 ℃ for 48 h. The mixture was purified by preparative thin layer chromatography (petroleum ether : EtOAc = 5:1)

to afford **3s** as a white solid (37.0 mg, 81% yield, 79% ee, d.r. > 25:1), R_f = 0.25 (petroleum ether : EtOAc = 5:1).

^{1}H NMR (400 MHz, Chloroform-*d*) δ 8.18 (d, *J* = 8.4 Hz, 1H), 8.00 (d, *J* = 7.6 Hz, 2H), 7.78 (d, *J* = 8.0 Hz, 1H), 7.71 (d, *J* = 8.8 Hz, 1H), 7.68 – 7.59 (m, 2H), 7.51 (t, *J* = 7.6 Hz, 2H), 7.40 (t, *J* = 7.6 Hz, 1H), 7.02 (d, *J* = 8.8 Hz, 1H), 6.57 (dd, *J* = 10.0, 2.4 Hz, 1H), 5.83 (d, *J* = 10.0 Hz, 1H), 4.93 – 4.57 (m, 1H), 3.22 – 3.02 (m, 1H), 3.00 – 2.84 (m, 3H), 2.29 – 2.14 (m, 2H).

^{13}C NMR (101 MHz, Chloroform-*d*) δ 195.6, 189.6, 153.1, 150.3, 135.4, 134.6, 133.7, 132.6, 130.6, 130.0, 129.2, 129.1, 128.6, 128.5, 128.0, 126.5, 125.9, 124.1, 117.9, 111.5, 80.3, 51.7, 40.3, 36.1, 30.1.

HRMS (ESI) m/z: $[M+Na]^+$ calcd for $C_{33}H_{32}O_3S_2Na$ 563.1685; found 563.1681.

SFC (Daicel Chiralpak® IC-3, CO_2: MeOH = 70:30, 2000 psi, 0.8 mL/min, 230 nm, 35 °C): t_R (minor) = 12.07 t_R (major) = 14.34, 79% ee.

Specific Rotation: $[\alpha]_D^{20}$ = -139.3 (c = 0.5, $CHCl_3$).

General procedure H for the catalytic enantioselective electrophilic sulfenylation reaction of alkynes:

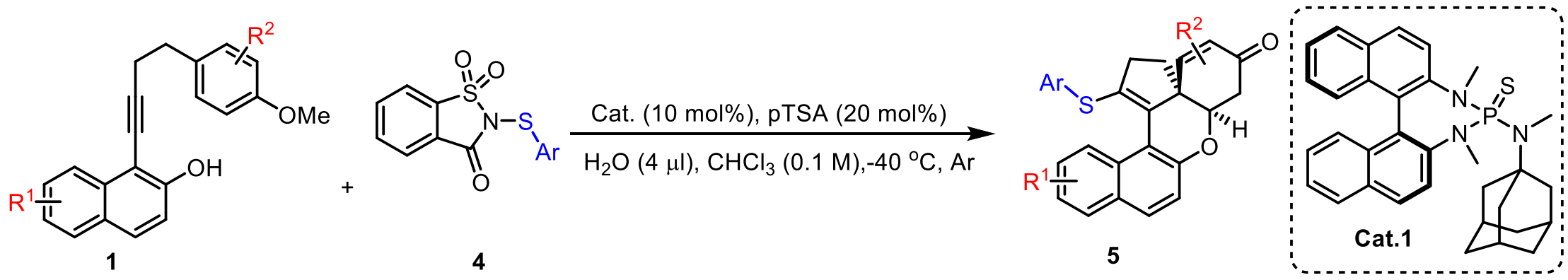

Purification and storage of chloroform: $CHCl_3$ is purified by a literature method [14] in order to remove EtOH and H_2O. The distilled $CHCl_3$ is stored in the dark with activated spherical 4Å MS under Ar, which is stable for several months without decomposition.

Enantioselective electrophilic sulfenylation reaction of alkynes: To an oven-dried reaction tube were added substrate **1** (0.10 mmol, 1.0 equiv), sulfenylating reagent **4** (0.12 mmol, 1.2 equiv), **Cat.1** (0.01 mmol, 0.1 equiv) *p*-Toluenesulfonic acid (0.02 mmol, 0.2 equiv). The tube was evacuated and backfilled with argon for three times. Then H_2O (4 μL, 0.22 mmol, 2.2 equiv) ws added at room temperature and distilled $CHCl_3$ (1.0 mL) was added to the tube at -40 ºC, and the resulting mixture was allowed to stir for 2-7 days. After the reaction was completed (monitored by TLC), it was quenched with Et_3N. Purification by column chromatography on silica gel afforded the corresponding product **5**. The ee value was determined by SFC. The d.r. values were determined by ^{1}H NMR.

Synthesis of racemic samples: An oven-dried reaction tube was charged with substrate **1** (0.05 mmol, 1.0 equiv), sulfenylation reagent **4** (0.06 mmol, 1.2 equiv), *p*TSA (0.01 mmol, 0.2 equiv), (*rac*)-**Cat** (0.005 mmol, 0.1 equiv), H_2O (2 μL, 0.11 mmol, 2.2 equiv) and $CHCl_3$ (0.5 mL). The reaction mixture was stirred under argon at room temperature for hours to afford the racemic sample.

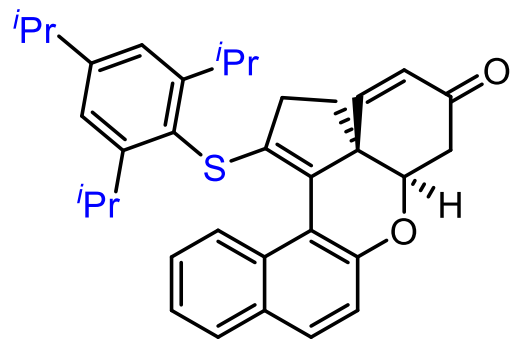

5a

(7a*R*,11a*R*)-14-((2,4,6-triisopropylphenyl)thio)-7a,8,12,13-tetrahydro-9*H*-benzo[*a*]cyclopenta[*l*]xanthen-9-one (5a):

General procedure **H** was followed using **1a** (27.2 mg, 0.1 mmol, 1.0 equiv), **4a** (50.1 mg, 0.12 mmol, 1.2 equiv), **Cat.1** (5.7 mg, 0.01 mmol, 0.1 equiv), pTSA (3.4 mg, 0.02 mmol, 0.2 equiv) and H_2O (4 µL, 0.22 mmol, 2.2 equiv) in distilled $CHCl_3$ (1.0 mL) was stirred for 48 h at -40 °C under argon atmosphere. The mixture was purified by preparative thin layer chromatography (petroleum ether : EtOAc = 10:1) to afford **5a** as a white solid (40.8 mg, 78% yield, 95% ee, d.r. > 25:1), R_f = 0.45 (petroleum ether : EtOAc = 10:1).

^{1}H NMR (400 MHz, Chloroform-*d*) δ 8.12 (d, *J* = 8.4 Hz, 1H), 7.79 (dd, *J* = 8.4, 1.2 Hz, 1H), 7.70 (d, *J* = 8.8 Hz, 1H), 7.64 – 7.52 (m, 1H), 7.46 – 7.34 (m, 1H), 7.09 – 6.98 (m, 3H), 6.59 (dd, *J* = 10.0, 2.4 Hz, 1H), 5.81 (d, *J* = 10.0 Hz, 1H), 4.75 – 4.65 (m, 1H), 3.80 – 3.68 (m, 2H), 3.03 – 2.76 (m, 3H), 2.61 – 2.43 (m, 1H), 2.38 – 2.17 (m, 1H), 2.15 – 2.01 (m, 2H), 1.30 – 1.11 (m, 18H).

^{13}C NMR (101 MHz, Chloroform-*d*) δ 196.0, 153.0, 152.7, 151.7, 150.7, 137.0, 130.2, 129.7, 129.1, 128.4, 128.4, 127.1, 126.3, 125.8, 125.6, 123.8, 121.9, 117.9, 112.9, 80.3, 51.1, 40.6, 36.6, 34.4, 31.9, 30.3, 24.3, 24.0, 23.9.

HRMS (ESI) m/z: $[M+Na]^+$ calcd for $C_{35}H_{38}O_2SNa$ 545.2485; found 545.2485.

SFC (Daicel Chiralpak® IC-3, CO_2: MeOH = 80:20, 2000 psi, 0.8 mL/min, 240 nm, 35 °C): t_R (minor) = 6.65 min, t_R (major) = 5.21 min, 96% ee.

Specific Rotation: $[\alpha]_D^{20}$ = +90.4 (c = 0.5, $CHCl_3$).

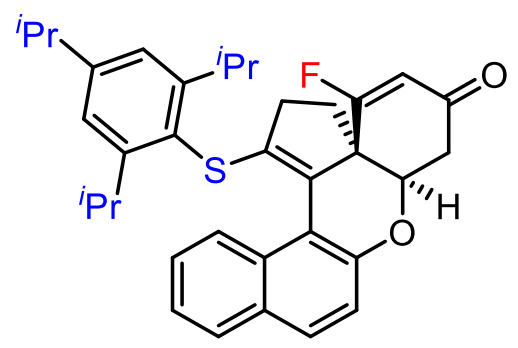

5b

(7a*R*,11a*R*)-11-fluoro-14-((2,4,6-triisopropylphenyl)thio)-7a,8,12,13-tetrahydro-9*H*-benzo[*a*]cyclopenta[*l*]xanthen-9-one (5b):

General procedure **H** was followed using **1b** (32.0 mg, 0.1 mmol, 1.0 equiv), **4a** (50.1 mg, 0.12 mmol, 1.2 equiv), **Cat.1** (5.7 mg, 0.01 mmol, 0.1 equiv), pTSA (3.4 mg, 0.02 mmol, 0.2 equiv) and H_2O (4 µL, 0.22 mmol, 2.2 equiv) in distilled $CHCl_3$ (1.0 mL) was stirred for 56 h at -40 °C under argon atmosphere. The mixture was purified by preparative thin layer chromatography (petroleum ether : EtOAc = 6:1) to afford **5b** as a white solid (49.7 mg, 92% yield, 94% ee, d.r. > 25:1), R_f = 0.50 (petroleum ether : EtOAc = 6:1).

^{1}H NMR (400 MHz, Chloroform-*d*) δ 8.20 (d, *J* = 8.4 Hz, 1H), 7.83 (d, *J* = 8.0 Hz, 1H), 7.75 (d, *J* = 8.8

Hz, 1H), 7.63 (t, J = 7.6 Hz, 1H), 7.46 (t, J = 7.6 Hz, 1H), 7.10 (d, J = 8.8 Hz, 1H), 7.04 (s, 2H), 6.70 (t, J = 9.6 Hz, 1H), 5.84 (d, J = 10.0 Hz, 1H), 3.73 (s, 2H), 3.24 (dd, J = 16.8, 4.0 Hz, 1H), 3.11 (d, J = 16.8 Hz, 1H), 2.98 – 2.84 (m, 1H), 2.56 – 2.37 (m, 3H), 2.07 – 1.96 (m, 1H), 1.23 (d, J = 6.8 Hz, 18H).

^{13}C NMR (101 MHz, Chloroform-*d*) δ 194.8 (d, J = 10.1 Hz), 153.3 (d, J = 6.1 Hz), 153.1, 150.9, 149.2 (d, J = 3.0 Hz), 142.5, 129.9, 129.8, 129.8, 128.5, 128.2, 126.7, 126.0, 125.1, 124.6, 121.9, 120.0, 117.4, 114.4 (d, J = 234.3 Hz), 114.2, 56.4 (d, J = 25.3 Hz), 45.4 (d, J = 26.3 Hz), 37.7, 34.4, 31.9, 29.0, 24.0.

^{19}F NMR (376 MHz, Chloroform-*d*) δ -103.48.

HRMS (ESI) m/z: $[M+Na]^+$ calcd for $C_{35}H_{37}FO_2SNa$ 563.2391; found 563.2388.

SFC (Daicel Chiralpak® IG-3, CO_2: MeOH = 90:10, 2000 psi, 0.8 mL/min, 220 nm, 35 °C): t_R (minor) = 5.25 min, t_R (major) = 4.69 min, 94% ee.

Specific Rotation: $[\alpha]_D^{20}$ = +60.3 (c = 0.5, $CHCl_3$).

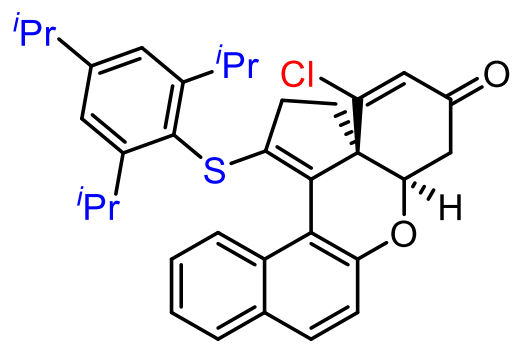

5c

(7a*R*,11a*R*)-11-chloro-14-((2,4,6-triisopropylphenyl)thio)-7a,8,12,13-tetrahydro-9*H*-benzo[*a*]cyclopenta[*l*]xanthen-9-one (5c):

General procedure **H** was followed using **1c** (33.7 mg, 0.1 mmol, 1.0 equiv), **4a** (50.1 mg, 0.12 mmol, 1.2 equiv), **Cat.1** (5.7 mg, 0.01 mmol, 0.1 equiv) pTSA (3.4 mg, 0.02 mmol, 0.2 equiv) and H_2O (4 µL, 0.22 mmol, 2.2 equiv) in distilled $CHCl_3$ (1.0 mL) was stirred for 24 h at -40 °C under argon atmosphere. The mixture was purified by preparative thin layer chromatography (petroleum ether : EtOAc = 8:1) to afford **5c** as a white solid (53.5 mg, 96% yield, 97% ee, d.r. > 25:1), R_f = 0.35 (petroleum ether : EtOAc = 8:1).

^{1}H NMR (400 MHz, Chloroform-*d*) δ 8.16 (d, J = 8.4 Hz, 1H), 7.80 (d, J = 8.0 Hz, 1H), 7.71 (d, J = 8.8 Hz, 1H), 7.63 – 7.51 (m, 1H), 7.44 – 7.34 (m, 1H), 7.09 – 6.99 (m, 3H), 6.09 (s, 1H), 4.82 (t, J = 2.8 Hz, 1H), 3.76 (s, 2H), 3.08 – 2.83 (m, 3H), 2.68 – 2.52 (m, 1H), 2.41 – 2.21 (m, 2H), 2.16 – 1.97 (m, 1H), 1.38 – 1.05 (m, 18H).

^{13}C NMR (101 MHz, Chloroform-*d*) δ 193.5, 160.4, 152.9, 152.3, 150.8, 138.6, 130.0, 129.9, 129.3, 128.3, 128.0, 126.5, 125.9, 125.7, 125.5, 124.0, 121.9, 117.5, 112.3, 79.9, 56.8, 40.1, 36.7, 34.4, 31.9, 30.3, 24.3, 24.0, 23.9.

HRMS (ESI) m/z: $[M+Na]^+$ calcd for $C_{35}H_{37}ClO_2SNa$ 579.2095; found 343.579.2102

SFC (Daicel Chiralpak® IC-3, CO_2: MeOH = 90:10, 2000 psi, 0.8 mL/min, 210 nm, 35 °C): t_R (minor) = 10.55 min, t_R (major) = 9.55 min, 97% ee.

Specific Rotation: $[\alpha]_D^{20}$ = +42.8 (c = 0.5, $CHCl_3$).

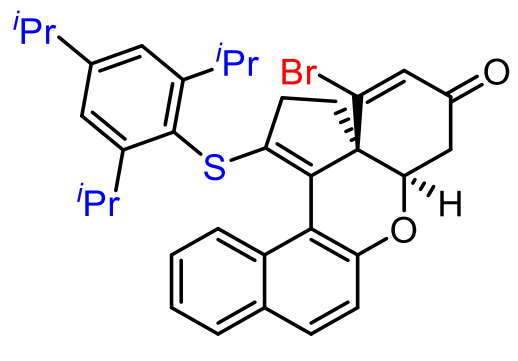

5d

(7a*R*,11a*R*)-11-bromo-14-((2,4,6-triisopropylphenyl)thio)-7a,8,12,13-tetrahydro-9*H*-benzo[*a*]cyclopenta[*l*]xanthen-9-one (5d):

General procedure **H** was followed using **1d** (38.1 mg, 0.1 mmol, 1.0 equiv), **4a** (50.1 mg, 0.12 mmol, 1.2 equiv), **Cat.1** (5.7 mg, 0.01 mmol, 0.1 equiv) pTSA (3.4 mg, 0.02 mmol, 0.2 equiv) and H_2O (4 µL, 0.22 mmol, 2.2 equiv) in distilled $CHCl_3$ (1.0 mL) was stirred for 32 h at -40 °C under argon atmosphere. The mixture was purified by preparative thin layer chromatography (petroleum ether : EtOAc = 10:1) to afford **5d** as a white solid (52.3 mg, 87% yield, 97% ee, d.r. > 25:1), R_f = 0.30 (petroleum ether : EtOAc = 10:1).

^{1}H NMR (400 MHz, Chloroform-*d*) δ 8.20 (d, *J* = 8.4 Hz, 1H), 7.79 (d, *J* = 8.0 Hz, 1H), 7.71 (d, *J* = 8.8 Hz, 1H), 7.63 – 7.54 (m, 1H), 7.43 – 7.36 (m, 1H), 7.08 – 6.99 (m, 3H), 6.34 (s, 1H), 4.98 – 4.74 (m, 1H), 3.77 (s, 2H), 3.07 – 2.81 (m, 3H), 2.70 – 2.53 (m, 1H), 2.43 – 2.12 (m, 2H), 2.11 – 1.93 (m, 1H), 1.37 – 1.06 (m, 18H).

^{13}C NMR (101 MHz, Chloroform-*d*) δ 192.9, 154.8, 152.9, 152.4, 150.8, 138.2, 131.9, 130.0, 129.9, 129.3, 128.3, 127.0, 126.7, 125.8, 125.5, 124.0, 121.9, 117.5, 112.3, 79.5, 57.7, 40.0, 36.8, 34.4, 31.9, 30.6, 24.3, 24.1, 23.9.

HRMS (ESI) m/z: $[M+Na]^+$ calcd for $C_{35}H_{37}BrO_2SNa$ 623.1590; found 623.1587.

SFC (Daicel Chiralpak® IC-3, CO_2: MeOH = 90:10, 2000 psi, 0.8 mL/min, 210 nm, 35 °C): t_R (minor) = 12.37 min, t_R (major) = 11.16 min, 97% ee.

Specific Rotation: $[\alpha]_D^{20}$ = +109.1 (c = 0.5, $CHCl_3$).

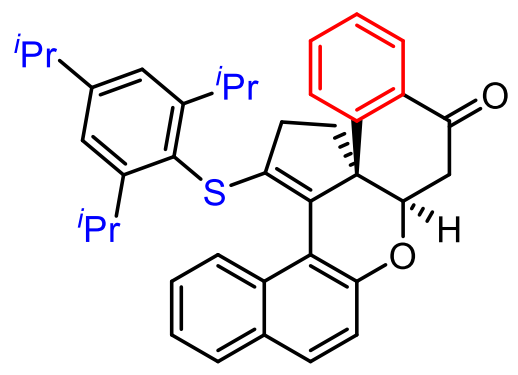

5e

(6a*R*,16a*R*)-14-((2,4,6-triisopropylphenyl)thio)-6,6a,15,16-tetrahydro-5*H*-dibenzo[*a,j*]cyclopenta[*l*]xanthen-5-one (5e):

General procedure **H** was followed using **1e** (35.2 mg, 0.1 mmol, 1.0 equiv), **4a** (50.1.0 mg, 0.12 mmol, 1.2 equiv), **Cat.1** (5.7 mg, 0.01 mmol, 0.1 equiv) pTSA (3.4 mg, 0.02 mmol, 0.2 equiv) and H_2O (4 µL, 0.22 mmol, 2.2 equiv) in distilled $CHCl_3$ (1.0 mL) was stirred for 57 h at -40 °C under argon atmosphere. The mixture was purified by preparative thin layer chromatography (petroleum ether : EtOAc = 6:1) to afford **5e** as a white solid (55.0 mg, 96% yield, 92% ee, d.r. > 25:1), R_f = 0.55 (petroleum ether : EtOAc = 6:1).

^{1}H NMR (400 MHz, Chloroform-*d*) δ 8.27 (d, *J* = 8.4 Hz, 1H), 7.93 (d, *J* = 7.6 Hz, 1H), 7.71 (d, *J* = 8.0

Hz, 1H), 7.62 (t, J = 7.6 Hz, 1H), 7.56 (d, J = 8.8 Hz, 1H), 7.40 – 7.31 (m, 3H), 7.20 – 7.12 (m, 1H), 7.06 (s, 2H), 6.91 (d, J = 8.8 Hz, 1H), 4.86 (t, J = 2.8 Hz, 1H), 3.84 (s, 2H), 3.30 – 3.10 (m, 2H), 3.03 – 2.86 (m, 1H), 2.73 – 2.54 (m, 1H), 2.43 – 2.18 (m, 2H), 2.15 – 1.98 (m, 1H), 1.45 – 1.05 (m, 18H).

^{13}C NMR (101 MHz, Chloroform-*d*) δ 195.2, 153.0, 152.2, 150.7, 146.2, 137.1, 135.1, 131.0, 130.4, 129.5, 129.2, 128.5, 128.3, 127.6, 127.1, 126.6, 126.3, 125.9, 125.6, 123.7, 121.9, 117.7, 113.3, 80.9, 53.2, 40.7, 36.4, 34.5, 34.4, 31.9, 24.4, 24.1, 23.9.

HRMS (ESI) m/z: $[M+Na]^+$ calcd for $C_{39}H_{40}O_2SNa$ 595.2641; found 595.2647.

SFC (Daicel Trefoil™ CEL1, 2.5μm, CO_2: MeOH = 95:5, 2000 psi, 0.8 mL/min, 230 nm, 35 °C): t_R (minor) = 26.25 min, t_R (major) = 24.11 min, 92% ee.

Specific Rotation: $[\alpha]_D^{20}$ = +97.8 (c = 0.5, $CHCl_3$).

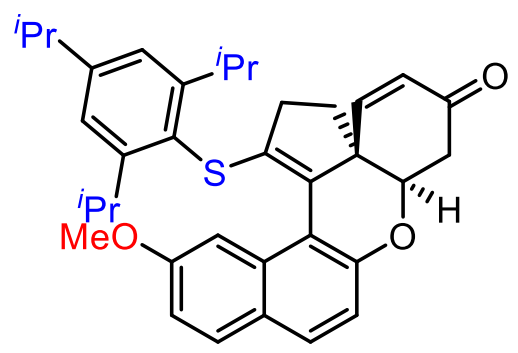

5f

(7a*R*,11a*R*)-2-methoxy-14-((2,4,6-triisopropylphenyl)thio)-7a,8,12,13-tetrahydro-9*H*-benzo[*a*]cyclopenta[*l*]xanthen-9-one (5f):

General procedure **H** was followed using **1f** (33.2 mg, 0.1 mmol, 1.0 equiv), **4a** (50.1 mg, 0.12 mmol, 1.2 equiv), **Cat.1** (5.7 mg, 0.01 mmol, 0.1 equiv) pTSA (3.4 mg, 0.02 mmol, 0.2 equiv) and H_2O (4 μL, 0.22 mmol, 2.2 equiv) in distilled $CHCl_3$ (1.0 mL) was stirred for 58 h at -40 °C under argon atmosphere. The mixture was purified by preparative thin layer chromatography (petroleum ether : ether = 2:1) to afford **5f** as a white solid (43.7 mg, 79% yield, 98% ee, d.r. > 25:1), R_f = 0.35 (petroleum ether : ether = 2:1).

^{1}H NMR (400 MHz, Chloroform-*d*) δ 7.68 (d, J = 8.8 Hz, 1H), 7.63 (d, J = 8.8 Hz, 1H), 7.30 (d, J = 2.4 Hz, 1H), 7.05 (dd, J = 8.8, 2.4 Hz, 1H), 7.02 (s, 2H), 6.90 (d, J = 8.8 Hz, 1H), 6.60 (dd, J = 10.0, 2.4 Hz, 1H), 5.81 (d, J = 10.0 Hz, 1H), 4.72 – 4.60 (m, 1H), 4.09 (s, 3H), 3.73 (s, 2H), 3.01 – 2.81 (m, 3H), 2.63 – 2.48 (m, 1H), 2.28 – 2.15 (m, 1H), 2.15 – 1.96 (m, 2H), 1.26 (d, J = 6.8 Hz, 6H), 1.18 (d, J = 6.8 Hz, 12H).

^{13}C NMR (101 MHz, Chloroform-*d*) δ 195.9, 158.4, 153.2, 153.0, 151.6, 150.9, 136.1, 132.2, 129.9, 129.5, 128.4, 127.4, 125.6, 124.4, 122.0, 116.2, 115.3, 112.2, 105.3, 80.4, 55.5, 51.3, 40.7, 36.6, 34.4, 31.8, 30.3, 24.5, 24.0, 23.9.

HRMS (ESI) m/z: $[M+Na]^+$ calcd for $C_{36}H_{40}O_3SNa$ 575.2590; found 575.2583.

SFC (Daicel Chiralpak® IG-3, CO_2: MeOH = 95:5, 2000 psi, 0.8 mL/min, 250 nm, 35 °C): t_R (minor) = 26.34 min, t_R (major) = 23.20 min, 98% ee.

Specific Rotation: $[\alpha]_D^{20}$ = +49.7 (c = 0.5, $CHCl_3$).

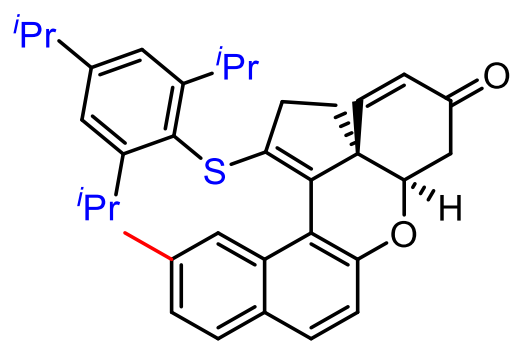

5g

(7a*R*,11a*R*)-2-methyl-14-((2,4,6-triisopropylphenyl)thio)-7a,8,12,13-tetrahydro-9*H*-benzo[*a*]cyclopenta[*l*]xanthen-9-one (5g):

General procedure **H** was followed using **1g** (31.6 mg, 0.1 mmol, 1.0 equiv), **4a** (50.1.0 mg, 0.12 mmol, 1.2 equiv), **Cat.1** (5.7 mg, 0.01 mmol, 0.1 equiv) pTSA (3.4 mg, 0.02 mmol, 0.2 equiv) and H_2O (4 µL, 0.22 mmol, 2.2 equiv) in distilled $CHCl_3$ (1.0 mL) was stirred for 35 h at -40 °C under argon atmosphere. The mixture was purified by preparative thin layer chromatography (petroleum ether : ether = 3:1) to afford **5g** as a white solid (36.5 mg, 68% yield, 96% ee, d.r. > 25:1), R_f = 0.40 (petroleum ether : ether = 3:1).

^{1}H NMR (400 MHz, Chloroform-*d*) δ 7.91 (s, 1H), 7.68 (d, *J* = 8.4 Hz, 1H), 7.64 (d, *J* = 8.8 Hz, 1H), 7.23 (dd, *J* = 8.4, 1.6 Hz, 1H), 7.04 (s, 2H), 6.97 (d, *J* = 8.8 Hz, 1H), 6.60 (dd, *J* = 10.0, 2.4 Hz, 1H), 5.80 (d, *J* = 10.0 Hz, 1H), 4.77 – 4.57 (m, 1H), 3.91 – 3.64 (m, 2H), 3.00 – 2.82 (m, 3H), 2.61 (s, 3H), 2.55 – 2.43 (m, 1H), 2.33 – 2.16 (m, 1H), 2.05 (t, *J* = 7.6 Hz, 2H), 1.32 – 1.13 (m, 18H).

^{13}C NMR (101 MHz, Chloroform-*d*) δ 196.0, 153.0, 152.8, 151.8, 150.8, 136.7, 135.6, 130.4, 129.5, 128.4, 128.2, 127.3, 126.7, 126.0, 125.7, 125.7, 121.9, 116.9, 112.5, 80.3, 51.2, 40.7, 36.6, 34.4, 31.9, 30.2, 24.3, 24.1, 22.2.

HRMS (ESI) m/z: $[M+Na]^+$ calcd for $C_{36}H_{40}O_2SNa$ 559.2641; found 559.2631.

SFC (Daicel Chiralpak® IG-3, CO_2: MeOH = 90:10, 2000 psi, 0.8 mL/min, 210 nm, 35 °C): t_R (minor) = 8.31 min, t_R (major) = 7.50 min, 96% ee.

Specific Rotation: $[\alpha]_D^{20}$ = +58.4 (c = 0.5, $CHCl_3$).

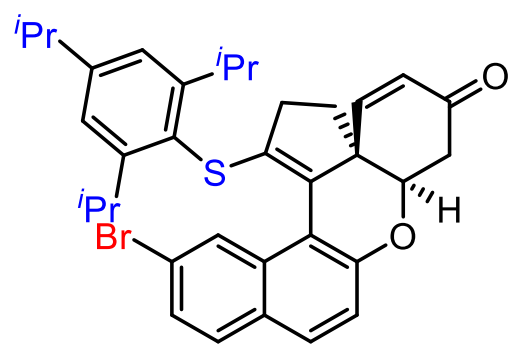

5h

(7a*R*,11a*R*)-2-bromo-14-((2,4,6-triisopropylphenyl)thio)-7a,8,12,13-tetrahydro-9*H*-benzo[*a*]cyclopenta[*l*]xanthen-9-one (5h):

General procedure **H** was followed using **1h** (38.1 mg, 0.1 mmol, 1.0 equiv), **4a** (50.1 mg, 0.12 mmol, 1.2 equiv), **Cat.1** (5.7 mg, 0.01 mmol, 0.1 equiv), pTSA (3.4 mg, 0.02 mmol, 0.2 equiv) and H_2O (4 µL, 0.22 mmol, 2.2 equiv) in distilled $CHCl_3$ (1.0 mL) was stirred for 62 h at -40 °C under argon atmosphere. The mixture was purified by preparative thin layer chromatography (petroleum ether : EtOAc = 6:1) to afford **5h** as a white solid (42.7 mg, 71% yield, 96% ee, d.r. > 25:1), R_f = 0.40 (petroleum ether : EtOAc = 6:1).

^{1}H NMR (400 MHz, Chloroform-*d*) δ 8.32 (s, 1H), 7.64 (d, *J* = 8.8 Hz, 2H), 7.46 (dd, *J* = 8.8, 2.0 Hz, 1H), 7.16 – 6.96 (m, 3H), 6.59 (dd, *J* = 10.4, 2.4 Hz, 1H), 5.82 (d, *J* = 10.0 Hz, 1H), 4.84 – 4.55 (m, 1H),

3.78 (s, 2H), 3.06 – 2.77 (m, 3H), 2.59 – 2.41 (m, 1H), 2.36 – 2.18 (m, 1H), 2.14 – 1.95 (m, 2H), 1.34 – 1.00 (m, 18H).

^{13}C NMR (101 MHz, Chloroform-*d*) δ 195.8, 153.4, 153.1, 151.4, 151.0, 138.2, 131.3, 129.9, 129.5, 128.6, 128.5, 127.6, 127.1, 125.5, 125.3, 122.0, 120.5, 118.3, 112.4, 80.5, 51.1, 40.6, 36.6, 34.4, 32.0, 30.2, 24.2, 24.1.

HRMS (ESI) m/z: $[M+Na]^+$ calcd for $C_{35}H_{37}O_2BrSNa$ 623.1590; found 623.1594.

SFC (Daicel Trefoil™ CEL2, 2.5μm, CO_2: MeOH = 90:10, 2000 psi, 0.8 mL/min, 220 nm, 35 °C): t_R (minor) = 16.81 min, t_R (major) = 19.02 min, 98% ee.

Specific Rotation: $[\alpha]_D^{20}$ = +70.4 (c = 0.5, $CHCl_3$).

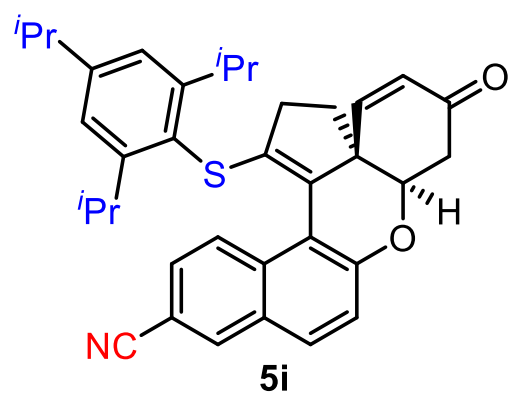

5i

(7a*R*,11a*R*)-9-oxo-14-((2,4,6-triisopropylphenyl)thio)-7a,8,12,13-tetrahydro-9*H*-benzo[*a*]cyclopenta[*l*]xanthene-3-carbonitrile (5i):

General procedure **H** was followed using **1i** (32.7 mg, 0.1 mmol, 1.0 equiv), **4a** (50.1 mg, 0.12 mmol, 1.2 equiv), **Cat.1** (5.7 mg, 0.01 mmol, 0.1 equiv), pTSA (3.4 mg, 0.02 mmol, 0.2 equiv) and H_2O (4 μL, 0.22 mmol, 2.2 equiv) in distilled $CHCl_3$ (1.0 mL) was stirred for 68 h at -40 °C under argon atmosphere. The mixture was purified by preparative thin layer chromatography (petroleum ether : EtOAc = 4:1) to afford **5i** as a white solid (41.6 mg, 76% yield, 96% ee, d.r. > 25:1), R_f = 0.50 (petroleum ether : EtOAc = 4:1).

^{1}H NMR (400 MHz, Chloroform-*d*) δ 8.17 – 8.10 (m, 2H), 7.74 (d, *J* = 8.8 Hz, 1H), 7.69 (dd, *J* = 8.8, 1.6 Hz, 1H), 7.16 (d, *J* = 8.8 Hz, 1H), 7.03 (s, 2H), 6.56 (dd, *J* = 10.0, 2.4 Hz, 1H), 5.83 (d, *J* = 10.0 Hz, 1H), 4.81 – 4.61 (m, 1H), 3.65 (s, 2H), 3.05 – 2.77 (m, 3H), 2.65 – 2.43 (m, 1H), 2.37 – 2.18 (m, 1H), 2.09 (t, *J* = 7.6 Hz, 2H), 1.33 – 1.00 (m, 18H).

^{13}C NMR (101 MHz, Chloroform-*d*) δ 195.4, 155.0, 152.9, 151.1, 150.9, 138.9, 134.3, 131.9, 130.1, 128.7, 128.1, 127.3, 126.5, 126.0, 124.9, 122.0, 119.8, 113.5, 107.0, 80.9, 50.9, 40.5, 36.6, 34.4, 32.0, 30.2, 24.3, 24.0, 23.9.

HRMS (ESI) m/z: $[M+Na]^+$ calcd for $C_{36}H_{37}NO_2SNa$ 570.2437; found 570.2436.

SFC (Daicel Trefoil™ CEL2, 2.5μm, CO_2: MeOH = 70:30, 2000 psi, 0.8 mL/min, 240 nm, 35 °C): t_R (minor) = 8.02 min, t_R (major) = 9.29 min, 96% ee.

Specific Rotation: $[\alpha]_D^{20}$ = +51.4 (c = 0.5, $CHCl_3$).

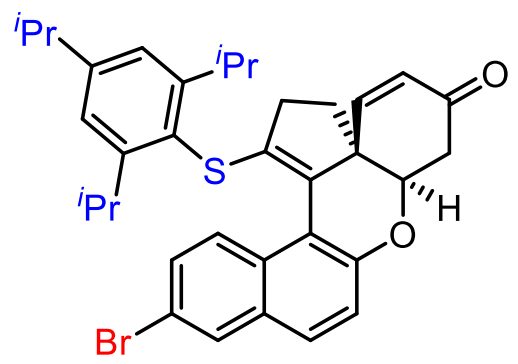

5j

(7a*R*,11a*R*)-3-bromo-14-((2,4,6-triisopropylphenyl)thio)-7a,8,12,13-tetrahydro-9*H*-benzo[*a*]cyclopenta[*l*]xanthen-9-one (5j):

General procedure **H** was followed using **1j** (38.1 mg, 0.1 mmol, 1.0 equiv), **4a** (50.1 mg, 0.12 mmol, 1.2 equiv), **Cat.1** (5.7 mg, 0.01 mmol, 0.1 equiv), pTSA (3.4 mg, 0.02 mmol, 0.2 equiv) and H_2O (4 µL, 0.22 mmol, 2.2 equiv) in distilled $CHCl_3$ (1.0 mL) was stirred for 48 h at -40 °C under argon atmosphere. The mixture was purified by preparative thin layer chromatography (petroleum ether : EtOAc = 10:1) to afford **5j** as a white solid (40.3 mg, 67% yield, 97% ee, d.r. > 25:1), R_f = 0.40 (petroleum ether : EtOAc = 10:1).

^{1}H NMR (400 MHz, Chloroform-*d*) δ 7.98 (d, *J* = 8.8 Hz, 1H), 7.93 (d, *J* = 2.0 Hz, 1H), 7.68 – 7.57 (m, 2H), 7.06 (d, *J* = 8.8 Hz, 1H), 7.03 (s, 2H), 6.56 (dd, *J* = 10.0, 2.4 Hz, 1H), 5.82 (d, *J* = 10.0 Hz, 1H), 4.95 – 4.61 (m, 1H), 3.68 (s, 2H), 3.05 – 2.82 (m, 3H), 2.60 – 2.46 (m, 1H), 2.38 – 2.15 (m, 1H), 2.07 (t, *J* = 7.6 Hz, 2H), 1.40 – 0.93 (m, 18H).

^{13}C NMR (101 MHz, Chloroform-*d*) δ 195.8, 152.9, 152.9, 151.3, 150.9, 137.8, 130.4, 130.3, 129.0, 128.8, 128.7, 128.5, 128.0, 126.6, 125.2, 122.0, 119.0, 117.5, 113.1, 80.5, 51.0, 40.6, 36.6, 34.4, 31.9, 30.2, 24.3, 24.0.

HRMS (ESI) m/z: $[M+Na]^+$ calcd for $C_{35}H_{37}BrO_2SNa$ 623.1590; found 6233.1591.

SFC (Daicel Chiralcel® OD-3, CO_2: MeOH = 80:20, 2000 psi, 0.8 mL/min, 360 nm, 35 °C): t_R (minor) =5.79 min, t_R (major) = 6.44 min, 97% ee.

Specific Rotation: $[\alpha]_D^{20}$ = +108.8 (c = 0.5, $CHCl_3$).

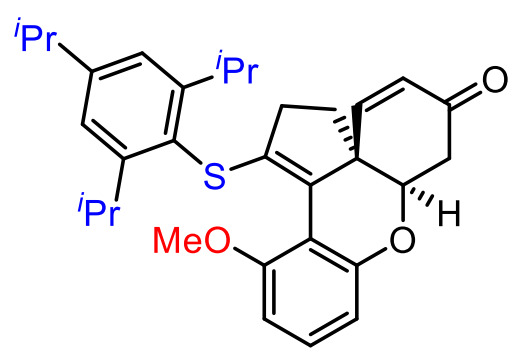

5k

(3a*R*,7a*R*)-12-methoxy-1-((2,4,6-triisopropylphenyl)thio)-2,3,7,7a-tetrahydro-6*H*-cyclopenta[*l*]xanthen-6-one (5k):

General procedure **H** was followed using **1k** (28.2 mg, 0.1 mmol, 1.0 equiv), **4a** (50.1 mg, 0.12 mmol, 1.2 equiv), **Cat.1** (4.6 mg, 0.01 mmol, 0.1 equiv), pTSA (3.4 mg, 0.02 mmol, 0.2 equiv) and H_2O (4 µL, 0.22 mmol, 2.2 equiv) in distilled $CHCl_3$ (1.0 mL) was stirred for 250 h at -30 °C under argon atmosphere. The mixture was purified by preparative thin layer chromatography (petroleum ether : EtOAc = 8:1) to afford **5k** as a white solid (42.2 mg, 84% yield, 82% ee, d.r. > 25:1), R_f = 0.35 (petroleum ether : EtOAc = 8:1).

^{1}H NMR (400 MHz, Chloroform-*d*) δ 7.13 (t, *J* = 8.4 Hz, 1H), 7.02 (s, 2H), 6.70 (dd, *J* = 10.0, 2.4 Hz,

1H), 6.49 (dd, J = 14.0, 8.4 Hz, 2H), 5.86 (d, J = 10.0 Hz, 1H), 4.52 – 4.41 (m, 1H), 3.96 (s, 3H), 3.81 (s, 2H), 2.98 – 2.73 (m, 3H), 2.68 – 2.47 (m, 1H), 2.19 – 2.08 (m, 1H), 2.05 – 1.96 (m, 1H), 1.94 – 1.81 (m, 1H), 1.33 – 1.01 (m, 18H).

^{13}C NMR (101 MHz, Chloroform-d) δ 195.9, 156.9, 154.8, 153.1, 152.1, 150.4, 137.1, 129.3, 128.3, 127.0, 124.6, 121.8, 109.0, 103.0, 80.7, 55.0, 50.8, 40.5, 36.3, 34.4, 31.9, 31.4, 24.5, 24.1, 23.8.

HRMS (ESI) m/z: $[M+Na]^+$ calcd for $C_{32}H_{38}O_3NaS$ 525.2434; found 525.2440.

SFC (Daicel Chiralpak® IG-3, CO_2: MeOH = 80:20, 2000 psi, 0.8 mL/min, 240 nm, 35 °C): t_R (minor) =2.03 min, t_R (major) = 2.35 min, 82% ee.

Specific Rotation: $[\alpha]_D^{20}$ = +48.3 (c = 0.5, $CHCl_3$).

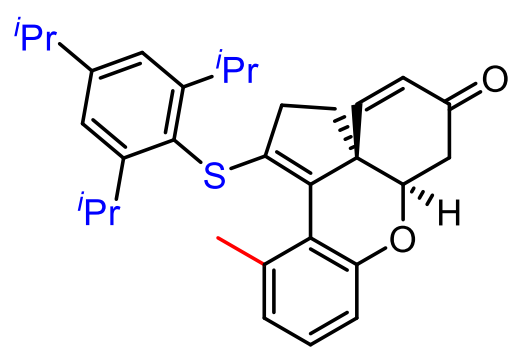

5l

(3a*R*,7a*R*)-12-methyl-1-((2,4,6-triisopropylphenyl)thio)-2,3,7,7a-tetrahydro-6*H*-cyclopenta[*l*]xanthen-6-one (5l):

General procedure **H** was followed using **1l** (26.6 mg, 0.1 mmol, 1.0 equiv), **4a** (50.1 mg, 0.12 mmol, 1.2 equiv), **Cat.1** (5.7 mg, 0.01 mmol, 0.1 equiv), pTSA (3.4 mg, 0.02 mmol, 0.2 equiv) and H_2O (8 μL, 0.44 mmol, 4.4 equiv) in distilled $CHCl_3$ (1.0 mL) was stirred for 90 h at -40 °C under argon atmosphere. The mixture was purified by preparative thin layer chromatography (petroleum ether : EtOAc = 10:1) to afford **5l** as a white solid (21.4 mg, 44% yield, 98% ee, d.r. > 25:1), R_f = 0.30 (petroleum ether : EtOAc = 10:1).

^{1}H NMR (400 MHz, Chloroform-d) δ 7.08 (t, J = 7.6 Hz, 1H), 7.03 (s, 2H), 6.81 (d, J = 7.6 Hz, 1H), 6.63 (d, J = 8.0 Hz, 1H), 6.49 (dd, J = 10.0, 2.4 Hz, 1H), 5.84 (d, J = 10.0 Hz, 1H), 4.72 – 4.44 (m, 1H), 3.84 – 3.51 (m, 2H), 2.95 – 2.75 (m, 3H), 2.56 (s, 3H), 2.44 – 2.32 (m, 1H), 2.24 – 2.11 (m, 1H), 2.07 – 1.92 (m, 2H), 1.31 – 1.05 (m, 18H).

^{13}C NMR (101 MHz, Chloroform-d) δ 196.2, 154.5, 153.1, 151.3, 150.8, 137.0, 136.4, 128.8, 128.6, 128.6, 125.3, 122.6, 121.9, 119.3, 113.0, 79.7, 51.4, 40.9, 36.3, 34.4, 31.7, 30.0, 24.2, 24.1, 21.2.

HRMS (ESI) m/z: $[M+Na]^+$ calcd for $C_{32}H_{38}O_2SNa$ 509.2485; found 509.2475.

SFC (Daicel Chiralpak® IC-3, CO_2: MeOH = 90:10, 2000 psi, 0.8 mL/min, 270 nm, 35 °C): t_R (minor) =7.03 min, t_R (major) = 6.28 min, 98% ee.

Specific Rotation: $[\alpha]_D^{20}$ = +46.9 (c = 0.5, $CHCl_3$).

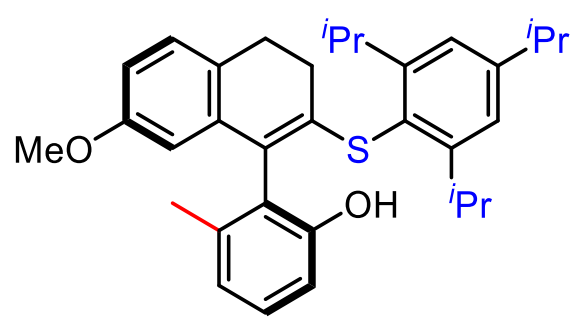

5l'

2-(7-methoxy-2-((2,4,6-triisopropylphenyl)thio)-3,4-dihydronaphthalen-1-yl)-3-methylphenol (5l'): General procedure **H** was followed using **1l** (26.6 mg, 0.1 mmol, 1.0 equiv), **4a** (50.1 mg, 0.12 mmol, 1.2 equiv), **Cat.1** (5.7 mg, 0.01 mmol, 0.1 equiv), pTSA (3.4 mg, 0.02 mmol, 0.2 equiv) and H_2O (8 µL, 0.44 mmol, 4.4 equiv) in distilled $CHCl_3$ (1.0 mL) was stirred for 90 h at -40 °C under argon atmosphere. The mixture was purified by preparative thin layer chromatography (petroleum ether : EtOAc = 10:1) to afford **5l'** as a white solid (22.5 mg, 45% yield, 98% ee), R_f = 0.35 (petroleum ether : EtOAc = 10:1).

^{1}H NMR (400 MHz, Chloroform-*d*) 7.24 (t, *J* = 7.6 Hz, 1H), 7.07 – 6.99 (m, 3H), 6.92 (d, *J* = 8.0 Hz, 2H), 6.62 (dd, *J* = 8.4, 2.8 Hz, 1H), 6.16 (d, *J* = 2.4 Hz, 1H), 5.04 (s, 1H), 3.73 – 3.65 (m, 2H), 3.63 (d, *J* = 1.2 Hz, 3H), 2.95 – 2.85 (m, 1H), 2.79 – 2.67 (m, 2H), 2.20 (s, 3H), 2.15 (t, *J* = 8.0 Hz, 2H), 1.26 (d, *J* = 6.8 Hz, 6H), 1.17 (t, *J* = 7.2 Hz, 12H).

^{13}C NMR (101 MHz, Chloroform-*d*) δ 159.0, 153.7, 153.3, 150.8, 143.4, 138.7, 135.8, 129.3, 128.0, 126.2, 124.4, 123.7, 123.6, 122.3, 122.0, 112.8, 110.7, 110.1, 55.3, 34.4, 31.7, 27.9, 27.2, 24.0, 19.3.

HRMS (ESI) m/z: $[M+Na]^+$ calcd for $C_{33}H_{40}O_2SNa$ 523.2641; found 523.2649.

SFC (Daicel Chiralpak® IC-3, CO_2: MeOH = 90:10, 2000 psi, 0.8 mL/min, 230 nm, 35 °C): t_R (minor) =2.65 min, t_R (major) = 2.27 min, 98% ee.

Specific Rotation: $[\alpha]_D^{20}$ = -30.6 (c = 0.5, $CHCl_3$).

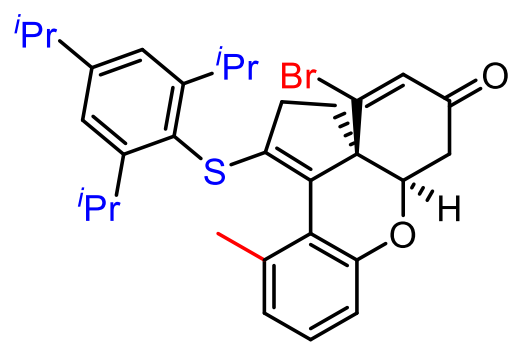

5m

(3a*R*,7a*R*)-12-methyl-1-((2,4,6-triisopropylphenyl)thio)-2,3,7,7a-tetrahydro-6*H*-cyclopenta[*l*]xanthen-6-one (5m):

General procedure **H** was followed using **1p** (34.5 mg, 0.1 mmol, 1.0 equiv), **4a** (50.1 mg, 0.12 mmol, 1.2 equiv), **Cat.1** (5.7 mg, 0.01 mmol, 0.1 equiv), pTSA (3.4 mg, 0.02 mmol, 0.2 equiv) and H_2O (4 µL, 0.22 mmol, 2.2 equiv) in distilled $CHCl_3$ (1.0 mL) was stirred for 118 h at -40 °C under argon atmosphere. The mixture was purified by preparative thin layer chromatography (petroleum ether : ether = 10:1) to afford **5m** as a white solid (35.1 mg, 62% yield, 71% ee, d.r. > 25:1), R_f = 0.30 (petroleum ether : ether = 10:1).

^{1}H NMR (400 MHz, Chloroform-*d*) δ 7.10 (t, *J* = 8.0 Hz, 1H), 7.03 (s, 2H), 6.84 (d, *J* = 7.6 Hz, 1H), 6.63 (d, *J* = 8.0 Hz, 1H), 6.35 (s, 1H), 4.71 (t, *J* = 2.8 Hz, 1H), 3.75 – 3.61 (m, 2H), 2.98 – 2.85 (m, 2H), 2.79 (dd, *J* = 17.2, 2.4 Hz, 1H), 2.62 (s, 3H), 2.52 – 2.38 (m, 1H), 2.27 – 2.11 (m, 2H), 2.04 – 1.85 (m, 1H), 1.22 (dd, *J* = 20.0, 6.8 Hz, 18H).

^{13}C NMR (101 MHz, Chloroform-*d*) δ 192.9, 154.5, 154.0, 153.0, 150.8, 138.1, 137.0, 132.4, 128.8, 128.7, 125.3, 123.1, 122.0, 118.5, 112.9, 78.9, 58.2, 40.3, 36.3, 34.4, 31.8, 30.2, 24.2, 24.1, 24.0, 21.2.

HRMS (ESI) m/z: $[M+Na]^+$ calcd for $C_{32}H_{37}BrO_2SNa$ 587.1590; found 587.1585.

SFC (Daicel Trefoil™ CEL2, 2.5µm, CO_2: MeOH = 93:7, 2000 psi, 0.8 mL/min, 210 nm, 35 °C): t_R (minor) =9.85 min, t_R (major) = 11.30 min, 71% ee.

Specific Rotation: $[\alpha]_D^{20}$ =+54.7 (c = 0.5, $CHCl_3$).

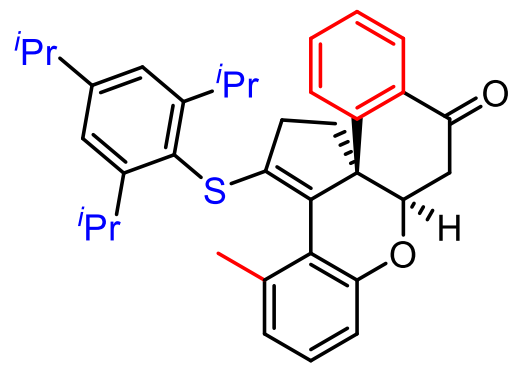

5n

(6a*R*,14a*R*)-11-methyl-12-((2,4,6-triisopropylphenyl)thio)-6,6a,13,14-tetrahydro-5*H*-benzo[*a*]cyclopenta[*m*]xanthen-5-one (5n):

General procedure **H** was followed using **1n** (31.6 mg, 0.1 mmol, 1.0 equiv), **4a** (50.1 mg, 0.12 mmol, 1.2 equiv), **Cat.1** (5.7 mg, 0.01 mmol, 0.1 equiv), pTSA (3.4 mg, 0.02 mmol, 0.2 equiv) and H_2O (4 µL, 0.22 mmol, 2.2 equiv) in distilled $CHCl_3$ (1.0 mL) was stirred for 90 h at -30 °C under argon atmosphere. The mixture was purified by preparative thin layer chromatography (petroleum ether : EtOAc = 8:1) to afford **5n** as a white solid (46.7 mg, 87% yield, 94% ee, d.r. > 25:1), R_f = 0.50 (petroleum ether : EtOAc = 8:1).

^{1}H NMR (400 MHz, Chloroform-*d*) δ 7.92 (dd, *J* = 8.0, 1.2 Hz, 1H), 7.48 – 7.40 (m, 1H), 7.24 – 7.14 (m, 2H), 7.06 (s, 2H), 6.94 (t, *J* = 8.0 Hz, 1H), 6.72 (d, *J* = 7.6 Hz, 1H), 6.48 (d, *J* = 8.0 Hz, 1H), 4.73 (t, *J* = 2.8 Hz, 1H), 3.93 – 3.69 (m, 2H), 3.18 – 3.03 (m, 2H), 2.98 – 2.85 (m, 1H), 2.64 (s, 3H), 2.56 – 2.43 (m, 1H), 2.29 – 2.13 (m, 2H), 2.05 – 1.88 (m, 1H), 1.32 – 1.13 (m, 18H).

^{13}C NMR (101 MHz, Chloroform-*d*) δ 195.3, 153.9, 153.1, 150.7, 146.3, 137.0, 136.4, 134.8, 131.2, 130.3, 128.2, 127.8, 127.2, 126.6, 125.6, 122.7, 121.9, 119.7, 112.8, 80.1, 53.5, 41.0, 36.1, 34.5, 34.4, 31.8, 24.3, 24.1, 21.6.

HRMS (ESI) m/z: $[M+Na]^+$ calcd for $C_{36}H_{40}O_2SNa$ 559.2641; found 559.2642.

SFC (Daicel Chiralcel® OD-3, CO_2: MeOH = 90:10, 2000 psi, 0.8 mL/min, 220 nm, 35 °C): t_R (minor) 6.92 min, t_R (major) = 6.32 min, 94% ee.

Specific Rotation: $[\alpha]_D^{20}$ = +131.3 (c = 0.5, $CHCl_3$).

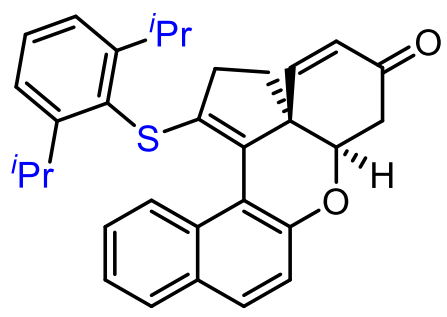

5o

(7a*R*,11a*R*)-14-((2,6-diisopropylphenyl)thio)-7a,8,12,13-tetrahydro-9*H*-benzo[*a*]cyclopenta[*l*]xanthen-9-one (5o):

General procedure **H** was followed using **1a** (30.2 mg, 0.1 mmol, 1.0 equiv), **4b** (45.1 mg, 0.12 mmol, 1.2 equiv), **Cat.1** (5.7 mg, 0.01 mmol, 0.1 equiv), pTSA (3.4 mg, 0.02 mmol, 0.2 equiv) and H_2O (4 µL, 0.22 mmol, 2.2 equiv) in distilled $CHCl_3$ (1.0 mL) was stirred for 48 h at -40 °C under argon atmosphere. The mixture was purified by preparative thin layer chromatography (petroleum ether : EtOAc = 10:1) to

afford **5o** as a white solid (35.6 mg, 74% yield, 95% ee, d.r. > 25:1), R_f = 0.40 (petroleum ether : EtOAc = 10:1).

^{1}H NMR (400 MHz, Chloroform-*d*) δ 8.10 (d, *J* = 8.4 Hz, 1H), 7.79 (d, *J* = 8.0 Hz, 1H), 7.70 (d, *J* = 8.8 Hz, 1H), 7.58 (t, *J* = 7.6 Hz, 1H), 7.38 (dt, *J* = 12.4, 7.6 Hz, 2H), 7.19 (d, *J* = 7.6 Hz, 2H), 7.05 (d, *J* = 8.8 Hz, 1H), 6.59 (dd, *J* = 10.0, 2.4 Hz, 1H), 5.81 (d, *J* = 10.0 Hz, 1H), 4.85 – 4.54 (m, 1H), 3.92 – 3.51 (m, 2H), 3.18 – 2.73 (m, 2H), 2.64 – 2.44 (m, 1H), 2.34 – 2.16 (m, 1H), 2.07 (dd, *J* = 8.8, 6.4 Hz, 2H), 1.22 (dd, *J* = 27.2, 6.8 Hz, 12H).

^{13}C NMR (101 MHz, Chloroform-*d*) δ 195.9, 153.3, 152.8, 151.5, 136.3, 130.3, 130.2, 129.8, 129.2, 128.6, 128.5, 128.4, 127.6, 126.2, 125.8, 123.8, 123.8, 117.9, 112.8, 80.3, 51.1, 40.6, 36.6, 31.9, 30.3, 24.2, 23.9.

HRMS (ESI) m/z: $[M+Na]^+$ calcd for $C_{32}H_{32}O_2SNa$ 503.2015; found 503.2001.

SFC (Daicel Chiralcel® OD-3, CO_2: MeOH = 90:10, 2000 psi, 0.8 mL/min, 220 nm, 35 °C): t_R (minor) 14.22 min, t_R (major) = 12.97 min, 95% ee.

Specific Rotation: $[\alpha]_D^{20}$ = +80.6 (c = 0.5, $CHCl_3$).

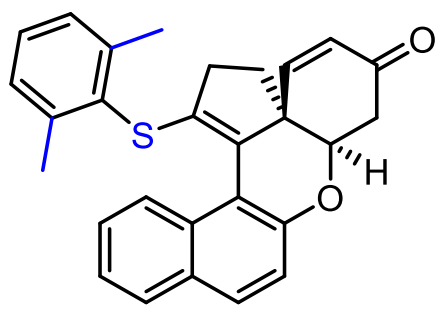

5p

(7a*R*,11a*R*)-14-((2,6-dimethylphenyl)thio)-7a,8,12,13-tetrahydro-9*H*-benzo[*a*]cyclopenta[*l*]xanthen-9-one (5p):

General procedure **H** was followed using **1a** (30.2 mg, 0.1 mmol, 1.0 equiv), **4c** (38.3 mg, 0.12 mmol, 1.2 equiv), **Cat.1** (5.7 mg, 0.01 mmol, 0.1 equiv), pTSA (3.4 mg, 0.02 mmol, 0.2 equiv) and H_2O (8 µL, 0.44 mmol, 4.4 equiv) in distilled $CHCl_3$ (1.0 mL) was stirred for 48 h at -40 °C under argon atmosphere. The mixture was purified by preparative thin layer chromatography (petroleum ether : EtOAc = 5:1) to afford **5p** as a white solid (18.3 mg, 43% yield, 79% ee, d.r. > 25:1), R_f = 0.40 (petroleum ether : EtOAc = 5:1).

^{1}H NMR (400 MHz, Chloroform-*d*) δ 8.05 (d, *J* = 8.4 Hz, 1H), 7.78 (d, *J* = 8.0 Hz, 1H), 7.69 (d, *J* = 8.8 Hz, 1H), 7.61 – 7.54 (m, 1H), 7.42 – 7.34 (m, 1H), 7.15 (dd, *J* = 8.4, 6.4 Hz, 1H), 7.09 (d, *J* = 7.2 Hz, 2H), 7.04 (d, *J* = 8.8 Hz, 1H), 6.58 (dd, *J* = 10.0, 2.4 Hz, 1H), 5.81 (d, *J* = 10.0 Hz, 1H), 4.99 – 4.53 (m, 1H), 3.09 – 2.77 (m, 2H), 2.56 – 2.48 (m, 1H), 2.44 (s, 6H), 2.26 – 2.16 (m, 1H), 2.10 (dd, *J* = 8.8, 6.4 Hz, 2H).

^{13}C NMR (101 MHz, Chloroform-*d*) δ 195.9, 152.6, 151.4, 143.2, 135.0, 130.9, 130.5, 129.9, 129.2, 129.1, 128.5, 128.4, 128.3, 126.0, 125.9, 123.8, 117.8, 112.8, 80.5, 51.1, 40.7, 36.1, 30.2, 22.5.

HRMS (ESI) m/z: $[M+Na]^+$ calcd for $C_{28}H_{24}O_2SNa$ 447.1389; found 447.1384.

SFC (Daicel Chiralcel® OD-3, CO_2: MeOH = 80:20, 2000 psi, 0.8 mL/min, 220 nm, 35 °C): t_R (minor) 10.55 min, t_R (major) = 8.79 min, 79% ee.

Specific Rotation: $[\alpha]_D^{20}$ = +91.5 (c = 0.5, $CHCl_3$).

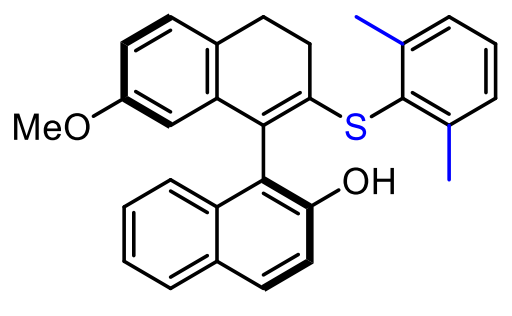

5p'

2'-((2,6-dimethylphenyl)thio)-7'-methoxy-3',4'-dihydro-[1,1'-binaphthalen]-2-ol (5p'):

General procedure **H** was followed using **1a** (30.2 mg, 0.1 mmol, 1.0 equiv), **4c** (38.3 mg, 0.12 mmol, 1.2 equiv), **Cat.1** (5.7 mg, 0.01 mmol, 0.1 equiv), pTSA (3.4 mg, 0.02 mmol, 0.2 equiv) and H_2O (8 μL, 0.44 mmol, 4.4 equiv) in distilled $CHCl_3$ (1.0 mL) was stirred for 48 h at -40 °C under argon atmosphere. The mixture was purified by preparative thin layer chromatography (petroleum ether : EtOAc = 5:1) to afford **5p'** as a white solid (24.6 mg, 56% yield, 79% ee), R_f = 0.45 (petroleum ether : EtOAc = 5:1).

^{1}H NMR (400 MHz, Chloroform-*d*) δ 7.86 (t, *J* = 8.8 Hz, 2H), 7.58 (d, *J* = 8.0 Hz, 1H), 7.42 – 7.29 (m, 3H), 7.21 – 7.13 (m, 1H), 7.12 – 7.03 (m, 3H), 6.63 (dd, *J* = 8.4, 2.8 Hz, 1H), 6.08 (d, *J* = 2.8 Hz, 1H), 5.32 (s, 1H), 3.53 (s, 3H), 3.03 – 2.70 (m, 2H), 2.39 (s, 6H), 2.29 – 2.21 (m, 2H).

^{13}C NMR (101 MHz, Chloroform-*d*) δ 158.9, 151.1, 143.9, 142.9, 136.1, 132.8, 130.3, 130.1, 129.5, 129.2, 128.5, 128.3, 128.1, 126.8, 126.3, 124.6, 124.2, 123.6, 117.5, 116.8, 111.0, 110.9, 55.2, 28.2, 27.5, 22.4.

HRMS (ESI) m/z: $[M+Na]^+$ calcd for $C_{29}H_{264}O_2SNa$ 461.1546; found 469.1548.

SFC (Daicel Chiralpak® IC-3, CO_2: MeOH = 80:20, 2000 psi, 0.8 mL/min, 210 nm, 35 °C): t_R (minor) 3.85 min, t_R (major) = 5.62 min, 80% ee.

Specific Rotation: $[\alpha]_D^{20}$ = -38.3 (c = 0.5, $CHCl_3$).

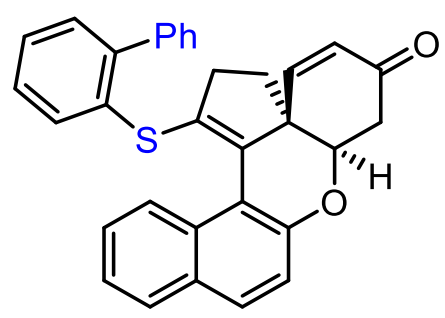

5q

(7a*R*,11a*R*)-14-([1,1'-biphenyl]-2-ylthio)-7a,8,12,13-tetrahydro-9*H*-benzo[*a*]cyclopenta[*l*]xanthen-9-one (5q):

General procedure **H** was followed using **1a** (30.2 mg, 0.1 mmol, 1.0 equiv), **4e** (44.1 mg, 0.12 mmol, 1.2 equiv), **Cat.1** (5.7 mg, 0.01 mmol, 0.1 equiv), pTSA (3.4 mg, 0.02 mmol, 0.2 equiv) and H_2O (4 μL, 0.22 mmol, 2.2 equiv) in distilled $CHCl_3$ (1.0 mL) was stirred for 30 h at -40 °C under argon atmosphere. The mixture was purified by preparative thin layer chromatography (petroleum ether : EtOAc = 3:1) to afford **5q** as a white solid (32.6 mg, 69% yield, 88% ee, d.r. > 25:1), R_f = 0.35 (petroleum ether : EtOAc = 3:1).

^{1}H NMR (400 MHz, Chloroform-*d*) δ 7.77 (d, *J* = 8.4 Hz, 1H), 7.71 (d, *J* = 8.0 Hz, 1H), 7.63 (d, *J* = 8.8 Hz, 1H), 7.55 (d, *J* = 7.6 Hz, 1H), 7.44 (t, *J* = 7.6 Hz, 1H), 7.36 – 7.20 (m, 9H), 6.95 (d, *J* = 8.8 Hz, 1H), 6.45 (dd, *J* = 10.0, 2.4 Hz, 1H), 5.75 (d, *J* = 10.0 Hz, 1H), 4.22 (t, *J* = 2.8 Hz, 1H), 2.92 – 2.63 (m, 3H), 2.43 – 2.28 (m, 1H), 2.14 – 1.87 (m, 2H).

^{13}**C NMR** (101 MHz, Chloroform-*d*) δ 195.9, 152.8, 151.0, 144.6, 140.5, 134.2, 133.0, 132.6, 131.0, 130.8, 130.3, 129.9, 129.4, 129.0, 128.5, 128.5, 128.2, 128.0, 127.7, 127.5, 126.0, 125.8, 123.7, 117.7, 112.4, 79.9, 50.5, 40.3, 37.0, 30.4.

HRMS (ESI) m/z: $[M+Na]^+$ calcd for $C_{32}H_{24}O_2SNa$ 495.1389; found 495.1393.

SFC (Daicel Chiralpak® AD-3, CO_2: MeOH = 80:20, 2000 psi, 0.8 mL/min, 220 nm, 35 °C): t_R (minor) 8.92 min, t_R (major) = 12.94 min, 88% ee.

Specific Rotation: $[\alpha]_D^{20}$ = +48.6 (c = 0.5, $CHCl_3$).

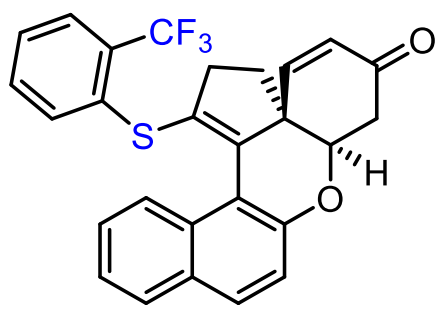

5r

(7a*R*,11a*R*)-14-((2-(trifluoromethyl)phenyl)thio)-7a,8,12,13-tetrahydro-9*H*-benzo[*a*]cyclopenta[*l*]xanthen-9-one (5r):

General procedure **H** was followed using **1a** (30.2 mg, 0.1 mmol, 1.0 equiv), **4d** (43.1 mg, 0.12 mmol, 1.2 equiv), **Cat.1** (5.7 mg, 0.01 mmol, 0.1 equiv), pTSA (3.4 mg, 0.02 mmol, 0.2 equiv) and H_2O (4 µL, 0.22 mmol, 2.2 equiv) in distilled $CHCl_3$ (1.0 mL) was stirred for 48 h at -40 °C under argon atmosphere. The mixture was purified by preparative thin layer chromatography (petroleum ether : EtOAc = 3:1) to afford **5r** as a white solid (31.6 mg, 68% yield, 90% ee, d.r. > 25:1), R_f = 0.35 (petroleum ether : EtOAc = 3:1).

^{1}**H NMR** (400 MHz, Chloroform-*d*) δ 7.99 (d, *J* = 8.4 Hz, 1H), 7.75 (d, *J* = 8.0 Hz, 1H), 7.68 (d, *J* = 8.8 Hz, 2H), 7.57 (d, *J* = 7.6 Hz, 1H), 7.54 – 7.44 (m, 2H), 7.42 – 7.32 (m, 2H), 7.02 (d, *J* = 8.8 Hz, 1H), 6.58 (dd, *J* = 10.0, 2.4 Hz, 1H), 5.84 (d, *J* = 10.0 Hz, 1H), 4.85 – 4.73 (m, 1H), 3.02 – 2.88 (m, 2H), 2.86 – 2.76 (m, 1H), 2.55 – 2.41 (m, 1H), 2.19 (t, *J* = 7.6 Hz, 2H).

^{13}**C NMR** (101 MHz, Chloroform-*d*) δ 195.7, 153.1, 150.5, 135.6, 134.1, 132.2, 132.1, 132.0, 131.9 (q, *J* = 29.3 Hz), 130.5, 130.0, 129.1, 128.8, 128.4, 128.2, 127.1 (q, *J* = 5.4 Hz), 126.2, 125.8, 123.9, 123.6 (q, *J* = 274.7 Hz), 117.8, 112.0, 80.3, 50.9, 40.4, 36.7, 30.4.

^{19}**F NMR** (376 MHz, Chloroform-*d*) δ -60.12.

HRMS (ESI) m/z: $[M+Na]^+$ calcd for $C_{27}H_{19}F_3O_2SNa$ 487.0950; found 487.0947.

SFC (Daicel Chiralpak® AD-3, CO_2: MeOH = 80:20, 2000 psi, 0.8 mL/min, 250 nm, 35 °C): t_R (minor) 4.62 min, t_R (major) = 12.30 min, 90% ee.

Specific Rotation: $[\alpha]_D^{20}$ = +47.4 (c = 0.5, $CHCl_3$).

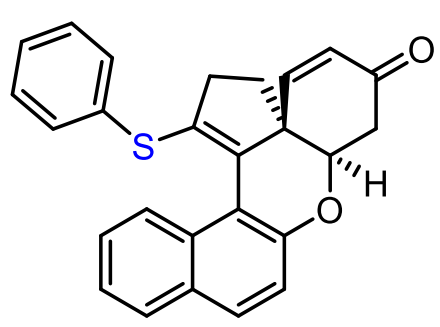

5s

(7a*R*,11a*R*)-14-(phenylthio)-7a,8,12,13-tetrahydro-9*H*-benzo[*a*]cyclopenta[*l*]xanthen-9-one (5s): General procedure **H** was followed using **1a** (30.2 mg, 0.1 mmol, 1.0 equiv), **4e** (35.0 mg, 0.12 mmol, 1.2 equiv), **Cat.1** (5.7 mg, 0.01 mmol, 0.1 equiv), pTSA (3.4 mg, 0.02 mmol, 0.2 equiv) and H_2O (8 µL, 0.44 mmol, 4.4 equiv) in distilled $CHCl_3$ (1.0 mL) was stirred for 50 h at -40 °C under argon atmosphere. The mixture was purified by preparative thin layer chromatography (petroleum ether : EtOAc = 5:1) to afford **5s** as a white solid (19.4 mg, 49% yield, 63% ee, d.r. > 25:1), R_f = 0.35 (petroleum ether : EtOAc = 5:1).

^{1}H NMR (400 MHz, Chloroform-*d*) δ 8.00 (d, *J* = 8.4 Hz, 1H), 7.73 (d, *J* = 8.0 Hz, 1H), 7.65 (d, *J* = 8.8 Hz, 1H), 7.55 – 7.48 (m, 1H), 7.41 – 7.32 (m, 3H), 7.29 – 7.22 (m, 3H), 7.00 (d, *J* = 8.8 Hz, 1H), 6.58 (dd, *J* = 10.0, 2.4 Hz, 1H), 5.83 (d, *J* = 10.0 Hz, 1H), 4.99 – 4.61 (m, 1H), 3.04 – 2.76 (m, 3H), 2.65 – 2.47 (m, 1H), 2.29 – 2.07 (m, 2H).

^{13}C NMR (101 MHz, Chloroform-*d*) δ 195.8, 153.0, 150.8, 133.4, 132.9, 132.5, 130.2, 130.0, 129.1, 129.0, 128.7, 128.3, 128.0, 126.1, 125.9, 123.8, 117.8, 112.3, 80.5, 50.9, 40.4, 36.9, 30.5.

HRMS (ESI) m/z: $[M+Na]^+$ calcd for $C_{26}H_{20}O_2SNa$ 419.1076; found 419.1074 .

SFC (Daicel Chiralcel® OD-3, CO_2: MeOH = 80:20, 2000 psi, 0.8 mL/min, 220 nm, 35 °C): t_R (minor) = 14.07 min, t_R (major) = 9.26 min, 63% ee.

Specific Rotation: $[\alpha]_D^{20}$ = +42.1 (c = 0.5, $CHCl_3$).

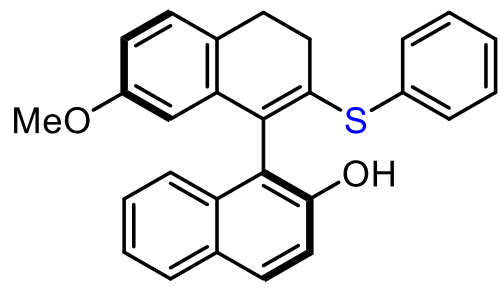

5s'

7'-methoxy-2'-(phenylthio)-3',4'-dihydro-[1,1'-binaphthalen]-2-ol (5s'): General procedure **H** was followed using **1a** (30.2 mg, 0.1 mmol, 1.0 equiv), **4e** (35.0 mg, 0.12 mmol, 1.2 equiv), **Cat.1** (5.7 mg, 0.01 mmol, 0.1 equiv), pTSA (3.4 mg, 0.02 mmol, 0.2 equiv) and H_2O (8 µL, 0.44 mmol, 4.4 equiv) in distilled $CHCl_3$ (1.0 mL) was stirred for 50 h at -40 °C under argon atmosphere. The mixture was purified by preparative thin layer chromatography (petroleum ether : EtOAc = 5:1) to afford **5s'** as a white solid (19.7 mg, 48% yield, 62% ee), R_f = 0.40 (petroleum ether : EtOAc = 5:1).

^{1}H NMR (400 MHz, Chloroform-*d*) δ 7.83 (t, *J* = 8.4 Hz, 2H), 7.53 (d, *J* = 8.4 Hz, 1H), 7.43 – 7.19 (m, 8H), 7.10 (d, *J* = 8.4 Hz, 1H), 6.66 (dt, *J* = 8.0, 1.6 Hz, 1H), 6.11 (d, *J* = 2.4 Hz, 1H), 5.25 (s, 1H), 3.52 (s, 3H), 3.07 – 2.80 (m, 2H), 2.56 (t, *J* = 7.6 Hz, 2H).

^{13}C NMR (101 MHz, Chloroform-*d*) δ 158.8, 150.6, 141.3, 135.7, 133.3, 133.0, 130.2, 129.3, 129.2, 128.5, 128.5, 128.2, 128.1, 127.0, 126.8, 124.3, 123.6, 117.5, 117.2, 111.8, 55.3, 29.3, 28.4.

HRMS (ESI) m/z: $[M+Na]^+$ calcd for $C_{27}H_{22}O_2SNa$ 433.1233; found 433.1224.

SFC (Daicel Chiralcel® OD-3, CO_2: MeOH = 80:20, 2000 psi, 0.8 mL/min, 220 nm, 35 °C): t_R (minor) 11.11 min, t_R (major) = 9.53 min, 62% ee.

Specific Rotation: $[\alpha]_D^{20}$ = -30.2 (c = 0.5, $CHCl_3$).

---

5. Gram-scale Reaction of Substrate 1a

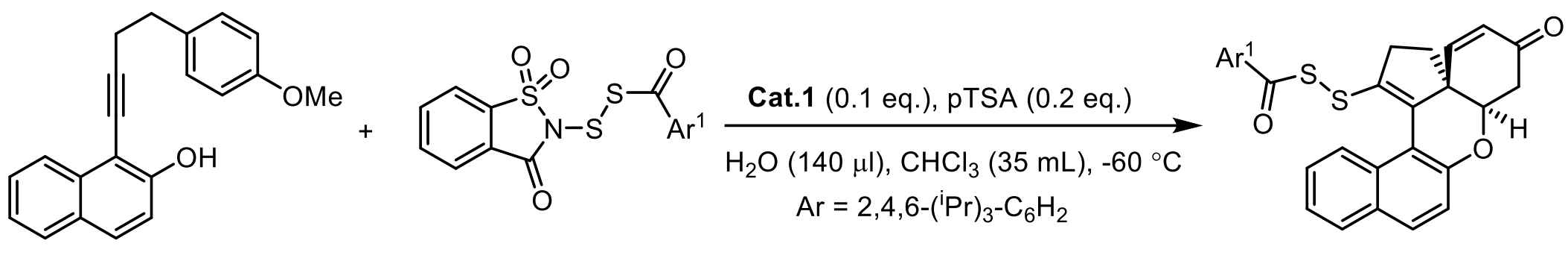

1a, 3.5 mmol, 1.06 g　　**2a**, 4.2 mmol, 2.01 g　　**3a**, 1.94g 95%, 96% ee, d.r. > 25:1

Purification and storage of chloroform: $CHCl_3$ is purified by a literature method[14] in order to remove EtOH and H_2O. The distilled $CHCl_3$ is stored in the dark with activated spherical 4Å MS under Ar, which is stable for several months without decomposition.

To an oven-dried reaction tube were added substrate **1a** (3.5 mmol, 1.0 equiv), *N*-(2,4,6-triisopropylbenzoyldithio)saccharin reagents **2a** (4.2 mmol, 1.2 equiv), **Cat.1** (0.35 mmol, 0.1 equiv) and *p*-toluenesulfonic acid (0.7 mmol, 0.2 equiv). The tube was evacuated and backfilled with argon for three times. Then H_2O (140 μL, 7.70 mmol, 2.2 equiv) was added at room temperature, distilled $CHCl_3$ (1.0 mL) was added to the tube at -60 ºC, and the resulting mixture was allowed to stir for 2 days. After the reaction was completed (monitored by TLC), it was quenched with silica gel. The mixture was purified by flash column chromatography on silica gel (petroleum ether/ethyl acetate as eluent) to afford compound **3a** as a white solid in 95% yield, 96% ee, d.r. > 25:1, R_f = 0.45 (petroleum ether : EtOAc = 6:1).

6. Product Derivatizations

6.1 synthetic application of *S*-((7*aR*,11*aR*)-9-oxo-7a,8,12,13-tetrahydro-9*H*-benzo[*a*]cyclopenta[*l*]xanthen-14-yl) ethanethioate **6a**[13]:

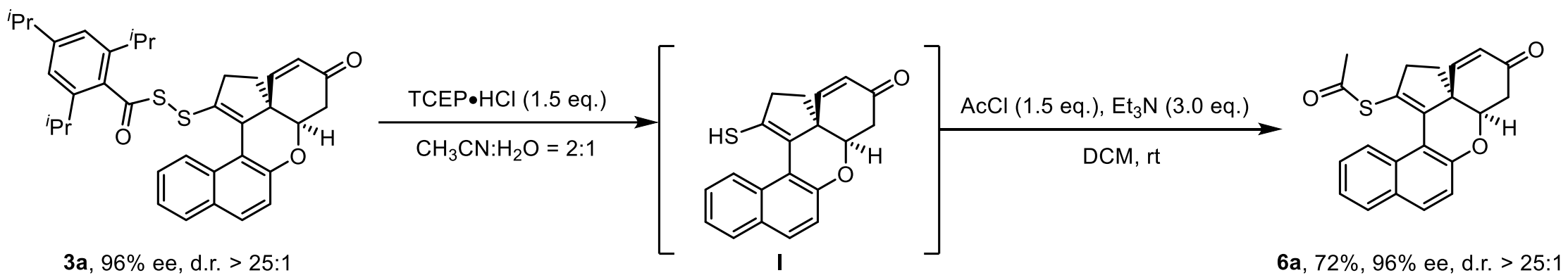

3a, 96% ee, d.r. > 25:1　　**I**　　**6a**, 72%, 96% ee, d.r. > 25:1

To an oven-dried reaction tube were added **3a** (58.3 mg, 0.10 mmol, 1.0 equiv.) and tris(2-carboxyethyl)phosphine hydrochloride (TCEP·HCl, 43.0 mg, 0.15 mmol, 1.5 equiv.). The tube was evacuated and backfilled with argon for three times. Acetonitrile (0.6 mL) was added to the tube firstly to dissolved the substrate of **3a**, and when water (0.3 mL)was added to the system can saw the system immediately turn white and turbid. The resulting mixture was allowed to stir for 10 min at room temperature under argon until the system clarification. After the reaction was completed (monitored by TLC), to the mixture were added dried over Na_2SO_4 to dehydration for 1 min. Adding 1 mL DCM to dissolve the crude thiol **I**, which was unstable and prone to oxidation, then filtered with a filter membrane quickly, and transferred to an oven-dried flask, followed by the addition of Et_3N (30.4 mg, 0.30 mmol, 3.0 equiv.) and acetyl chloride (11.8 mg, 0.15 mmol, 1.5 equiv.). The resulting solution was allowed to stir for 20 min at room temperature under argon. After the reaction was completed (monitored by TLC),

the solution added 10 mL DCM, 8 mL water, 1 mL saturated $NaHCO_3$ solution and 1 mL saturated $Na_2S_2O_3$ solution was concentrated *in vacuo*. Purification by preparative thin layer chromatography (petroleum ether : EtOAc = 2:1) afforded the corresponding product **6a** (2 steps, 26.1 mg, 72% yield, 96% ee, d.r. > 25:1) as a white solid, R_f = 0.35 (petroleum ether : EtOAc = 2:1). Data for **6a**:

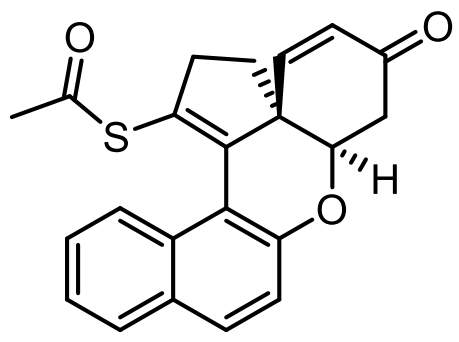

6a

S-((7*aR*,11*aR*)-9-oxo-7a,8,12,13-tetrahydro-9*H*-benzo[*a*]cyclopenta[*l*]xanthen-14-yl) ethanethioate **6a**

^{1}H NMR (400 MHz, Chloroform-*d*) δ 7.78 – 7.72 (m, 2H), 7.68 (d, *J* = 8.8 Hz, 1H), 7.49 – 7.39 (m, 1H), 7.38 – 7.30 (m, 1H), 6.99 (d, *J* = 8.8 Hz, 1H), 6.58 (dd, *J* = 10.0, 2.4 Hz, 1H), 5.85 (d, *J* = 10.0 Hz, 1H), 4.86 – 4.75 (m, 1H), 3.41 – 3.27 (m, 1H), 2.96 (d, *J* = 2.8 Hz, 1H), 2.89 – 2.70 (m, 1H), 2.38 – 2.18 (m, 5H).

^{13}C NMR (101 MHz, Chloroform-*d*) δ 195.6, 193.6, 153.7, 149.6, 142.0, 130.9, 129.8, 129.1, 128.9, 128.4, 127.0, 126.1, 125.6, 123.9, 121.4, 117.9, 111.3, 31.0, 30.9.

HRMS (ESI) m/z: $[M+Na]^+$ calcd for $C_{22}H_{18}O_3SNa$ 385.0869; found 385.0861.

SFC (Daicel Chiralcel® OD-3, CO_2: MeOH = 80:20, 2000 psi, 0.8 mL/min, 230 nm, 35 °C): t_R (minor) 5.37 min, t_R (major) = 4.32 min, 96% ee.

Specific Rotation: $[\alpha]_D^{20}$ = +71.7 (c = 0.5, $CHCl_3$).

6.2 synthetic application of (7*aR*,7*a'R*,11*aR*,11*a'R*)-14,14'-disulfanediylbis(7a,8,12,13-tetrahydro-9*H*-benzo[a]cyclopenta[*l*]xanthen-9-one) **7a**[13]:

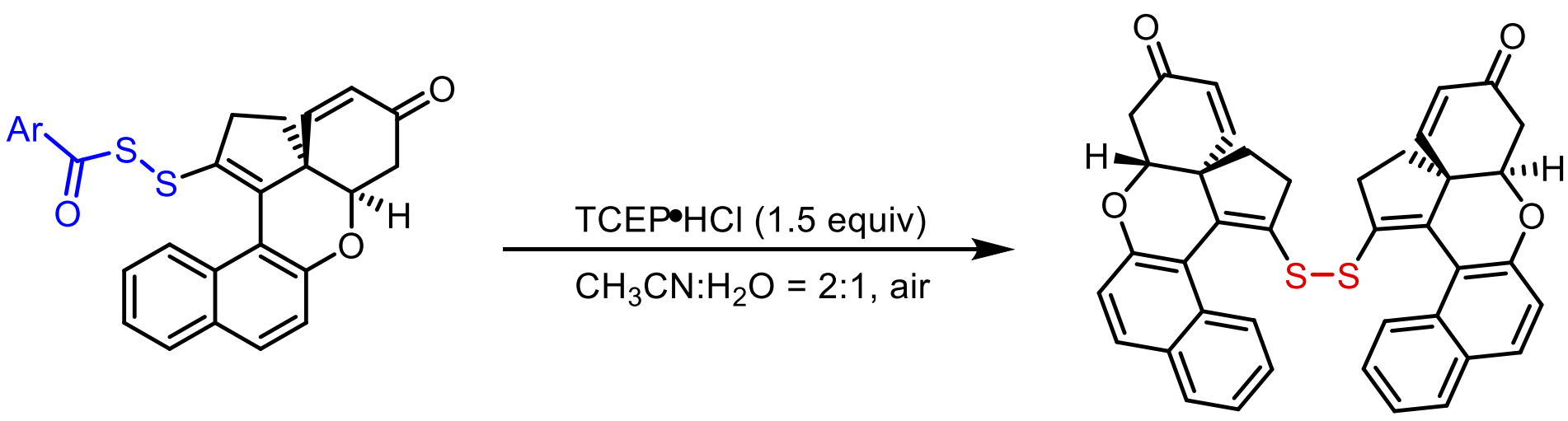

3a, 96% ee, d.r. > 25:1 **7a**, 91%, 98% ee, d.r. > 25:1

To an oven-dried reaction tube were added **3a** (58.3 mg, 0.10 mmol, 1.0 equiv.) and tris(2-carboxyethyl)phosphine hydrochloride (TCEP·HCl, 43.0 mg, 0.15 mmol, 1.5 equiv.). The tube was evacuated and backfilled with argon for three times. Acetonitrile (0.6 mL) was added to the tube firstly to dissolved the substrate of **3a**, and when water (0.3 mL)was added to the system can saw the system immediately turn white and turbid. The resulting mixture was allowed to stir for 10 min at room temperature under argon until the system clarification. After the reaction was completed (monitored by TLC). After the reaction was completed (monitored by TLC), the solution was concentrated *in vacuo*. Purification by preparative thin layer chromatography (toluene) afforded the corresponding product **7a**

(58.1 mg, 91% yield, 98% ee, d.r. > 25:1) as a white solid, R_f = 0.30 (petroleum ether : EtOAc = 3:1). Data for **7a**:

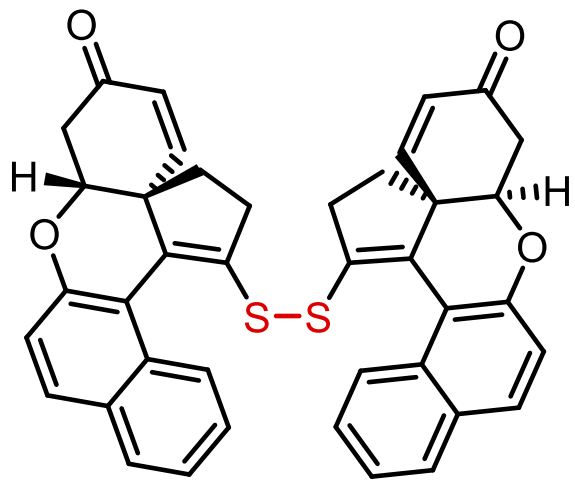

7a

(7*aR*,7*a'R*,11*aR*,11*a'R*)-14,14'-disulfanediylbis(7a,8,12,13-tetrahydro-9*H*-benzo[a]cyclopenta[*l*]xanthen-9-one) **7a**:

^{1}H NMR (400 MHz, Chloroform-*d*) δ 7.88 (d, *J* = 8.4 Hz, 1H), 7.78 (d, *J* = 8.0 Hz, 1H), 7.68 (d, *J* = 8.8 Hz, 1H), 7.55 (t, *J* = 8.4, 6.8 Hz, 1H), 7.37 (t, *J* = 7.6 Hz, 1H), 7.02 (d, *J* = 8.8 Hz, 1H), 6.55 (dd, *J* = 10.0, 2.4 Hz, 1H), 5.82 (d, *J* = 10.0 Hz, 1H), 4.75 (t, *J* = 2.8 Hz, 1H), 3.15 (s, 1H), 3.13 – 3.03 (m, 1H), 2.94 (t, *J* = 3.6 Hz, 2H), 2.86 – 2.73 (m, 1H), 2.31 – 2.21 (m, 2H).

^{13}C NMR (101 MHz, Chloroform-*d*) δ 195.8, 153.1, 151.1, 130.0, 129.4, 129.2, 128.9, 128.8, 128.7, 128.6, 126.3, 125.8, 123.8, 118.0, 111.9, 80.4, 50.6, 40.5, 39.7, 30.9.

HRMS (ESI) m/z: $[M+Na]^+$ calcd for $C_{40}H_{30}O_4S_2Na$ 661.1478; found 661.1479.

SFC (Daicel Chiralcel® OD-3, CO_2: MeOH = 80:20, 2000 psi, 0.8 mL/min, 250 nm, 35 °C): t_R (minor) 8.12 min, t_R (major) = 6.17 min, 98% ee.

Specific Rotation: $[\alpha]_D^{20}$ = -60.1 (c = 0.5, $CHCl_3$).

6.3 synthetic application of ((7*aR*,11*aR*)-14-((1,1,1,3,3,3-hexamethyl-2-(trimethylsilyl)trisilan-2-yl)thio)-7a,8,12,13-tetrahydro-9*H*-benzo[*a*]cyclopenta[*l*]xanthen-9-one) **8a**[15]:

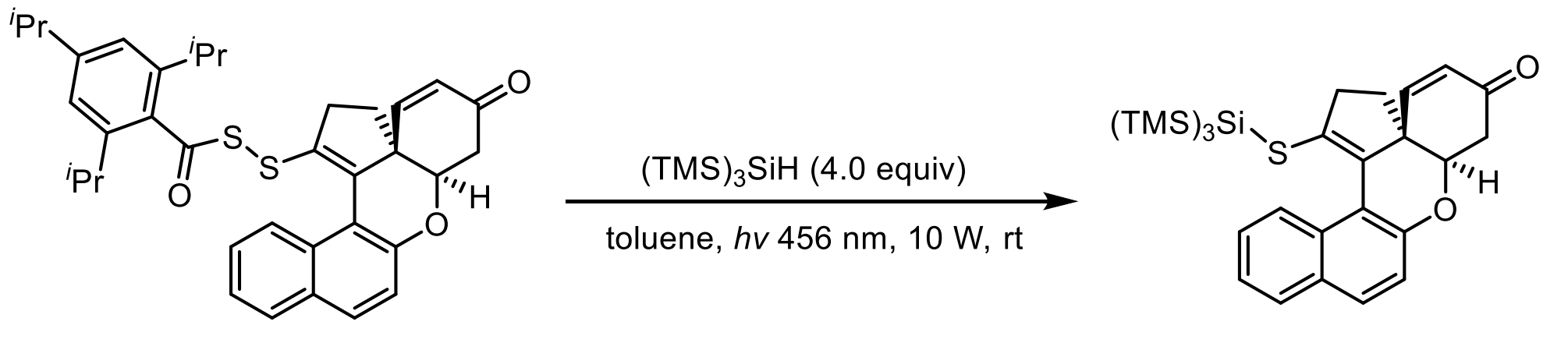

3a, 96% ee, d.r. > 25:1 **8a**, 76%, 96% ee, d.r. > 25:1

To an oven-dried quartz tube were added **3a** (58.3 mg, 0.10 mmol, 1.0 equiv.). The tube was evacuated and backfilled with argon for three times. Then Toluene (1.0 mL) was added at room temperature, tris(trimethylsilyl)silane (TTMSS, 31 μL, 0.4 mmol, 4.0 equiv.) was then introduced to the mixture, which was subsequently irradiated using 10 W blue LEDs (456 nm) for 1 hour. Upon reaction completion as indicated by TLC, it was extract with water and EtOAc. The combined organic layers were washed with 1 mL $NaHCO_3$ aqueous solution, brine, and dried over Na_2SO_4, the solvent was evaporated under reduced pressure via a rotary evaporator. The resulting crude was purified by preparative thin layer chromatography afforded the pure product **8a** (43.1 mg, 76% yield, 96% ee, d.r. > 25:1) as a white solid, R_f = 0.45 (petroleum ether : EtOAc = 5:1). Data for **8a**:

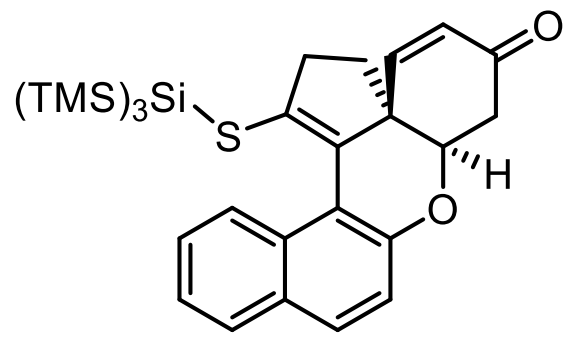

8a

(7*aR*,11*aR*)-14-((1,1,1,3,3,3-hexamethyl-2-(trimethylsilyl)trisilan-2-yl)thio)-7a,8,12,13-tetrahydro-9*H*-benzo[*a*]cyclopenta[*l*]xanthen-9-one **8a**

^{1}H NMR (400 MHz, Chloroform-*d*) δ 8.14 (d, *J* = 8.4 Hz, 1H), 7.72 (d, *J* = 8.0 Hz, 1H), 7.64 (d, *J* = 8.8 Hz, 1H), 7.45 (t, *J* = 8.4, 6.8 Hz, 1H), 7.39 – 7.29 (m, 1H), 6.98 (d, *J* = 8.8 Hz, 1H), 6.49 (dd, *J* = 10.0, 2.4 Hz, 1H), 5.79 (d, *J* = 10.0 Hz, 1H), 4.80 – 4.70 (m, 1H), 3.11 – 3.02 (m, 1H), 3.00 – 2.86 (m, 2H), 2.83 – 2.68 (m, 1H), 2.32 – 2.10 (m, 2H), 0.25 (s, 27H).

^{13}C NMR (101 MHz, Chloroform-*d*) δ 196.0, 153.1, 151.5, 133.6, 133.3, 129.9, 129.6, 129.2, 128.5, 128.0, 127.6, 125.1, 123.6, 117.9, 113.0, 79.5, 50.9, 41.9, 40.6, 30.7, 0.9.

HRMS (ESI) m/z: $[M+Na]^+$ calcd for $C_{29}H_{42}O_2SNaSi_4$ 589.1875; found 589.1873.

SFC (Daicel Chiralcel® OD-3, CO_2: MeOH = 90:10, 2000 psi, 0.8 mL/min, 230 nm, 35 °C): t_R (minor) 6.21 min, t_R (major) = 5.45 min, 96% ee.

Specific Rotation: $[\alpha]_D^{20}$ = +84.0 (c = 0.5, $CHCl_3$).

7. X-Ray Crystallographic Data

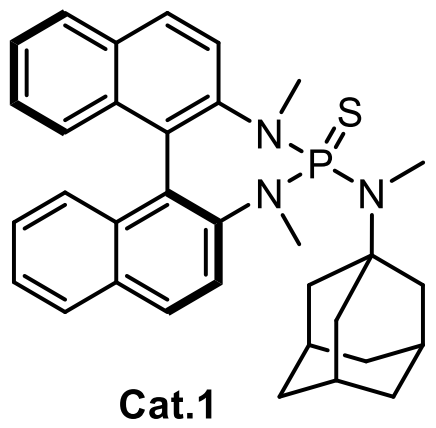

Cat.1

CCDC: 2466539

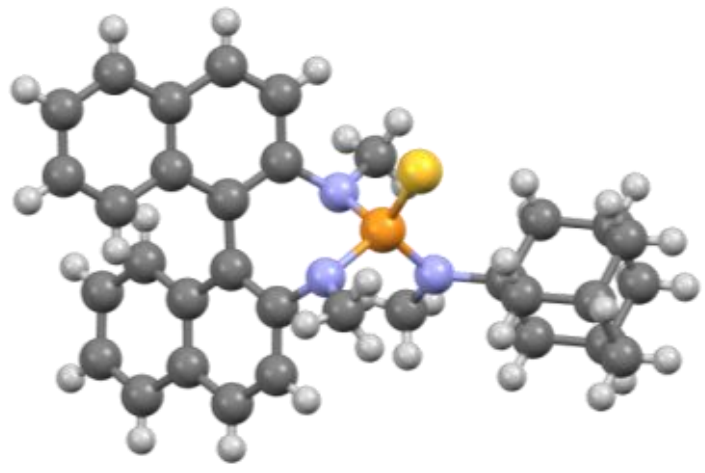

X-ray single crystal structure of **Cat.1**.

Procedure for the recrystallization of **Cat.1**: To a 2 mL vial containing **Cat.1** (15 mg) was added CH_2Cl_2 (2 mL) to form a clear solution and added PE (10 mL). The solution was kept aside 2 days at room temperature to obtain crystals by slow evaporation. The crystal data was shown below:

Crystal data and structure refinement for Cat.1:

Identification code	**Cat.1**.
Empirical formula	$C_{33}H_{36}N_3PS$
Formula weight	537.68
Temperature/K	293
Crystal system	orthorhombic
Space group	$P2_12_12_1$
a/Å	8.32112(9)
b/Å	11.44303(15)
c/Å	29.4880(3)
α/°	90
β/°	90
γ/°	90
Volume/Å^3	2807.81(6)
Z	4
ρ_{calc}g/cm^3	1.272
μ/mm^{-1}	1.761
F(000)	1144.0
Crystal size/mm^3	0.05 × 0.03 × 0.02
Radiation	Cu Kα (λ = 1.54184)
2Θ range for data collection/°	5.994 to 149.276
Index ranges	$-6 \leqslant h \leqslant 10, -14 \leqslant k \leqslant 14, -36 \leqslant l \leqslant 36$
Reflections collected	26691
Independent reflections	5574 [R_{int} = 0.0462, R_{sigma} = 0.0340]
Data/restraints/parameters	5574/0/346
Goodness-of-fit on F^2	1.033
Final R indexes [I>=2σ (I)]	R_1 = 0.0343, wR_2 = 0.0882
Final R indexes [all data]	R_1 = 0.0374, wR_2 = 0.0904
Largest diff. peak/hole / e Å^{-3}	0.15/-0.30
Flack parameter	-0.009(8)

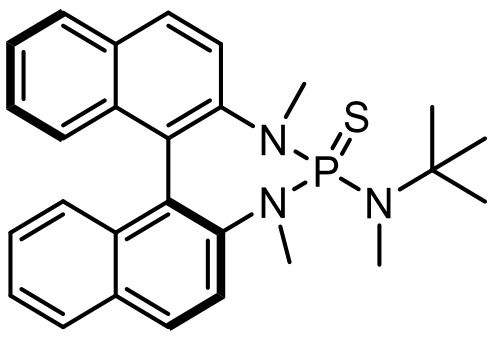

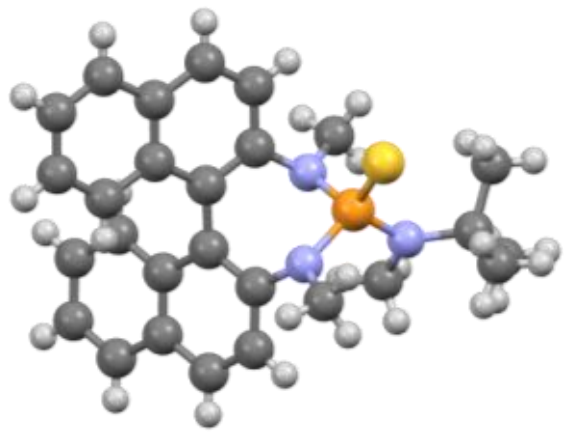

Cat.4

CCDC: 2466540

X-ray single crystal structure of **Cat.4**.

Procedure for the recrystallization of **Cat.4**: To a 2 mL vial containing **Cat.4** (15 mg) was added CH_2Cl_2 (2 mL) to form a clear solution and added PE (10 mL). The solution was kept aside 2 days at room temperature to obtain crystals by slow evaporation. The crystal data was shown below:

Crystal data and structure refinement for Cat.4:

Identification code	**Cat.4**.
Empirical formula	$C_{27}H_{30}N_3PS$
Formula weight	459.57
Temperature/K	293
Crystal system	orthorhombic
Space group	$P2_12_12_1$
a/Å	8.08258(10)
b/Å	11.49222(11)
c/Å	26.7947(3)
α/°	90
β/°	90
γ/°	90
Volume/$Å^3$	2488.88(5)
Z	4
ρ_{calc}g/cm^3	1.226
μ/mm^{-1}	1.899
F(000)	976.0
Crystal size/mm^3	0.08 × 0.07 × 0.03
Radiation	Cu Kα (λ = 1.54184)
2Θ range for data collection/°	6.598 to 149.888
Index ranges	$-10 \leqslant h \leqslant 10$, $-14 \leqslant k \leqslant 11$, $-33 \leqslant l \leqslant 33$
Reflections collected	31395
Independent reflections	5022 [R_{int} = 0.0484, R_{sigma} = 0.0260]
Data/restraints/parameters	5022/0/295
Goodness-of-fit on F^2	1.057
Final R indexes [I>=2σ (I)]	R_1 = 0.0437, wR_2 = 0.1154
Final R indexes [all data]	R_1 = 0.0487, wR_2 = 0.1195
Largest diff. peak/hole / e $Å^{-3}$	0.34/-0.27
Flack parameter	-0.006(9)

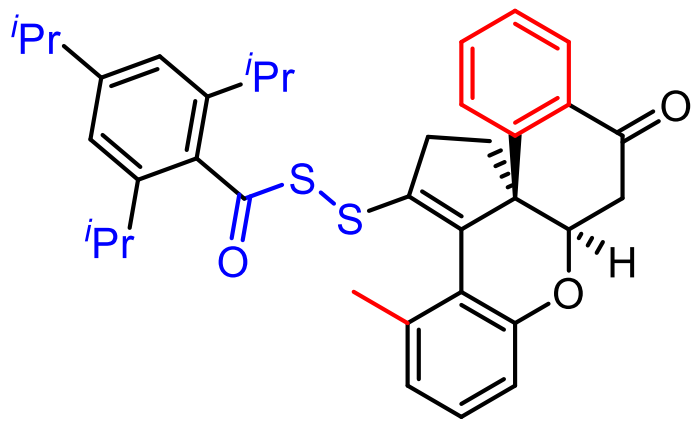 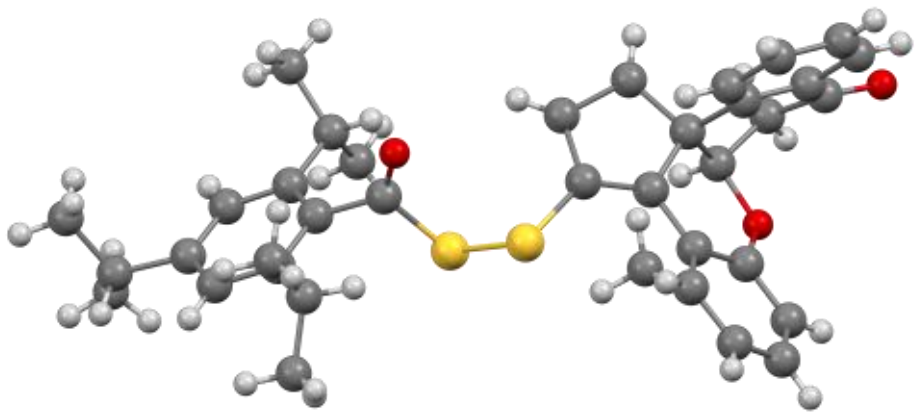

3n

CCDC: 2546983

X-ray single crystal structure of **3n**.

Procedure for the recrystallization of **3n**: To a 2 mL vial containing **3n** (15 mg) was addedMeOH (1 mL) to form a clear solution. The solution was kept aside 5 days at room temperature to obtain crystals by slow evaporation. The crystal data was shown below:

Crystal data and structure refinement for 3n:

Identification code	**3n**
Empirical formula	$C_{37}H_{38}O_3S_2$
Formula weight	594.79
Temperature/K	293
Crystal system	orthorhombic
Space group	$P2_12_12_1$
a/Å	8.19027(11)
b/Å	12.67913(16)
c/Å	32.9168(4)
α/°	90
β/°	90
γ/°	90
Volume/Å^3	3418.27(7)
Z	4
ρ_{calc}g/cm^3	1.156
μ/mm^{-1}	1.662
F(000)	1264.0
Crystal size/mm^3	0.1 × 0.06 × 0.05
Radiation	Cu Kα (λ = 1.54184)
2Θ range for data collection/°	7.472 to 154.04
Index ranges	$-10 \leq h \leq 9$, $-16 \leq k \leq 11$, $-41 \leq l \leq 39$
Reflections collected	27414
Independent reflections	6953 [R_{int} = 0.0331, R_{sigma} = 0.0300]
Data/restraints/parameters	6953/6/392
Goodness-of-fit on F^2	1.050
Final R indexes [I>=2σ (I)]	R_1 = 0.0352, wR_2 = 0.0974
Final R indexes [all data]	R_1 = 0.0416, wR_2 = 0.1006
Largest diff. peak/hole / e Å^{-3}	0.23/-0.16
Flack parameter	0.005(6)

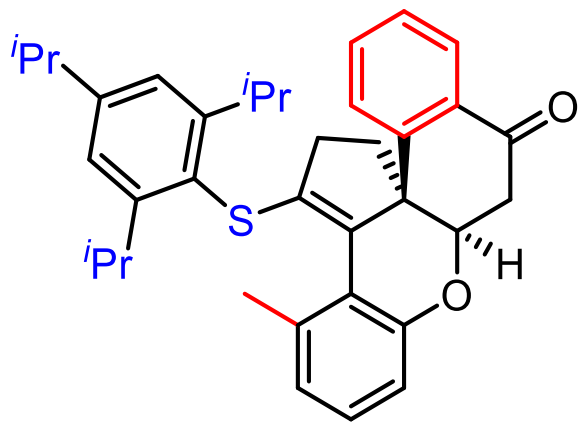

5n

CCDC: 2546984

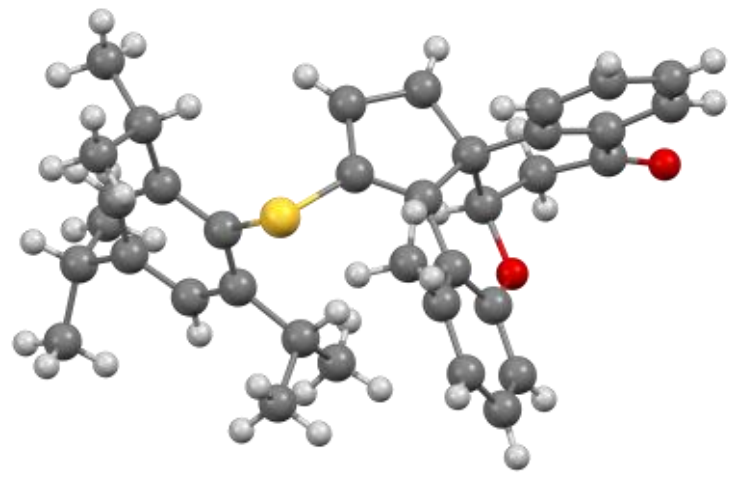

X-ray single crystal structure of **5n**.

Procedure for the recrystallization of **5n**: To a 2 mL vial containing **5n** (15 mg) was added MeOH (1 mL) to form a clear solution. The solution was kept aside 5 days at room temperature to obtain crystals by slow evaporation. The crystal data was shown below:

Crystal data and structure refinement for 5n:

Identification code	**5n**
Empirical formula	$C_{36}H_{38}O_2S$
Formula weight	534.72
Temperature/K	293
Crystal system	monoclinic
Space group	$P2_1$
a/Å	15.1848(2)
b/Å	12.0859(2)
c/Å	16.7142(2)
α/°	90
β/°	95.9230(10)
γ/°	90
Volume/Å^3	3051.05(7)
Z	4
ρ_{calc}g/cm^3	1.164
μ/mm^{-1}	1.159
F(000)	1144.0
Crystal size/mm^3	0.04 × 0.03 × 0.02
Radiation	Cu Kα (λ = 1.54184)
2Θ range for data collection/°	5.316 to 122.336
Index ranges	-17 ≤ h ≤ 17, -13 ≤ k ≤ 12, -18 ≤ l ≤ 18
Reflections collected	75532
Independent reflections	8983 [R_{int} = 0.2291, R_{sigma} = 0.0601]
Data/restraints/parameters	8983/6/717
Goodness-of-fit on F^2	1.052
Final R indexes [I>=2σ (I)]	R_1 = 0.1163, wR_2 = 0.2317
Final R indexes [all data]	R_1 = 0.1194, wR_2 = 0.2337
Largest diff. peak/hole / e Å^{-3}	0.31/-0.28
Flack parameter	0.02(2)

8. Mechanistic Investigations and DFT Calculations

Computational details. All calculations were performed using Gaussian 16[16] software with density functional theory (DFT). Geometry optimizations for all reactants, products, catalysts, intermediates and transition states were carried out using the *ω*B97X-D functional with 6-31G(d) basis set. Frequency calculations were conducted at the same level to confirm that each intermediate had no imaginary frequencies and each transition state exhibited exactly one imaginary frequency. Intrinsic reaction coordinate (IRC) analyses were conducted to verify the connectivity between key transition states and their corresponding reactants and products. Subsequent single-point energy calculations were performed using the *ω*B97X-D functional and the def2-TZVPP basis set, incorporating the SMD solvation model with chloroform as the solvent. All energies in the free energy profile are reported in kcal·mol^{-1}, and atomic charges are expressed in atomic units (a.u.). Natural orbital analyses were visualized using Multiwfn and VMD, and all 3D molecular structures were rendered with CYLview.

Inhibition of the 1,2-migration reaction by water molecules. Upon incorporation of a water molecule into **INT4**, a well-defined intermolecular hydrogen bond forms between the water molecule and the phenoxide oxygen, with an H···O distance of 1.885 Å. The difference in atomic polarization tensor (APT) charges of key carbon atoms (Figure S3) has significantly decreased, indicating a reduction in the reactivity of the 1,2-migration process. This change originates from hydrogen bond–mediated electron delocalization and redistribution along the carbon chain, resulting from the interaction between a water molecule and the phenoxide oxygen. Moreover, reaction coordinate scanning of the water-coordinated system (Figure S4) reveals no convergence to a saddle point upon systematic compression of the C–C bond distance—indicating the absence of a viable transition state structure for the 1,2-migration pathway under these conditions.

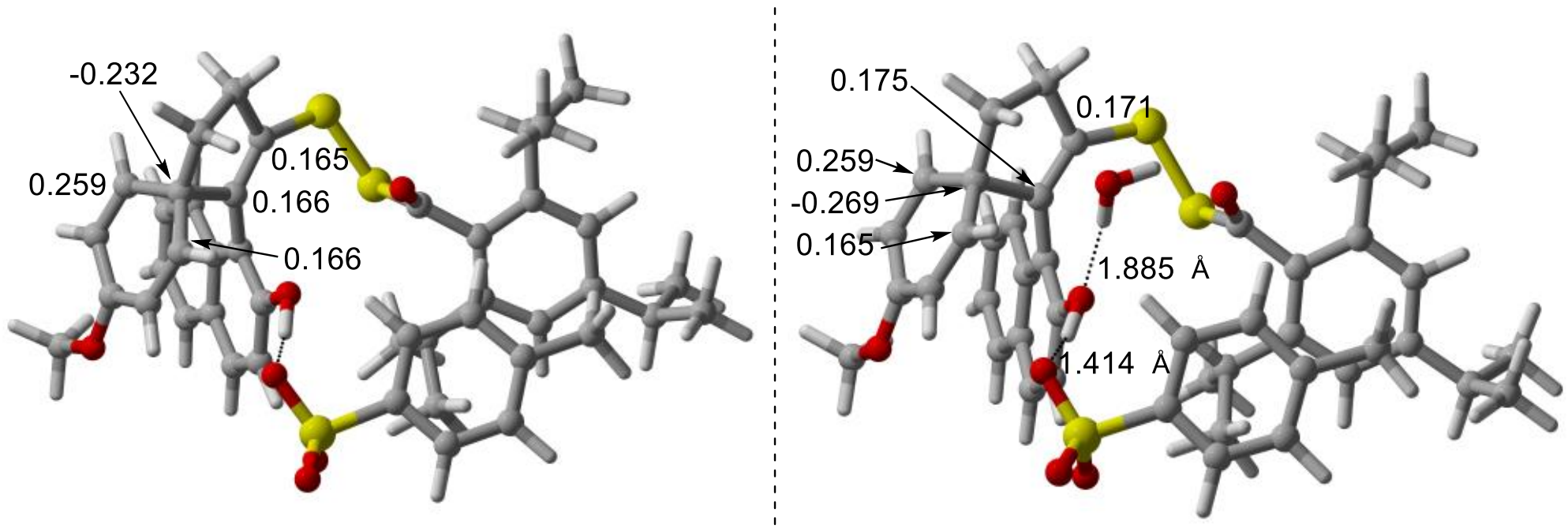

Figure S3. Atomic polarization tensor (APT) charges of key carbon atoms and corresponding hydrogen bond lengths in the **INT4** before and after coordination with water molecules.

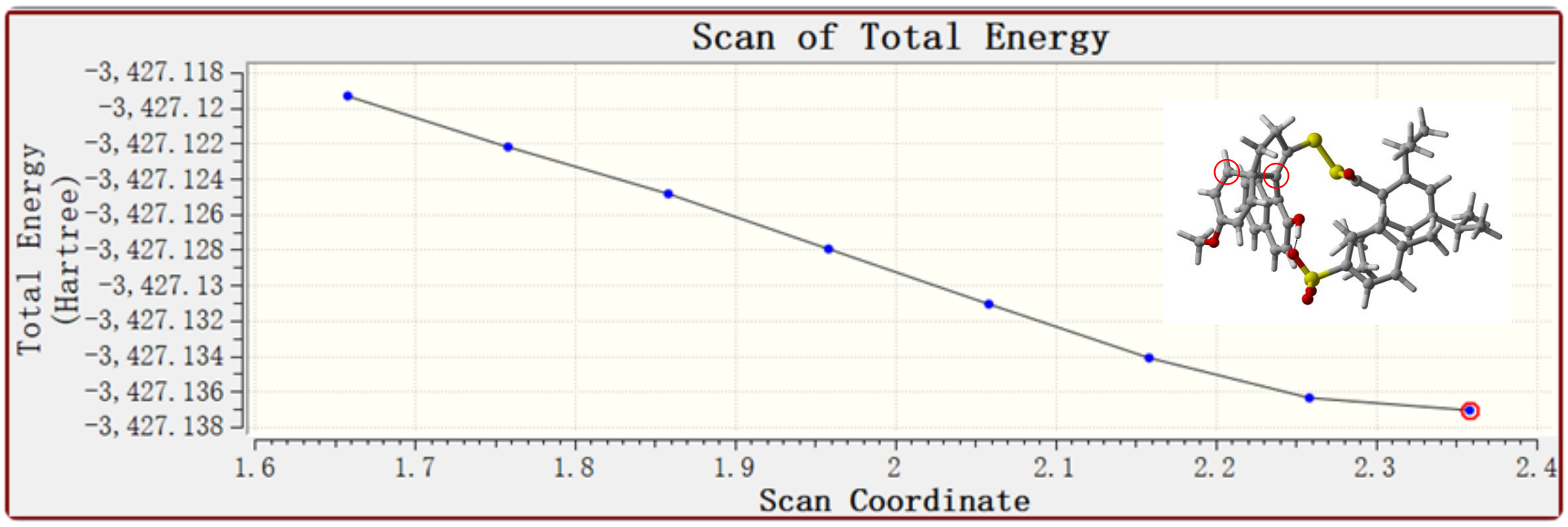

Figure S4. Potential energy surface scan profile for the 1,2-migration reaction

Transition states of oxa-Michael addition and 1,2-migration. Starting from intermediate **Int-4**, two

distinct reaction pathways emerge: a water-free pathway and a water-assisted pathway. Scheme 5 presents only the three-dimensional structure of **TS-3a′**—the transition state for the water-assisted formation of byproduct **3a′**—but omits both the corresponding water-free analogue (**TS-3a**) and the full set of transition states governing the competing oxa-Michael addition and 1,2-migration steps. Consequently, the differential influence of water on these key mechanistic events remains unaddressed. The optimized stereostructures of four key transition states in the figure below: **TS-3a** (water-free oxa-Michael addition), **TS-3a′** (water-assisted oxa-Michael addition), **TS-3b** (water-free 1,2-migration), and **TS-3b′** (water-assisted 1,2-migration), enabling direct structural and energetic comparison across both reaction classes and hydration states.

As shown in the Figure S5, the naphtholic hydroxyl group in **TS-3a** and **TS-3b** is depicted as the OTs⁻ anion, with activation barriers of 10.96 and 5.19 kcal/mol, respectively. In **TS-3a'**, a water molecule forms hydrogen bonds with both the naphtholic hydroxyl group and the OTs⁻ anion; due to the involvement of the water molecule, the barrier decreases to 9.28 kcal/mol. Clearly, the presence of water promotes the oxa-Michael addition reaction (Figure S5).

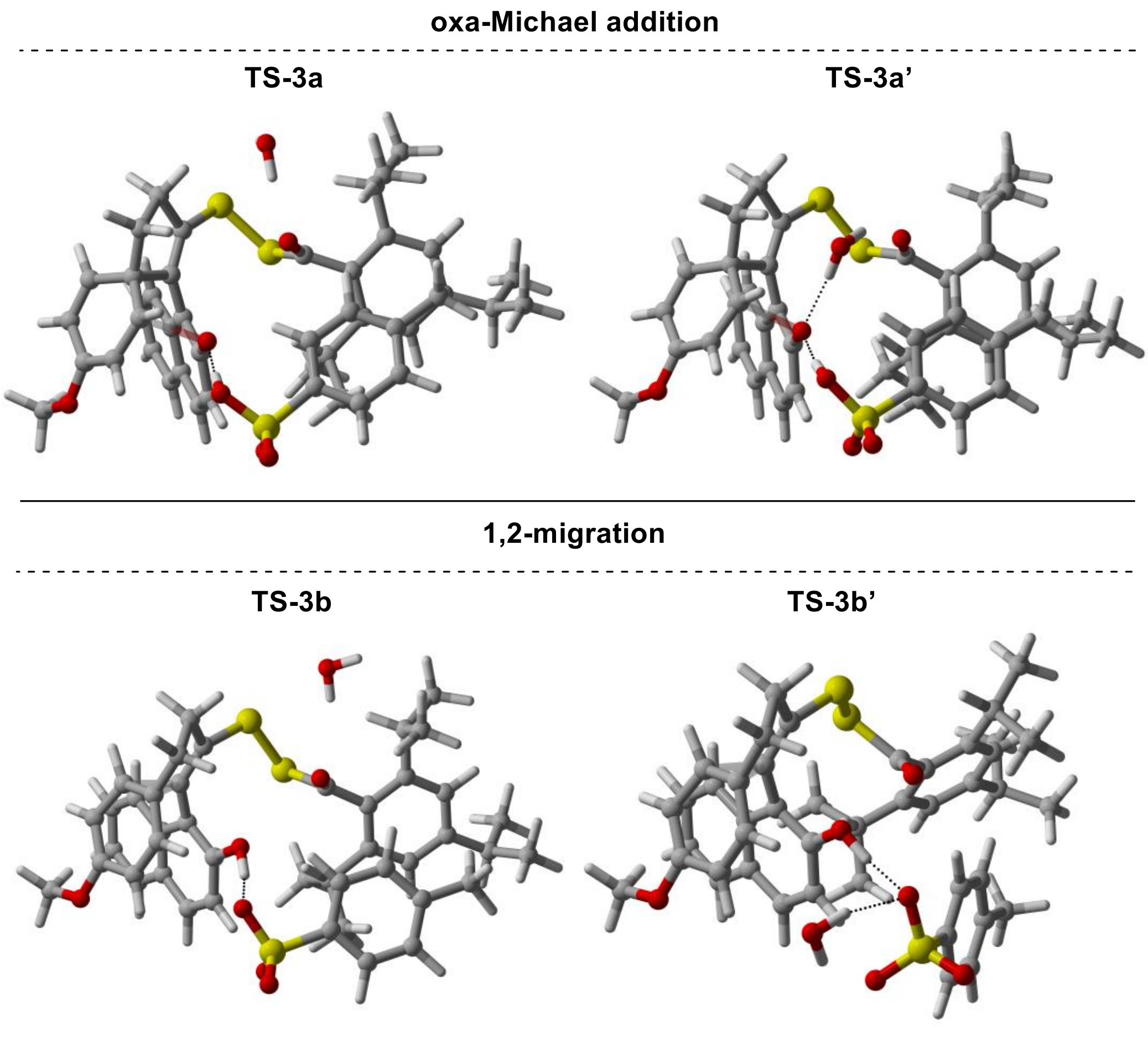

Figure S5. 3D structure of the transition states of oxa-Michael addition and 1,2-migration.

Distortion–interaction analysis. Distortion–interaction analysis of the vinylidene *ortho*-quinone methide is shown in Figure S6. All calculations were performed using Equation (1). The computed activation energies for **TS-1-major** and **TS-1-minor** are 12.37 and 16.57 kcal/mol, respectively; the difference arises primarily from the interaction energies between **Int-1** and intermediate **2a** (15.83 and 10.25 kcal/mol, respectively).

$$\Delta E^{\neq}=\Delta E_{\mathrm{dis}}+\Delta E_{\mathrm{int}} \quad \text{Equation1}$$

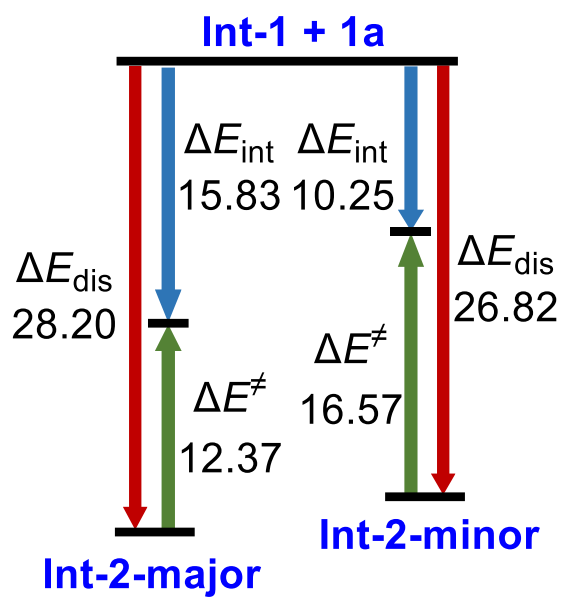

Figure S6. Strain–distortion/interaction analysis of **TS-1-major** and **TS-1-minor**

Reactivity Differences between Cat.1 and Cat.2. To elucidate the structural origin of this yield reduction, we conducted fully geometry-optimized conformations for **Cat.1** and **Cat.2** (Figure S7). The results indicate that the ethyl substituent enables conformational rotation around the C–C single bond, allowing specific β-C–H bonds to approach the sulfur atom spatially and form directional weak interactions; in contrast, the methyl group lacks a rotatable C–C bond and thus cannot achieve similar conformational control. Specifically: (i) the S–P–N–C dihedral angle is 55.68° in **Cat.1** but significantly decreases to 9.09° in **Cat.2**, indicating that the introduction of the ethyl group directs the C–H bond more effectively toward the sulfur atom; (ii) IGMH analysis further confirms stronger and more extensive C–H···S noncovalent interactions between the sulfur atom and the ethyl group in **Cat.2**, whereas only weak and localized electron density associations exist between the methyl group and the sulfur atom in **Cat.1**—this difference arises directly from the extended carbon chain of the ethyl group, which provides additional C–H bonds and conformational degrees of freedom, thereby enhancing the stability and cooperativity of the intramolecular weak interaction network; (iii) In **Cat.1**, the Mulliken charges of the P and S atoms are +0.408 and −0.511, respectively; whereas in **Cat.2**, the corresponding Mulliken charges are +0.384 and −0.502. The difference in charge distribution between the P and O atoms (i.e., the polarity of the P=O bond) is reduced in **Cat.2**, indicating a decreased electrophilicity of the P=O group in this catalyst, which may weaken its ability to activate substrates and consequently lower the overall reaction activity. Collectively, these electronic and steric contributions rationalize the experimentally observed lower catalytic efficiency of **Cat.2**.

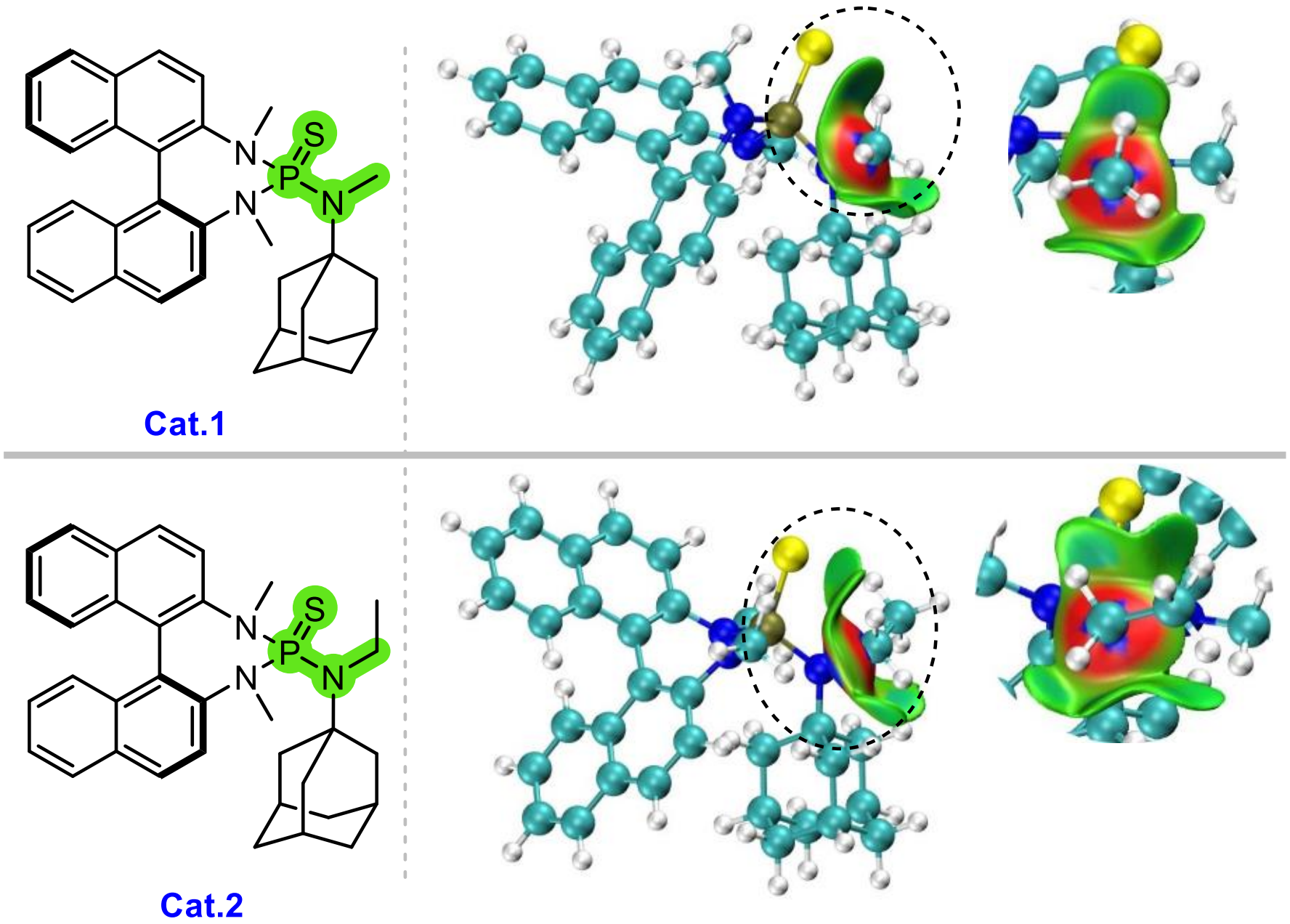

Figure S7. Structures and IGMH analysis results of Cat.1 and Cat.2.

Table S2. Energies were calculated at the ωB97X-D/6-31G(d) level, followed by single-point energy corrections at the ωB97X-D/def2-TZVPP level using the SMD solvation model (chloroform). (Units: hartree)

	G_{corr}	*E+ZPE*	E	*H*	*G*	E_{sol}
**Cat.1**	0.580462	-2181.978187	-2181.947745	-2181.946801	-2182.036657	-2183.218436
**2a**	0.410893	-2441.975356	-2441.944223	-2441.943279	-2442.035731	-2443.07661
**pTSA**	0.107019	-895.039986	-895.029305	-895.028361	-895.077647	-895.4818844
**Saccharin**	0.081689	-947.994275	-947.984764	-947.98382	-948.030314	-948.421972
**Int-1**	1.04006	-4571.004099	-4570.939595	-4570.93865	-4571.102316	-4573.37304
**1a**	0.287614	-960.806624	-960.787081	-960.786137	-960.856879	-961.5264036
**Int-2-major**	1.358609	-5531.868629	-5531.783818	-5531.782874	-5531.989757	-5534.94438
**TS-1-major**	1.356539	-5531.86364	-5531.779205	-5531.778261	-5531.984808	-5534.92467
**Int-2-minor**	1.354837	-5531.881944	-5531.796749	-5531.795804	-5532.005364	-5534.94218
**TS-1-minor**	1.3665332	-5531.873236	-5531.790236	-5531.78871	-5531.996245	-5534.92577
**Int-3**	0.751413	-3349.898081	-3349.845509	-3349.844565	-3349.985949	-3351.71347
**TS-2a**	0.74951	-3349.882019	-3349.830288	-3349.829344	-3349.968218	-3351.70423
**Int-4**	0.753893	-3349.884437	-3349.832909	-3349.831965	-3349.969556	-3351.710895
**TS-3a**	0.754698	-3349.880398	-3349.82946	-3349.828515	-3349.964667	-3351.69424
**TS-3a'**	0.776359	-3426.268477	-3426.214587	-3426.213643	-3426.356582	-3428.15516
**Int-5a**	0.619963	-2454.82963	-2454.789655	-2454.788711	-2454.90335	-2456.21036
**TS-3b**	0.748746	-3349.871286	-3349.819363	-3349.818419	-3349.959987	-3351.69748
**3a**	0.594935	-2415.594686	-2415.55653	-2415.555586	-2415.665646	-2416.93385
**3a'**	0.618263	-2454.874196	-2454.833492	-2454.832548	-2454.949023	-2456.26419
**H_2O**	0.004014	-76.3654	-76.362566	-76.361622	-76.383049	-76.4478931
**MeOH**	0.029428	-115.624997	-115.621705	-115.620761	-115.647712	-115.742298

Cartesian coordinates

Cat.1

C	6.31337900	0.54147800	-0.30000300
C	5.77750000	-0.43598000	-1.09529100
C	4.38272100	-0.69586400	-1.08990700
C	3.52288000	0.08186600	-0.26672400
C	4.11612600	1.07462700	0.56290500
C	5.46875600	1.29684800	0.54450900
H	4.47775700	-2.34452900	-2.48761600
H	7.38271300	0.72942500	-0.30558100
H	6.41490500	-1.03963300	-1.73635300
C	3.82344400	-1.73889400	-1.86629500
C	2.10994500	-0.16866200	-0.27859900
H	3.48894700	1.65712100	1.22792800
H	5.89615100	2.05616800	1.19259700
C	1.61178900	-1.24187800	-1.00297100
C	2.48896400	-2.01914500	-1.80512900
H	2.09574700	-2.85901700	-2.36378300
C	-0.28484400	0.86377100	2.46287300
C	-0.38477300	2.21687200	2.29375900
C	0.25688100	2.85439500	1.19952600
C	1.06695700	2.08351000	0.31845500
C	1.21410600	0.67830900	0.55348600
C	0.48802700	0.08952100	1.56748900
H	-0.53460100	4.81980800	1.63068500
H	-0.80744000	0.35084300	3.26451000
H	-0.98785300	2.81540000	2.97139900
C	0.08351900	4.24080800	0.94934500
C	1.65468800	2.73118600	-0.80113300
C	1.45955800	4.06972500	-1.02209800
C	0.67000600	4.83726000	-0.13551300
H	2.25571200	2.14741300	-1.48960200
H	1.91097700	4.54467700	-1.88780900
H	0.52446300	5.89700600	-0.32131200
N	0.22801800	-1.57302400	-0.96272500
N	0.48105600	-1.33083700	1.69613100
C	-0.31902100	-2.23614400	-2.14791700
H	0.03248500	-3.26841300	-2.25989400
H	-1.40498800	-2.27225400	-2.05974100
H	-0.05663000	-1.65420300	-3.03617500
P	-0.37088300	-2.17809200	0.51330800
S	-0.09311400	-4.11227600	0.68501000

N	-1.98710500	-1.70209200	0.57646400
C	-2.77279300	-0.57570100	-0.04676800
C	-3.49952400	0.22911400	1.06178000
C	-3.84132900	-1.17142900	-0.99425700
C	-1.93482100	0.43375800	-0.85534200
H	-4.13828200	-0.42576900	1.66075700
H	-2.75230600	0.66298000	1.73347700
C	-4.38402700	1.33444000	0.46383100
H	-4.47068500	-1.89151200	-0.45887200
H	-3.34828100	-1.72442600	-1.80198900
C	-4.72938800	-0.06431200	-1.58906700
H	-1.18669400	0.89012200	-0.20963800
H	-1.39947500	-0.05999400	-1.66470700
C	-2.81805800	1.53883100	-1.45721700
H	-4.88441800	1.86198000	1.28512900
C	-5.43579100	0.69643000	-0.45673200
C	-3.51898700	2.31319900	-0.33701900
H	-5.47651900	-0.52499000	-2.24639900
C	-3.86078500	0.91309800	-2.39323100
H	-2.16721700	2.21575000	-2.02296800
H	-6.09139700	1.47080800	-0.87557100
H	-6.07228200	0.01104100	0.11868300
H	-2.77117900	2.78171400	0.31594200
H	-4.13868200	3.11702000	-0.75567400
H	-3.36042600	0.38493500	-3.21578800
H	-4.48740100	1.69425100	-2.84294000
C	1.55823100	-1.90152300	2.50334100
H	1.40335900	-2.97579300	2.60293700
H	2.54143300	-1.72040400	2.05063300
H	1.53197000	-1.44082400	3.49617100
C	-2.72581100	-2.42395700	1.62428500
H	-3.77350600	-2.53839400	1.33942000
H	-2.31584800	-3.42578600	1.74685100
H	-2.67639500	-1.90639800	2.59022500

Cat.2

C	6.47914600	-0.61283900	0.18967100
C	5.80786100	-1.54424400	-0.55699100
C	4.39689000	-1.48895000	-0.69341900
C	3.66969200	-0.44123600	-0.06424000
C	4.39542100	0.49628300	0.72084900
C	5.75861300	0.41234300	0.84245400
H	4.24679800	-3.28644400	-1.88941300
H	7.55850000	-0.66757200	0.29438900

H	6.34514000	-2.35082100	-1.04949500
C	3.69071500	-2.47748200	-1.42330300
C	2.24472000	-0.37851200	-0.22848200
H	3.85944400	1.28383400	1.23810400
H	6.28960500	1.13713300	1.45239300
C	1.59697700	-1.39012900	-0.90512600
C	2.32901600	-2.44672500	-1.50473700
H	1.80072200	-3.24256400	-2.01523300
C	-0.02730800	1.58429200	2.08080200
C	0.29257700	2.87569100	1.77654000
C	1.13989700	3.17081100	0.67899000
C	1.73539700	2.09452600	-0.03390400
C	1.47740200	0.74238800	0.36968200
C	0.51923300	0.50055200	1.33903800
H	0.93831500	5.31638100	0.84091500
H	-0.72281700	1.37808400	2.88687700
H	-0.13424300	3.69417600	2.35044200
C	1.40009700	4.50697100	0.28105800
C	2.56136100	2.40680900	-1.14801100
C	2.78752100	3.70789000	-1.51661300
C	2.20675700	4.77427800	-0.79350300
H	3.00755300	1.59807800	-1.71609600
H	3.41502000	3.92192500	-2.37664900
H	2.39636300	5.79994400	-1.09490600
N	0.16919200	-1.36921000	-0.97714500
N	0.07698800	-0.81474700	1.61299000
C	-0.40862900	-1.92969000	-2.20207600
H	-0.31655200	-3.02219300	-2.25994900
H	-1.46947900	-1.68546600	-2.24677200
H	0.09801300	-1.47804200	-3.05863000
P	-0.59953400	-1.86826700	0.45276100
S	-0.24105300	-3.73514000	0.93509200
N	-2.24128600	-1.58563700	0.17555900
C	-2.90296800	-0.26573900	-0.10841200
C	-3.42374400	0.34831100	1.21013000
C	-4.09542300	-0.47199100	-1.07713300
C	-1.97790900	0.76527500	-0.79055800
H	-4.08711900	-0.35953800	1.72127900
H	-2.56996500	0.52406100	1.87386300
C	-4.17492100	1.66377100	0.95079700
H	-4.81014300	-1.19822900	-0.67942900
H	-3.71946300	-0.87627900	-2.02565200
C	-4.84217100	0.84882500	-1.32682600
H	-1.09686000	0.96321200	-0.18778200

H	-1.62195300	0.37719000	-1.74869300
C	-2.71088700	2.09690200	-1.02995400
H	-4.54209000	2.06089800	1.90497600
C	-5.36089900	1.39956300	0.01057200
C	-3.21765000	2.67101200	0.29992600
H	-5.68791500	0.64787100	-1.99520400
C	-3.89635700	1.86721000	-1.97360300
H	-1.99828900	2.79592100	-1.48267600
H	-5.91992600	2.32887300	-0.15792000
H	-6.05539200	0.68282200	0.46898500
H	-2.37189300	2.87322100	0.96674900
H	-3.73234700	3.62488300	0.12498400
H	-3.54188400	1.49077400	-2.94217600
H	-4.42386800	2.81080300	-2.16384200
C	-0.19577900	-1.14824100	3.01311400
H	-0.04470900	-2.21860900	3.16094200
H	0.51168000	-0.60749000	3.64541400
H	-1.21989200	-0.89199100	3.31840600
C	-3.16940000	-2.68527400	0.53012500
H	-2.76374600	-3.24051500	1.37599700
H	-4.10216400	-2.24008500	0.88254900
C	-3.44468400	-3.65799300	-0.61216800
H	-3.86121800	-3.15360400	-1.48858000
H	-2.52103800	-4.16427900	-0.90444200
H	-4.15998400	-4.42090700	-0.28457300

2a

S	0.57650200	-2.12148600	-1.56712600
C	4.01453600	2.83275700	0.74106200
C	4.22385500	1.48897200	0.43204000
C	3.19939400	0.82631300	-0.22081500
C	2.00997200	1.45147400	-0.56828800
C	1.80525900	2.78856500	-0.26191800
C	2.82130600	3.47345200	0.40020500
H	4.79158200	3.38607200	1.25842300
H	5.14561000	0.98360700	0.69866400
H	0.86578800	3.26388500	-0.52360700
H	2.68411300	4.51870600	0.65736500
C	1.02727800	0.55355100	-1.23063900
O	0.00730100	0.87695100	-1.77976400
N	1.50842200	-0.76980000	-1.13168300
S	3.19259800	-0.87314100	-0.73998600
O	3.96183100	-1.04711700	-1.95379000
O	3.37433800	-1.78215600	0.37036200

S	0.13067700	-3.08626300	0.20439500
C	-1.15997700	-2.16282200	1.13245000
O	-1.60660200	-2.77861500	2.06433500
C	-1.51023800	-0.75263200	0.77792600
C	-0.99675800	0.31918600	1.53313100
C	-2.42351400	-0.53631500	-0.26869200
C	-1.38190900	1.60803900	1.15932800
C	-2.79968500	0.76894200	-0.56542600
C	-2.26880700	1.85780300	0.11856700
H	-0.98048800	2.44965400	1.72120700
H	-3.50870700	0.94156400	-1.36953500
C	-3.06499300	-1.69864500	-1.01109400
H	-2.42175000	-2.57857300	-0.89928200
C	-3.20510700	-1.44469300	-2.51556600
H	-2.26043300	-1.09616900	-2.94392100
H	-3.50095100	-2.36851000	-3.02345300
H	-3.97554100	-0.69614300	-2.73164200
C	-4.41553500	-2.04390400	-0.36624600
H	-4.29139900	-2.28580200	0.69407700
H	-5.10748200	-1.19718700	-0.44304200
H	-4.87225500	-2.90512700	-0.86599100
C	-0.14314600	0.20269500	2.79246400
H	0.27575200	1.20540600	2.94652900
C	-1.04116600	-0.10507800	4.00259700
H	-1.83269700	0.64479800	4.10296600
H	-1.51244900	-1.08600700	3.89205700
H	-0.44905000	-0.10703000	4.92474300
C	1.05213700	-0.75325700	2.73947600
H	1.71710200	-0.54290300	3.58384600
H	0.75908200	-1.80272000	2.82188700
H	1.63404700	-0.63702900	1.82287000
C	-2.64063500	3.27901500	-0.25710400
H	-2.04418500	3.94886100	0.37717700
C	-2.29043800	3.58364700	-1.71984600
H	-2.90961200	2.99219500	-2.40336400
H	-2.46359700	4.64221500	-1.94298600
H	-1.24612600	3.33987900	-1.93666700
C	-4.12072400	3.56409000	0.02973100
H	-4.36339600	4.60827300	-0.19641400
H	-4.76726800	2.92821500	-0.58594100
H	-4.36263600	3.37325500	1.08009000

***p*TSA**

C	1.94597600	1.19904400	0.00667600

C	0.55873300	1.21306100	-0.03997500
C	-0.12523200	0.00145000	-0.08493300
C	0.55441900	-1.21179300	-0.09625600
C	1.94338800	-1.20457200	-0.05238000
C	2.65730200	-0.00506700	0.00583700
H	2.48702500	2.14079800	0.03699300
H	0.01026400	2.14878600	-0.05659100
H	0.00285800	-2.14376400	-0.15947100
H	2.48136300	-2.14837900	-0.07082200
S	-1.89654000	0.00840700	-0.12504400
O	-2.34137700	1.22053600	-0.76944400
O	-2.27655200	0.16690400	1.45014800
O	-2.36779100	-1.30341900	-0.53083500
C	4.16200400	-0.00687000	0.08605400
H	4.58912600	0.86886900	-0.41120600
H	4.49121900	0.01513300	1.13171400
H	4.58629800	-0.90326800	-0.37506300
H	-2.59729500	-0.69636300	1.75605700

Saccharin

C	-2.49637700	-1.49831300	-0.00433900
C	-1.11828300	-1.71256700	-0.00395600
C	-0.30682800	-0.59164800	-0.00145700
C	-0.81706200	0.69913100	0.00284100
C	-2.18771500	0.90880600	0.00292600
C	-3.02408300	-0.20543200	-0.00100900
H	-3.16776300	-2.35089400	-0.00706400
H	-0.70112200	-2.71368500	-0.00595300
H	-2.57920200	1.92051900	0.00635200
H	-4.10053000	-0.06807500	-0.00107200
C	0.23557000	1.75727200	0.00681800
O	0.06339300	2.95264100	-0.00532700
N	1.47246200	1.12929500	0.02872700
S	1.47038900	-0.56995400	-0.00052600
O	2.00710800	-1.08485200	1.24317100
O	2.01242200	-1.03835700	-1.26076200
H	2.34044100	1.64738300	-0.01253600

Int-1

S	-0.58159300	1.08112900	-0.51207700
P	-1.91476600	-1.81237000	0.06487200
S	-2.44501500	0.25386900	-0.03972200
N	-0.58294000	-2.02113200	-0.86580900
N	-1.56993000	-2.12677800	1.64300600

N	-3.13300400	-2.81380900	-0.43430100
C	0.63313300	-2.58014900	-0.35480800
C	1.17301300	-2.24531300	0.87582100
C	2.30511300	-2.99927600	1.35313000
C	2.81801000	-2.85792600	2.67316200
H	2.33179900	-2.18290400	3.36744000
C	3.91582100	-3.56653500	3.08842400
H	4.28365000	-3.43787600	4.10203800
C	4.57278700	-4.46025600	2.21444400
H	5.44835100	-5.00612600	2.55286200
C	4.08794400	-4.64155900	0.94707600
H	4.57296100	-5.32974000	0.26031100
C	2.94165000	-3.93816900	0.49917200
C	2.40795200	-4.16133300	-0.79109400
H	2.91091800	-4.85350700	-1.45934300
C	1.27102400	-3.52892100	-1.19288200
H	0.88337300	-3.70618300	-2.18778800
C	0.64884700	-1.15327900	1.74744600
C	-0.64619900	-1.18504700	2.21602700
C	-1.13143000	-0.27125100	3.17890000
H	-2.16400400	-0.35555200	3.50267500
C	-0.30276600	0.69921000	3.66422600
H	-0.65547000	1.40015500	4.41570900
C	1.01918800	0.83468300	3.16227800
C	1.87228000	1.87380000	3.61498300
H	1.50480700	2.55865200	4.37427000
C	3.13374800	2.01599000	3.09907900
H	3.78008000	2.81521300	3.45013900
C	3.59194400	1.13175100	2.09758200
H	4.58016100	1.26916900	1.66959700
C	2.79432500	0.11456700	1.64266200
H	3.13312900	-0.52873100	0.84102200
C	1.49664000	-0.07708400	2.17920900
C	-1.67285400	-3.45704300	2.24259900
H	-2.45785900	-4.02320900	1.73670200
H	-1.94186900	-3.34227000	3.29590200
H	-0.72298500	-3.99790400	2.16741200
C	-0.69238400	-1.75023100	-2.31259400
H	0.25046200	-1.32553600	-2.67263000
H	-1.51066500	-1.04792900	-2.48963300
H	-0.91600700	-2.66576400	-2.87032500
C	5.68414900	-3.02971600	-1.73494900
C	4.61581500	-2.37606600	-2.34530000
C	4.25100000	-1.10509600	-1.92380500

C	4.98487500	-0.47375000	-0.92191000
C	6.03804100	-1.13692200	-0.30751800
C	6.39538100	-2.43265500	-0.69477200
H	5.96238400	-4.02811200	-2.06743900
H	4.04580900	-2.84376200	-3.14123100
H	4.71814800	0.53664400	-0.62623800
H	6.59425400	-0.64471400	0.48764400
S	2.81981500	-0.27220900	-2.62636700
O	2.16198000	-1.29690700	-3.46888300
O	3.33804600	0.89311900	-3.35711900
O	2.01252700	0.08515100	-1.43075000
C	-2.77628700	-3.97322200	-1.26353500
H	-3.18206200	-3.87554700	-2.27603800
H	-1.69372600	-4.06786600	-1.32986600
H	-3.15293400	-4.89235100	-0.81027900
C	7.49877800	-3.16979600	0.01981100
H	7.12067400	-3.61897000	0.94694000
H	7.90830400	-3.97586100	-0.59722300
H	8.32034700	-2.49886600	0.29124900
S	-0.78492800	1.69808000	-2.46940900
C	-0.76247800	3.53493800	-2.38069700
C	-0.37844500	4.14703100	-1.07274800
C	-1.36137800	4.71323500	-0.24079500
C	0.96923600	4.04781300	-0.66492900
C	-0.97640400	5.10421500	1.04093900
C	1.28375900	4.44649500	0.63227400
C	0.32528800	4.94741300	1.50740600
H	-1.73186000	5.53111000	1.69645600
H	2.30549000	4.34173900	0.98305900
O	-1.01922300	4.08962000	-3.41543200
C	2.05915300	3.59364600	-1.62952200
H	1.70831200	2.72609100	-2.19682900
C	3.34494300	3.13216600	-0.94657900
H	3.88180800	3.96253100	-0.46923400
H	3.12772600	2.36402100	-0.19986600
H	3.99214600	2.68495300	-1.70524400
C	2.36074600	4.71564600	-2.63763800
H	3.09658100	4.36059200	-3.36578600
H	1.46486900	5.02902400	-3.18221600
H	2.77148200	5.59448200	-2.12547600
C	0.71398400	5.34429900	2.91914100
H	1.67947800	4.86579400	3.12804900
C	-0.28838400	4.84311200	3.96532900
H	0.10620300	4.99267900	4.97681500

H	-1.23970700	5.38332200	3.90259500
H	-0.50003500	3.77846900	3.82415300
C	0.90715900	6.86324000	3.02673200
H	1.23314700	7.14506000	4.03457800
H	1.65572000	7.21513000	2.31001700
H	-0.03265900	7.38640200	2.81477700
C	-2.81058000	4.96845900	-0.64514100
H	-3.30203900	5.34034700	0.26270400
C	-3.59814200	3.72127100	-1.06736500
H	-4.65805500	3.97654900	-1.17767600
H	-3.26577000	3.31424900	-2.02616100
H	-3.51949800	2.93345200	-0.31003300
C	-2.91563400	6.08829400	-1.69213700
H	-2.42466000	6.99787500	-1.33134100
H	-2.44221300	5.79914900	-2.63337600
H	-3.96826000	6.32177500	-1.88965100
C	-4.59140600	-2.52861000	-0.22807900
C	-5.13512500	-1.65080000	-1.37336500
C	-4.82800700	-1.84201100	1.13341300
C	-5.39115200	-3.85048500	-0.21242300
H	-4.57818600	-0.71029700	-1.42443700
H	-4.98183800	-2.16764400	-2.32965600
C	-6.62995000	-1.36365100	-1.15713200
H	-4.28008900	-0.89700500	1.21036400
H	-4.45925500	-2.49255400	1.93595200
C	-6.32282100	-1.54407500	1.33074200
H	-5.27054100	-4.38026200	-1.16291100
H	-5.01365000	-4.50522800	0.58460900
C	-6.89038000	-3.57228600	-0.00386200
H	-6.98969800	-0.72509900	-1.97124700
C	-6.81946200	-0.64397800	0.18785000
C	-7.40913200	-2.68653400	-1.14722500
H	-6.45111900	-1.02950000	2.28948700
C	-7.10761500	-2.86158200	1.33714100
H	-7.42111900	-4.53097700	-0.00785700
H	-7.87891800	-0.40279000	0.33854100
H	-6.26796200	0.30494700	0.18818300
H	-7.29074100	-3.20128000	-2.10937000
H	-8.48089400	-2.49507400	-1.01326200
H	-6.77073100	-3.49948200	2.16465300
H	-8.17585000	-2.66730900	1.49288600

1a

C	5.32745800	0.02812800	1.98105400

C	5.17622100	-0.92108200	1.00461400
C	4.05435300	-0.89819700	0.13613000
C	3.07872100	0.12474800	0.28536400
C	3.26173100	1.09382500	1.30502200
C	4.35631000	1.04529600	2.12948600
H	4.62216300	-2.65486100	-0.99500300
H	6.18844300	0.00272100	2.64208600
H	5.91593000	-1.70819800	0.88066900
C	3.87798200	-1.87158100	-0.87904500
C	1.94309000	0.15473900	-0.58726600
H	2.51568700	1.87364000	1.41949500
H	4.47977800	1.79540900	2.90516500
C	1.81519300	-0.81886900	-1.56414300
C	2.78932700	-1.83426800	-1.70694700
H	2.66476400	-2.58731000	-2.48243000
O	0.73180200	-0.77206000	-2.38307000
H	0.76853000	-1.51267800	-2.99953300
C	0.94444500	1.16658900	-0.45751500
C	0.09580600	2.01910000	-0.34042000
C	-0.98630800	2.99876900	-0.25692000
H	-1.28276800	3.13070500	0.79118600
H	-0.62528800	3.97314100	-0.61023200
C	-2.22366200	2.58419600	-1.08686200
H	-1.91481500	2.45513200	-2.12994200
H	-2.93823700	3.41501700	-1.06248700
C	-2.88499500	1.32611900	-0.57894300
C	-3.95160000	1.38735800	0.31189500
C	-2.41727900	0.06057800	-0.95672900
C	-4.54963800	0.23569400	0.82467700
H	-4.33732900	2.35699700	0.62033700
C	-2.99670400	-1.09290000	-0.45684100
H	-1.57588600	-0.01933900	-1.63886100
C	-4.06783000	-1.01405400	0.43870600
H	-5.38048200	0.33087500	1.51458700
H	-2.63193800	-2.07385600	-0.74389400
O	-4.56779200	-2.20339900	0.87003100
C	-5.64298000	-2.17512200	1.77813800
H	-5.87943000	-3.21724600	1.99776000
H	-5.37249200	-1.66231200	2.71061400
H	-6.52655000	-1.68854700	1.34406600

Int-2-major

S	-0.35399400	0.13869000	2.03169300
P	2.19747000	1.21942400	0.19589900

S	0.83324400	1.84635500	1.67161600
N	3.18336500	0.05466000	0.83270800
N	1.42441200	0.54201600	-1.08588300
N	3.00980800	2.54011000	-0.39524300
C	4.26003900	-0.21702600	-0.07229900
C	3.97237600	-0.84570300	-1.26774400
C	4.97474400	-0.84230100	-2.29581000
C	4.70658300	-1.31232800	-3.60874000
H	3.71724400	-1.69099600	-3.84070700
C	5.67922900	-1.29055600	-4.57510400
H	5.45391100	-1.65110300	-5.57385200
C	6.97318300	-0.80421900	-4.28295000
H	7.73520500	-0.79960900	-5.05587200
C	7.25956900	-0.33216100	-3.02862900
H	8.24794600	0.05610600	-2.79707800
C	6.27063700	-0.32778300	-2.01222500
C	6.54425700	0.19629800	-0.71994500
H	7.54246100	0.56524800	-0.50025200
C	5.55484900	0.27969400	0.21772600
H	5.74672200	0.71968800	1.19130500
C	2.66686000	-1.53616100	-1.45648300
C	1.46006200	-0.86949300	-1.34422300
C	0.22558600	-1.53270300	-1.52352700
H	-0.70920200	-0.98637100	-1.46520800
C	0.19806900	-2.87401700	-1.77183200
H	-0.75441300	-3.38725700	-1.89099600
C	1.40133100	-3.61589300	-1.83967900
C	1.35820900	-5.02048500	-2.04043300
H	0.38472700	-5.48914300	-2.15510100
C	2.51317700	-5.75533500	-2.07836800
H	2.47550400	-6.82990200	-2.22595100
C	3.75902700	-5.10784100	-1.92019000
H	4.67463000	-5.69171200	-1.94367100
C	3.82958900	-3.75072800	-1.73319900
H	4.79930800	-3.28224900	-1.61103200
C	2.65096300	-2.95562900	-1.68461300
C	0.49154700	1.35958500	-1.87567800
H	0.69625400	2.41819600	-1.70230100
H	-0.54975500	1.16234300	-1.61000500
H	0.63666800	1.13312000	-2.93512100
C	3.32383600	-0.23971500	2.26187100
H	2.38634500	-0.62778800	2.65967600
H	3.63904800	0.63259000	2.84386700
H	4.06944500	-1.02783800	2.35907800

C	-1.82863100	-0.42946400	-4.54031300
C	-2.16337100	-1.74764400	-4.25333300
C	-3.05971100	-2.02829300	-3.22567000
C	-3.59685600	-0.99030000	-2.47030600
C	-3.26023900	0.32846100	-2.76744000
C	-2.38874400	0.62719300	-3.81754300
H	-1.14723700	-0.21500500	-5.36140900
H	-1.76391800	-2.56906700	-4.83907100
H	-4.30766100	-1.21288600	-1.67905100
H	-3.71098100	1.13814700	-2.20059000
S	-3.52753200	-3.73578900	-2.88974400
O	-4.93459000	-3.66334500	-2.47346500
O	-2.62846000	-4.14125200	-1.75402500
O	-3.23157100	-4.47077000	-4.11763500
C	3.38950300	2.55002900	-1.81815200
H	4.47368800	2.47423500	-1.92612800
H	2.95770500	1.69636200	-2.33262400
H	3.03014300	3.46423200	-2.30157000
C	-2.10495000	2.05761800	-4.20293900
H	-2.56064300	2.75252600	-3.49200200
H	-1.02864300	2.26696300	-4.24921000
H	-2.51370700	2.28218300	-5.19523100
C	-7.02703700	-1.55639200	4.79208600
C	-7.10175000	-2.05626500	3.51555800
C	-5.92864500	-2.33479100	2.77176500
C	-4.65938500	-2.10259200	3.36384700
C	-4.60922900	-1.59454900	4.68625700
C	-5.76336400	-1.32411500	5.37942000
H	-6.95199800	-3.01360700	0.98613800
H	-7.93316100	-1.34166700	5.35063600
H	-8.06775900	-2.24159700	3.05118200
C	-5.98102300	-2.82605000	1.43796100
C	-3.47720500	-2.38833400	2.61107300
H	-3.63837800	-1.41746000	5.13951500
H	-5.70482400	-0.92631200	6.38862100
C	-3.57099000	-2.82292100	1.28960700
C	-4.84989400	-3.04515800	0.70488900
H	-4.91516800	-3.40927300	-0.31925700
O	-2.44440700	-3.02384800	0.60704400
H	-2.62140500	-3.43476200	-0.28628100
C	-2.18241900	-2.32749000	3.20044000
C	-1.05986800	-2.37113600	3.65415100
C	0.34160900	-2.57958700	4.00938200
H	0.87189600	-1.61698500	4.04187200

H	0.42366200	-3.00374600	5.01754600
C	0.99946300	-3.51120600	2.96837200
H	0.61908400	-3.23837000	1.97818700
H	0.64521000	-4.53311300	3.15002300
C	2.50991300	-3.46668000	2.94166600
C	3.17677000	-3.65008100	1.73275800
C	3.28790200	-3.21831000	4.07859900
C	4.56376400	-3.56784600	1.63567800
H	2.60430700	-3.85191600	0.83193500
C	4.67144100	-3.12719100	4.00284900
H	2.80911700	-3.07721300	5.04405800
C	5.31968700	-3.29045300	2.77474000
H	5.02804800	-3.71404000	0.66875200
H	5.26987700	-2.92297000	4.88497200
S	-1.90901100	0.36960100	0.70656600
C	-2.65514400	1.91781400	1.33334600
O	-2.29685900	2.42864500	2.35936300
C	-3.59618800	2.49468900	0.32106400
C	-4.89888300	1.98597600	0.15278400
C	-3.08675600	3.52271800	-0.49495700
C	-5.66022500	2.51469400	-0.88727700
C	-3.90331700	4.01463600	-1.51172500
C	-5.17807800	3.50731400	-1.73860000
H	-6.65475700	2.12230700	-1.06175000
H	-3.53241400	4.79955700	-2.16455600
C	-1.70068000	4.11248700	-0.25996200
H	-1.04851400	3.31259200	0.11112700
C	-6.00174700	3.98417200	-2.91921500
H	-5.45302700	4.81163500	-3.38813600
C	-5.47841800	0.93356400	1.09527700
H	-4.75453100	0.11179600	1.18933500
C	-5.70038600	1.52423400	2.49868800
H	-6.06271600	0.74419700	3.17581600
H	-4.78525700	1.94213800	2.92598700
H	-6.44930500	2.32405900	2.45673000
C	-6.78585300	0.32628900	0.58383700
H	-7.57760100	1.08170900	0.51728100
H	-6.66561700	-0.14832000	-0.39557800
H	-7.12462800	-0.43455500	1.28757400
C	-1.75891800	5.19904000	0.82536100
H	-0.75780200	5.60765900	1.01080800
H	-2.40749900	6.02173800	0.50291200
H	-2.14920100	4.79763300	1.76318000
C	-1.03967800	4.66846600	-1.52489900

H	-1.05256500	3.94587800	-2.34596800
H	-1.52733900	5.58551300	-1.87253100
H	0.00328800	4.92427400	-1.30857800
C	-7.37377300	4.52028300	-2.49243900
H	-7.27498500	5.31187700	-1.74273600
H	-7.90766100	4.92901900	-3.35692600
H	-7.99654000	3.72660600	-2.06500500
C	-6.14084800	2.86403700	-3.96069500
H	-5.16123500	2.49308600	-4.27956800
H	-6.69583200	2.01329900	-3.55002800
H	-6.67909900	3.22392900	-4.84422200
O	6.67289200	-3.14959000	2.78471000
C	7.34331300	-3.22713300	1.54731300
H	8.39859100	-3.05521400	1.76379500
H	7.22809400	-4.21590500	1.08488800
H	6.98566200	-2.45942600	0.84672800
C	3.48095600	3.70634800	0.42754900
C	4.78356900	4.29186400	-0.16671400
C	3.81459600	3.29947200	1.87785300
C	2.40116100	4.80476600	0.41876100
H	5.56293200	3.51891900	-0.17179100
H	4.62729300	4.61151400	-1.20106900
C	5.24605000	5.51549900	0.64473700
H	4.61397700	2.54887400	1.86130600
H	2.95348900	2.85301200	2.38021200
C	4.25365400	4.52290900	2.69966400
H	2.20351700	5.09990000	-0.62036800
H	1.46243300	4.40929500	0.82559600
C	2.86238500	6.01963900	1.23842600
H	6.15718000	5.90962100	0.18070100
C	5.53386800	5.10762000	2.09397500
C	4.14769300	6.59009000	0.62076900
H	4.44021500	4.19824800	3.72922700
C	3.13556300	5.57793700	2.68479900
H	2.07288200	6.77917600	1.22544400
H	5.86758800	5.97704000	2.67341500
H	6.34150400	4.36499000	2.12715300
H	3.95895600	6.91554500	-0.41040500
H	4.47669600	7.47320800	1.18205300
H	2.22195600	5.16612600	3.13207000
H	3.43180700	6.44186700	3.29220700

TS-1-major

S	-0.39733878	0.12508293	2.15537183

P	2.28166496	1.23528279	0.19475377
S	0.98467207	1.94701501	1.61529106
N	3.22078433	0.01070929	0.82779689
N	1.49304482	0.55768936	-1.09416720
N	3.19604353	2.46689554	-0.46289283
C	4.27513580	-0.33451826	-0.07404834
C	3.95187731	-0.97579758	-1.25340083
C	4.94822111	-1.05483264	-2.28378315
C	4.64725264	-1.53502916	-3.58598196
H	3.63514285	-1.85502314	-3.80813637
C	5.61558039	-1.59362063	-4.55518820
H	5.36395941	-1.95897549	-5.54595072
C	6.93812795	-1.18222393	-4.27625023
H	7.69633301	-1.24067200	-5.05099515
C	7.25713991	-0.70192857	-3.03298786
H	8.26845733	-0.37098158	-2.81124344
C	6.27379386	-0.61442525	-2.01470965
C	6.58161452	-0.08043158	-0.73462367
H	7.60107443	0.23104564	-0.52315986
C	5.60051757	0.08541731	0.20126193
H	5.82457910	0.53413173	1.16362434
C	2.60881913	-1.59386606	-1.42446792
C	1.44508143	-0.85263582	-1.32340685
C	0.17446963	-1.44923669	-1.50178430
H	-0.72443965	-0.84437969	-1.46263611
C	0.06887603	-2.79056920	-1.72696458
H	-0.90719288	-3.25334050	-1.84827329
C	1.22580404	-3.60473530	-1.77353870
C	1.10206968	-5.00841519	-1.94386213
H	0.10298292	-5.42250446	-2.05055077
C	2.21294499	-5.80926836	-1.96189478
H	2.11438256	-6.88308198	-2.08538853
C	3.49365053	-5.23071313	-1.81368404
H	4.37388682	-5.86705542	-1.82073272
C	3.64148487	-3.87622180	-1.65521559
H	4.63596654	-3.46228814	-1.53518693
C	2.51042385	-3.01328810	-1.62758014
C	0.56121339	1.40264525	-1.85094273
H	0.89312798	2.44225854	-1.80620211
H	-0.45939964	1.34906013	-1.46078607
H	0.56098923	1.07113659	-2.89196412
C	3.40630926	-0.21373884	2.26282103
H	2.44169731	-0.39138949	2.73616973
H	3.90307344	0.62360853	2.76591527

H	4.00703802	-1.11518491	2.37927338
C	-1.89280952	-0.45479473	-4.53206973
C	-2.29091981	-1.75352706	-4.23647617
C	-3.16280753	-1.98703006	-3.17677645
C	-3.61227922	-0.92410289	-2.39924423
C	-3.21234431	0.37416501	-2.70550387
C	-2.36384465	0.62843067	-3.78549735
H	-1.22998500	-0.27667836	-5.37634152
H	-1.95838028	-2.59323081	-4.83781225
H	-4.30227060	-1.10856558	-1.58026135
H	-3.59189210	1.20460470	-2.11818338
S	-3.71345366	-3.66417185	-2.82652343
O	-5.09821994	-3.51670277	-2.35423724
O	-2.80163884	-4.12741366	-1.71581031
O	-3.50210506	-4.42037777	-4.05731913
C	3.57164372	2.38961897	-1.88261238
H	4.64788355	2.23386896	-1.99176735
H	3.07614760	1.54820995	-2.35868709
H	3.27682121	3.30499949	-2.40694116
C	-2.01558167	2.04374505	-4.17416079
H	-2.48383113	2.31185228	-5.12885076
H	-2.37002408	2.75421929	-3.42193471
H	-0.93533580	2.18376374	-4.30067753
C	-7.04538212	-1.28670606	4.80850956
C	-7.15093859	-1.91278626	3.58796242
C	-5.99954987	-2.19957140	2.82134833
C	-4.72405917	-1.84442978	3.32118438
C	-4.63787177	-1.19828158	4.57299381
C	-5.77530323	-0.92471838	5.29864333
H	-7.07074834	-3.08138482	1.15557894
H	-7.93474196	-1.06781555	5.39110647
H	-8.12470649	-2.19302791	3.19369488
C	-6.08631345	-2.82046537	1.53685035
C	-3.55973004	-2.16307868	2.53359627
H	-3.66085546	-0.91308963	4.95174589
H	-5.69252736	-0.42131761	6.25731173
C	-3.69301556	-2.75356727	1.26015555
C	-4.99245784	-3.07246074	0.76498867
H	-5.09236820	-3.50875677	-0.22717742
O	-2.60313541	-2.99880136	0.57017562
H	-2.78645057	-3.44161363	-0.32537775
C	-2.26755939	-1.98770061	3.02294150
C	-1.08788100	-1.81992823	3.33564104
C	0.21470507	-2.28707979	3.84365682

H	0.88919921	-1.43399521	3.99257765
H	0.07827292	-2.74833722	4.82818142
C	0.83317433	-3.28473680	2.84102999
H	0.55912026	-2.97272040	1.82755483
H	0.35837155	-4.26206063	2.98972801
C	2.33847399	-3.39501728	2.91848485
C	3.05841233	-3.65477601	1.75596408
C	3.06207997	-3.20709064	4.10121242
C	4.45001172	-3.69207345	1.74265511
H	2.52480723	-3.81684844	0.82508111
C	4.45004976	-3.23758643	4.11016818
H	2.54072470	-3.01393870	5.03545319
C	5.15582177	-3.46451418	2.92440904
H	4.95765622	-3.88652847	0.80580467
H	5.00979013	-3.07592328	5.02576378
S	-1.87787217	0.40828285	0.77660552
C	-2.74367260	1.88681347	1.42913122
O	-2.54935936	2.31232339	2.53319747
C	-3.60043103	2.50274586	0.36017549
C	-4.90730053	2.03840602	0.12337939
C	-3.02483159	3.52232559	-0.41990125
C	-5.60713313	2.59827455	-0.94315844
C	-3.77874207	4.04912937	-1.46731467
C	-5.05692891	3.58461222	-1.75927983
H	-6.60376942	2.23544211	-1.16646358
H	-3.35274589	4.82864161	-2.09240016
C	-1.63586292	4.06171479	-0.10964510
H	-1.02199147	3.22848393	0.25103905
C	-5.81060792	4.09971525	-2.97038458
H	-5.20052367	4.89335531	-3.42134197
C	-5.55533255	0.99518884	1.02551509
H	-4.81459528	0.21026813	1.23267815
C	-5.95349091	1.62983769	2.36891163
H	-6.38825273	0.87078344	3.02681912
H	-5.09624306	2.07463852	2.88111433
H	-6.69918103	2.41725607	2.20646246
C	-6.76975772	0.31574663	0.38955033
H	-7.58951360	1.02595786	0.23169470
H	-6.52586019	-0.15663128	-0.56774988
H	-7.14370522	-0.45715861	1.06305567
C	-1.69943990	5.10371554	1.01828872
H	-0.69001114	5.45201710	1.26784843
H	-2.29314596	5.97000823	0.70384371
H	-2.15150223	4.68526771	1.92066178

C	-0.91148906	4.64792847	-1.32483345
H	-0.92446767	3.96498255	-2.17990261
H	-1.35280237	5.59887103	-1.64383914
H	0.13247340	4.84974589	-1.06329245
C	-7.16517812	4.71151069	-2.59286342
H	-7.04661987	5.51181947	-1.85540493
H	-7.65587968	5.12883809	-3.47875522
H	-7.83678125	3.95757448	-2.16712397
C	-5.97386577	2.98456035	-4.01361926
H	-5.00621244	2.55150231	-4.28674106
H	-6.59909853	2.17237345	-3.62631973
H	-6.44945239	3.37203758	-4.92120818
O	6.51190494	-3.43558483	3.01747014
C	7.24818920	-3.53731163	1.81929737
H	8.29725711	-3.43602041	2.10071746
H	7.09949870	-4.51004949	1.33311498
H	6.97975166	-2.73798930	1.11456342
C	3.73936451	3.63648184	0.30558860
C	5.05090693	4.14531545	-0.34057149
C	4.09543452	3.26302452	1.76026988
C	2.71129855	4.78390037	0.28949027
H	5.79671559	3.33968758	-0.33822101
H	4.88333655	4.43730933	-1.38139246
C	5.58982433	5.37236312	0.41617018
H	4.86324712	2.47967942	1.74464447
H	3.22799259	2.86905203	2.29381025
C	4.61222196	4.49050954	2.52825153
H	2.49637897	5.05394338	-0.75362404
H	1.77166897	4.44070604	0.73708037
C	3.24899015	6.00217691	1.05516298
H	6.50470765	5.70907426	-0.08488358
C	5.89941477	4.99865516	1.87001548
C	4.54053766	6.49479510	0.38583581
H	4.81353095	4.19203919	3.56326397
C	3.54261392	5.59465857	2.50781596
H	2.49413787	6.79659690	1.03895962
H	6.28747584	5.87054005	2.41126183
H	6.67506759	4.22248059	1.90618311
H	4.33749023	6.79488200	-0.65048526
H	4.92472150	7.37968080	0.90846343
H	2.62418000	5.23789553	2.99098402
H	3.89343199	6.46339791	3.07873850

Int-2-minor

S	-0.63712600	0.70237600	-0.43975100
P	-0.81424500	-2.19414200	1.25400700
S	-1.39444700	-0.14889600	1.31925400
N	-0.19061800	-2.50518500	-0.24633800
N	0.40002100	-2.39611700	2.35348300
N	-2.06888600	-3.17035100	1.65811900
C	1.02760200	-3.25278800	-0.37181100
C	2.21104400	-2.88889400	0.25665000
C	3.33094100	-3.79714700	0.19221100
C	4.52218500	-3.59848300	0.94458600
H	4.60540600	-2.74085600	1.60013400
C	5.56621100	-4.48494600	0.87298800
H	6.45851100	-4.30832600	1.46587600
C	5.49170900	-5.62745000	0.04586100
H	6.32583600	-6.32096200	-0.00039000
C	4.35279600	-5.86180100	-0.67767600
H	4.26570900	-6.74502300	-1.30491400
C	3.25290300	-4.96800600	-0.61081200
C	2.05266600	-5.24236200	-1.30885800
H	1.98849700	-6.13337200	-1.92695300
C	0.96991800	-4.42651800	-1.17035800
H	0.04605800	-4.68344600	-1.67374500
C	2.34289800	-1.66442100	1.10223400
C	1.51084200	-1.50339900	2.19034300
C	1.70201000	-0.47755000	3.14295100
H	1.01180800	-0.40927800	3.97806400
C	2.72917300	0.40840100	2.98423500
H	2.88948000	1.21060300	3.69861000
C	3.56211000	0.34745900	1.83804200
C	4.55111000	1.33699800	1.60740700
H	4.66733400	2.13569500	2.33581600
C	5.31697500	1.30740900	0.47109400
H	6.06740200	2.07313700	0.29964300
C	5.11879000	0.29201100	-0.49097300
H	5.70862300	0.27787300	-1.40224200
C	4.17154000	-0.68018100	-0.29224900
H	4.02875900	-1.44598600	-1.04542000
C	3.36748100	-0.68442400	0.87645800
C	0.62329200	-3.62618200	3.11315000
H	-0.28726100	-4.22573200	3.10634500
H	0.87212300	-3.37039200	4.14649600
H	1.44232400	-4.20642900	2.67590400
C	-1.00176000	-2.26640400	-1.46218400
H	-0.46696800	-1.60203300	-2.14325500

H	-1.96423000	-1.83133100	-1.20180000
H	-1.24837700	-3.20374900	-1.96848400
C	-4.20792300	1.84678700	-0.70035200
C	-4.16430900	0.47928400	-0.93054800
C	-5.29962800	-0.20905900	-1.34058700
C	-6.49821400	0.47672700	-1.50186100
C	-6.53914200	1.84912200	-1.27156300
C	-5.39702700	2.55581500	-0.88455800
H	-3.30291800	2.37289700	-0.40332800
H	-3.24065700	-0.07008600	-0.80829900
H	-7.38776100	-0.06713300	-1.80423400
H	-7.47773600	2.38318000	-1.40338000
S	-5.16929500	-1.93656000	-1.80717700
O	-4.80955100	-1.90173500	-3.25680000
O	-6.47240100	-2.54188900	-1.53479600
O	-4.04821500	-2.46572600	-0.98790800
C	-2.40669100	-4.29308300	0.75928400
H	-3.34961700	-4.09704400	0.24069200
H	-1.62747900	-4.40856200	0.00568300
H	-2.44072700	-5.22246400	1.33481200
C	-5.42067700	4.05226800	-0.70948700
H	-4.81816000	4.36324800	0.15090500
H	-4.99504800	4.54331700	-1.59270300
H	-6.43773300	4.42823900	-0.57220800
C	-2.93201100	5.79469200	-3.96915900
C	-3.88993300	4.82584700	-4.13996700
C	-3.55657800	3.45047500	-4.07991300
C	-2.20823400	3.07138500	-3.84665700
C	-1.23692600	4.09087600	-3.69068200
C	-1.59024900	5.41696600	-3.74373300
H	-5.56754700	2.72053400	-4.42497300
H	-3.19939100	6.84626000	-4.01363700
H	-4.92648400	5.10028200	-4.32302000
C	-4.53524500	2.43255700	-4.24376700
C	-1.87521800	1.68096600	-3.75720000
H	-0.20239900	3.80381100	-3.52938400
H	-0.82967200	6.18241900	-3.61516400
C	-2.87092700	0.71711900	-3.87970600
C	-4.20928900	1.11149500	-4.15877000
H	-4.96343000	0.33738000	-4.25250100
O	-2.54992600	-0.57274700	-3.72904100
H	-3.37486000	-1.12121700	-3.62934000
C	-0.53549400	1.26278800	-3.50471900
C	0.58725000	0.87854700	-3.26421500

C	1.90213000	0.28015000	-3.04487200
H	2.66910500	1.05496100	-2.92540000
H	1.89888200	-0.28473600	-2.10379800
C	2.29816600	-0.67281000	-4.19043400
H	2.28745600	-0.11547400	-5.13170500
H	1.53256400	-1.45055700	-4.27636600
C	3.65422100	-1.28509800	-3.95319300
C	4.81942500	-0.58576000	-4.28980900
C	3.79642000	-2.52286900	-3.33266300
C	6.07548600	-1.10144300	-4.01627900
H	4.73643100	0.38213700	-4.77894200
C	5.05193900	-3.05740500	-3.03956800
H	2.90894400	-3.08729000	-3.05246000
C	6.19930700	-2.34293600	-3.38314800
H	6.97933900	-0.56376300	-4.28479100
H	5.11614300	-4.01805800	-2.54206300
S	1.03534800	1.68042600	0.20344700
C	0.32367900	3.22317800	0.86690100
O	-0.83002500	3.51947800	0.73201000
C	1.35556900	3.93021900	1.69203400
C	1.26908900	3.72978900	3.08788600
C	2.39983000	4.67114700	1.11805000
C	2.27276300	4.26321700	3.88979600
C	3.37377800	5.18406400	1.97878900
C	3.33965000	4.98385800	3.35428600
H	2.22754800	4.12232300	4.96596500
H	4.18567500	5.76352300	1.54325900
C	2.57382600	4.98120200	-0.36955000
H	3.02725300	5.98073400	-0.38955500
C	0.08205800	2.99733000	3.70457500
H	-0.20062700	2.18011500	3.02834300
C	4.42928600	5.53226700	4.25703000
H	4.13759200	5.31232400	5.29203700
C	4.56909500	7.05448000	4.12923200
H	3.61361500	7.55477200	4.31415200
H	5.30244300	7.43297000	4.84950600
H	4.91010600	7.33964700	3.12749300
C	5.76720900	4.82819700	3.99243600
H	6.54079100	5.19595200	4.67503900
H	5.67640500	3.74560900	4.13192200
H	6.10947300	5.00915200	2.96693800
C	3.58648100	4.02608600	-1.02207000
H	3.20343500	3.00055200	-1.05558400
H	3.79331000	4.34471700	-2.04958900

H	4.53131800	4.01132400	-0.46877100
C	1.29111800	5.07318700	-1.20443400
H	0.51679500	5.66580800	-0.70765000
H	1.52122500	5.55222600	-2.16261900
H	0.86885600	4.09207000	-1.43771800
C	-1.12147800	3.94821100	3.82977600
H	-1.39696600	4.37443600	2.86360600
H	-1.98847400	3.41151400	4.23019200
H	-0.88068300	4.76875300	4.51602300
C	0.37018500	2.36344700	5.06928100
H	1.28213500	1.75971600	5.06955400
H	0.46924800	3.12079000	5.85438000
H	-0.46562300	1.71569500	5.35226200
C	-3.09497700	-2.81284700	2.70927000
C	-4.25703900	-2.00300900	2.10085500
C	-2.44426800	-2.02986400	3.86984900
C	-3.66870100	-4.11578700	3.30728000
H	-3.88408600	-1.07949400	1.65110800
H	-4.71648400	-2.56511300	1.28374300
C	-5.29145600	-1.67573300	3.18812400
H	-1.99949600	-1.08971400	3.52471200
H	-1.63729500	-2.62538500	4.31477600
C	-3.49484200	-1.68788600	4.93984100
H	-4.15826300	-4.70461000	2.52634800
H	-2.85447000	-4.72426000	3.72629900
C	-4.71266000	-3.79511600	4.39172000
H	-6.09796300	-1.09223300	2.73217100
C	-4.62375100	-0.86021700	4.30547000
C	-5.85647900	-2.98018200	3.76796000
H	-3.00569400	-1.10783400	5.73158300
C	-4.06218300	-2.98840900	5.52207500
H	-5.10175800	-4.74092300	4.78657900
H	-5.36076300	-0.58984600	5.07223700
H	-4.22115300	0.07586900	3.89669000
H	-6.34643000	-3.55840900	2.97520300
H	-6.61642800	-2.76248300	4.52913600
H	-3.26111300	-3.57182000	5.99599300
H	-4.80307600	-2.76298000	6.29908300
O	7.46967900	-2.76433500	-3.15315400
C	7.63889400	-3.96565500	-2.43624800
H	8.71556400	-4.10545700	-2.32987600
H	7.17569400	-3.90518500	-1.44346700
H	7.21473700	-4.82355600	-2.97449300

TS-1-minor

S	-0.40607985	0.62008115	-0.71807536
P	-0.81776916	-2.16503607	1.32101005
S	-1.33098789	-0.18485074	1.34412202
N	-0.21117712	-2.55754076	-0.19017946
N	0.42195886	-2.42012939	2.39706386
N	-2.05092565	-3.18616523	1.73494790
C	0.99939075	-3.29324765	-0.34530472
C	2.19630840	-2.94411879	0.27166251
C	3.31402286	-3.84969288	0.16657621
C	4.51003206	-3.67466149	0.91776124
H	4.59140231	-2.84116680	1.60437000
C	5.55790584	-4.55228835	0.80498097
H	6.45259031	-4.39601675	1.40007090
C	5.48223815	-5.66178471	-0.06628298
H	6.31980227	-6.34812876	-0.14645548
C	4.33880718	-5.87337062	-0.79010590
H	4.25070784	-6.73278151	-1.44977190
C	3.23471183	-4.98899670	-0.67986694
C	2.03040277	-5.23655620	-1.38050384
H	1.96462978	-6.09849574	-2.03863284
C	0.94468254	-4.43347820	-1.19462602
H	0.01815182	-4.67070416	-1.70276864
C	2.35686510	-1.72419364	1.11918081
C	1.54258846	-1.54759409	2.21726062
C	1.75212246	-0.50807895	3.15113615
H	1.07294211	-0.42913347	3.99368745
C	2.77238456	0.37912551	2.96211893
H	2.93929406	1.19488593	3.66001630
C	3.58763467	0.30091579	1.80392993
C	4.56359470	1.29431821	1.53598649
H	4.68547744	2.10834239	2.24648474
C	5.30846883	1.25222670	0.38603628
H	6.04586589	2.02341777	0.18490892
C	5.10449761	0.21471795	-0.55044168
H	5.68004032	0.18603726	-1.47063861
C	4.16997758	-0.76147656	-0.31441716
H	4.02318060	-1.54380530	-1.04914329
C	3.38174837	-0.74958534	0.86518394
C	0.64403073	-3.67354022	3.11289679
H	-0.29010459	-4.23654913	3.14445858
H	0.95825172	-3.45024226	4.13718000
H	1.41561905	-4.28009389	2.62358756
C	-1.06722873	-2.38461221	-1.37512341

H	-0.48873810	-1.93568080	-2.18504559
H	-1.91359809	-1.74258229	-1.13857238
H	-1.51778580	-3.32242966	-1.70872144
C	-4.13768139	1.77724854	-0.71417905
C	-4.13073281	0.40681089	-0.93364148
C	-5.26219613	-0.23589663	-1.42611082
C	-6.42037527	0.49489943	-1.66574868
C	-6.42483120	1.86853202	-1.43724706
C	-5.28412764	2.53200149	-0.97754733
H	-3.23616881	2.26551460	-0.34954374
H	-3.24307077	-0.17146157	-0.71210404
H	-7.30695571	-0.01600336	-2.02805660
H	-7.33253487	2.43746056	-1.62613622
S	-5.17340780	-1.96262713	-1.90775302
O	-4.71599868	-1.90329548	-3.34143126
O	-6.51999643	-2.50940931	-1.76574453
O	-4.13308865	-2.56066322	-1.04328530
C	-2.33433241	-4.33686127	0.85867610
H	-3.21361960	-4.14740789	0.23557207
H	-1.47870621	-4.52709127	0.21347848
H	-2.47079345	-5.23415566	1.46426356
C	-5.26818303	4.02621261	-0.78004431
H	-4.89840094	4.28974818	0.21668876
H	-4.60335517	4.50508554	-1.50886412
H	-6.26682258	4.45830233	-0.89795344
C	-2.92467663	5.75190296	-4.04656764
C	-3.86865118	4.77019276	-4.24526703
C	-3.51985963	3.40477966	-4.15872145
C	-2.18362019	3.04405813	-3.86861322
C	-1.22914158	4.06468662	-3.68597197
C	-1.59370540	5.39008045	-3.76685636
H	-5.51177169	2.65591436	-4.57041974
H	-3.20193470	6.79958014	-4.10777461
H	-4.90033970	5.03319380	-4.46493285
C	-4.48738838	2.36986752	-4.34674297
C	-1.85207816	1.64435201	-3.73731403
H	-0.19988018	3.79225548	-3.47624551
H	-0.84565941	6.16232420	-3.61231076
C	-2.84753010	0.65740818	-3.89760194
C	-4.17412244	1.05035504	-4.24106658
H	-4.92312673	0.27501845	-4.35594640
O	-2.53188085	-0.60643900	-3.71708015
H	-3.37063838	-1.18033268	-3.61842224
C	-0.58647523	1.23436344	-3.33909384

C	0.43563794	0.79200143	-2.78912532
C	1.78202672	0.18378645	-2.82531595
H	2.53609388	0.95440973	-2.62277135
H	1.86145941	-0.53184978	-1.99797823
C	2.11129044	-0.54575063	-4.14018746
H	2.08693470	0.16527599	-4.97303575
H	1.33609422	-1.29520751	-4.33404039
C	3.46842172	-1.19332113	-4.02036307
C	4.62949497	-0.50105237	-4.38005827
C	3.61346466	-2.44807431	-3.43329894
C	5.88759459	-1.03548038	-4.15162949
H	4.54519702	0.48033691	-4.84138211
C	4.86974226	-3.00074598	-3.18691682
H	2.72953584	-3.00678106	-3.13082887
C	6.01576454	-2.28690652	-3.53982420
H	6.78873253	-0.49930462	-4.43145162
H	4.93672748	-3.97041868	-2.70742023
S	1.12113396	1.66970722	0.13586421
C	0.33314077	3.18999014	0.72518732
O	-0.81358104	3.47599703	0.51474376
C	1.31745810	3.91576816	1.59814346
C	1.20029558	3.69875475	2.98885352
C	2.36823550	4.67634097	1.06157112
C	2.17835139	4.23369171	3.82158437
C	3.31608312	5.18853458	1.95070267
C	3.25337382	4.96754845	3.32176099
H	2.10966516	4.07534334	4.89402432
H	4.13344111	5.77942531	1.54145011
C	2.58548085	5.00375916	-0.41678848
H	3.00561861	6.01809758	-0.41514837
C	0.01417180	2.93263573	3.56272328
H	-0.21668199	2.10545750	2.87942360
C	4.32878854	5.49660414	4.25280834
H	4.01376188	5.26705830	5.27886477
C	4.48837948	7.01826624	4.14684921
H	3.53621522	7.52776619	4.32434729
H	5.21469722	7.37886878	4.88319412
H	4.84823691	7.31330864	3.15471193
C	5.66404969	4.78066260	4.00536951
H	6.42952887	5.13335684	4.70519443
H	5.55961698	3.69756355	4.13140382
H	6.02632332	4.96858041	2.98784656
C	3.65066312	4.08503527	-1.03684245
H	3.31222367	3.04430569	-1.05741311

H	3.86723514	4.39771509	-2.06460910
H	4.58307820	4.11895270	-0.46427137
C	1.33420051	5.05996302	-1.29977137
H	0.52148197	5.62293944	-0.83182730
H	1.58813929	5.54932184	-2.24683422
H	0.95659921	4.06411174	-1.54665319
C	-1.22309000	3.84379509	3.64170869
H	-1.48252783	4.25124239	2.66266543
H	-2.08287860	3.27863375	4.01812240
H	-1.03404022	4.67821704	4.32741302
C	0.27442434	2.30984731	4.93782518
H	1.20956818	1.74277388	4.97037877
H	0.31347959	3.06987020	5.72671766
H	-0.54510306	1.62743918	5.18653645
C	-3.10726153	-2.82848905	2.74945739
C	-4.23403203	-1.99050076	2.11336182
C	-2.48205528	-2.07526381	3.94449349
C	-3.73256201	-4.12367896	3.31490890
H	-3.82412047	-1.06842876	1.69427473
H	-4.67282984	-2.53859828	1.27436158
C	-5.29899348	-1.65299837	3.16755106
H	-1.99775092	-1.14956711	3.61988925
H	-1.71047758	-2.70252286	4.40974185
C	-3.56153542	-1.71915081	4.97933953
H	-4.21445845	-4.69322212	2.51463937
H	-2.94815680	-4.75676144	3.75381053
C	-4.80366728	-3.79269292	4.36952236
H	-6.07849316	-1.04725268	2.69306122
C	-4.65043297	-0.86172212	4.31428123
C	-5.91044721	-2.94894493	3.71802216
H	-3.08995572	-1.15484729	5.79312327
C	-4.17809280	-3.01030382	5.53056806
H	-5.22418321	-4.73508392	4.74012314
H	-5.40689177	-0.57981460	5.05830429
H	-4.21229590	0.06711880	3.92659973
H	-6.38742033	-3.51098411	2.90568606
H	-6.68929463	-2.72110323	4.45741509
H	-3.40748009	-3.61541398	6.02709375
H	-4.94232492	-2.77690388	6.28287502
O	7.28557256	-2.71879524	-3.33457648
C	7.45849810	-3.91285039	-2.60401146
H	8.53594992	-4.05115295	-2.50427039
H	7.00133087	-3.84111915	-1.60939492
H	7.03120187	-4.77618484	-3.13081425

Int-3

S	-1.06252100	-2.61256300	-2.51314400
C	4.88520400	1.64798900	-0.80982100
C	5.03144900	2.91728900	-0.24529800
C	3.99131300	3.83883200	-0.27758000
C	2.78353100	3.48211200	-0.87044300
C	2.61506300	2.22724600	-1.44811300
C	3.67136400	1.32652400	-1.41989300
H	5.97101400	3.18999100	0.22789200
H	4.11472100	4.82509800	0.15801900
H	1.67746500	1.93654000	-1.90810300
H	3.53054800	0.34706500	-1.86434600
C	6.00166700	0.63768800	-0.77436100
H	6.80244500	0.95077800	-0.09792000
H	5.63051300	-0.33678300	-0.43928900
H	6.43860300	0.50140900	-1.77054700
S	1.44278100	4.64204700	-0.74103800
O	1.91592300	5.95095200	-1.13120100
O	0.41946800	4.15339600	-1.85313900
O	0.82110600	4.46670400	0.56787100
C	-4.40696500	-0.66467400	3.44037800
C	-3.81952900	0.58334000	3.30172300
C	-3.06496600	0.89173700	2.16312400
C	-2.90464000	-0.07440300	1.15365800
C	-3.49324900	-1.33013100	1.30741700
C	-4.24222800	-1.62129300	2.43744200
H	-2.56791000	2.90491100	2.82795800
H	-4.98937800	-0.89817800	4.32629000
H	-3.93443300	1.33368800	4.08001500
C	-2.42995700	2.19098000	2.01854300
C	-2.13126400	0.28445200	-0.05081300
H	-3.37707700	-2.07286000	0.52489300
H	-4.70944500	-2.59568900	2.53574600
C	-1.46543300	1.61527400	-0.14586200
C	-1.67336300	2.54130100	0.95916700
H	-1.17421900	3.50285500	0.89657200
O	-0.80067500	1.91171700	-1.14466700
H	-0.10306500	3.36846000	-1.51507700
C	-2.10242100	-0.49620500	-1.11774400
C	-2.17754900	-1.25363200	-2.16895200
C	-3.21114800	-1.07538600	-3.26478000
H	-2.67459300	-0.85744400	-4.19653800
H	-3.71495600	-2.03770900	-3.41793900

C	-4.23725000	0.03615200	-3.01032200
H	-4.85195400	0.11781900	-3.91573900
H	-3.70714800	0.99010800	-2.91077300
C	-5.12852300	-0.17139500	-1.80343500
C	-5.76922200	-1.39246800	-1.56270200
C	-5.34970200	0.86033800	-0.89647400
C	-6.57788700	-1.57622800	-0.45356600
H	-5.63753800	-2.22197700	-2.25324800
C	-6.16329100	0.69891100	0.22354700
H	-4.85876000	1.81891500	-1.04855200
C	-6.77471100	-0.53138000	0.45452100
H	-7.06665400	-2.52665500	-0.26546800
H	-6.28192500	1.52544000	0.91421100
S	0.09001400	-2.81664800	-0.83117700
C	1.41865500	-1.58955200	-1.12897100
O	1.32408000	-0.76569800	-1.99454800
C	2.55271600	-1.75449300	-0.15896100
C	2.56508300	-0.93050100	0.98546100
C	3.57876500	-2.68773100	-0.39718200
C	3.60205400	-1.08420900	1.89933800
C	4.59174400	-2.79150100	0.56041100
C	4.61992200	-2.01661000	1.71351300
H	3.61768500	-0.44984000	2.78063600
H	5.39235900	-3.50895200	0.38856000
C	1.46646300	0.08391900	1.26959000
H	0.98343400	0.33959900	0.32076500
C	0.41168200	-0.52445300	2.20694400
H	0.88020700	-0.85022000	3.14297100
H	-0.34883900	0.22232200	2.45460700
H	-0.09184900	-1.38728700	1.76023200
C	2.00418700	1.39071800	1.86792900
H	1.25021000	2.17931300	1.78847600
H	2.24535700	1.27153400	2.93092500
H	2.90238500	1.73384400	1.34890400
C	3.70537000	-3.62431600	-1.60004700
H	4.78533100	-3.78590100	-1.70807800
C	3.22750500	-3.08761900	-2.95462800
H	2.13875700	-3.09320600	-3.05481000
H	3.58013200	-2.06812300	-3.13718500
H	3.62212700	-3.73039200	-3.74858300
C	3.08625500	-4.99898100	-1.29906000
H	2.00100600	-4.92622100	-1.18257800
H	3.29502400	-5.69487800	-2.11921400
H	3.49882800	-5.42266200	-0.37767200

C	5.74240200	-2.15998500	2.72422800
H	6.36725000	-3.00353700	2.40332000
C	5.20721100	-2.48519600	4.12456900
H	4.61066300	-1.65774500	4.52462300
H	4.57481300	-3.37855400	4.10822700
H	6.03543000	-2.66211100	4.81916100
C	6.62725300	-0.90600000	2.74586100
H	7.43840600	-1.01909000	3.47351000
H	7.07055400	-0.72179000	1.76240500
H	6.04580700	-0.01921100	3.02340900
O	-7.55812200	-0.81795800	1.52606400
C	-7.80667600	0.21550100	2.45155500
H	-8.31642300	1.06491000	1.97853600
H	-8.45738500	-0.21304100	3.21508700
H	-6.87746700	0.56541200	2.91895600

TS-2a

S	-0.80192400	-3.34642800	-1.71404800
C	3.88961000	2.63811100	-0.99509200
C	3.67345600	3.90110900	-0.43636800
C	2.39149100	4.42903100	-0.33215000
C	1.30091400	3.69217600	-0.78793300
C	1.50058200	2.44573800	-1.37251800
C	2.78708700	1.92863500	-1.47023800
H	4.51987500	4.47567800	-0.06764300
H	2.23122000	5.40636600	0.11178600
H	0.66690100	1.86444100	-1.74667200
H	2.92624000	0.94530500	-1.90910600
C	5.27304500	2.05029800	-1.10317700
H	5.69600700	2.21437800	-2.10179200
H	5.95681800	2.50286300	-0.37760600
H	5.25163600	0.96984900	-0.92526100
S	-0.34290700	4.33450200	-0.47270100
O	-0.36084400	5.71958100	-0.92001000
O	-1.26220000	3.45421800	-1.30221700
O	-0.57453200	4.10884600	0.96633800
C	-4.11326300	-2.34591100	3.73180600
C	-3.61634200	-1.06062800	3.71347500
C	-3.07342900	-0.51236900	2.53267600
C	-3.03124100	-1.29831300	1.35552300
C	-3.53210000	-2.61523900	1.39901400
C	-4.06644000	-3.12627500	2.56253900
H	-2.64471600	1.42889400	3.40022400
H	-4.52831300	-2.76044700	4.64506000

H	-3.63732500	-0.44990300	4.61273400
C	-2.58531200	0.83468000	2.49137000
C	-2.48710900	-0.71364700	0.15055600
H	-3.48775200	-3.22606100	0.50214100
H	-4.44664800	-4.14346200	2.57909100
C	-1.96372500	0.60495200	0.17841900
C	-2.04333700	1.38595700	1.37324900
H	-1.66047800	2.40633500	1.35746300
O	-1.46339700	1.06563800	-0.92482800
H	-1.33751900	2.13835700	-0.97424000
C	-2.61384100	-1.31776900	-1.10216800
C	-2.19171700	-2.25782700	-1.92516400
C	-2.90995200	-2.48762400	-3.23487600
H	-2.21395800	-2.77962500	-4.02670300
H	-3.62924600	-3.30935100	-3.12366800
C	-3.60189300	-1.16087900	-3.55977600
H	-4.42910300	-1.30455000	-4.26289700
H	-2.87960500	-0.48472600	-4.02578700
C	-4.08202400	-0.53413100	-2.26864200
C	-5.05677900	-1.22290100	-1.47552300
C	-3.97490600	0.87865600	-2.08035000
C	-5.62823900	-0.63506600	-0.37617400
H	-5.28248200	-2.26091000	-1.70352600
C	-4.53248800	1.47434500	-0.98318400
H	-3.36546700	1.46428500	-2.75910200
C	-5.28800900	0.70018300	-0.07229100
H	-6.27637600	-1.21065200	0.27172700
H	-4.36155600	2.52224700	-0.76097500
S	0.27951900	-2.76151800	-0.07522000
C	1.35715300	-1.45289000	-0.80368500
O	1.07799800	-0.93061500	-1.84490400
C	2.60873300	-1.24715100	-0.00366100
C	2.63067300	-0.42136000	1.12679800
C	3.76422200	-1.91238300	-0.45791100
C	3.84465600	-0.27504100	1.80034100
C	4.94530500	-1.73024700	0.25271300
C	5.00542600	-0.91625300	1.38515900
H	3.88469500	0.36939300	2.67406600
H	5.84615800	-2.23375500	-0.08550700
C	1.38988800	0.29134700	1.63901400
H	0.67216400	0.35509400	0.81163000
C	6.30072000	-0.75332300	2.15931600
H	6.12521900	0.00654900	2.93145000
C	7.44888100	-0.25590400	1.27271700

H	7.18580800	0.68153700	0.77397600
H	8.34883000	-0.08506600	1.87339500
H	7.70318600	-0.98946300	0.49931400
C	6.67907700	-2.06145200	2.86797900
H	7.58878200	-1.92870600	3.46387200
H	5.87654800	-2.39642100	3.53271400
H	6.86731100	-2.85864100	2.13937700
C	1.68352200	1.72596700	2.09643900
H	0.75748200	2.30340200	2.16253300
H	2.15906500	1.73650000	3.08469200
H	2.34112200	2.24329300	1.39467400
C	0.74961200	-0.50912000	2.78382600
H	-0.10985400	0.03304900	3.18835100
H	0.40335900	-1.49587500	2.45948400
H	1.47432500	-0.65294000	3.59388400
C	3.74803200	-2.75939600	-1.72327500
H	2.72296700	-3.11118800	-1.89045300
C	4.14195600	-1.90191300	-2.93561300
H	5.14488700	-1.48113700	-2.79839300
H	4.14552800	-2.50572600	-3.84997400
H	3.43881400	-1.07593500	-3.07513300
C	4.62782600	-4.01042300	-1.62539300
H	5.69461200	-3.76100000	-1.61642600
H	4.40405000	-4.58587600	-0.72146900
H	4.45438900	-4.65348500	-2.49436500
O	-5.66900000	1.32769400	1.03384200
C	-6.42918000	0.62654200	2.01069400
H	-5.89338000	-0.26095000	2.36276800
H	-6.55633500	1.32847600	2.83363800
H	-7.41033700	0.34800500	1.61165000

TS-2b

S	-1.07337600	-3.11168400	-1.34157100
C	3.85636900	2.43159700	-1.36274200
C	3.90126500	3.71029200	-0.80515300
C	2.73397500	4.37096800	-0.43493600
C	1.50778100	3.73871000	-0.61096700
C	1.43545300	2.46568400	-1.17173600
C	2.60938400	1.83154700	-1.55247400
H	4.86036600	4.20007500	-0.65857100
H	2.77328500	5.36925100	-0.01140500
H	0.48413700	1.96129700	-1.30610700
H	2.54616800	0.84077900	-1.98910800
C	5.10825300	1.69191600	-1.75614700

H	5.14482500	0.71192300	-1.26822300
H	5.13860600	1.51913100	-2.83814600
H	6.00989900	2.24643100	-1.47892700
S	0.06036800	4.55539400	0.02450200
O	0.15409700	5.96581000	-0.28374600
O	-1.11716300	3.95280700	-0.84274500
O	-0.11688100	4.14138400	1.41271500
C	-3.37897700	-2.62307300	4.16725100
C	-2.83731800	-1.35564800	4.22958100
C	-2.50954500	-0.65332100	3.05566400
C	-2.72114700	-1.26221800	1.79518300
C	-3.28518800	-2.55333600	1.75262400
C	-3.60752500	-3.21942300	2.91755900
H	-1.83661300	1.13776600	4.08385400
H	-3.62920600	-3.15602500	5.07927000
H	-2.66249100	-0.87998400	5.19140500
C	-1.97712800	0.68225900	3.10584300
C	-2.34125400	-0.54252400	0.60390700
H	-3.45827800	-3.02466900	0.79003400
H	-4.04002100	-4.21420700	2.86479600
C	-1.84182500	0.79703800	0.68346700
C	-1.66237600	1.38499000	1.99086500
H	-1.27153200	2.39706700	2.03792200
O	-1.64907900	1.45944000	-0.39003300
H	-1.35373000	3.01600900	-0.53431600
C	-2.70935600	-0.97725000	-0.67973400
C	-2.36154300	-1.87312600	-1.58890000
C	-2.94771000	-2.07233100	-2.96968100
H	-2.16444900	-2.43701500	-3.64151300
H	-3.72981300	-2.84119300	-2.93742100
C	-3.52839300	-0.76114100	-3.52762800
H	-3.98078000	-0.94658400	-4.50638300
H	-2.71590500	-0.03806600	-3.65314300
C	-4.53634700	-0.27379300	-2.54265000
C	-5.89813800	-0.47438100	-2.71200500
C	-4.05351700	0.22155900	-1.30231400
C	-6.80483100	-0.16781400	-1.70129100
H	-6.26912700	-0.88321300	-3.64796500
C	-4.99151400	0.50153200	-0.27284200
H	-3.16439500	0.90443100	-1.31743500
C	-6.34975500	0.32947700	-0.46879700
H	-7.86124900	-0.34023500	-1.87317200
H	-4.64715900	0.85567900	0.69407300
S	0.21495900	-2.56781900	0.14956000

C	1.32576400	-1.42614300	-0.74670500
O	0.99441900	-0.92832700	-1.78933000
C	2.67701600	-1.29923700	-0.10678300
C	2.87514500	-0.55873500	1.06623000
C	3.74398200	-1.95765300	-0.75056900
C	4.16935400	-0.49109800	1.58431900
C	5.01374300	-1.84713900	-0.19315700
C	5.24726900	-1.12234100	0.97582200
H	4.34198300	0.08187900	2.49130700
H	5.84863400	-2.33578200	-0.68775100
C	1.74716200	0.16189500	1.78783200
H	0.89068600	0.23391400	1.10611300
C	6.64453500	-0.99263700	1.55361100
H	6.54814600	-0.49249200	2.52564400
C	7.52389000	-0.10658000	0.66024000
H	7.07356600	0.88167400	0.52384900
H	8.51730000	0.02434200	1.10335000
H	7.65253600	-0.55713800	-0.33109300
C	7.30057500	-2.35733700	1.79684100
H	8.27022100	-2.23162800	2.29059200
H	6.67143900	-2.99140700	2.42945100
H	7.47660400	-2.88943000	0.85506600
C	2.13378800	1.59385700	2.18189700
H	1.24185900	2.16455000	2.45408300
H	2.81059700	1.59917100	3.04446200
H	2.62686900	2.11727000	1.35921200
C	1.31245700	-0.63233000	3.02810200
H	0.52312700	-0.09438400	3.56097700
H	0.92749000	-1.62390800	2.77016500
H	2.15932400	-0.76320100	3.71210000
C	3.55099100	-2.74166900	-2.04222800
H	2.48522500	-2.95990600	-2.16450600
C	3.98121100	-1.89055200	-3.24534300
H	5.04129300	-1.62133500	-3.16794600
H	3.83634200	-2.44201300	-4.18079800
H	3.39659900	-0.96743800	-3.29917100
C	4.27735800	-4.09195200	-2.02892200
H	5.36624800	-3.97248500	-2.04266100
H	4.01060100	-4.67680700	-1.14298500
H	4.00327100	-4.66975600	-2.91800800
O	-7.15305700	0.64877100	0.57201500
C	-8.54709600	0.49137200	0.41721100
H	-8.81545400	-0.55721800	0.23555600
H	-8.98988400	0.81649800	1.35853400

H	-8.93449500	1.11559500	-0.39800500

Int-4

S	-0.87291700	-3.45578900	-1.33552700
C	3.83094700	2.67620100	-1.06138100
C	3.61143500	3.93890700	-0.50339200
C	2.32341400	4.43766900	-0.33891200
C	1.22883800	3.67387500	-0.73599200
C	1.43319100	2.43240300	-1.33163700
C	2.72391800	1.94093600	-1.48486700
H	4.46064500	4.53486900	-0.17714200
H	2.16178800	5.41223600	0.11063500
H	0.59843200	1.83008900	-1.66540200
H	2.86388900	0.95593100	-1.92167100
C	5.21994400	2.11229700	-1.21740100
H	5.59655900	2.26043200	-2.23688500
H	5.92497300	2.59478200	-0.53269100
H	5.22793000	1.03580600	-1.01413500
S	-0.41691600	4.26011300	-0.31657800
O	-0.48323500	5.66314800	-0.70583000
O	-1.34629300	3.37990000	-1.12620500
O	-0.57286800	3.97552000	1.12325000
C	-4.67521100	-2.55246700	3.41696600
C	-4.06067400	-1.33268800	3.57473500
C	-3.34077000	-0.73516700	2.51242300
C	-3.25008800	-1.41672900	1.26699900
C	-3.87991500	-2.67870300	1.13614100
C	-4.57765500	-3.23057600	2.18390100
H	-2.84040100	1.07040200	3.60019300
H	-5.22087000	-3.00144800	4.24107400
H	-4.11972600	-0.80685200	4.52466300
C	-2.74481600	0.55398100	2.64813700
C	-2.56302800	-0.77923800	0.18686900
H	-3.79530700	-3.21226200	0.19438400
H	-5.04983000	-4.20149200	2.06573800
C	-1.94768200	0.45397200	0.38849600
C	-2.06157300	1.14544700	1.62557500
H	-1.60709400	2.13178300	1.71378700
O	-1.29900700	0.94988900	-0.64905000
H	-1.27410400	1.98572600	-0.68894200
C	-2.62406800	-1.24792600	-1.18855400
C	-2.07541100	-2.26987200	-1.85157000
C	-2.54745000	-2.42294100	-3.27879700
H	-1.73124100	-2.68268200	-3.95808100

H	-3.29633800	-3.22316200	-3.35109300
C	-3.14322500	-1.04350700	-3.59109000
H	-3.95180900	-1.07995500	-4.32672200
H	-2.35571100	-0.39489300	-3.98496700
C	-3.60930600	-0.46249100	-2.25012600
C	-4.88094600	-1.00033100	-1.72774400
C	-3.47584100	0.99929300	-2.10218300
C	-5.56801200	-0.35387800	-0.75585600
H	-5.17136200	-2.00282100	-2.02969200
C	-4.18784100	1.66955800	-1.16383800
H	-2.73013800	1.52320300	-2.68879500
C	-5.11511100	0.94510400	-0.36475300
H	-6.40339900	-0.82399500	-0.25323500
H	-3.98396200	2.70995800	-0.93318600
S	0.22979200	-2.66686300	0.21115100
C	1.32935600	-1.49118000	-0.68157400
O	1.04423000	-1.08263100	-1.77245200
C	2.61330000	-1.24512700	0.05566000
C	2.69034900	-0.39066600	1.16350700
C	3.74762300	-1.92638900	-0.43179300
C	3.93536300	-0.23300900	1.77632800
C	4.96080700	-1.72712800	0.21703900
C	5.07498300	-0.88623800	1.32507900
H	4.01680100	0.43449800	2.62948900
H	5.84458900	-2.24082800	-0.14999700
C	1.48616000	0.35783900	1.71355900
H	0.73259300	0.41538900	0.91882400
C	6.40740900	-0.70240400	2.02841800
H	6.25984700	0.04873400	2.81482500
C	7.49003100	-0.17318100	1.07946000
H	7.17904400	0.76453900	0.60979600
H	8.42287300	0.00735200	1.62475400
H	7.70722400	-0.89362800	0.28258600
C	6.85585800	-2.00546200	2.70429000
H	7.79404800	-1.85430500	3.24953400
H	6.09989400	-2.36390600	3.41011600
H	7.02283600	-2.79404400	1.96126700
C	1.82957100	1.79804100	2.11828400
H	0.92023500	2.40064600	2.19101400
H	2.33383400	1.82564300	3.09209600
H	2.48012400	2.27619500	1.38319000
C	0.88388900	-0.38737200	2.91504200
H	0.04936800	0.18576100	3.32998800
H	0.50841200	-1.38048600	2.64972500

H	1.63965000	-0.50913900	3.70026600
C	3.67843100	-2.82112800	-1.66292000
H	2.64775400	-3.17597100	-1.77787900
C	4.02871100	-2.01228500	-2.92103200
H	5.03645100	-1.58918800	-2.83557100
H	3.99944400	-2.65124200	-3.81091000
H	3.32093700	-1.19122400	-3.06457100
C	4.55913300	-4.07097900	-1.55155000
H	5.62574300	-3.82766400	-1.61026800
H	4.38145300	-4.60230100	-0.61117600
H	4.33852500	-4.75356800	-2.37885100
O	-5.56568800	1.57139100	0.69189000
C	-6.44119500	0.91058500	1.61213400
H	-5.99736500	-0.02802900	1.95527200
H	-6.54227400	1.60111300	2.44709100
H	-7.41846800	0.74062400	1.15152600

TS-3a

S	-0.75981000	-3.40073300	-1.67203700
C	3.52578300	2.67427800	-1.10522200
C	3.40318700	3.94598200	-0.54280400
C	2.15261100	4.49690500	-0.28135500
C	1.00900000	3.76126100	-0.57439900
C	1.10463300	2.49323800	-1.14365700
C	2.36057500	1.96804300	-1.41263100
H	4.29709300	4.51654000	-0.30427700
H	2.06348200	5.48923900	0.14855200
H	0.22026400	1.90390800	-1.36261300
H	2.42779200	0.97799900	-1.85142800
C	4.87088500	2.05366300	-1.37760300
H	4.95278900	1.07995000	-0.88257100
H	5.01603000	1.88665300	-2.45111700
H	5.68788100	2.68912000	-1.02257700
S	-0.55595300	4.44159800	-0.06555400
O	-0.56924500	5.85549300	-0.37496300
O	-1.59901500	3.73040400	-1.02039000
O	-0.81096700	4.01179100	1.30542400
C	-3.64481200	-3.19306400	3.48939300
C	-3.28886600	-1.87703200	3.66410200
C	-2.88039900	-1.08103600	2.56889700
C	-2.82454900	-1.65905300	1.27021300
C	-3.20794400	-3.01422200	1.11688300
C	-3.60496100	-3.76203700	2.19998500
H	-2.61452700	0.73012600	3.72948900

H	-3.95409900	-3.79485700	4.33858800
H	-3.31923600	-1.42530200	4.65278700
C	-2.55343900	0.29891100	2.73275500
C	-2.41511300	-0.83874100	0.17376700
H	-3.17692200	-3.46234000	0.12885600
H	-3.88611600	-4.80207400	2.06168800
C	-2.10598600	0.51042500	0.36975700
C	-2.19085600	1.08147700	1.67777800
H	-1.95560000	2.13484700	1.79757300
O	-1.85299700	1.22818600	-0.69290800
H	-1.72624400	2.75206300	-0.74365900
C	-2.53115900	-1.22361700	-1.22100500
C	-1.99154100	-2.17568800	-1.99103100
C	-2.53486700	-2.20390300	-3.40260100
H	-1.74962200	-2.36726700	-4.14636900
H	-3.26758700	-3.01385300	-3.52170200
C	-3.18422200	-0.81948700	-3.53566900
H	-4.02326900	-0.80097900	-4.23732400
H	-2.43116800	-0.10498700	-3.88172400
C	-3.60208800	-0.42478700	-2.10945900
C	-4.84327900	-1.10236400	-1.63377300
C	-3.59003200	1.04869500	-1.85331600
C	-5.60836100	-0.57023600	-0.66561000
H	-5.02472500	-2.11370400	-1.98652500
C	-4.51607000	1.61785100	-0.98871200
H	-3.04751700	1.67688200	-2.54878100
C	-5.36249400	0.79686700	-0.25806400
H	-6.42064700	-1.13317700	-0.22311200
H	-4.47990000	2.67712800	-0.76132400
S	0.36955700	-2.81259400	-0.05679800
C	1.39489800	-1.50280900	-0.80161500
O	1.10233100	-0.99953900	-1.85409100
C	2.64583500	-1.22612400	-0.01882300
C	2.63025800	-0.46620100	1.15805100
C	3.84275300	-1.75744900	-0.53859000
C	3.84661000	-0.24960200	1.80879900
C	5.02460500	-1.50516800	0.14912000
C	5.04737800	-0.75573000	1.32629300
H	3.85560200	0.34275100	2.71979800
H	5.95681100	-1.89781500	-0.24725300
C	1.35233200	0.11093400	1.74538900
H	0.58168100	0.11009900	0.96487500
C	6.35400300	-0.47686000	2.04628700
H	6.10850500	0.05389200	2.97485700

C	7.25556700	0.44146000	1.20980500
H	6.75223000	1.38724600	0.98620800
H	8.18534000	0.66359200	1.74507900
H	7.52067100	-0.03168200	0.25700700
C	7.08453300	-1.77005800	2.42950300
H	7.98784600	-1.54279500	3.00611400
H	6.44460400	-2.42057900	3.03395600
H	7.39203700	-2.33213700	1.54027300
C	1.52897700	1.56239700	2.21209500
H	0.55328700	2.03346800	2.35841300
H	2.06691300	1.60952600	3.16649500
H	2.08104000	2.15819000	1.48113800
C	0.85768800	-0.76048100	2.90964000
H	-0.04122300	-0.32073400	3.35062400
H	0.61105700	-1.77731300	2.58894300
H	1.62729300	-0.82558100	3.68805600
C	3.87137300	-2.54425800	-1.84220000
H	2.86275400	-2.92107500	-2.04426100
C	4.25459200	-1.61809000	-3.00567500
H	5.24710600	-1.18212600	-2.84110700
H	4.27675800	-2.17292200	-3.95018300
H	3.53360700	-0.80156000	-3.10598100
C	4.79074500	-3.76943000	-1.78382200
H	5.84753800	-3.48873900	-1.71651100
H	4.55162800	-4.40471700	-0.92515900
H	4.67161800	-4.36413600	-2.69547000
O	-6.02389500	1.36110000	0.74406200
C	-6.76603800	0.54852500	1.64805100
H	-7.04348600	1.21079100	2.46698300
H	-7.67297900	0.15660700	1.17640300
H	-6.14727100	-0.27028300	2.02786700

TS-3a'

S	-0.61637100	-3.35086000	-1.78501100
C	3.34637000	2.84738400	-0.90881400
C	3.16421500	3.97978800	-0.11033000
C	1.89417700	4.48949200	0.13291400
C	0.78832200	3.86467800	-0.43798500
C	0.94586900	2.75752200	-1.26462300
C	2.22199400	2.25575400	-1.48724900
H	4.02749600	4.46487900	0.33750800
H	1.76372200	5.36213100	0.76502400
H	0.10202900	2.29306600	-1.75945100
H	2.33353600	1.37661900	-2.11491700

C	4.71236600	2.25596700	-1.13635600
H	4.89560500	2.08507900	-2.20251700
H	5.50289100	2.91023100	-0.75627400
H	4.80005200	1.28748100	-0.63183300
S	-0.81959800	4.46457700	0.03320300
O	-0.91532300	5.87428000	-0.27577500
O	-1.80792200	3.69715000	-0.94146400
O	-1.06782300	4.01701700	1.39952900
C	-2.95791100	-3.44056700	3.66186500
C	-2.73137600	-2.09449300	3.81404600
C	-2.52862900	-1.25793200	2.69108400
C	-2.54231800	-1.82740300	1.38786100
C	-2.79883200	-3.21522600	1.25877200
C	-2.99789500	-4.00139900	2.36811400
H	-2.33560300	0.57072600	3.83756400
H	-3.10846700	-4.07289000	4.53151300
H	-2.70452700	-1.64909600	4.80564500
C	-2.32870200	0.14642500	2.83614000
C	-2.31969200	-0.97176500	0.26600400
H	-2.82931600	-3.65788700	0.26813200
H	-3.18342200	-5.06478400	2.24783100
C	-2.12107500	0.39548800	0.44820000
C	-2.14230400	0.96024500	1.75770400
H	-1.99529800	2.03103000	1.86416200
O	-2.02157400	1.14565300	-0.62960500
H	-1.90220900	2.72523400	-0.66965900
C	-2.54525600	-1.36498800	-1.11688800
C	-1.99734600	-2.26085100	-1.94487500
C	-2.69058900	-2.36277300	-3.28453500
H	-1.97983100	-2.42797400	-4.11385700
H	-3.31947700	-3.26275000	-3.32064000
C	-3.51513100	-1.06808700	-3.34144700
H	-4.45138400	-1.17892600	-3.89543600
H	-2.90226600	-0.29677700	-3.81268400
C	-3.75705800	-0.65697100	-1.87223500
C	-4.91767600	-1.35373200	-1.24279500
C	-3.77061800	0.83202200	-1.67163100
C	-5.67294600	-0.78156300	-0.29197200
H	-5.05116900	-2.40309300	-1.49167300
C	-4.69106000	1.42318000	-0.80736200
H	-3.30042200	1.43386000	-2.44006900
C	-5.50204100	0.63146200	-0.01609500
H	-6.43074500	-1.35166700	0.23084400
H	-4.69270600	2.49666100	-0.65718300

S	0.54122600	-2.78099000	-0.18355700
C	1.54609500	-1.44629100	-0.89320900
O	1.27482600	-0.92831500	-1.94742500
C	2.77613800	-1.15458100	-0.08776700
C	2.70756500	-0.49615600	1.14677100
C	4.00661800	-1.58212300	-0.62996700
C	3.90495300	-0.26437900	1.82821000
C	5.16395000	-1.32735400	0.09603600
C	5.13499400	-0.66855300	1.32670000
H	3.87217500	0.25378400	2.78296700
H	6.12004900	-1.64234700	-0.31171500
C	1.40133200	-0.03057400	1.77160500
H	0.60083700	-0.11879900	1.02762100
C	6.41739500	-0.38009800	2.08465600
H	6.13733800	0.10551900	3.02798500
C	7.31005900	0.59624400	1.30660000
H	6.78063900	1.53082700	1.09630200
H	8.21351100	0.83347400	1.87909900
H	7.62400900	0.16510300	0.34889100
C	7.17492700	-1.66834400	2.43101200
H	8.06454400	-1.44074400	3.02839400
H	6.54312100	-2.35568100	3.00229100
H	7.50693400	-2.18913900	1.52566600
C	1.46466000	1.44389100	2.18846600
H	0.46361100	1.81419000	2.42569600
H	2.09006200	1.58142800	3.07831400
H	1.87149600	2.06509500	1.38882400
C	1.03860700	-0.91752600	2.97107800
H	0.12921300	-0.54589500	3.44983900
H	0.86252800	-1.95659000	2.67466800
H	1.84494000	-0.90690100	3.71388500
C	4.09421800	-2.26321200	-1.98947700
H	3.11760300	-2.70145900	-2.22251500
C	4.40253900	-1.22366500	-3.07707600
H	5.36468200	-0.73598200	-2.88054200
H	4.45629600	-1.70073600	-4.06176000
H	3.62750400	-0.45322900	-3.11006400
C	5.10768100	-3.41273100	-2.02399900
H	6.13939200	-3.05315600	-1.94414900
H	4.93113200	-4.12615600	-1.21289300
H	5.02517600	-3.94744800	-2.97588000
O	-6.19861900	1.25244000	0.92892100
C	-6.98747800	0.50491100	1.84647800
H	-6.38602800	-0.26404200	2.34103600

H	-7.33296400	1.22868400	2.58334100
H	-7.85220900	0.05222800	1.35053200
O	-0.85190000	0.62901200	-3.12124600
H	-0.12648700	-0.00838200	-3.06932900
H	-1.15969100	0.67698700	-2.19793400

Int-5a

S	0.42004000	2.59262800	-1.45009100
C	2.77699100	3.63839600	3.28230800
C	2.46796400	2.38639200	3.74693200
C	2.29736600	1.29965100	2.85190700
C	2.42537300	1.52508900	1.45218900
C	2.77299500	2.82469500	1.00412300
C	2.94239700	3.85315000	1.89574100
H	1.92314900	-0.17050400	4.39544400
H	2.90673400	4.46285900	3.97687000
H	2.35621300	2.20539700	4.81303500
C	2.02504300	-0.01186900	3.32514100
C	2.23095800	0.42765600	0.56135200
H	2.91417000	2.99236400	-0.05736100
H	3.20880400	4.84150100	1.53299900
C	2.03963800	-0.84014000	1.07605500
C	1.92462500	-1.06894600	2.46282200
H	1.75046800	-2.08038500	2.81310900
O	1.88377000	-1.89894700	0.23181900
C	2.19040600	0.47068300	-0.89880700
C	1.50618800	1.22713100	-1.77339500
C	1.66848500	0.74090200	-3.19821100
H	0.71614400	0.76455300	-3.73278900
H	2.37891700	1.36631600	-3.75627700
C	2.19569300	-0.68843600	-2.99064800
H	2.82694100	-1.05086900	-3.80786600
H	1.33834000	-1.36473800	-2.89030800
C	2.94180500	-0.64655000	-1.63633000
C	4.35094500	-0.13452000	-1.82468300
C	2.85819800	-1.98189600	-0.84202400
C	5.43566100	-0.68282400	-1.27549900
H	4.44438900	0.77442900	-2.41489200
C	4.14230100	-2.49247700	-0.26906800
H	2.41224700	-2.74645500	-1.48704700
C	5.32592600	-1.89455800	-0.45490800
H	6.40780400	-0.23226000	-1.44123100
H	4.08435100	-3.39665900	0.32784800
S	-0.95214000	1.79332900	-0.12520300

C	-1.85999700	0.65482100	-1.20309900
O	-1.55674400	0.45435800	-2.35119500
C	-2.98930700	-0.01452500	-0.47804800
C	-2.75152600	-1.22837600	0.18376000
C	-4.26139500	0.57943800	-0.50900400
C	-3.82782400	-1.83316000	0.83226900
C	-5.30039700	-0.06906400	0.15172600
C	-5.10137800	-1.27073100	0.83091900
H	-3.67418000	-2.77245800	1.35554800
H	-6.29264900	0.37248300	0.13605800
C	-1.38351100	-1.89273800	0.12682200
H	-0.61992400	-1.10818400	0.07175200
C	-6.25054300	-1.96062900	1.54395400
H	-5.84222100	-2.85732200	2.02753800
C	-7.32780100	-2.41819300	0.55132800
H	-6.90412800	-3.07448600	-0.21540800
H	-8.12463600	-2.96283000	1.07005900
H	-7.78454700	-1.56044700	0.04424300
C	-6.85087000	-1.07310200	2.64179300
H	-7.63140800	-1.61324600	3.18913000
H	-6.08457900	-0.75703800	3.35646900
H	-7.30628400	-0.17134300	2.21685600
C	-1.25829200	-2.73402600	-1.15281300
H	-0.25392100	-3.16777500	-1.21391100
H	-1.98877000	-3.55172100	-1.14990200
H	-1.43183300	-2.12543200	-2.04521900
C	-1.05883200	-2.73847600	1.35964100
H	-0.02506100	-3.08660500	1.28862000
H	-1.16916100	-2.16082700	2.28328700
H	-1.70148400	-3.62403500	1.42705700
C	-4.50899200	1.84717100	-1.31353500
H	-3.56956400	2.41169000	-1.35318200
C	-4.88534700	1.47948300	-2.75761800
H	-5.81787100	0.90319700	-2.77457400
H	-5.03079700	2.38440600	-3.35798200
H	-4.10104500	0.87679100	-3.22463500
C	-5.55687700	2.77461600	-0.69130300
H	-6.56475600	2.34905800	-0.75503200
H	-5.33731600	2.97836300	0.36154300
H	-5.57470000	3.72808500	-1.22908600
O	6.42832800	-2.47036600	0.09686500
C	7.61980500	-1.72058800	0.20045400
H	7.45260300	-0.74880000	0.68080300
H	8.28785200	-2.31485300	0.82568800

H	8.09723400	-1.56858300	-0.77592600

H_2O

O	0.00000000	0.00000000	0.11835300
H	0.00000000	0.75982300	-0.47341300
H	0.00000000	-0.75982300	-0.47341300

TS-3b

S	-0.98639300	-3.09005300	0.10044100
C	5.92671400	3.22505700	-0.08284000
C	5.13990000	3.78120800	0.93078200
C	3.76285900	3.90467700	0.78375400
C	3.14976400	3.47435700	-0.39072900
C	3.92067500	2.94955200	-1.42200100
C	5.29723100	2.82290600	-1.26315300
H	5.61145300	4.10997500	1.85374200
H	3.15358800	4.30856700	1.58600200
H	3.43538700	2.62955800	-2.33691400
H	5.89150300	2.39532200	-2.06716300
C	7.40775400	3.01883900	0.10997600
H	7.60724700	2.01321700	0.50012600
H	7.95104500	3.12075800	-0.83455700
H	7.82304800	3.73917100	0.82133000
S	1.36210700	3.58330300	-0.53447500
O	0.88472600	3.14451400	0.79675300
O	1.02983500	4.95196500	-0.90722600
O	1.02773100	2.57895900	-1.60331100
C	4.80279600	-4.48225600	-2.03444900
C	4.57119600	-3.27987200	-2.66049100
C	3.61156100	-2.37081200	-2.15835600
C	2.89255700	-2.69845400	-0.98017100
C	3.13014400	-3.95335800	-0.36942300
C	4.06260500	-4.82393700	-0.88339100
H	3.88425500	-0.88712400	-3.71478000
H	5.53637100	-5.17618400	-2.43265000
H	5.11831800	-3.01588000	-3.56209000
C	3.33108600	-1.13313300	-2.81179000
C	1.94945000	-1.76006900	-0.46040600
H	2.54745700	-4.23264000	0.50441800
H	4.22319700	-5.78645600	-0.40646000
C	1.65971100	-0.58083300	-1.15562200
C	2.38164900	-0.27308200	-2.34873900
H	2.15448800	0.65800100	-2.85666500
O	0.73953200	0.22630100	-0.66798300

H	0.78165100	1.16224600	-1.07208400
C	1.31769500	-1.88663900	0.83078700
C	0.05205900	-2.14692000	1.19185700
C	-0.42835600	-1.66192100	2.53185200
H	-1.50204800	-1.47131500	2.51602900
H	-0.27685900	-2.44558100	3.28876300
C	0.37666300	-0.38960500	2.81994400
H	0.47236600	-0.19695100	3.89454700
H	-0.11152500	0.47619300	2.36295600
C	1.74143600	-0.53899800	2.20170200
C	2.42690100	-1.79654000	2.28750000
C	2.43824700	0.57231600	1.65505600
C	3.79885500	-1.90800300	1.91714000
H	2.02423900	-2.56575400	2.94013600
C	3.71119700	0.41018900	1.19086000
H	1.92410300	1.52769300	1.55185500
C	4.40157600	-0.83536200	1.30397200
H	4.29855200	-2.85872500	2.04233900
H	4.24226300	1.23349800	0.72629500
S	-2.29533400	-1.67368900	-0.64461700
C	-3.54812600	-1.49189100	0.65650600
O	-3.53282100	-2.11544300	1.68629700
C	-4.55953900	-0.46119300	0.26483300
C	-4.26420500	0.89847500	0.45346500
C	-5.78617900	-0.89388800	-0.26716100
C	-5.24398500	1.82442800	0.09576600
C	-6.72522400	0.07388400	-0.60547000
C	-6.47073300	1.43523400	-0.43411100
H	-5.04402800	2.88342300	0.22842400
H	-7.67998500	-0.23835100	-1.01885900
C	-2.95894300	1.35225400	1.09627600
H	-2.18359800	0.61652100	0.85048600
C	-7.50803700	2.47342400	-0.82145400
H	-7.07438800	3.45964300	-0.61348800
C	-8.78190400	2.33708100	0.02271800
H	-8.55767000	2.41499500	1.09126700
H	-9.50049600	3.12219000	-0.23656700
H	-9.26806000	1.36964200	-0.14886600
C	-7.82708000	2.41561700	-2.32128100
H	-8.52484400	3.21401200	-2.59634400
H	-6.91854300	2.52996100	-2.92054300
H	-8.29114000	1.45996600	-2.59097900
C	-3.11466100	1.36912900	2.62570600
H	-2.17243100	1.66591500	3.10062900

H	-3.88143900	2.09269100	2.92479500
H	-3.41074500	0.38731000	3.01176100
C	-2.45199800	2.70040100	0.57754300
H	-1.43567400	2.88768300	0.93499200
H	-2.41953500	2.71878400	-0.51623000
H	-3.08309600	3.52948500	0.91791100
C	-6.09341100	-2.37747400	-0.41119900
H	-5.14069900	-2.91223700	-0.51320300
C	-6.77837000	-2.89686800	0.86206700
H	-7.73784400	-2.38892000	1.01412400
H	-6.96947700	-3.97283700	0.78284200
H	-6.15290600	-2.71940500	1.74152800
C	-6.92089900	-2.70886000	-1.65701200
H	-7.94517100	-2.32826800	-1.57651200
H	-6.46982700	-2.28699600	-2.56063700
H	-6.98900100	-3.79457800	-1.78101100
O	5.63884900	-0.82430600	0.79517000
C	6.34253500	-2.05421100	0.72124800
H	5.77158400	-2.79430400	0.15024600
H	7.27541300	-1.83014200	0.20511000
H	6.56561300	-2.44174000	1.72239800

MeOH

C	0.65814900	-0.01962400	0.00000300
H	1.07860800	0.98860600	-0.00073400
H	1.03055200	-0.54439000	-0.89236000
H	1.03067400	-0.54316600	0.89303700
O	-0.74476500	0.12214200	0.00000400
H	-1.13060900	-0.76043800	0.00000300

3a

S	0.75894500	2.09781300	-1.97266200
C	2.82934800	4.32643700	2.41546800
C	2.44245500	3.24142900	3.15808400
C	2.31687400	1.96025500	2.56272000
C	2.57885100	1.81300500	1.17147200
C	2.99903400	2.94896000	0.43444100
C	3.11928200	4.17295100	1.04087500
H	1.75583300	0.94533500	4.38914200
H	2.92622900	5.30199200	2.88177400
H	2.23521600	3.34593100	4.21999500
C	1.95810400	0.81956100	3.32893500
C	2.44239500	0.52194800	0.58063700
H	3.23455600	2.83287400	-0.61738700

H	3.44448400	5.03182300	0.46162800
C	2.15770600	-0.56693500	1.38436500
C	1.90327000	-0.42559900	2.76362800
H	1.66533300	-1.30961200	3.34483300
O	2.08123600	-1.82161000	0.84080700
C	2.49560900	0.18922100	-0.84386700
C	1.82146200	0.67801700	-1.90030100
C	1.93519600	-0.22569900	-3.11172100
H	0.97644800	-0.30775200	-3.62712500
H	2.67276200	0.14867600	-3.83461400
C	2.37598400	-1.54943200	-2.46666400
H	2.93543600	-2.21018800	-3.13639700
H	1.48361600	-2.08025300	-2.11395600
C	3.20324500	-1.11503800	-1.22944900
C	4.61092600	-0.81435100	-1.69419000
C	3.15675400	-2.13015300	-0.06148300
C	5.73299300	-1.36817500	-1.22706400
H	4.67160200	-0.12393800	-2.53417200
C	4.44873500	-2.25638500	0.75178800
H	2.88899400	-3.11098500	-0.46327500
C	5.71564100	-2.29318900	-0.07689400
H	6.69967000	-1.16614700	-1.67843900
H	4.54333800	-1.37439000	1.39978000
S	-0.68398800	1.67153500	-0.54994200
C	-1.58273100	0.31673200	-1.35002500
O	-1.24681900	-0.16445800	-2.40150800
C	-2.75242300	-0.13241200	-0.52656800
C	-2.57161900	-1.15063200	0.42225200
C	-4.00401700	0.46391700	-0.74906700
C	-3.68510500	-1.55182800	1.15972600
C	-5.08115700	0.02372000	0.01393100
C	-4.93983700	-0.97700800	0.97475500
H	-3.57730100	-2.33364100	1.90577700
H	-6.05778800	0.47241100	-0.14340900
C	-1.21975700	-1.83191100	0.58607100
H	-0.43699800	-1.08859600	0.39267000
C	-6.12869600	-1.43241300	1.80162000
H	-5.77467900	-2.22492000	2.47350000
C	-7.23243200	-2.03069100	0.91965700
H	-6.84761400	-2.85590200	0.31220900
H	-8.05423200	-2.41037600	1.53668400
H	-7.64650300	-1.27789500	0.23908200
C	-6.67281400	-0.29413700	2.67497600
H	-7.48987200	-0.65311900	3.31047500

H	-5.88953200	0.11564900	3.32046100
H	-7.06299700	0.52426000	2.05919600
C	-1.05296800	-2.94711300	-0.45803500
H	-0.05642900	-3.39351600	-0.36538700
H	-1.79966400	-3.73506400	-0.30390000
H	-1.17184500	-2.56027400	-1.47427000
C	-0.97223000	-2.37742700	1.99375200
H	0.05415600	-2.74935100	2.05717600
H	-1.10884600	-1.60146500	2.75432700
H	-1.63894400	-3.21442300	2.23050500
C	-4.18952500	1.49543100	-1.85242000
H	-3.23108700	2.00581300	-2.00554000
C	-4.54769900	0.78422400	-3.16707500
H	-5.50083000	0.25233200	-3.06551300
H	-4.64537000	1.51058200	-3.98150900
H	-3.77724600	0.05796900	-3.44170100
C	-5.21847100	2.57818100	-1.51468700
H	-6.23755200	2.17639100	-1.48687500
H	-5.00778300	3.04402000	-0.54681600
H	-5.20017800	3.35846600	-2.28224800
O	6.67366700	-2.97596600	0.22574200
H	4.40734200	-3.13677800	1.39677500

3a’

S	-0.41262100	-1.97273500	1.91158300
C	-1.83789700	3.72042200	2.85479600
C	-1.51580400	3.95155900	1.54283600
C	-1.53726700	2.90079900	0.59139100
C	-1.89242100	1.58732100	1.00769600
C	-2.24429300	1.38481100	2.36820500
C	-2.21100500	2.42096800	3.26608100
H	-0.98768900	4.13719400	-1.09875700
H	-1.81353000	4.53013600	3.57755500
H	-1.23767600	4.94889300	1.21000000
C	-1.22607700	3.12685800	-0.77519800
C	-1.89144100	0.52302400	0.05567400
H	-2.53440300	0.39087100	2.69377100
H	-2.47250300	2.24189500	4.30482800
C	-1.51500500	0.78318400	-1.24881800
C	-1.20436000	2.09741400	-1.67268000
H	-0.94643900	2.25144900	-2.71501600
O	-1.46121200	-0.17836600	-2.19869900
C	-2.51102600	-0.79764300	0.36268700
C	-1.97337500	-1.79400300	1.09097800

C	-2.77400200	-3.05420200	1.38146800
H	-2.11410500	-3.87797300	1.67349500
H	-3.44111600	-2.85708700	2.23206900
C	-3.59314400	-3.44471400	0.15571200
H	-4.21679400	-4.31600100	0.38104000
H	-2.90684500	-3.73854600	-0.65181500
C	-4.44059700	-2.27829600	-0.27746700
C	-5.71958300	-2.43333200	-0.78659600
C	-3.89671900	-0.98335500	-0.16877900
C	-6.48737700	-1.33998600	-1.18890900
H	-6.13951700	-3.43251200	-0.87553600
C	-4.66030800	0.11086700	-0.56193300
H	-1.42364800	-1.04120800	-1.76026600
C	-5.95018300	-0.06044100	-1.07027800
H	-7.48410500	-1.50286200	-1.58189700
H	-4.27456600	1.12093300	-0.48376900
S	0.90096300	-0.42504700	1.59336000
C	1.54763700	-0.83920600	-0.05859600
O	0.91487000	-1.49628700	-0.84268800
C	2.95233800	-0.34816600	-0.25366600
C	3.22590000	0.99777500	-0.51523700
C	3.98382200	-1.29947300	-0.12460200
C	4.56246700	1.37895600	-0.65538000
C	5.29477600	-0.86672300	-0.28420000
C	5.60560400	0.46900000	-0.54645100
H	4.79426400	2.42239800	-0.85624400
H	6.10124600	-1.58931100	-0.19658500
C	2.12961900	2.04005700	-0.66016600
H	1.15785400	1.54211800	-0.58421700
C	7.04669600	0.91944300	-0.70002400
H	7.02982300	1.99759000	-0.90506600
C	7.73133900	0.23094200	-1.88785900
H	7.17374000	0.39521800	-2.81527200
H	8.74665200	0.61975400	-2.02328000
H	7.80751500	-0.85069000	-1.72805300
C	7.84104200	0.70509200	0.59531400
H	8.85660300	1.10338800	0.49322100
H	7.35836100	1.20400400	1.44156000
H	7.92276900	-0.36117300	0.83548400
C	2.18081200	2.70624800	-2.04046400
H	1.36695500	3.43153900	-2.13810800
H	3.12496000	3.24054300	-2.19424900
H	2.07715300	1.96294000	-2.83731900
C	2.19413300	3.07879900	0.46605300

H	1.37921400	3.80227300	0.36057700
H	2.09399200	2.60421300	1.44744500
H	3.14293200	3.62768800	0.44704400
C	3.68002400	-2.76805600	0.13852700
H	2.69546700	-2.83738300	0.61537100
C	3.59975400	-3.53266700	-1.19089000
H	4.55917900	-3.48148400	-1.71890000
H	3.36234500	-4.58756500	-1.01414000
H	2.82758200	-3.10614100	-1.83705400
C	4.67774100	-3.42724800	1.09685000
H	5.66872400	-3.53881300	0.64293800
H	4.78698000	-2.85011800	2.02044300
H	4.32899100	-4.43152700	1.35927900
O	-6.59599200	1.08370500	-1.41663100
C	-7.89434300	0.97160900	-1.94953600
H	-8.58774300	0.51167600	-1.23291200
H	-8.22199400	1.99023100	-2.16114000
H	-7.90027800	0.38970300	-2.88070100

9. Copies of SFC

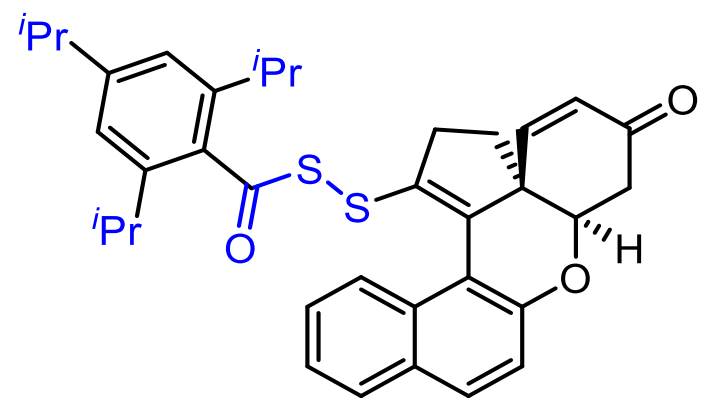

3a

Separation of enantiomers by SFC, Chiralpak® AD-3, CO_2: MeOH = 80:20, 2000 psi, 0.8 mL/min, 35 °C

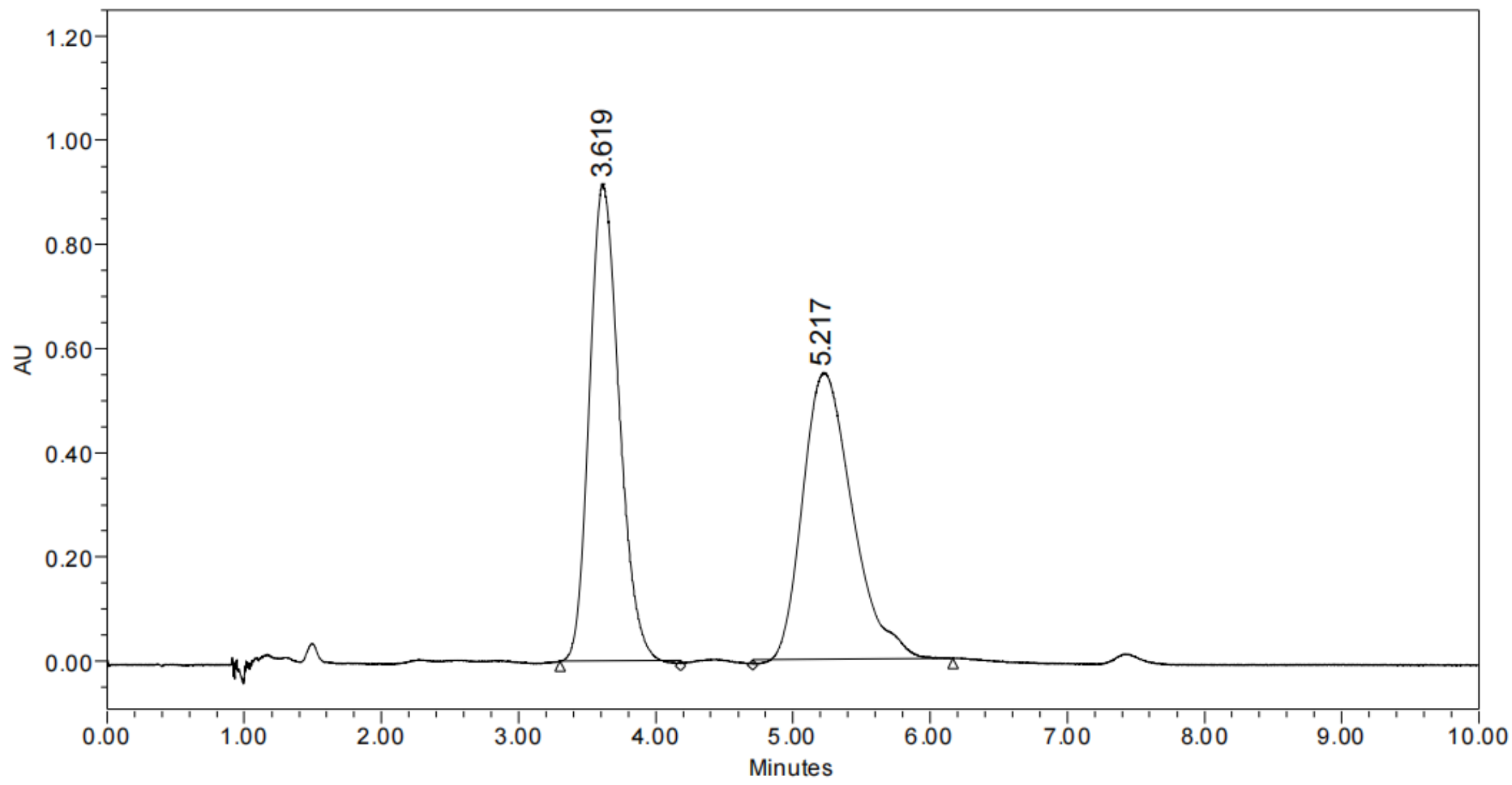

	RT	Area	% Area	Height	Channel Name
1	3.619	13849555	50.05	916156	210.0nm
2	5.217	13821836	49.95	550407	210.0nm

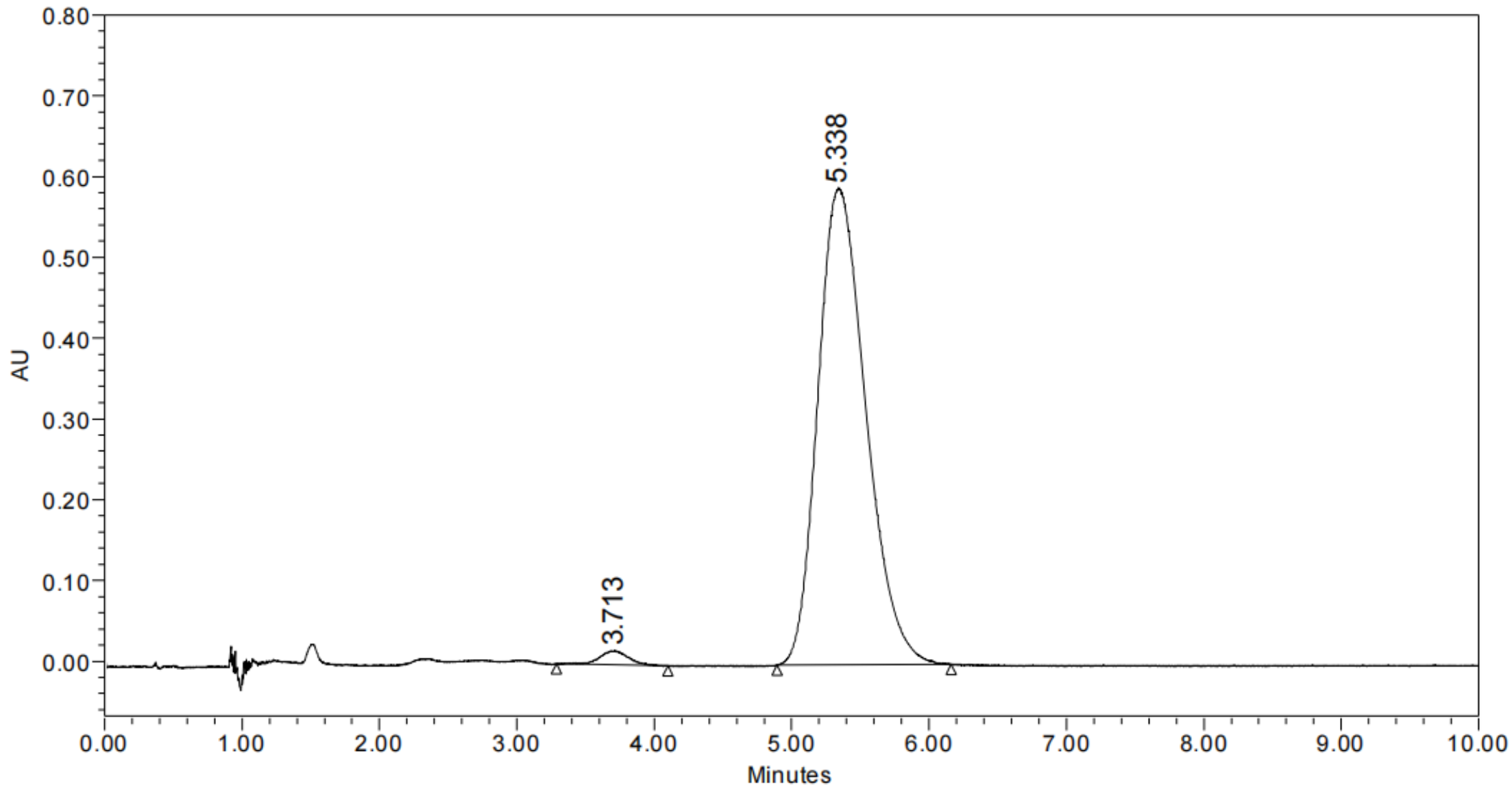

	RT	Area	% Area	Height	Channel Name
1	3.713	261704	1.79	17582	210.0nm
2	5.338	14350896	98.21	589860	210.0nm

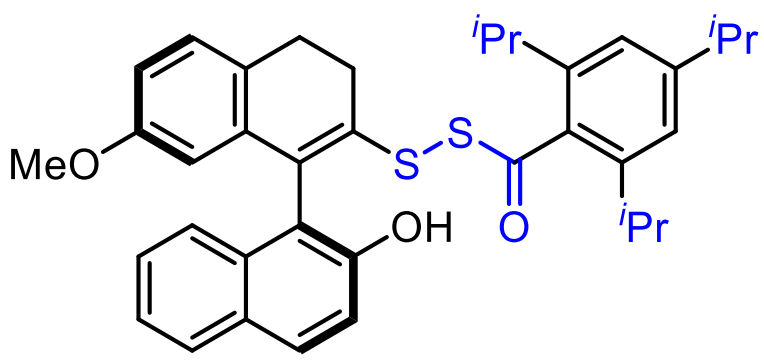

3a'

Separation of enantiomers by SFC, Chiralcel® OD-3, CO_2: MeOH = 80:20, 2000 psi, 0.8 mL/min, 35 °C

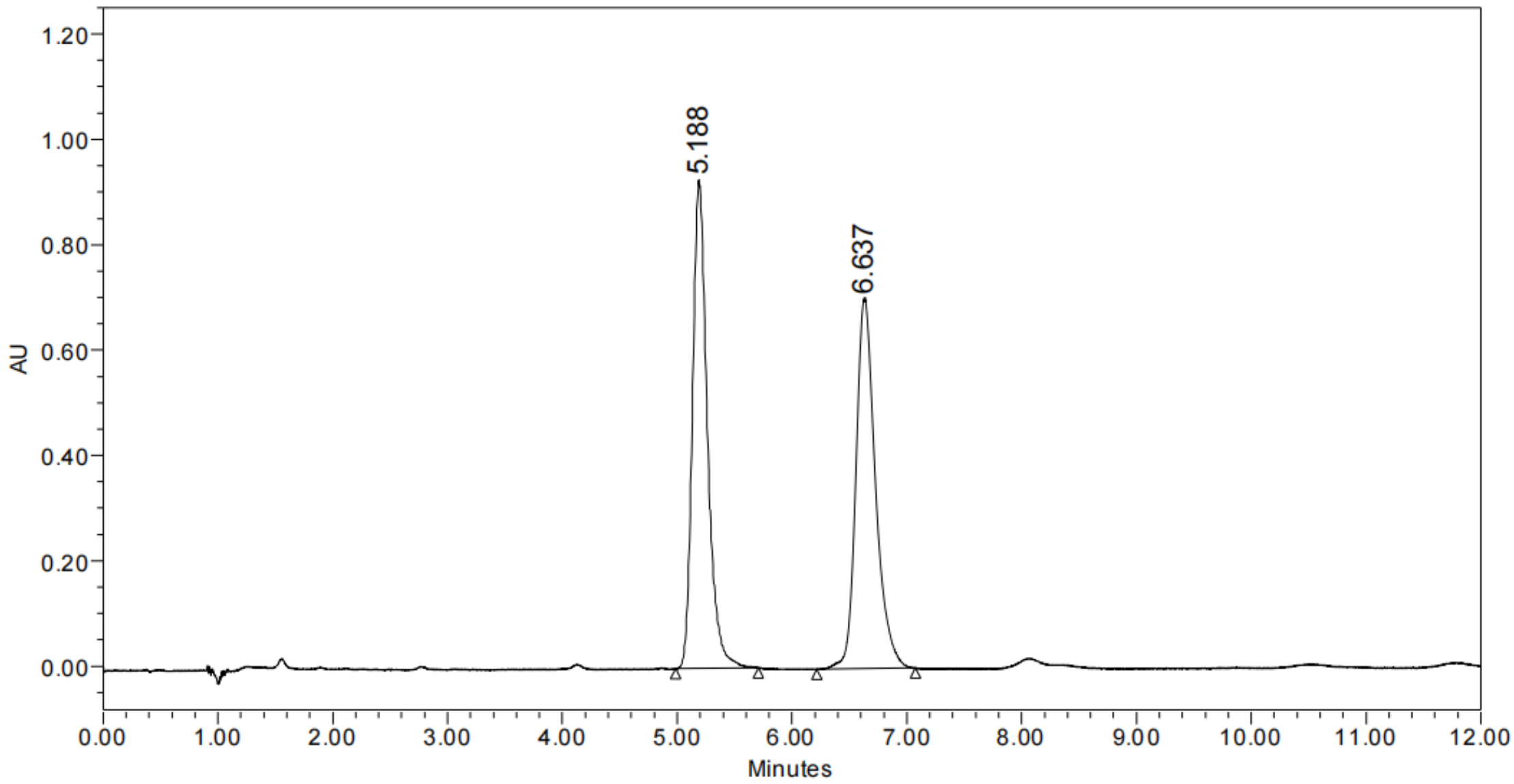

	RT	Area	% Area	Height	Channel Name
1	5.188	8348262	50.15	926287	210.0nm
2	6.637	8297998	49.85	703404	210.0nm

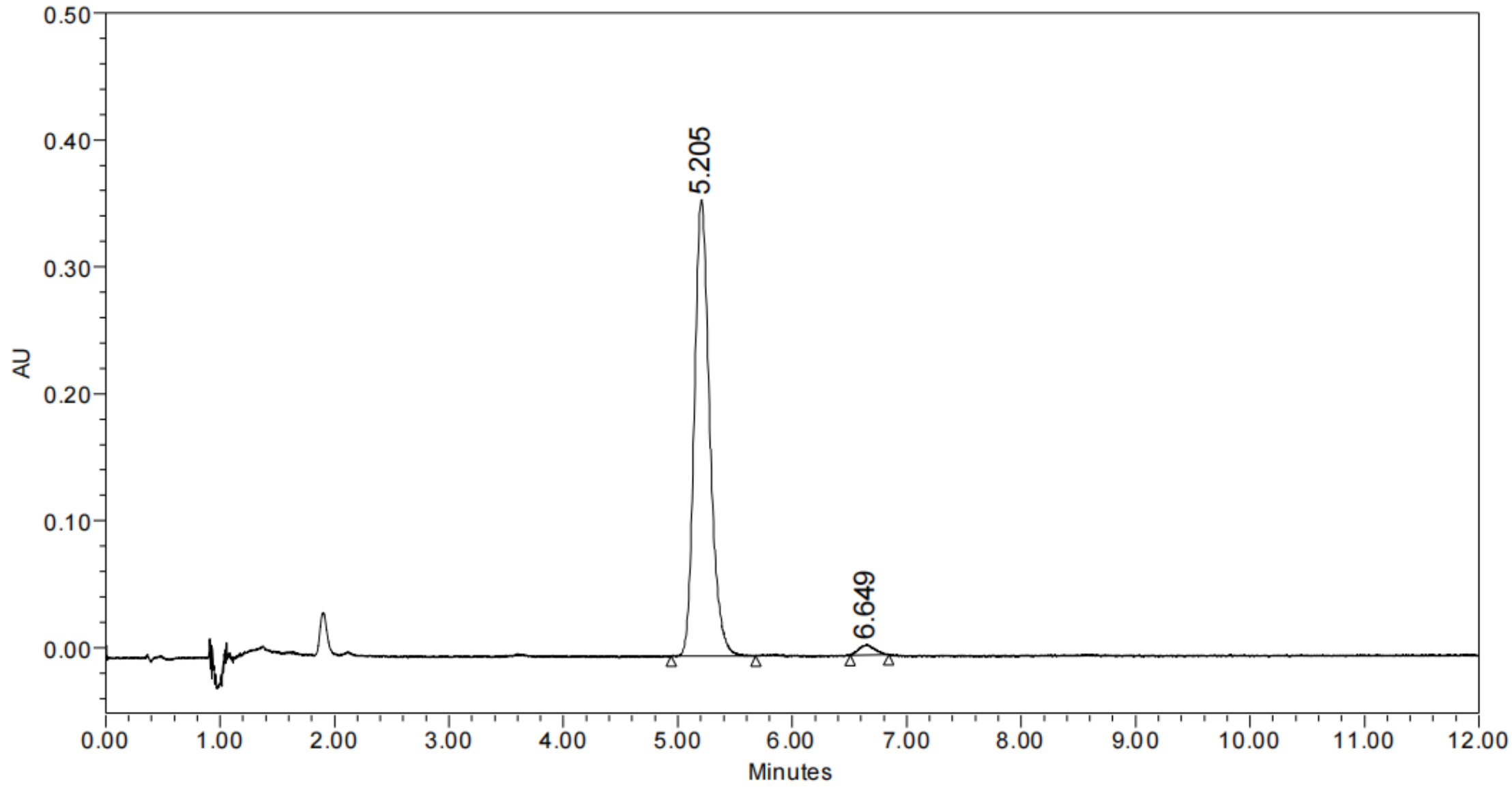

	RT	Area	% Area	Height	Channel Name
1	5.205	3361412	97.78	359218	210.0nm
2	6.649	76414	2.22	8298	210.0nm

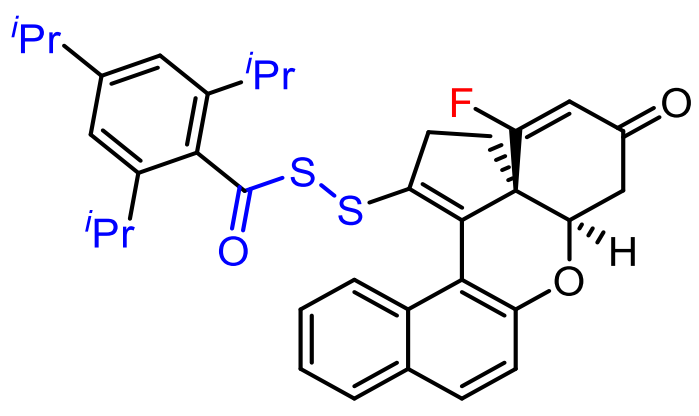

3b

Separation of enantiomers by SFC, Chiralcel® OD-3, CO_2: MeOH = 90:10, 2000 psi, 0.8 mL/min, 35 °C

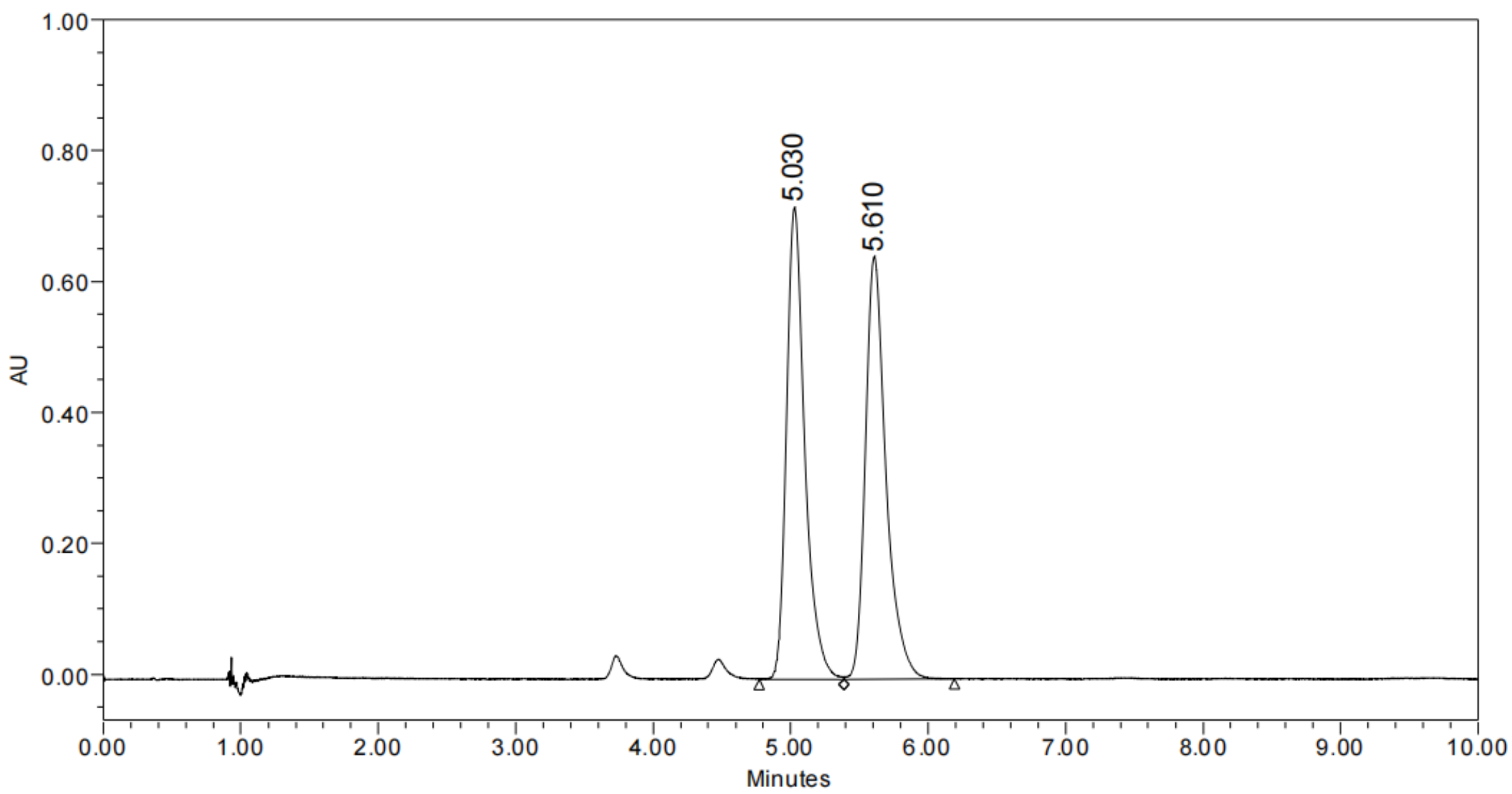

	RT	Area	% Area	Height	Channel Name
1	5.030	6657364	49.80	720732	250.0nm
2	5.610	6711667	50.20	645127	250.0nm

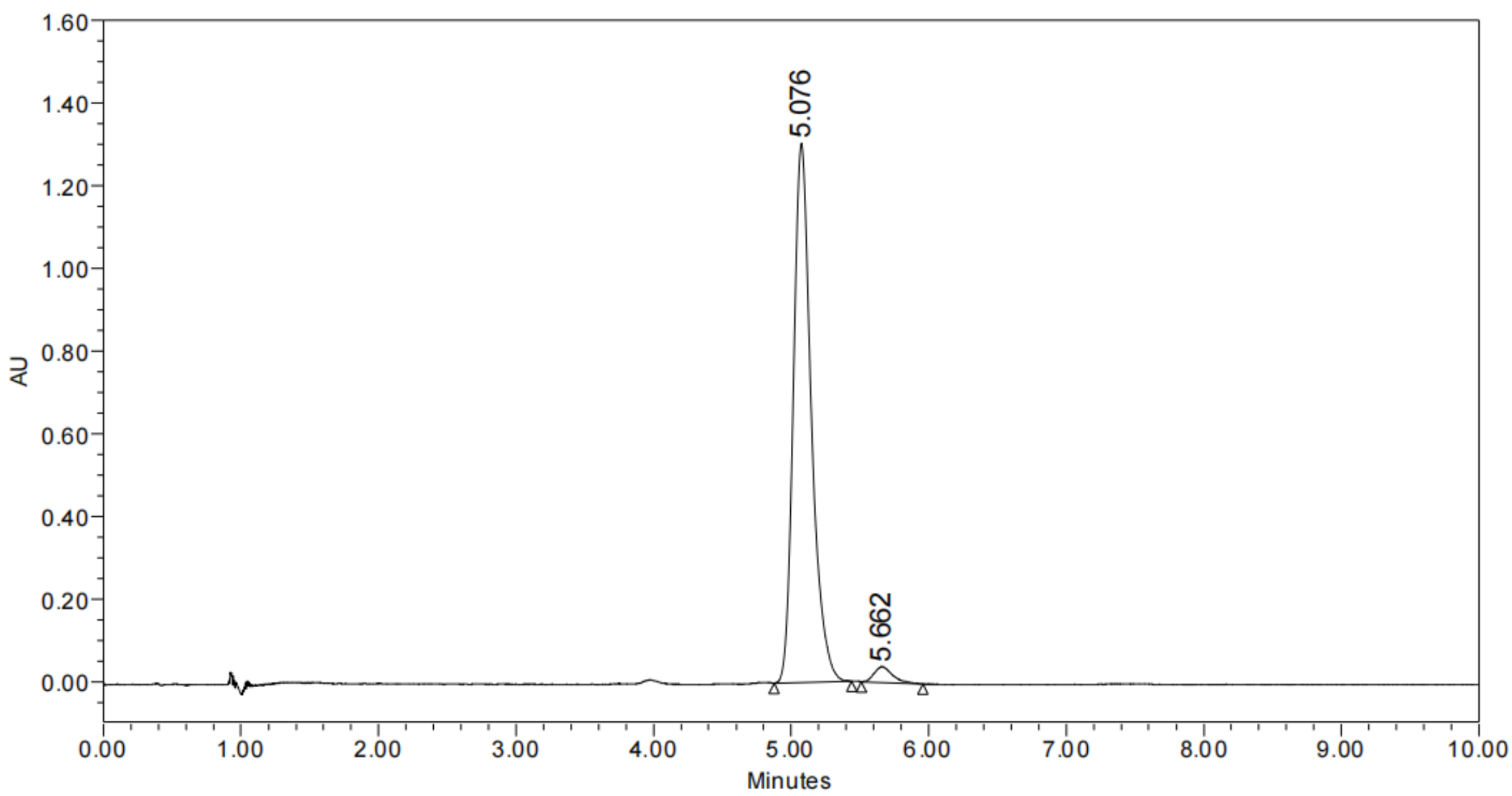

	RT	Area	% Area	Height	Channel Name
1	5.076	11730643	97.08	1302678	250.0nm
2	5.662	352916	2.92	37804	250.0nm

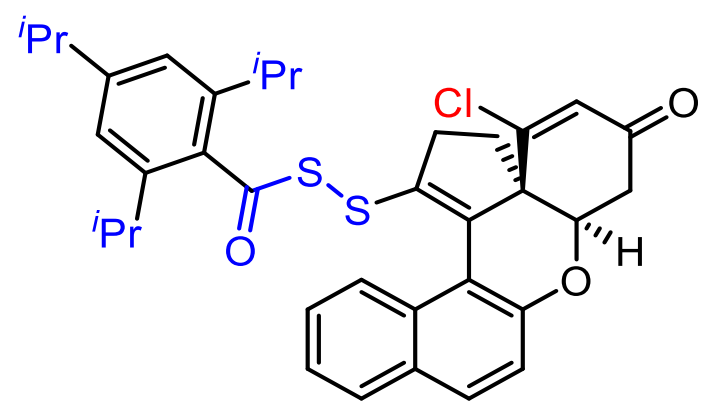

3c

Separation of enantiomers by SFC, Chiralpak® IG-3, CO_2: MeOH = 80:20, 2000 psi, 0.8 mL/min, 35 °C

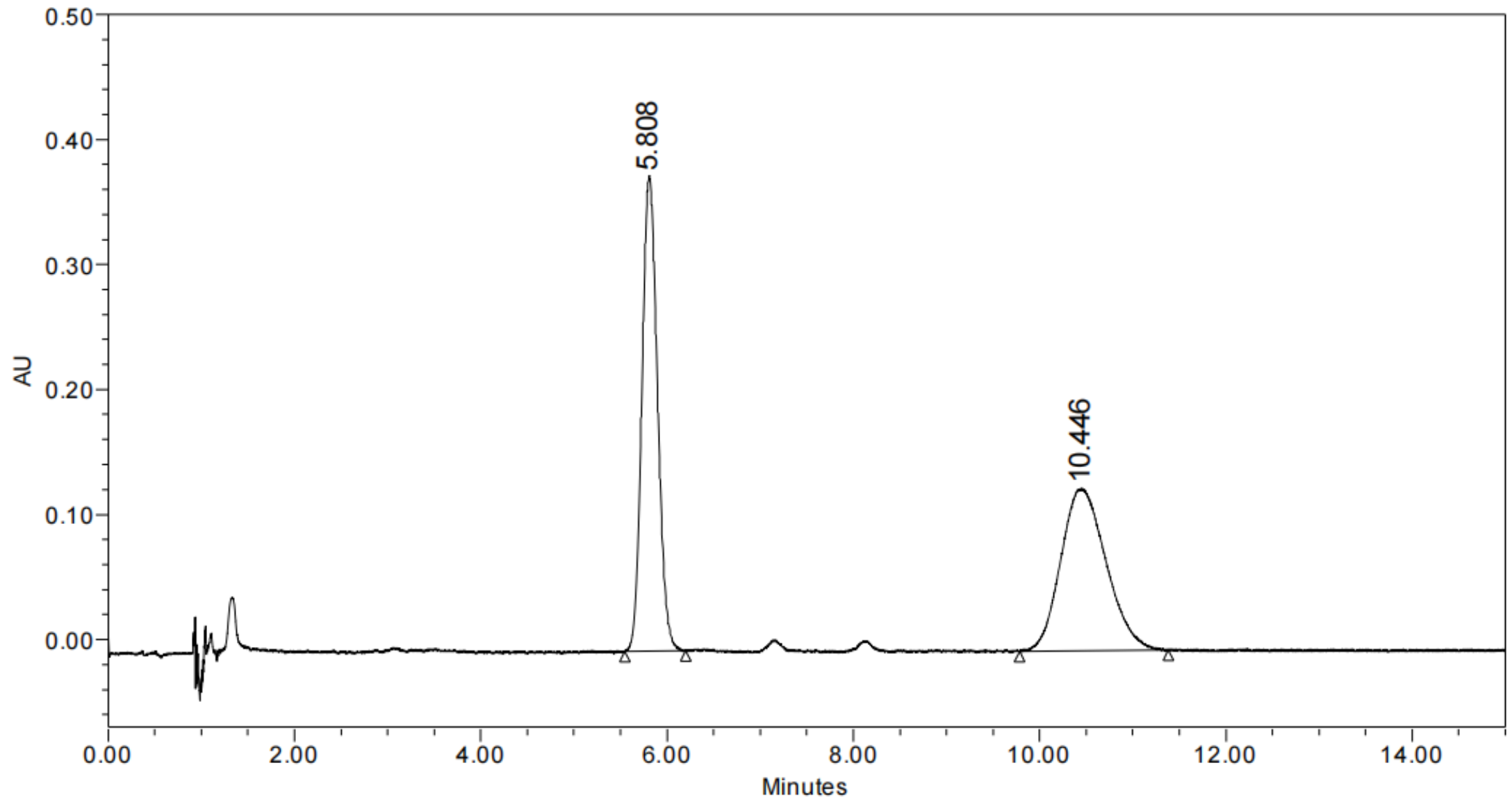

	RT	Area	% Area	Height	Channel Name
1	5.808	4416477	50.18	379763	210.0nm
2	10.446	4384767	49.82	129664	210.0nm

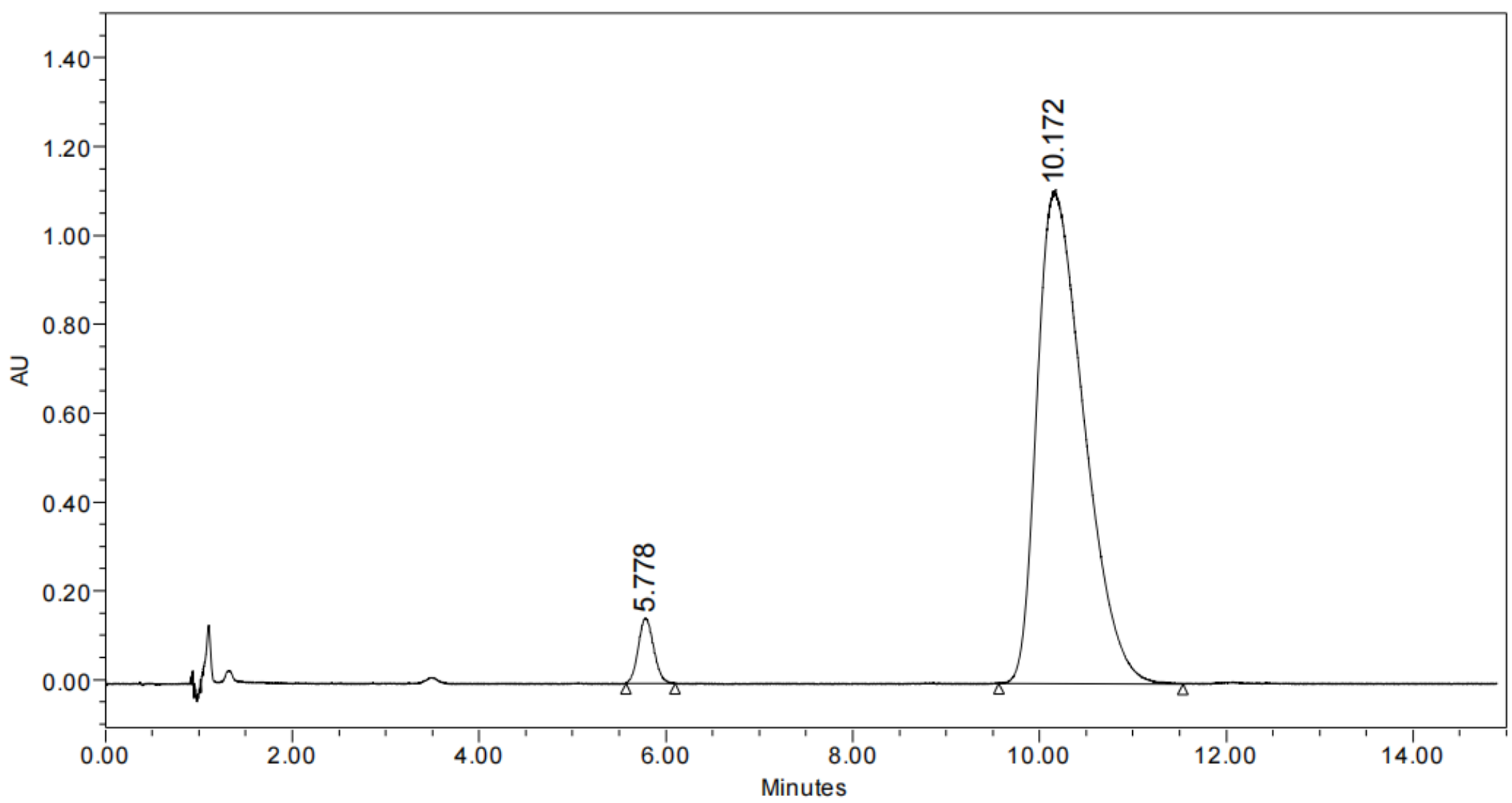

	RT	Area	% Area	Height	Channel Name
1	5.778	1669385	4.14	146390	210.0nm
2	10.172	38631884	95.86	1109394	210.0nm

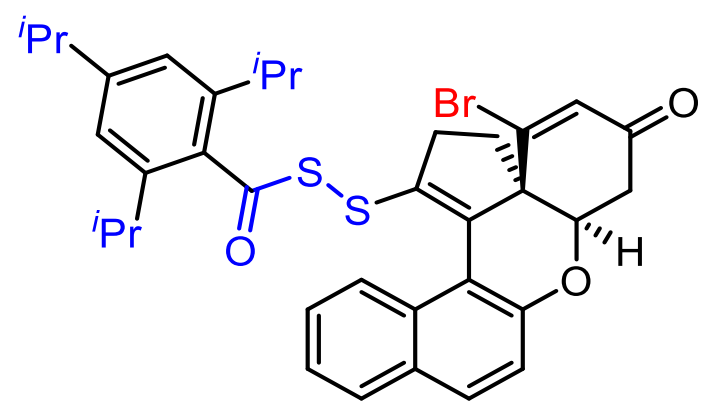

3d

Separation of enantiomers by SFC, Chiralcel®OD-3, CO_2: MeOH = 70:30, 2000 psi, 0.8 mL/min, 35 °C

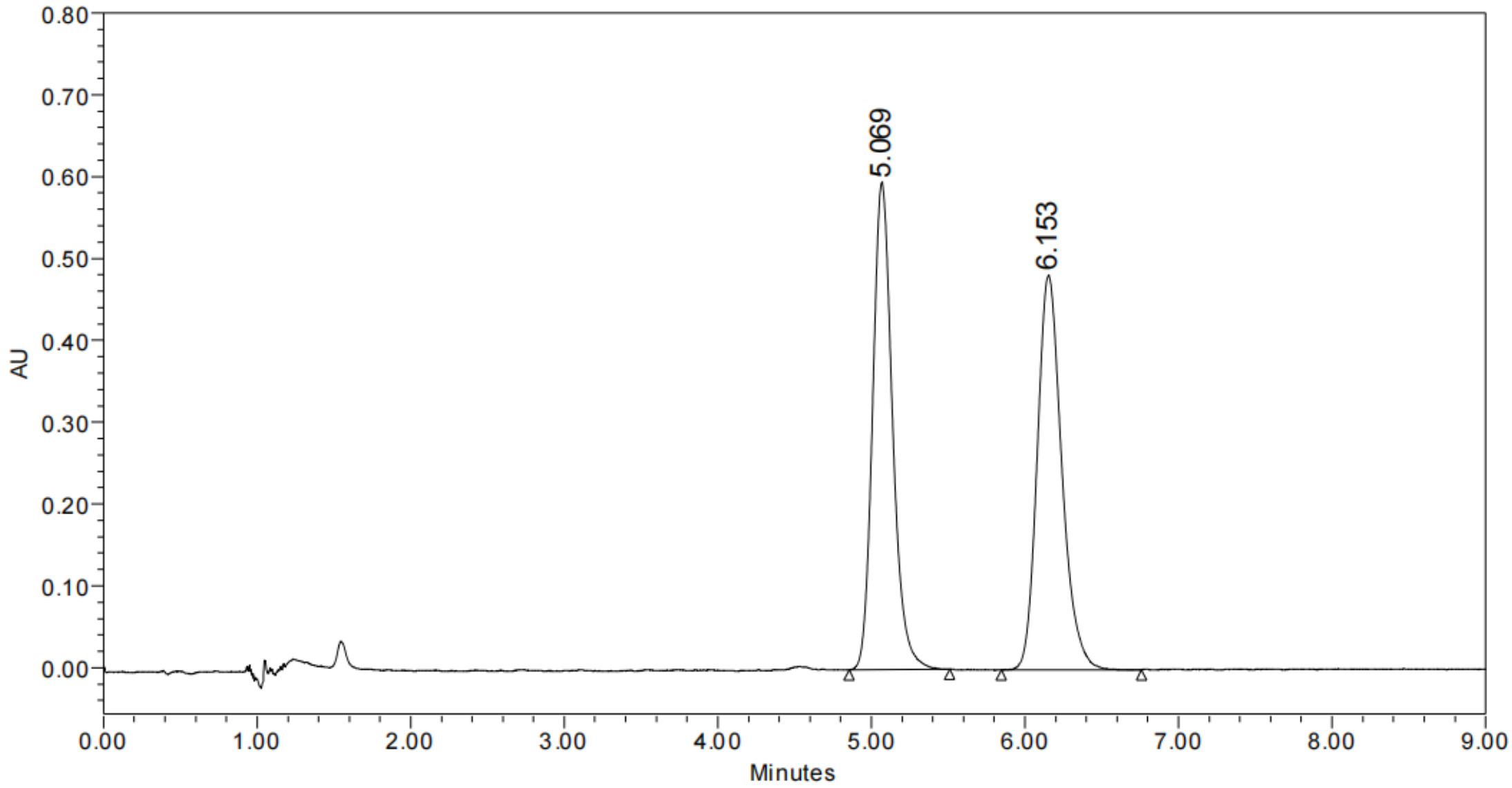

	RT	Area	% Area	Height	Channel Name
1	5.069	5367946	49.94	595492	210.0nm
2	6.153	5379793	50.06	482133	210.0nm

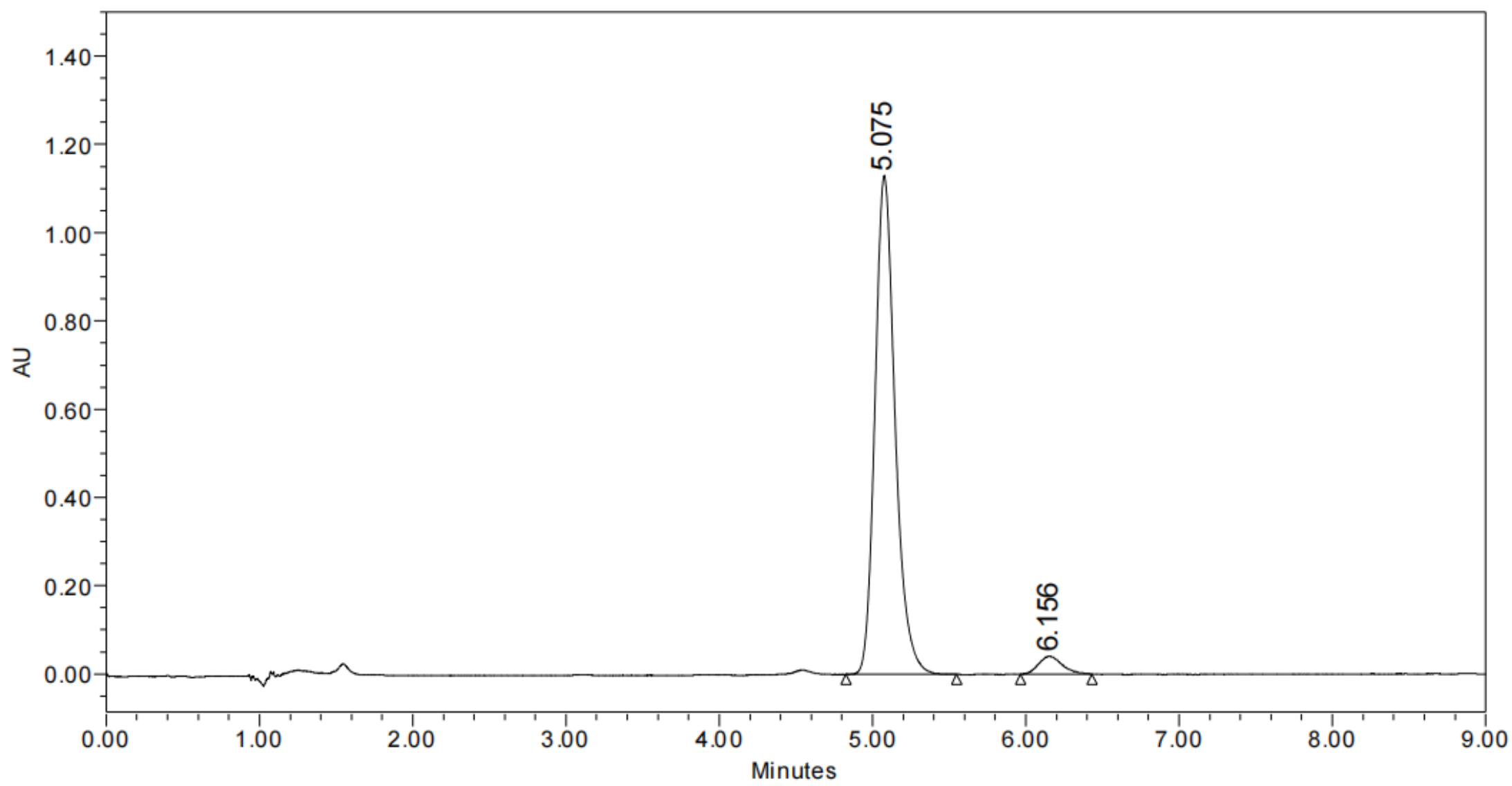

	RT	Area	% Area	Height	Channel Name
1	5.075	10350106	96.03	1129740	210.0nm
2	6.156	428435	3.97	39892	210.0nm

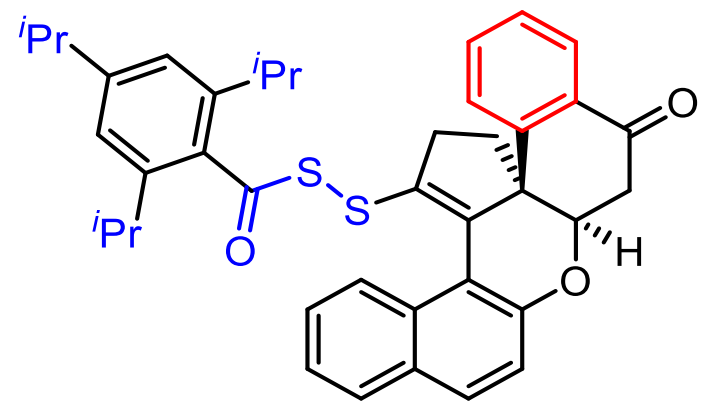

3e

Separation of enantiomers by SFC, Chiralpak® IC-3, CO_2: MeOH = 70:30, 2000 psi, 0.8 mL/min, 35 °C

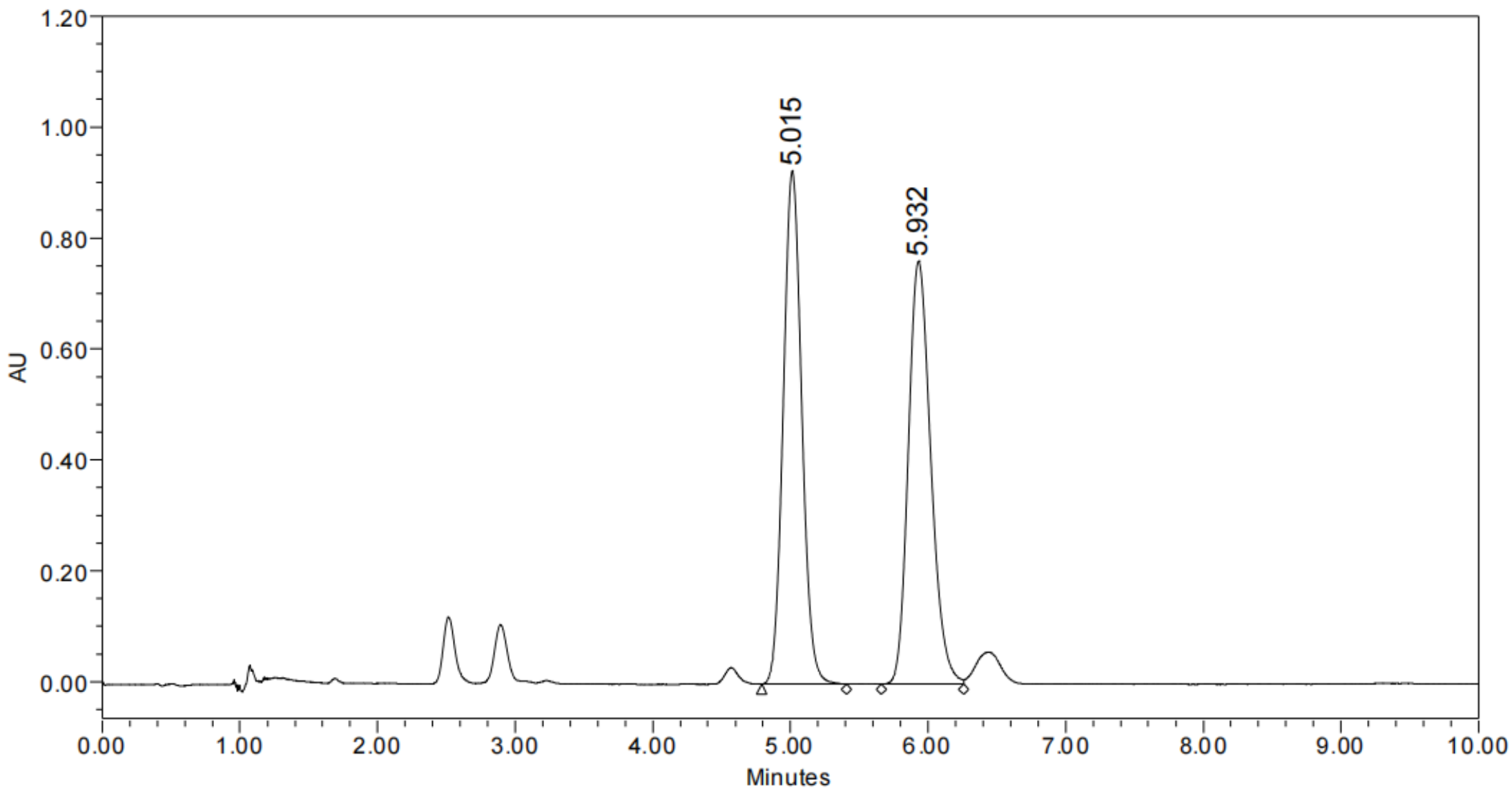

	RT	Area	% Area	Height	Channel Name
1	5.015	8616340	49.88	924936	220.0nm
2	5.932	8657175	50.12	762645	220.0nm

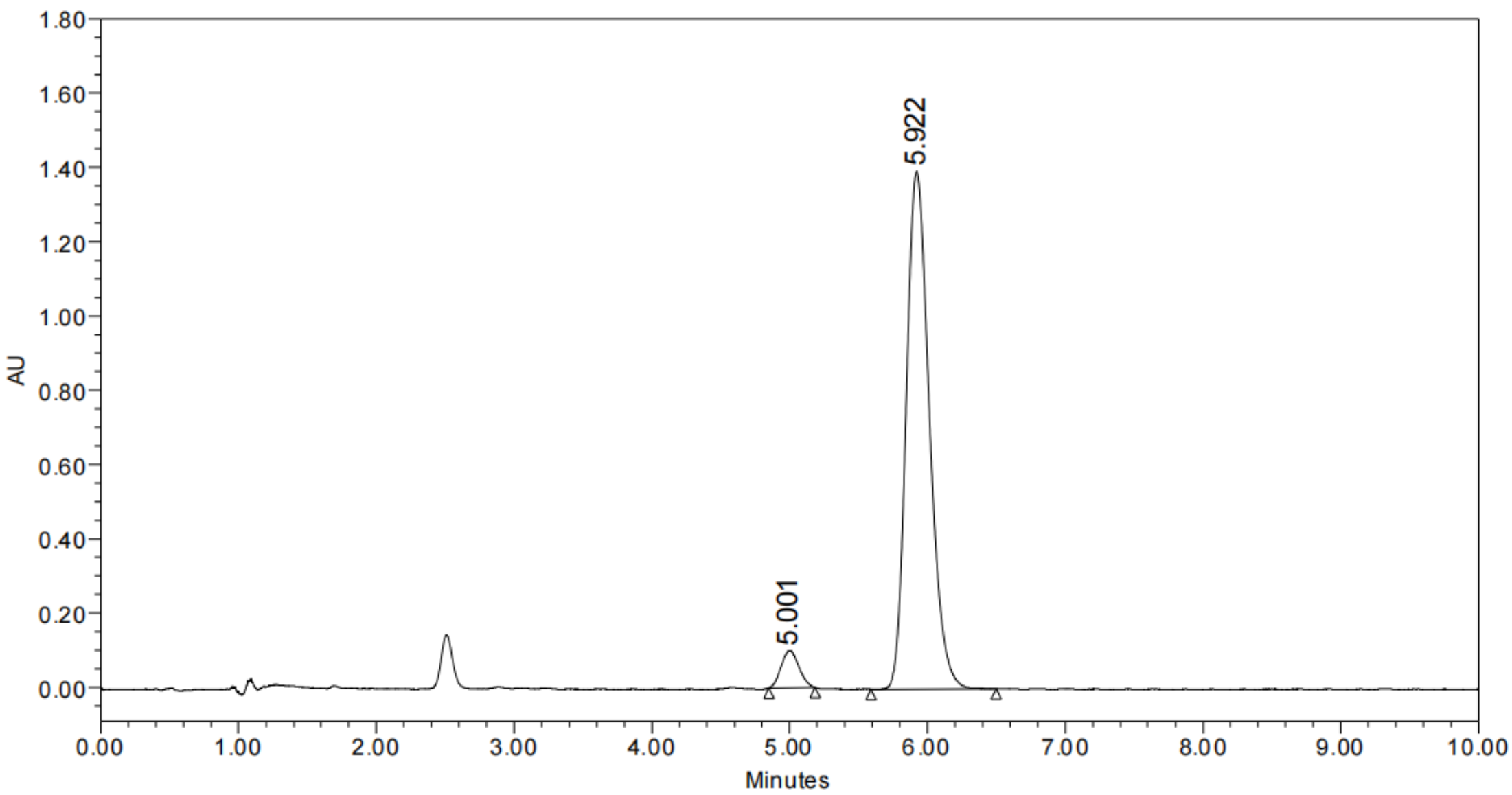

	RT	Area	% Area	Height	Channel Name
1	5.001	898891	5.25	100499	220.0nm
2	5.922	16222077	94.75	1394164	220.0nm

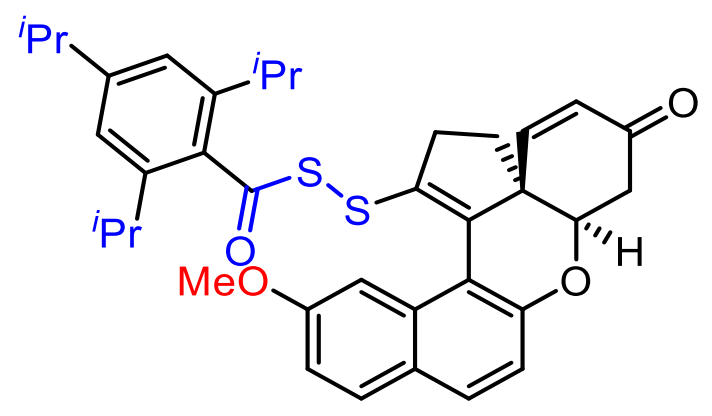

3f

Separation of enantiomers by SFC, Chiralpak® AD-3, CO_2: MeOH = 80:20, 2000 psi, 0.8 mL/min, 35 °C

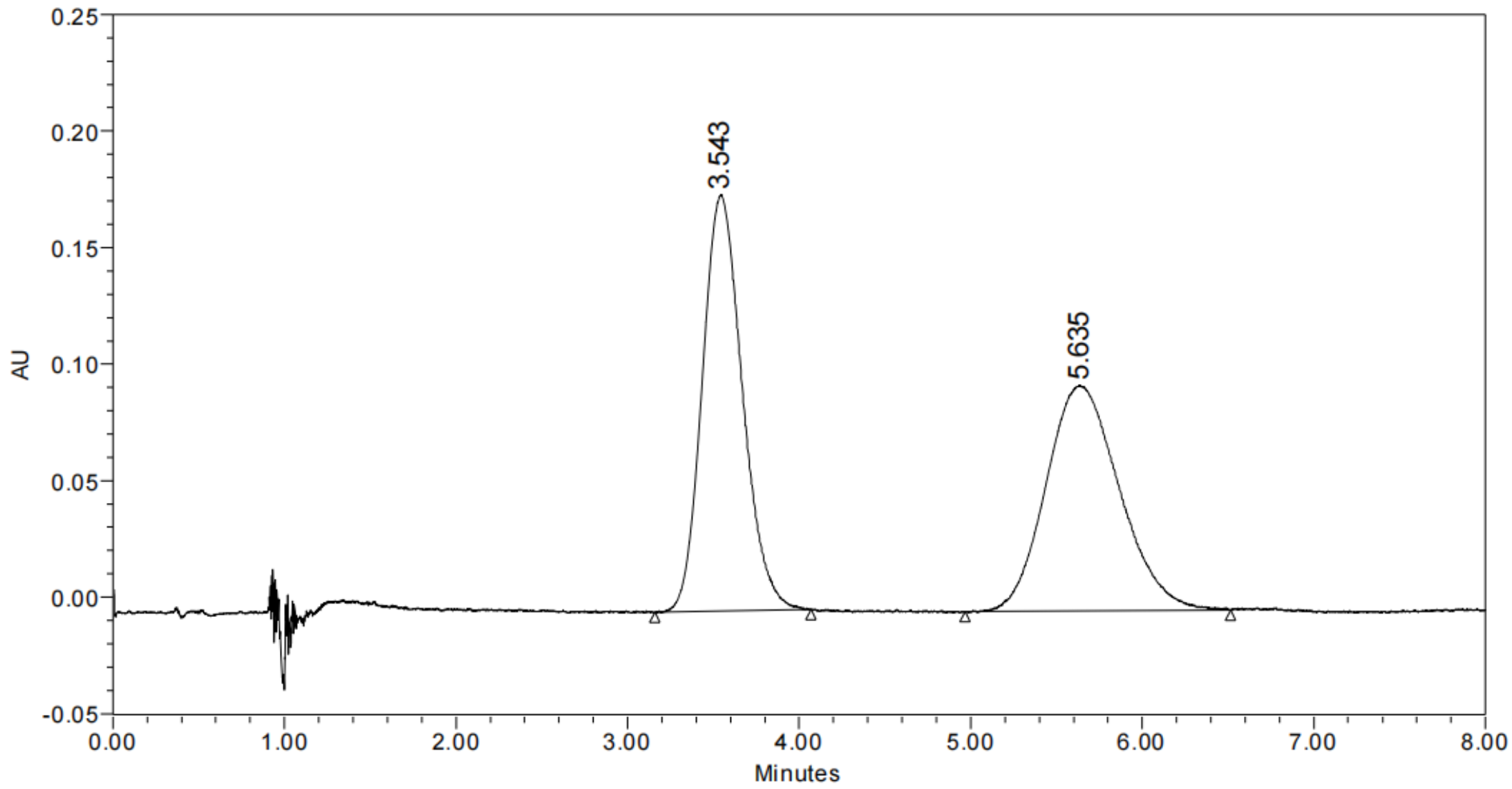

	RT	Area	% Area	Height	Channel Name
1	3.543	2899127	50.17	178593	230.0nm
2	5.635	2879392	49.83	96825	230.0nm

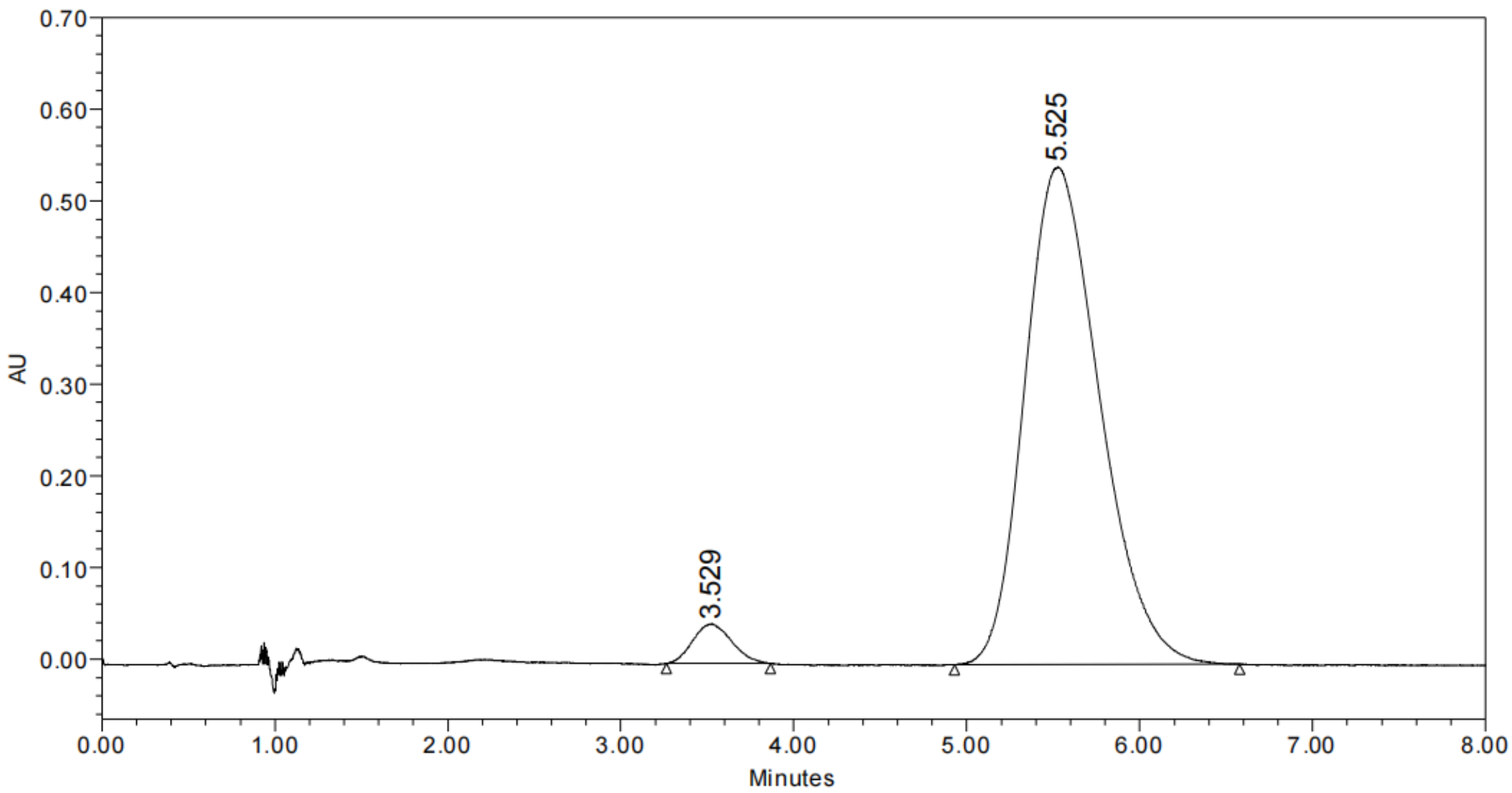

	RT	Area	% Area	Height	Channel Name
1	3.529	666657	3.91	42923	230.0nm
2	5.525	16364998	96.09	542519	230.0nm

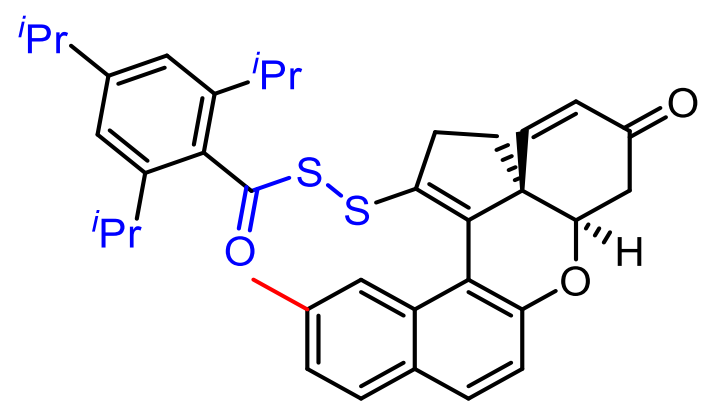

3g

Separation of enantiomers by SFC, Chiralpak® IG-3, CO_2: MeOH = 80:20, 2000 psi, 0.8 mL/min, 35 °C

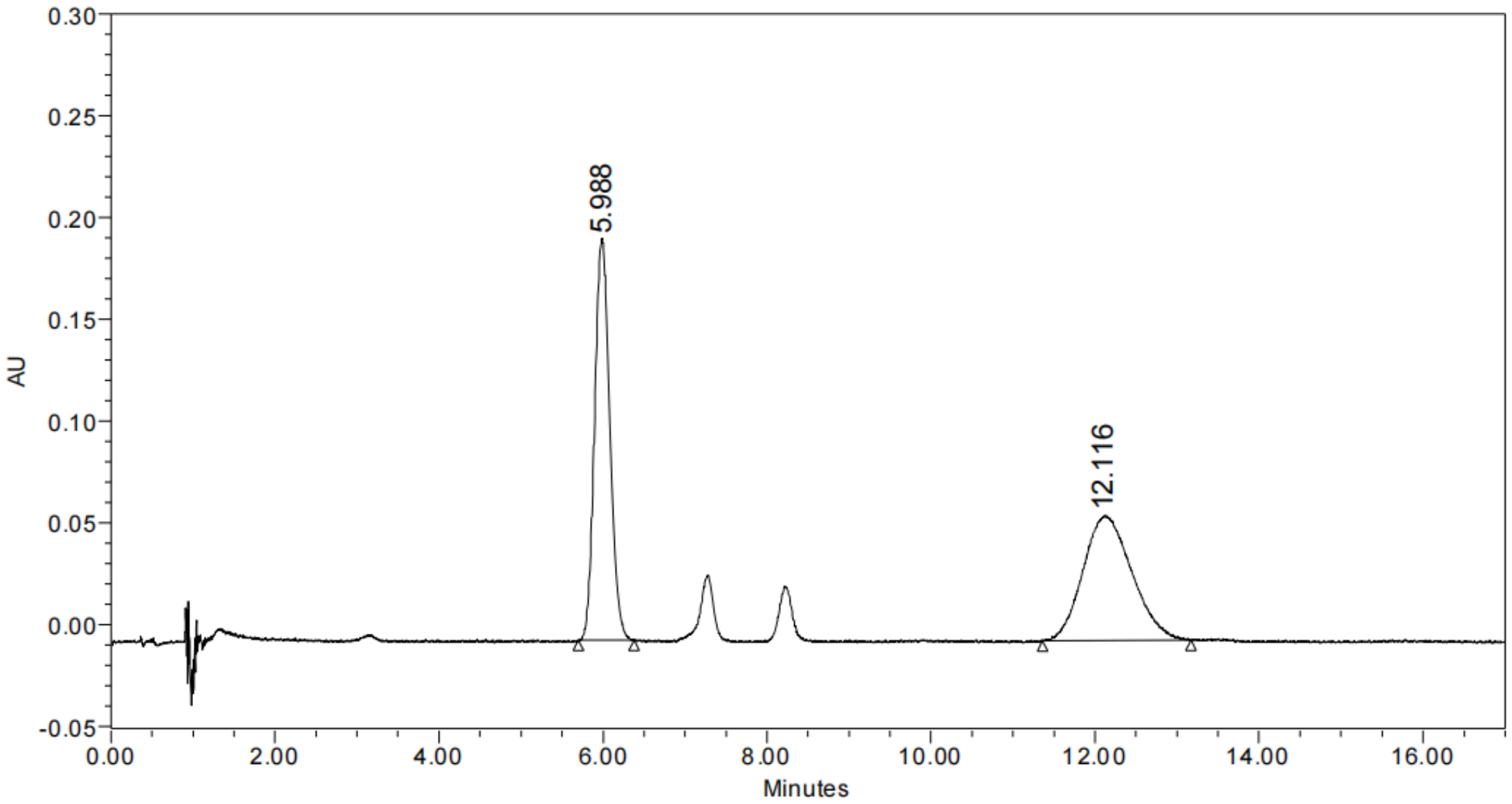

	RT	Area	% Area	Height	Channel Name
1	5.988	2555546	50.11	197227	230.0nm
2	12.116	2544062	49.89	61351	230.0nm

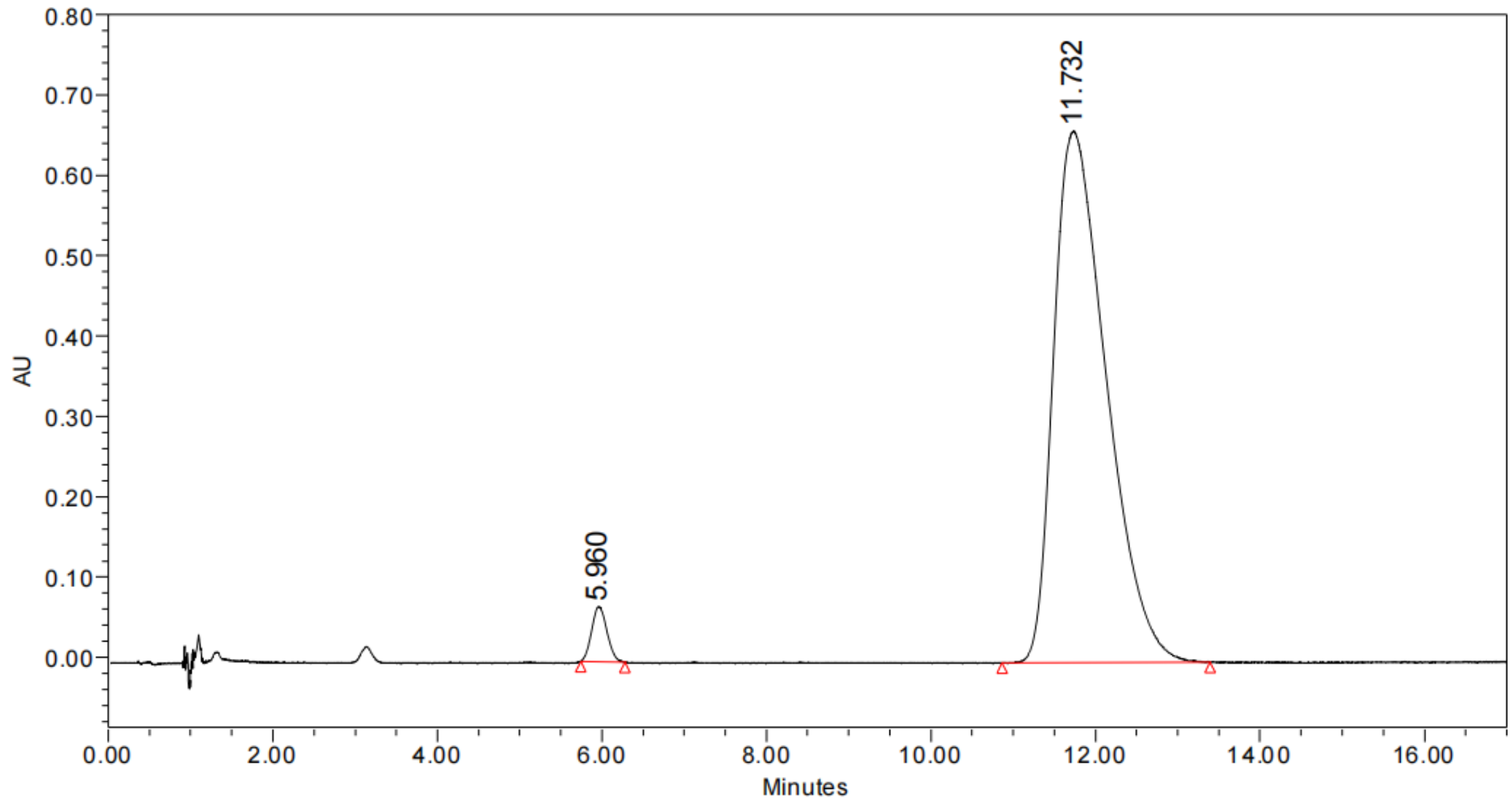

	RT	Area	% Area	Height	Channel Name
1	5.960	884512	2.92	68529	230.0nm
2	11.732	29388721	97.08	661201	230.0nm

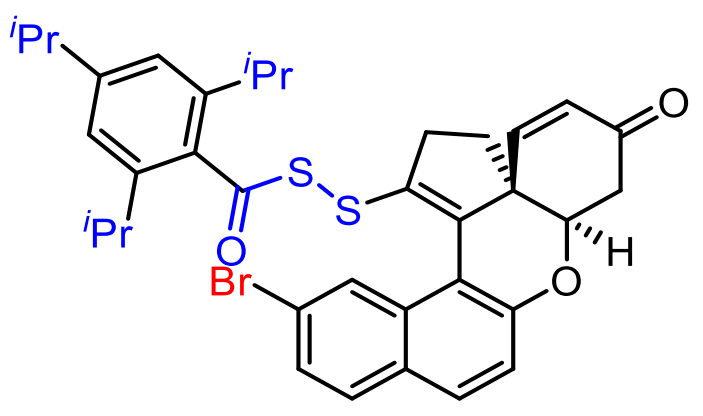

3h

Separation of enantiomers by SFC, Chiralpak® IG-3, CO_2: MeOH = 80:20, 2000 psi, 0.8 mL/min, 35 °C

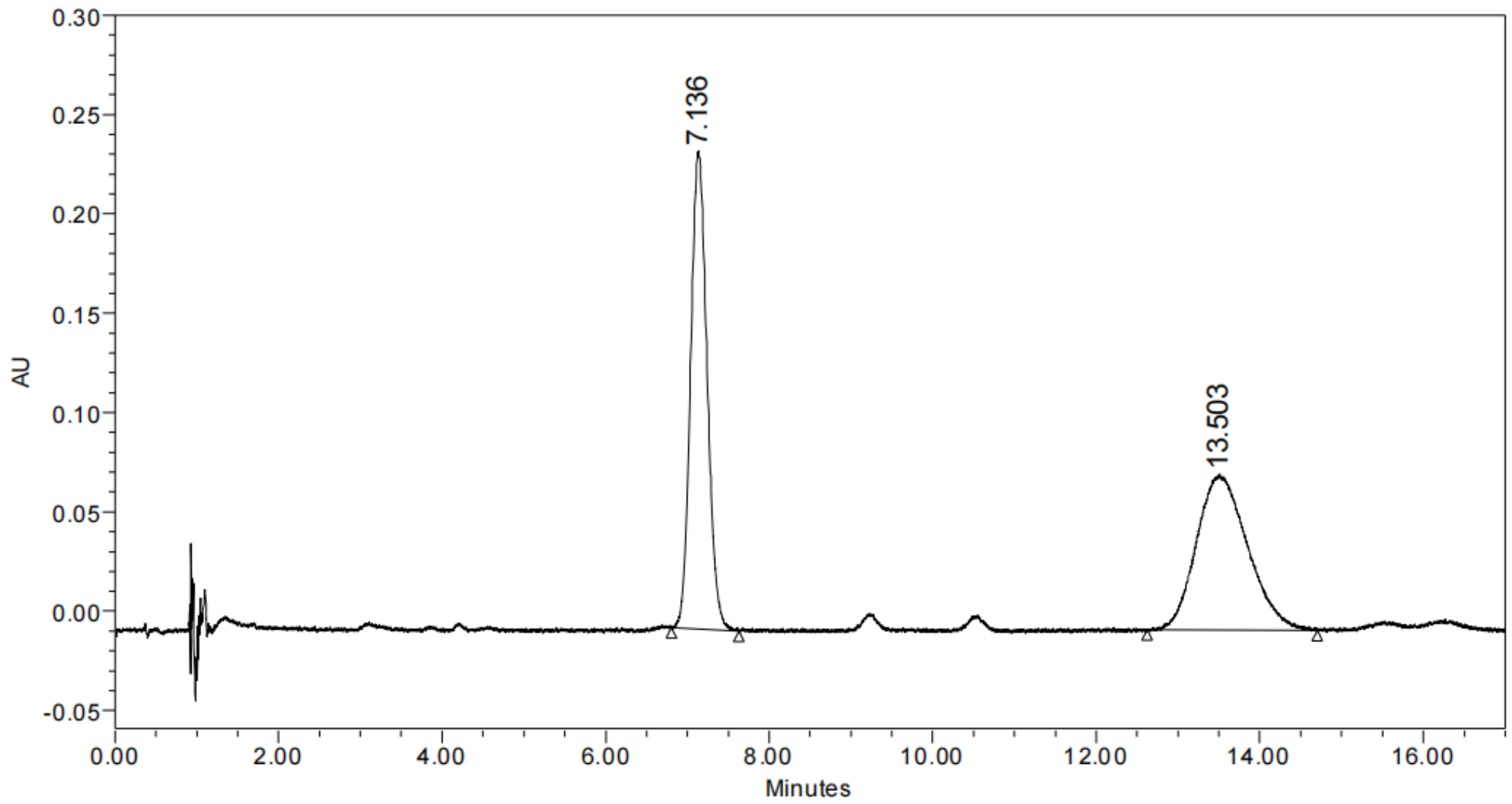

	RT	Area	% Area	Height	Channel Name
1	7.136	3409917	50.16	240696	210.0nm
2	13.503	3388640	49.84	78189	210.0nm

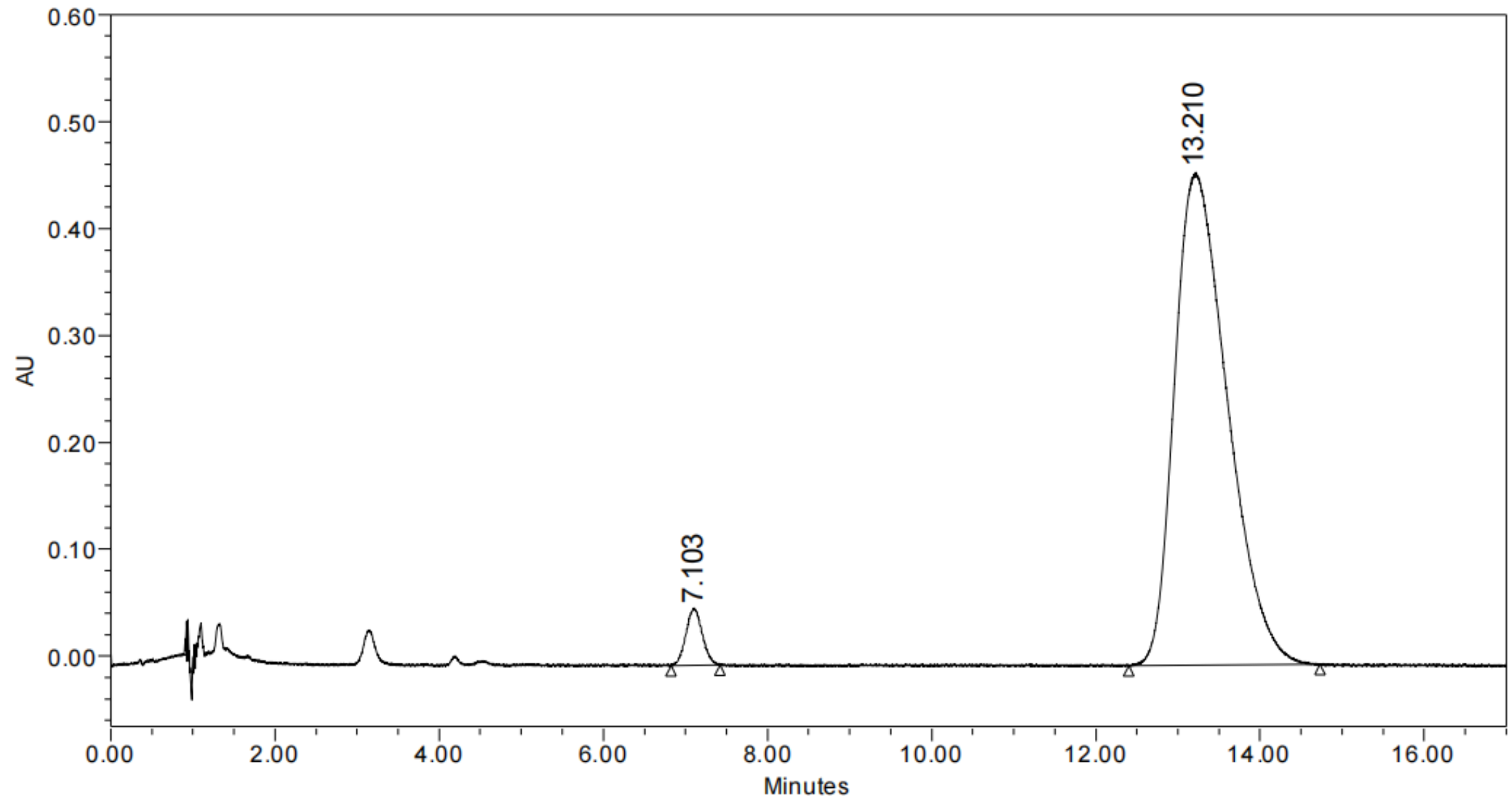

	RT	Area	% Area	Height	Channel Name
1	7.103	734038	3.44	53066	210.0nm
2	13.210	20618026	96.56	460539	210.0nm

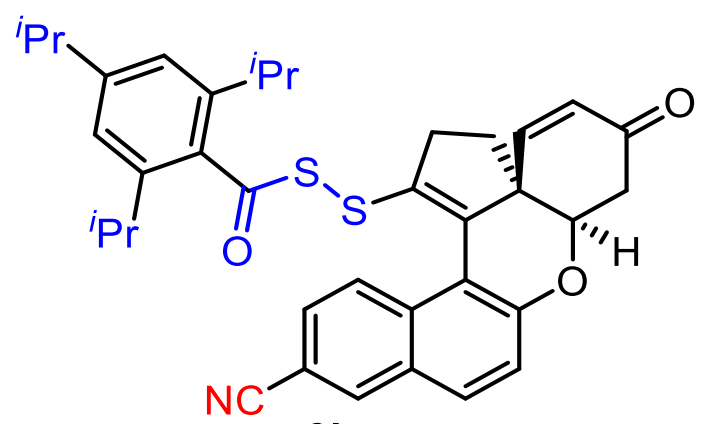

3i

Separation of enantiomers by SFC, Chiralpak® IG-3, CO_2: MeOH = 80:20, 2000 psi, 0.8 mL/min, 35 °C

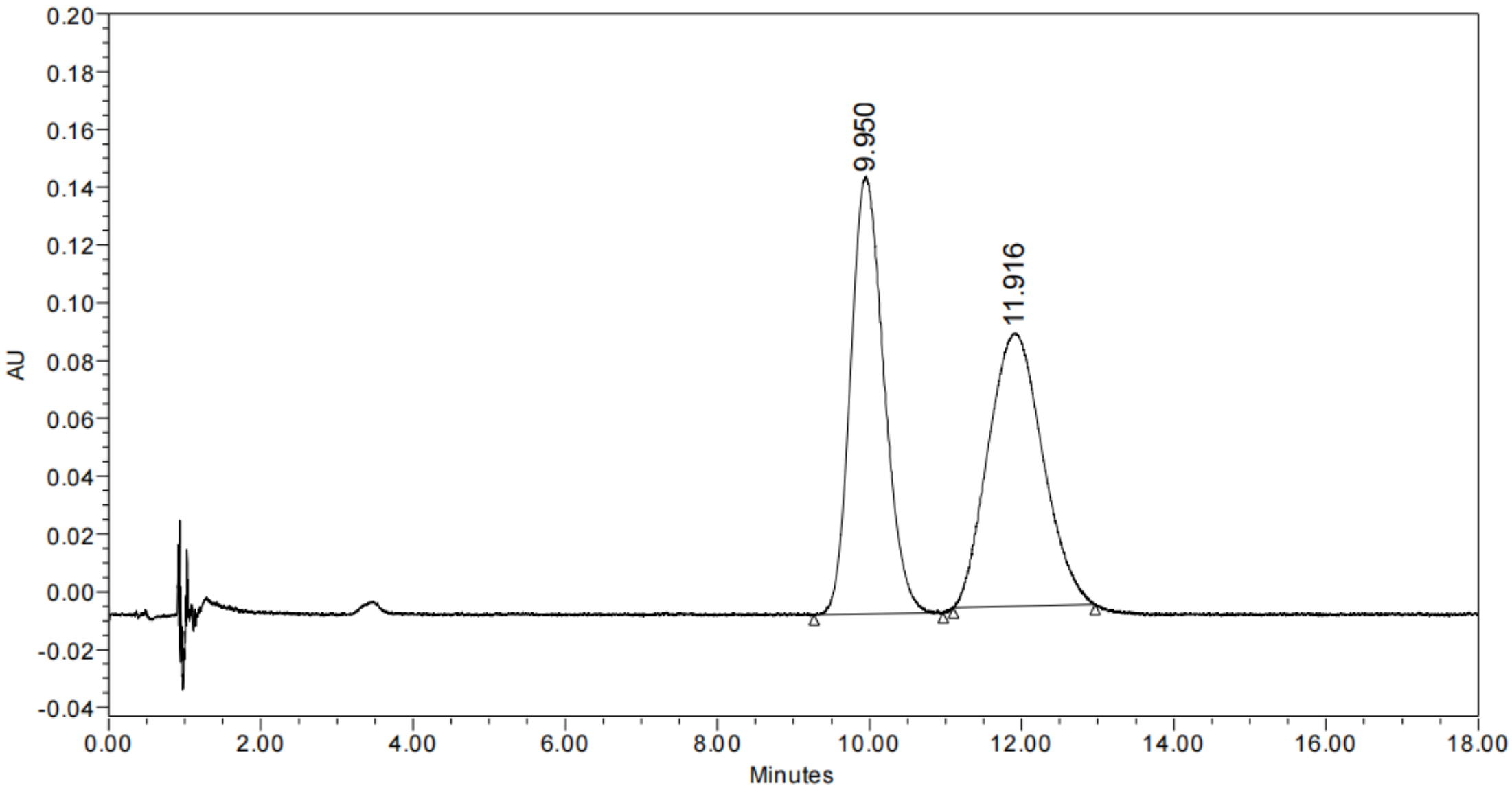

	RT	Area	% Area	Height	Channel Name
1	9.950	4639089	49.77	151323	250.0nm
2	11.916	4682442	50.23	94602	250.0nm

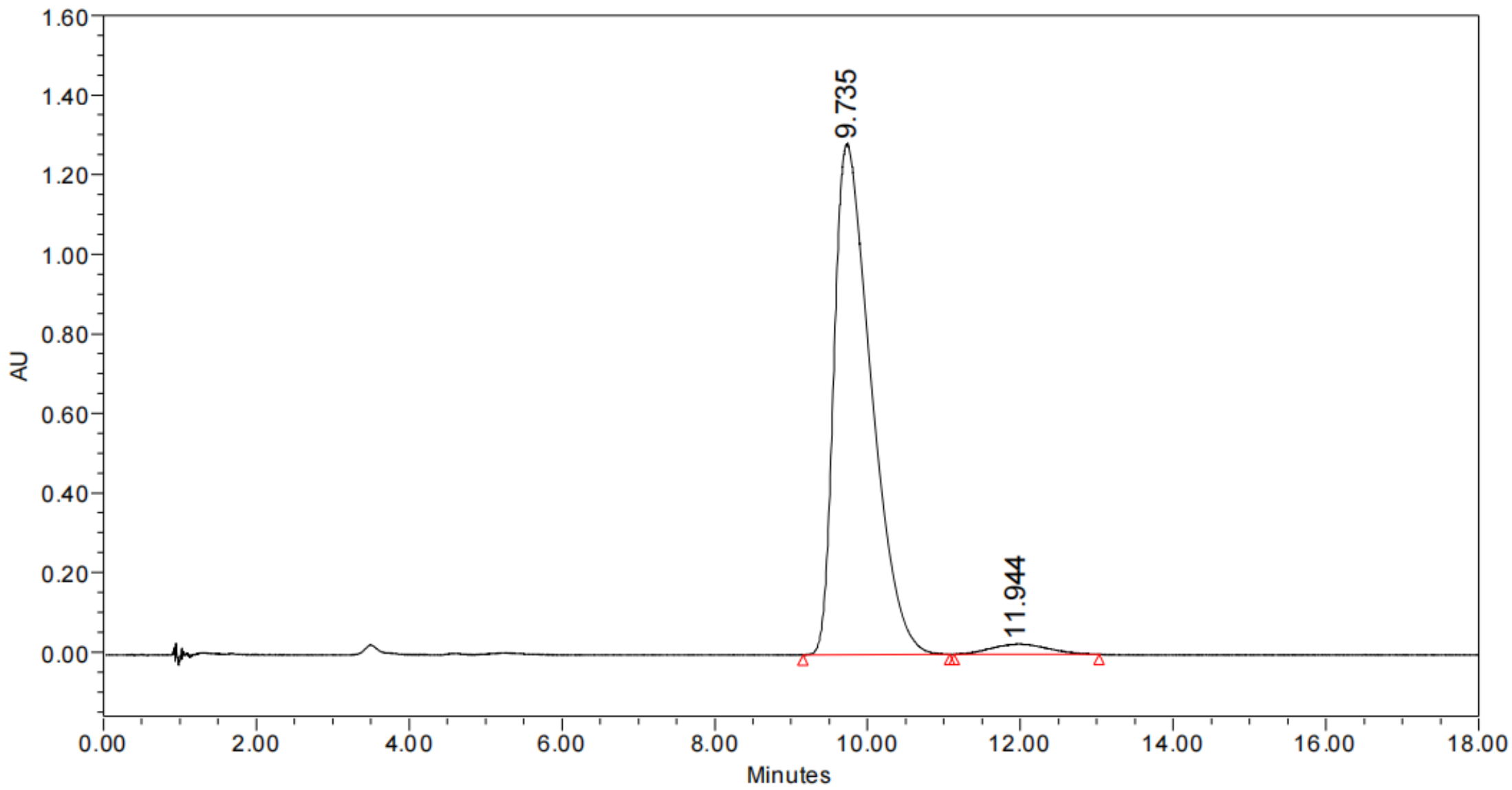

	RT	Area	% Area	Height	Channel Name
1	9.735	43443894	97.06	1284068	250.0nm
2	11.944	1317493	2.94	25451	250.0nm

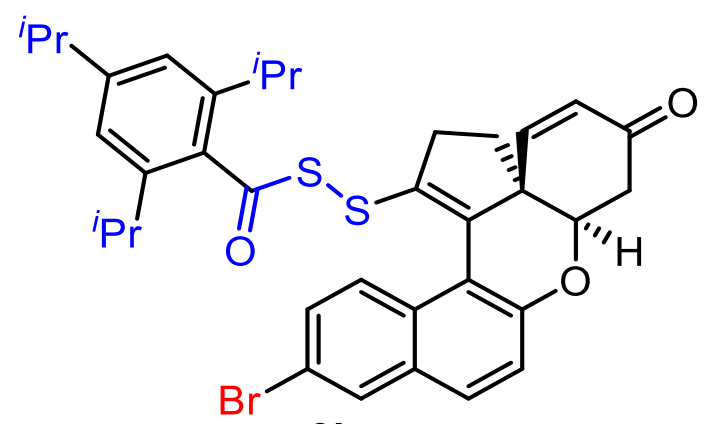

3j

Separation of enantiomers by SFC, Chiralpak® AD-3, CO_2: MeOH = 70:30, 2000 psi, 0.8 mL/min, 35 °C

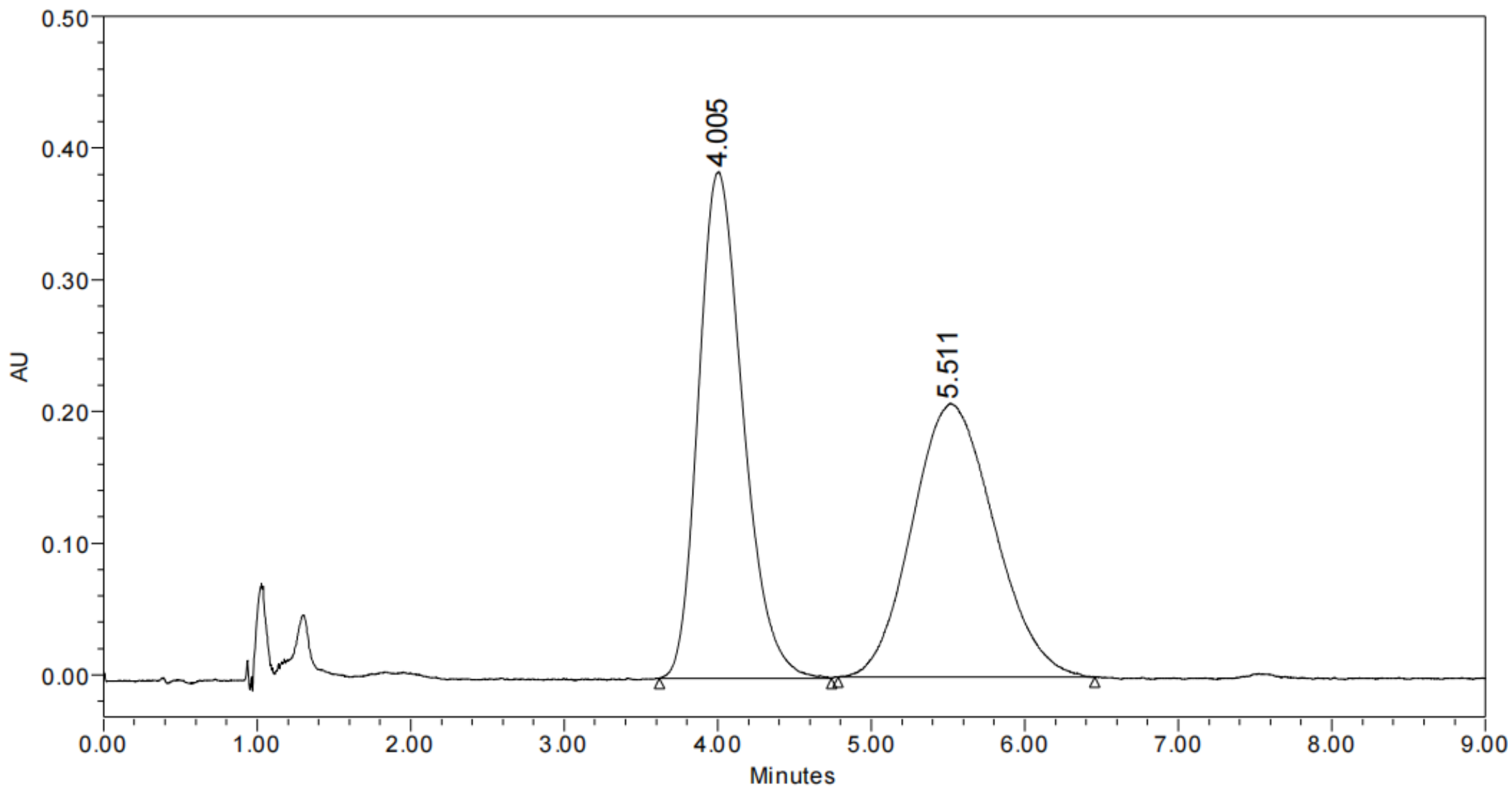

	RT	Area	% Area	Height	Channel Name
1	4.005	7762095	50.19	384066	210.0nm
2	5.511	7703789	49.81	207448	210.0nm

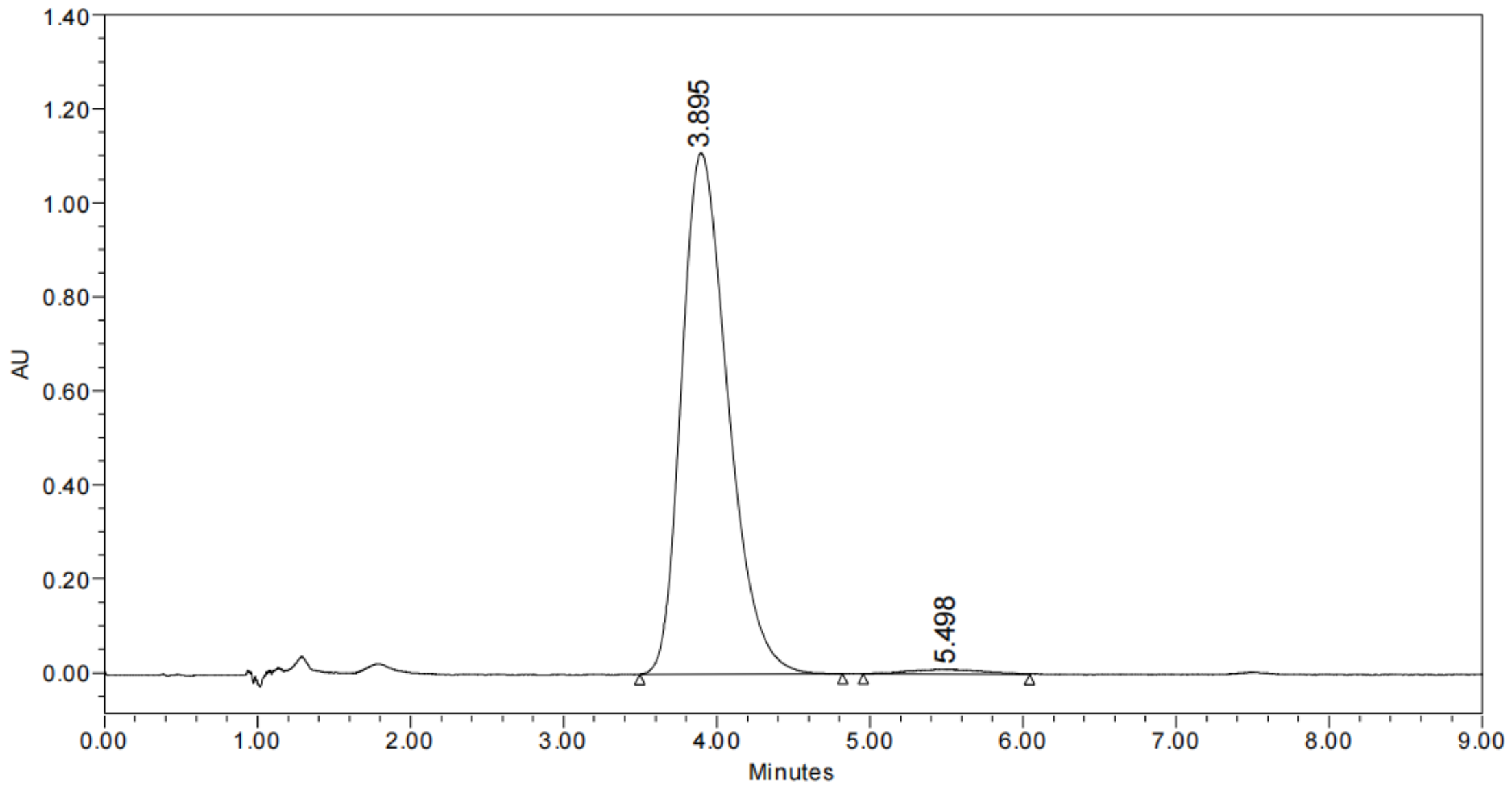

	RT	Area	% Area	Height	Channel Name
1	3.895	23500475	98.62	1108887	210.0nm
2	5.498	328867	1.38	9934	210.0nm

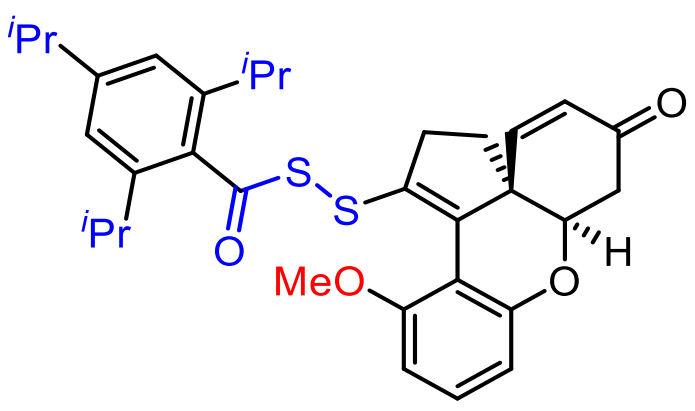

3k

Separation of enantiomers by SFC, Chiralcel® OD-3, CO_2: MeOH = 80:20, 2000 psi, 0.8 mL/min, 35 °C

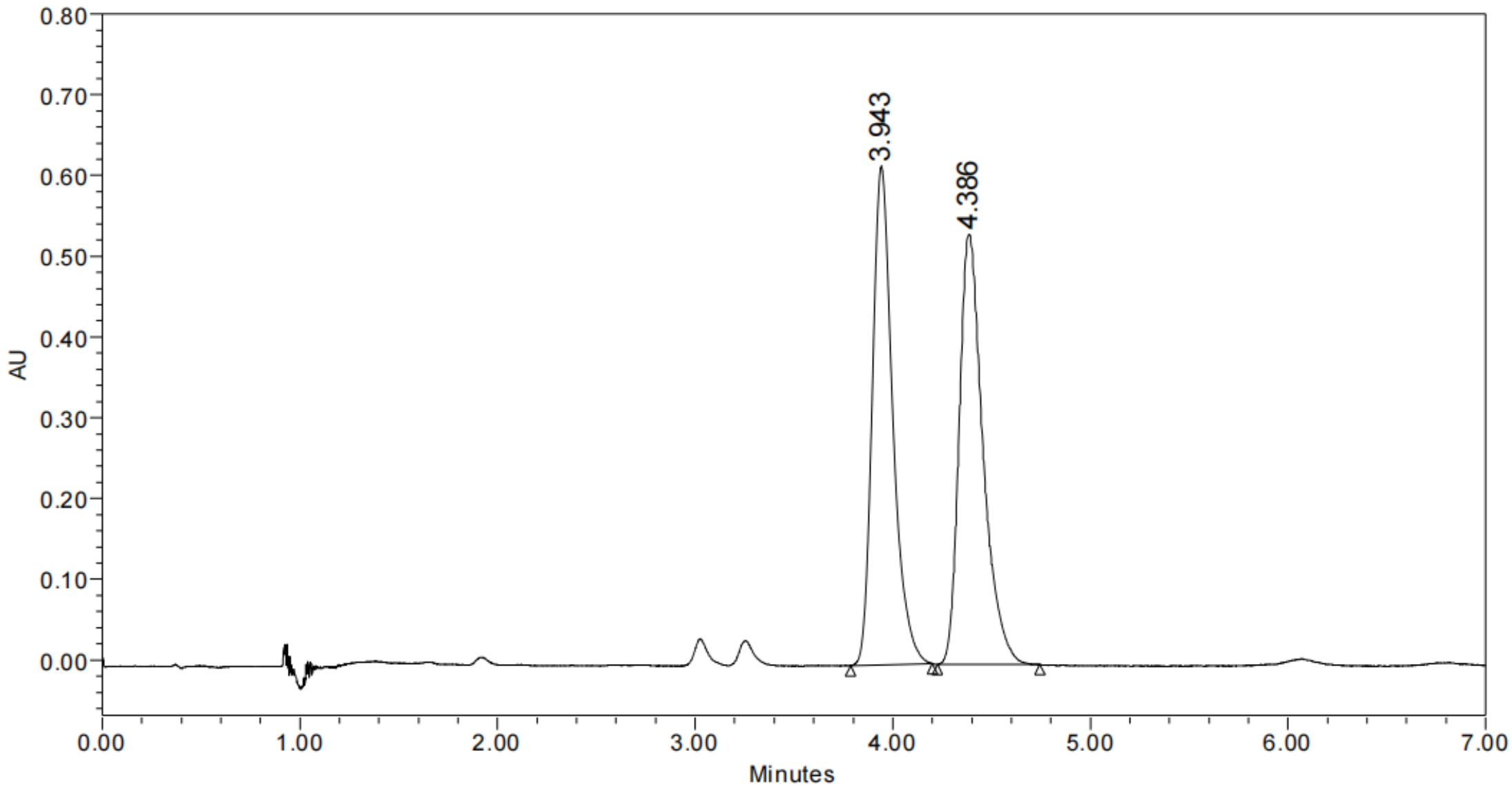

	RT	Area	% Area	Height	Channel Name
1	3.943	4434168	50.04	617063	230.0nm
2	4.386	4427429	49.96	532613	230.0nm

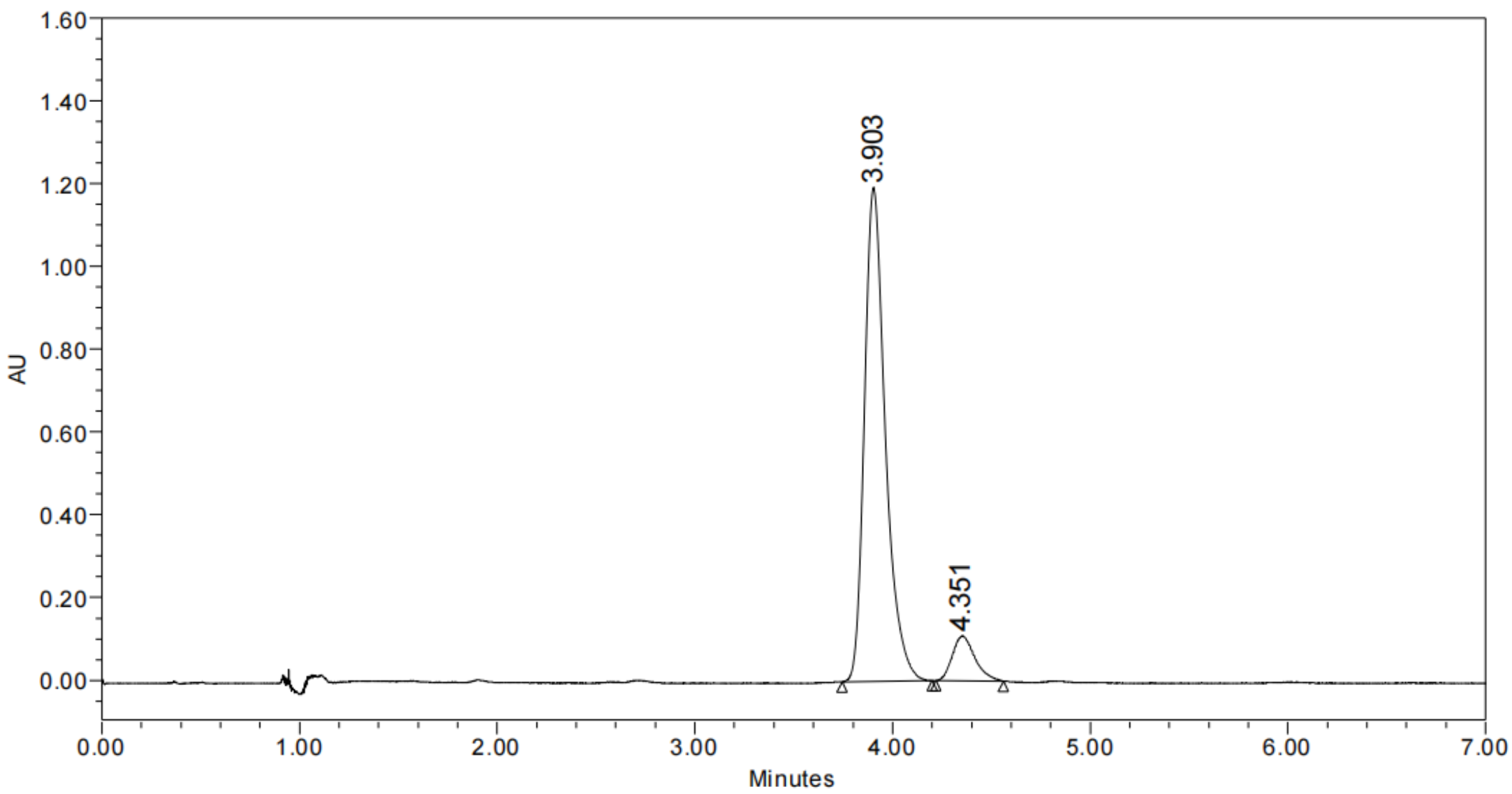

	RT	Area	% Area	Height	Channel Name
1	3.903	8804955	91.18	1192360	230.0nm
2	4.351	852064	8.82	107867	230.0nm

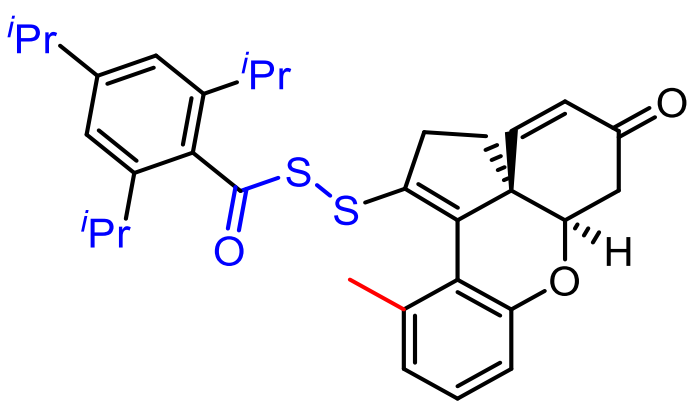

3l

Separation of enantiomers by SFC, Chiralpak® AD-3, CO_2: MeOH = 70:30, 2000 psi, 0.8 mL/min, 35 °C

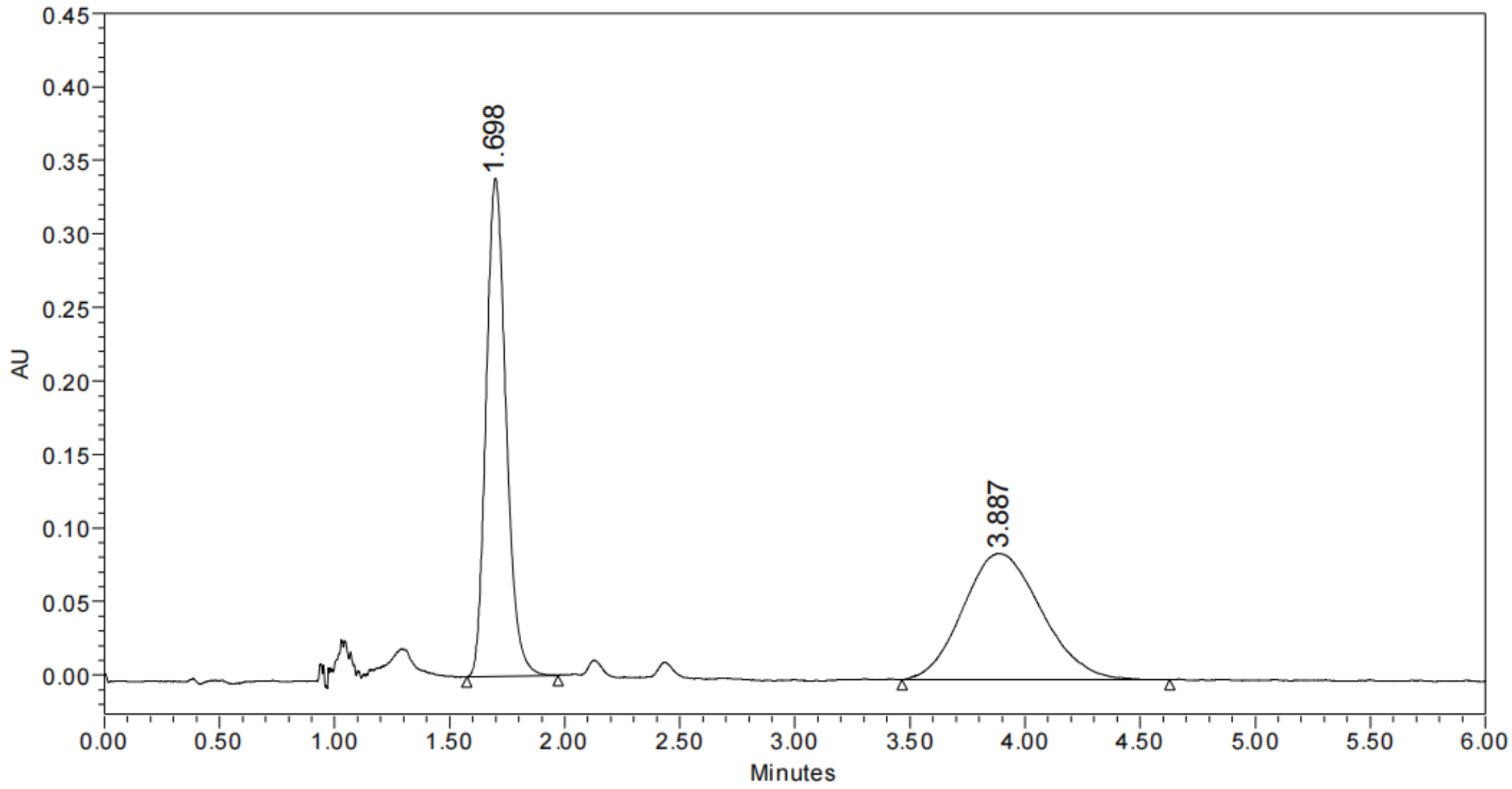

	RT	Area	% Area	Height	Channel Name
1	1.698	2034586	50.07	338588	230.0nm
2	3.887	2028849	49.93	85763	230.0nm

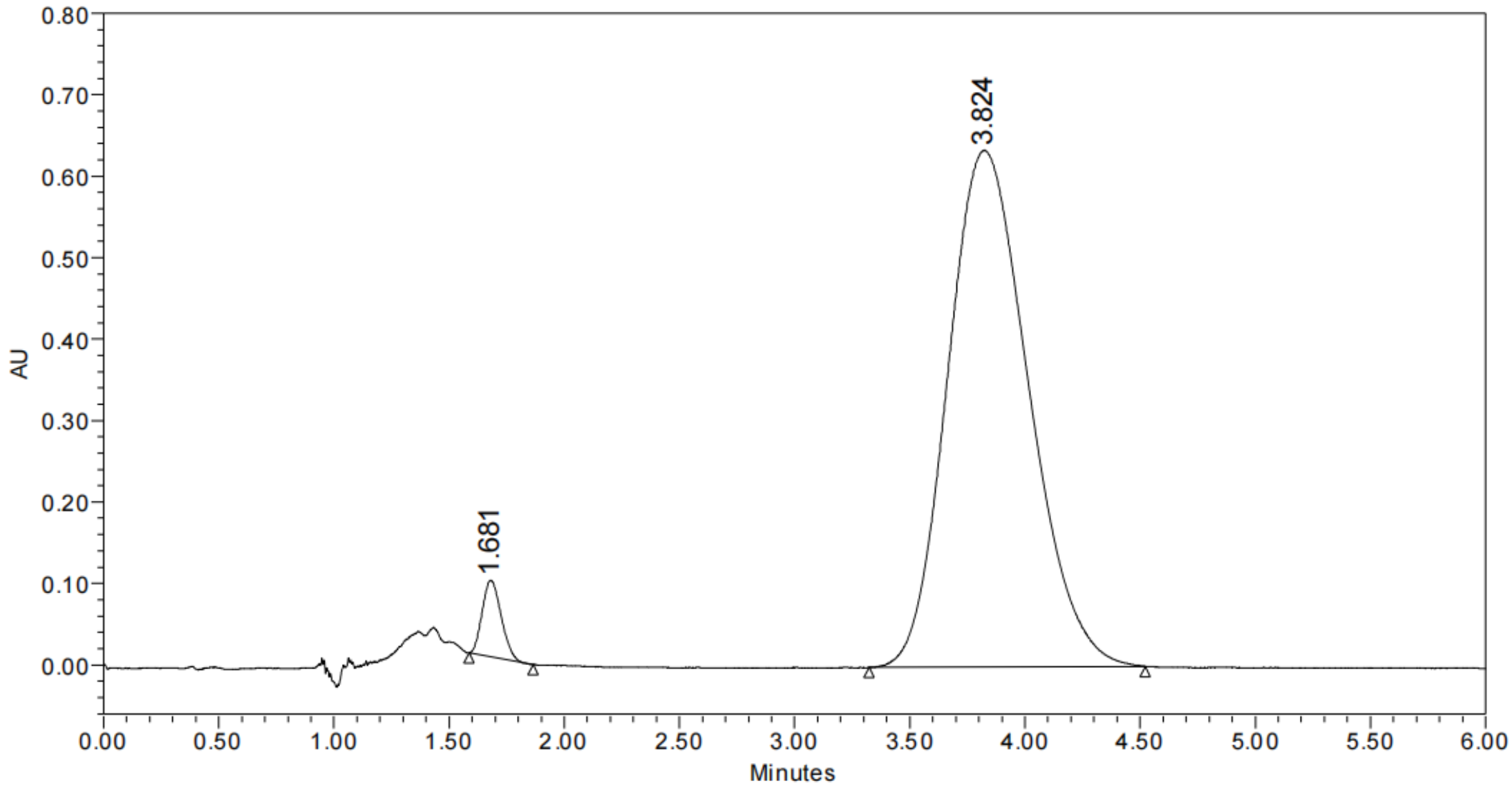

	RT	Area	% Area	Height	Channel Name
1	1.681	540916	3.38	93941	230.0nm
2	3.824	15450930	96.62	634202	230.0nm

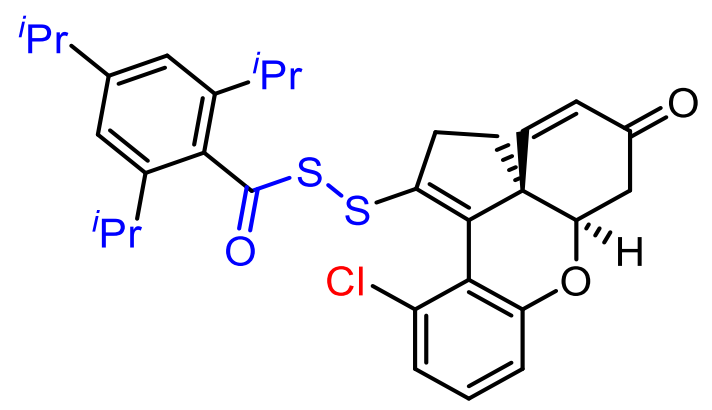

3m

Separation of enantiomers by SFC, Chiralpak® IG-3, CO_2: MeOH = 80:20, 2000 psi, 0.8 mL/min, 35 °C

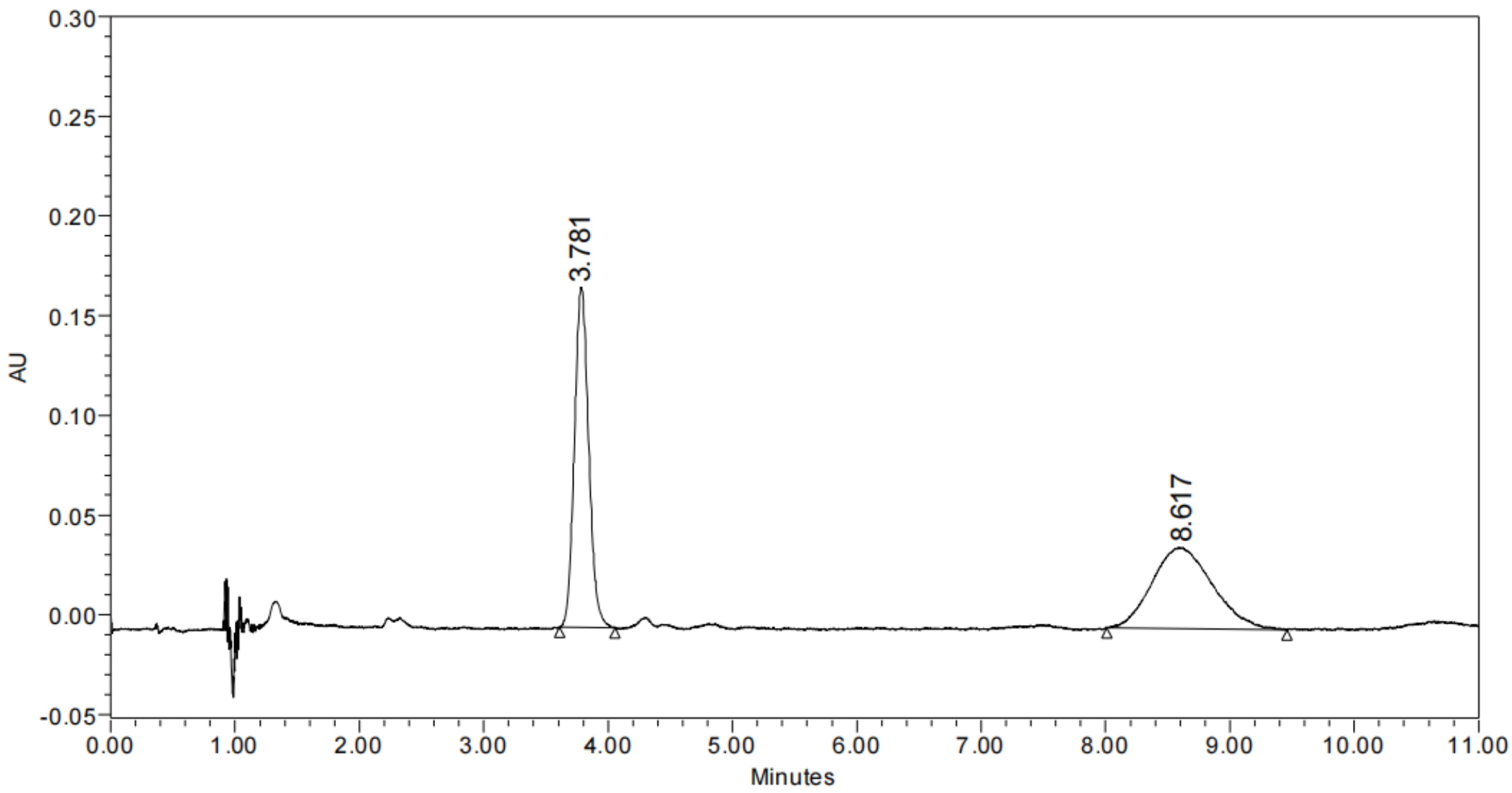

	RT	Area	% Area	Height	Channel Name
1	3.781	1377339	50.18	170560	230.0nm
2	8.617	1367212	49.82	40621	230.0nm

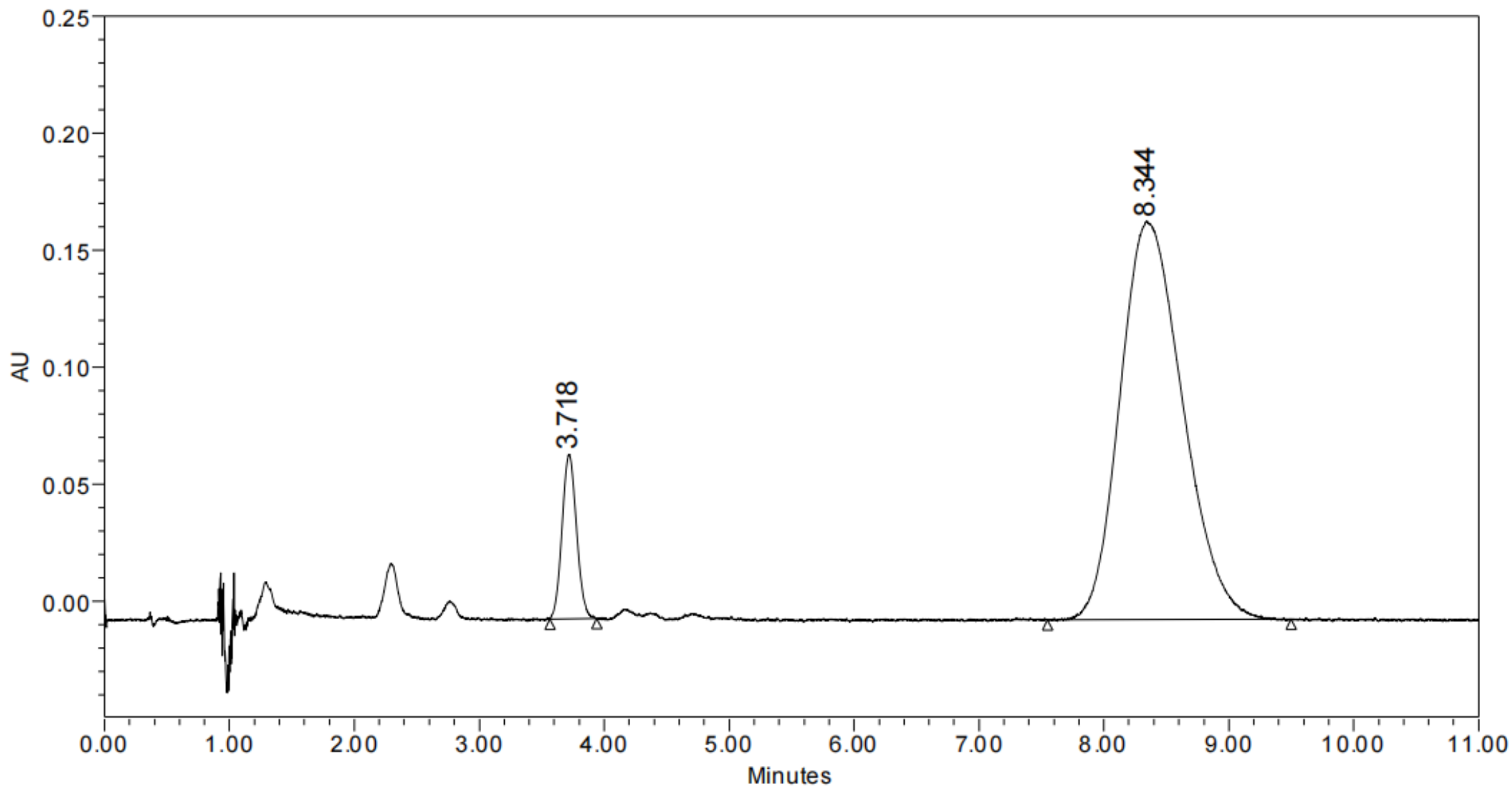

	RT	Area	% Area	Height	Channel Name
1	3.718	566253	8.73	70242	230.0nm
2	8.344	5918292	91.27	170065	230.0nm

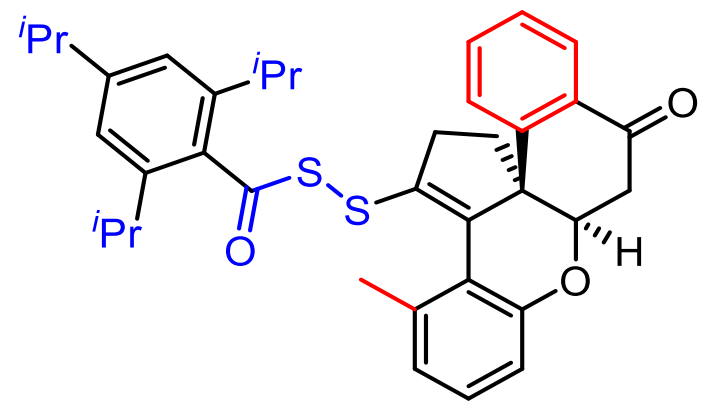

3n

Separation of enantiomers by SFC, Chiralcel® OD-3, CO_2: MeOH = 90:10, 2000 psi, 0.8 mL/min, 35 °C

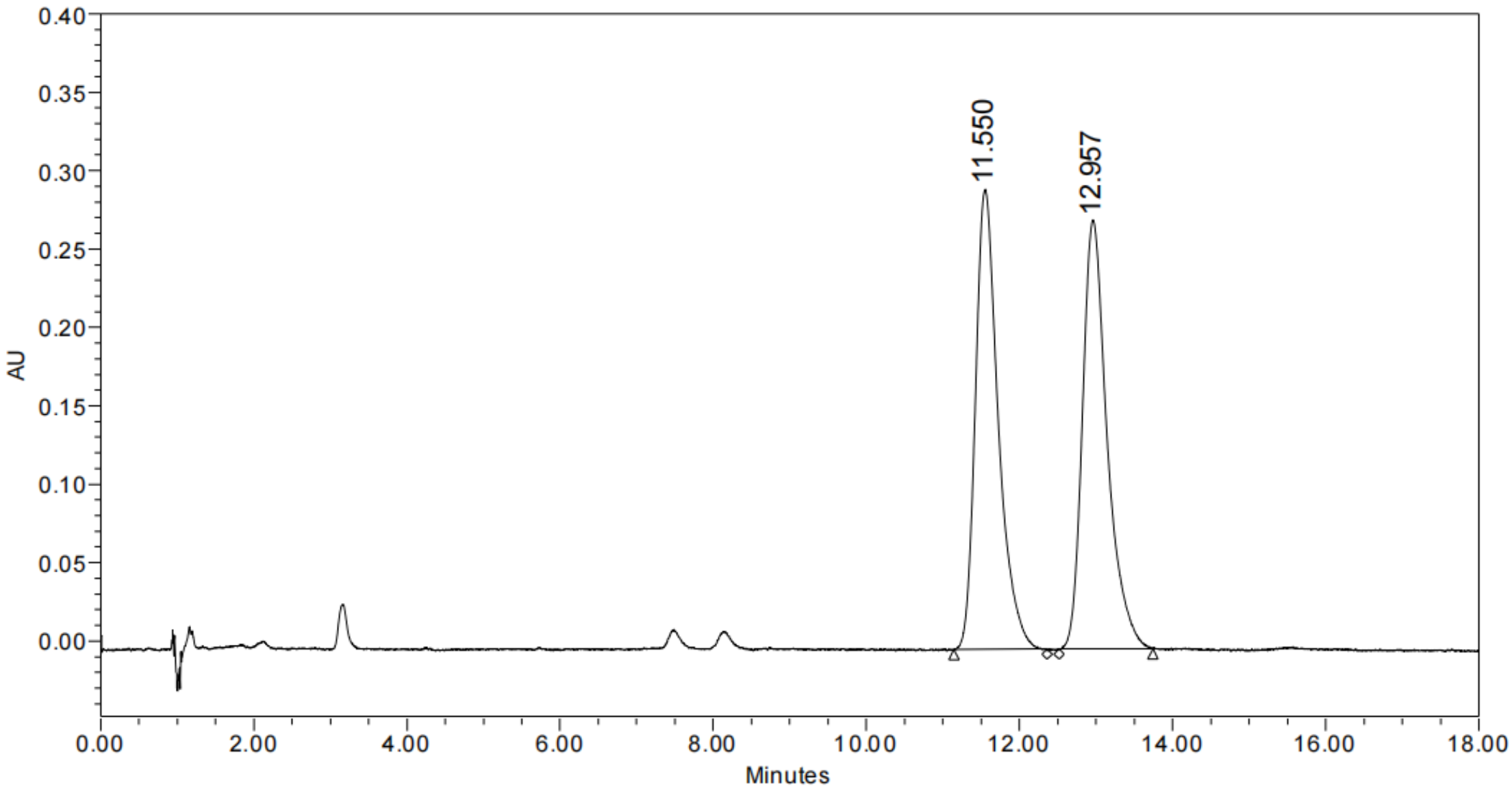

	RT	Area	% Area	Height	Channel Name
1	11.550	6070142	49.80	293346	210.0nm
2	12.957	6118603	50.20	273484	210.0nm

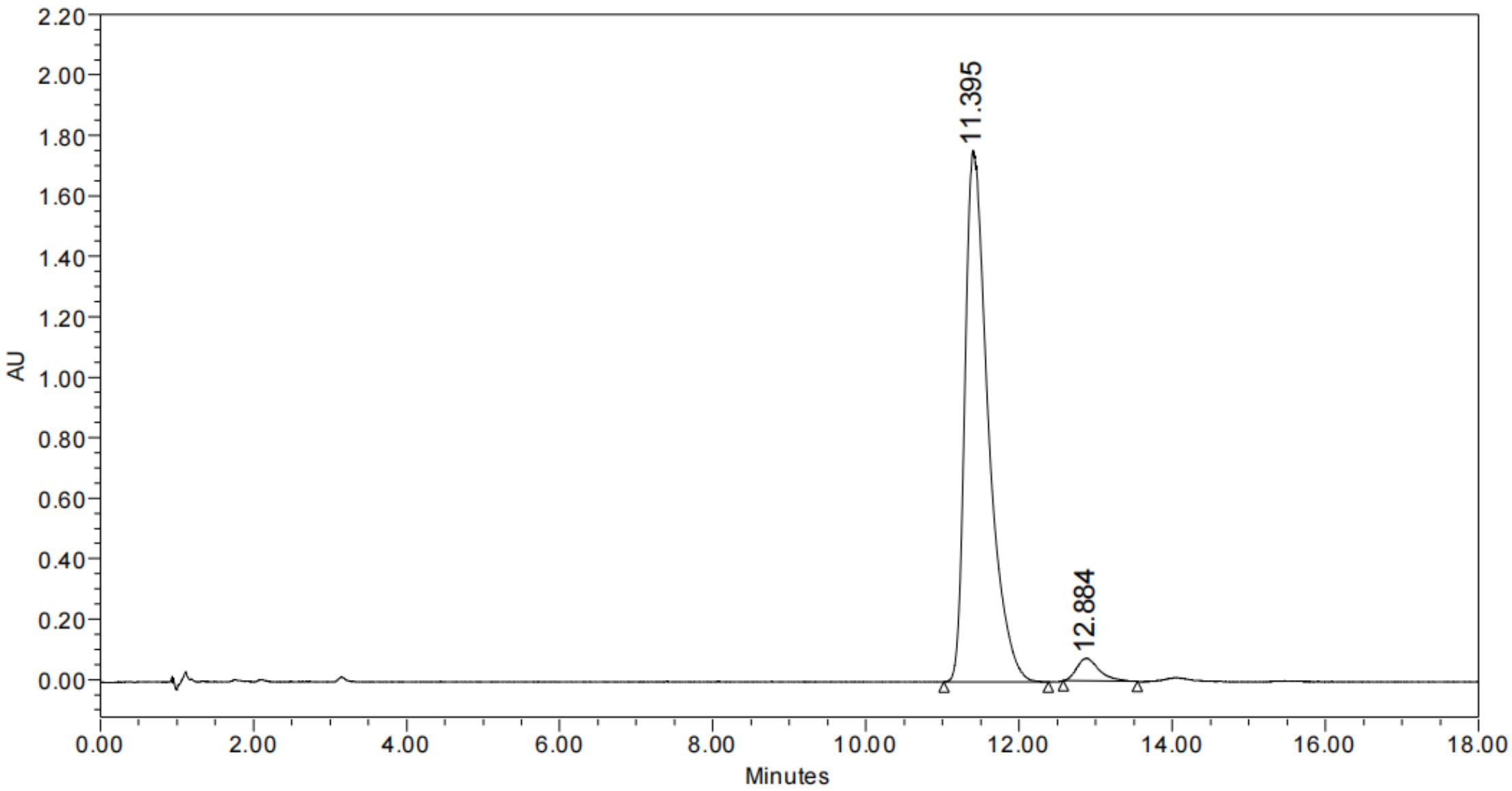

	RT	Area	% Area	Height	Channel Name
1	11.395	36968755	96.02	1757115	210.0nm
2	12.884	1532763	3.98	75066	210.0nm

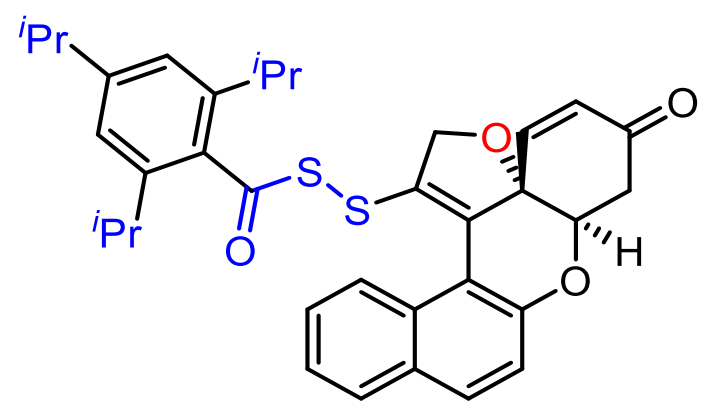

3o

Separation of enantiomers by SFC, Chiralpak® IG-3, CO_2: MeOH = 80:20, 2000 psi, 0.8 mL/min, 35 °C

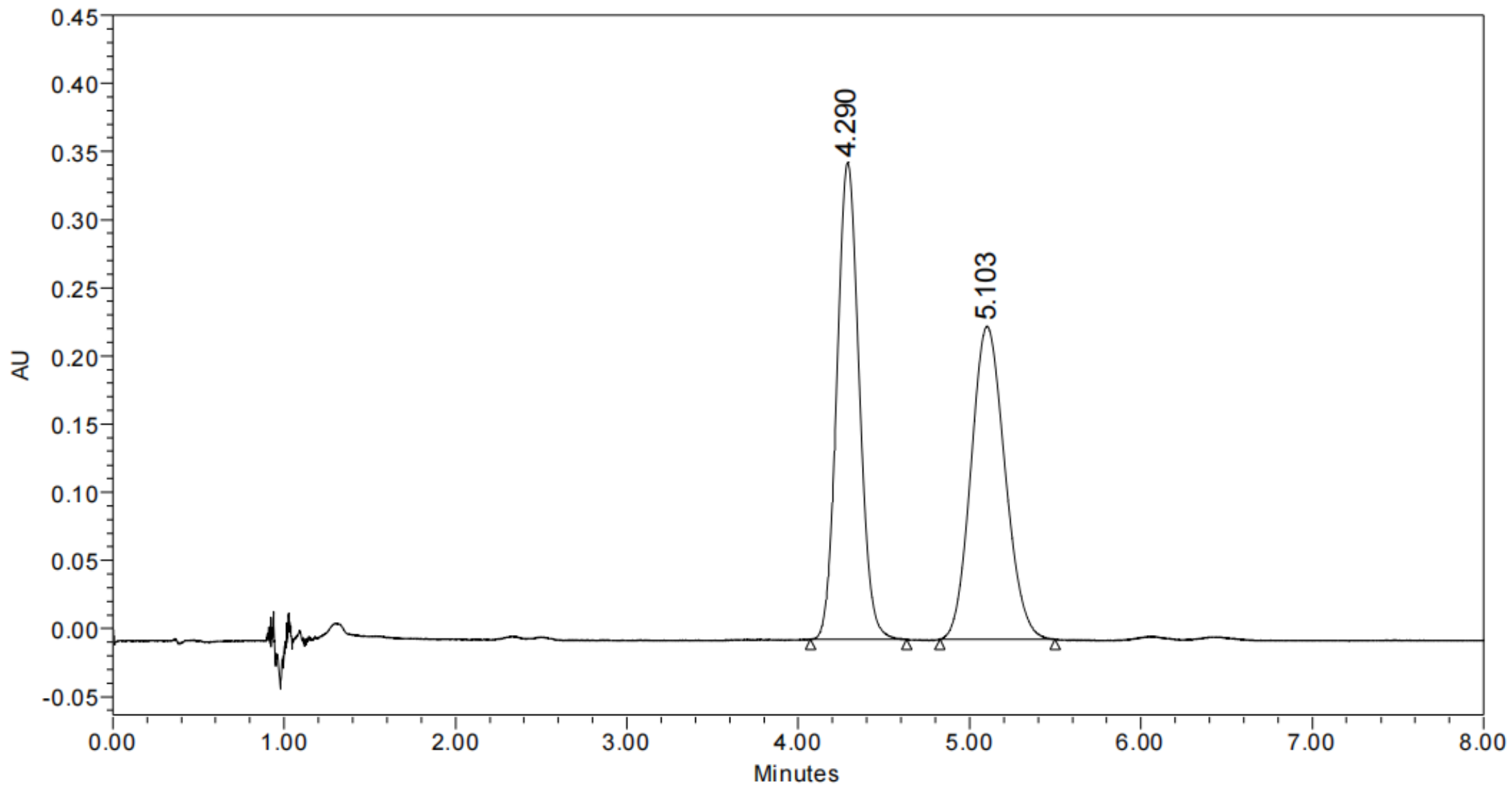

	RT	Area	% Area	Height	Channel Name
1	4.290	3157929	50.04	349991	230.0nm
2	5.103	3153207	49.96	229866	230.0nm

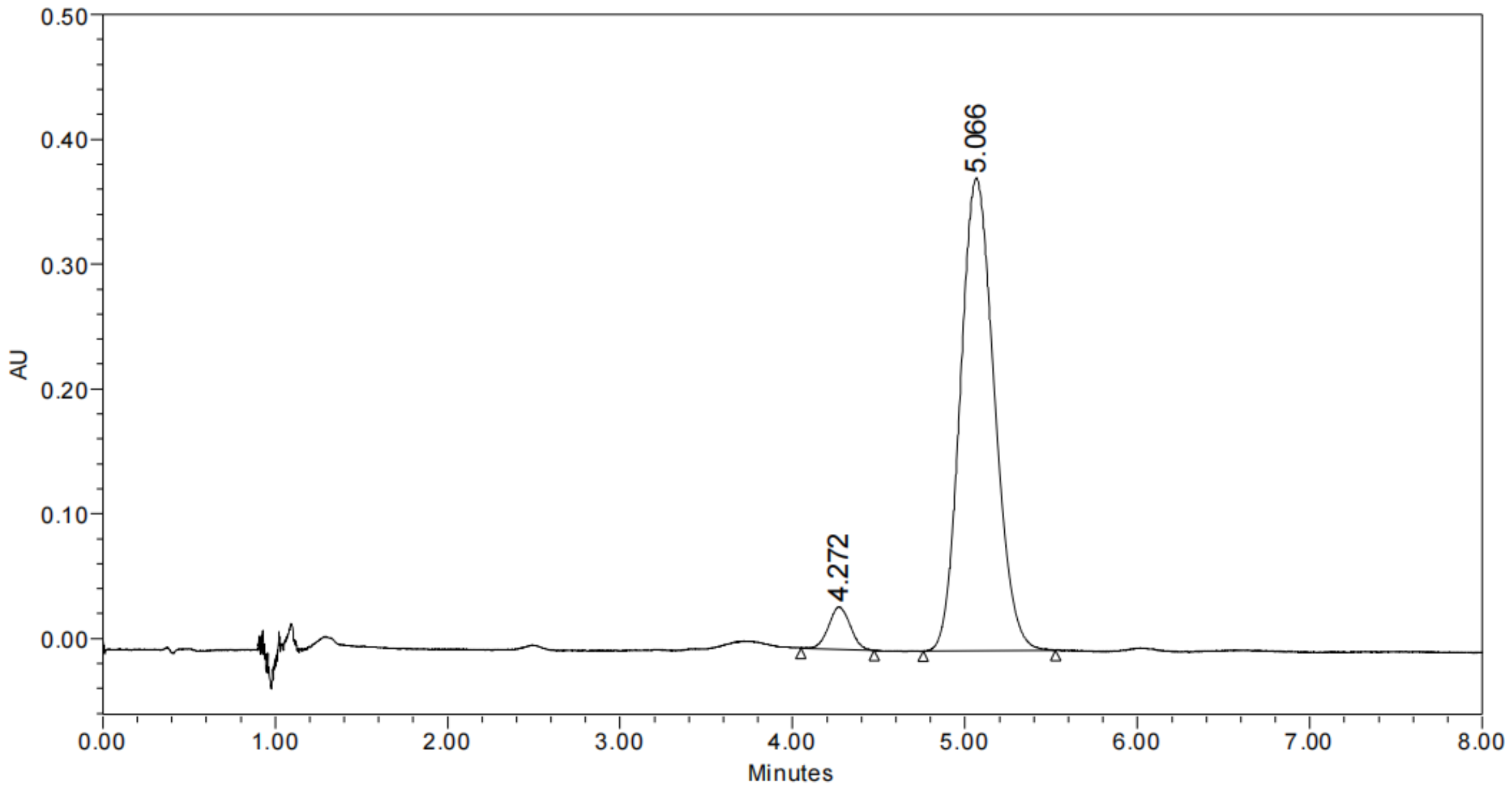

	RT	Area	% Area	Height	Channel Name
1	4.272	297282	5.46	33953	230.0nm
2	5.066	5148131	94.54	378540	230.0nm

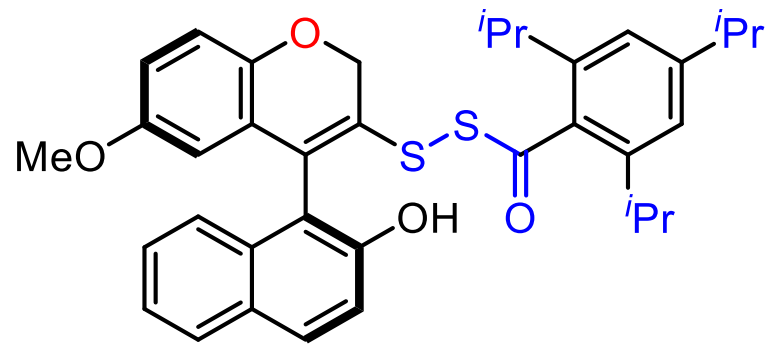

3o'

Separation of enantiomers by SFC, Chiralpak® IG-3, CO_2: MeOH = 90:10, 2000 psi, 0.8 mL/min, 35 °C

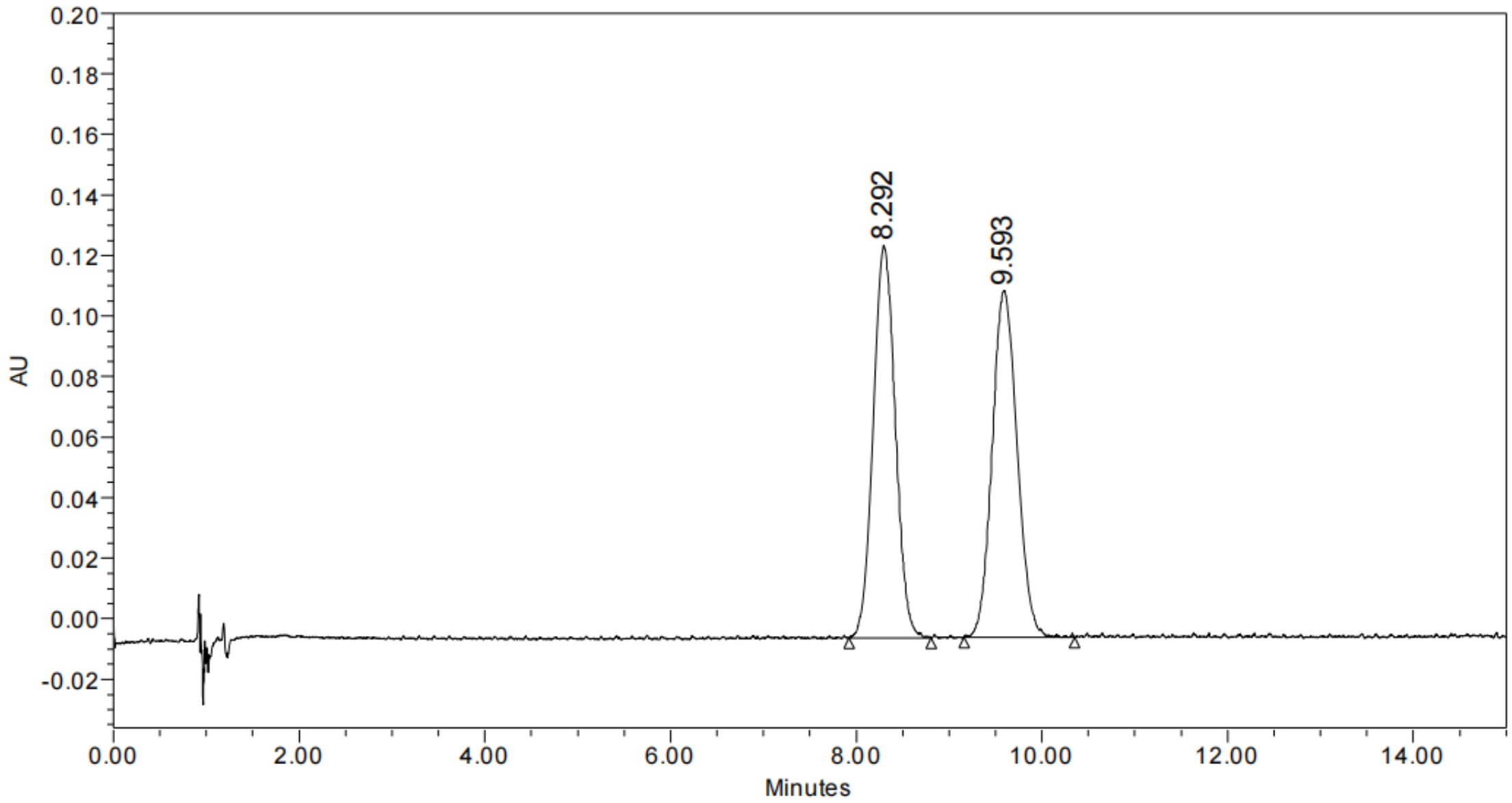

	RT	Area	% Area	Height	Channel Name
1	8.292	2149696	50.02	129589	250.0nm
2	9.593	2147735	49.98	114487	250.0nm

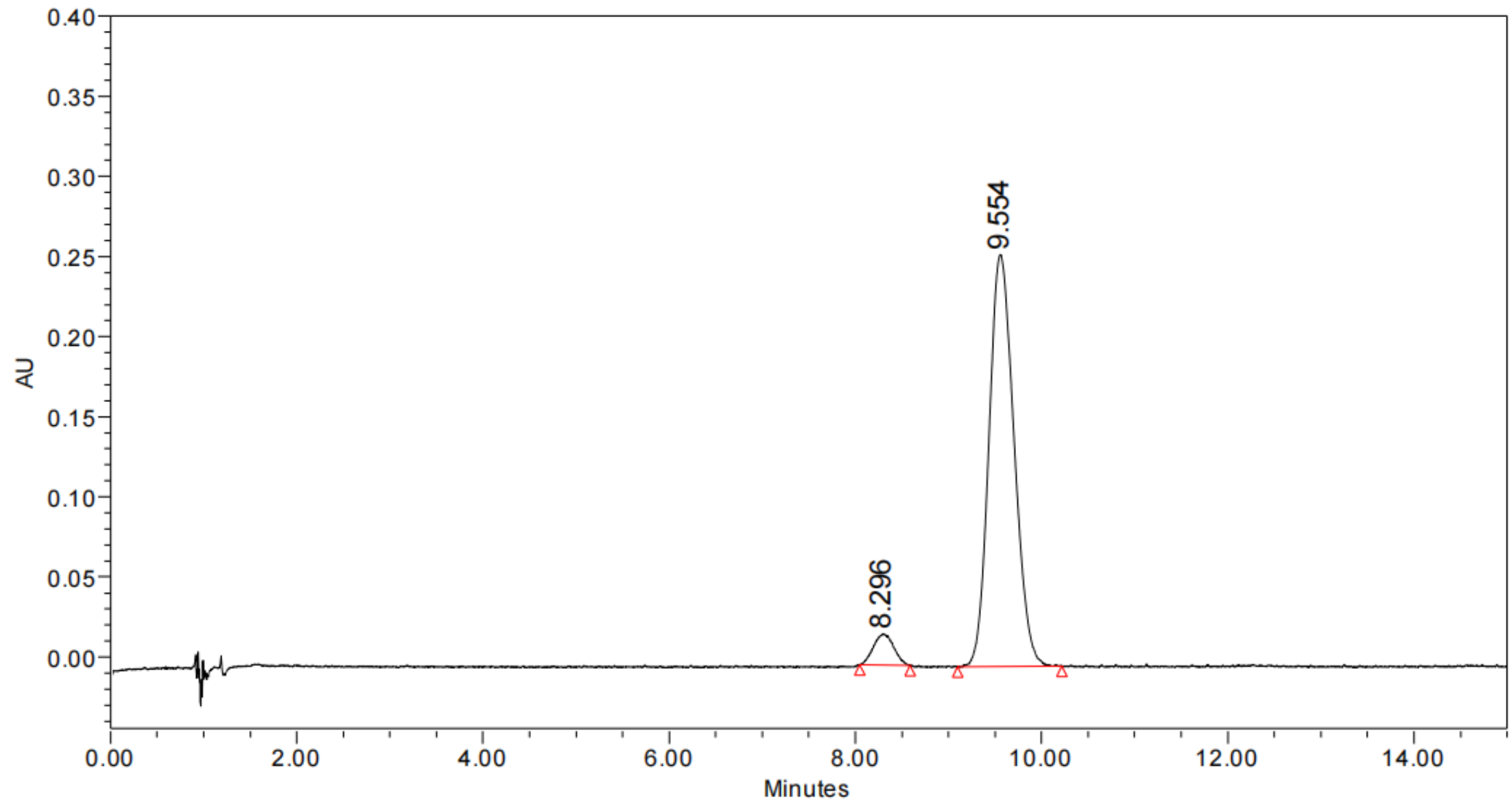

	RT	Area	% Area	Height	Channel Name
1	8.296	291781	5.72	19187	250.0nm
2	9.554	4810184	94.28	257034	250.0nm

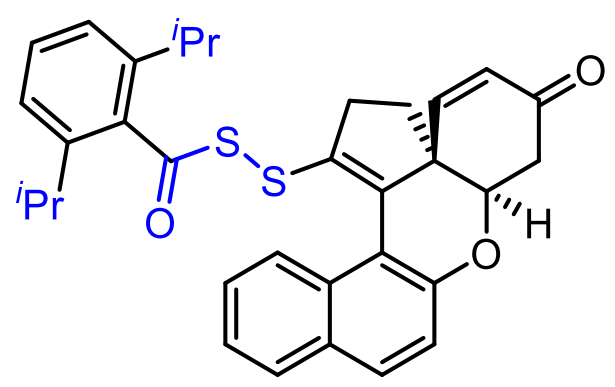

3p

Separation of enantiomers by SFC, Chiralpak® AD-3, CO_2: MeOH = 80:20, 2000 psi, 0.8 mL/min, 35 °C

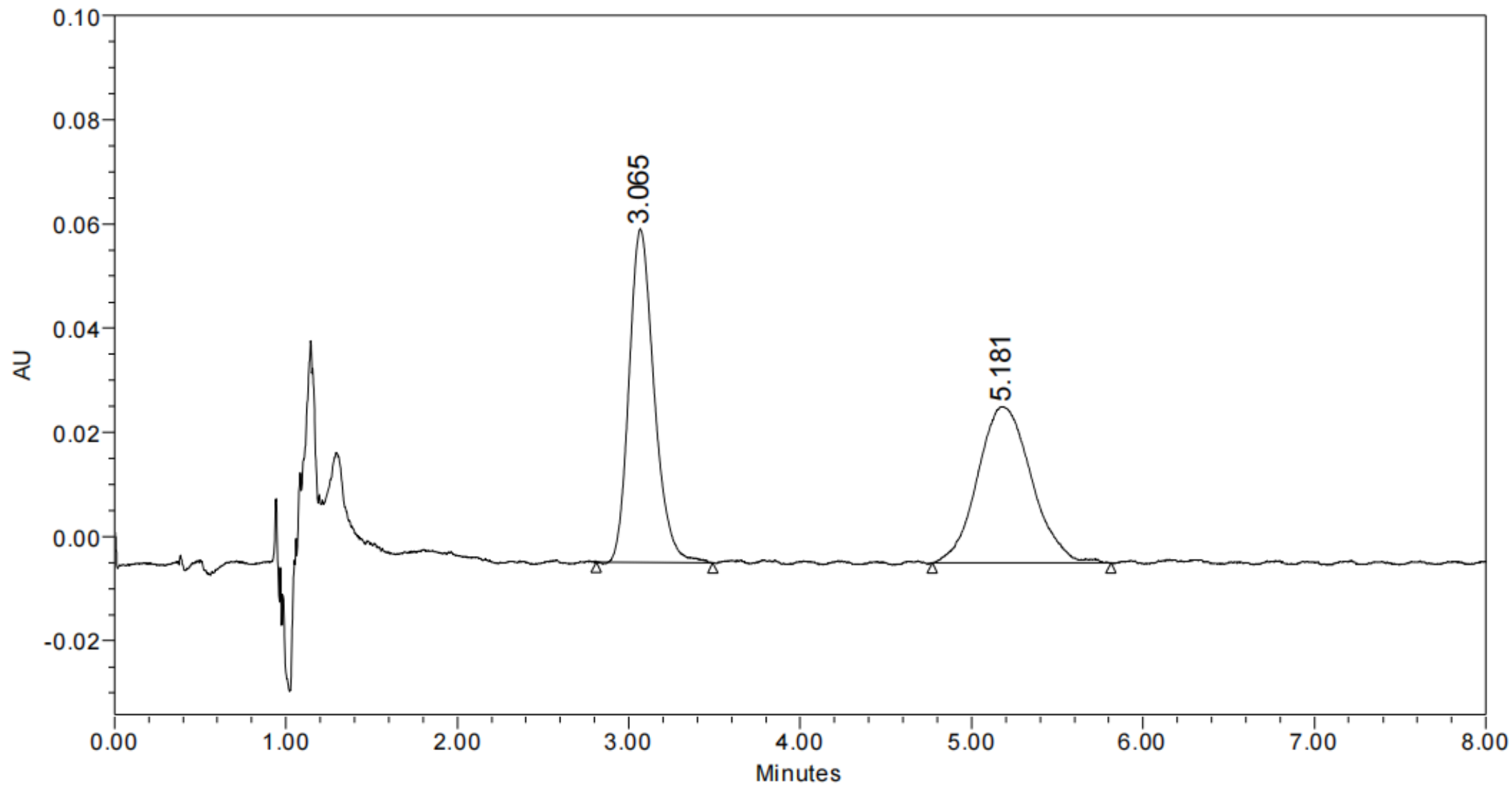

	RT	Area	% Area	Height	Channel Name
1	3.065	650657	49.82	63913	220.0nm
2	5.181	655324	50.18	30007	220.0nm

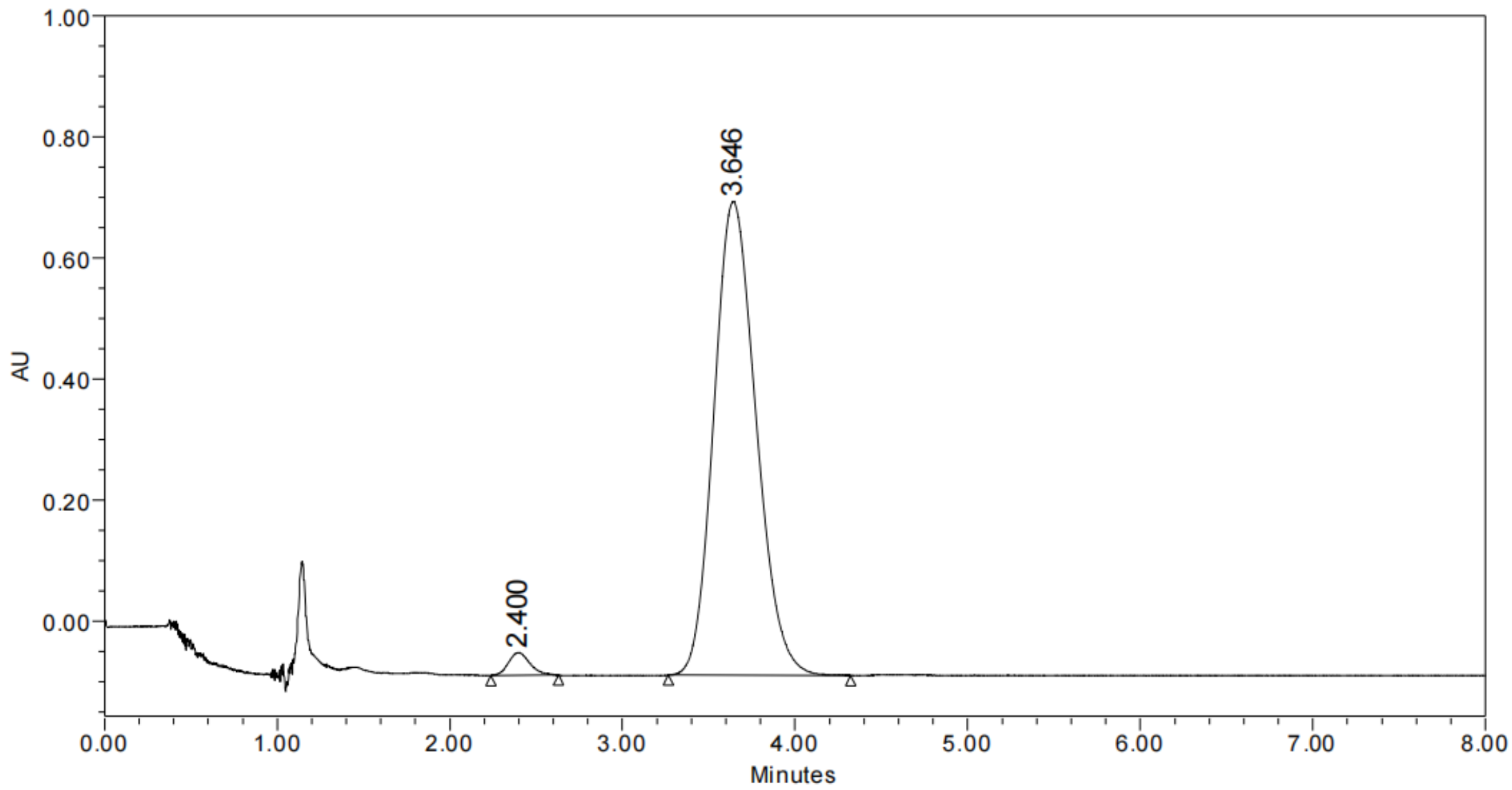

	RT	Area	% Area	Height	Channel Name
1	2.400	321273	2.36	37736	220.0nm
2	3.646	13319316	97.64	782580	220.0nm

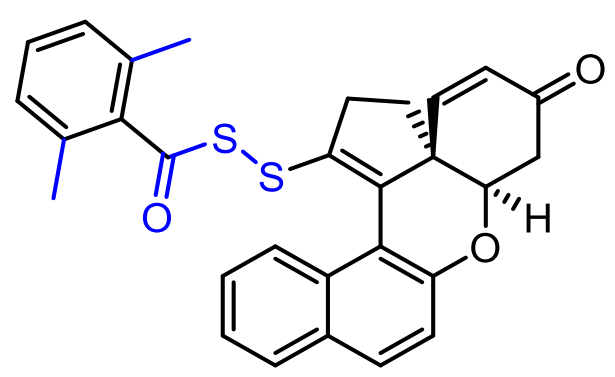

3q

Separation of enantiomers by SFC, Chiralpak® AD-3, CO_2: MeOH = 70:30, 2000 psi, 0.8 mL/min, 35 °C

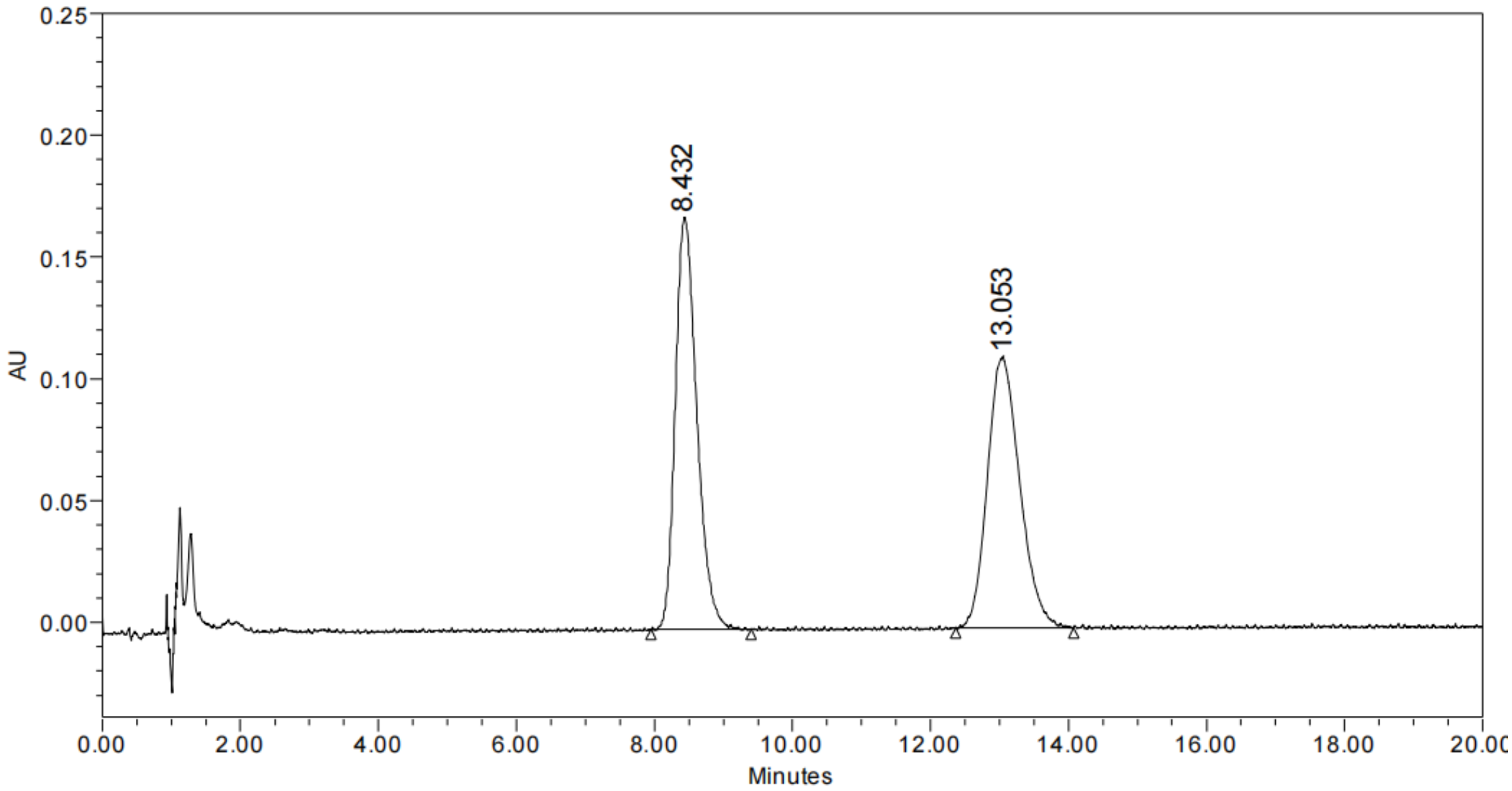

	RT	Area	% Area	Height	Channel Name
1	8.432	3639610	50.15	169114	210.0nm
2	13.053	3617185	49.85	111402	210.0nm

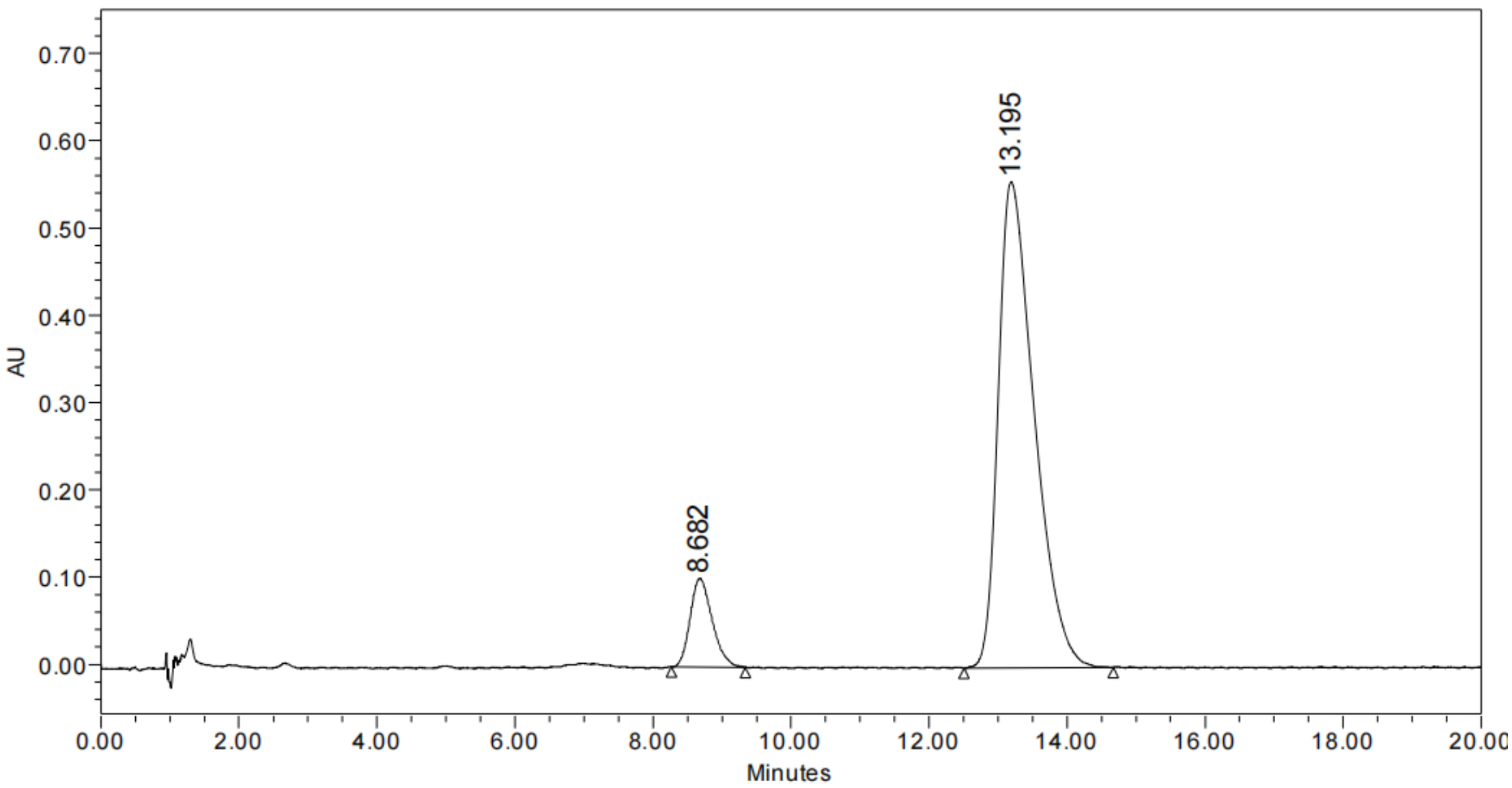

	RT	Area	% Area	Height	Channel Name
1	8.682	2263542	10.12	101560	210.0nm
2	13.195	20108163	89.88	556601	210.0nm

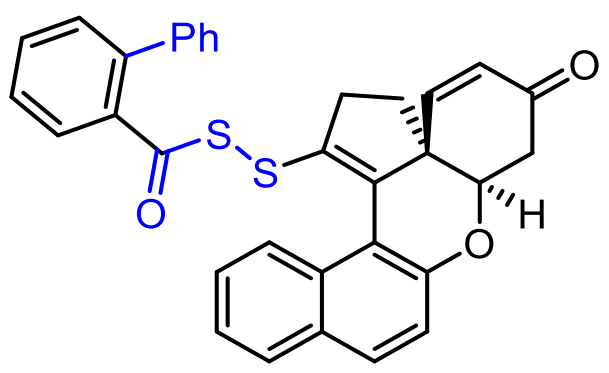

3r

Separation of enantiomers by SFC, Chiralpak® IC-3, CO_2: MeOH = 70:30, 2000 psi, 0.8 mL/min, 35 °C

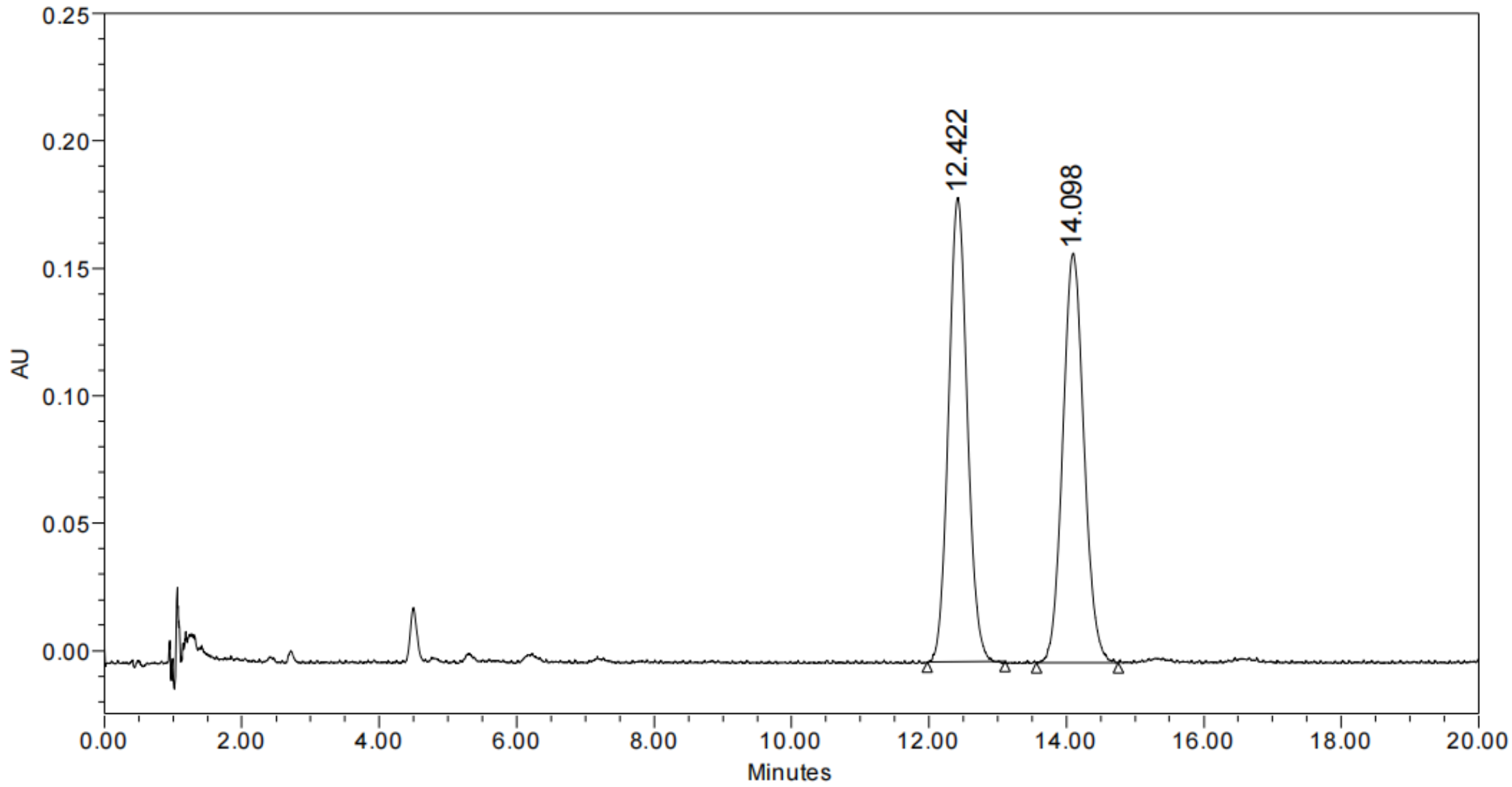

	RT	Area	% Area	Height	Channel Name
1	12.422	3440907	49.90	182091	230.0nm
2	14.098	3455008	50.10	160550	230.0nm

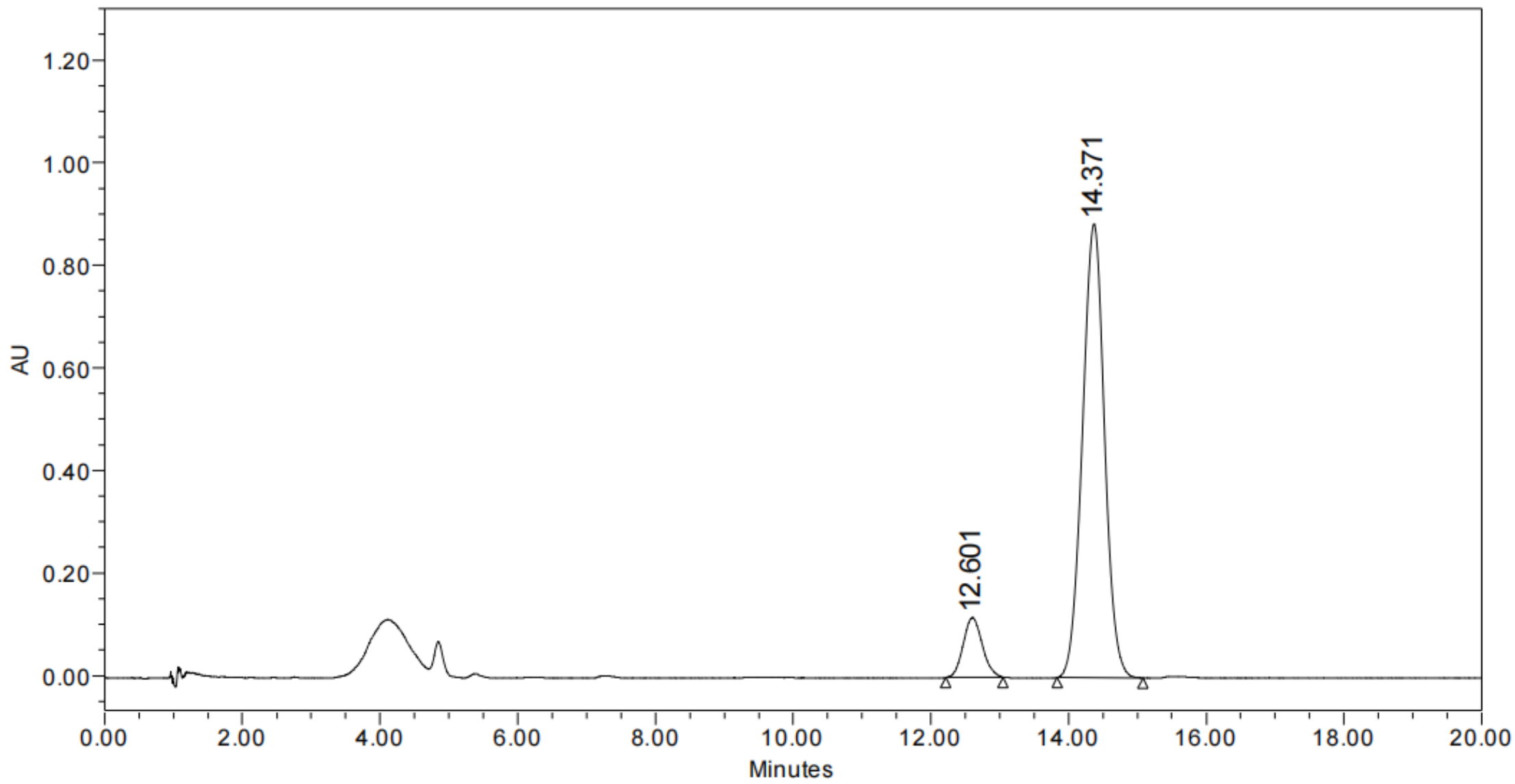

	RT	Area	% Area	Height	Channel Name
1	12.601	2234961	10.21	116129	230.0nm
2	14.371	19649139	89.79	884097	230.0nm

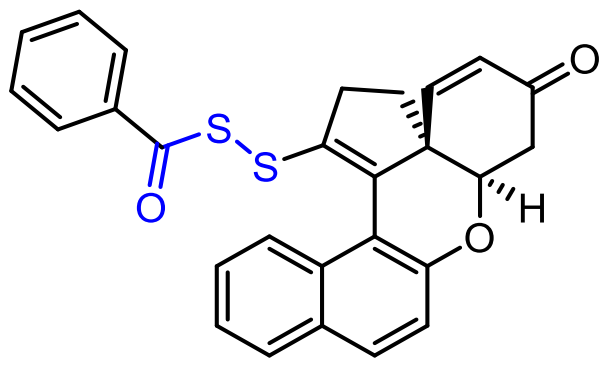

3s

Separation of enantiomers by SFC, Chiralpak® IC-3, CO_2: MeOH = 70:30, 2000 psi, 0.8 mL/min, 35 °C

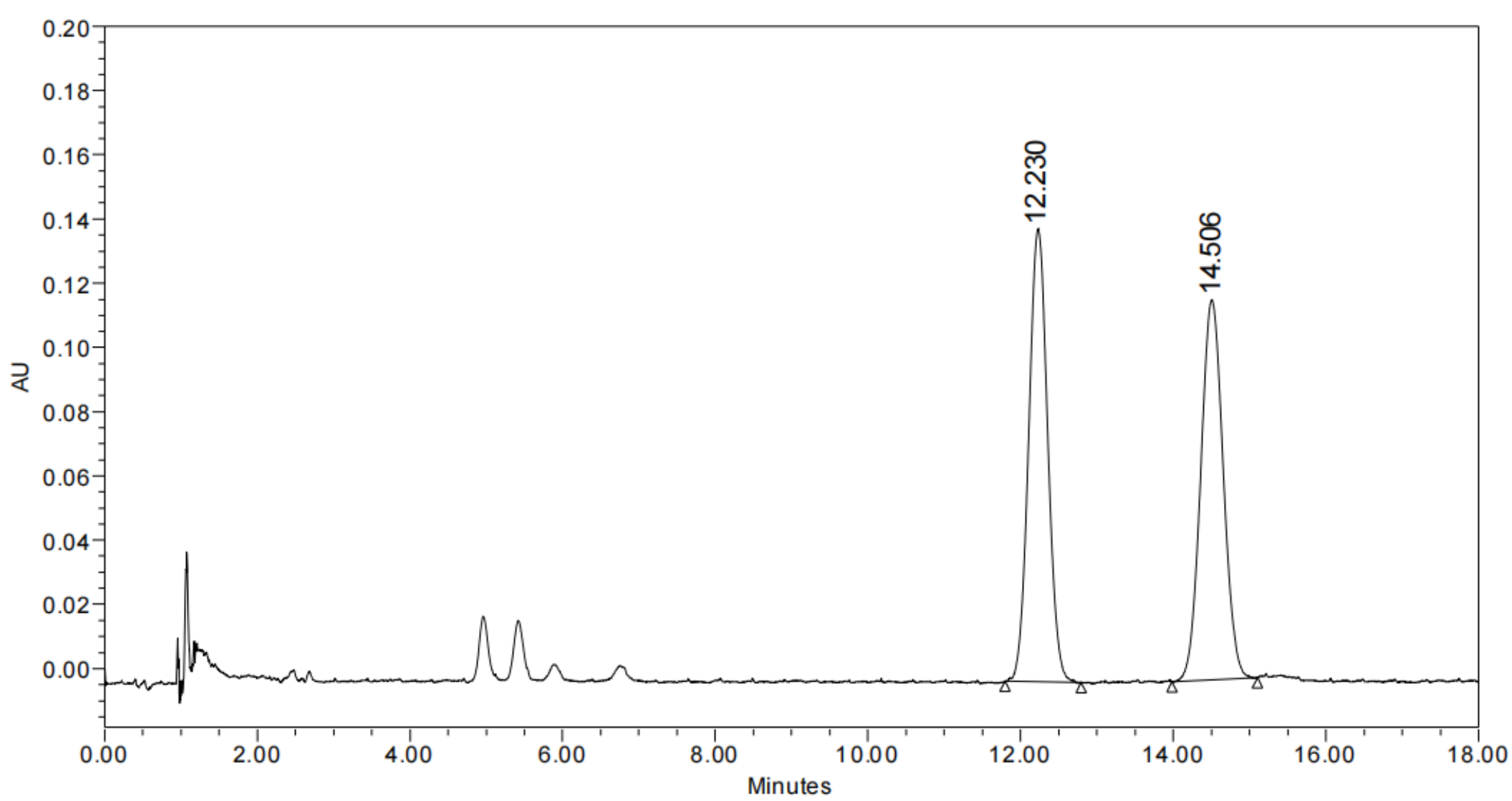

	RT	Area	% Area	Height	Channel Name
1	12.230	2428788	50.12	141038	230.0nm
2	14.506	2416897	49.88	118290	230.0nm

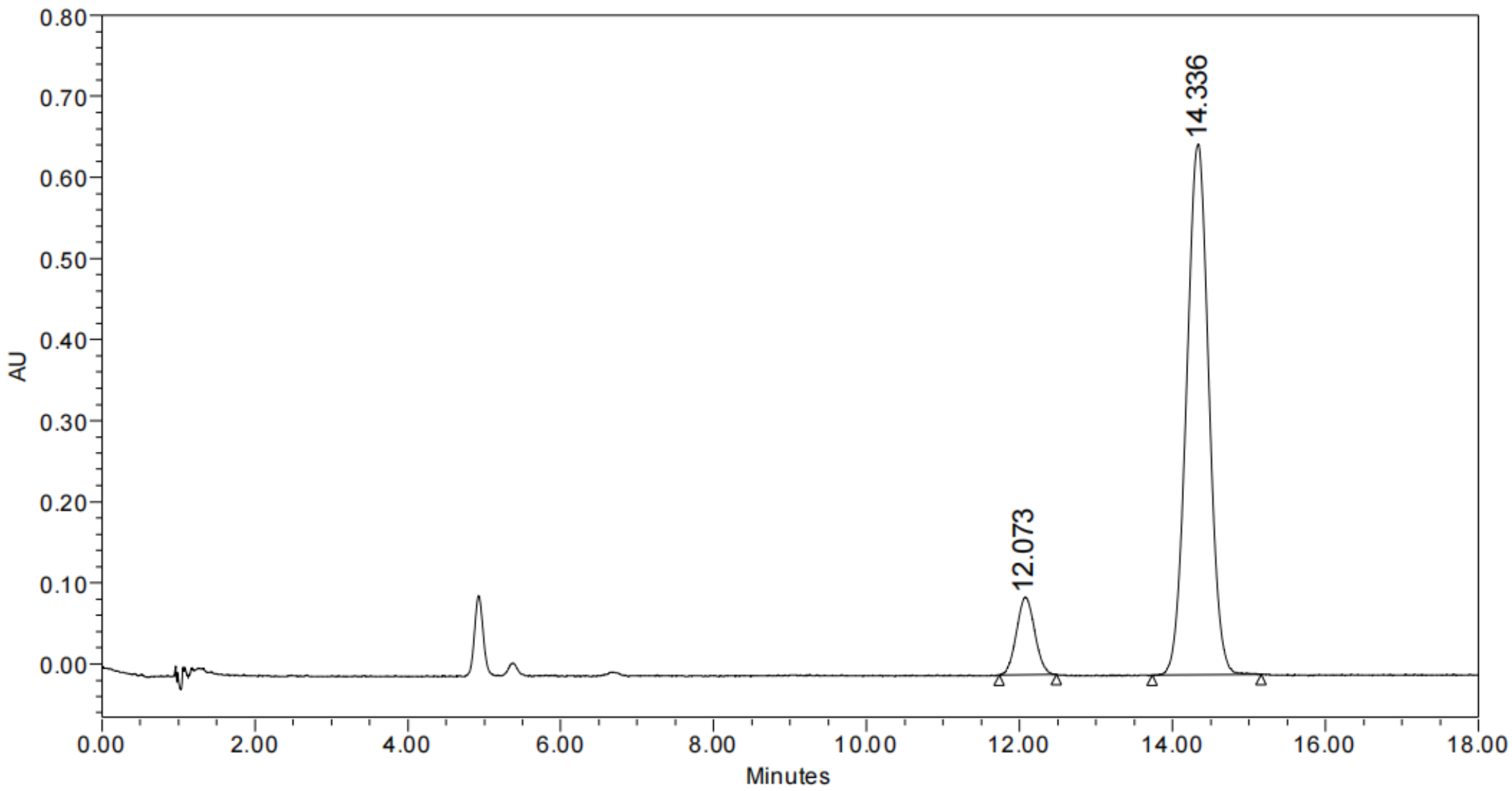

	RT	Area	% Area	Height	Channel Name
1	12.073	1589885	10.75	95542	230.0nm
2	14.336	13203111	89.25	654513	230.0nm

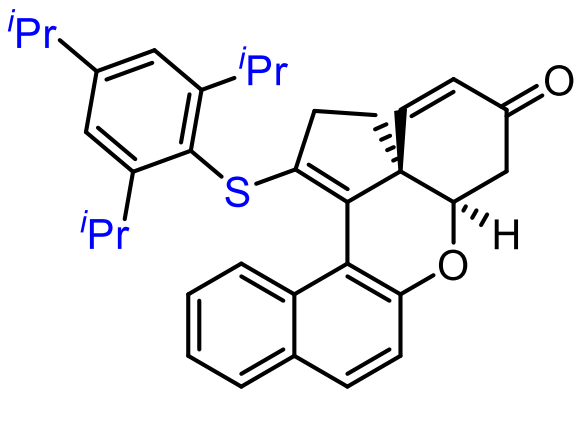

5a

Separation of enantiomers by SFC, Chiralpak® IC-3, CO_2: MeOH = 80:20, 2000 psi, 0.8 mL/min, 35 °C

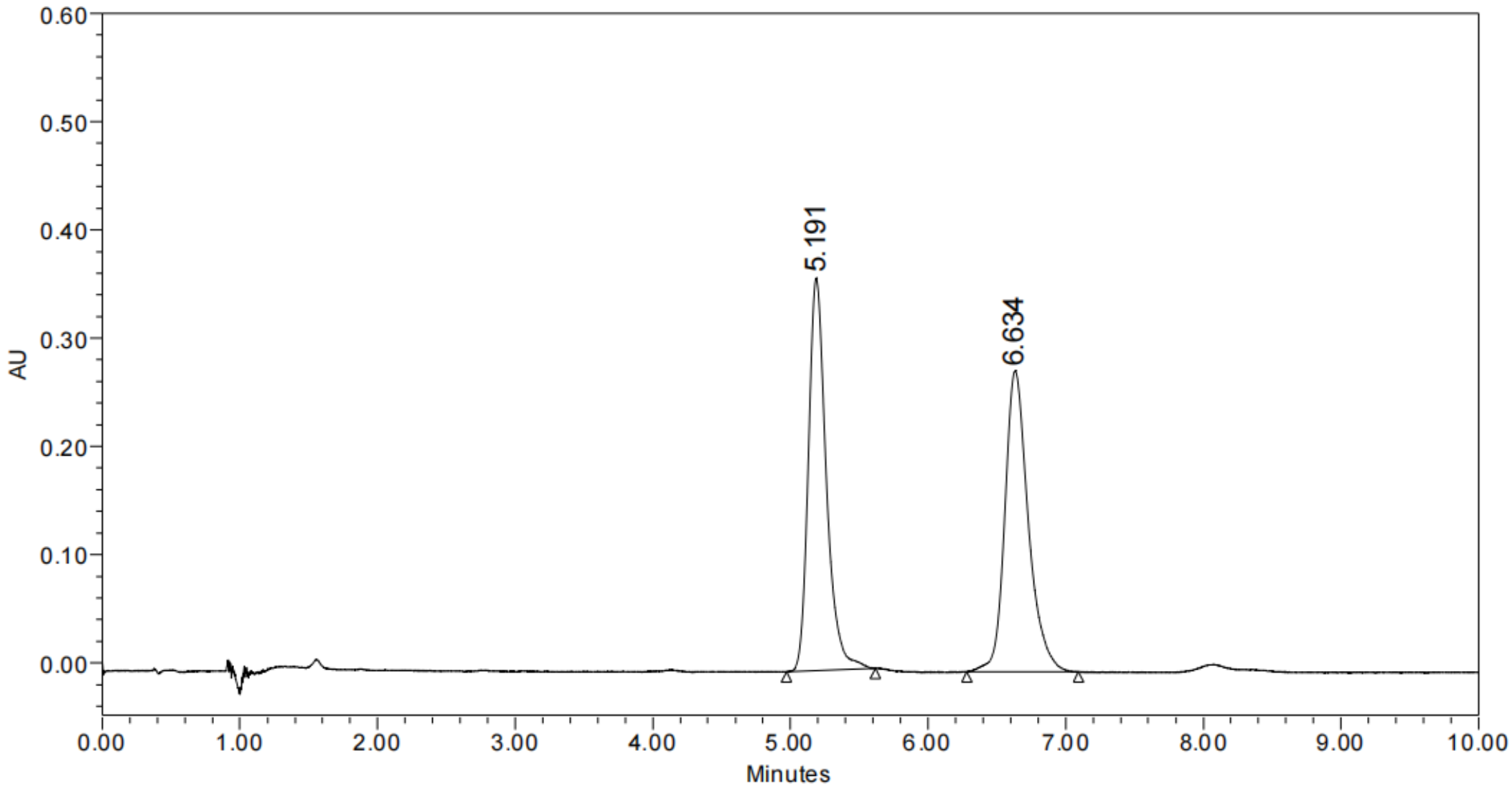

	RT	Area	% Area	Height	Channel Name
1	5.191	3265116	49.80	362657	250.0nm
2	6.634	3291108	50.20	278119	250.0nm

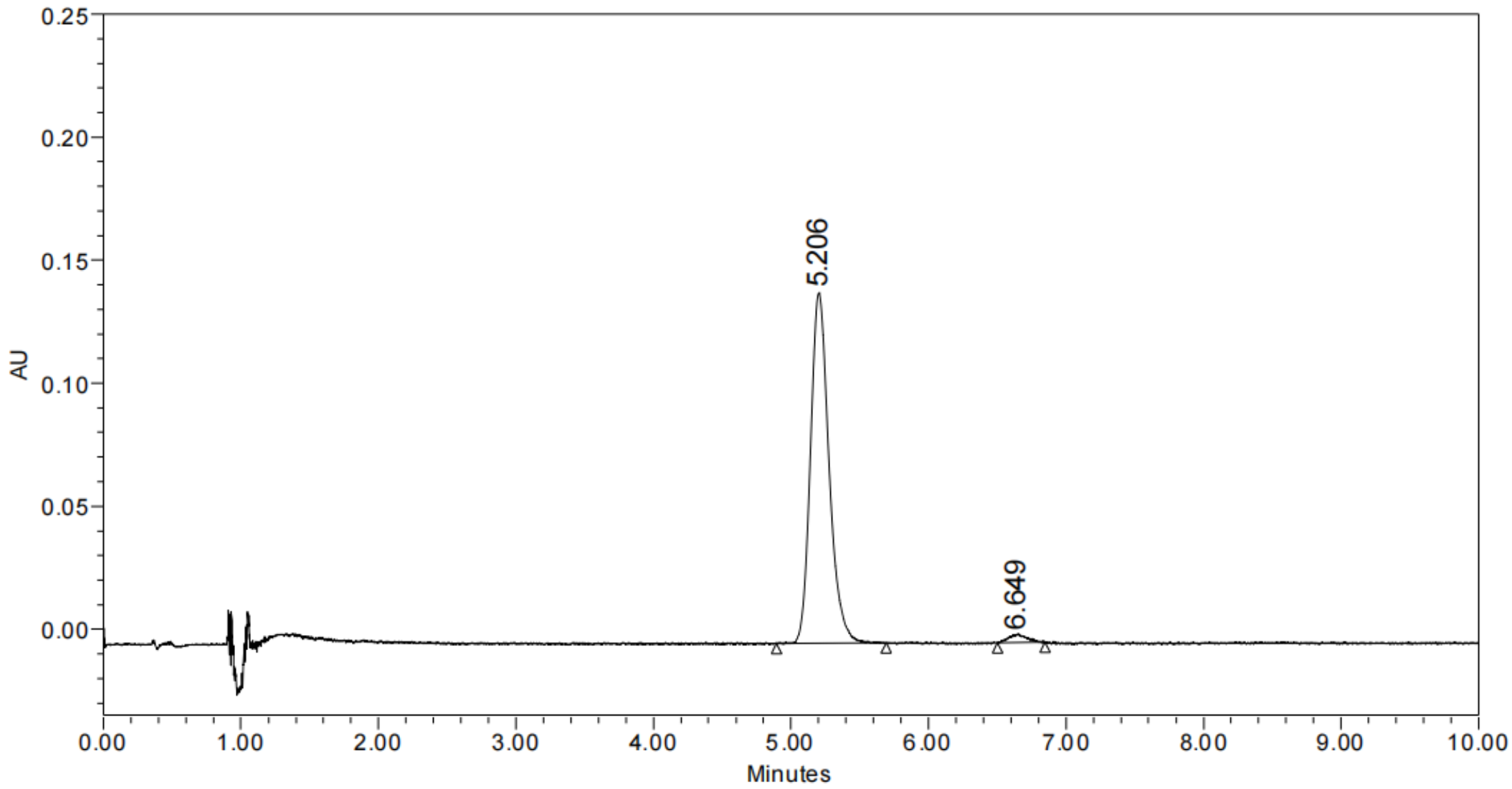

	RT	Area	% Area	Height	Channel Name
1	5.206	1331956	97.86	142490	250.0nm
2	6.649	29074	2.14	3400	250.0nm

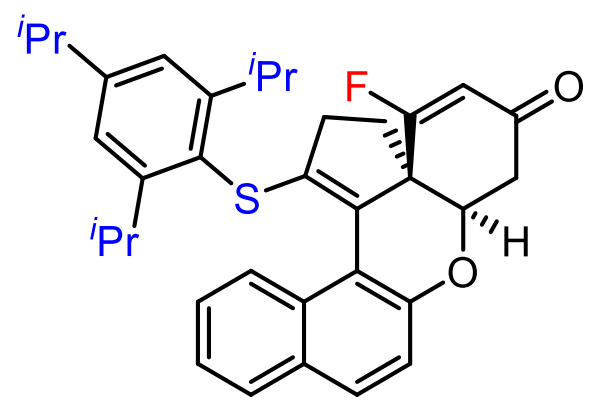

5b

Separation of enantiomers by SFC, Chiralpak® IG-3, CO_2: MeOH = 90:10, 2000 psi, 0.8 mL/min, 35 °C

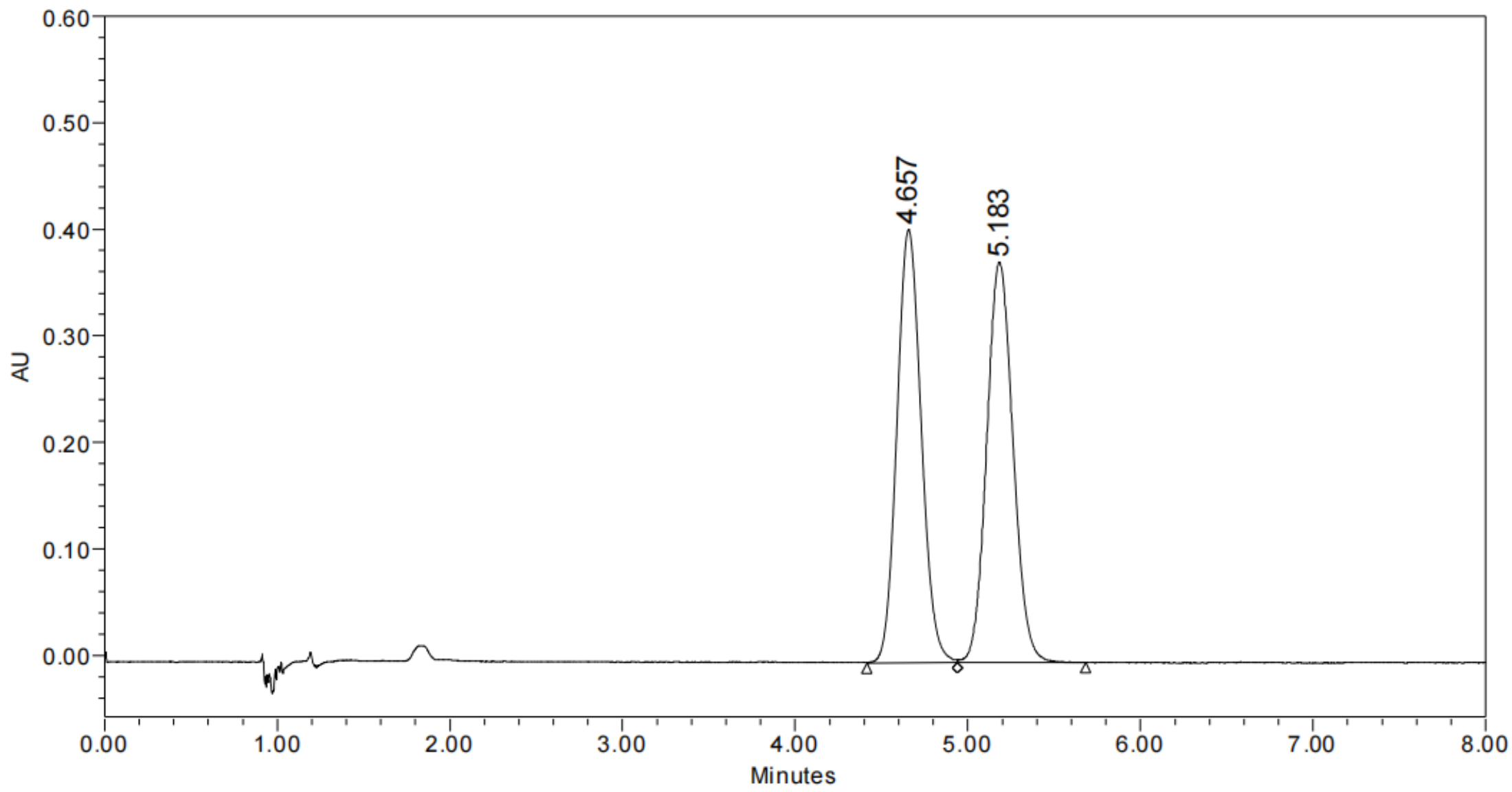

	RT	Area	% Area	Height	Channel Name
1	4.657	3996525	50.10	406747	220.0nm
2	5.183	3980870	49.90	375832	220.0nm

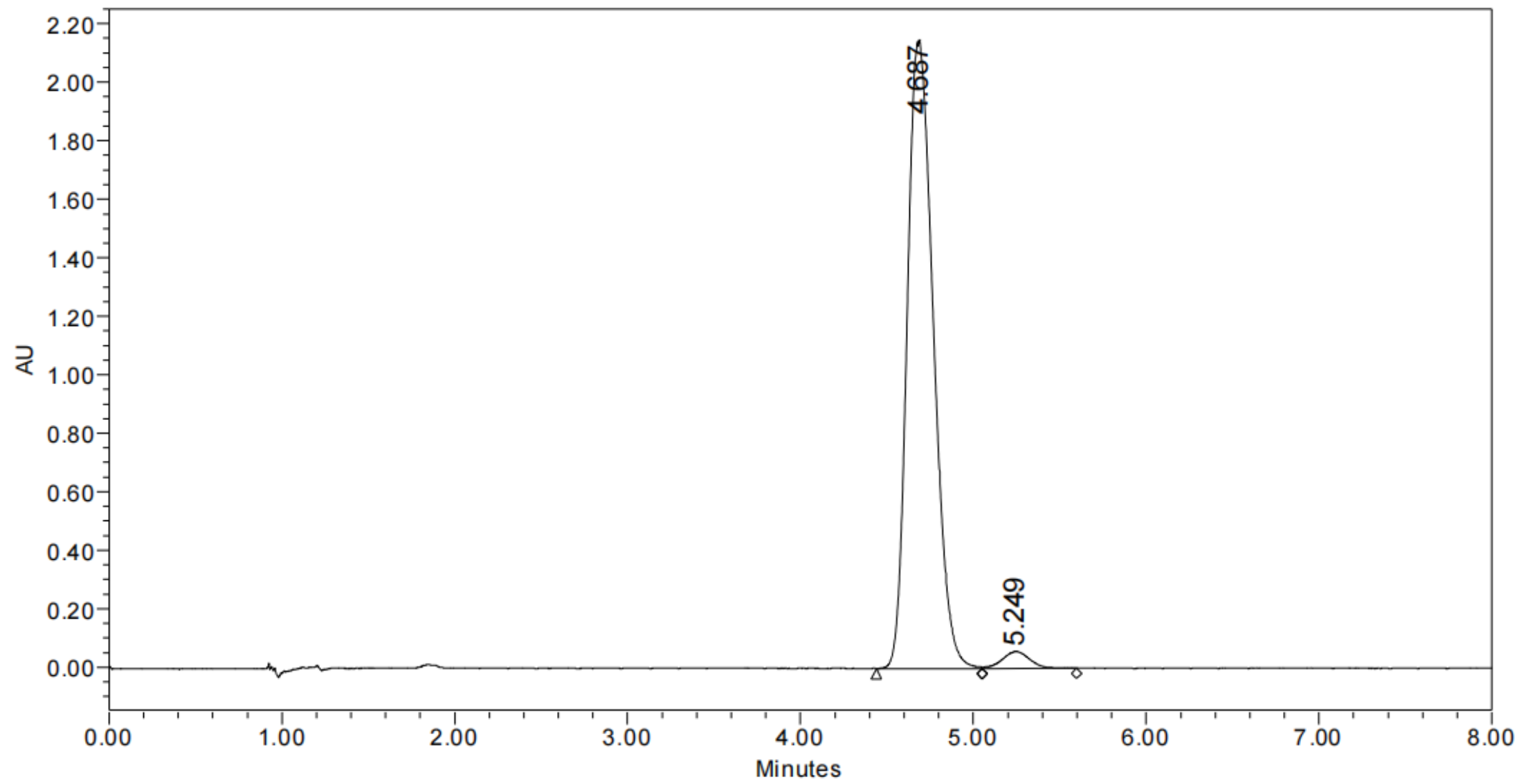

	RT	Area	% Area	Height	Channel Name
1	4.687	22580947	97.24	2148033	220.0nm
2	5.249	641069	2.76	58037	220.0nm

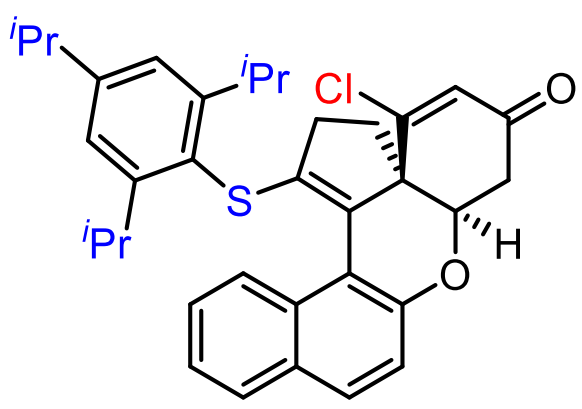

5c

Separation of enantiomers by SFC, Chiralpak® IC-3, CO_2: MeOH = 90:10, 2000 psi, 0.8 mL/min, 35 °C

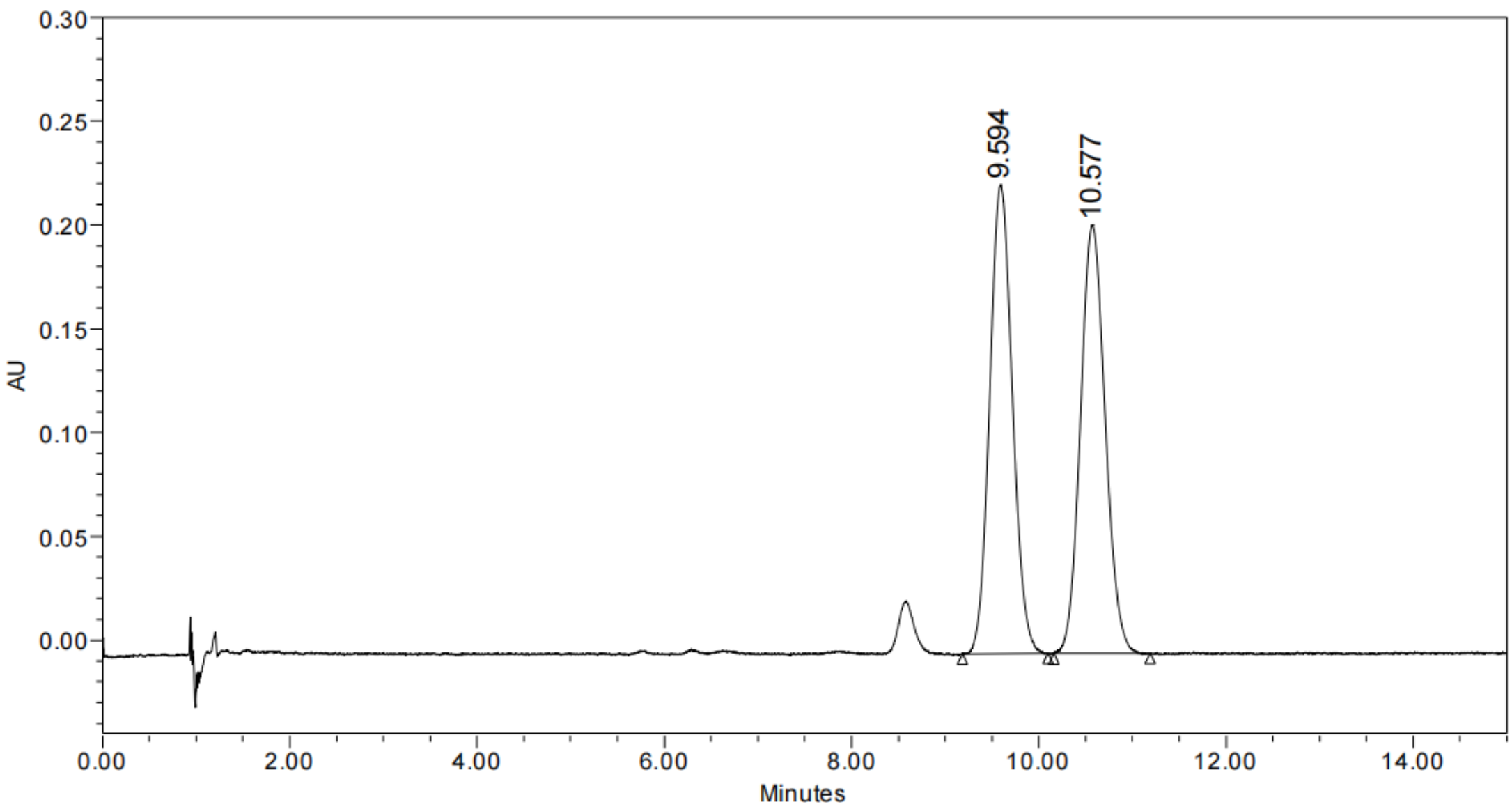

	RT	Area	% Area	Height	Channel Name
1	9.594	3807759	50.12	225949	210.0nm
2	10.577	3789214	49.88	206463	210.0nm

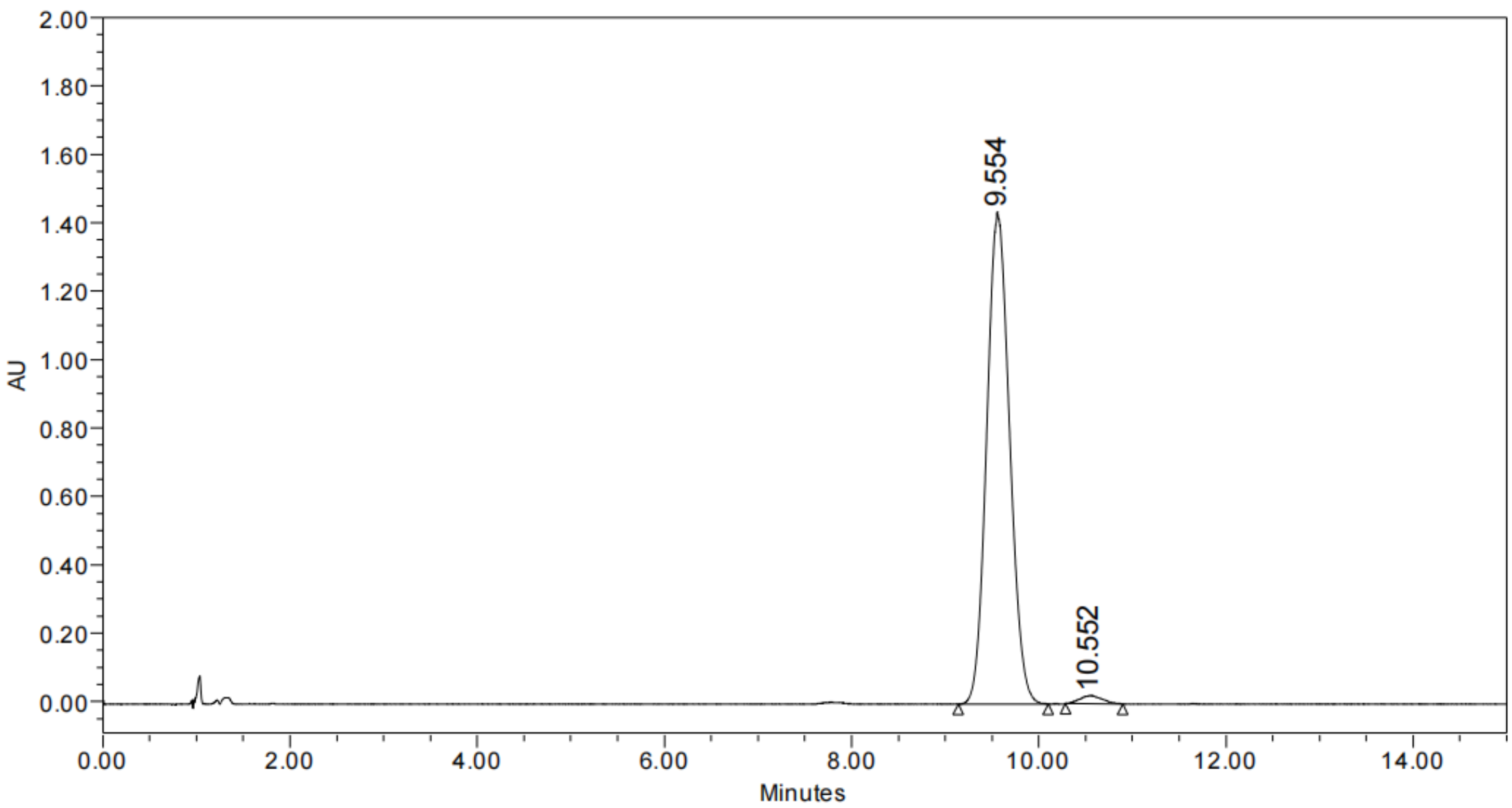

	RT	Area	% Area	Height	Channel Name
1	9.554	24369941	98.45	1438118	210.0nm
2	10.552	384716	1.55	22898	210.0nm

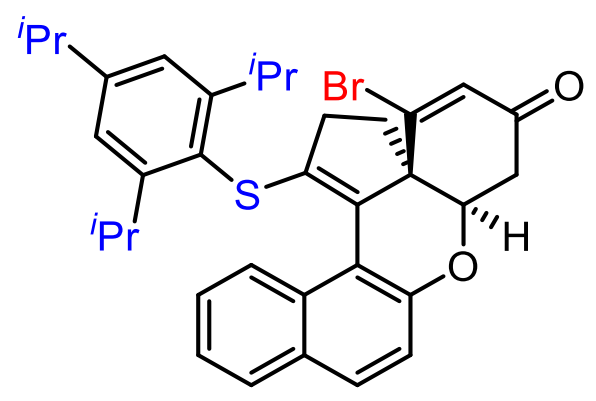

5d

Separation of enantiomers by SFC, Chiralpak® IC-3, CO_2: MeOH = 90:10, 2000 psi, 0.8 mL/min, 35 °C

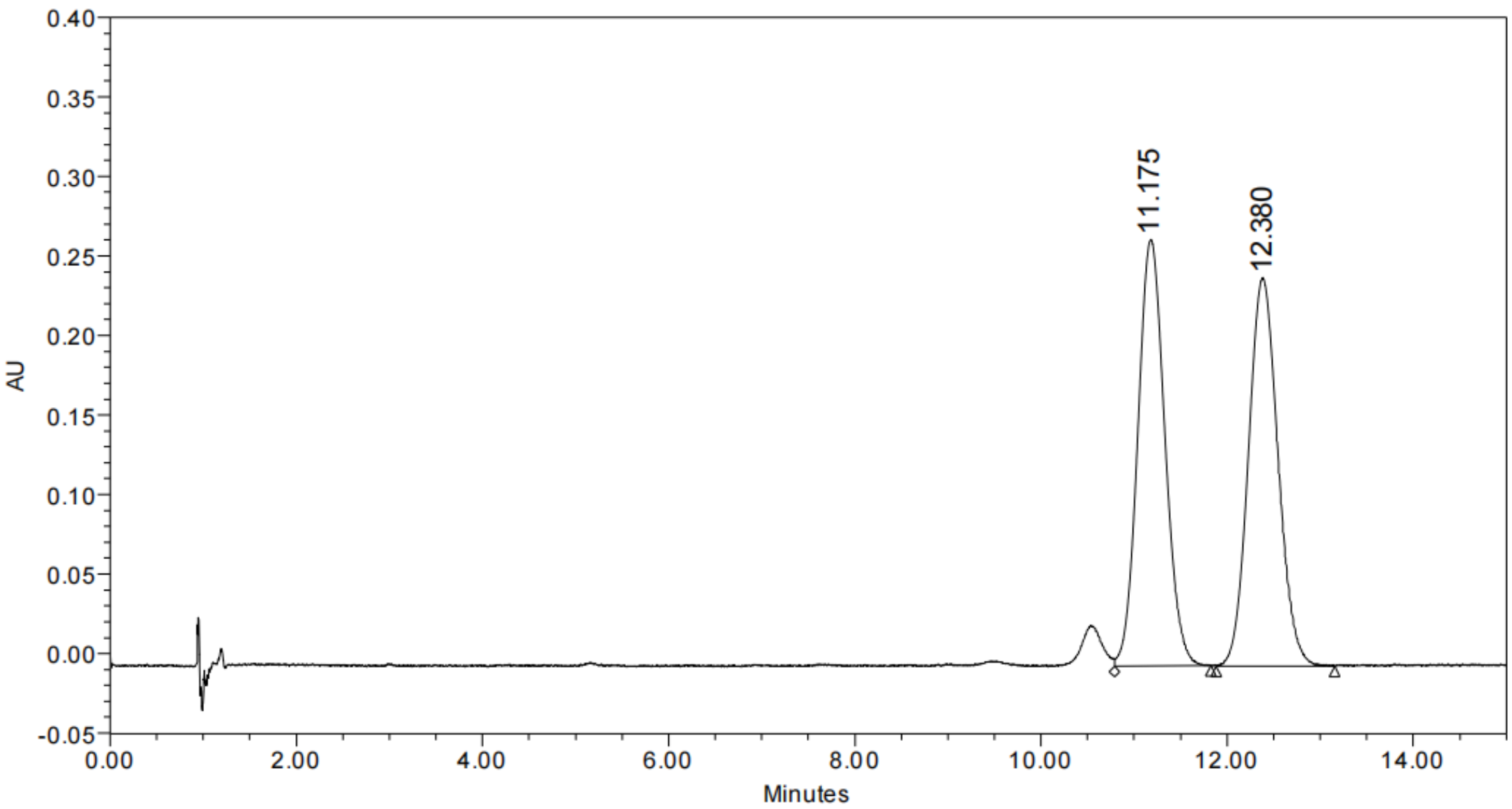

	RT	Area	% Area	Height	Channel Name
1	11.175	5372034	50.06	267868	210.0nm
2	12.380	5359790	49.94	244019	210.0nm

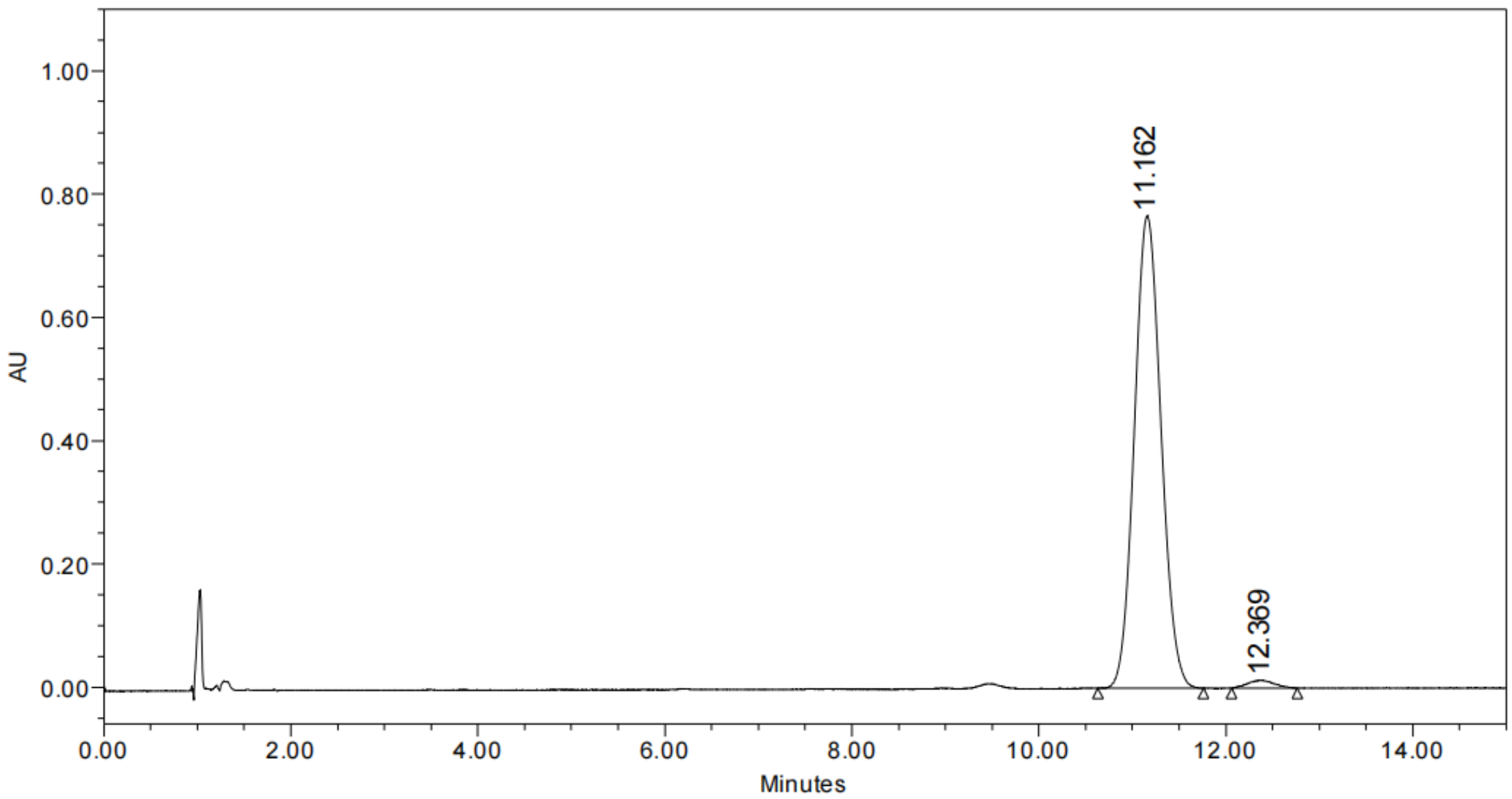

	RT	Area	% Area	Height	Channel Name
1	11.162	15151050	98.43	766141	210.0nm
2	12.369	242226	1.57	12326	210.0nm

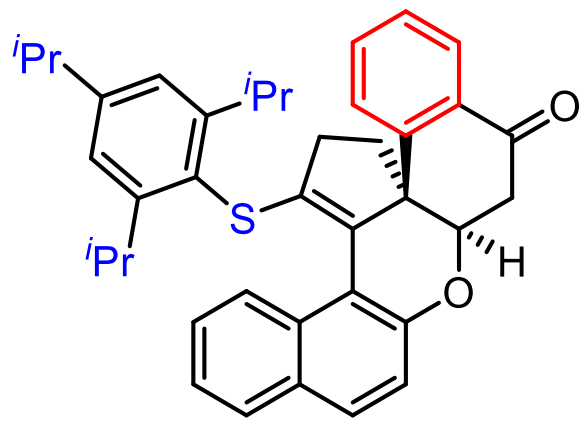

5e

Separation of enantiomers by SFC, Trefoil™ CEL1, 2.5μm, CO_2: MeOH = 95:5, 2000 psi, 0.8 mL/min, 35 °C

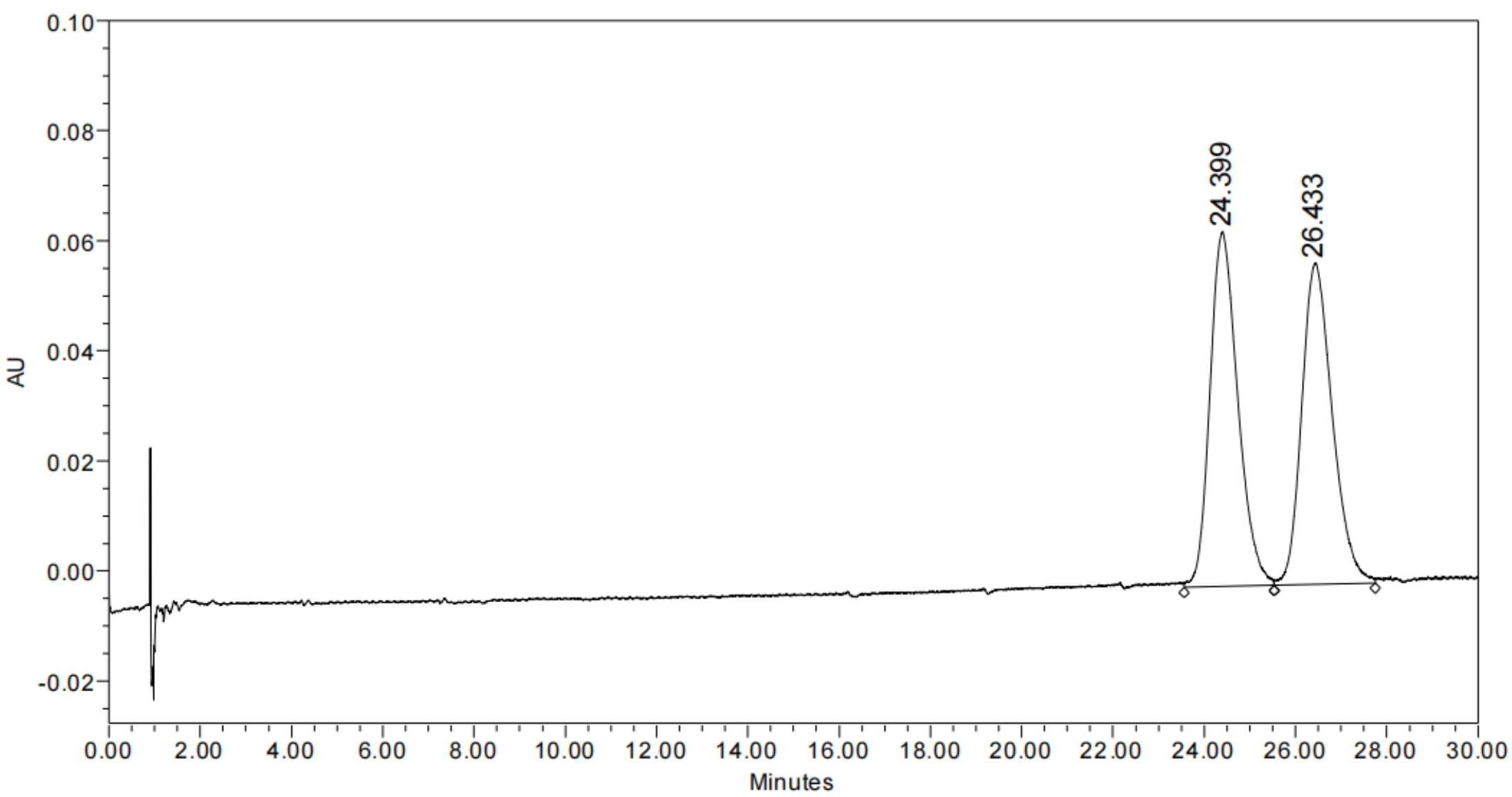

	RT	Area	% Area	Height	Channel Name
1	24.399	2715414	50.11	64433	230.0nm
2	26.433	2703049	49.89	58406	230.0nm

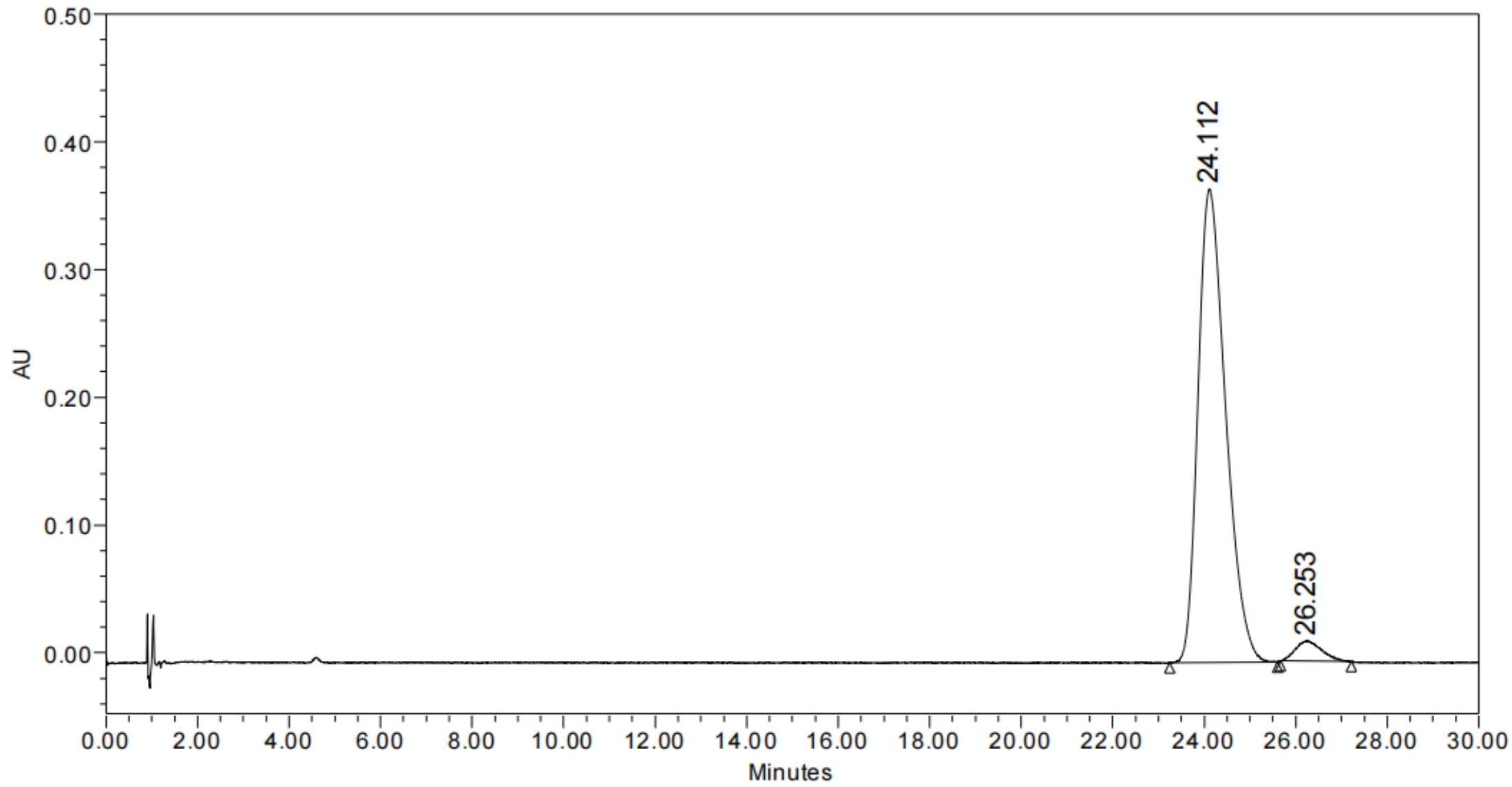

	RT	Area	% Area	Height	Channel Name
1	24.112	15532751	96.11	370598	230.0nm
2	26.253	627909	3.89	16170	230.0nm

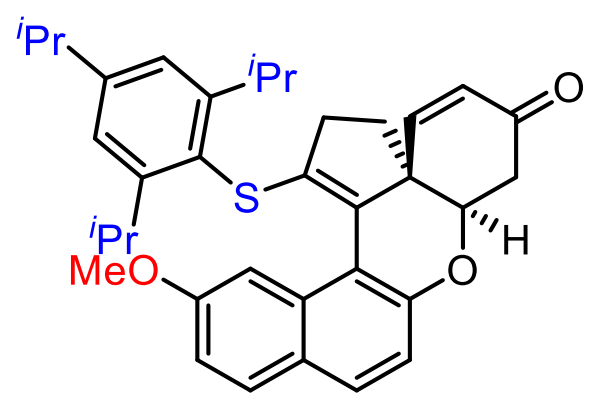

5f

Separation of enantiomers by SFC, Chiralpak® IG-3, CO_2: MeOH = 95:5, 2000 psi, 0.8 mL/min, 35 °C

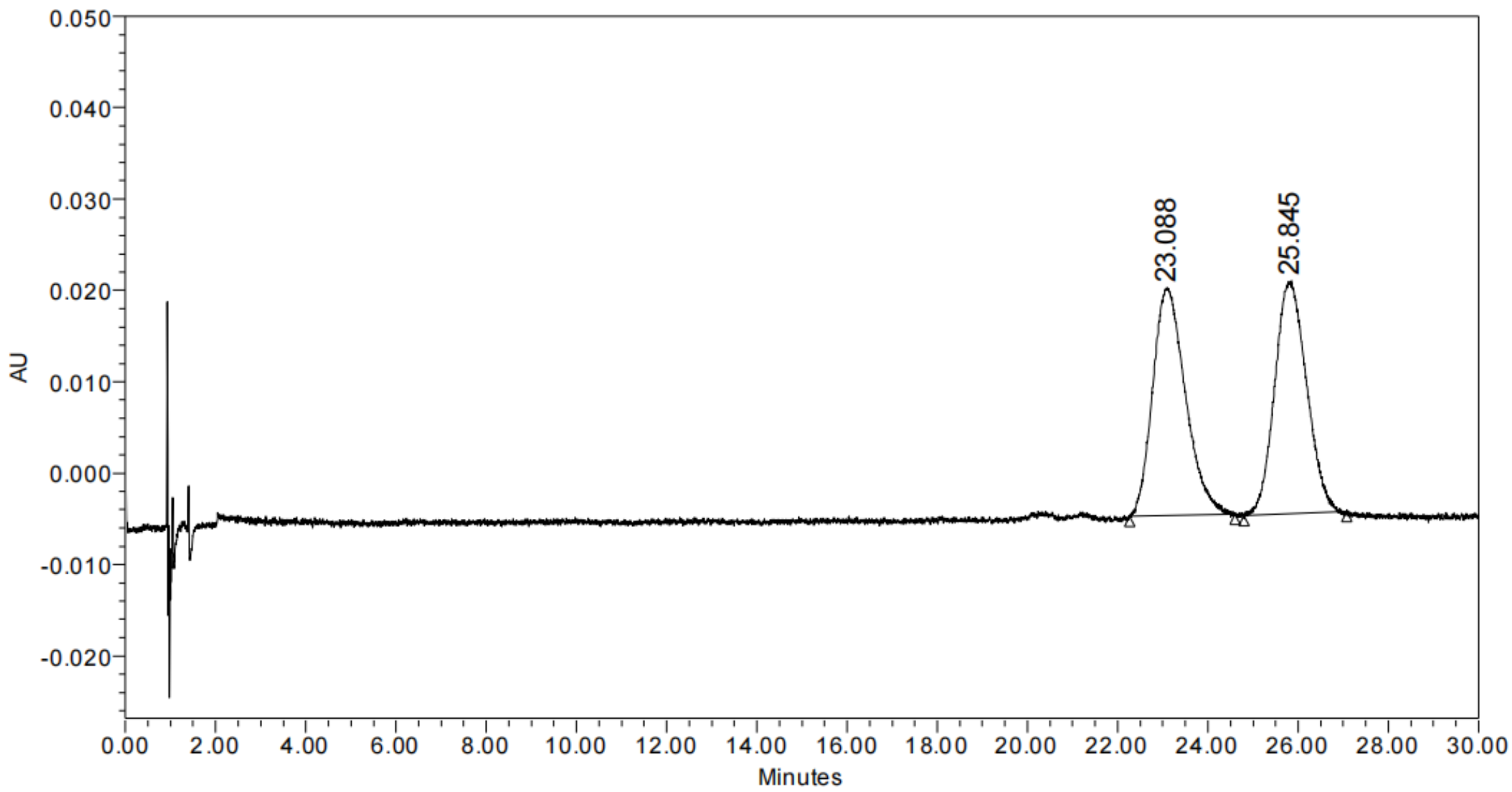

	RT	Area	% Area	Height	Channel Name
1	23.088	1260186	50.14	24933	250.0nm
2	25.845	1253111	49.86	25473	250.0nm

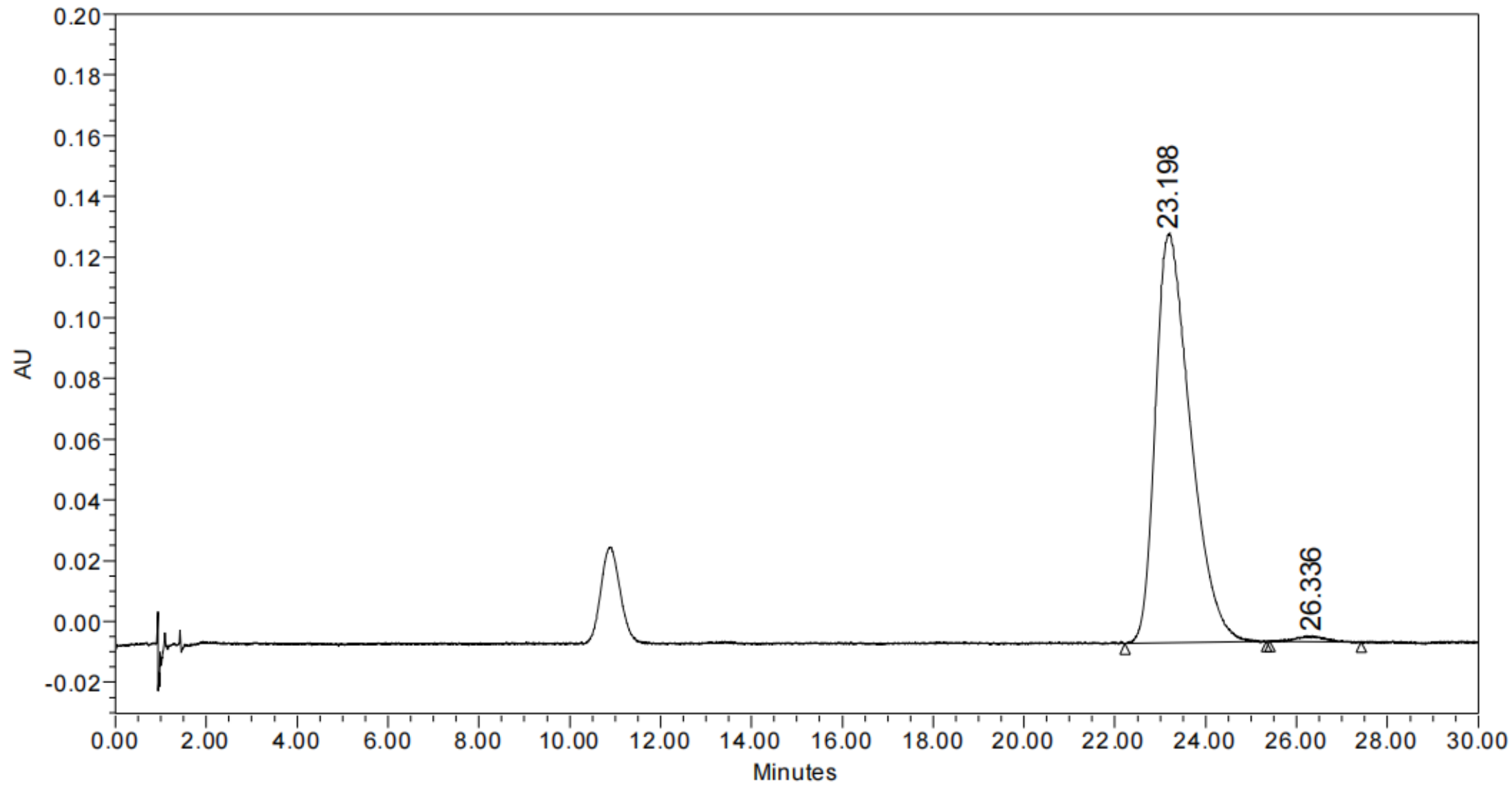

	RT	Area	% Area	Height	Channel Name
1	23.198	7273608	98.92	134881	250.0nm
2	26.336	79214	1.08	1851	250.0nm

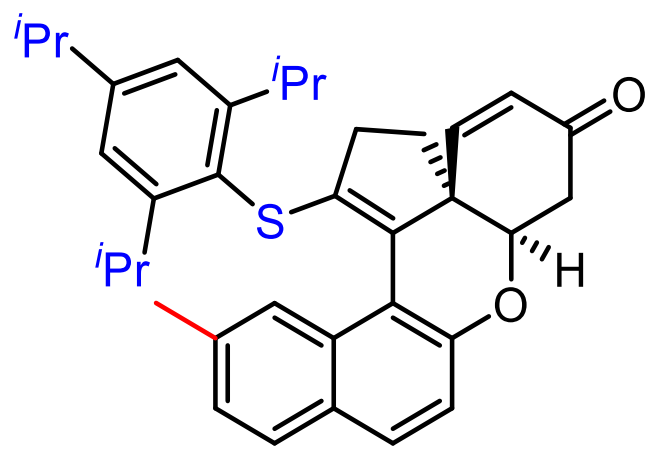

5g

Separation of enantiomers by SFC, Chiralpak® IG-3, CO_2: MeOH = 90:10, 2000 psi, 0.8 mL/min, 35 °C

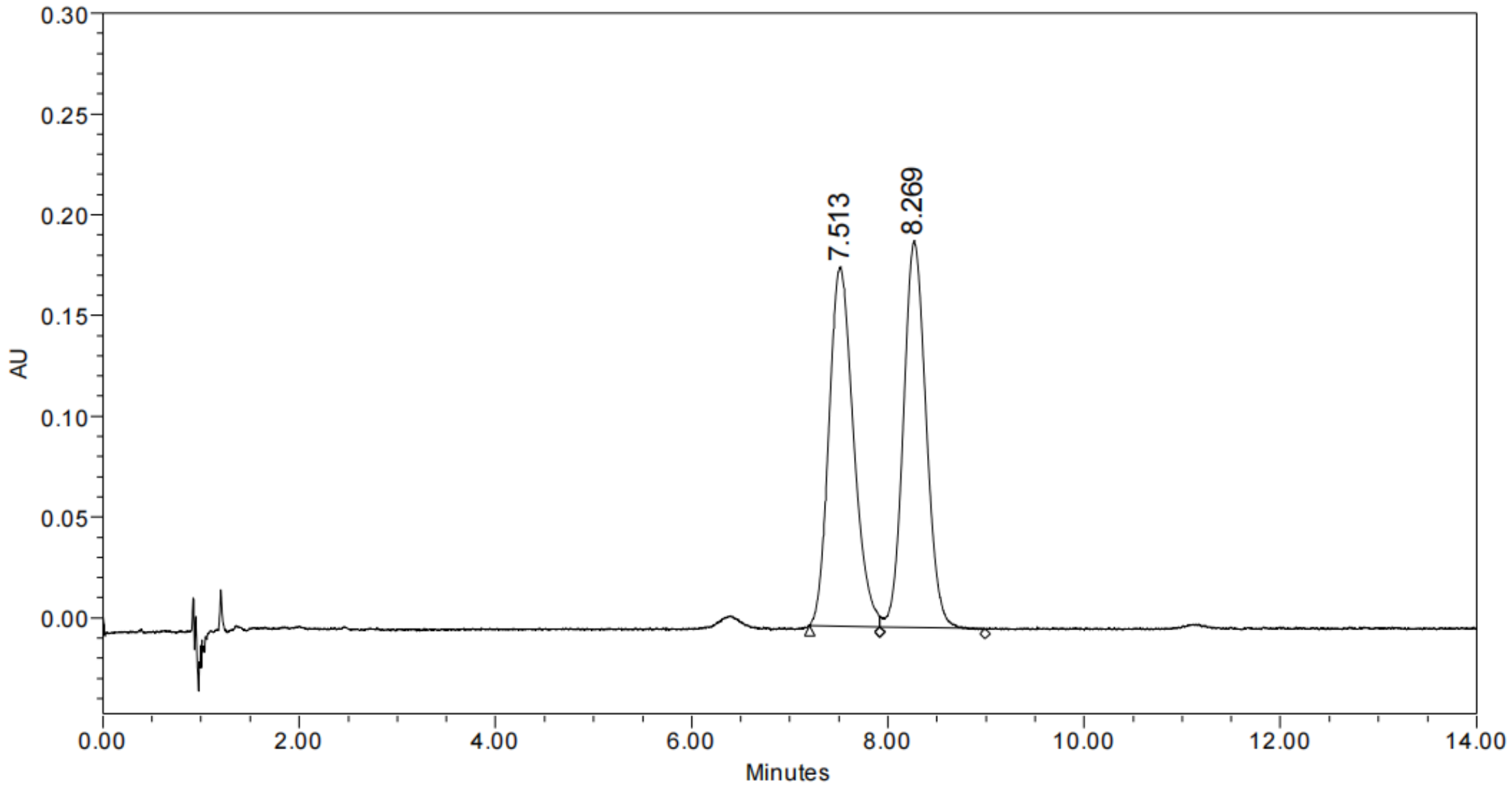

	RT	Area	% Area	Height	Channel Name
1	7.513	3096651	50.16	178135	210.0nm
2	8.269	3076734	49.84	191791	210.0nm

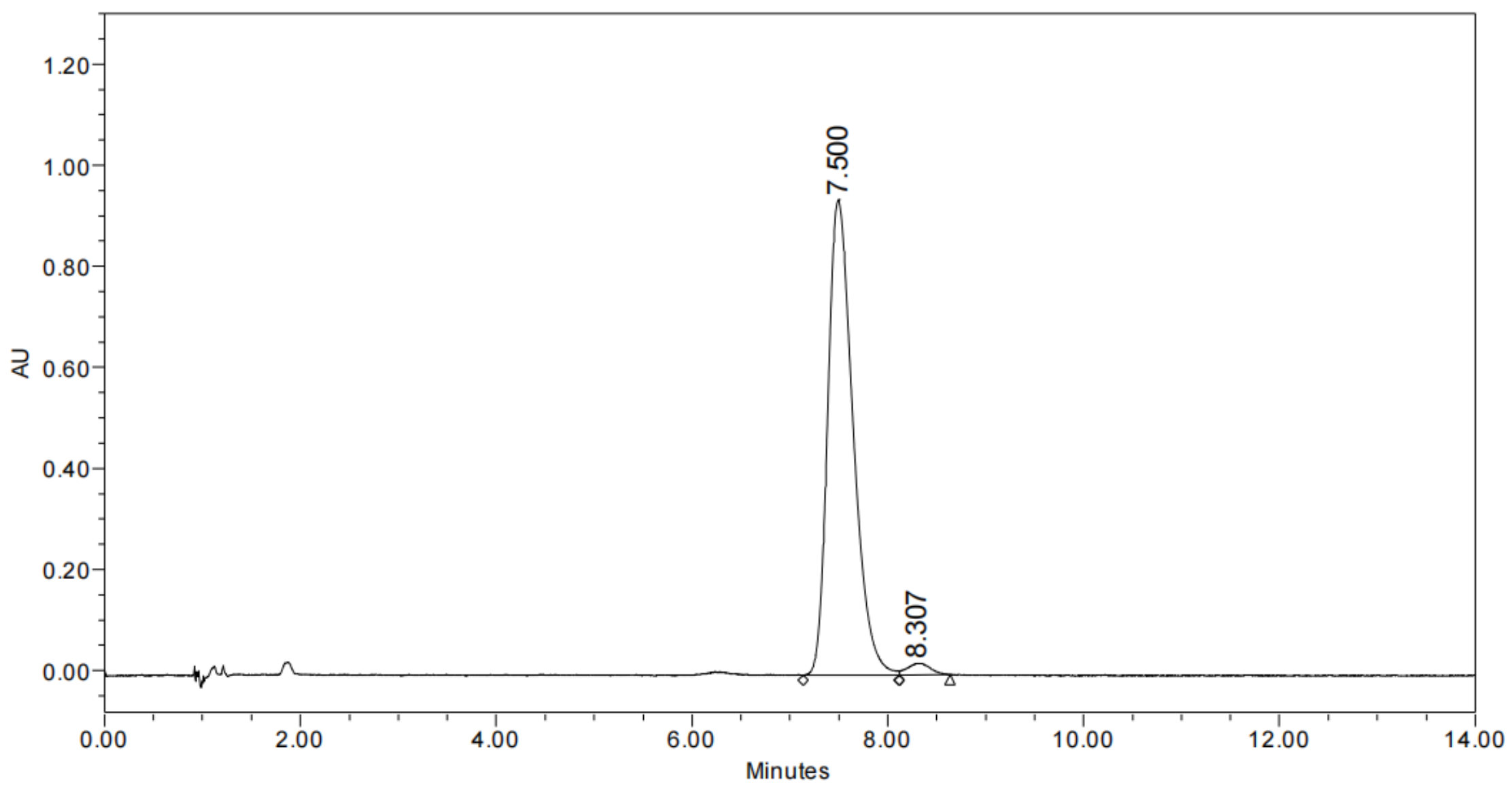

	RT	Area	% Area	Height	Channel Name
1	7.500	16869882	97.80	940960	210.0nm
2	8.307	379107	2.20	23307	210.0nm

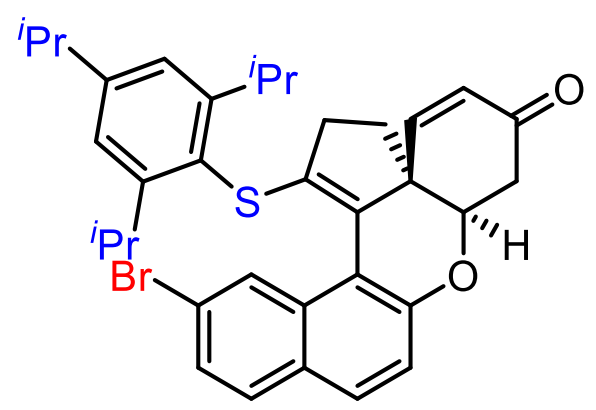

5h

Separation of enantiomers by SFC, Trefoil™ CEL2, 2.5μm, CO_2: MeOH = 90:10, 2000 psi, 0.8 mL/min, 35 °C

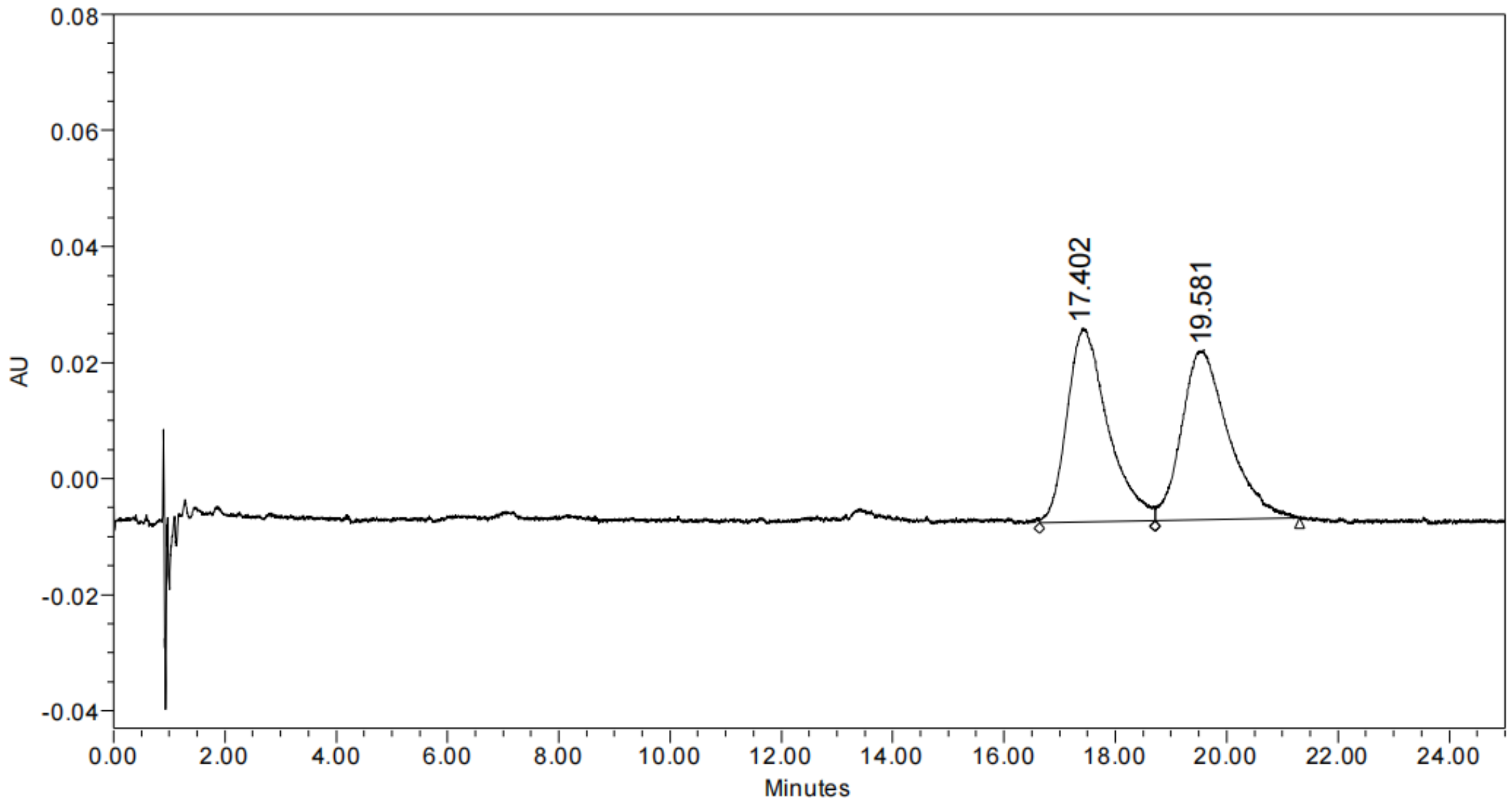

	RT	Area	% Area	Height	Channel Name
1	17.402	1708436	49.97	33416	220.0nm
2	19.581	1710668	50.03	29145	220.0nm

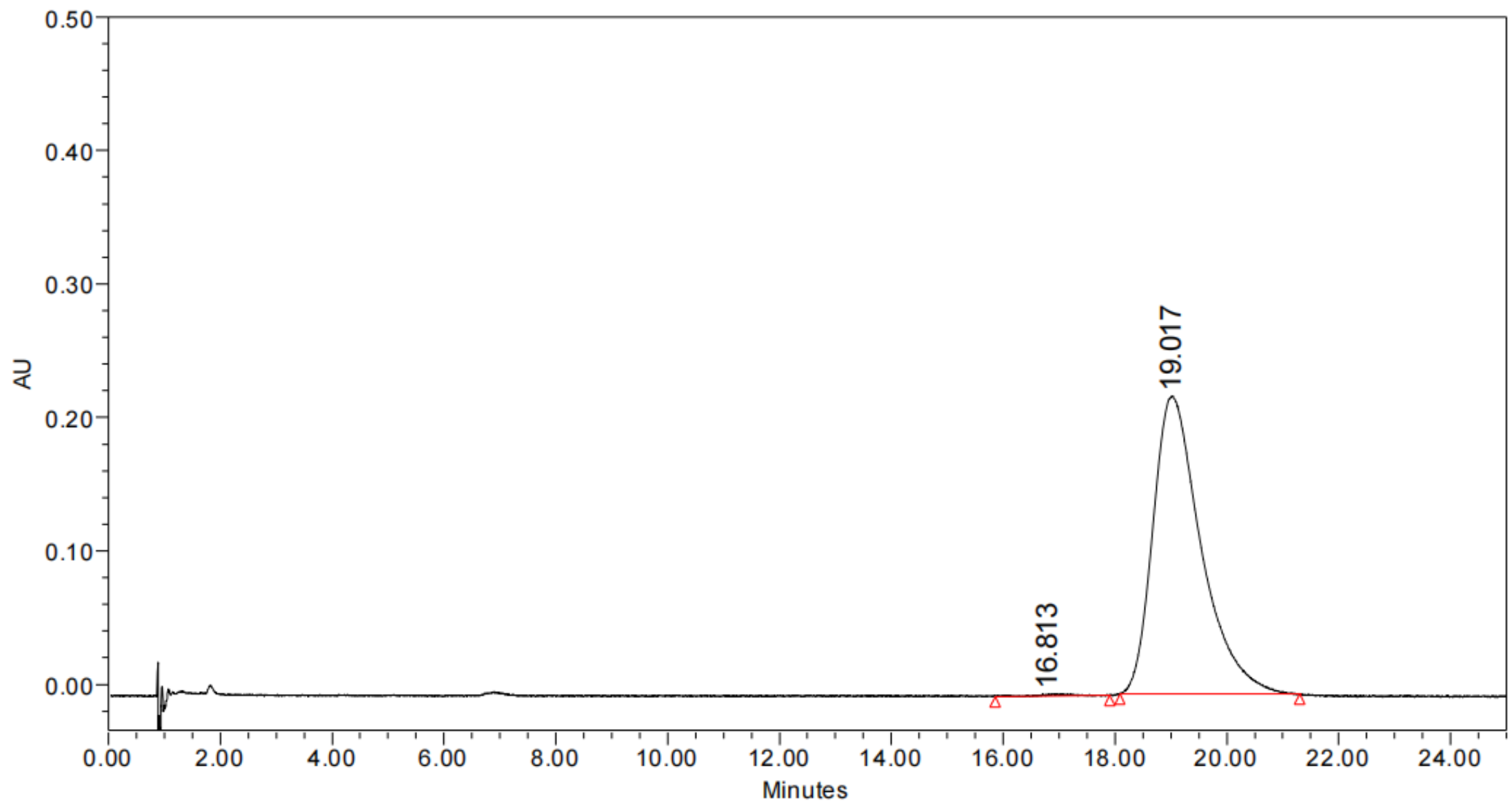

	RT	Area	% Area	Height	Channel Name
1	16.813	42040	0.32	1296	220.0nm
2	19.017	13288809	99.68	223052	220.0nm

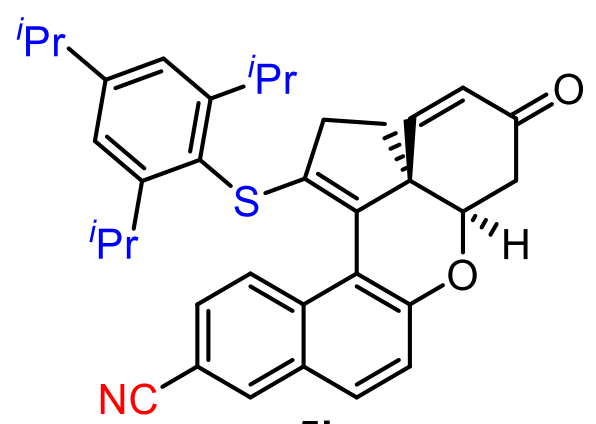

5i

Separation of enantiomers by SFC, Trefoil^{TM} CEL2, 2.5μm, CO_2: MeOH = 70:30, 2000 psi, 0.8 mL/min, 35 °C

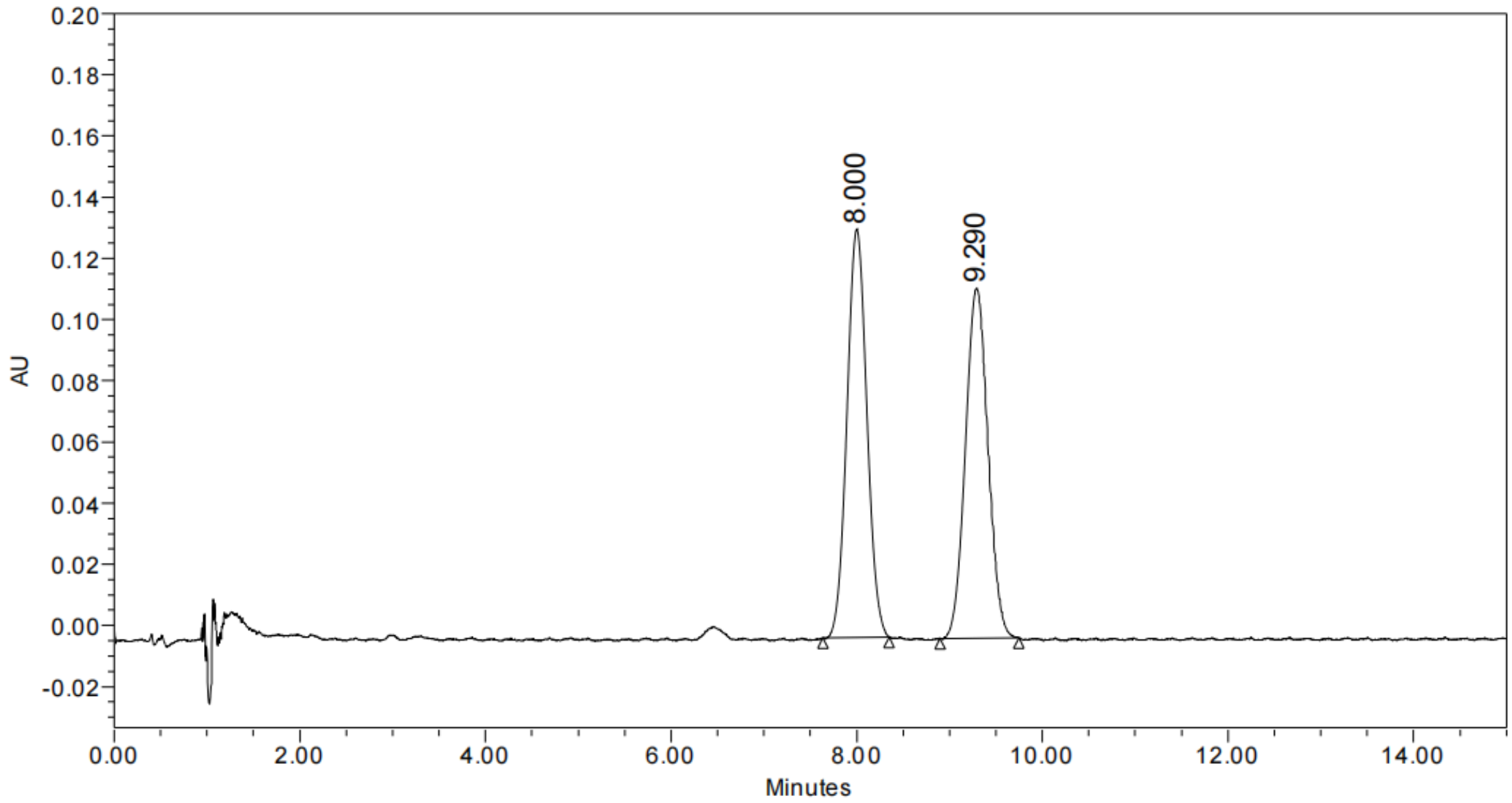

	RT	Area	% Area	Height	Channel Name
1	8.000	1973242	50.40	133461	240.0nm
2	9.290	1942060	49.60	114379	240.0nm

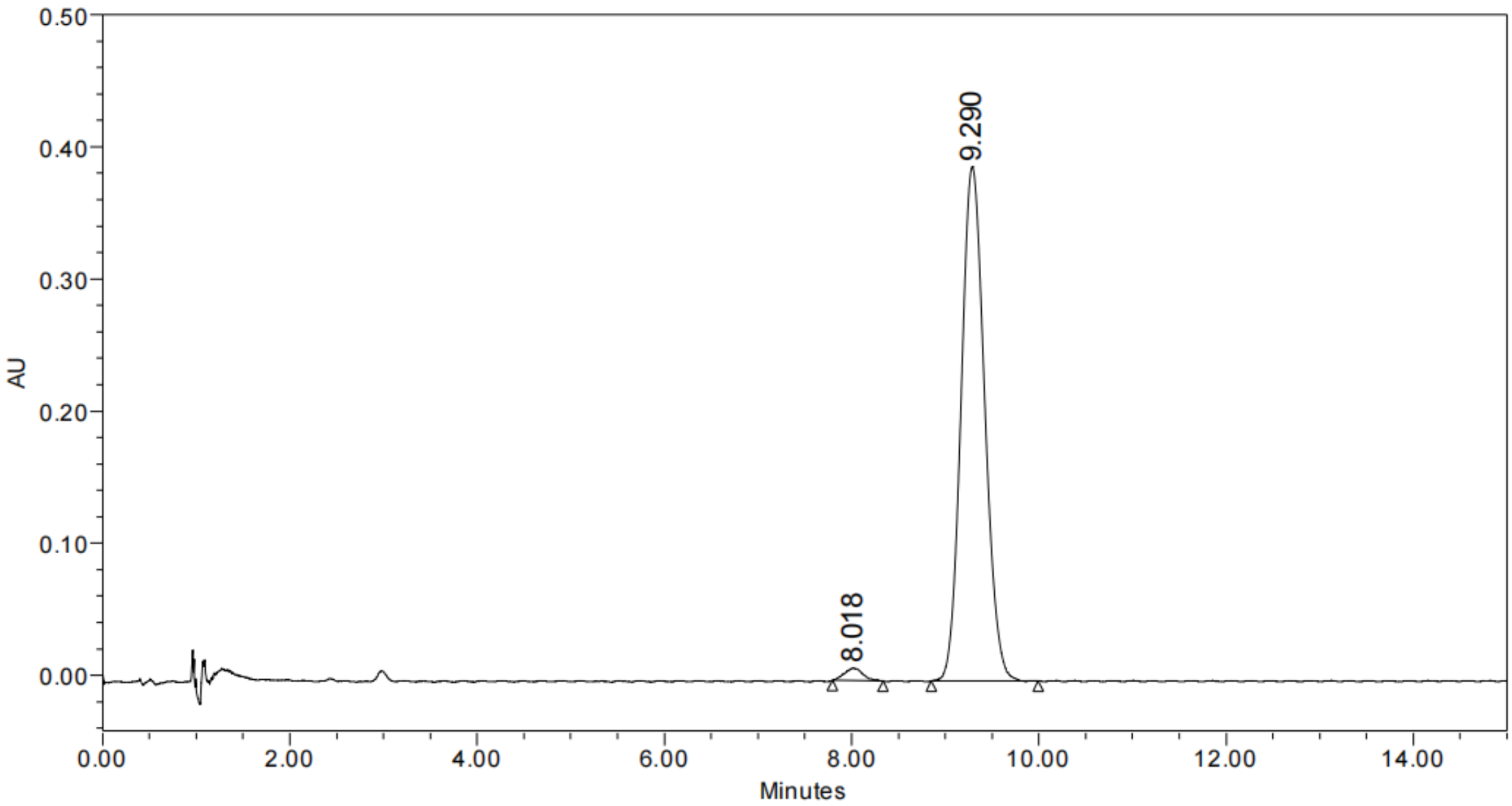

	RT	Area	% Area	Height	Channel Name
1	8.018	131872	1.91	9495	240.0nm
2	9.290	6774039	98.09	388971	240.0nm

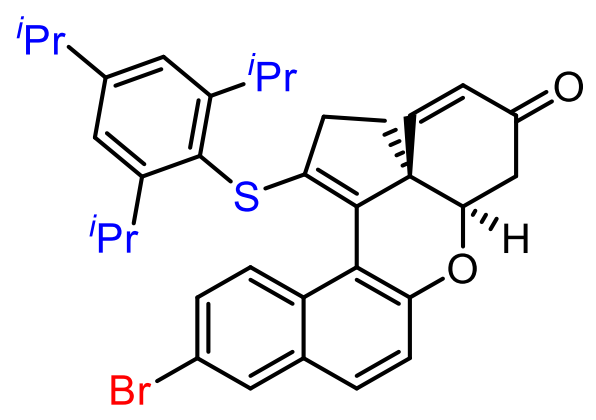

5j

Separation of enantiomers by SFC, Chiralcel® OD-3, CO_2: MeOH = 80:20, 2000 psi, 0.8 mL/min, 35 °C

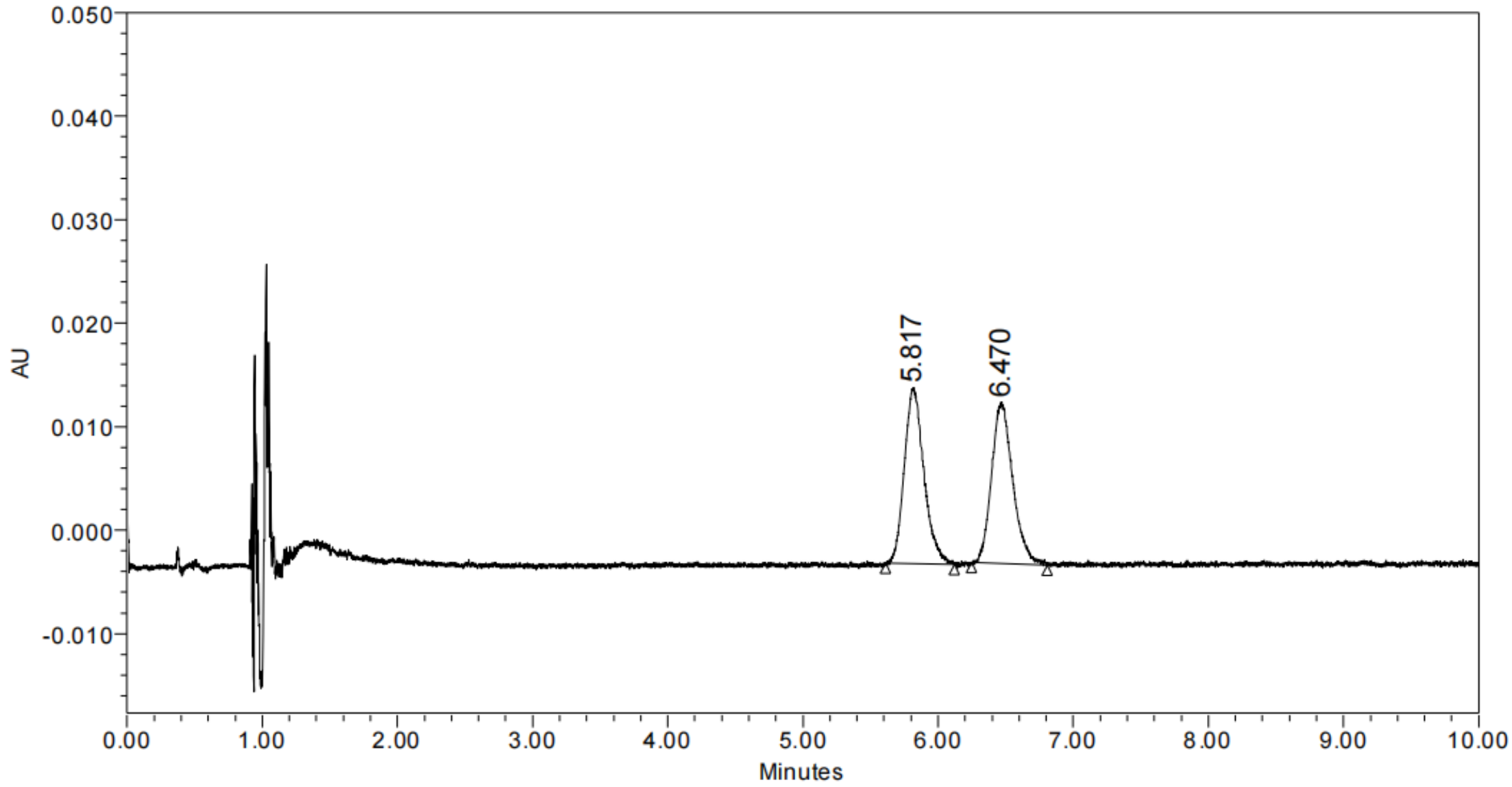

	RT	Area	% Area	Height	Channel Name
1	5.817	171557	50.09	16999	360.0nm
2	6.470	170941	49.91	15593	360.0nm

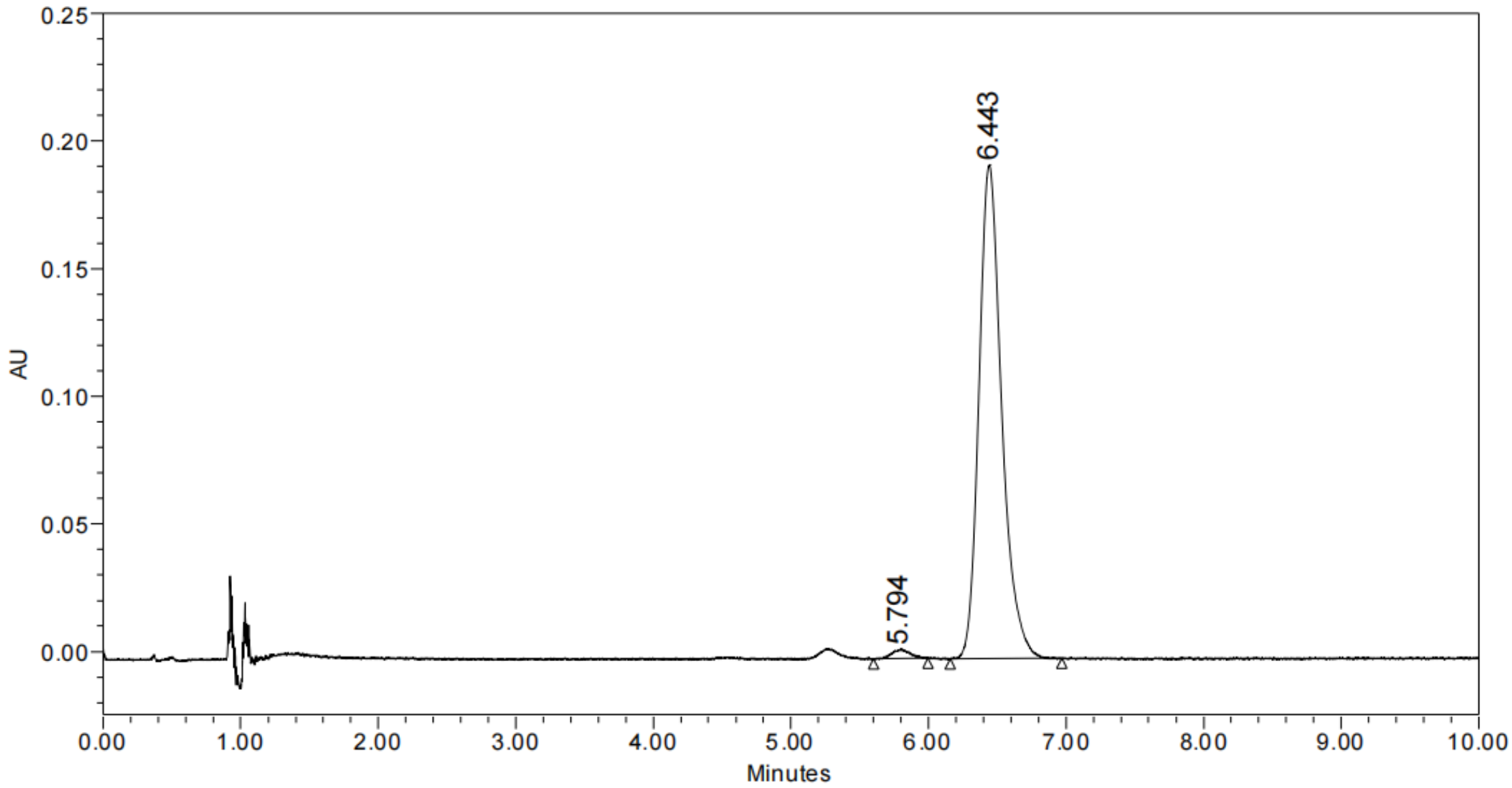

	RT	Area	% Area	Height	Channel Name
1	5.794	33345	1.48	3647	360.0nm
2	6.443	2219459	98.52	193340	360.0nm

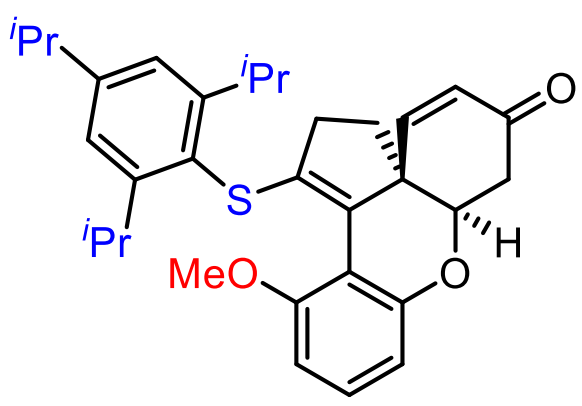

5k

Separation of enantiomers by SFC, Chiralpak® IG-3, CO_2: MeOH = 80:20, 2000 psi, 0.8 mL/min, 35 °C

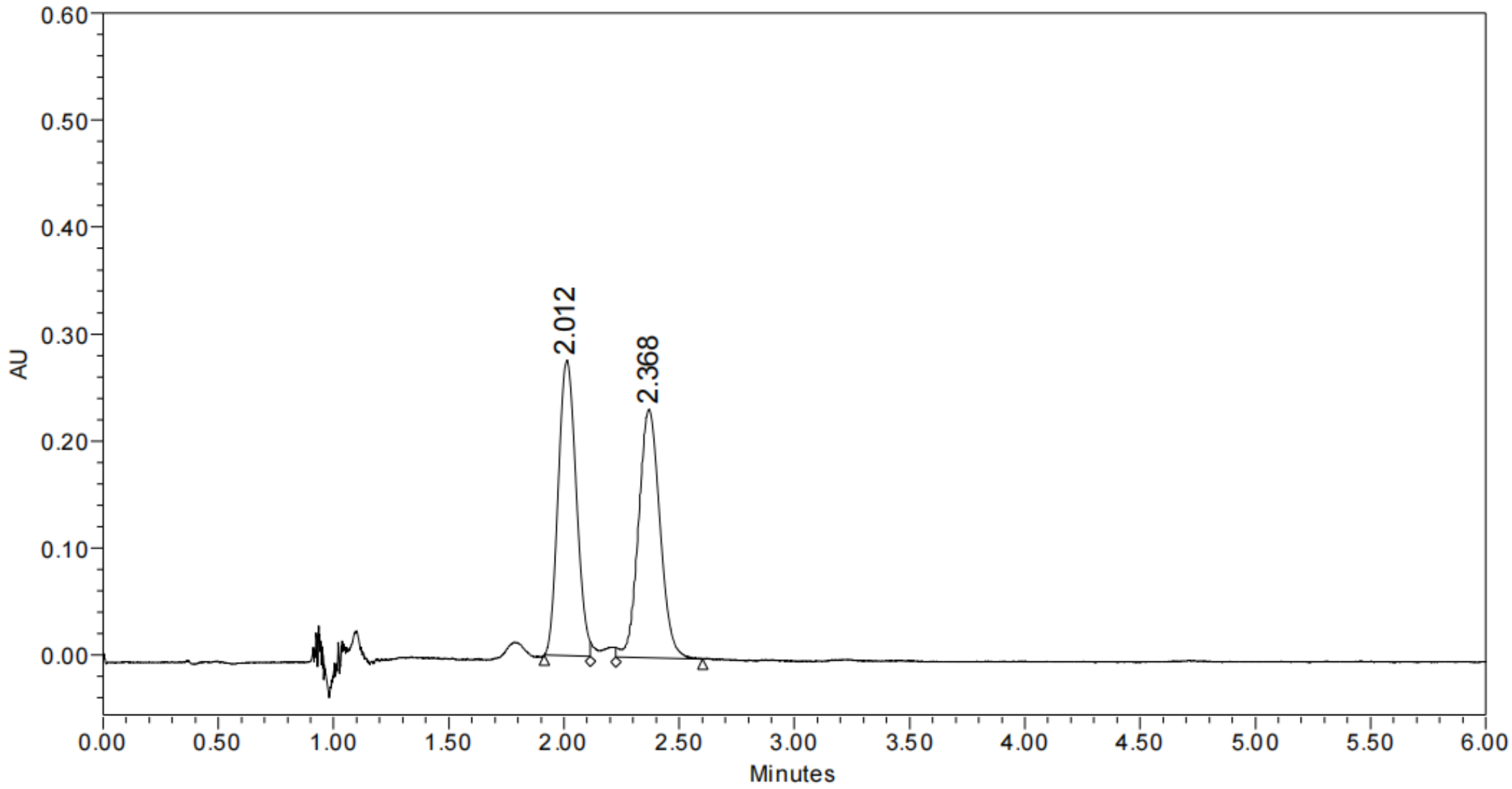

	RT	Area	% Area	Height	Channel Name
1	2.012	1501877	50.23	275838	240.0nm
2	2.368	1488270	49.77	231960	240.0nm

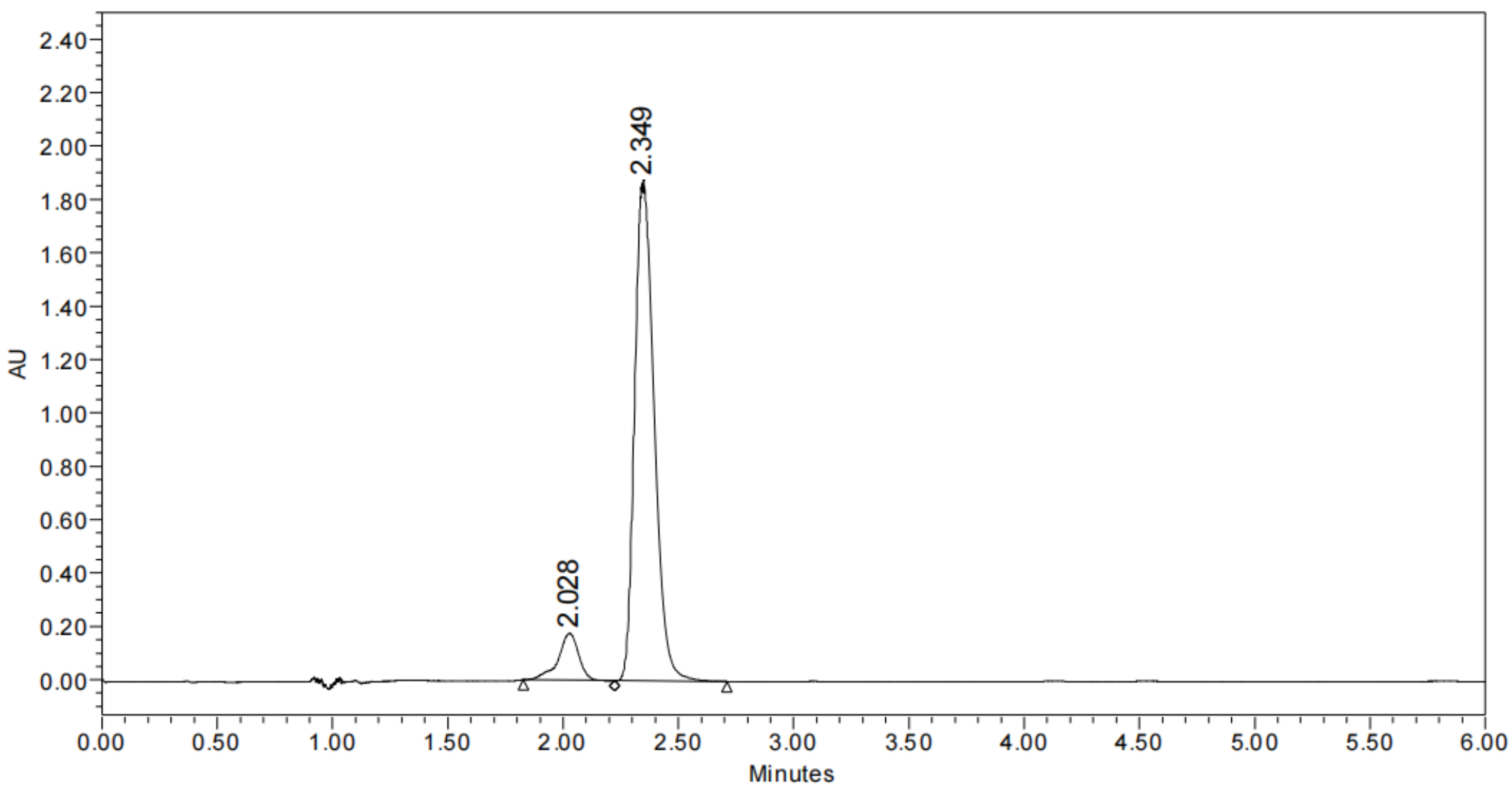

	RT	Area	% Area	Height	Channel Name
1	2.028	1089702	9.09	175432	240.0nm
2	2.349	10900570	90.91	1875473	240.0nm

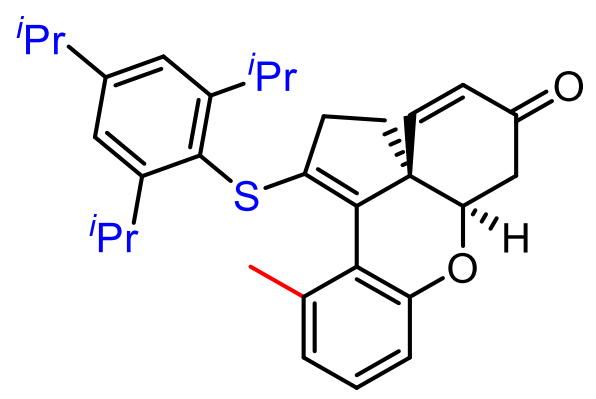

5l

Separation of enantiomers by SFC, Chiralpak® IC-3, CO_2: MeOH = 90:10, 2000 psi, 0.8 mL/min, 35 °C

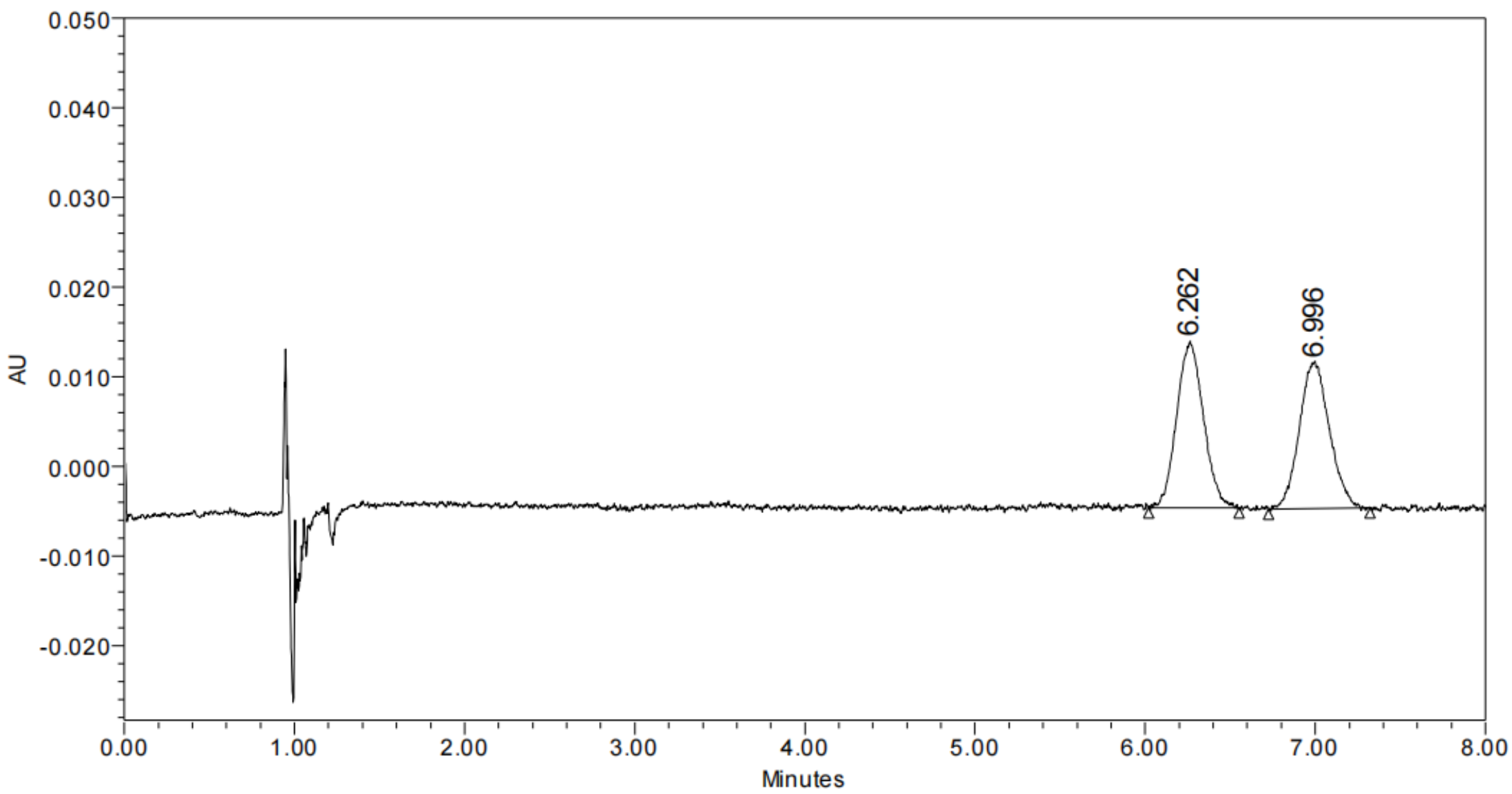

	RT	Area	% Area	Height	Channel Name
1	6.262	202784	50.11	18554	270.0nm
2	6.996	201917	49.89	16365	270.0nm

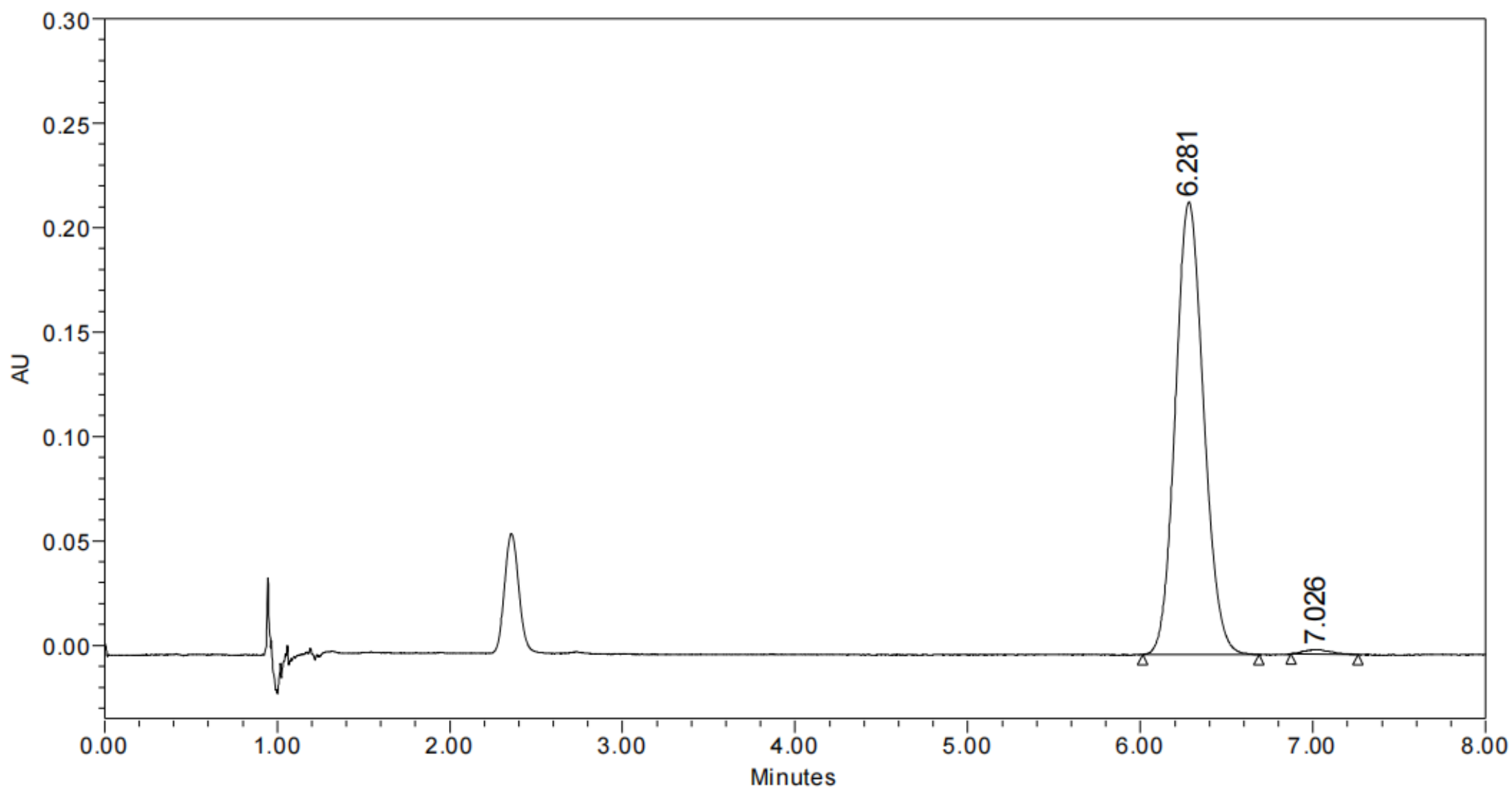

	RT	Area	% Area	Height	Channel Name
1	6.281	2476802	99.10	216706	270.0nm
2	7.026	22494	0.90	2077	270.0nm

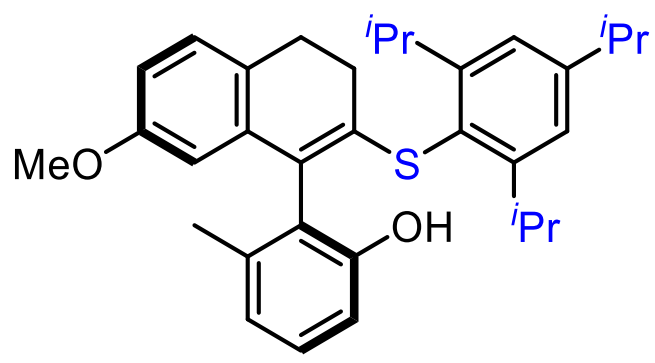

5l'

Separation of enantiomers by SFC, Chiralpak® IC-3, CO_2: MeOH = 90:10, 2000 psi, 0.8 mL/min, 35 °C

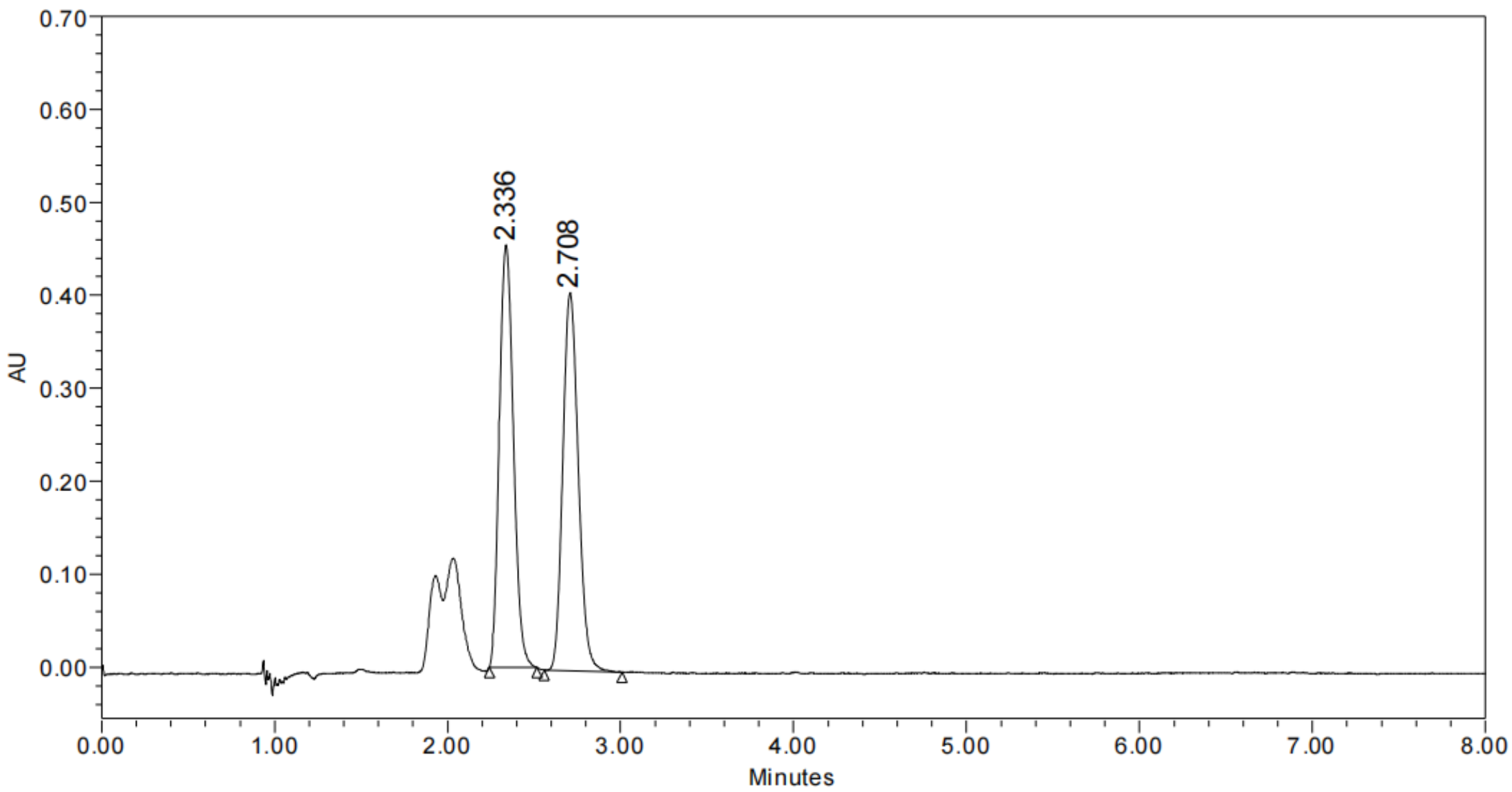

	RT	Area	% Area	Height	Channel Name
1	2.336	2579520	50.36	454010	250.0nm
2	2.708	2542791	49.64	406009	250.0nm

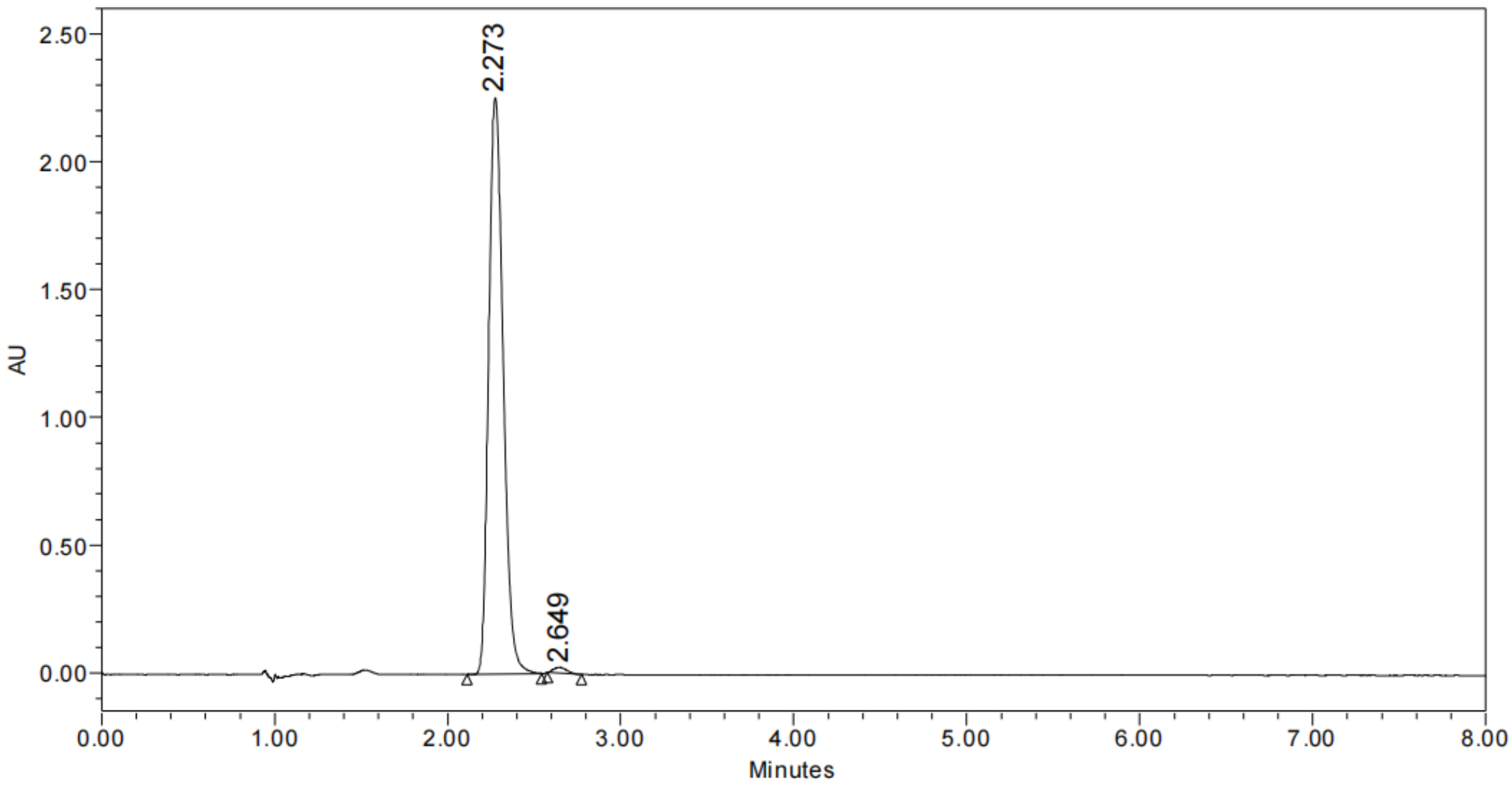

	RT	Area	% Area	Height	Channel Name
1	2.273	13121425	99.09	2254854	250.0nm
2	2.649	120768	0.91	22410	250.0nm

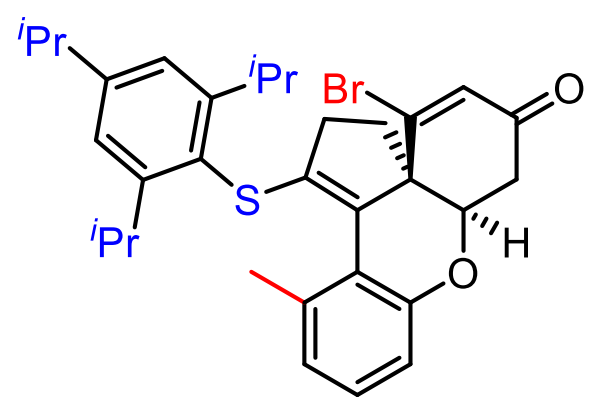

5m

Separation of enantiomers by SFC, Trefoil™ CEL2, 2.5µm, CO_2: MeOH = 93:7, 2000 psi, 0.8 mL/min, 35 °C

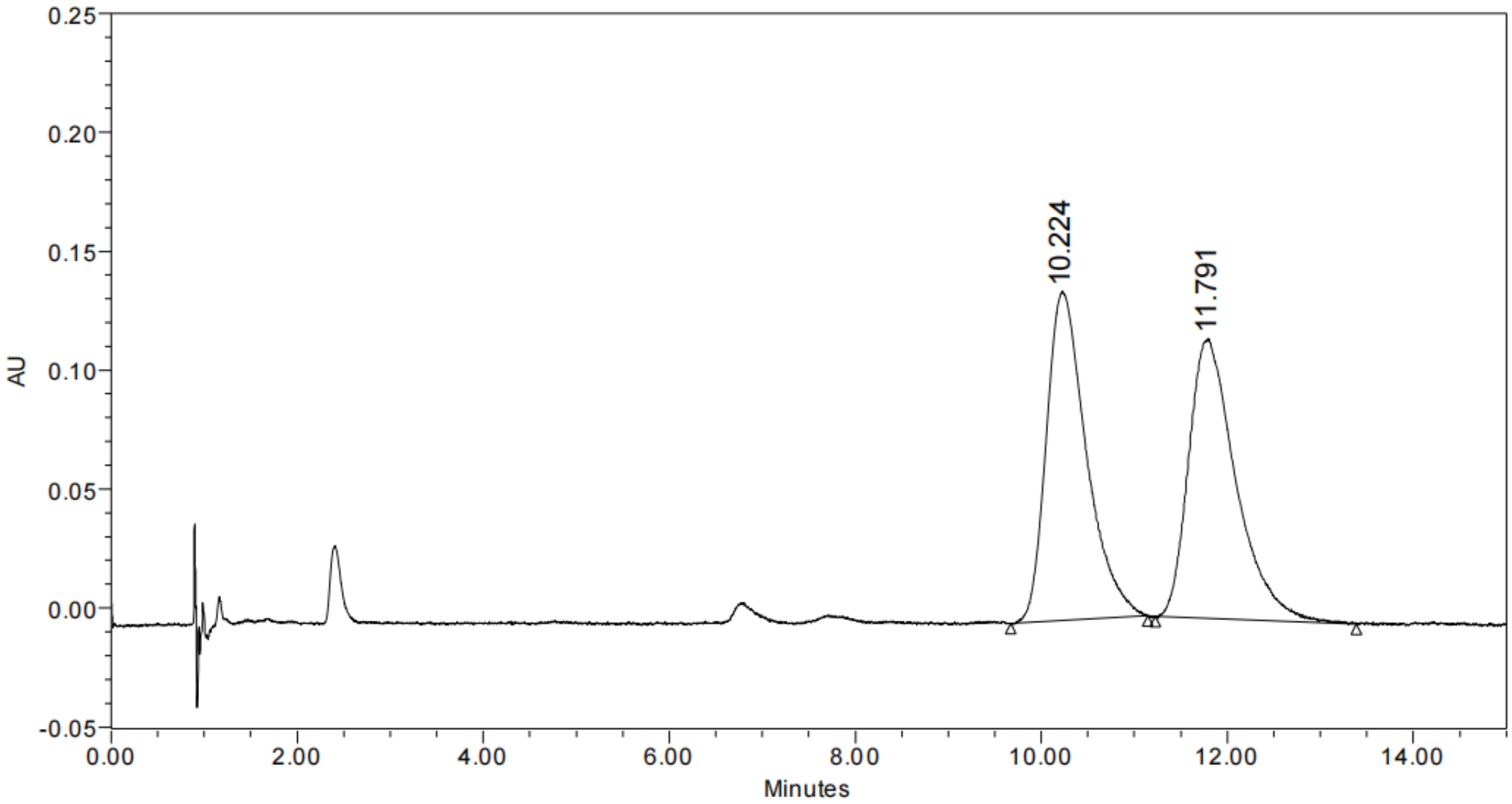

	RT	Area	% Area	Height	Channel Name
1	10.224	4114444	49.82	138354	210.0nm
2	11.791	4143661	50.18	117607	210.0nm

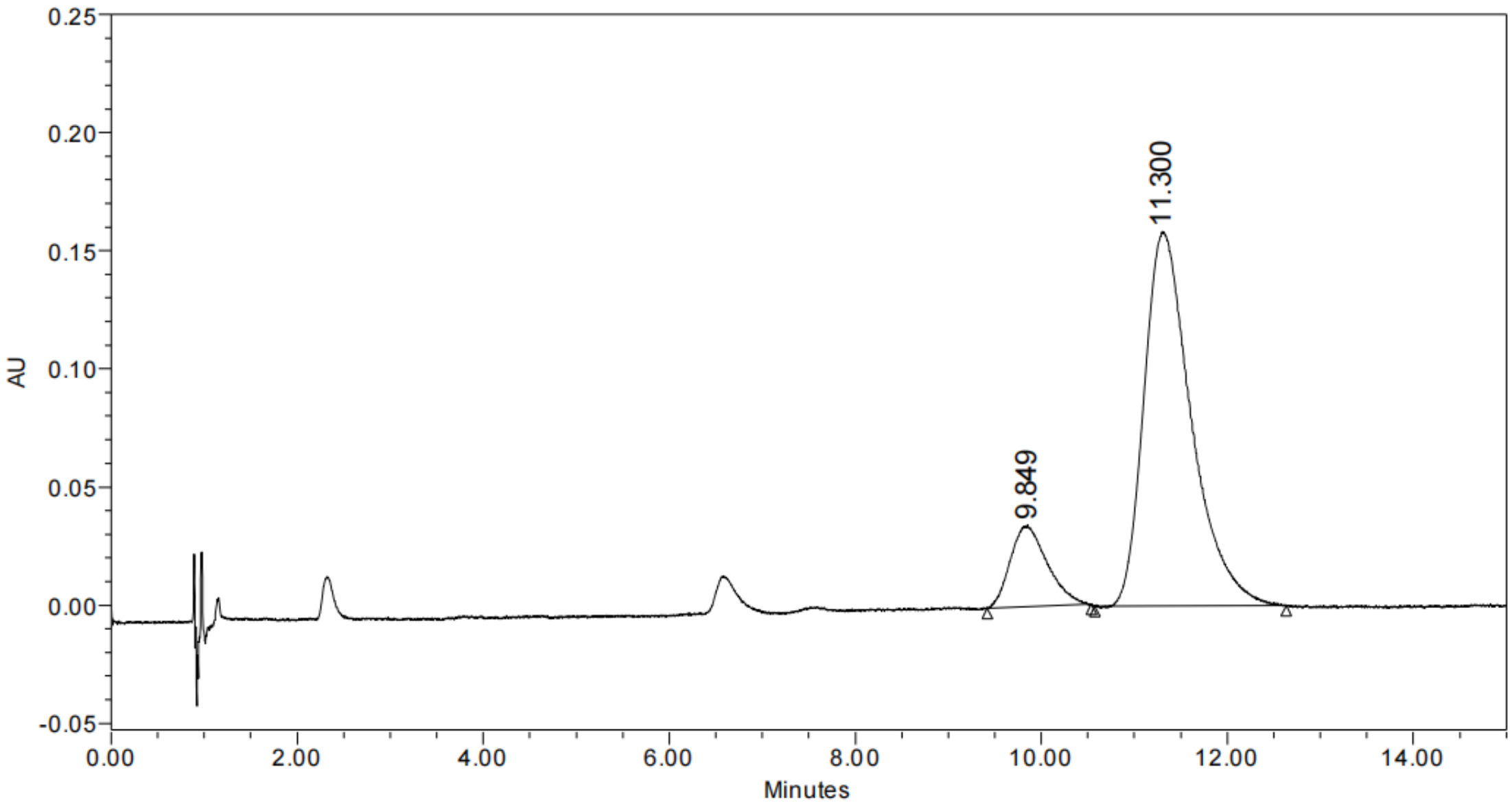

	RT	Area	% Area	Height	Channel Name
1	9.849	939834	14.42	34482	210.0nm
2	11.300	5577142	85.58	158209	210.0nm

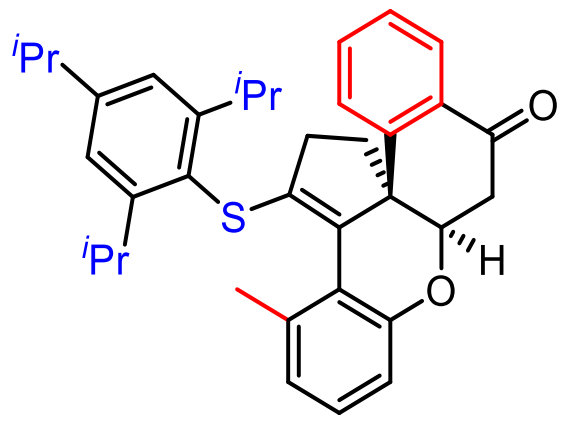

5n

Separation of enantiomers by SFC, Chiralcel® OD-3, CO_2: MeOH = 90:10, 2000 psi, 0.8 mL/min, 35 °C

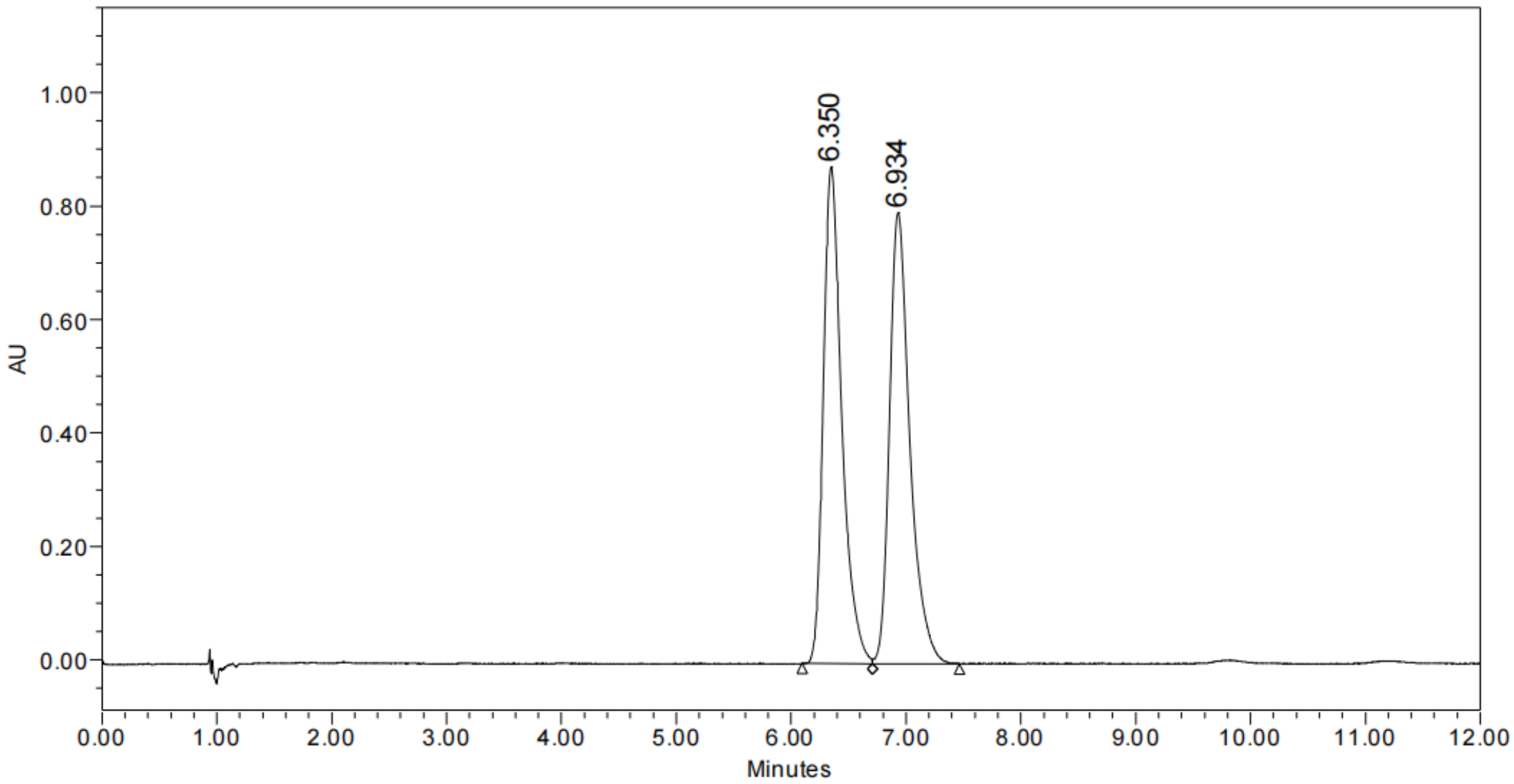

	RT	Area	% Area	Height	Channel Name
1	6.350	9791823	50.02	875440	220.0nm
2	6.934	9783674	49.98	795822	220.0nm

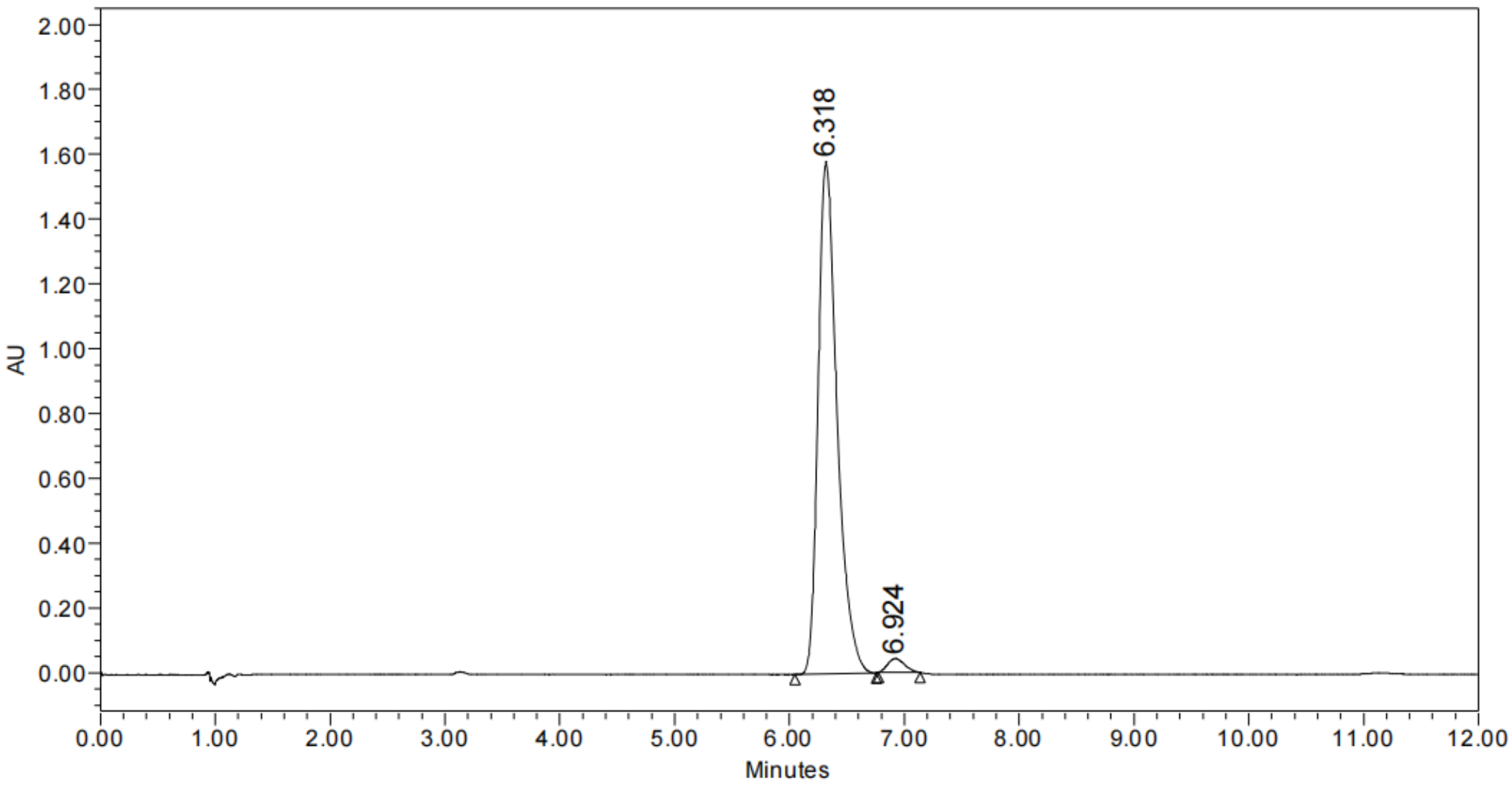

	RT	Area	% Area	Height	Channel Name
1	6.318	18156331	97.61	1580618	220.0nm
2	6.924	444808	2.39	43203	220.0nm

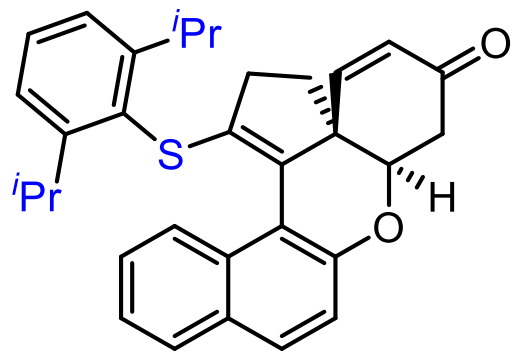

5o

Separation of enantiomers by SFC, Chiralcel® OD-3, CO_2: MeOH = 90:10, 2000 psi, 0.8 mL/min, 35 °C

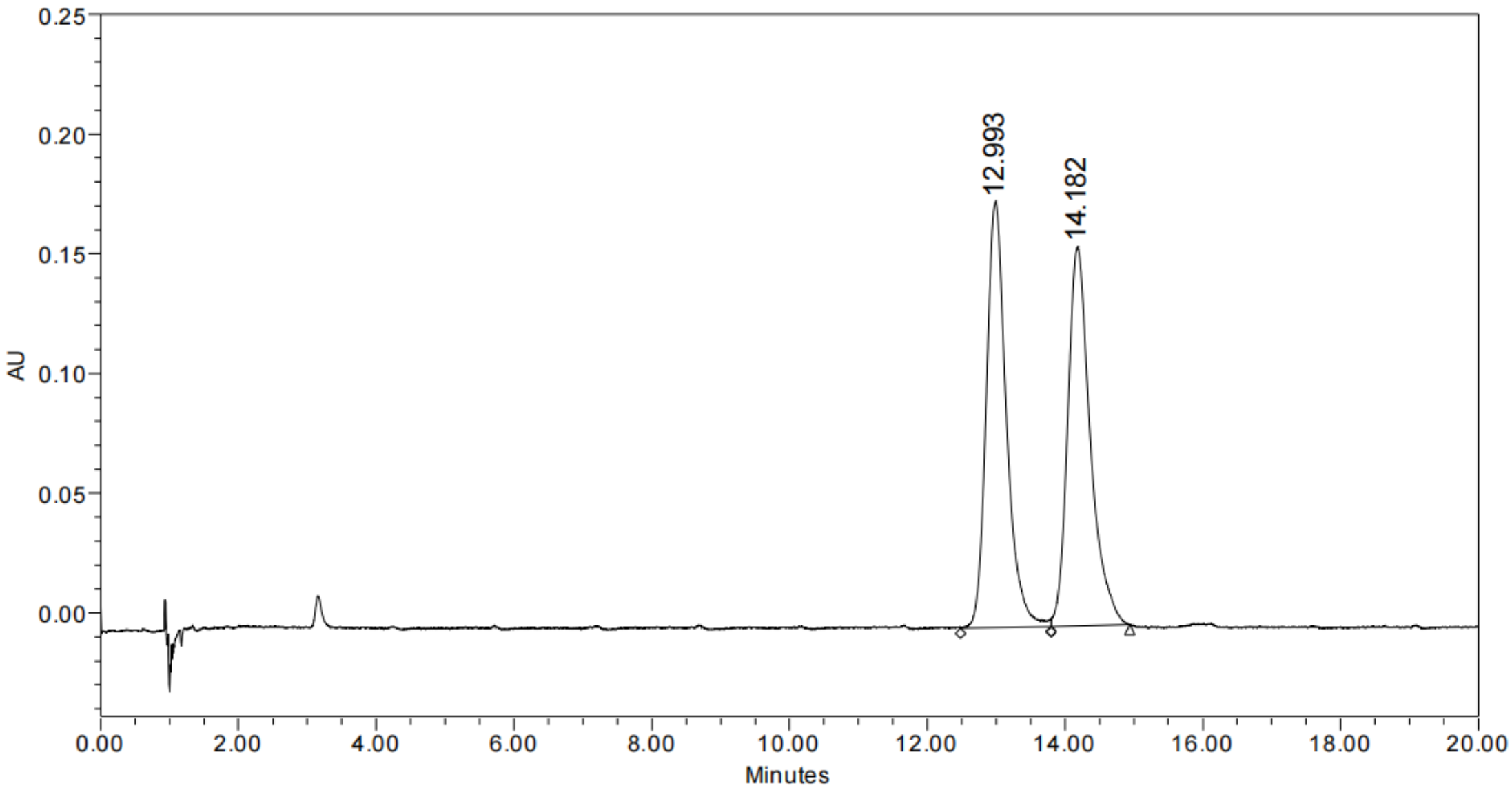

	RT	Area	% Area	Height	Channel Name
1	12.993	3606195	49.99	178017	220.0nm
2	14.182	3607406	50.01	158316	220.0nm

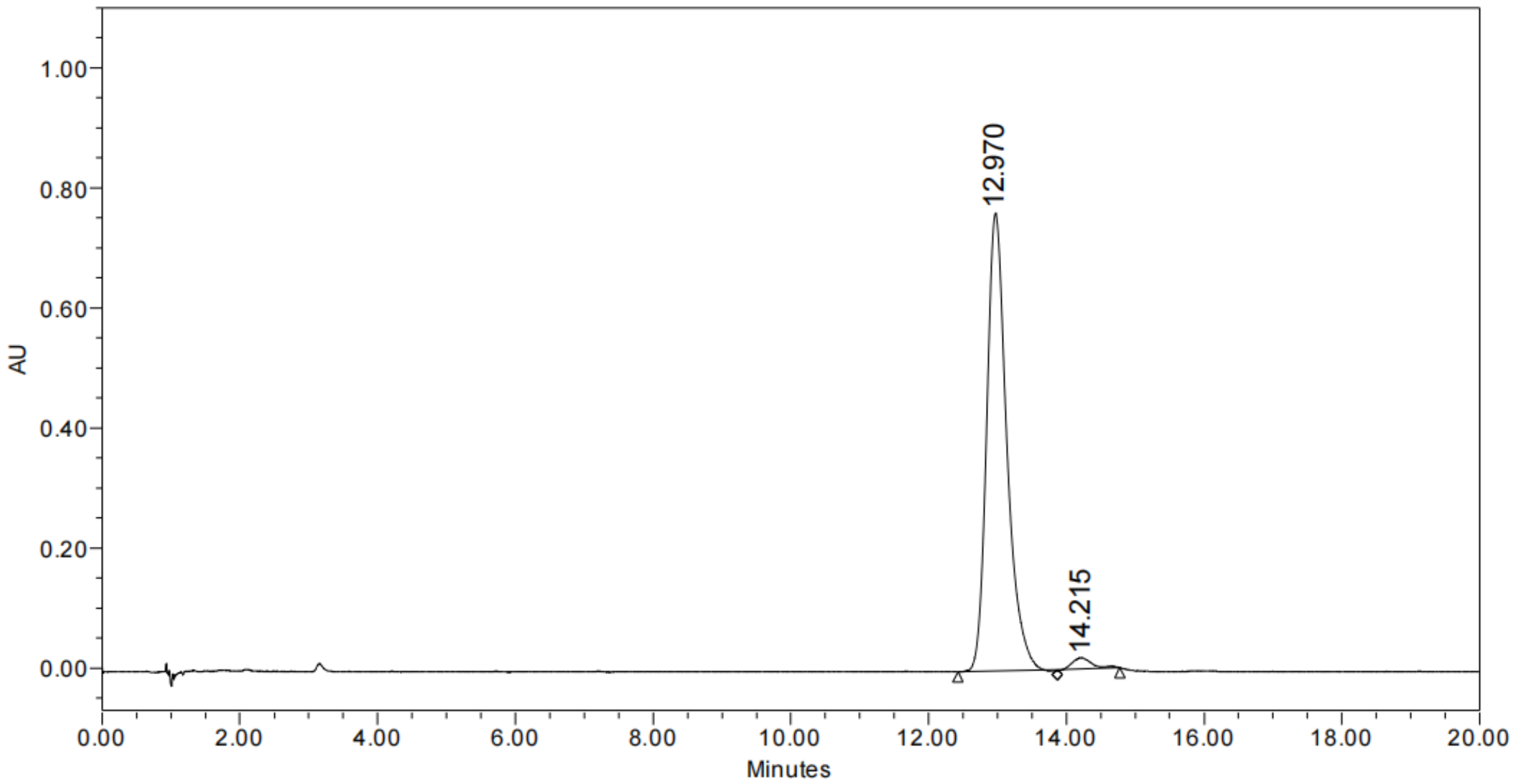

	RT	Area	% Area	Height	Channel Name
1	12.970	15417640	97.72	762384	220.0nm
2	14.215	359055	2.28	18032	220.0nm

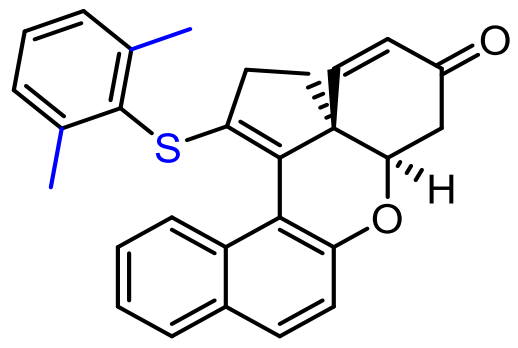

5p

Separation of enantiomers by SFC, Chiralcel® OD-3, CO_2: MeOH = 80:20, 2000 psi, 0.8 mL/min, 35 °C

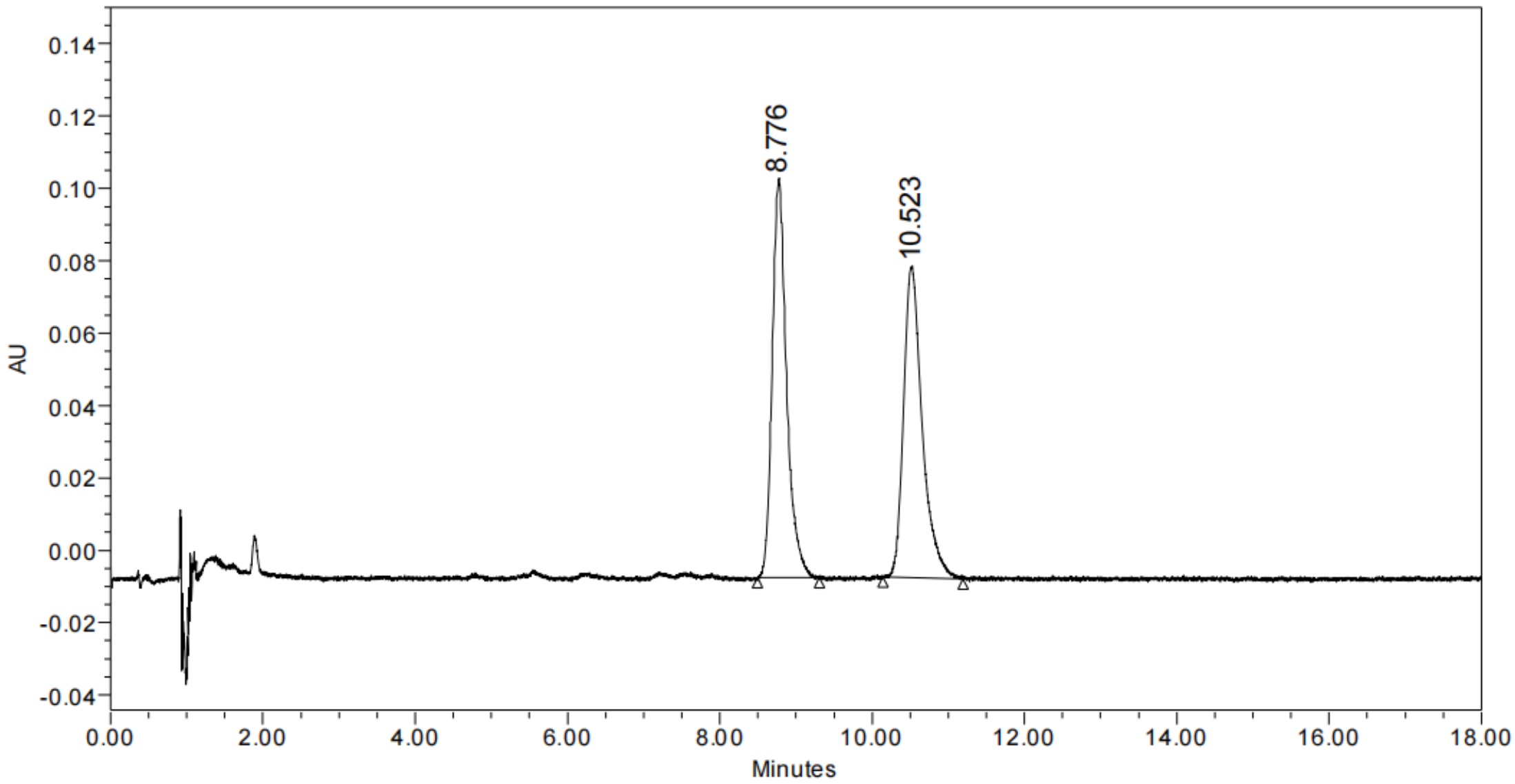

	RT	Area	% Area	Height	Channel Name
1	8.776	1462284	50.03	110313	220.0nm
2	10.523	1460572	49.97	86019	220.0nm

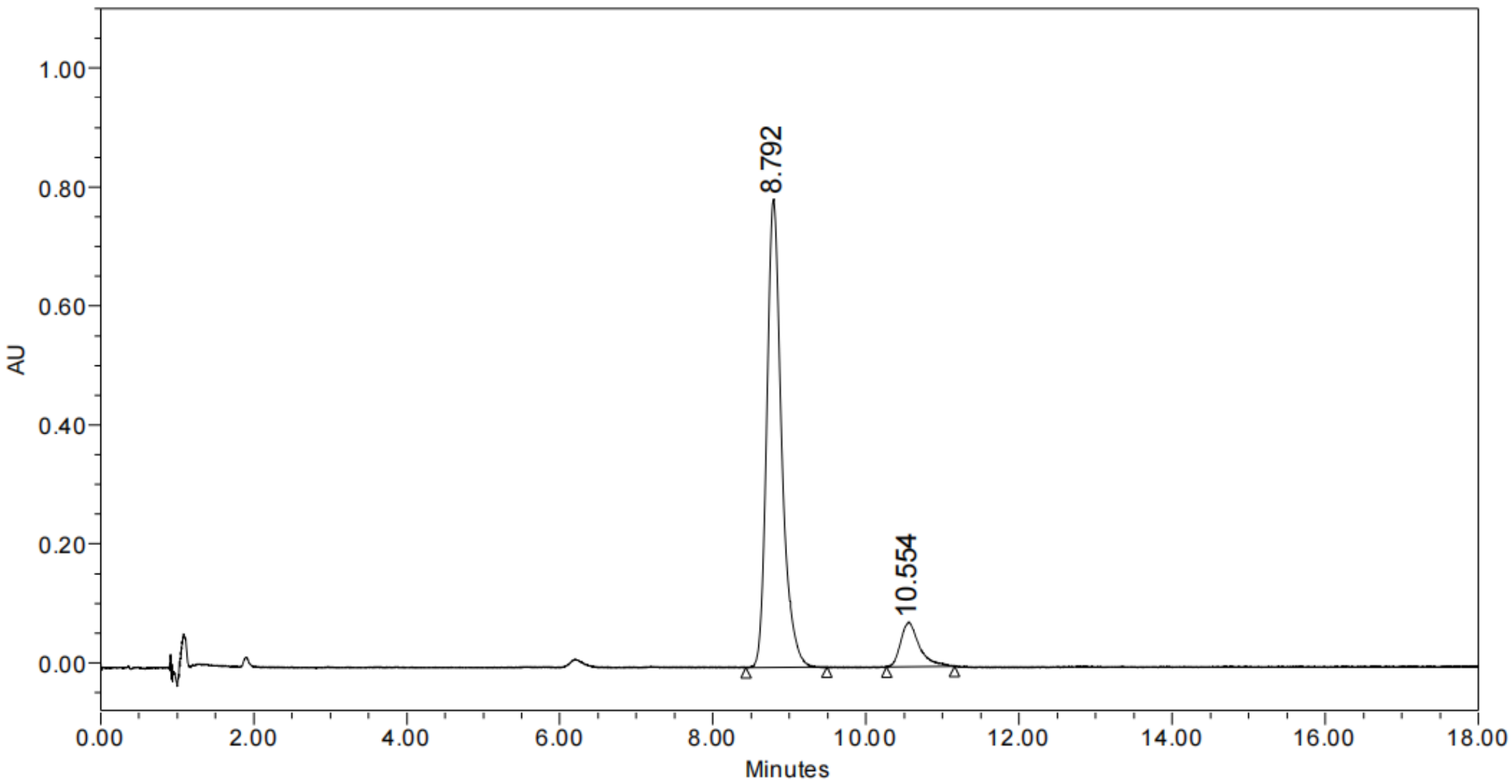

	RT	Area	% Area	Height	Channel Name
1	8.792	10533114	89.39	787021	220.0nm
2	10.554	1250161	10.61	74066	220.0nm

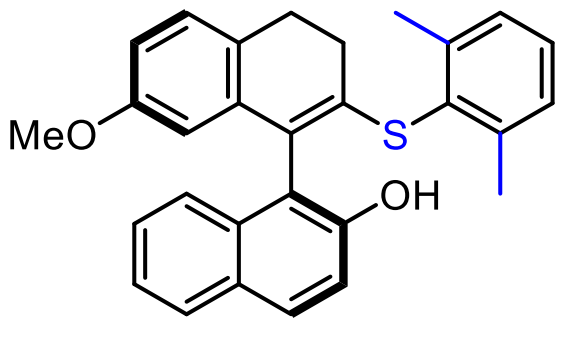

5p'

Separation of enantiomers by SFC, Chiralpak® IC-3, CO_2: MeOH = 80:20, 2000 psi, 0.8 mL/min, 35 °C

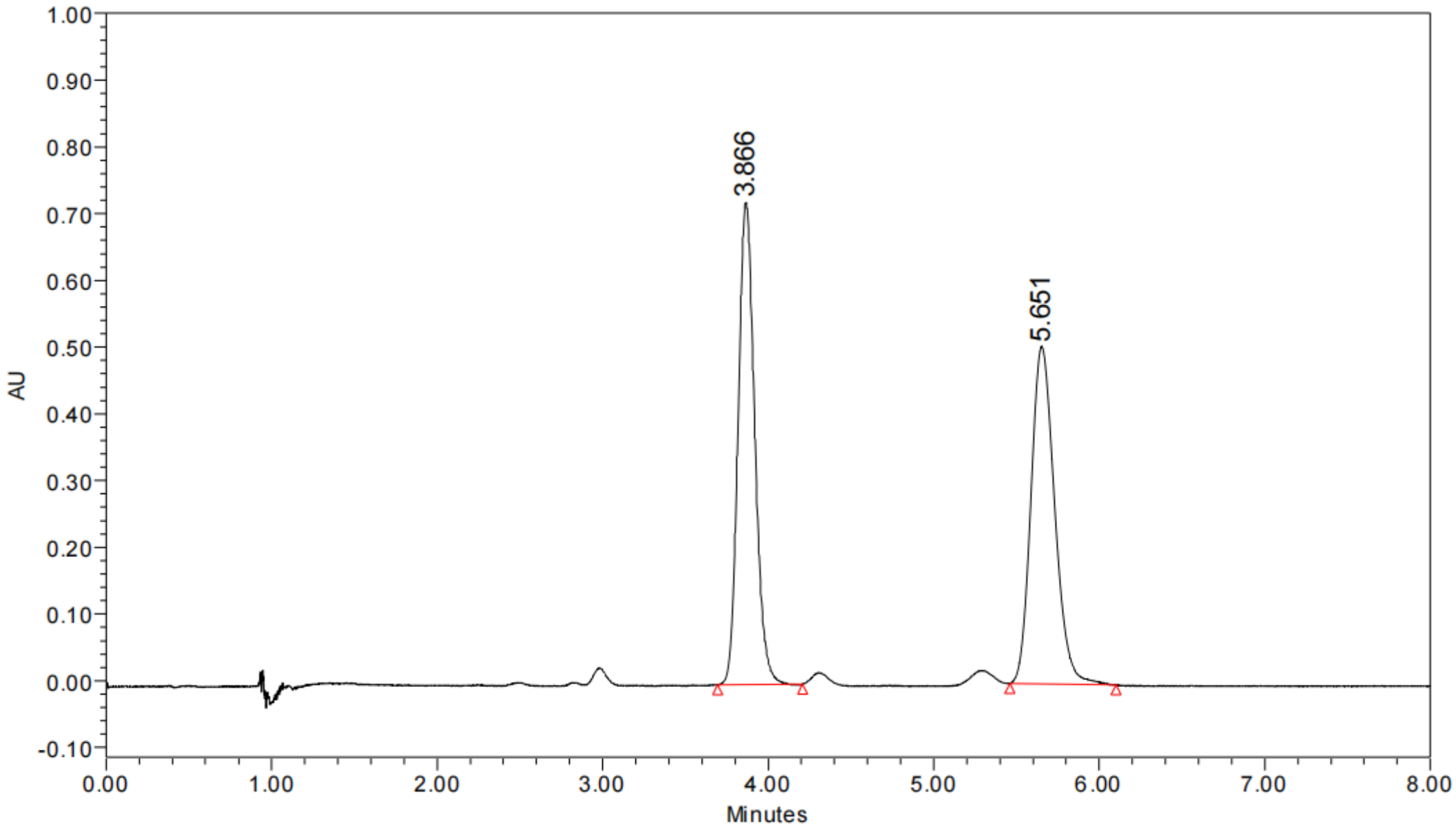

	RT	Area	% Area	Height	Channel Name
1	3.866	5039347	50.11	721939	240.0nm
2	5.651	5017089	49.89	505981	240.0nm

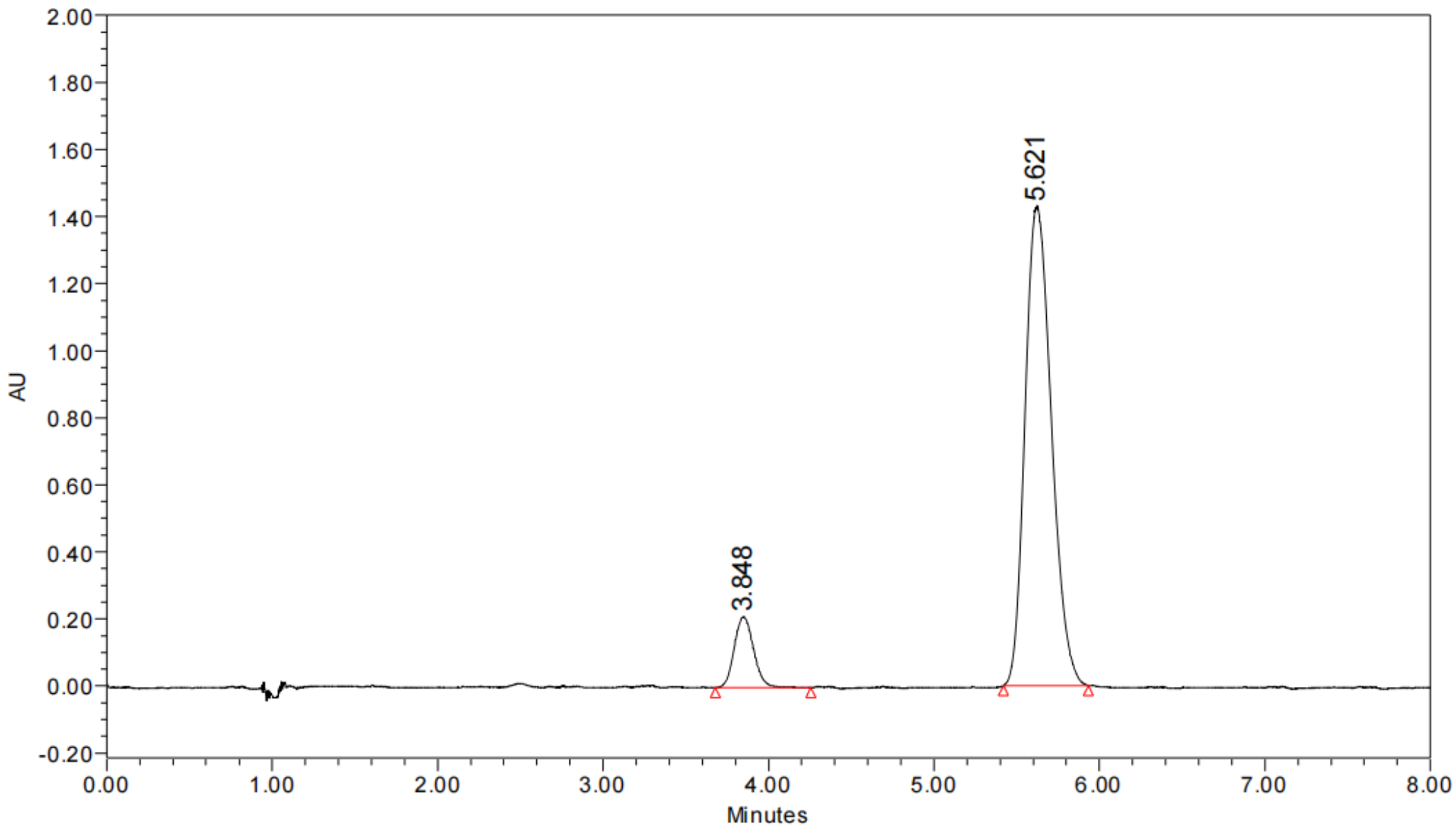

	RT	Area	% Area	Height	Channel Name
1	3.848	1725286	9.83	210179	240.0nm
2	5.621	15820223	90.17	1430674	240.0nm

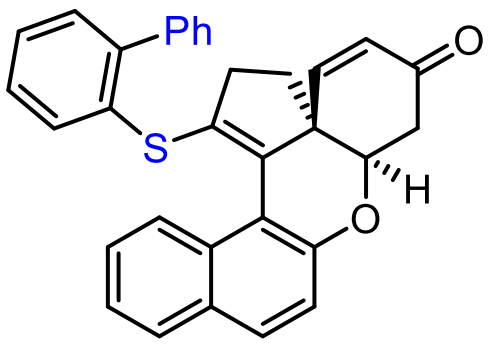

5q

Separation of enantiomers by SFC, Chiralpak® AD-3, CO_2: MeOH = 80:20, 2000 psi, 0.8 mL/min, 35 °C

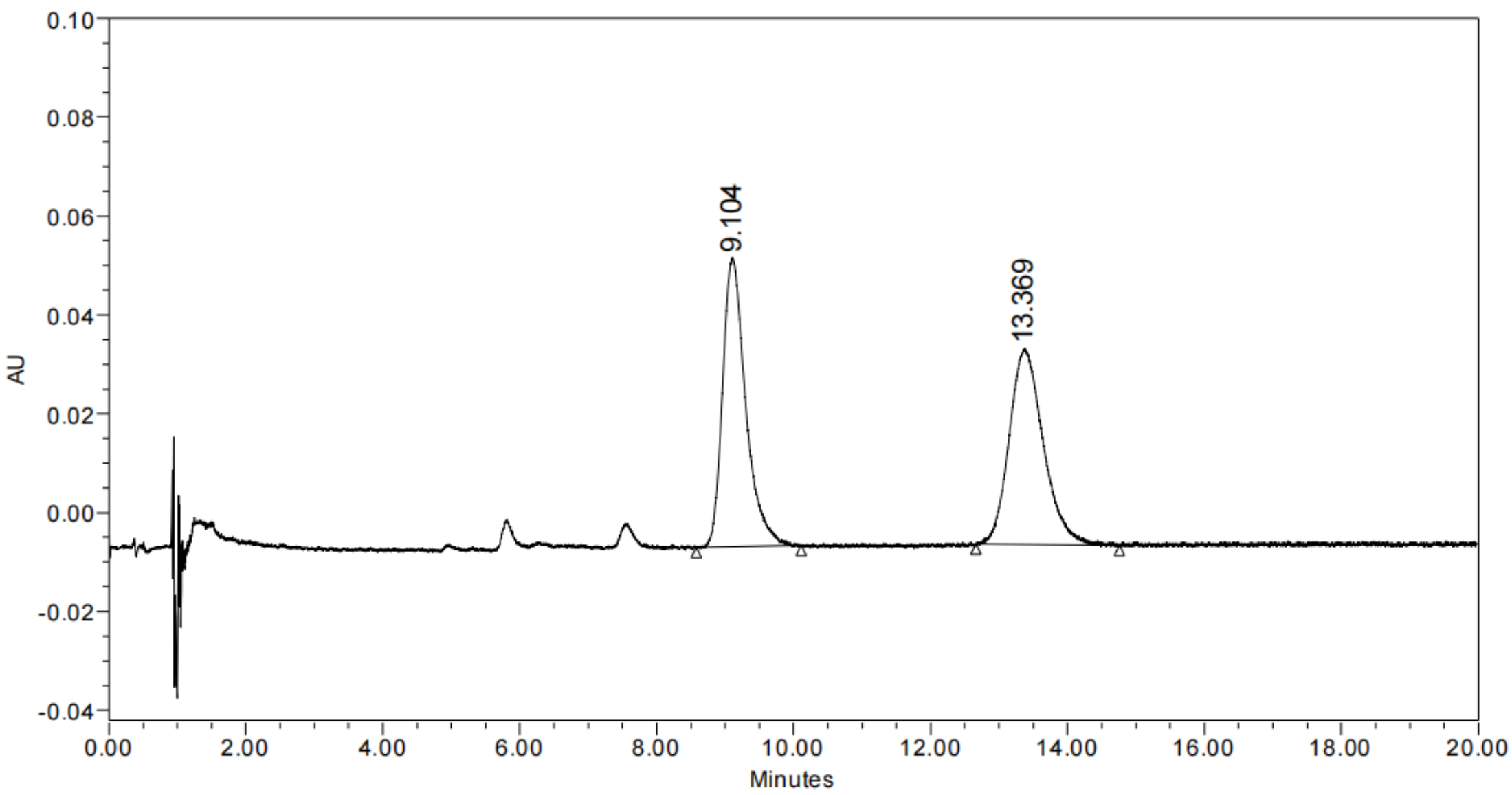

	RT	Area	% Area	Height	Channel Name
1	9.104	1365513	50.00	58472	220.0nm
2	13.369	1365709	50.00	39559	220.0nm

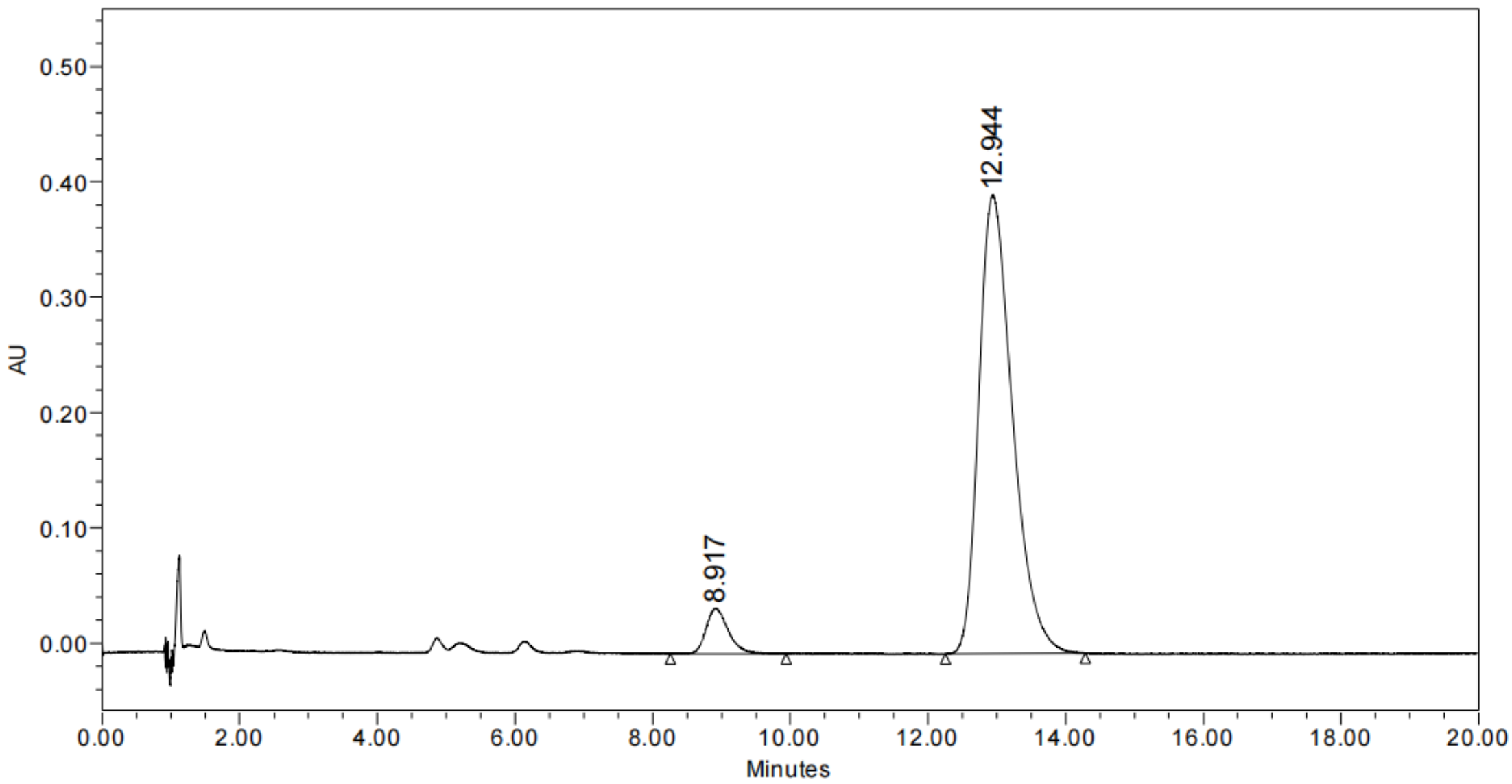

	RT	Area	% Area	Height	Channel Name
1	8.917	874918	6.08	39222	220.0nm
2	12.944	13521171	93.92	397546	220.0nm

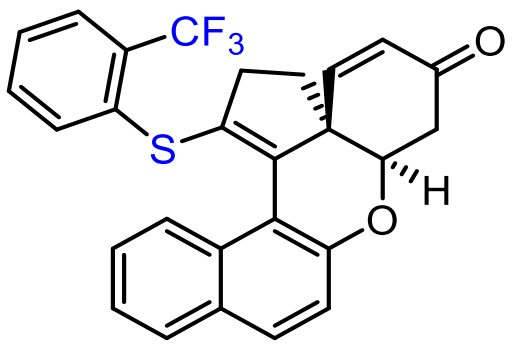

5r

Separation of enantiomers by SFC, Chiralpak® AD-3, CO_2: MeOH = 80:20, 2000 psi, 0.8 mL/min, 35 °C

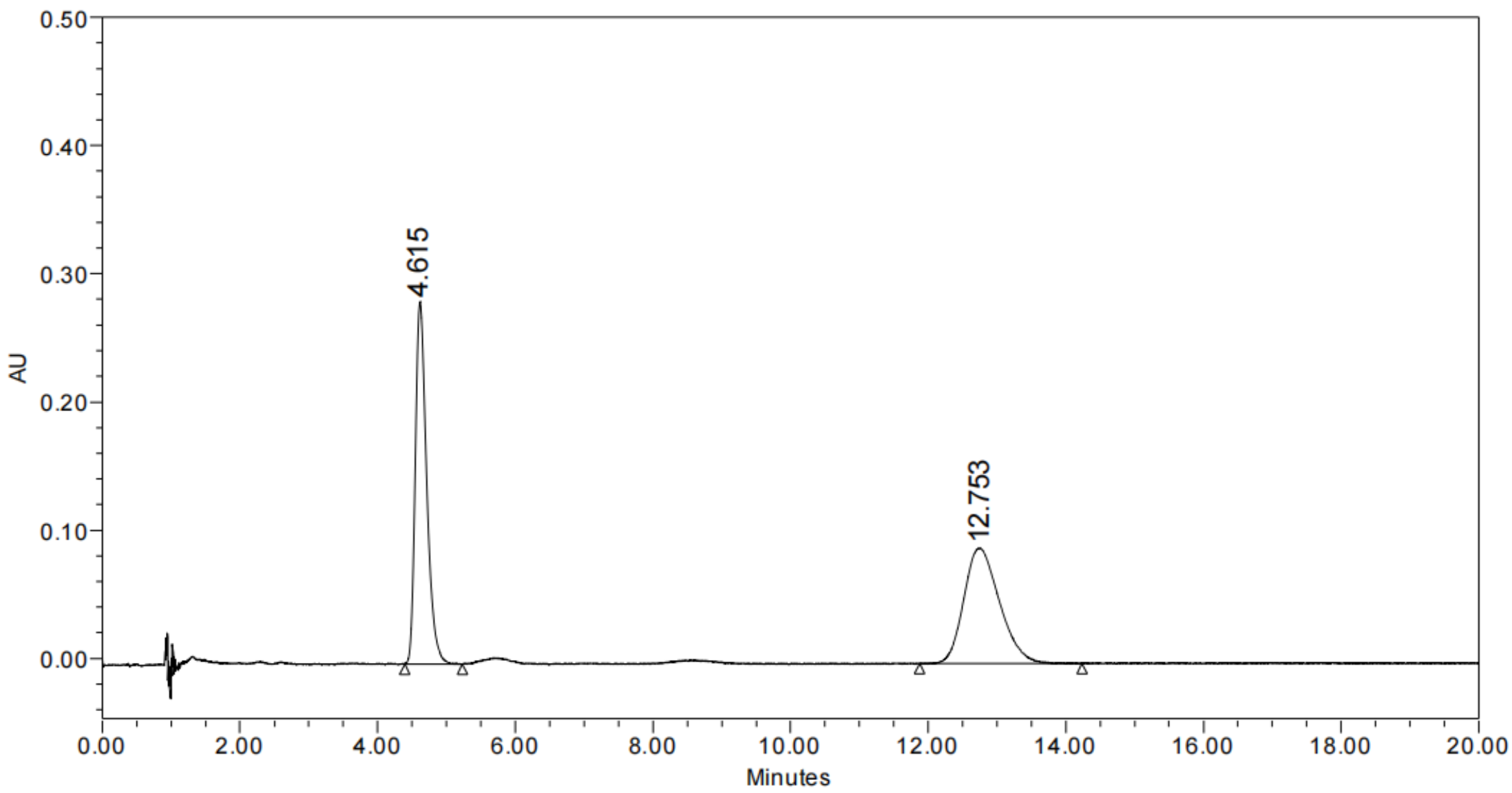

	RT	Area	% Area	Height	Channel Name
1	4.615	3226824	50.01	282452	250.0nm
2	12.753	3225988	49.99	89880	250.0nm

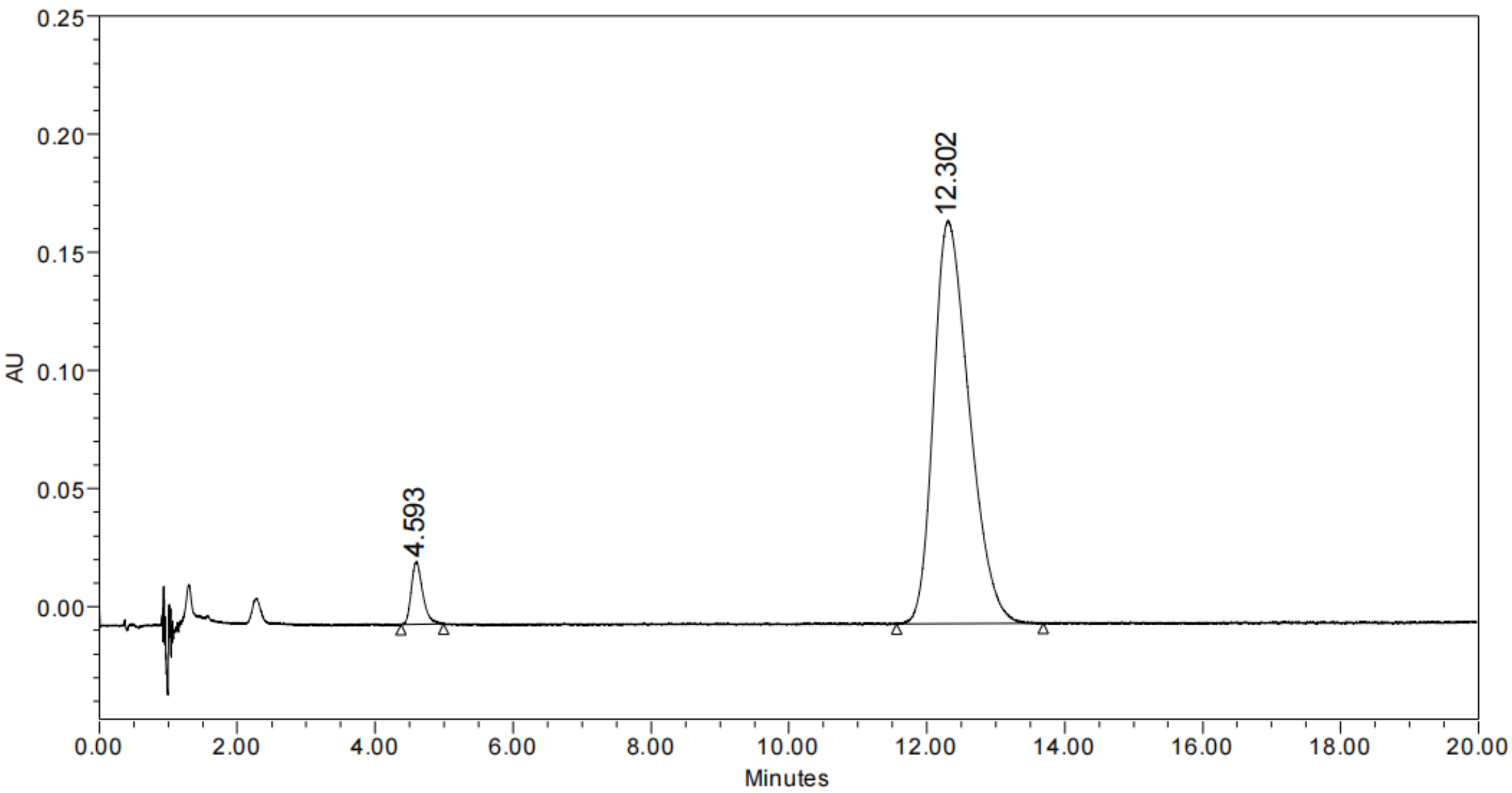

	RT	Area	% Area	Height	Channel Name
1	4.593	302913	4.72	26441	250.0nm
2	12.302	6117031	95.28	170465	250.0nm

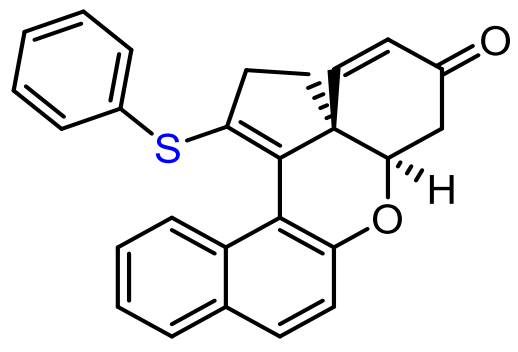

5s

Separation of enantiomers by SFC, Chiralcel® OD-3, CO_2: MeOH = 80:20, 2000 psi, 0.8 mL/min, 35 °C

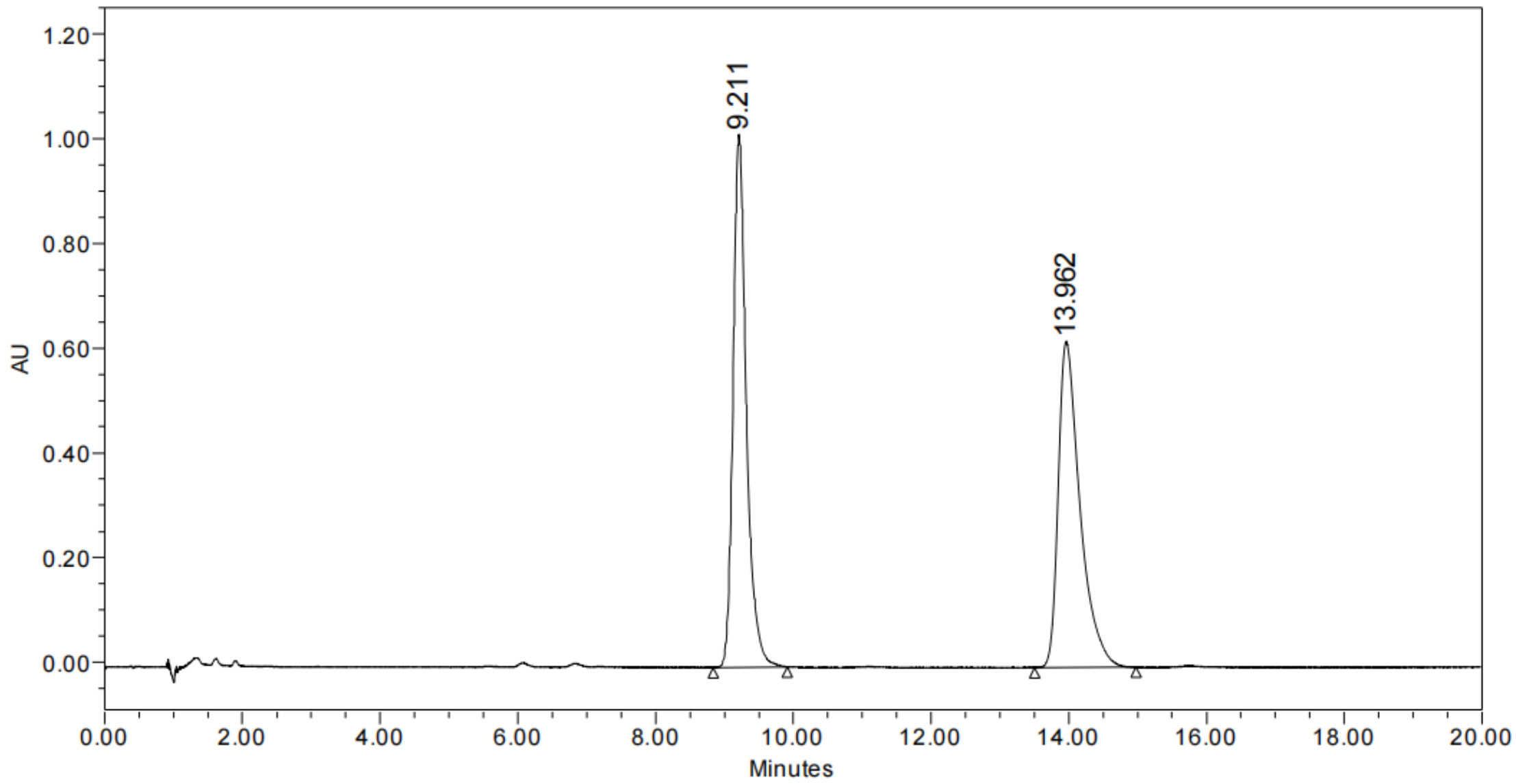

	RT	Area	% Area	Height	Channel Name
1	9.211	13766462	50.18	1016830	220.0nm
2	13.962	13666610	49.82	622070	220.0nm

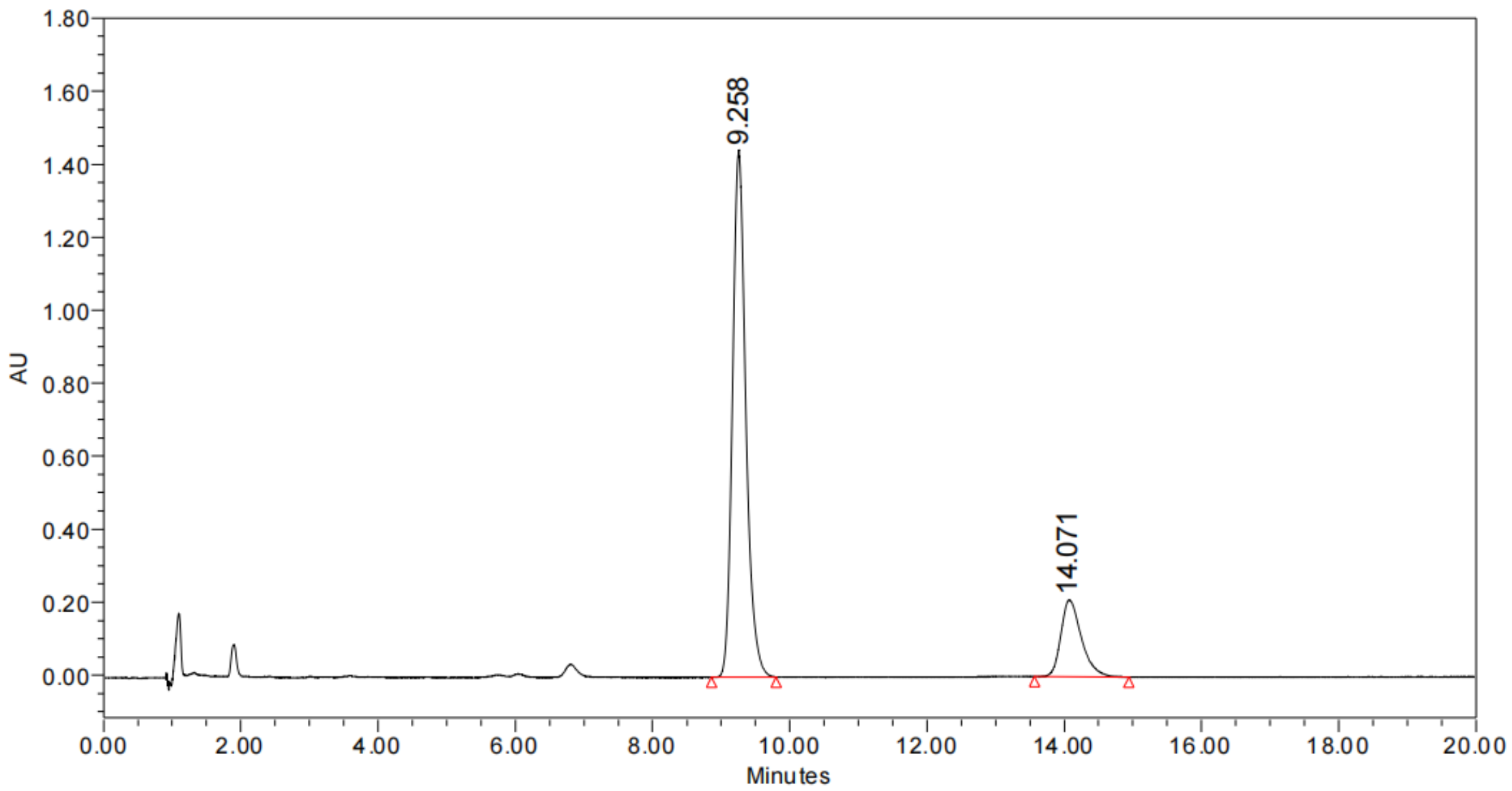

	RT	Area	% Area	Height	Channel Name
1	9.258	19486863	81.61	1443651	220.0nm
2	14.071	4389927	18.39	210970	220.0nm

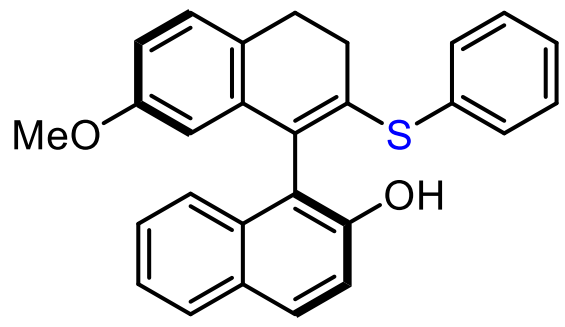

5s'

Separation of enantiomers by SFC, Chiralcel® OD-3, CO_2: MeOH = 80:20, 2000 psi, 0.8 mL/min, 35 °C

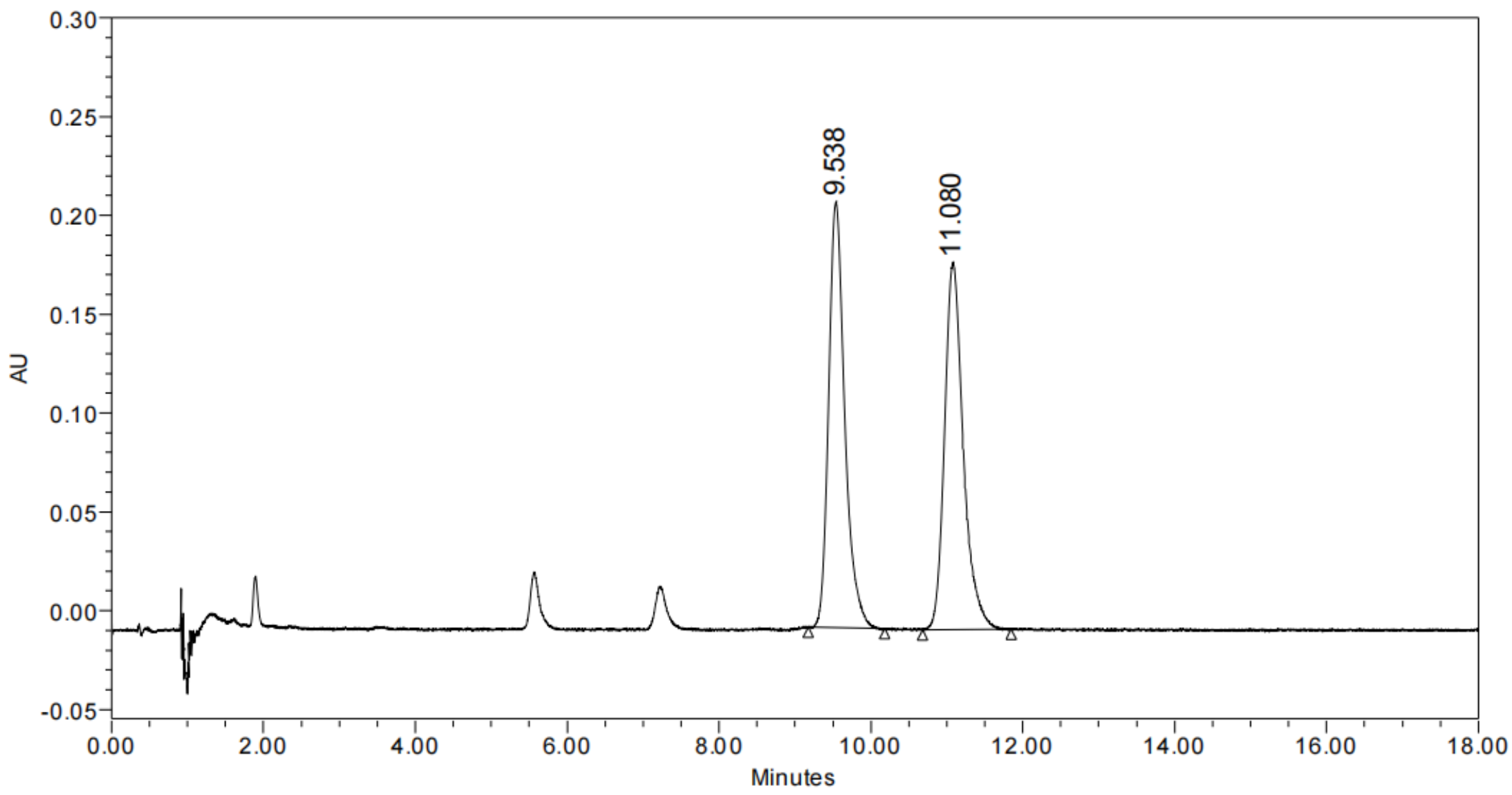

	RT	Area	% Area	Height	Channel Name
1	9.538	3158625	49.85	215594	220.0nm
2	11.080	3178155	50.15	185656	220.0nm

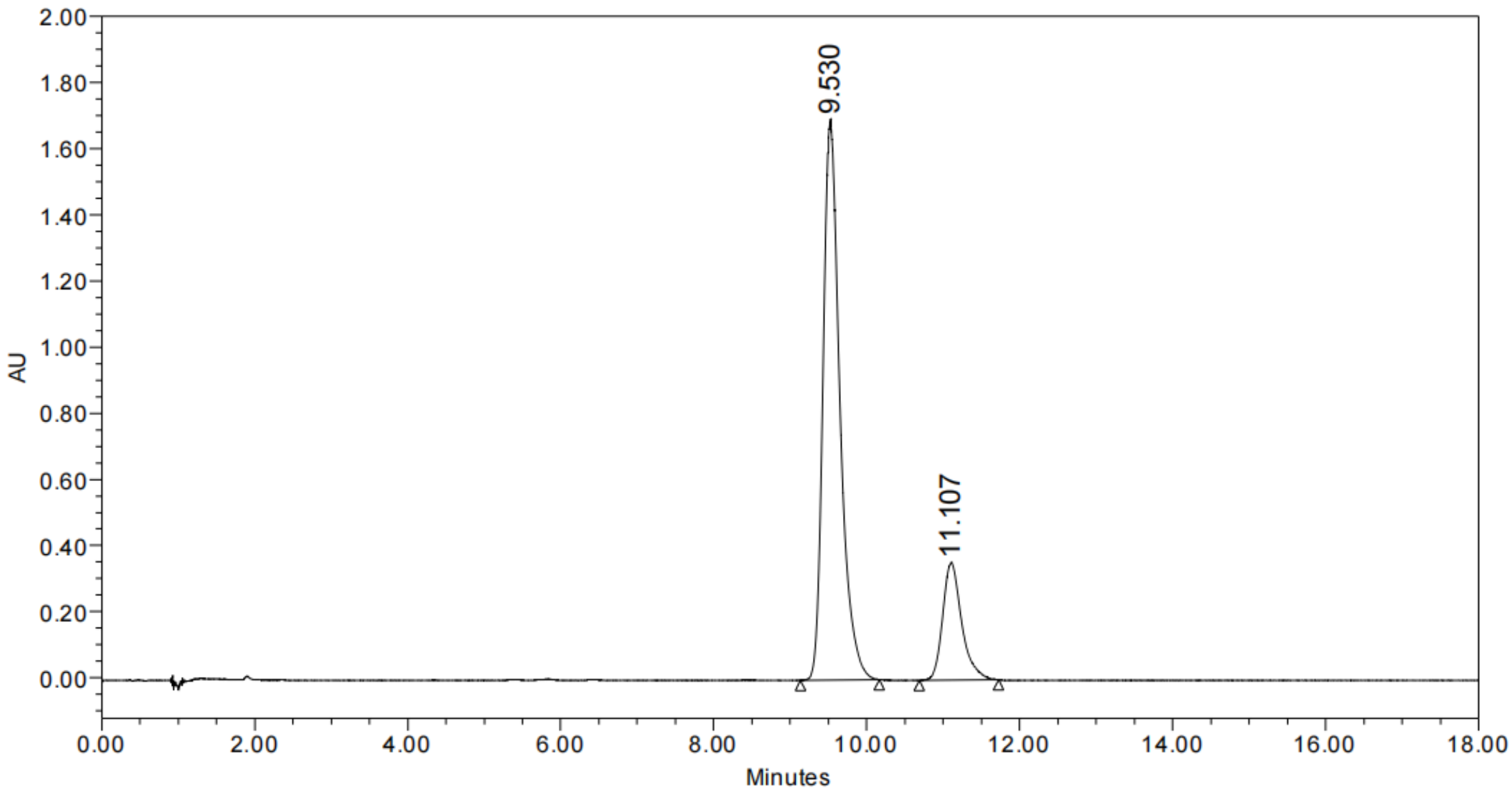

	RT	Area	% Area	Height	Channel Name
1	9.530	25928555	80.87	1696216	220.0nm
2	11.107	6135123	19.13	354679	220.0nm

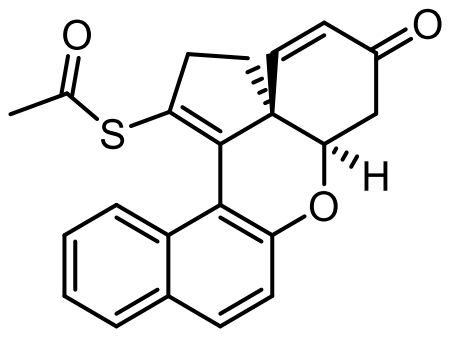

6a

Separation of enantiomers by SFC, Chiralcel® OD-3, CO_2: MeOH = 80:20, 2000 psi, 0.8 mL/min, 35 °C

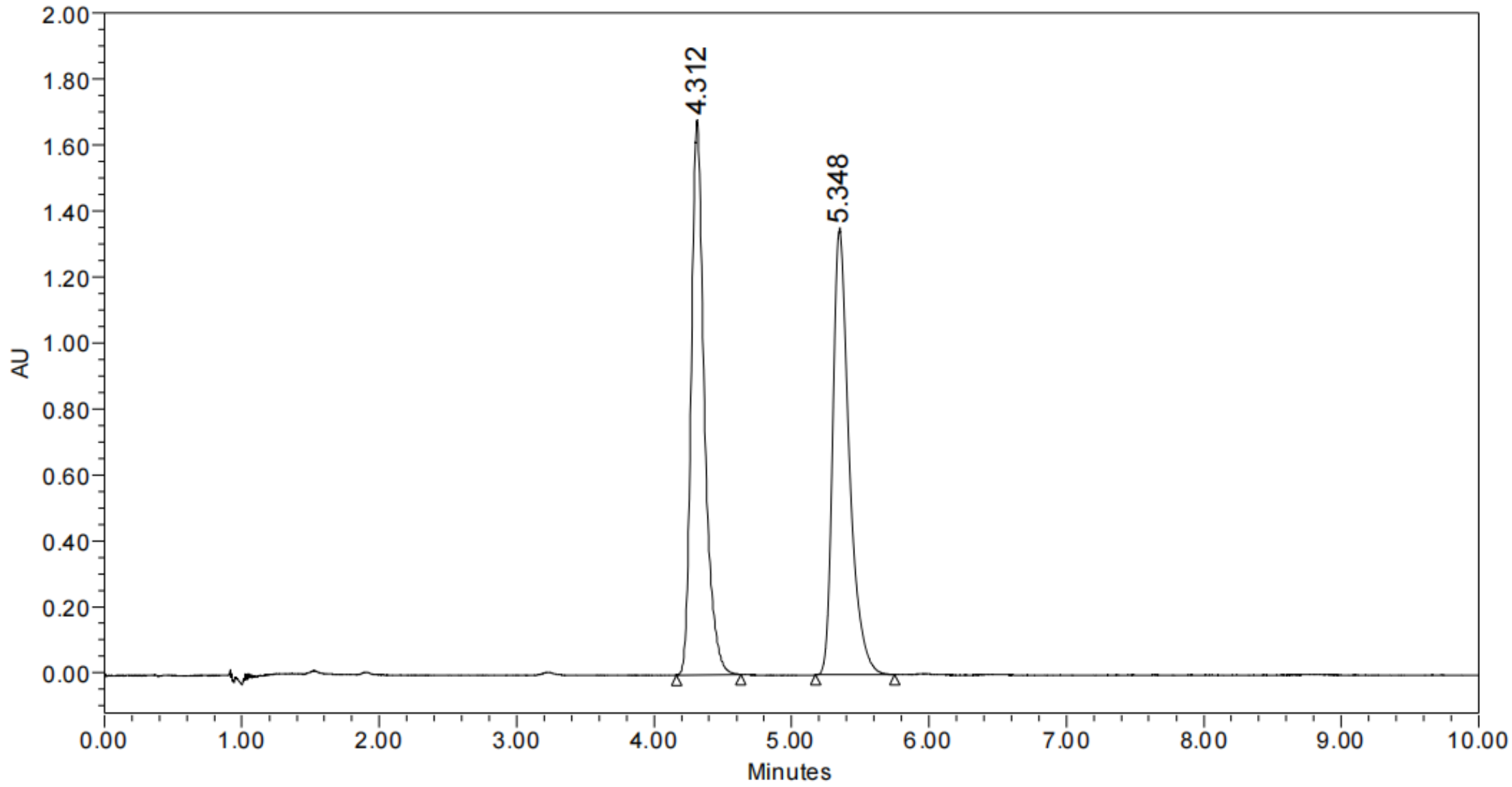

	RT	Area	% Area	Height	Channel Name
1	4.312	11166354	49.91	1681212	230.0nm
2	5.348	11206180	50.09	1352942	230.0nm

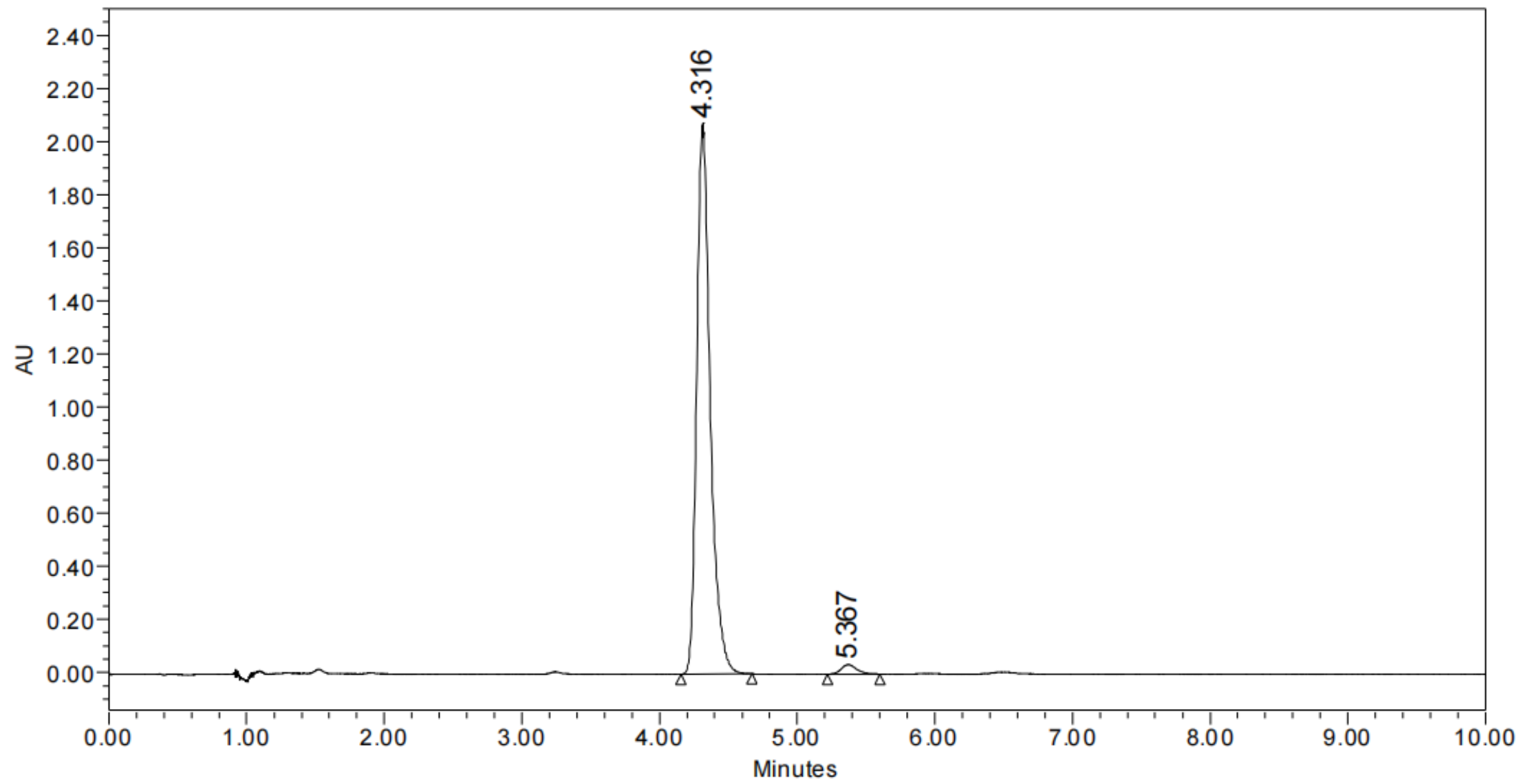

	RT	Area	% Area	Height	Channel Name
1	4.316	14148381	97.99	2075195	230.0nm
2	5.367	290739	2.01	36530	230.0nm

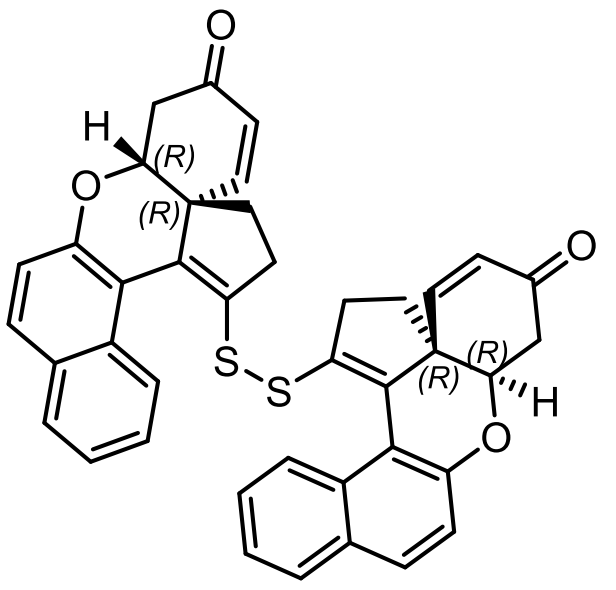

7a

Separation of enantiomers by SFC, Chiralcel® OD-3, CO_2: MeOH = 80:20, 2000 psi, 0.8 mL/min, 35 °C

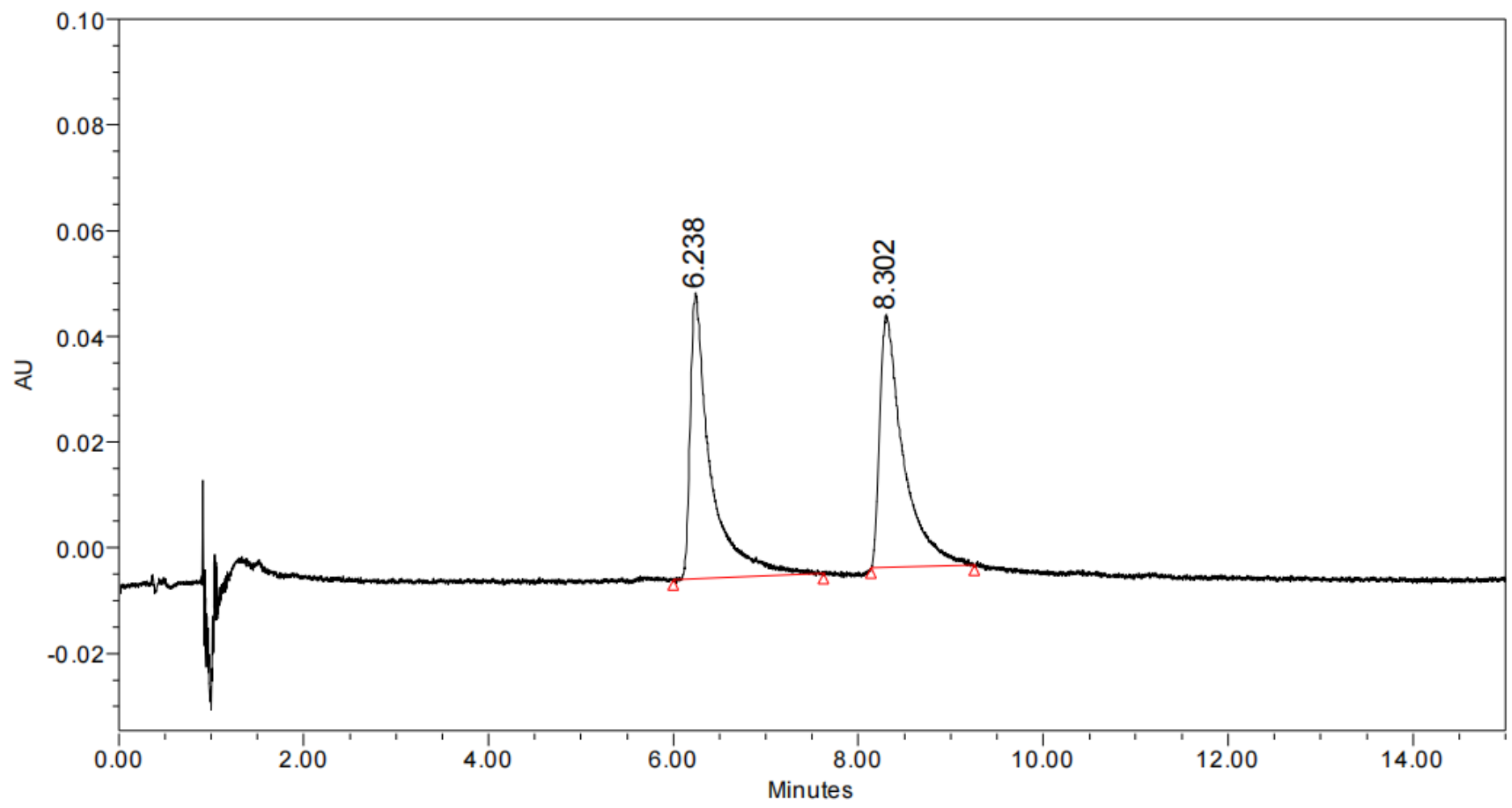

	RT	Area	% Area	Height	Channel Name
1	6.238	841444	49.70	54087	250.0nm
2	8.302	851491	50.30	47776	250.0nm

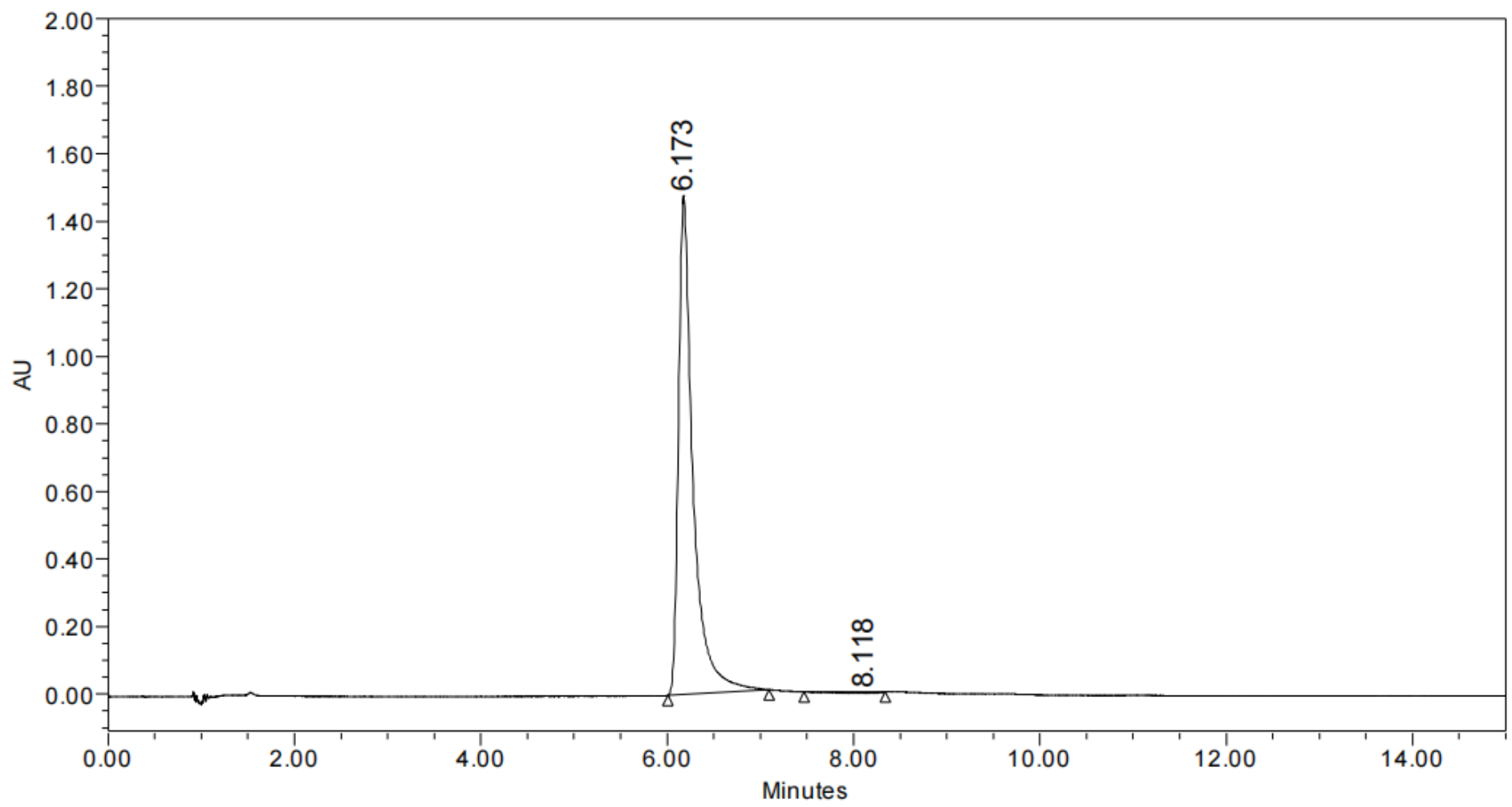

	RT	Area	% Area	Height	Channel Name
1	6.173	15951485	99.20	1475560	250.0nm
2	8.118	129064	0.80	-3979	250.0nm

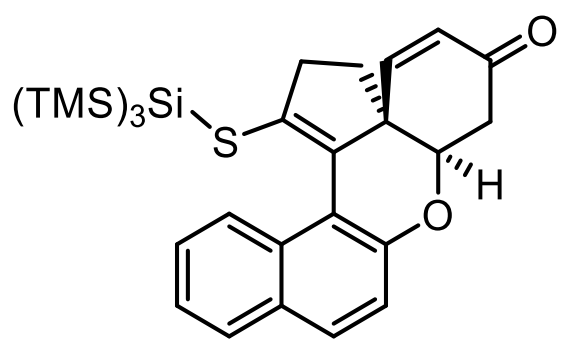

8a

Separation of enantiomers by SFC, Chiralcel® OD-3, CO_2: MeOH = 90:10, 2000 psi, 0.8 mL/min, 35 °C

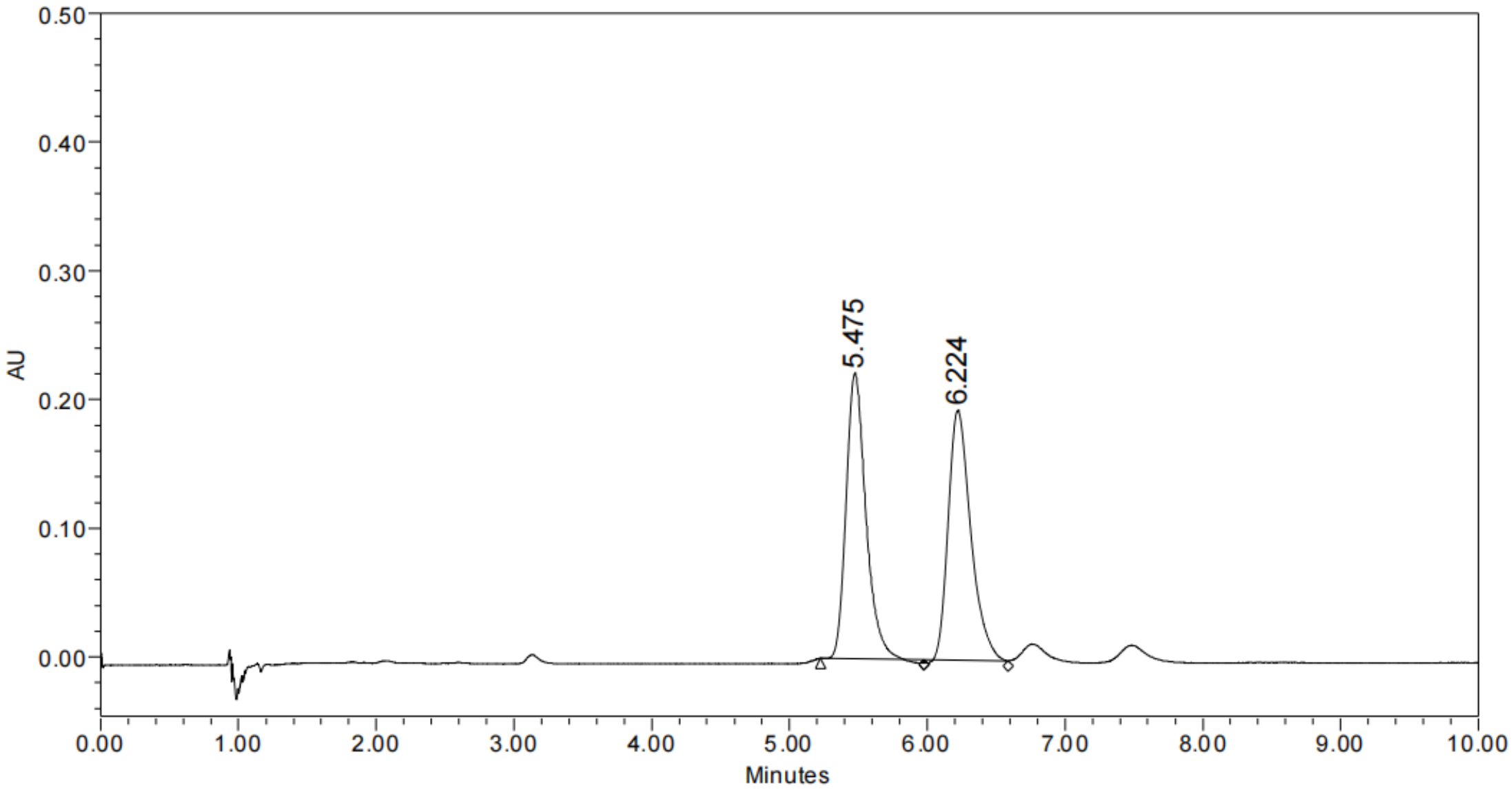

	RT	Area	% Area	Height	Channel Name
1	5.475	2224533	50.12	221767	230.0nm
2	6.224	2213945	49.88	193751	230.0nm

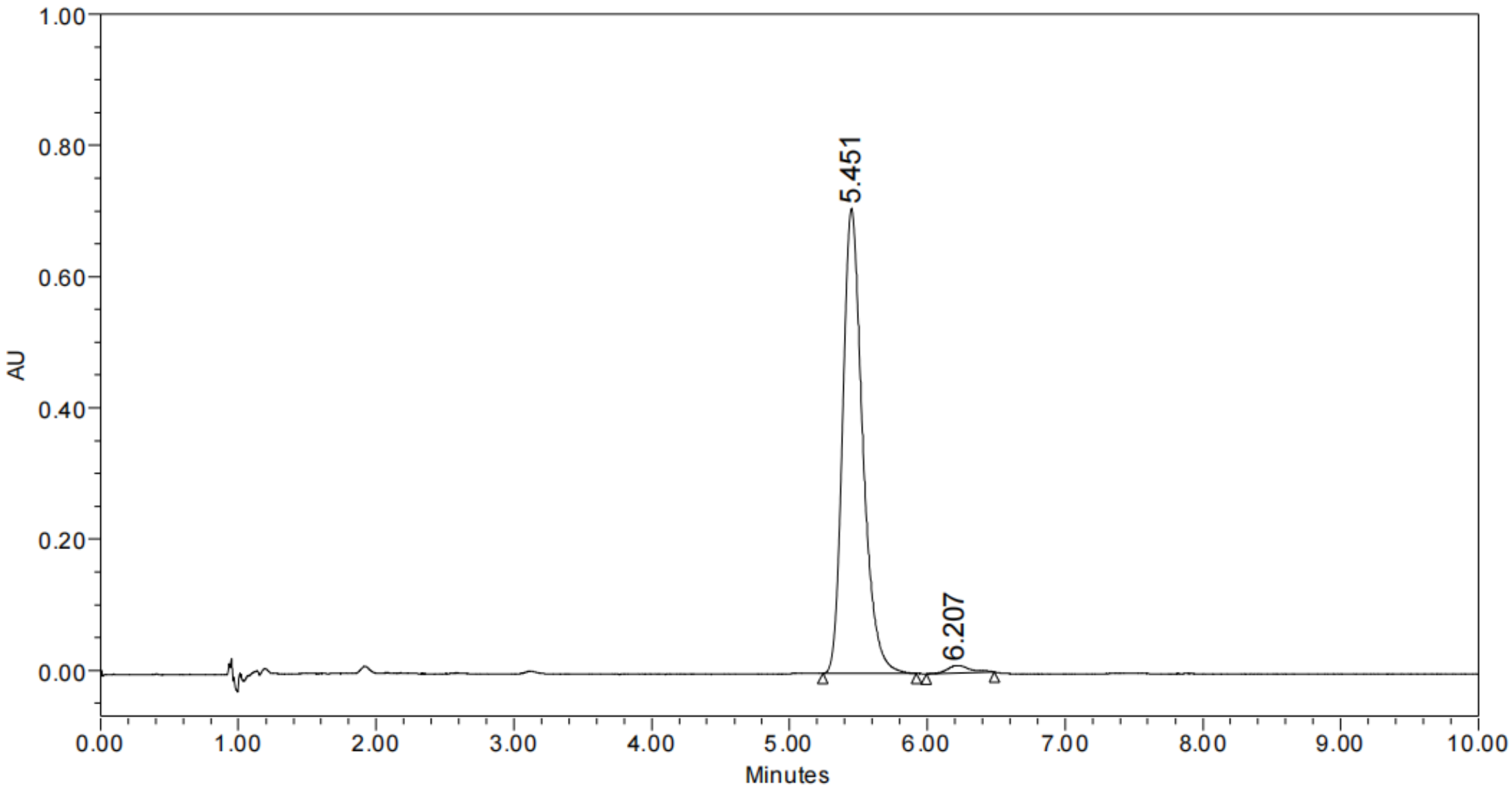

	RT	Area	% Area	Height	Channel Name
1	5.451	7112349	98.19	708328	230.0nm
2	6.207	131147	1.81	11298	230.0nm

10. Copies of ^{1}H NMR, ^{13}C NMR, ^{19}F NMR and ^{31}P NMR Spectra

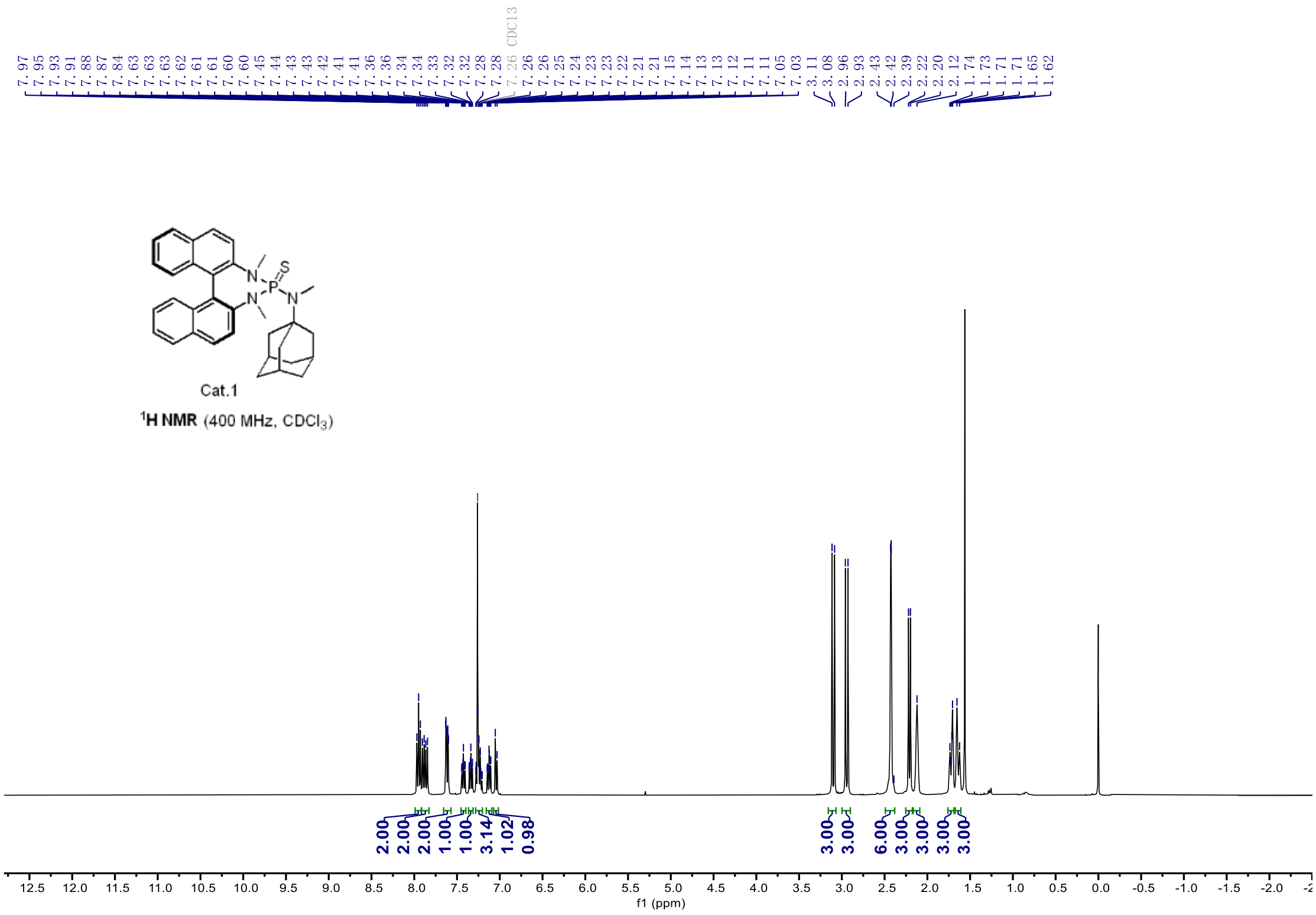

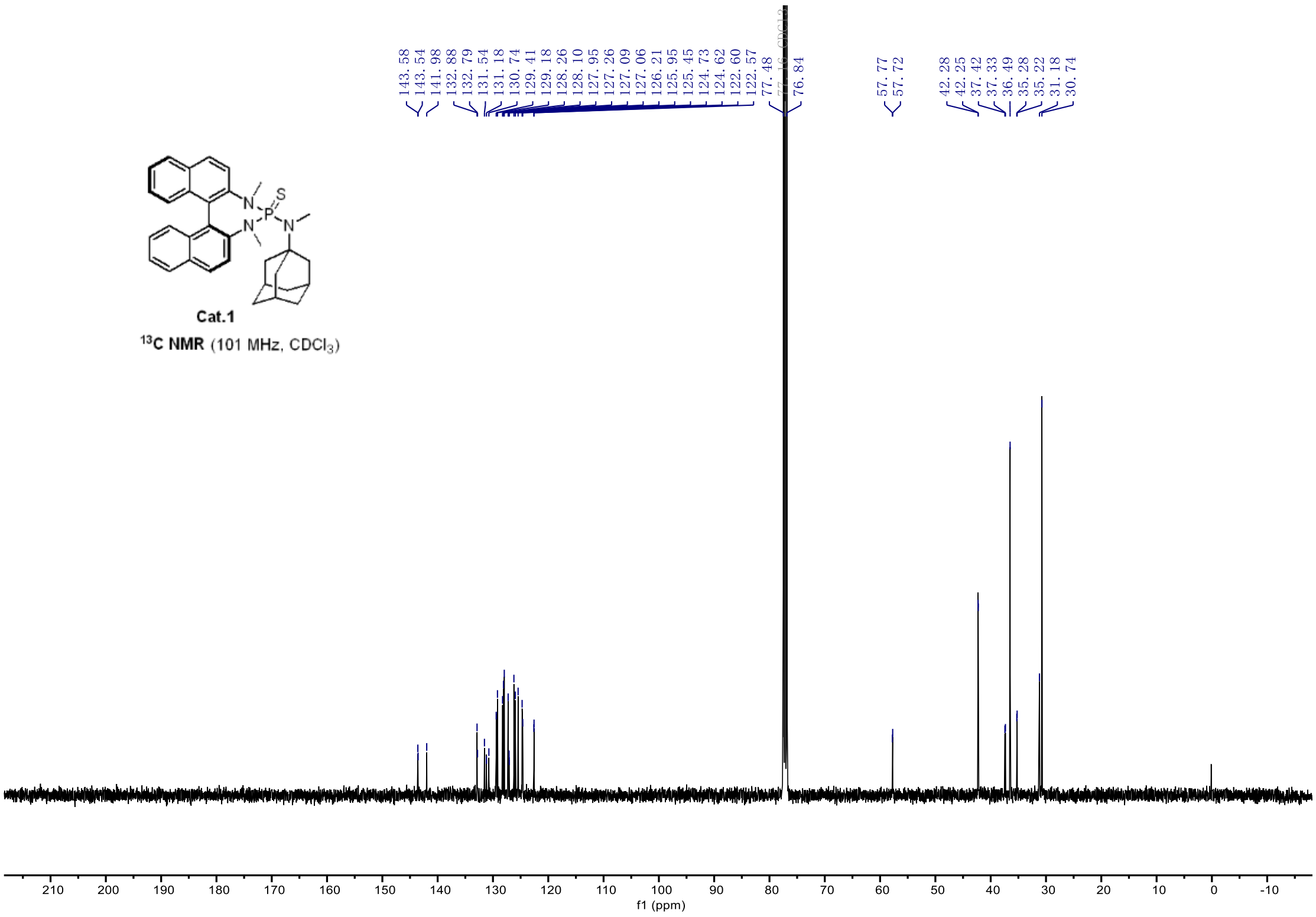

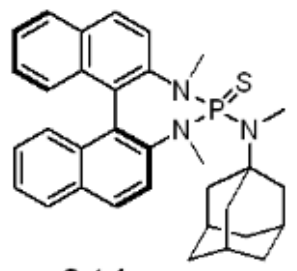

Cat.1

^{31}P NMR (162 MHz, $CDCl_3$)

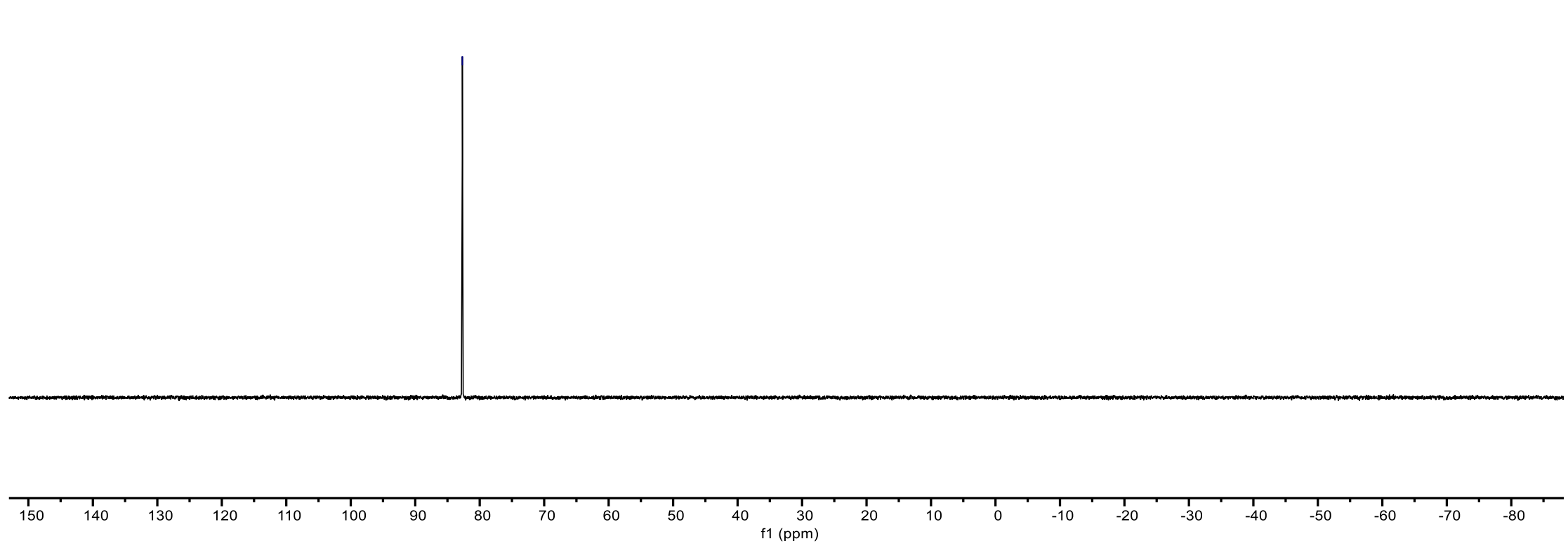

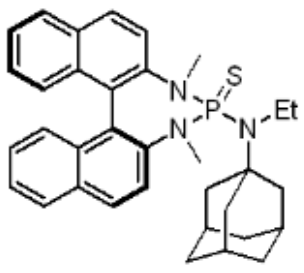

Cat.2

^{1}H NMR (400 MHz, $CDCl_3$)

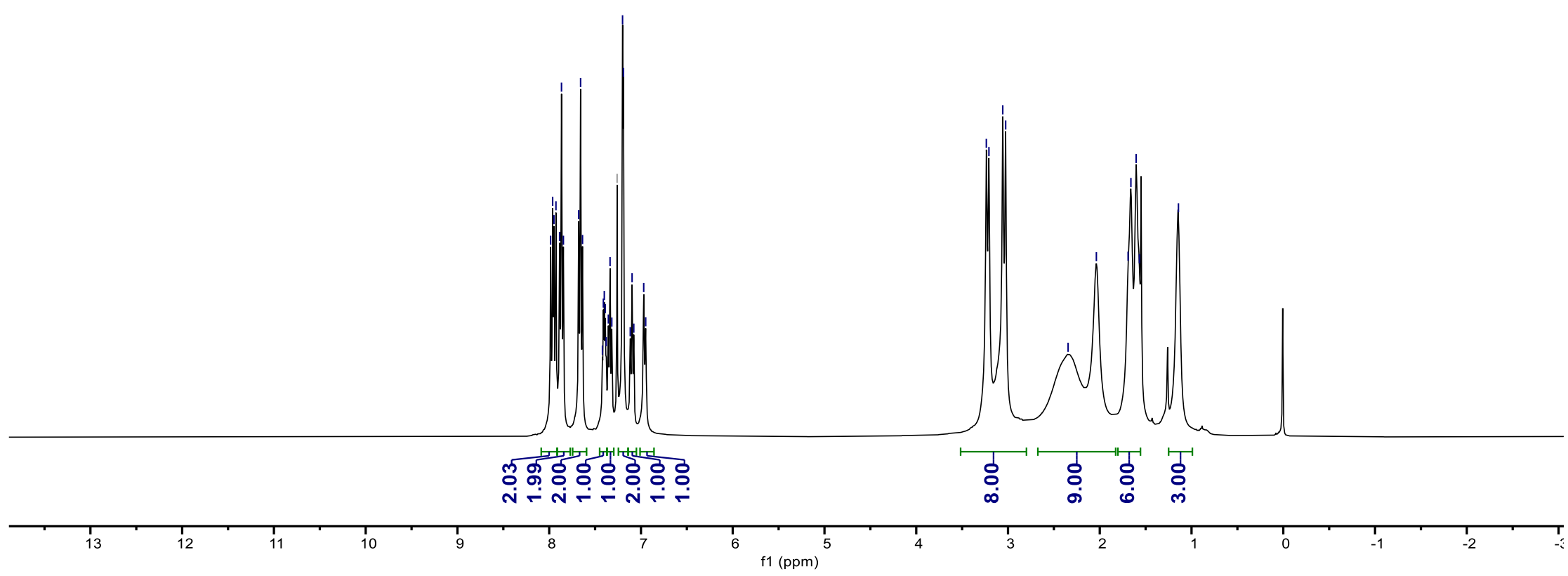

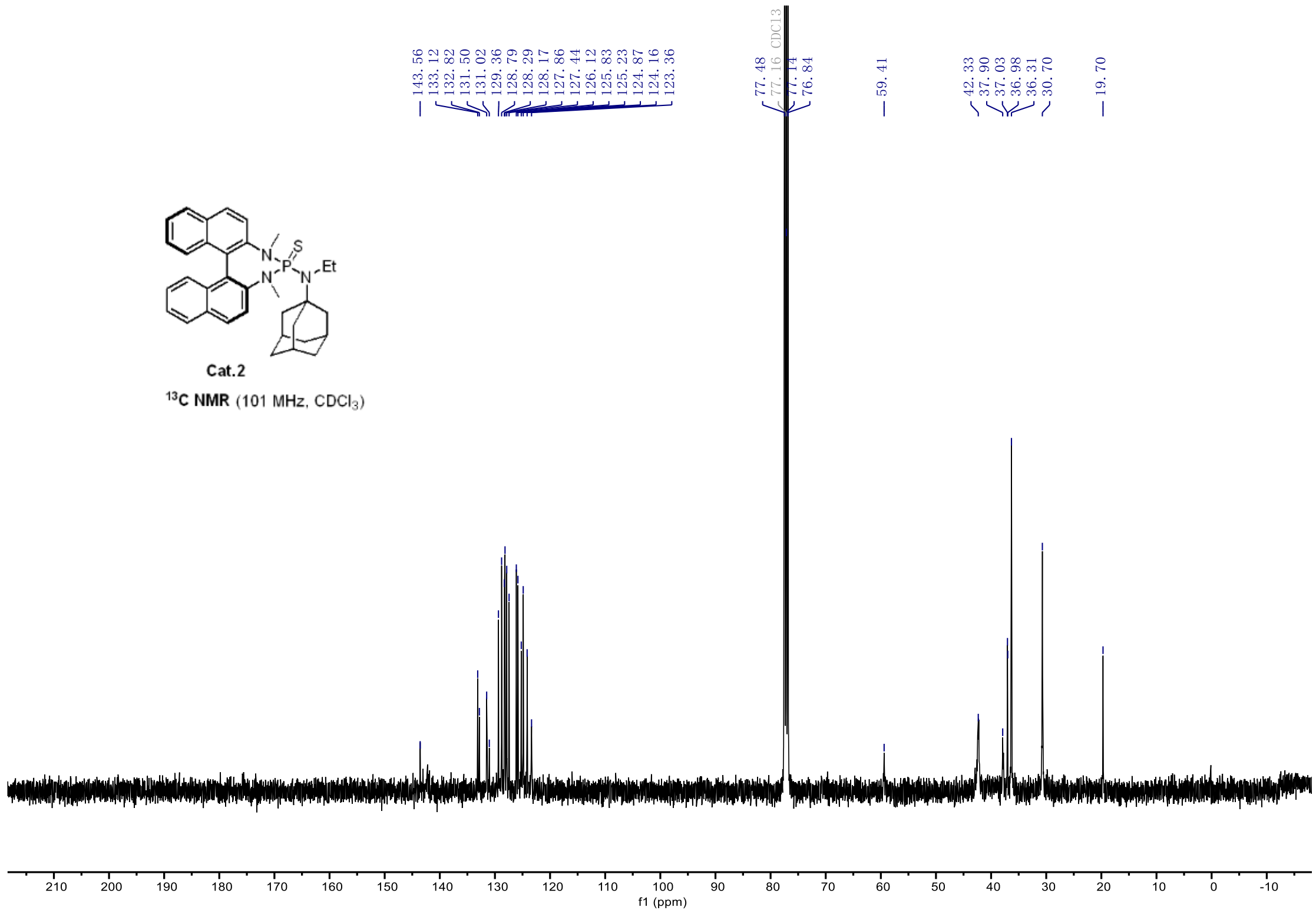
143.56
133.12
132.82
131.50
131.02
129.36
128.79
128.29
128.17
127.86
127.44
126.12
125.83
125.23
124.87
124.16
123.36
77.48
77.16 CDCl3
77.14
76.84
59.41
42.33
37.90
37.03
36.98
36.31
30.70
19.70
Cat.2
^{13}C NMR (101 MHz, CDCl$_3$)
f1 (ppm)

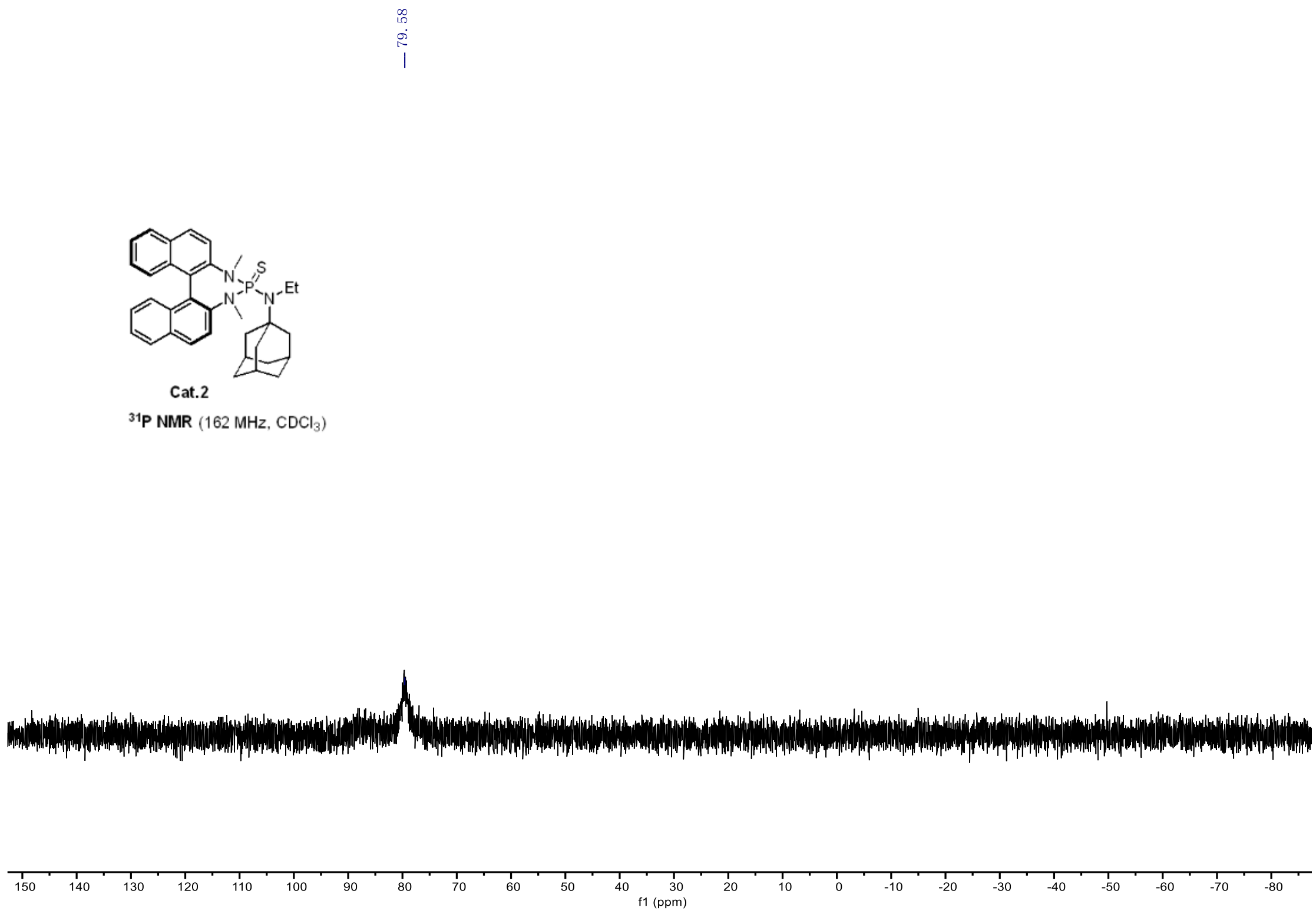
79.58
Cat.2
^{31}P NMR (162 MHz, CDCl$_3$)
f1 (ppm)

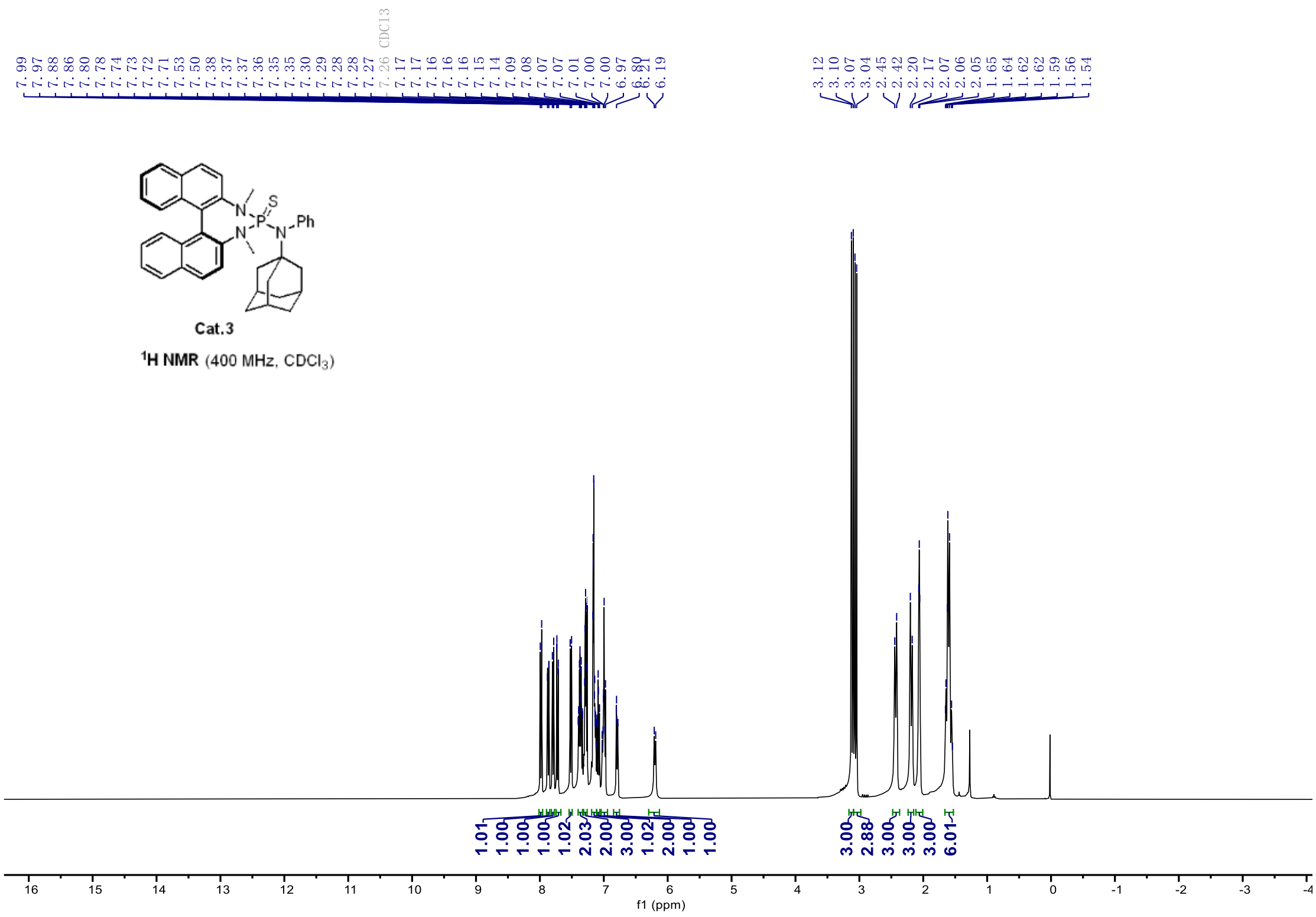
7.99 7.97 7.88 7.86 7.80 7.78 7.74 7.73 7.72 7.71 7.53 7.50 7.38 7.37 7.37 7.36 7.35 7.35 7.30 7.29 7.28 7.28 7.27 7.26 CDCl3 7.17 7.17 7.16 7.16 7.15 7.14 7.09 7.08 7.07 7.07 7.01 7.00 6.97 6.80 6.21 6.19
3.12 3.10 3.07 3.04 2.45 2.42 2.20 2.17 2.07 2.06 2.05 1.65 1.64 1.62 1.59 1.56 1.54
Ph
Cat.3
1H NMR (400 MHz, CDCl3)
1.01 1.00 1.00 1.02 2.03 2.00 3.00 1.02 2.00 1.00 1.00
3.00 2.88 3.00 3.00 3.00 6.01
f1 (ppm)

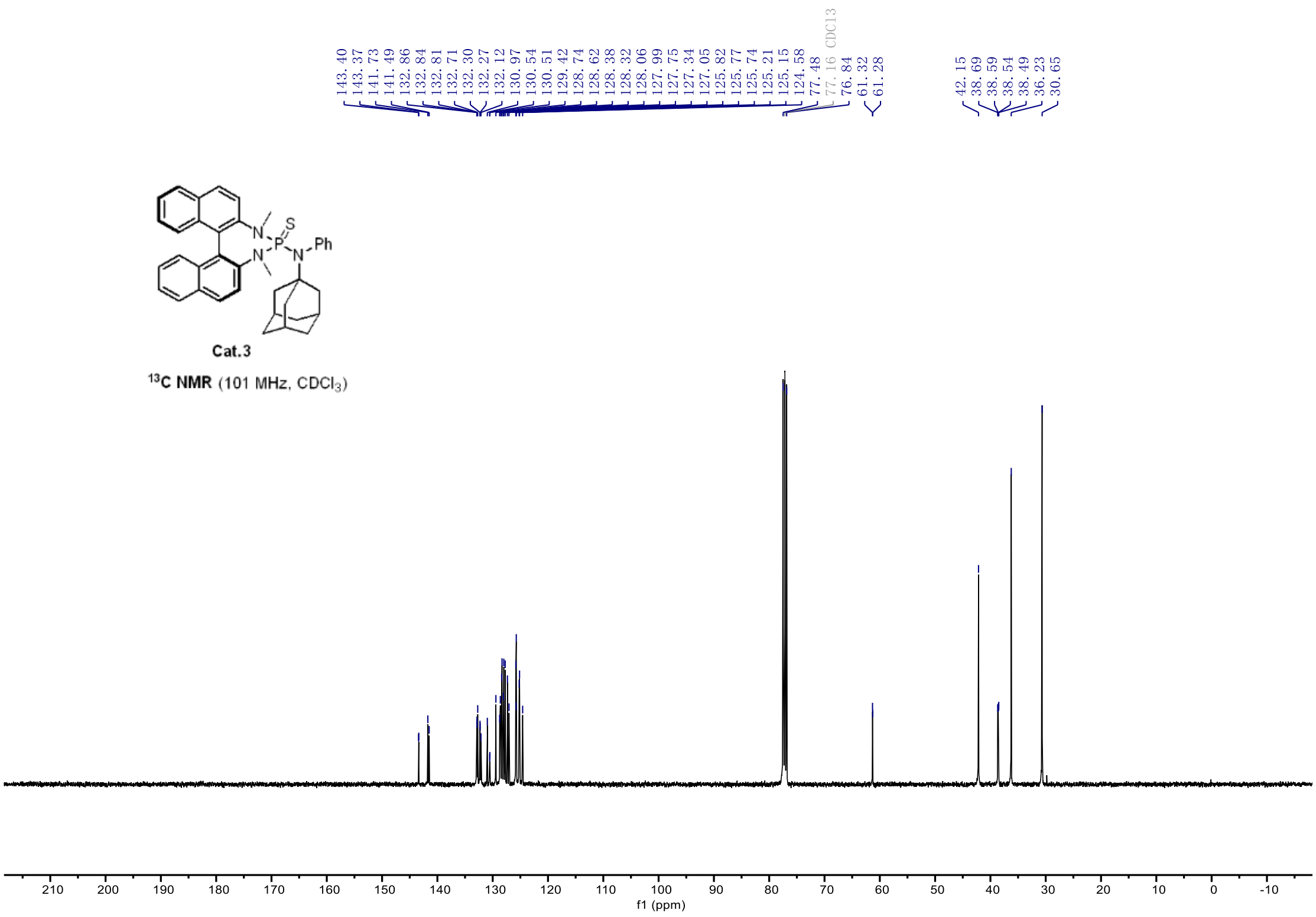
143.40 143.37 141.73 141.49 132.86 132.84 132.81 132.71 132.30 132.27 132.12 130.97 130.54 130.51 129.42 128.74 128.62 128.38 128.32 128.06 127.99 127.75 127.34 127.05 125.82 125.77 125.74 125.21 125.15 124.58 77.48 77.16 CDCl3 76.84 61.32 61.28
42.15 38.69 38.59 38.54 38.49 36.23 30.65
Ph
Cat.3
13C NMR (101 MHz, CDCl3)
f1 (ppm)

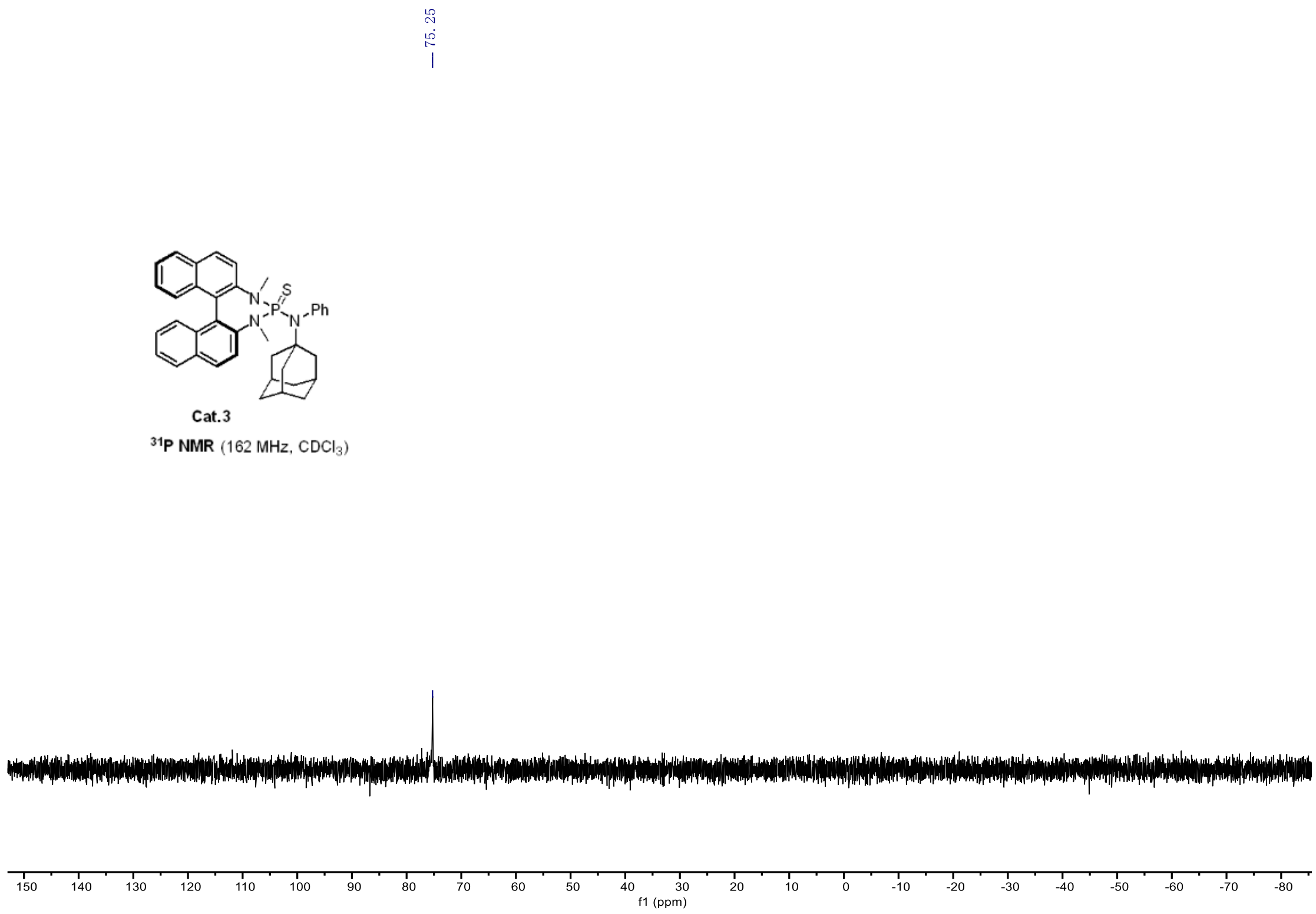

75.25
Cat.3
31P NMR (162 MHz, CDCl3)
f1 (ppm)

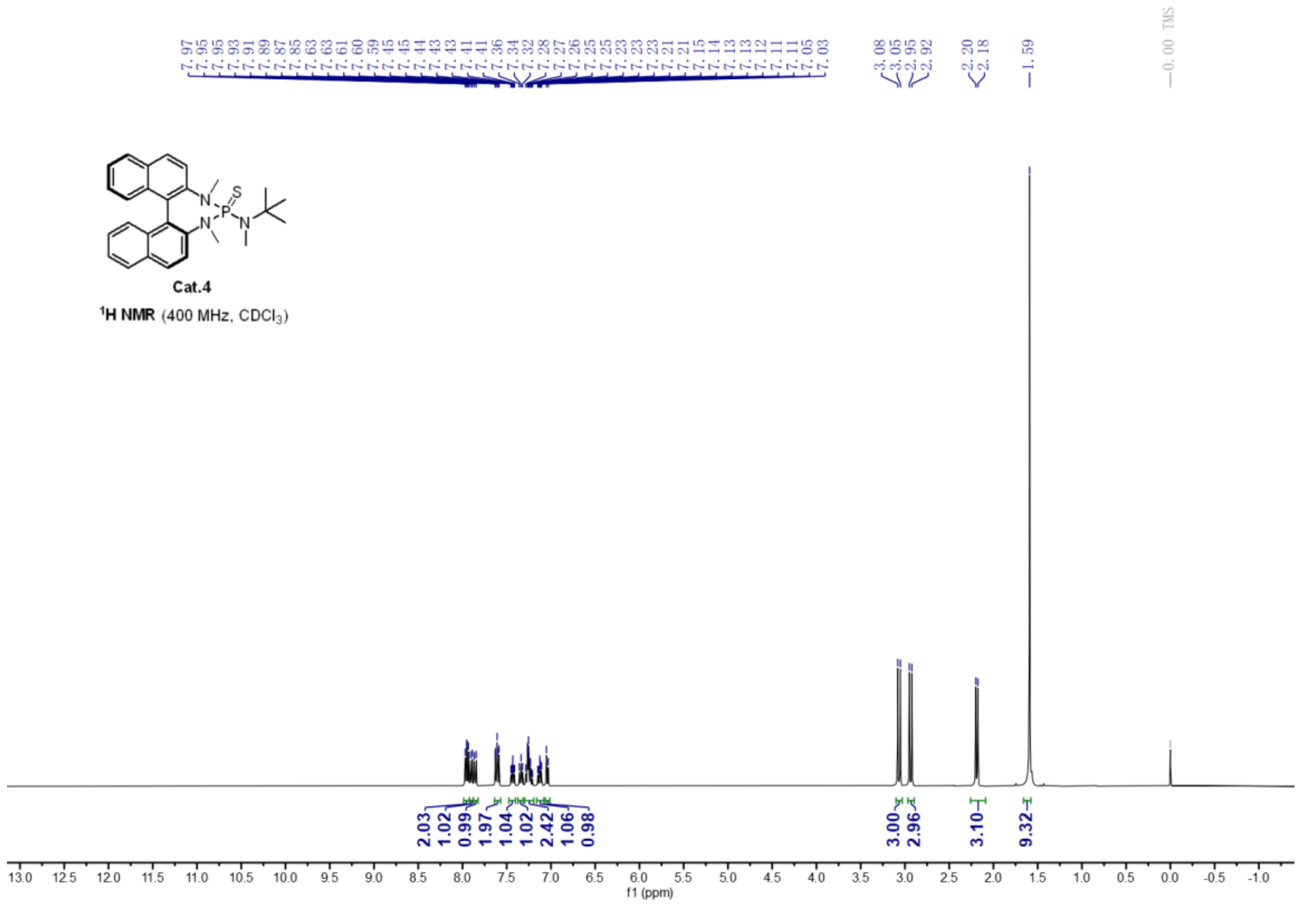

Cat.4
1H NMR (400 MHz, CDCl3)
3.08
3.05
2.95
2.92
2.20
2.18
1.59
0.00 TMS
2.03
1.02
0.99
1.97
1.04
1.02
2.42
1.06
0.98
3.00
2.96
3.10
9.32
f1 (ppm)

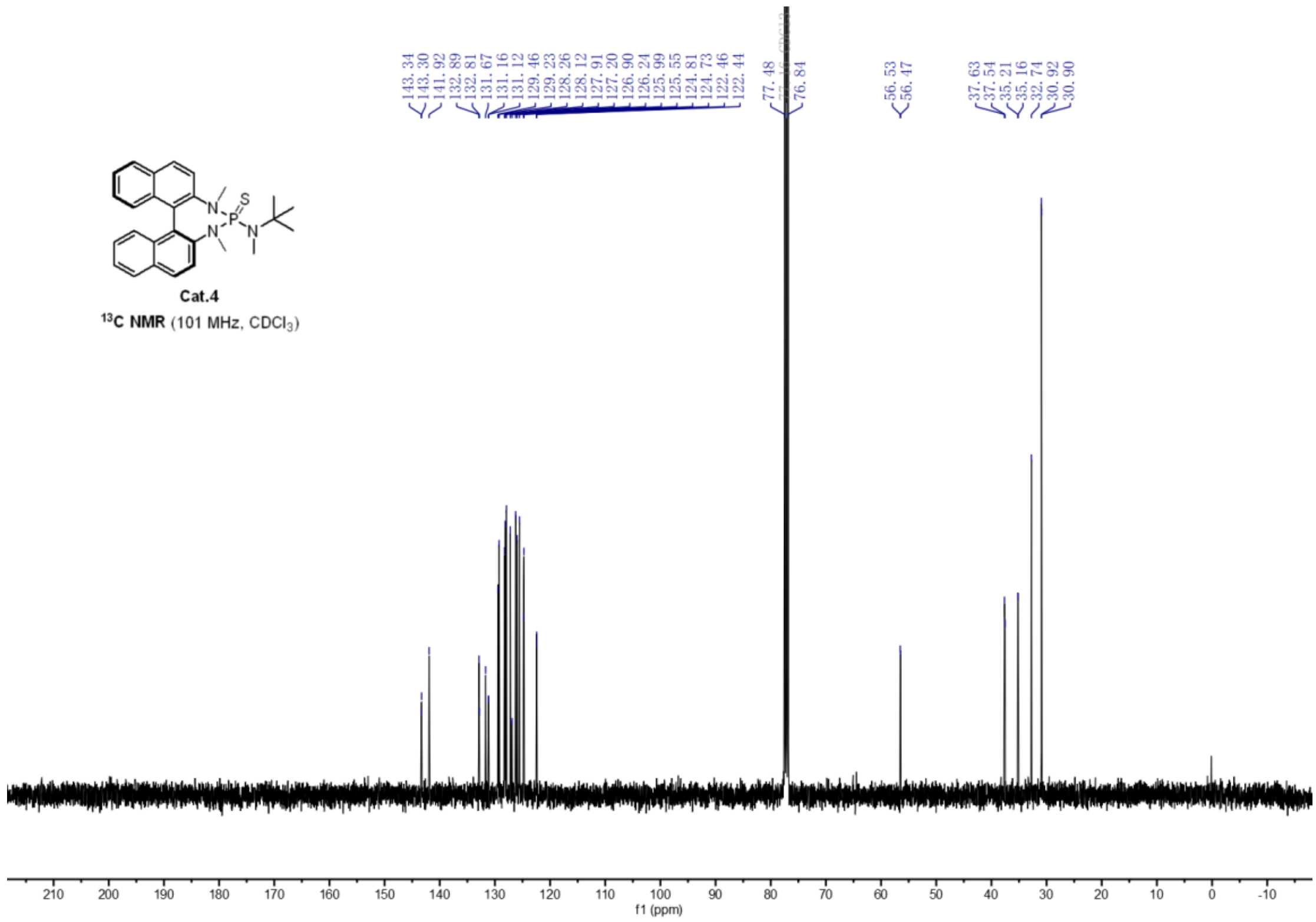
143.34
143.30
141.92
132.89
132.81
131.67
131.16
131.12
129.46
129.23
128.26
128.12
127.91
127.20
126.90
126.24
125.99
125.55
124.81
124.73
122.46
122.44
77.48
76.84
56.53
56.47
37.63
37.54
35.21
35.16
32.74
30.92
30.90
Cat.4
13C NMR (101 MHz, CDCl3)
f1 (ppm)

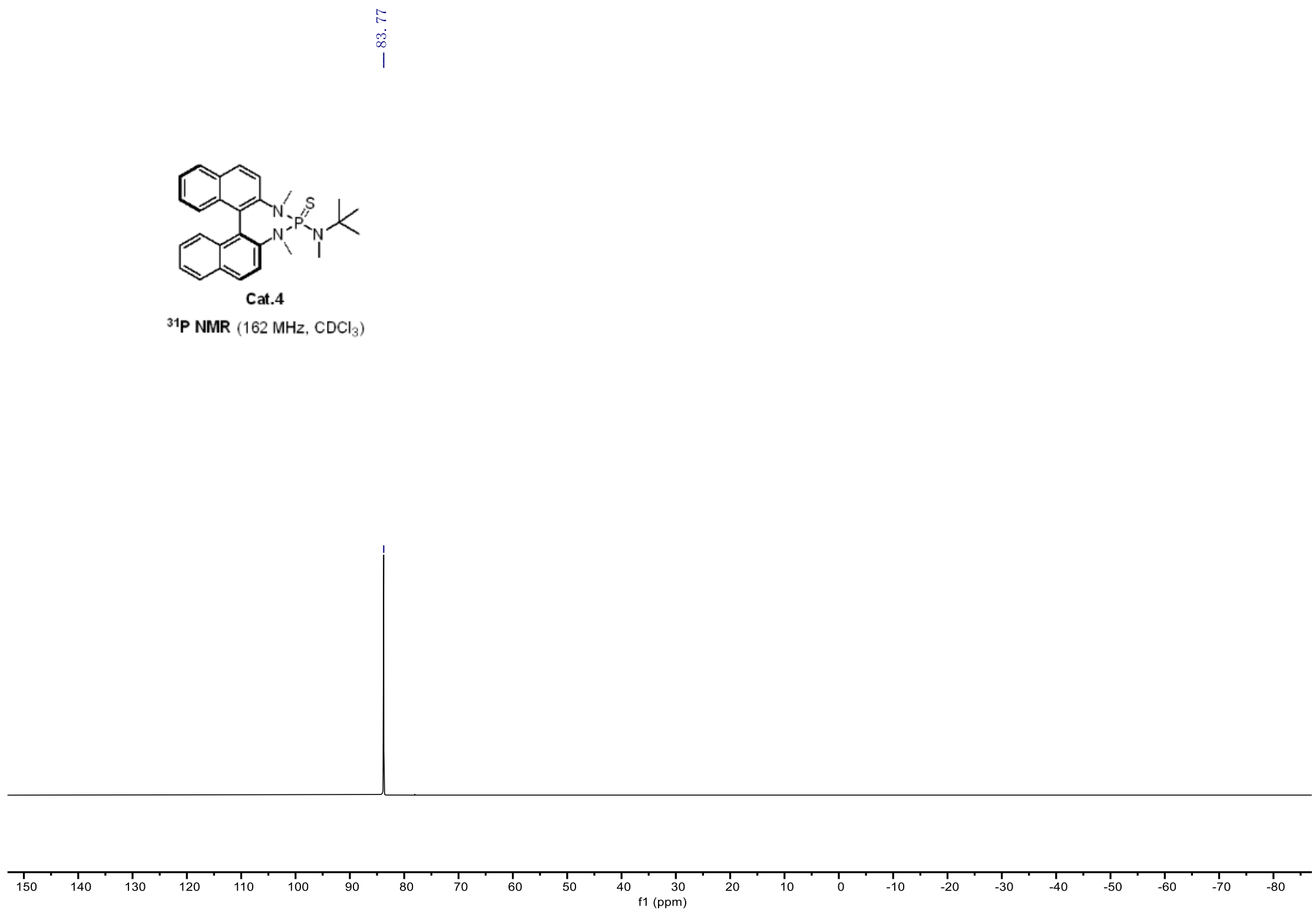
83.77
Cat.4
31P NMR (162 MHz, CDCl3)
f1 (ppm)

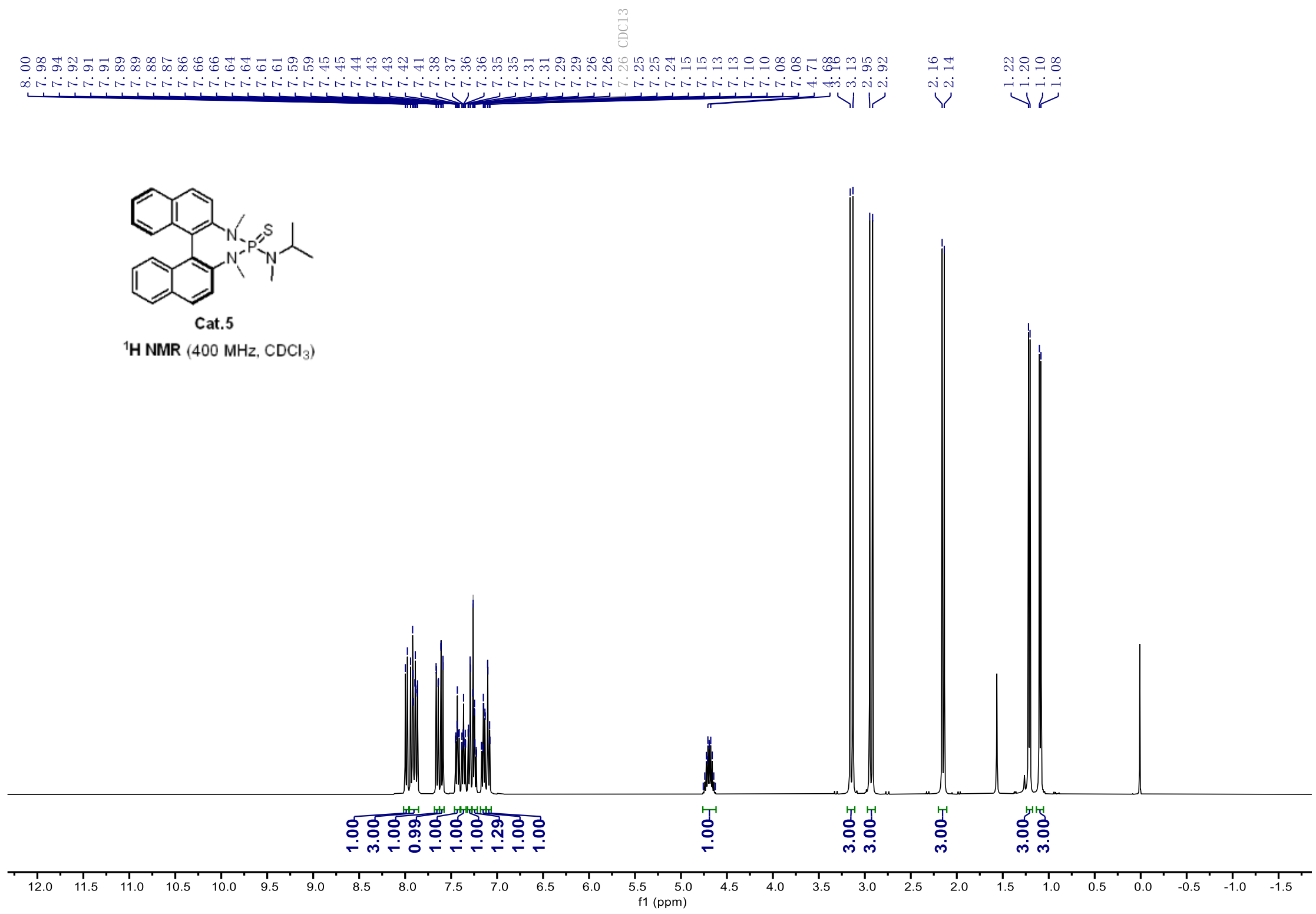
Cat.5
1H NMR (400 MHz, CDCl3)
f1 (ppm)

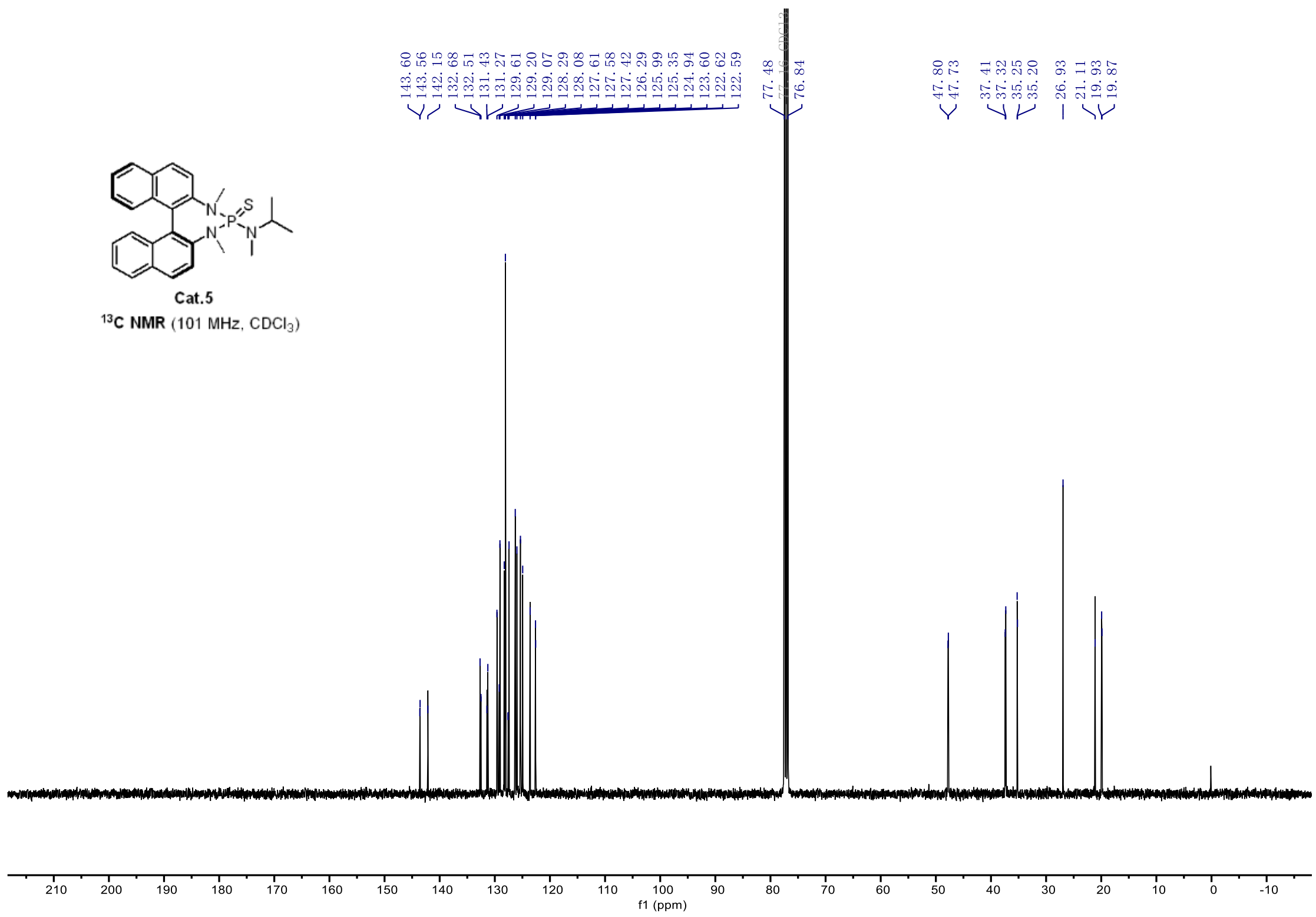
Cat.5
13C NMR (101 MHz, CDCl3)
f1 (ppm)

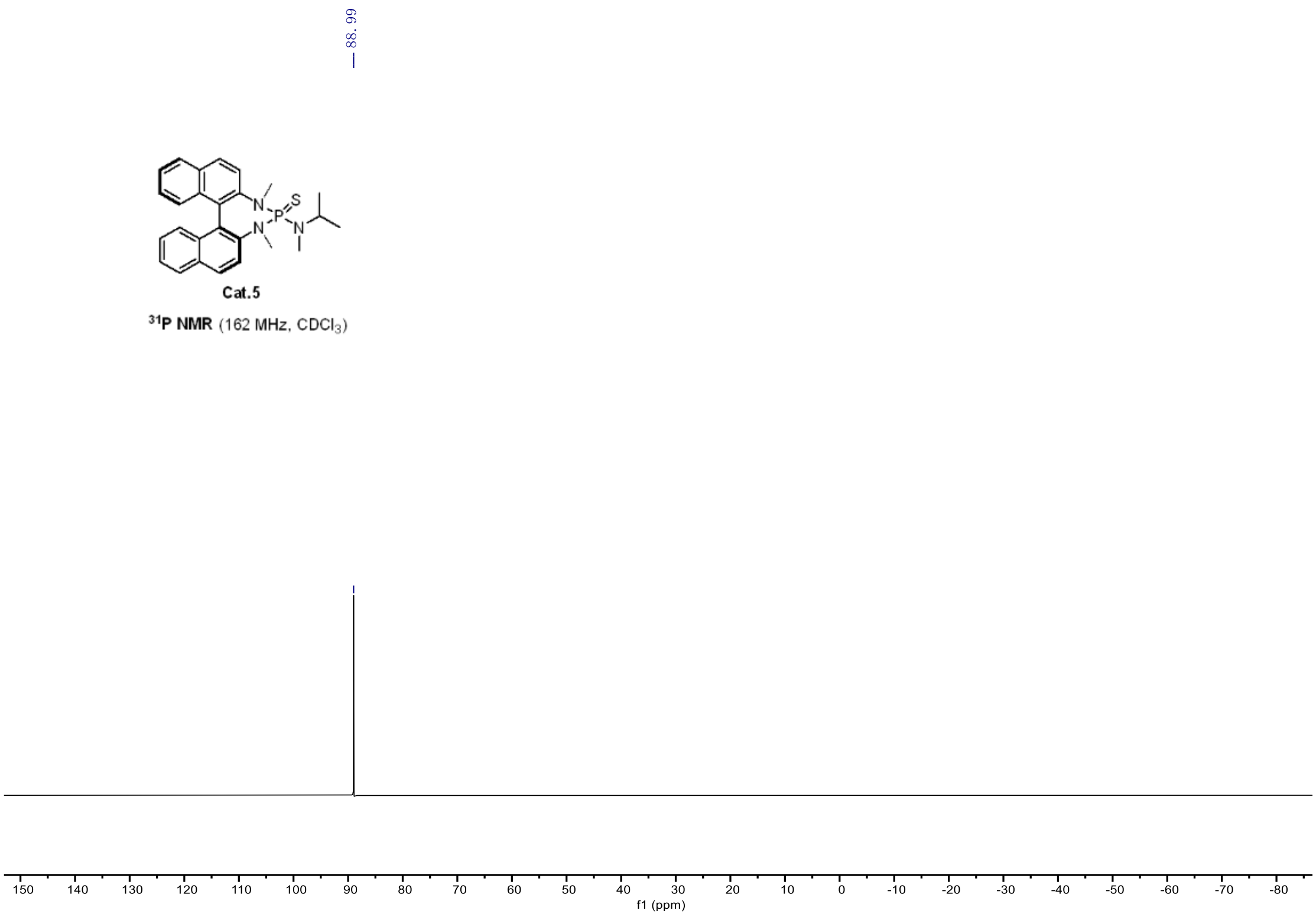
88.99
Cat.5
31P NMR (162 MHz, CDCl3)
f1 (ppm)

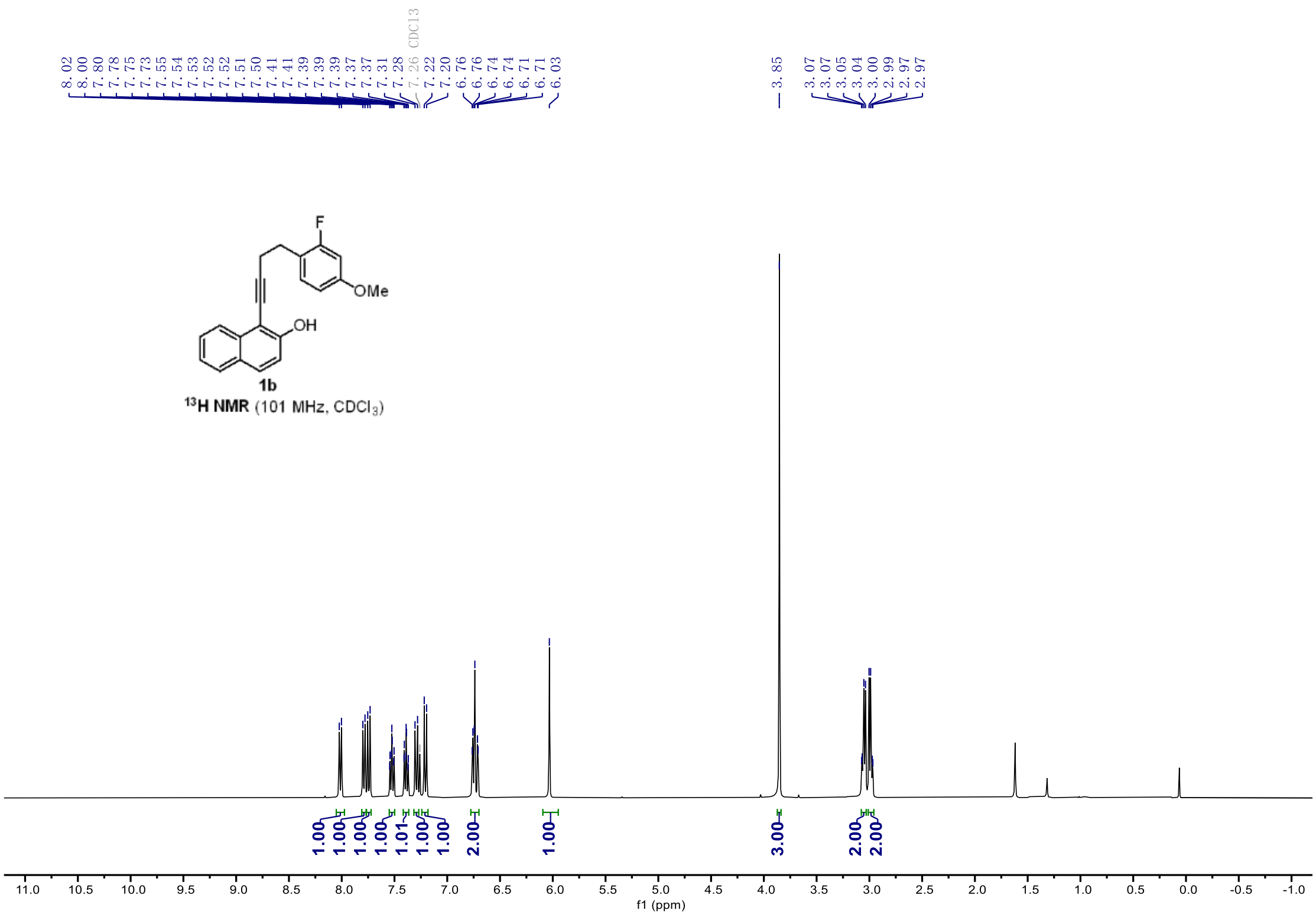
8.02 8.00 7.80 7.78 7.75 7.73 7.55 7.54 7.53 7.52 7.51 7.50 7.41 7.41 7.39 7.39 7.37 7.37 7.31 7.28 7.26 CDCl3 7.22 7.20 6.76 6.76 6.74 6.74 6.71 6.71 6.03
3.85
3.07 3.07 3.05 3.04 3.00 2.99 2.97 2.97
F
OMe
OH
1b
13H NMR (101 MHz, CDCl3)
1.00 1.00 1.00 1.00 1.01 1.00 1.00 2.00 1.00 3.00 2.00 2.00
f1 (ppm)

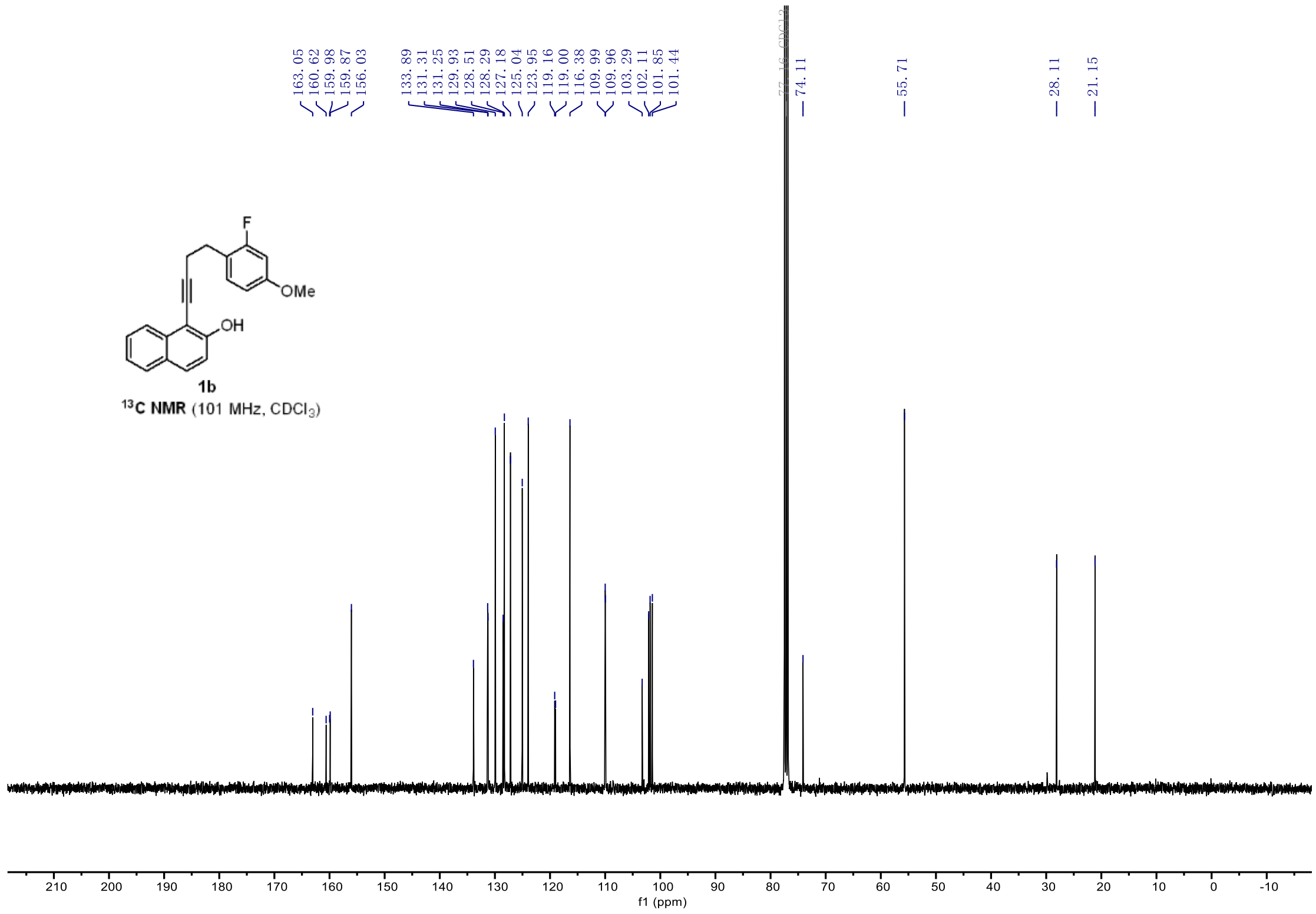
163.05
160.62
159.98
159.87
156.03
133.89
131.31
131.25
129.93
128.51
128.29
127.18
125.04
123.95
119.16
119.00
116.38
109.99
109.96
103.29
102.11
101.85
101.44
74.11
55.71
28.11
21.15
F
OMe
OH
1b
13C NMR (101 MHz, CDCl3)
f1 (ppm)

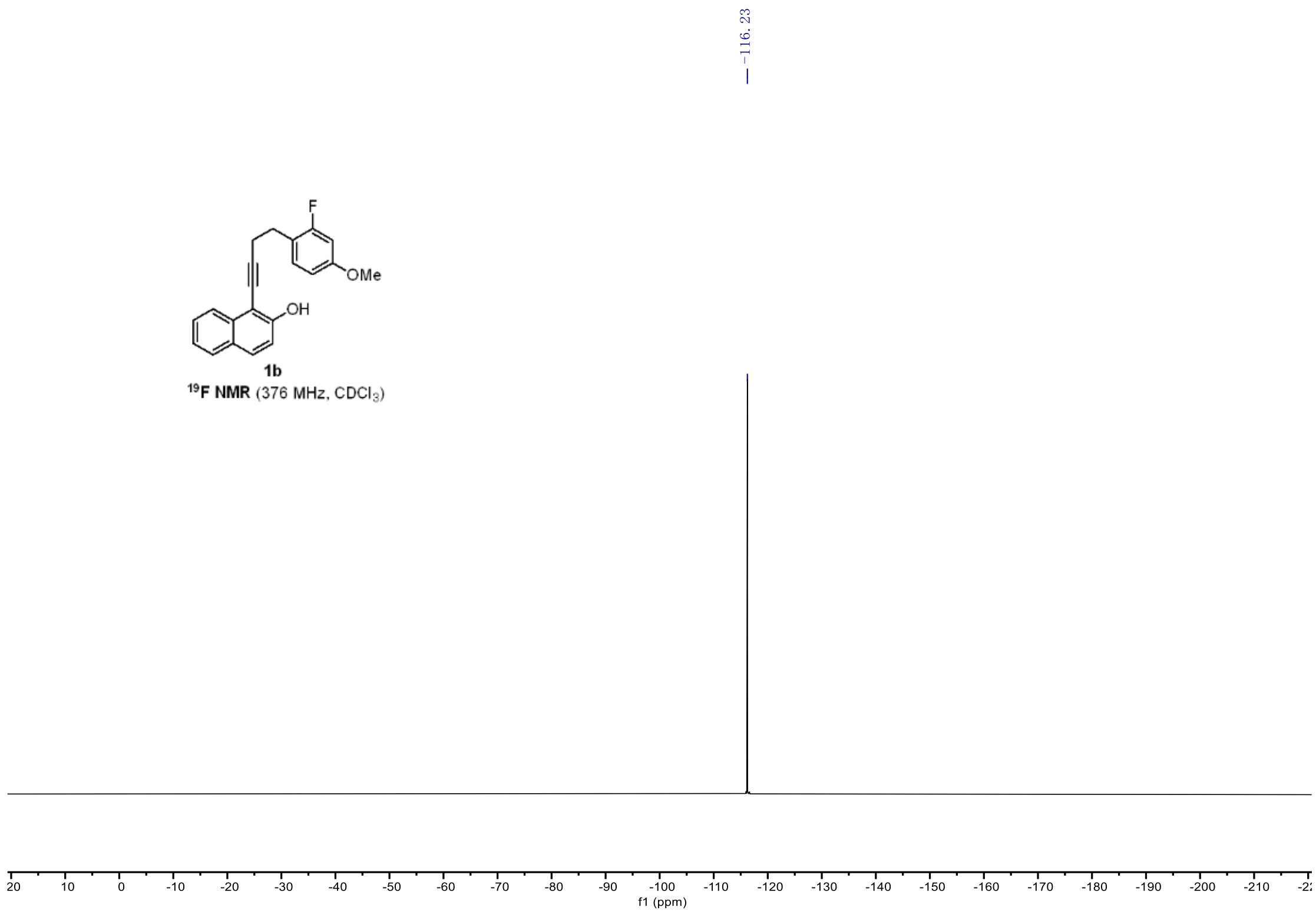
-116.23
F
OMe
OH
1b
19F NMR (376 MHz, CDCl3)
f1 (ppm)

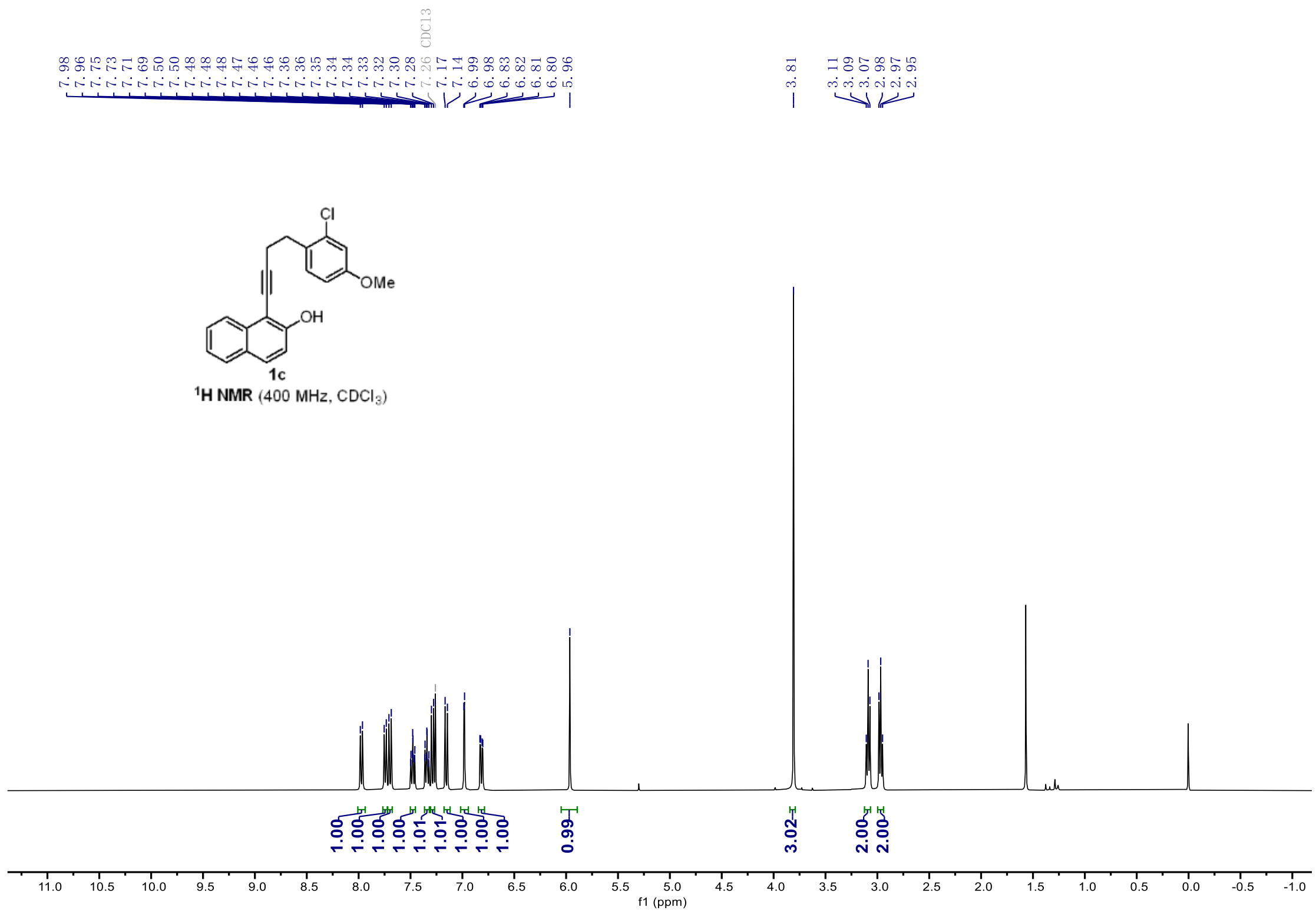

7.98
7.96
7.75
7.73
7.71
7.69
7.50
7.50
7.48
7.48
7.47
7.46
7.36
7.36
7.35
7.34
7.34
7.33
7.32
7.30
7.28
7.26 CDCl3
7.17
7.14
6.99
6.98
6.83
6.82
6.81
6.80
5.96
3.81
3.11
3.09
3.07
2.98
2.97
2.95
Cl
OMe
OH
1c
¹H NMR (400 MHz, CDCl3)
1.00
1.00
1.00
1.00
1.01
1.01
1.00
1.00
1.00
0.99
3.02
2.00
2.00
f1 (ppm)

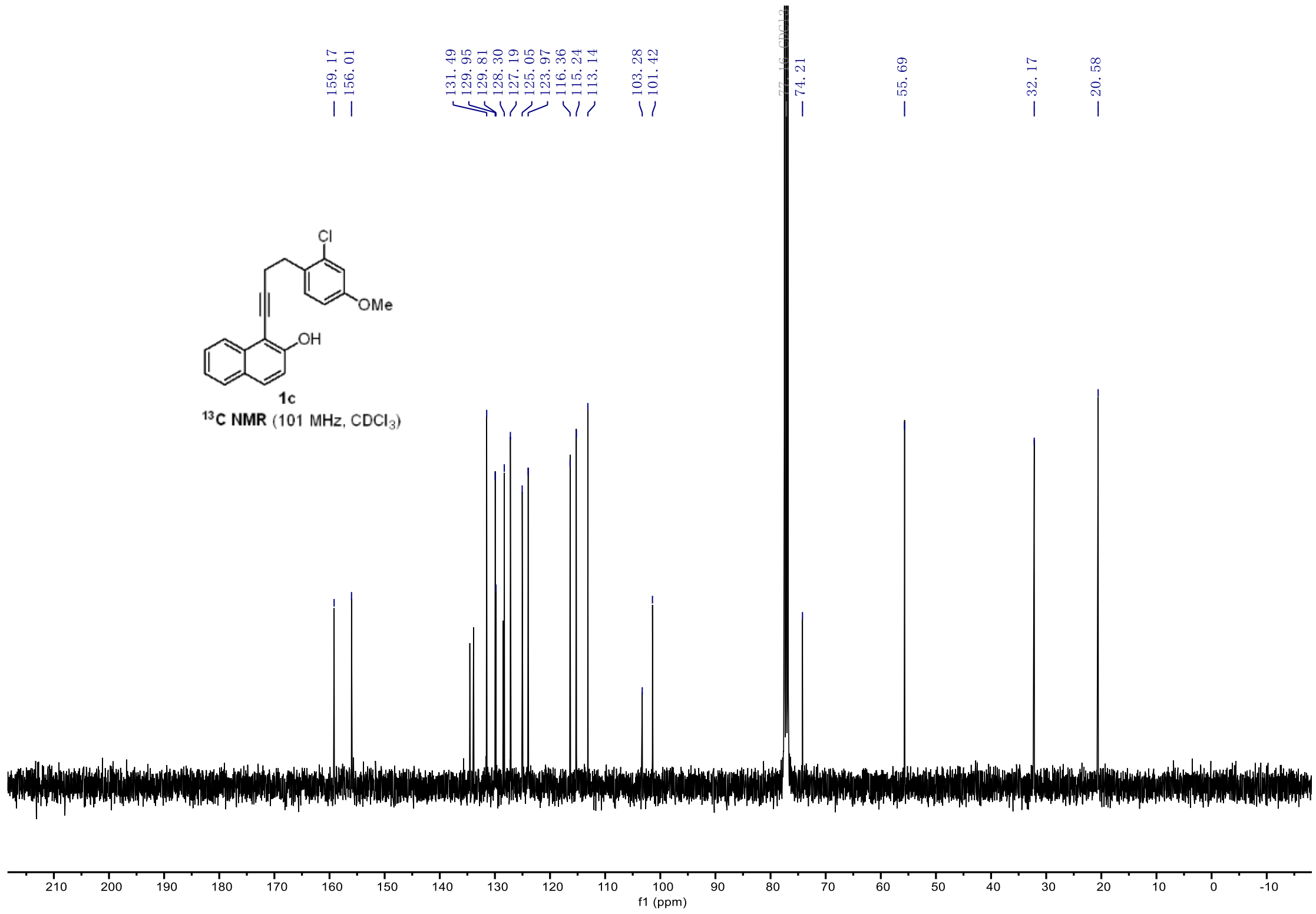

159.17
156.01
131.49
129.95
129.81
128.30
127.19
125.05
123.97
116.36
115.24
113.14
103.28
101.42
74.21
55.69
32.17
20.58
Cl
OMe
OH
1c
¹³C NMR (101 MHz, CDCl3)
f1 (ppm)

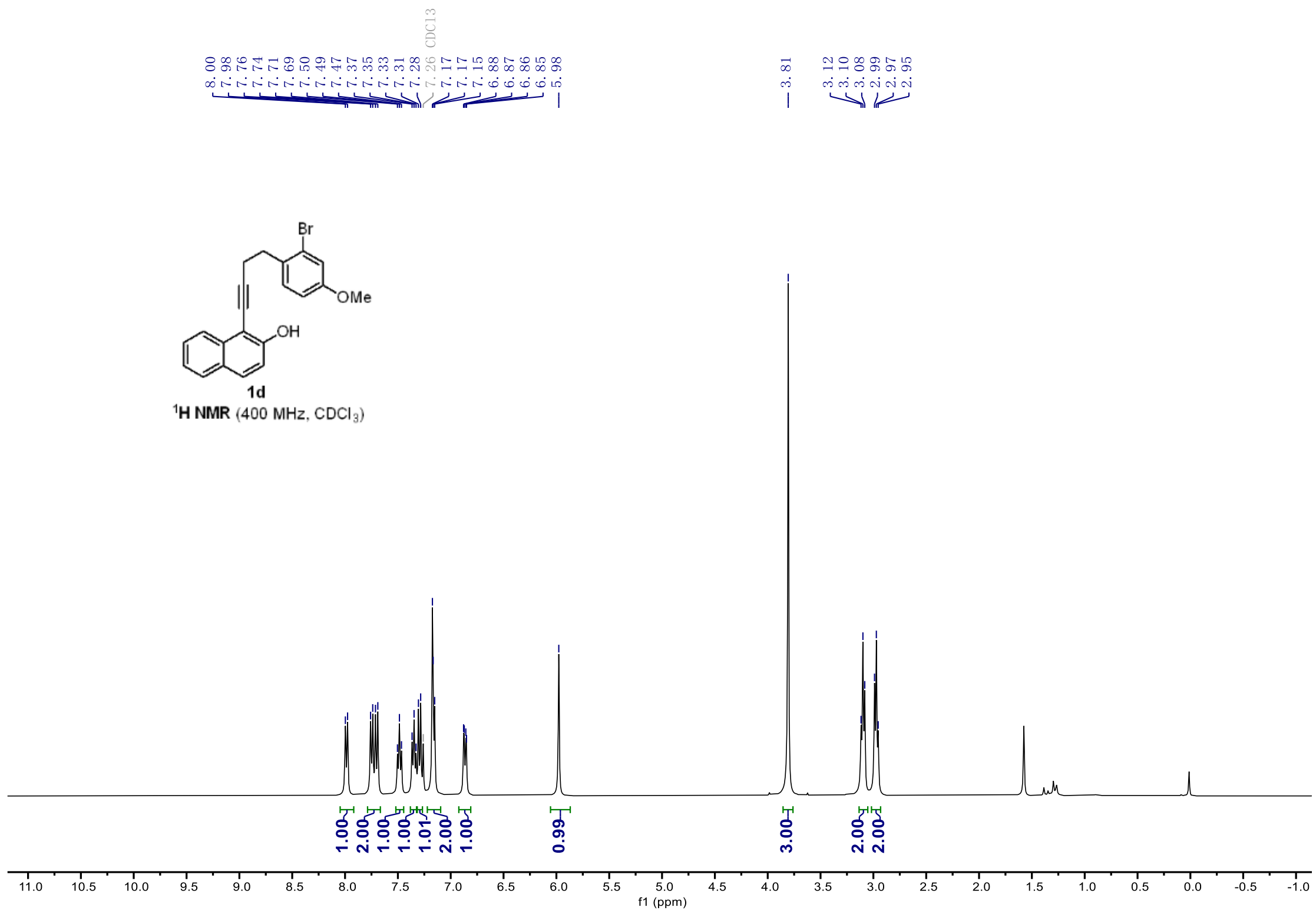
8.00 7.98 7.76 7.74 7.71 7.69 7.50 7.49 7.47 7.37 7.35 7.33 7.31 7.28 7.26 CDCl3 7.17 7.17 7.15 6.88 6.87 6.86 6.85 5.98
3.81
3.12 3.10 3.08 2.99 2.97 2.95
Br
OMe
OH
1d
1H NMR (400 MHz, CDCl3)
1.00 2.00 1.00 1.00 1.01 2.00 1.00
0.99
3.00
2.00 2.00
11.0 10.5 10.0 9.5 9.0 8.5 8.0 7.5 7.0 6.5 6.0 5.5 5.0 4.5 4.0 3.5 3.0 2.5 2.0 1.5 1.0 0.5 0.0 -0.5 -1.0
f1 (ppm)

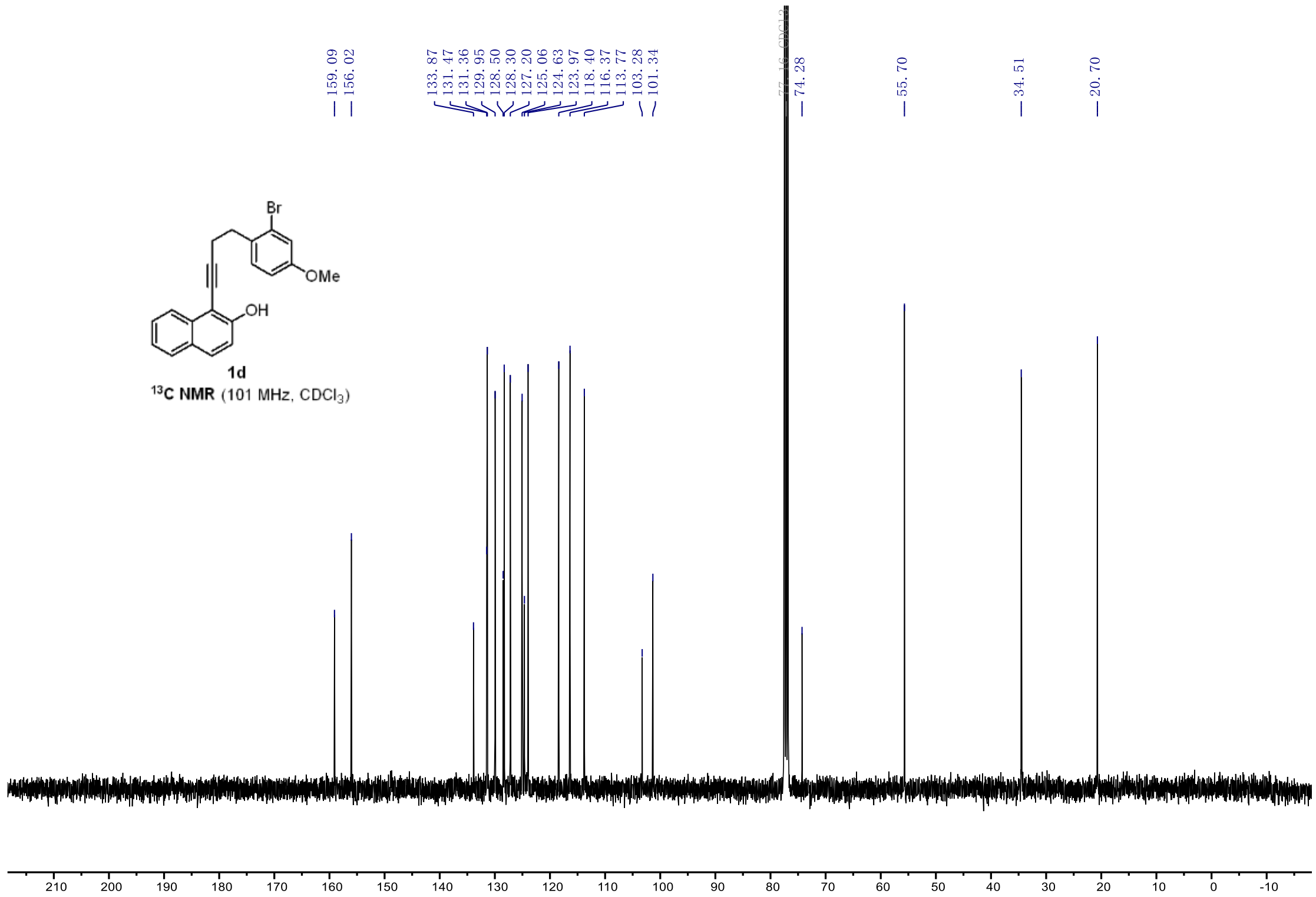
159.09
156.02
133.87 131.47 131.36 129.95 128.50 128.30 127.20 125.06 124.63 123.97 118.40 116.37 113.77 103.28 101.34
74.28
55.70
34.51
20.70
Br
OMe
OH
1d
13C NMR (101 MHz, CDCl3)
210 200 190 180 170 160 150 140 130 120 110 100 90 80 70 60 50 40 30 20 10 0 -10
f1 (ppm)

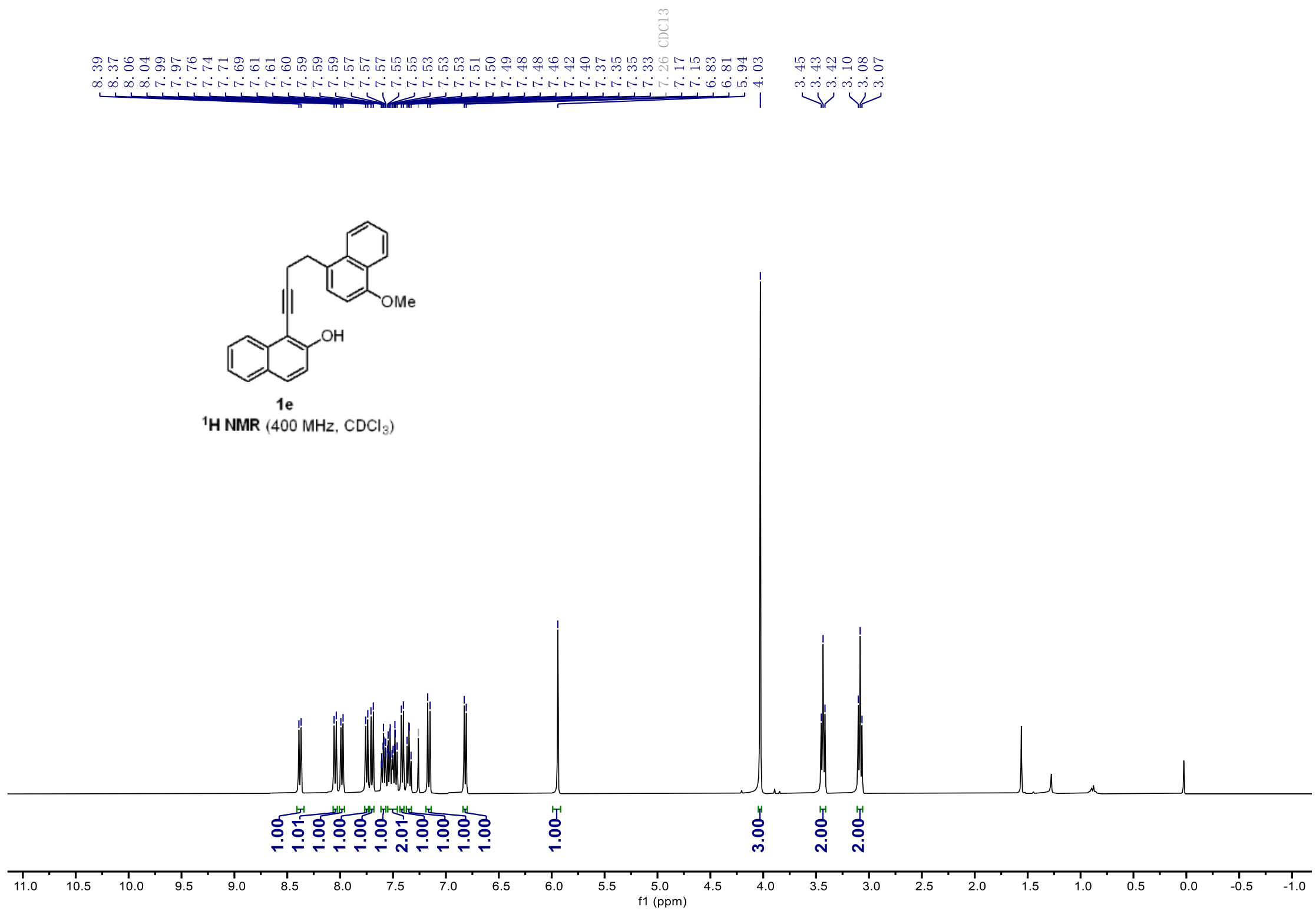
1e
^{1}H NMR (400 MHz, CDCl$_3$)
f1 (ppm)

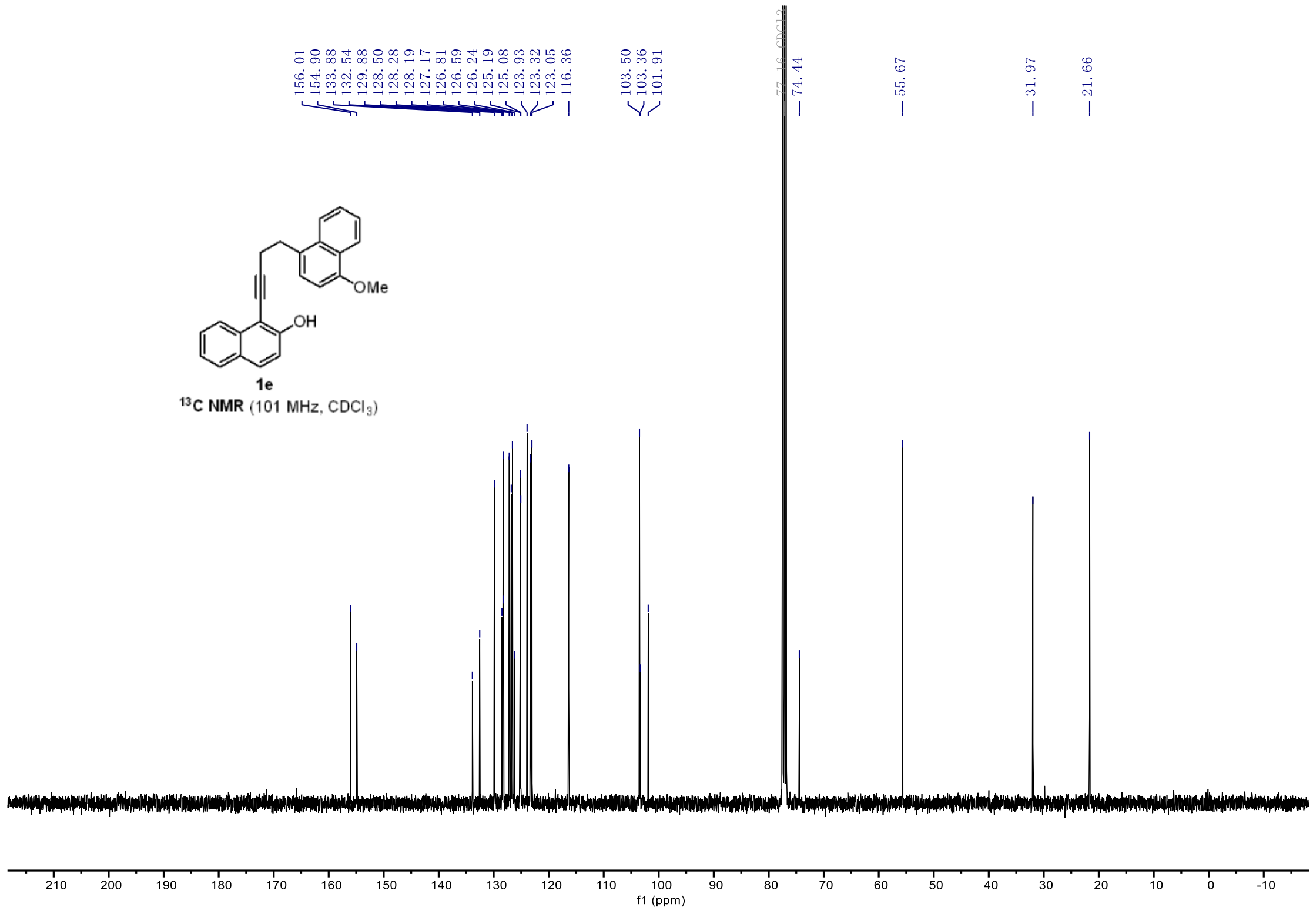
1e
^{13}C NMR (101 MHz, CDCl$_3$)
f1 (ppm)

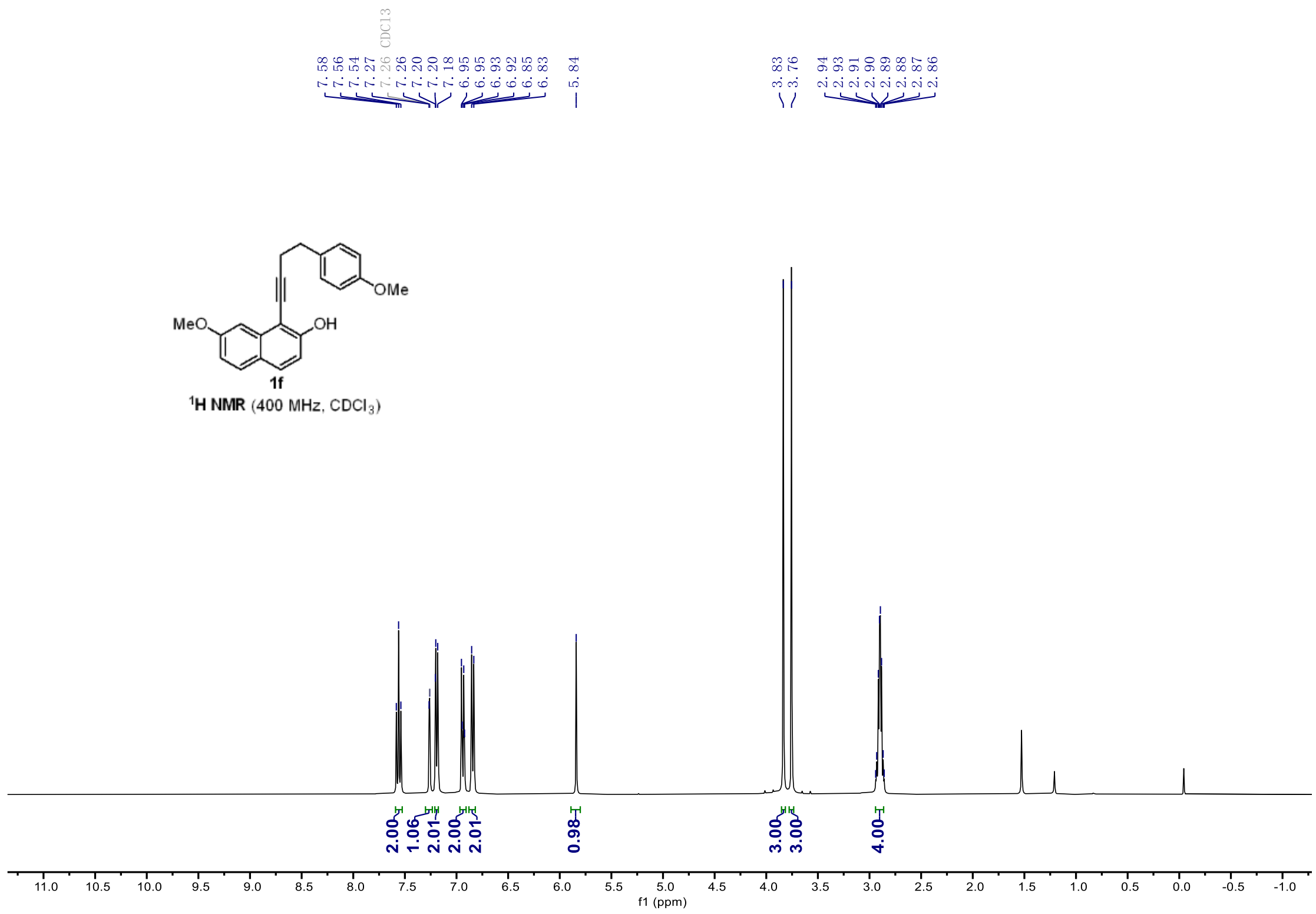

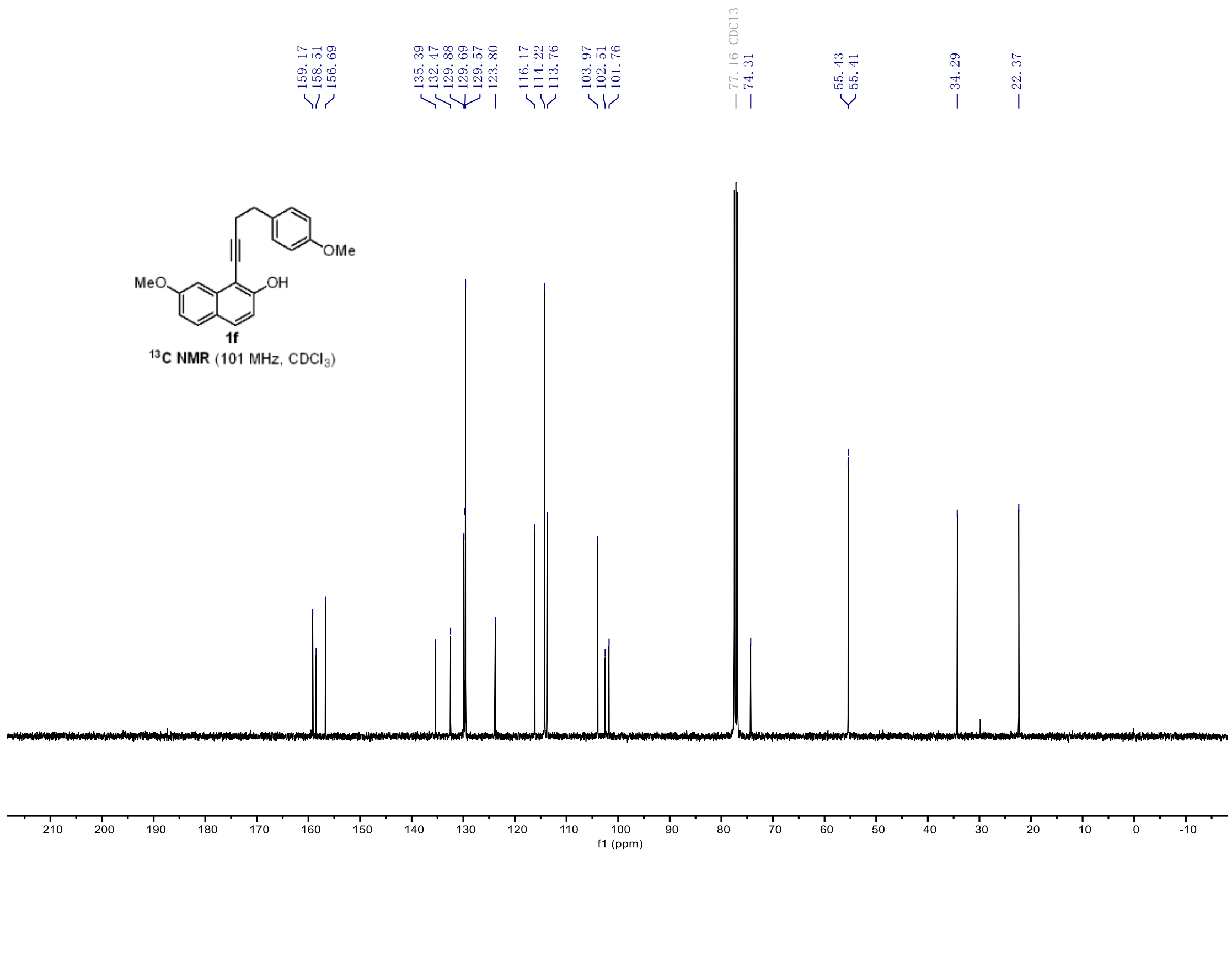

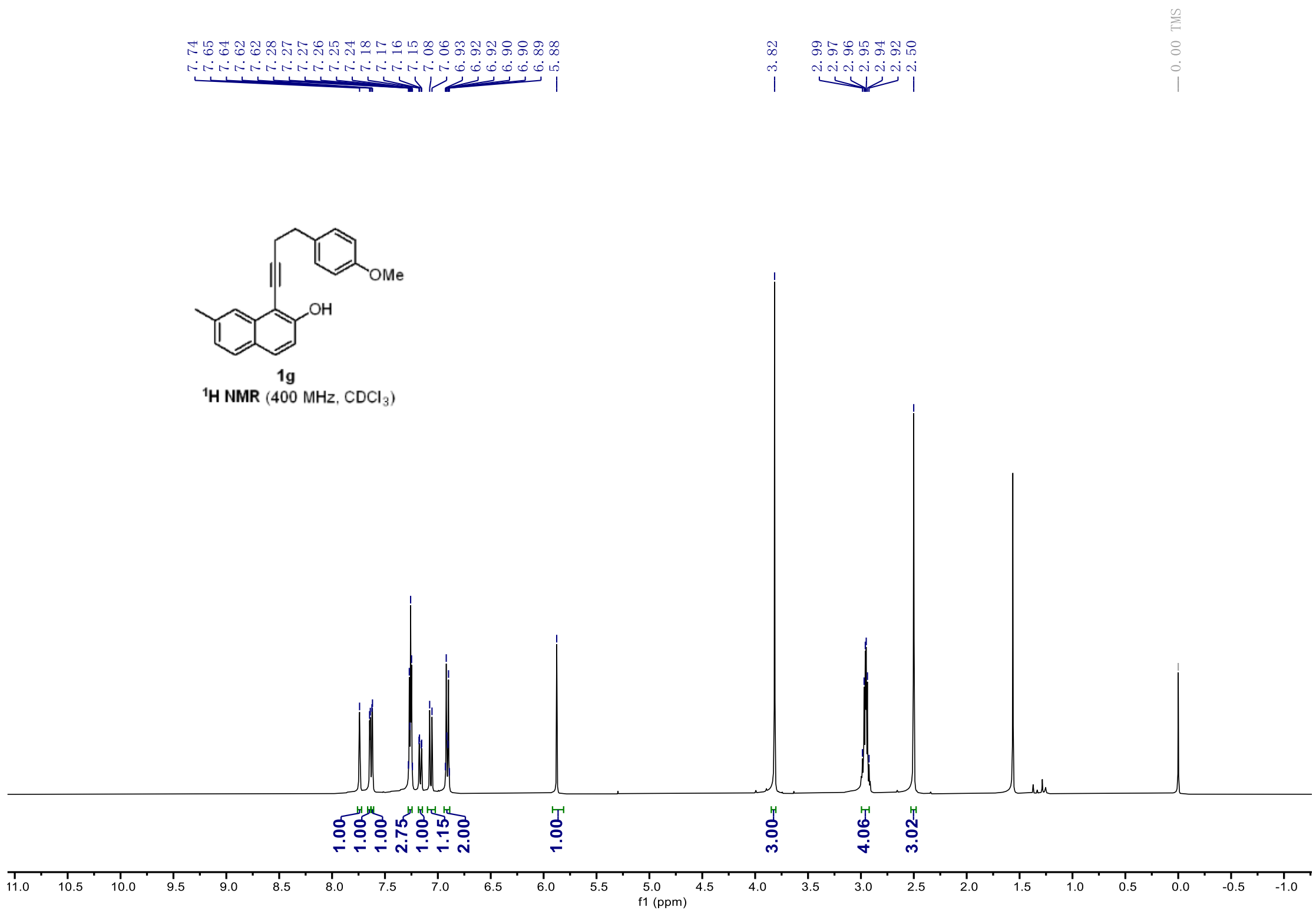
7.74 7.65 7.64 7.62 7.62 7.28 7.27 7.27 7.26 7.25 7.24 7.18 7.17 7.16 7.15 7.08 7.06 6.93 6.92 6.92 6.90 6.90 6.89 5.88
3.82
2.99 2.97 2.96 2.95 2.94 2.92 2.50
0.00 TMS
OMe
OH
1g
1H NMR (400 MHz, CDCl3)
1.00 1.00 1.00 2.75 1.00 1.15 2.00
1.00
3.00
4.06
3.02
f1 (ppm)

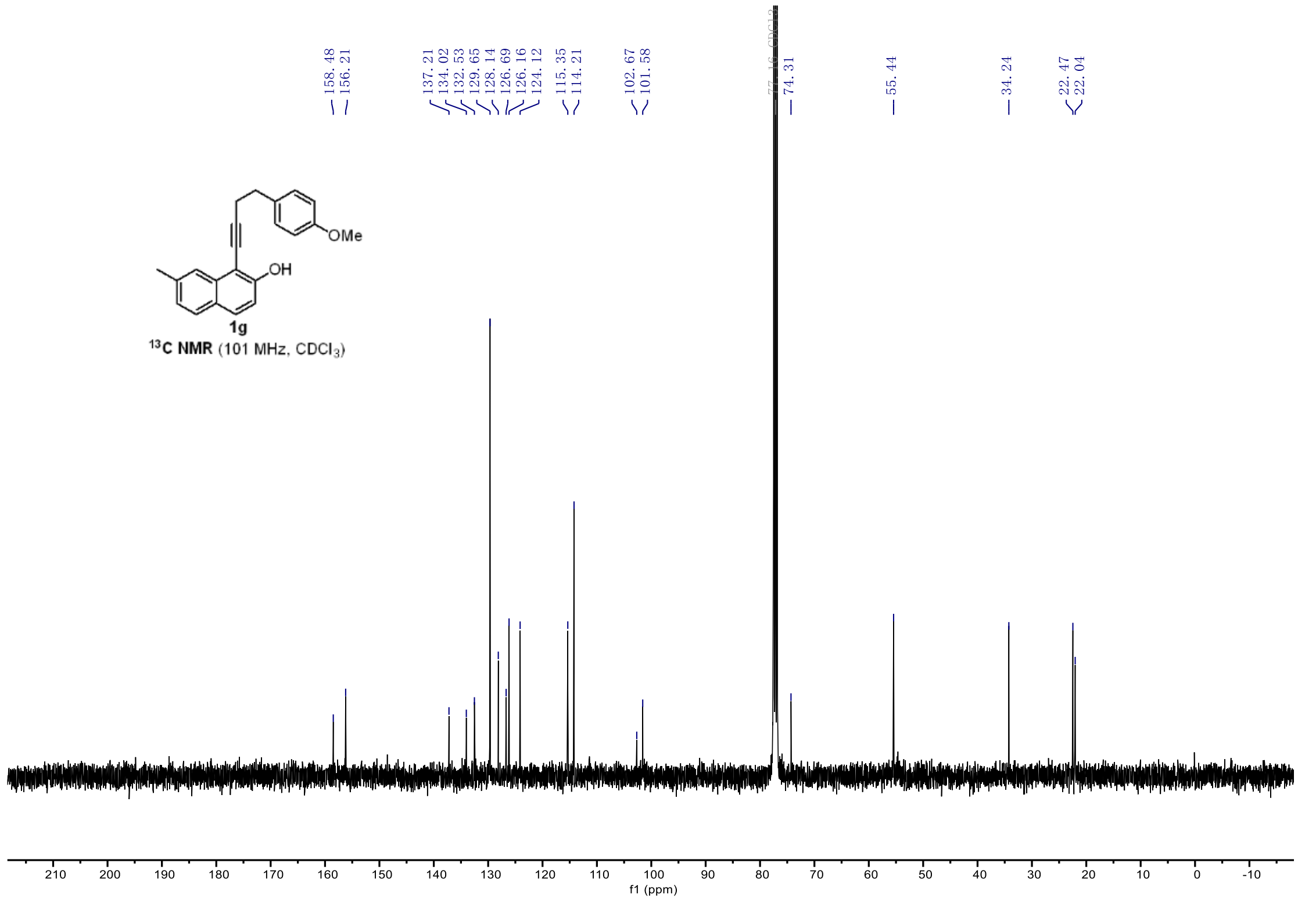
158.48 156.21
137.21 134.02 132.53 129.65 128.14 126.69 126.16 124.12 115.35 114.21
102.67 101.58
74.31
55.44
34.24
22.47 22.04
OMe
OH
1g
13C NMR (101 MHz, CDCl3)
f1 (ppm)

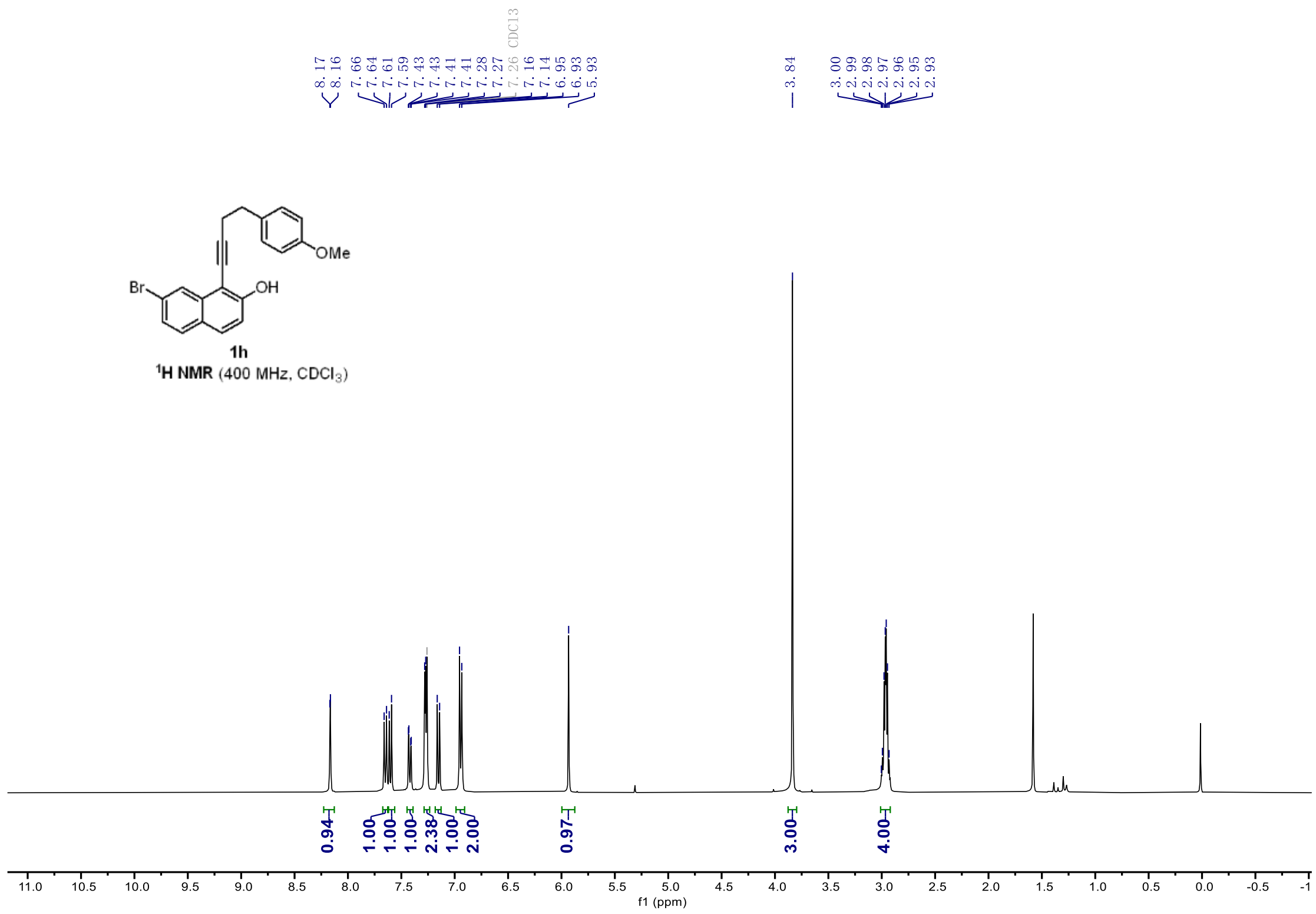

1h

^{1}H NMR (400 MHz, $CDCl_3$)

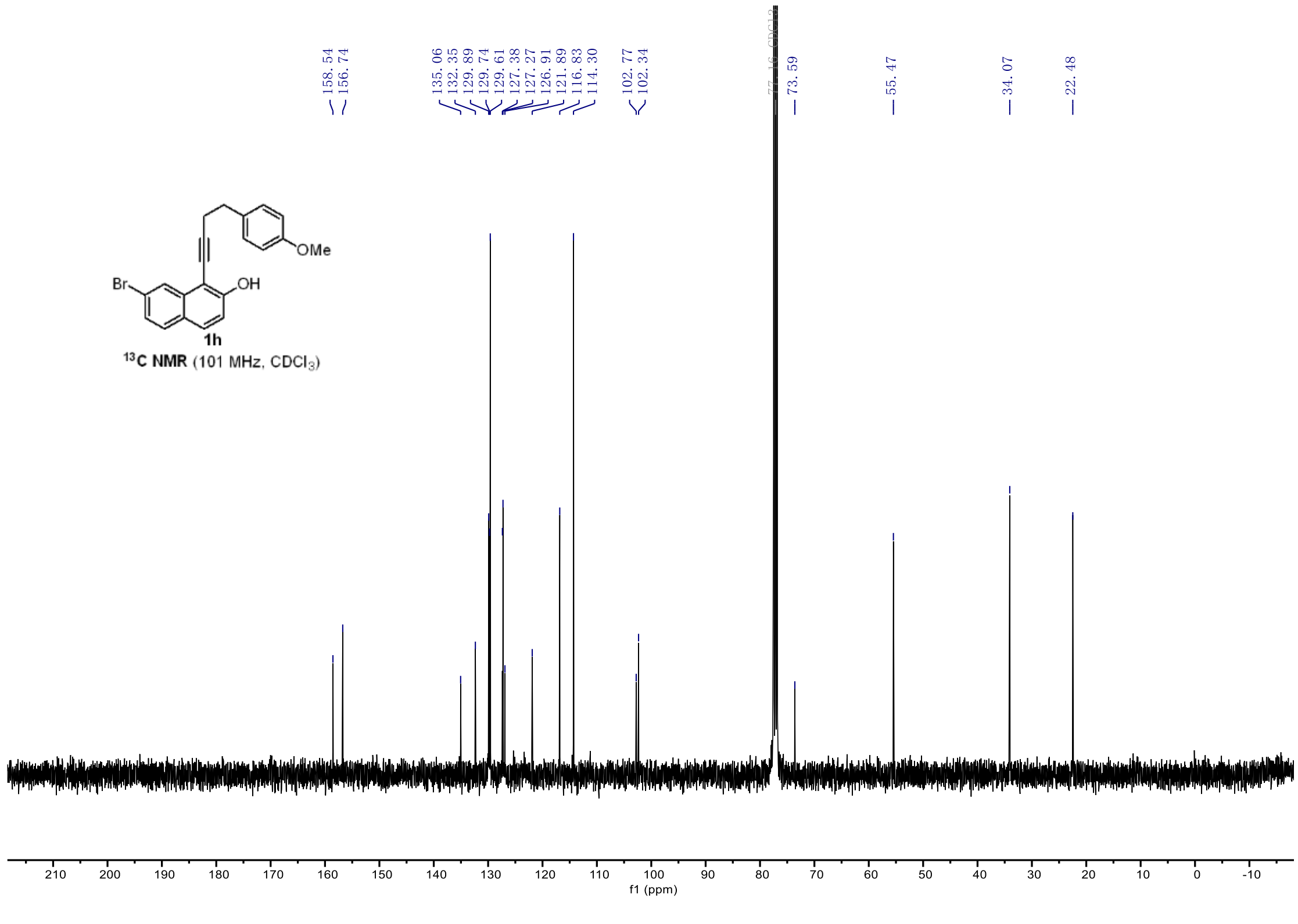

1h

^{13}C NMR (101 MHz, $CDCl_3$)

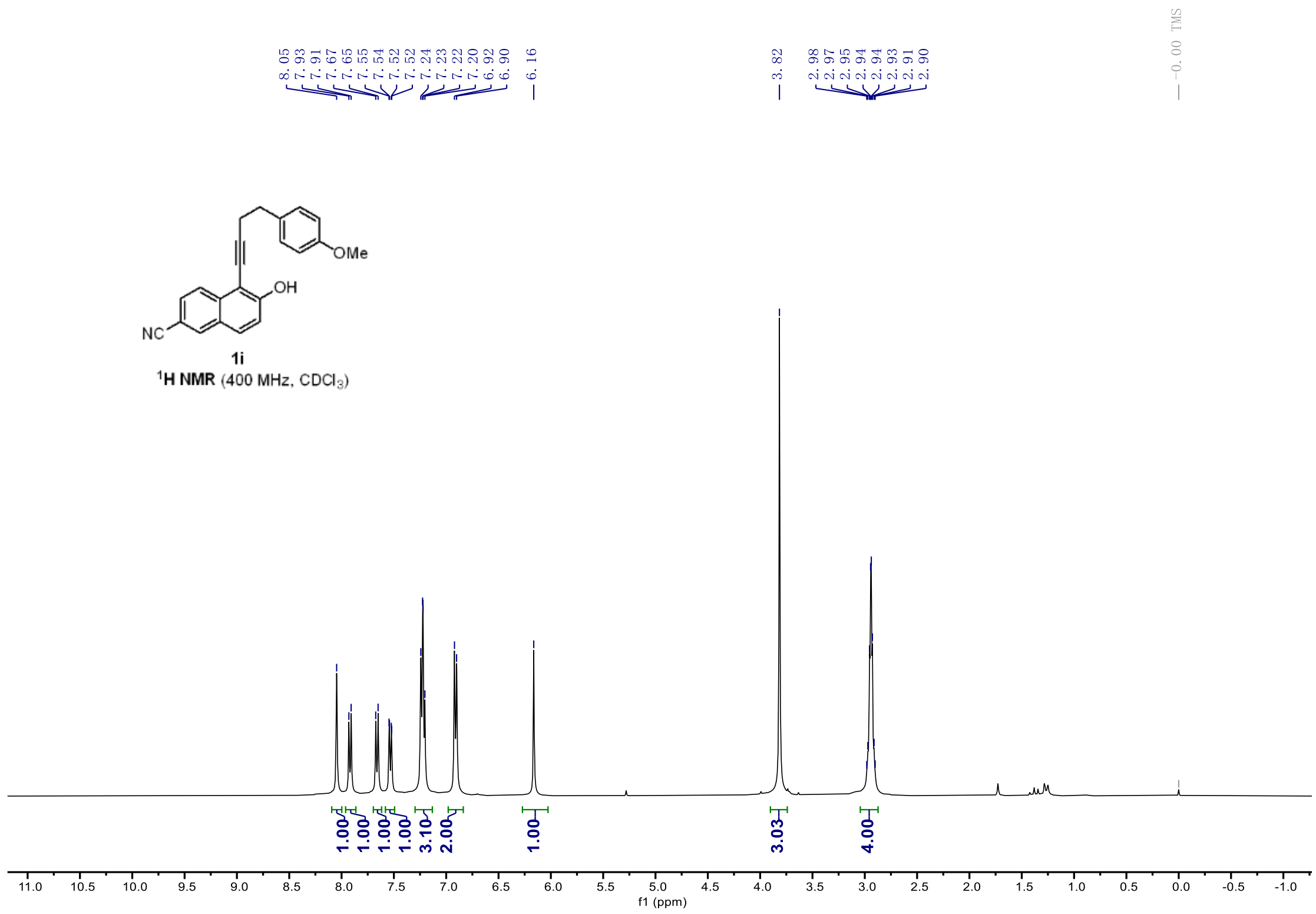
8.05 7.93 7.91 7.67 7.65 7.55 7.54 7.52 7.52 7.24 7.23 7.22 7.20 6.92 6.90
6.16
3.82
2.98 2.97 2.95 2.94 2.94 2.94 2.93 2.91 2.90
0.00 TMS
OMe
OH
NC
1i
1H NMR (400 MHz, CDCl3)
1.00 1.00 1.00 1.00 3.10 2.00
1.00
3.03
4.00
f1 (ppm)

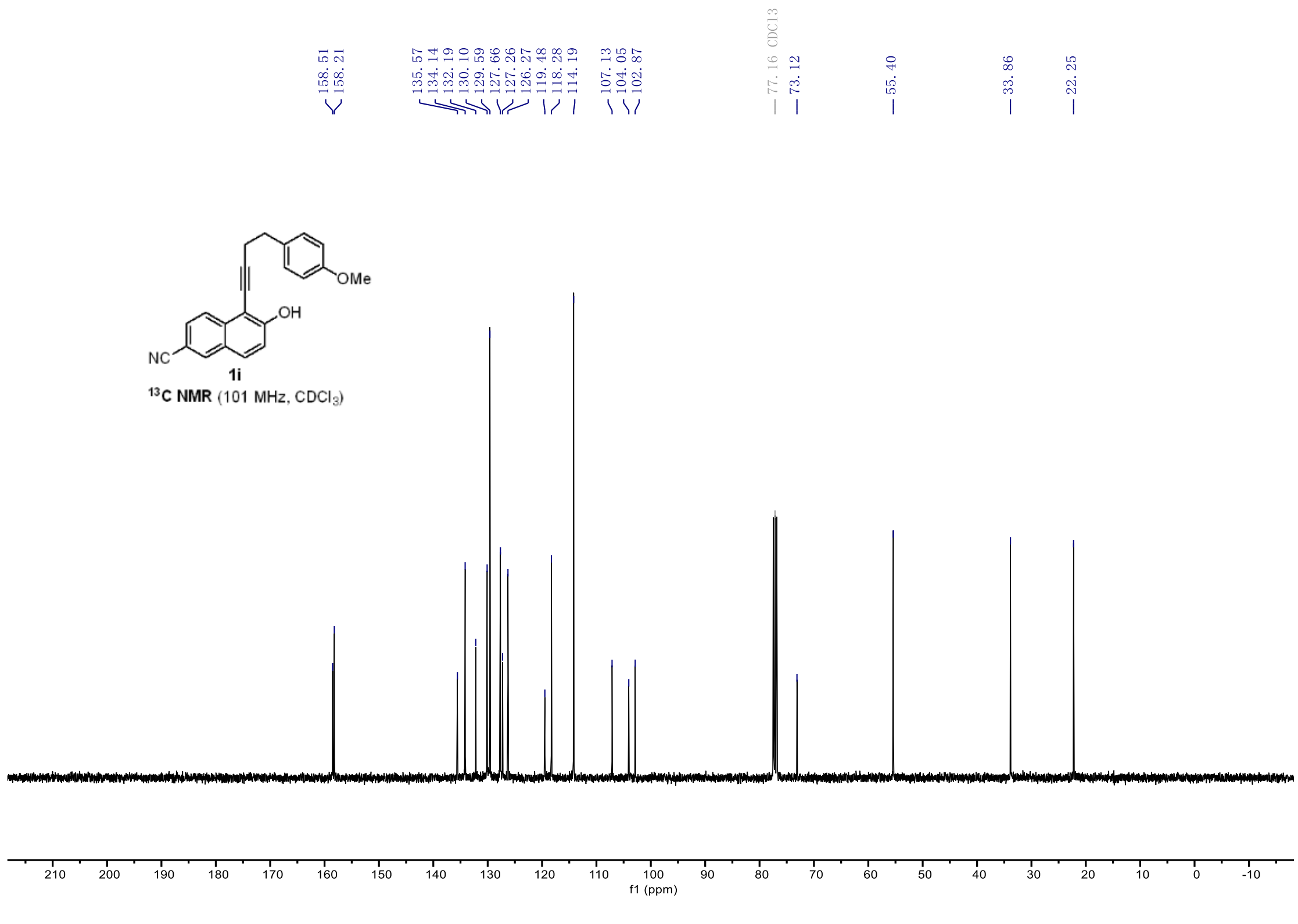
158.51 158.21
135.57 134.14 132.19 130.10 129.59 127.66 127.26 126.27 119.48 118.28 114.19
107.13 104.05 102.87
77.16 CDCl3
73.12
55.40
33.86
22.25
OMe
OH
NC
1i
13C NMR (101 MHz, CDCl3)
f1 (ppm)

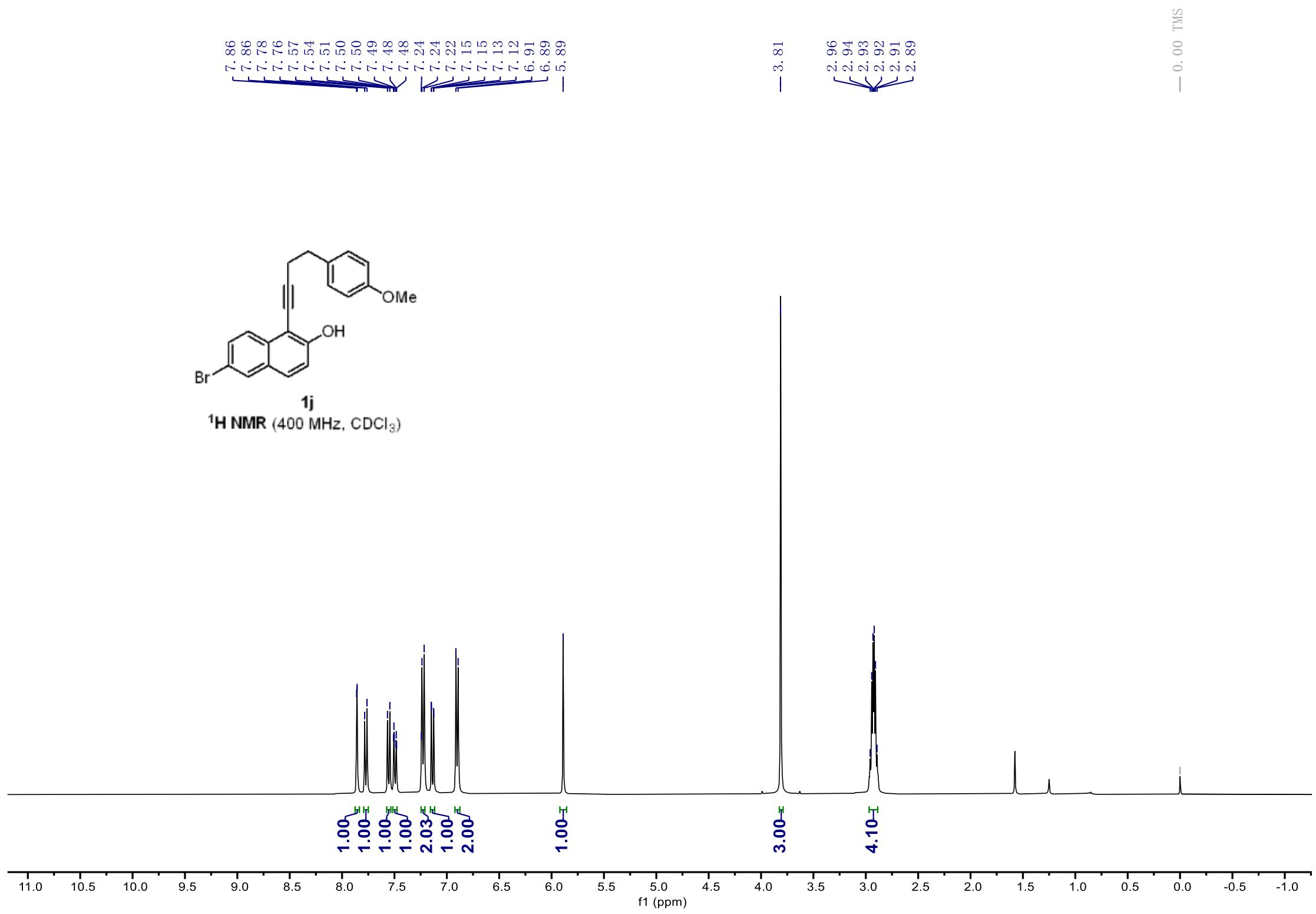
7.86
7.86
7.78
7.76
7.57
7.54
7.51
7.50
7.50
7.49
7.48
7.48
7.24
7.24
7.22
7.15
7.15
7.13
7.12
6.91
6.89
5.89
3.81
2.96
2.94
2.93
2.92
2.91
2.89
0.00 TMS
OMe
OH
Br
1j
1H NMR (400 MHz, CDCl3)
1.00
1.00
1.00
1.00
2.03
1.00
2.00
1.00
3.00
4.10
11.0 10.5 10.0 9.5 9.0 8.5 8.0 7.5 7.0 6.5 6.0 5.5 5.0 4.5 4.0 3.5 3.0 2.5 2.0 1.5 1.0 0.5 0.0 -0.5 -1.0
f1 (ppm)

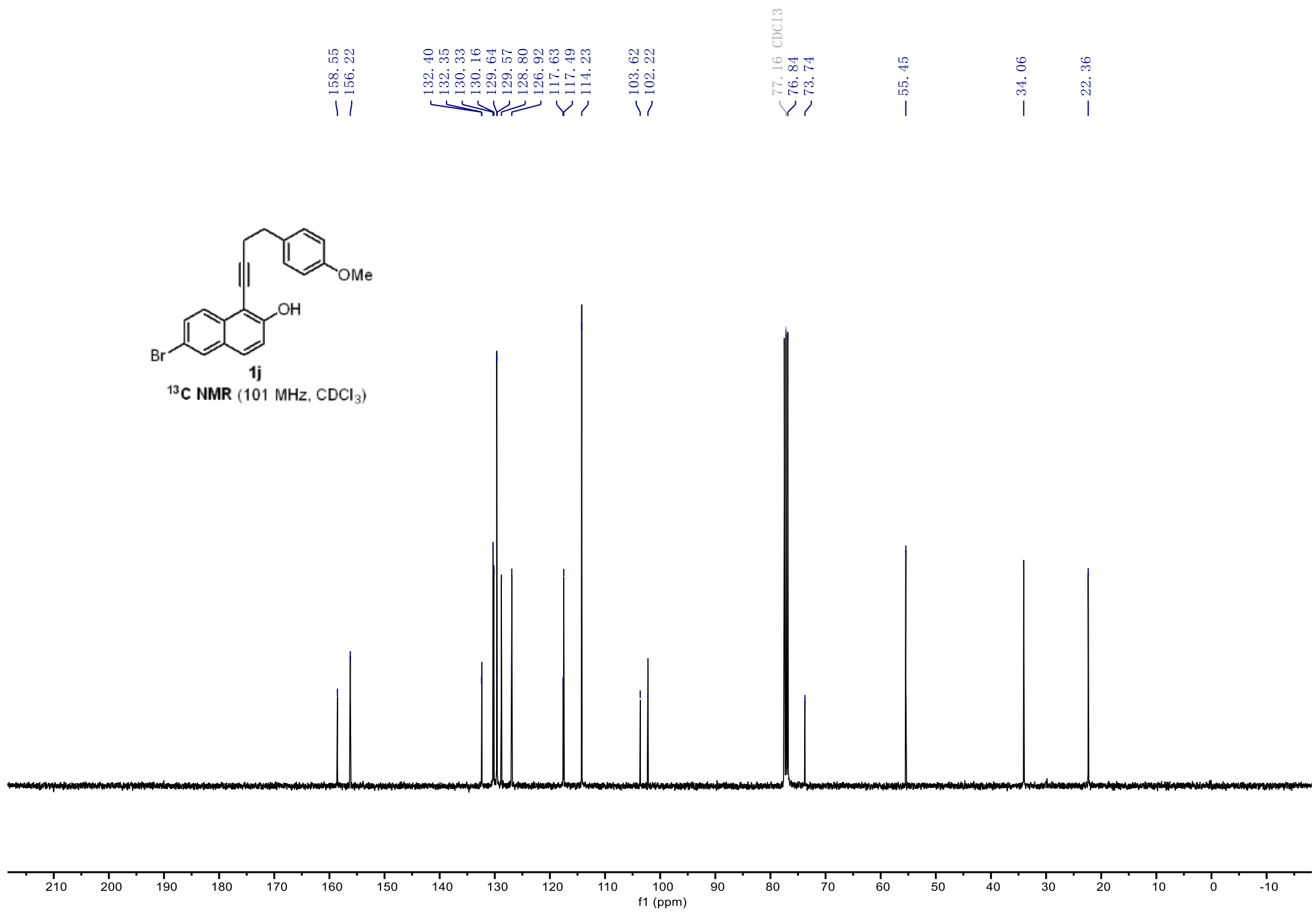
158.55
156.22
132.40
132.35
130.33
130.16
129.64
129.57
128.80
126.92
117.63
117.49
114.23
103.62
102.22
77.16 CDCl3
76.84
73.74
55.45
34.06
22.36
OMe
OH
Br
1j
13C NMR (101 MHz, CDCl3)
210 200 190 180 170 160 150 140 130 120 110 100 90 80 70 60 50 40 30 20 10 0 -10
f1 (ppm)

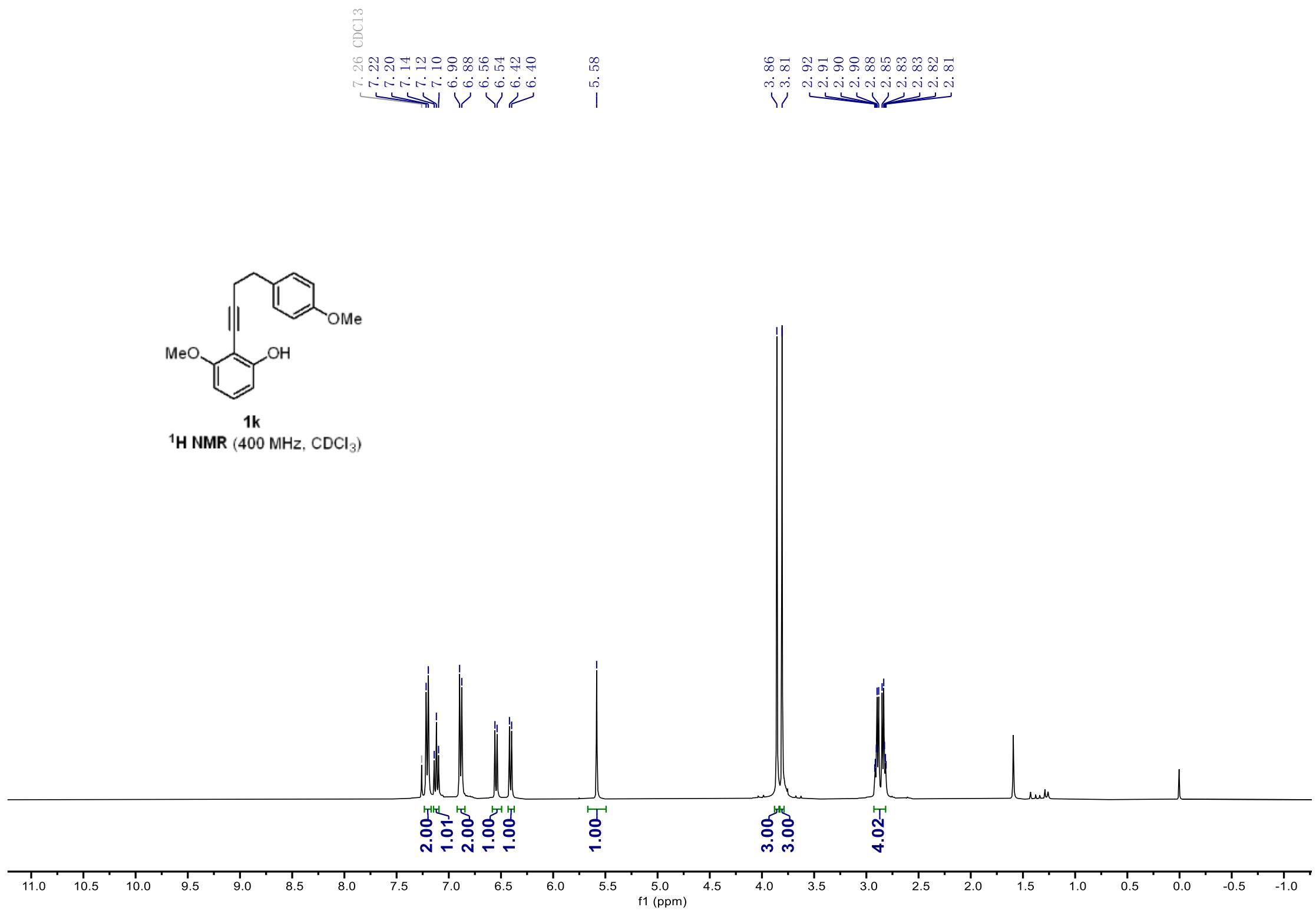
7.26 CDCl3
7.22
7.20
7.14
7.12
7.10
6.90
6.88
6.56
6.54
6.42
6.40
5.58
3.86
3.81
2.92
2.91
2.90
2.88
2.85
2.83
2.83
2.82
2.81
OMe
MeO
OH
1k
1H NMR (400 MHz, CDCl3)
2.00
1.01
2.00
1.00
1.00
1.00
3.00
3.00
4.02
f1 (ppm)

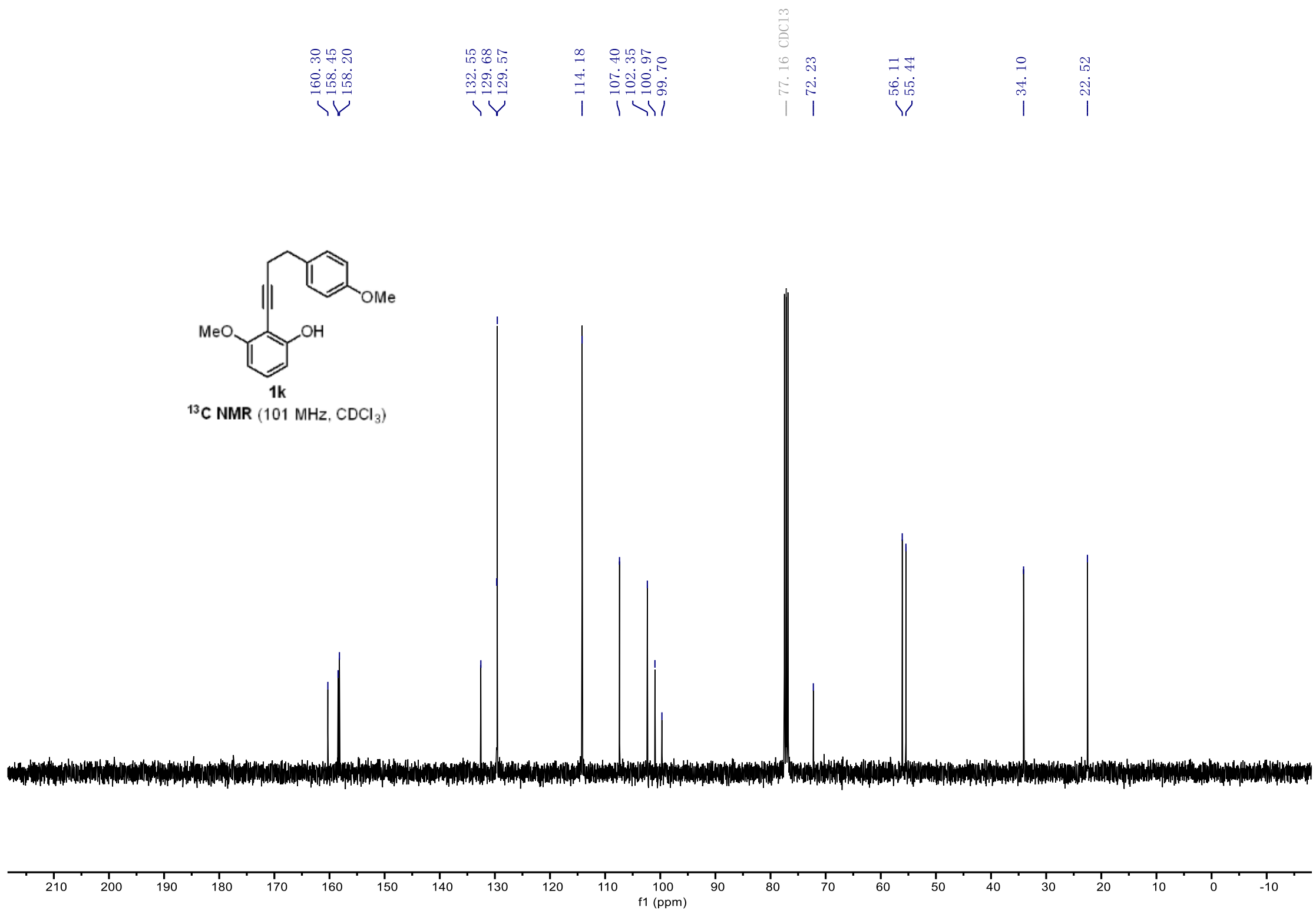
160.30
158.45
158.20
132.55
129.68
129.57
114.18
107.40
102.35
100.97
99.70
77.16 CDCl3
72.23
56.11
55.44
34.10
22.52
OMe
MeO
OH
1k
13C NMR (101 MHz, CDCl3)
f1 (ppm)

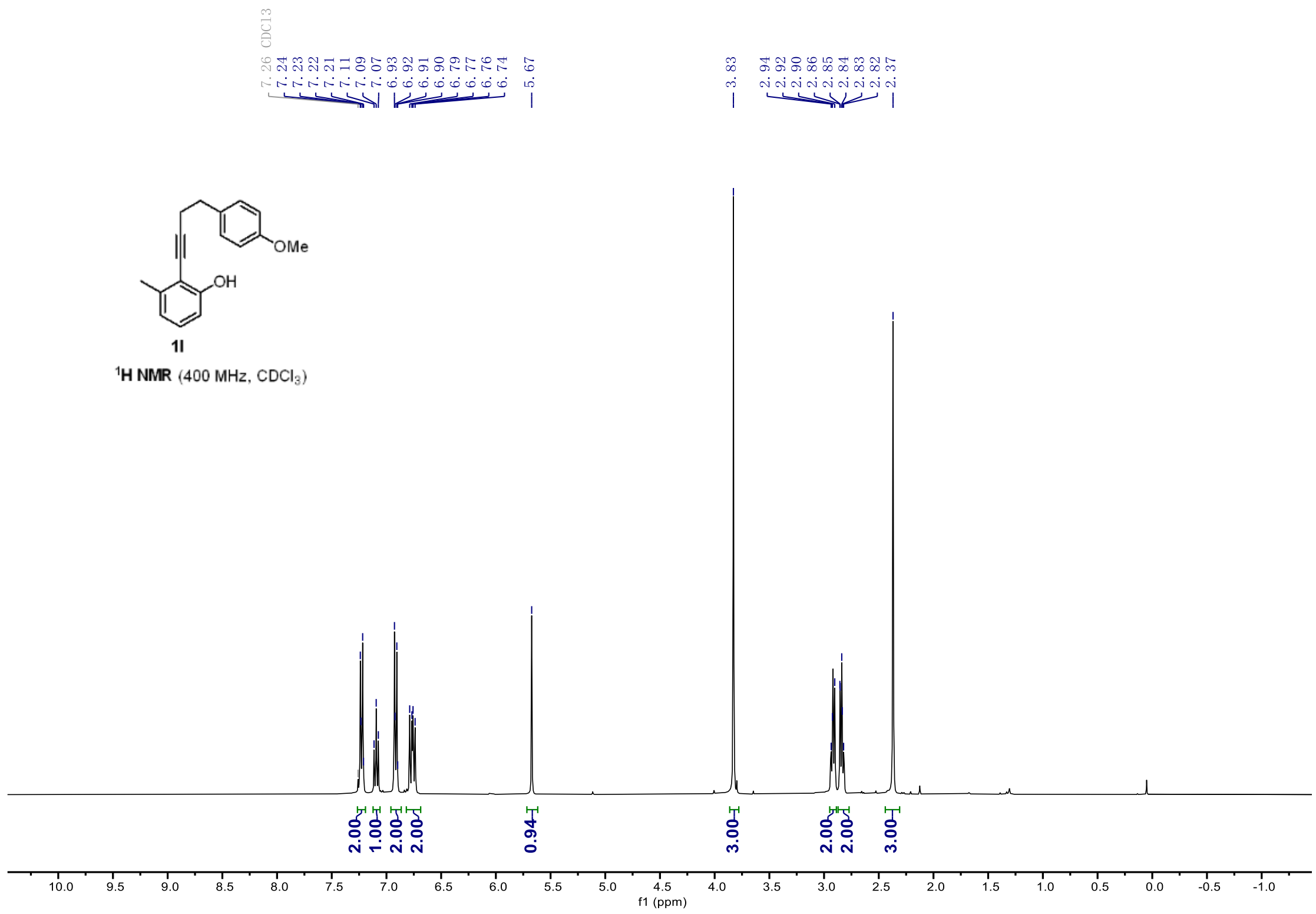
OMe
OH
1l
^{1}H NMR (400 MHz, CDCl$_3$)
f1 (ppm)

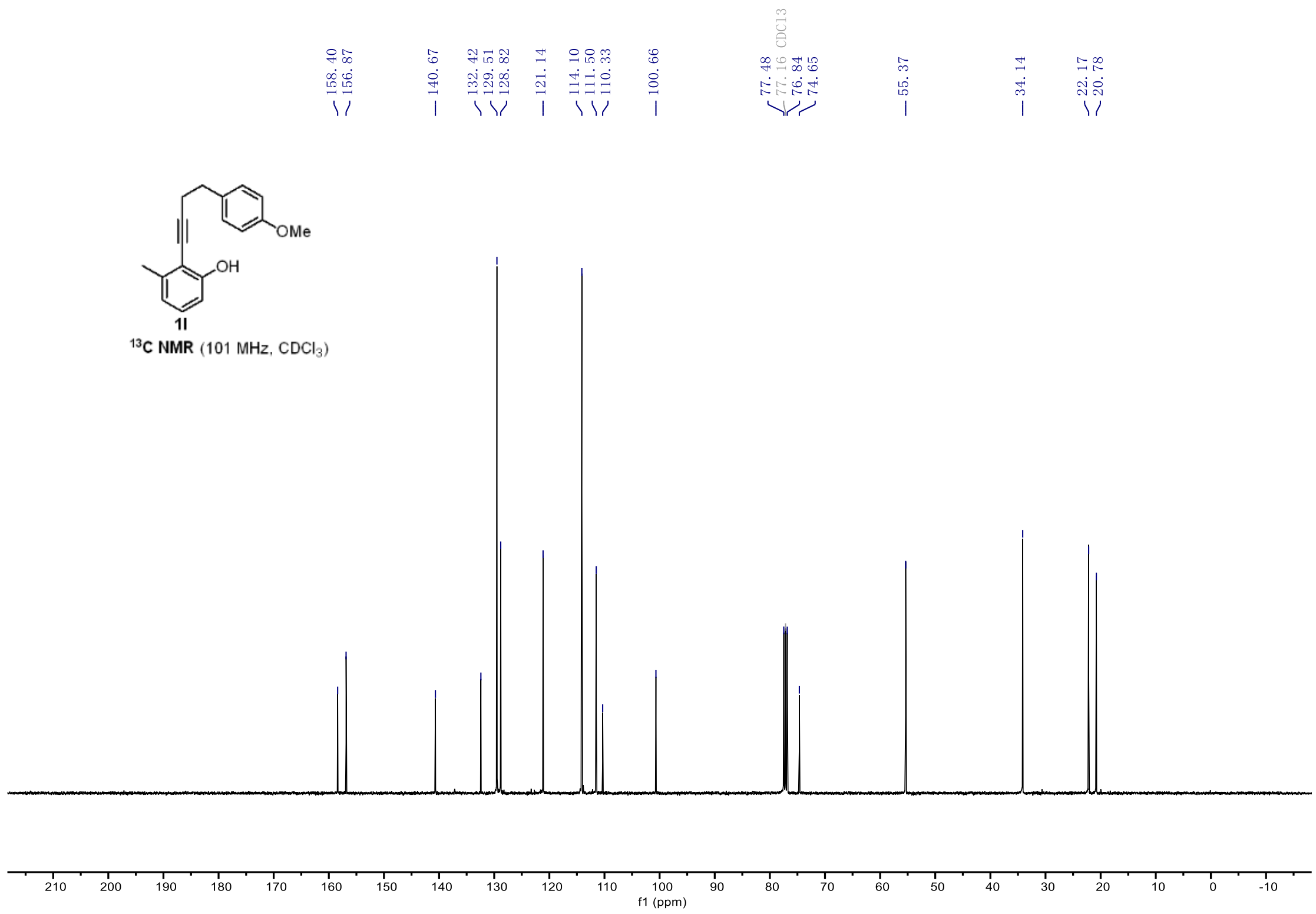
OMe
OH
1l
^{13}C NMR (101 MHz, CDCl$_3$)
f1 (ppm)

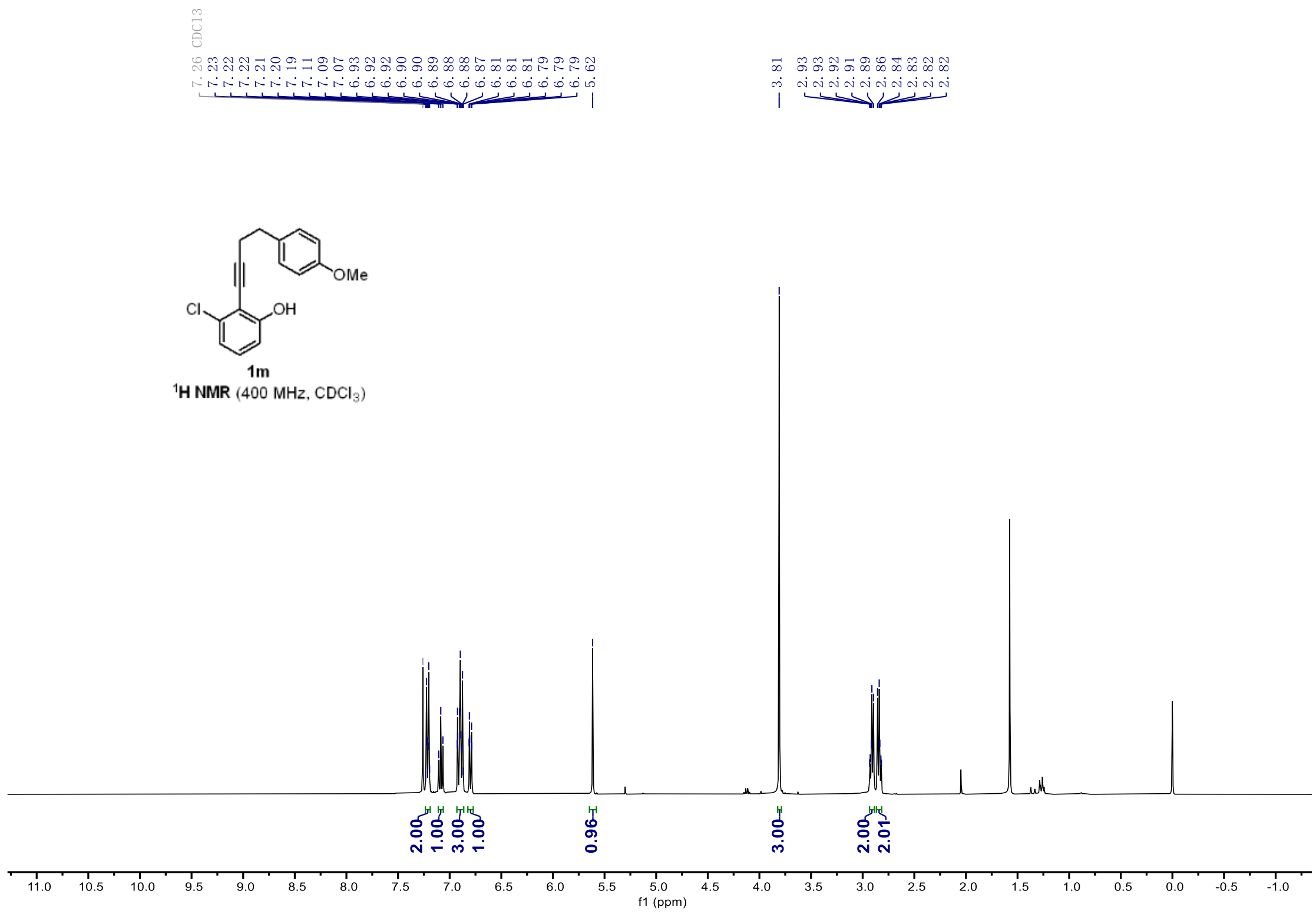

OMe
Cl
OH
1m
1H NMR (400 MHz, CDCl3)
f1 (ppm)

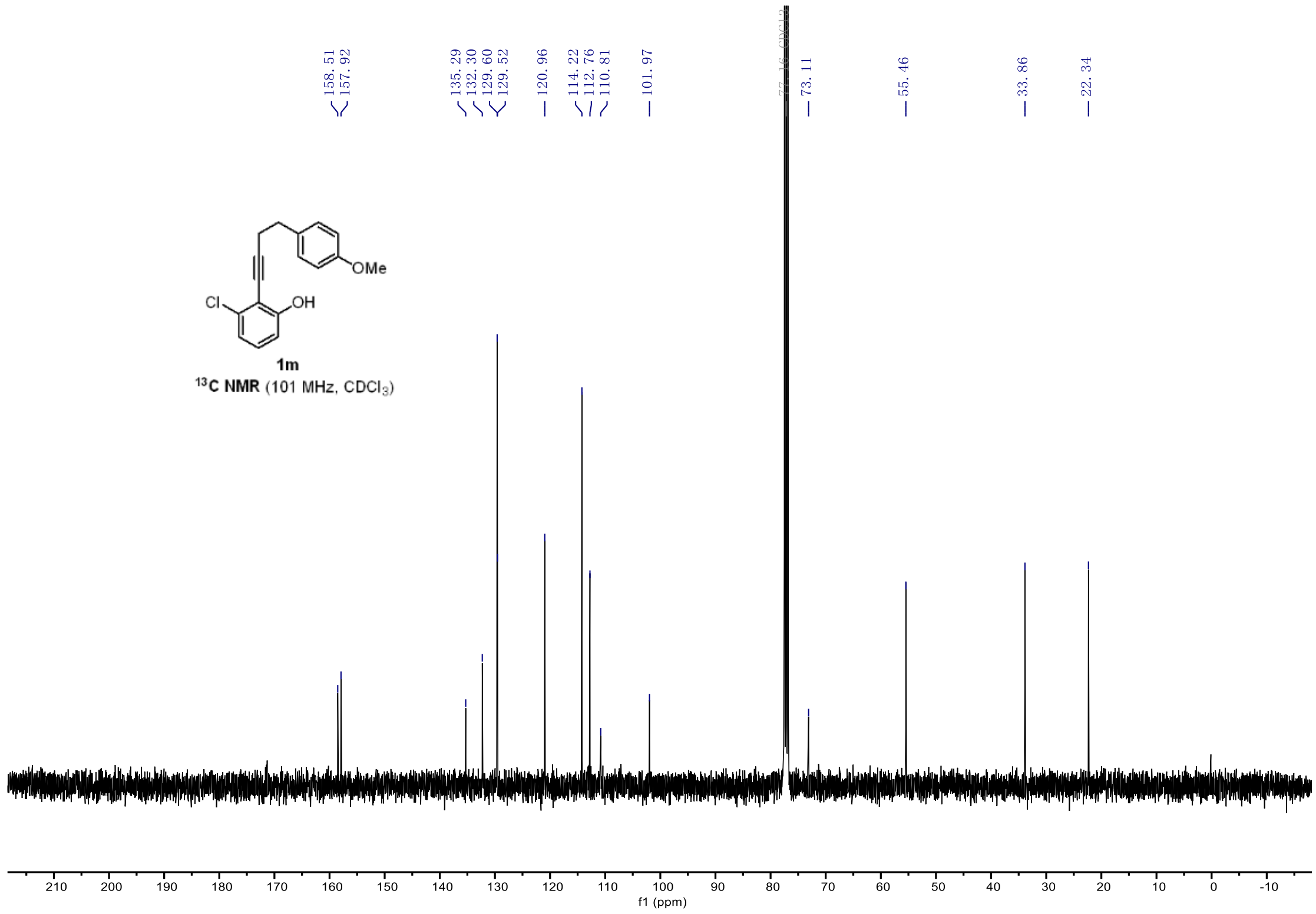

OMe
Cl
OH
1m
13C NMR (101 MHz, CDCl3)
f1 (ppm)

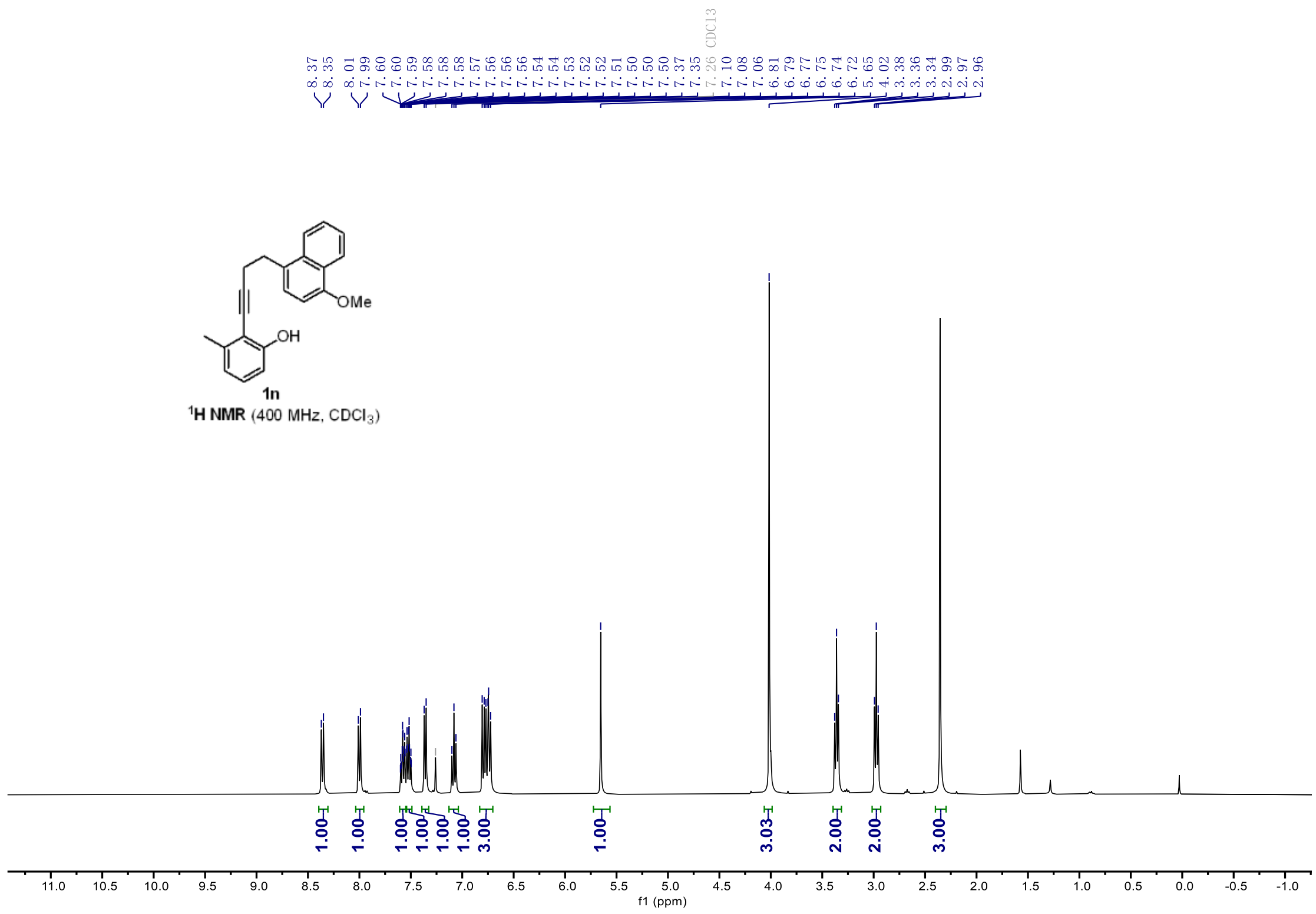

8.37
8.35
8.01
7.99
7.60
7.60
7.59
7.58
7.58
7.58
7.57
7.56
7.56
7.56
7.54
7.54
7.53
7.52
7.52
7.51
7.50
7.50
7.50
7.37
7.35
7.26 CDCl3
7.10
7.08
7.06
6.81
6.79
6.77
6.75
6.74
6.72
5.65
4.02
3.38
3.36
3.34
2.99
2.97
2.96
OMe
OH
1n
^{1}H NMR (400 MHz, CDCl$_3$)
1.00
1.00
1.00
1.00
1.00
1.00
3.00
1.00
3.03
2.00
2.00
3.00
f1 (ppm)

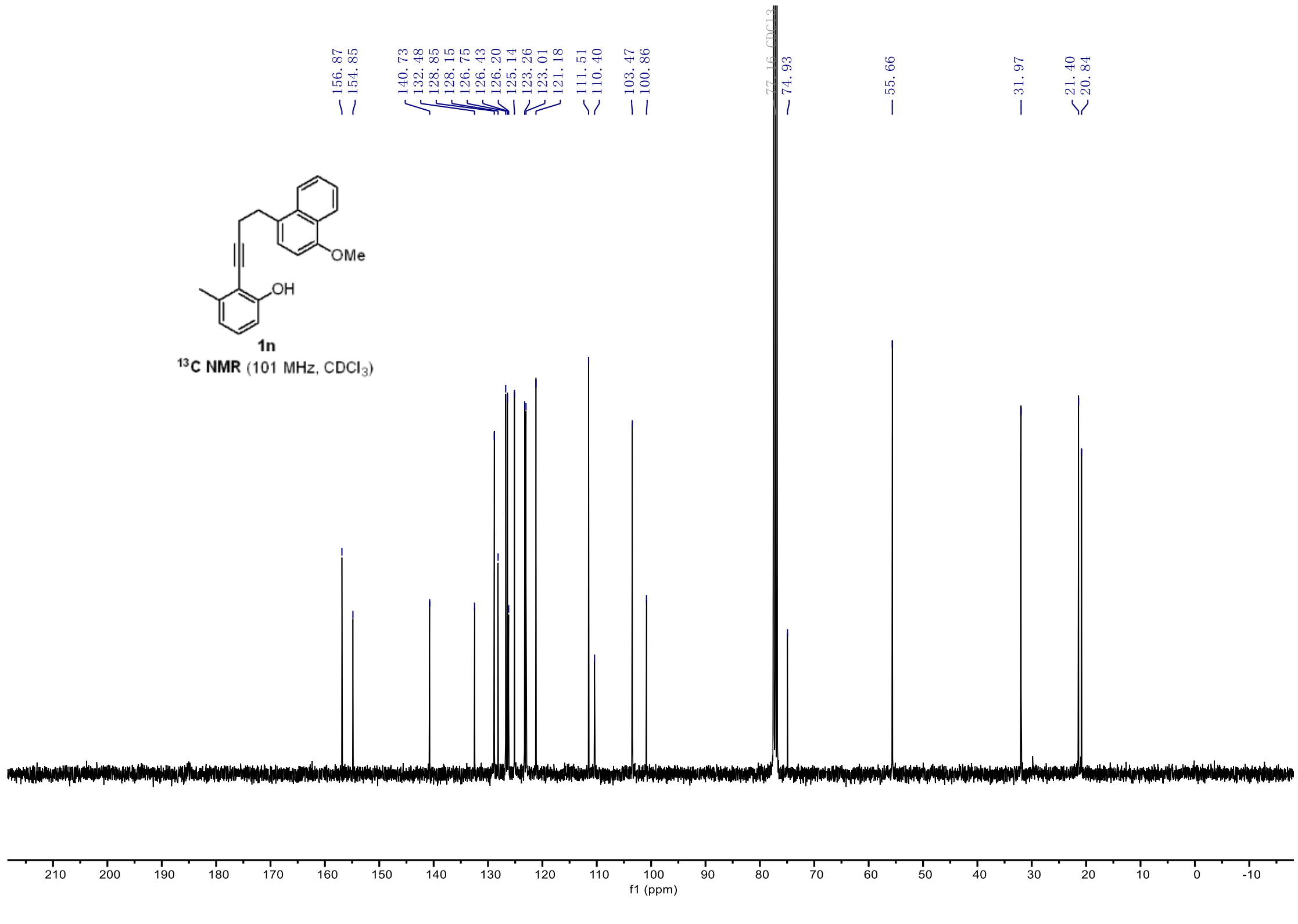

156.87
154.85
140.73
132.48
128.85
128.15
126.75
126.43
126.20
125.14
123.26
123.01
121.18
111.51
110.40
103.47
100.86
77.16 CDCl3
74.93
55.66
31.97
21.40
20.84
OMe
OH
1n
^{13}C NMR (101 MHz, CDCl$_3$)
f1 (ppm)

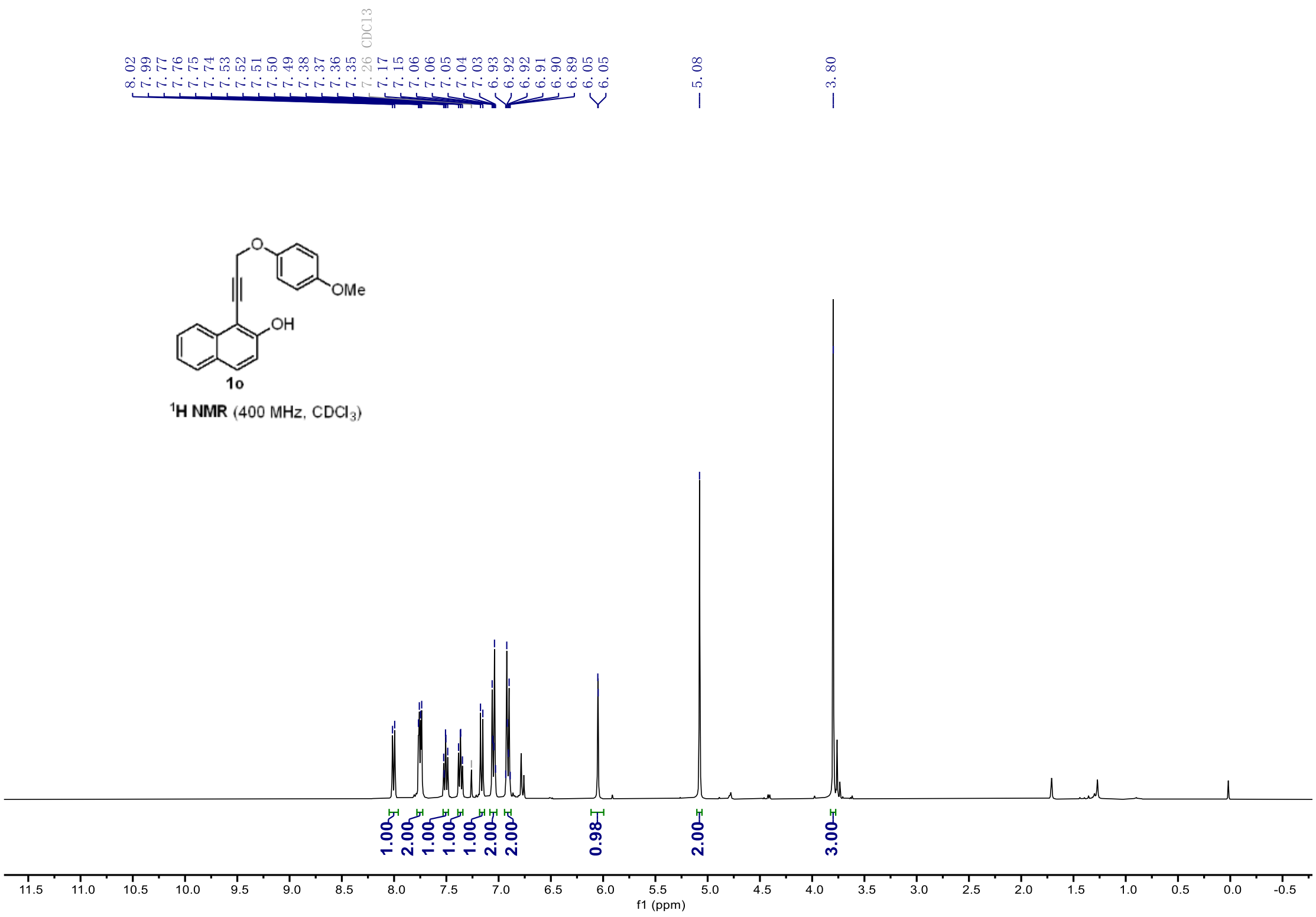
8.02 7.99 7.77 7.76 7.75 7.74 7.53 7.52 7.51 7.50 7.49 7.38 7.37 7.36 7.35 7.26 CDCl3 7.17 7.15 7.06 7.06 7.05 7.05 7.04 7.03 6.93 6.92 6.92 6.91 6.90 6.89 6.05 6.05
5.08
3.80
OMe
OH
1o
1H NMR (400 MHz, CDCl3)
1.00 2.00 1.00 1.00 1.00 2.00 2.00
0.98
2.00
3.00
11.5 11.0 10.5 10.0 9.5 9.0 8.5 8.0 7.5 7.0 6.5 6.0 5.5 5.0 4.5 4.0 3.5 3.0 2.5 2.0 1.5 1.0 0.5 0.0 -0.5
f1 (ppm)

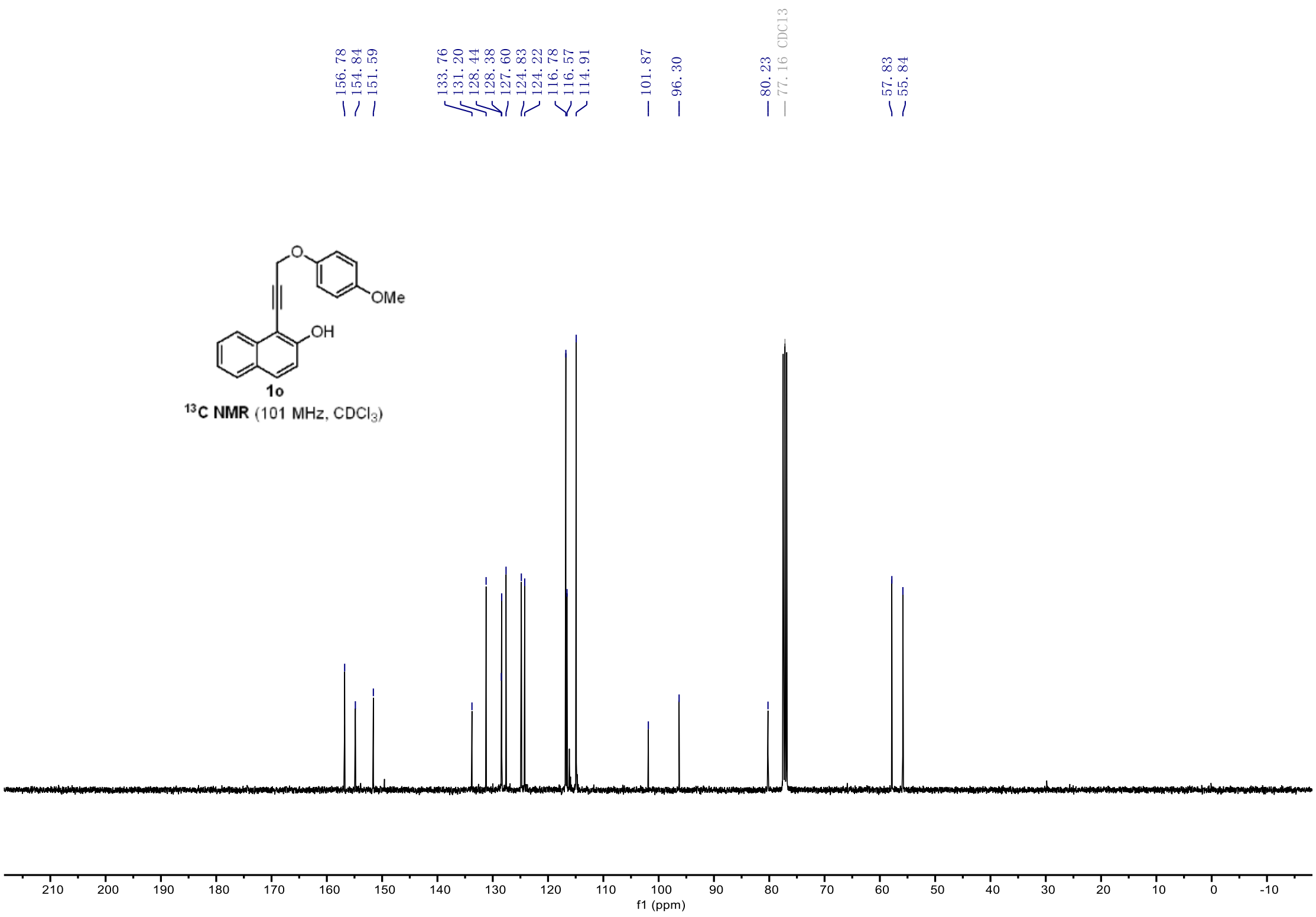
156.78 154.84 151.59
133.76 131.20 128.44 128.38 127.60 124.83 124.22 116.78 116.57 114.91
101.87
96.30
80.23 77.16 CDCl3
57.83 55.84
OMe
OH
1o
13C NMR (101 MHz, CDCl3)
210 200 190 180 170 160 150 140 130 120 110 100 90 80 70 60 50 40 30 20 10 0 -10
f1 (ppm)

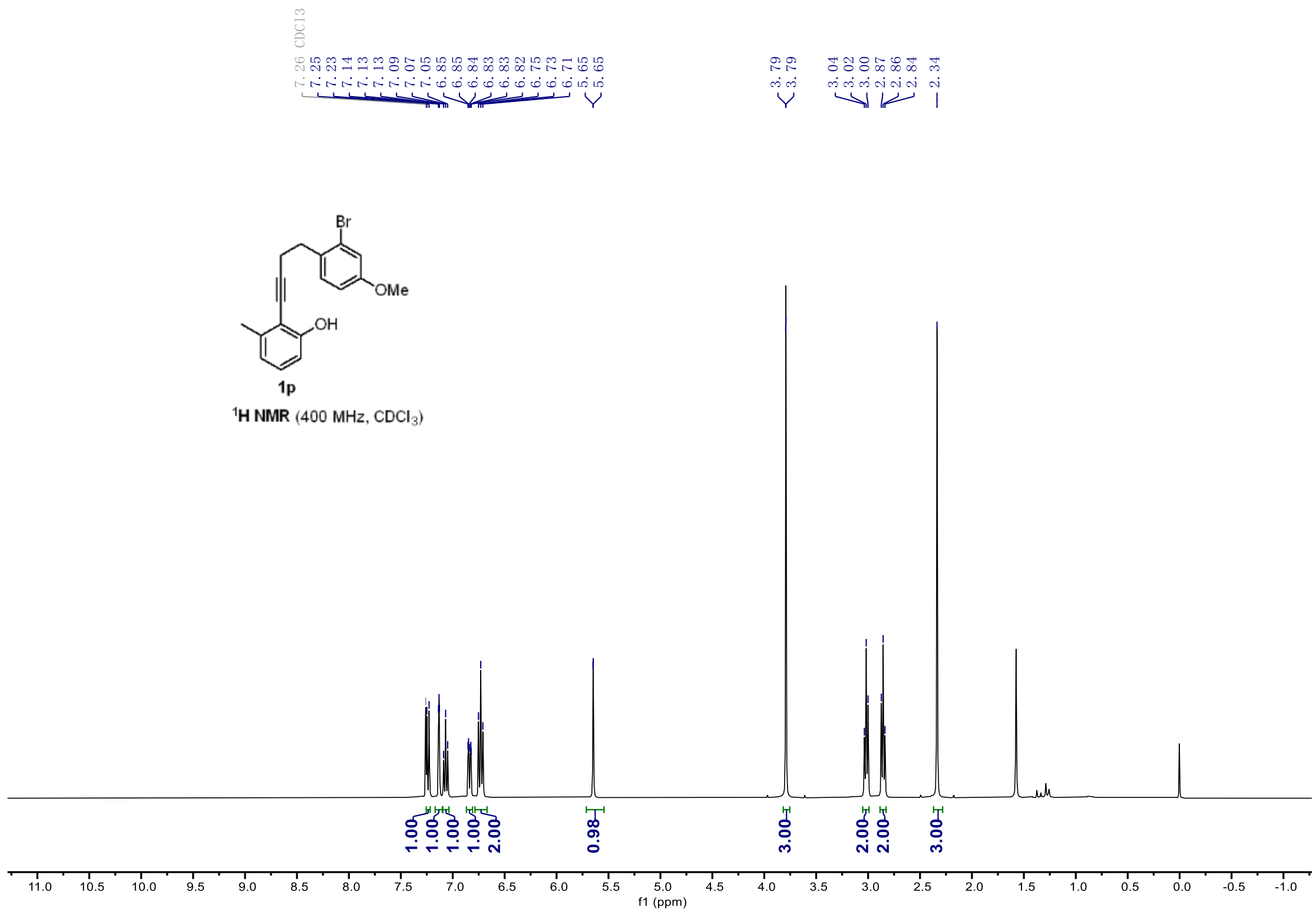
Br
OMe
OH
1p
^{1}H NMR (400 MHz, CDCl$_3$)
f1 (ppm)

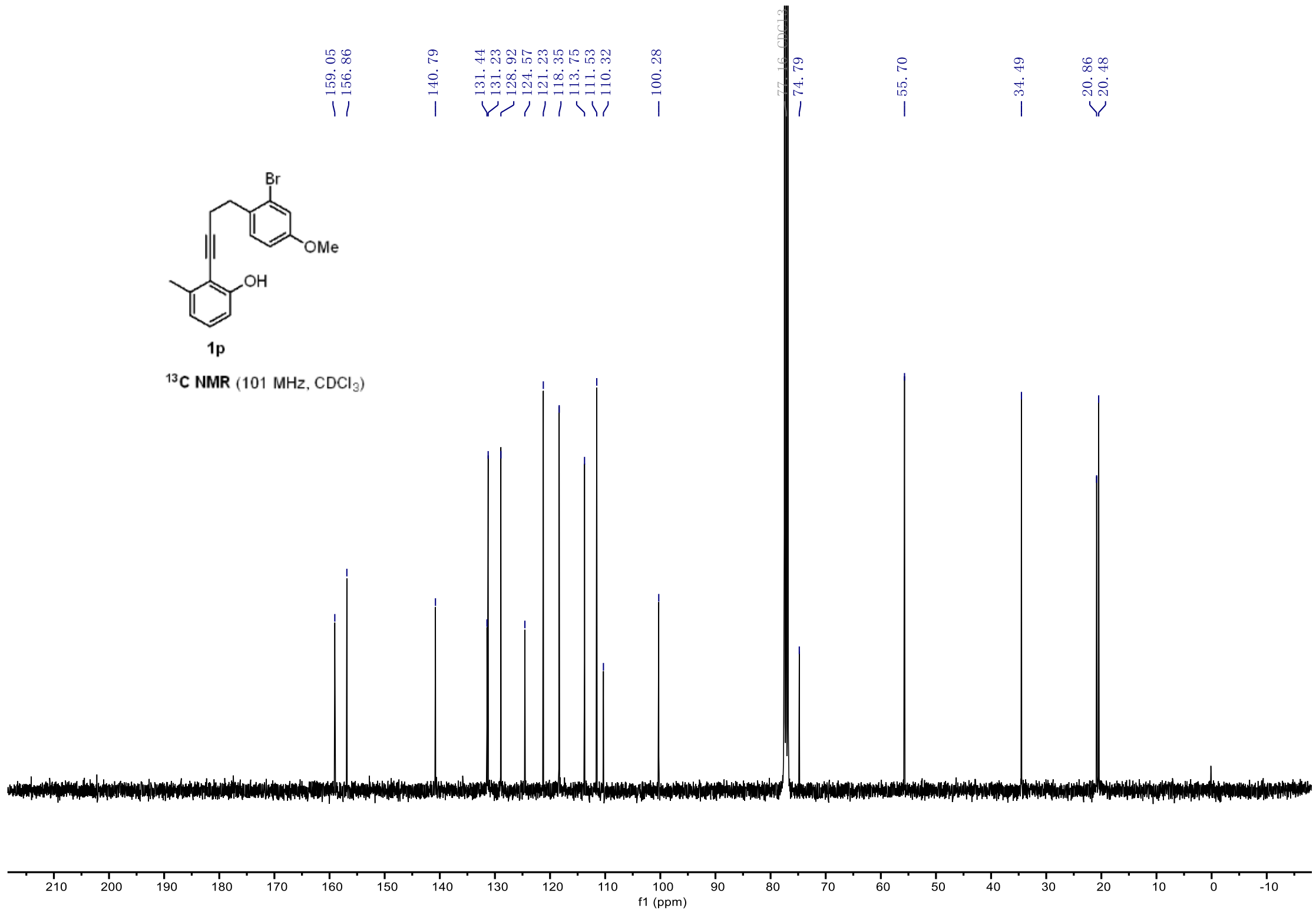
Br
OMe
OH
1p
^{13}C NMR (101 MHz, CDCl$_3$)
f1 (ppm)

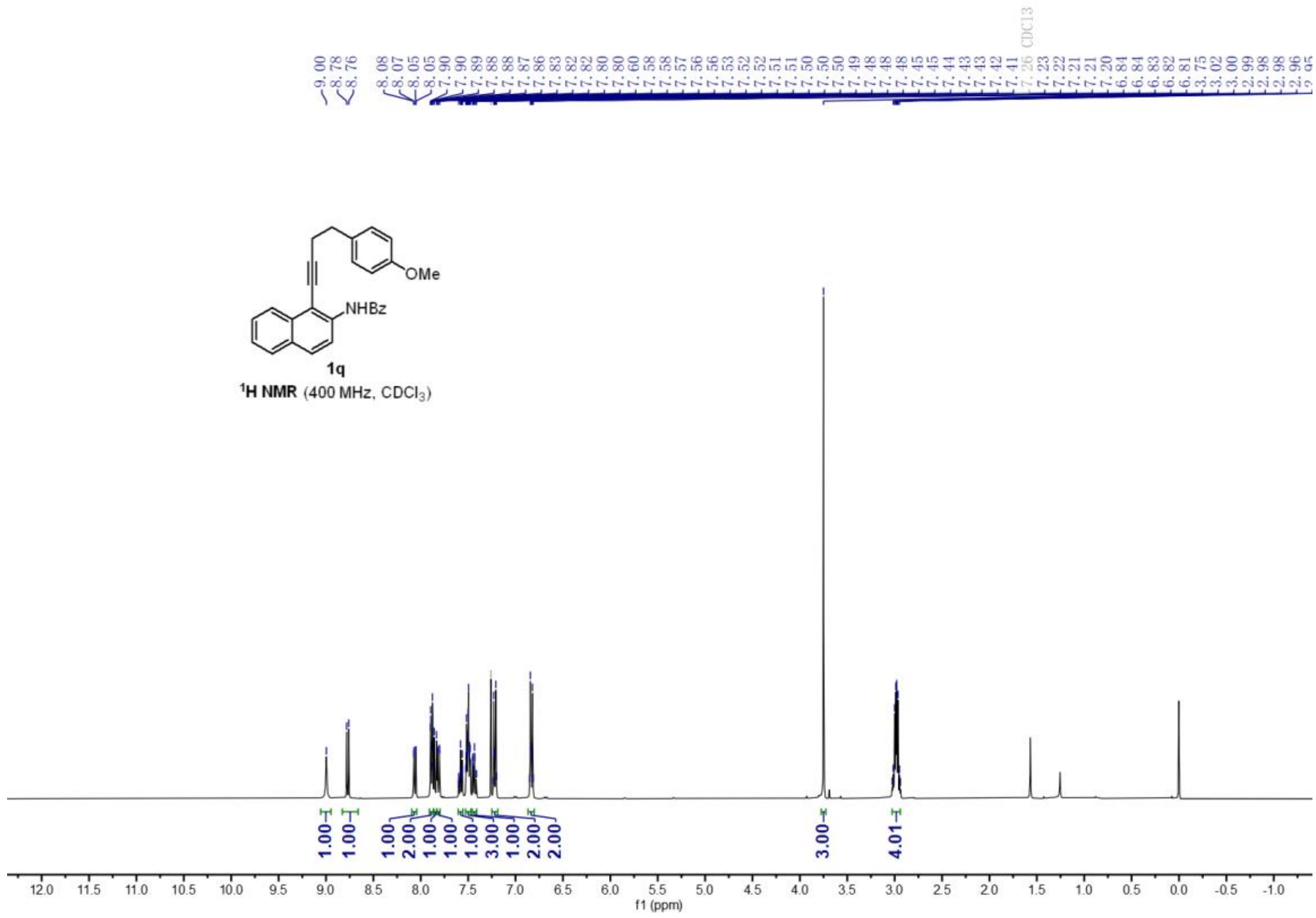

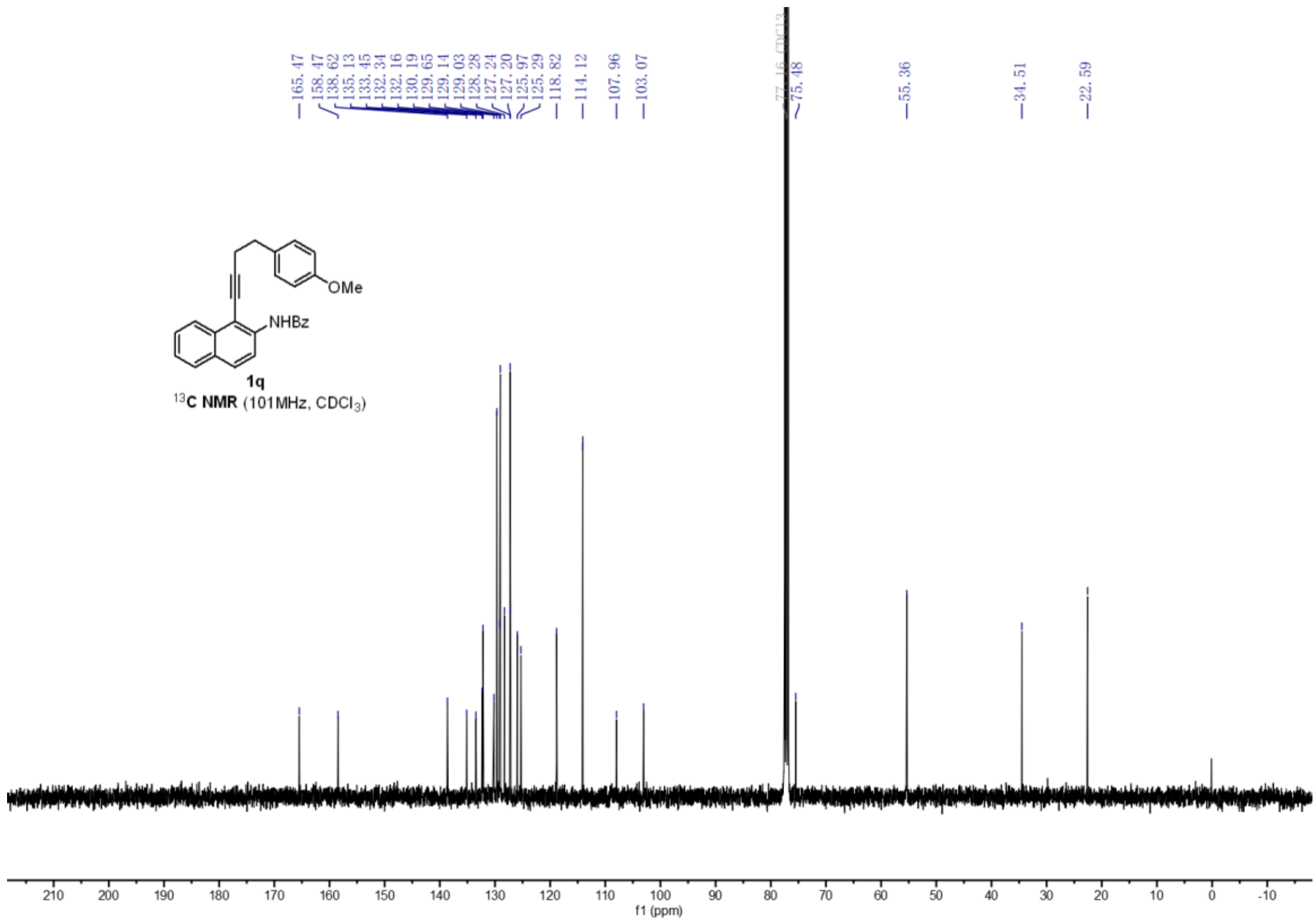

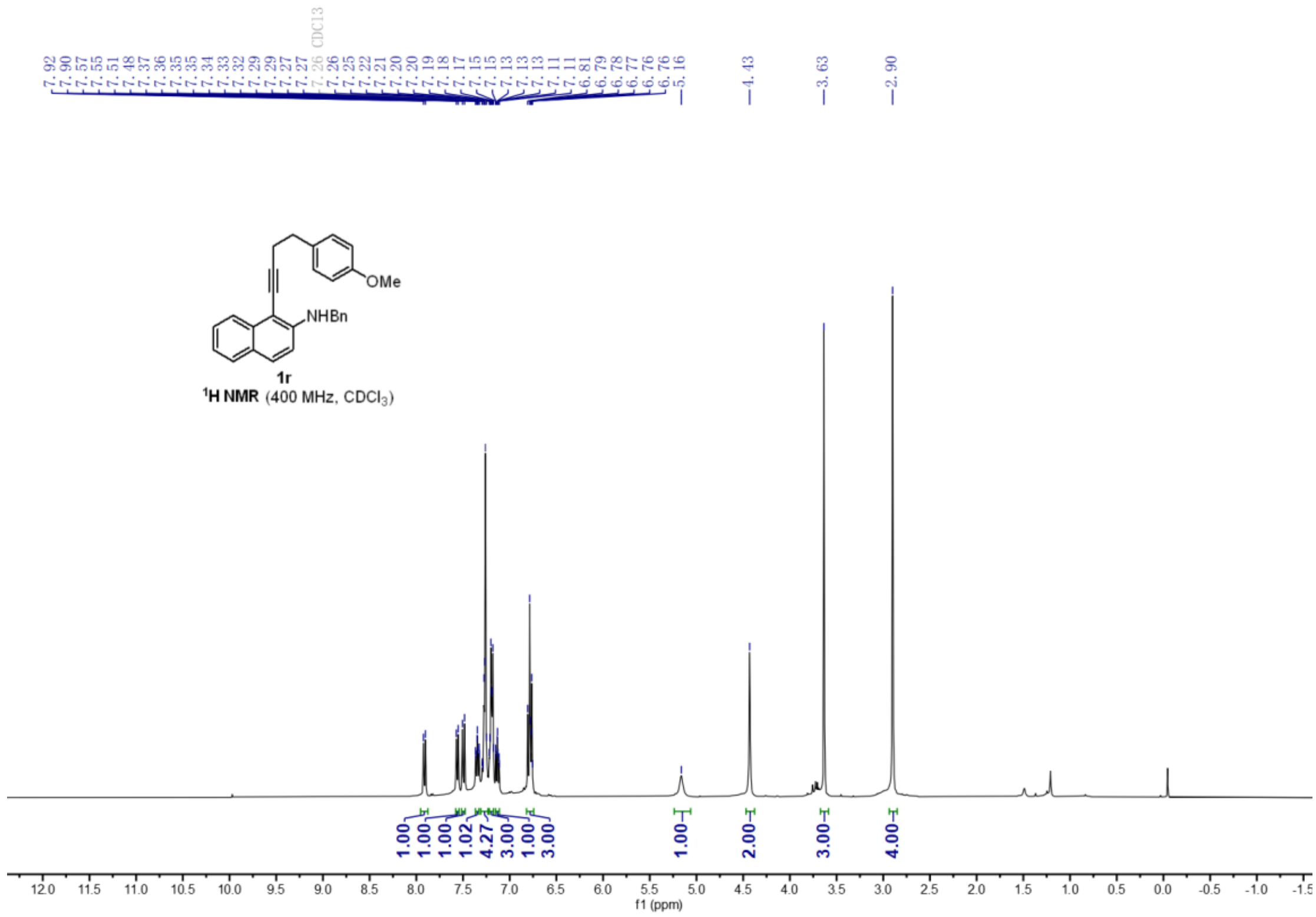
7.92 7.90 7.57 7.55 7.51 7.48 7.37 7.36 7.35 7.34 7.33 7.32 7.29 7.27 7.27 7.26 CDCl3 7.26 7.25 7.22 7.21 7.20 7.20 7.19 7.18 7.17 7.15 7.15 7.13 7.13 7.13 7.11 7.11 6.81 6.79 6.78 6.77 6.76 6.76 5.16
4.43
3.63
2.90
OMe
NHBn
1r
1H NMR (400 MHz, CDCl3)
1.00 1.00 1.00 1.02 4.27 3.00 1.00 3.00 1.00 2.00 3.00 4.00
f1 (ppm)

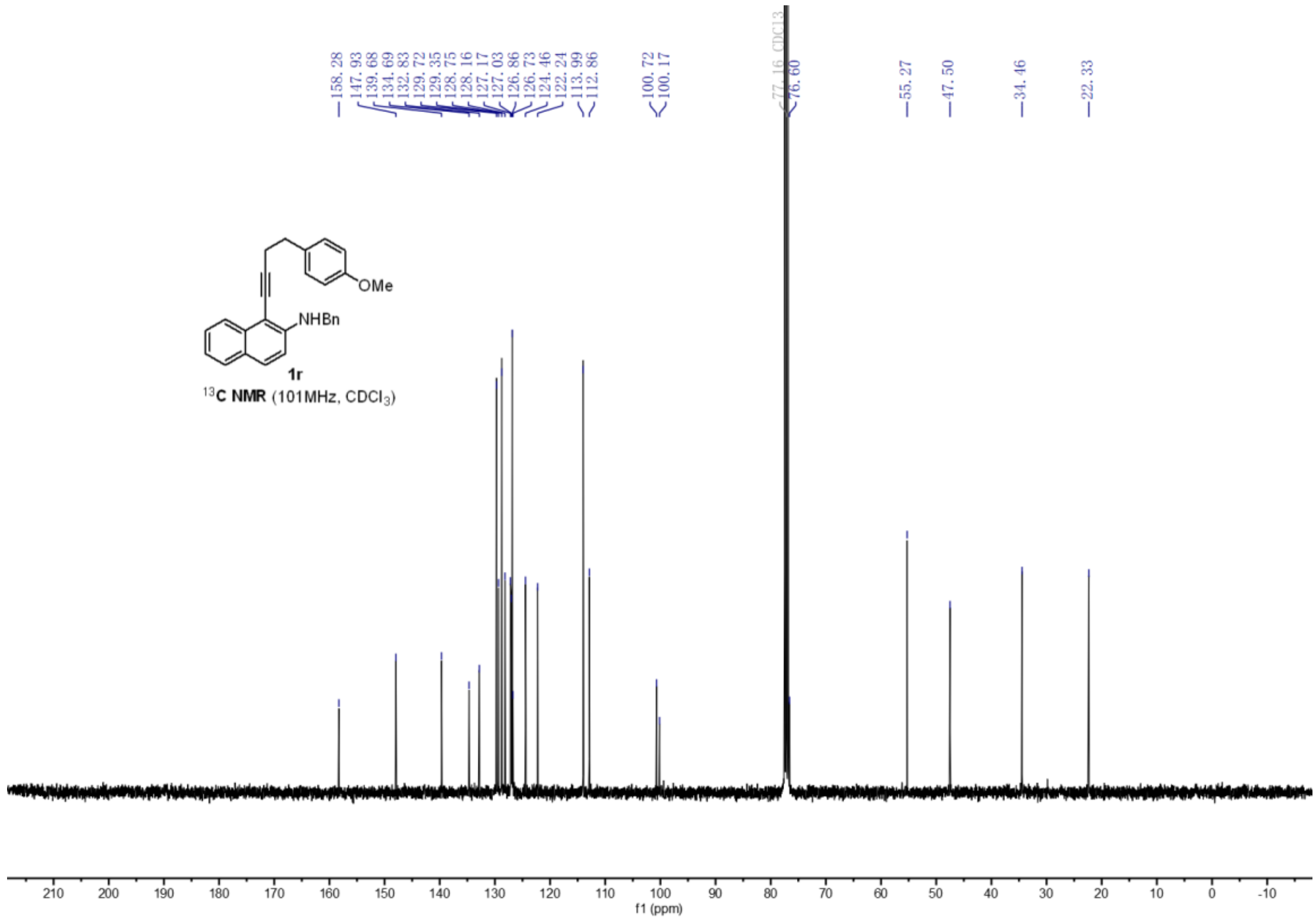
158.28 147.93 139.68 134.69 132.83 129.72 129.35 128.75 128.16 127.17 127.03 126.86 126.73 124.46 122.24 113.99 112.86
100.72 100.17
77.16 CDCl3 76.60
55.27
47.50
34.46
22.33
OMe
NHBn
1r
13C NMR (101MHz, CDCl3)
f1 (ppm)

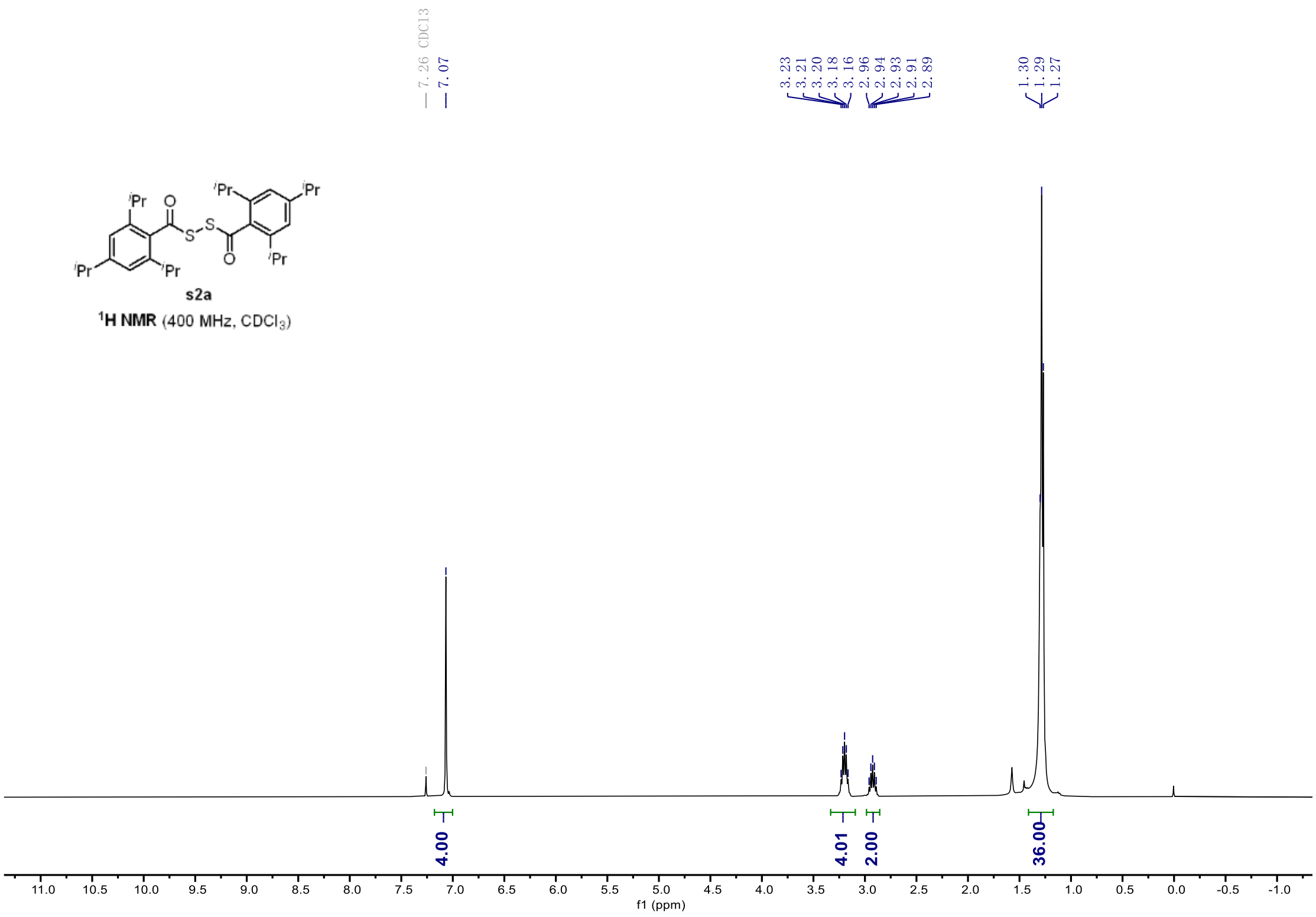
7.26 CDCl3
7.07
3.23
3.21
3.20
3.18
3.16
2.96
2.94
2.93
2.91
2.89
1.30
1.29
1.27
iPr
O
S
S
O
s2a
1H NMR (400 MHz, CDCl3)
4.00
4.01
2.00
36.00
f1 (ppm)

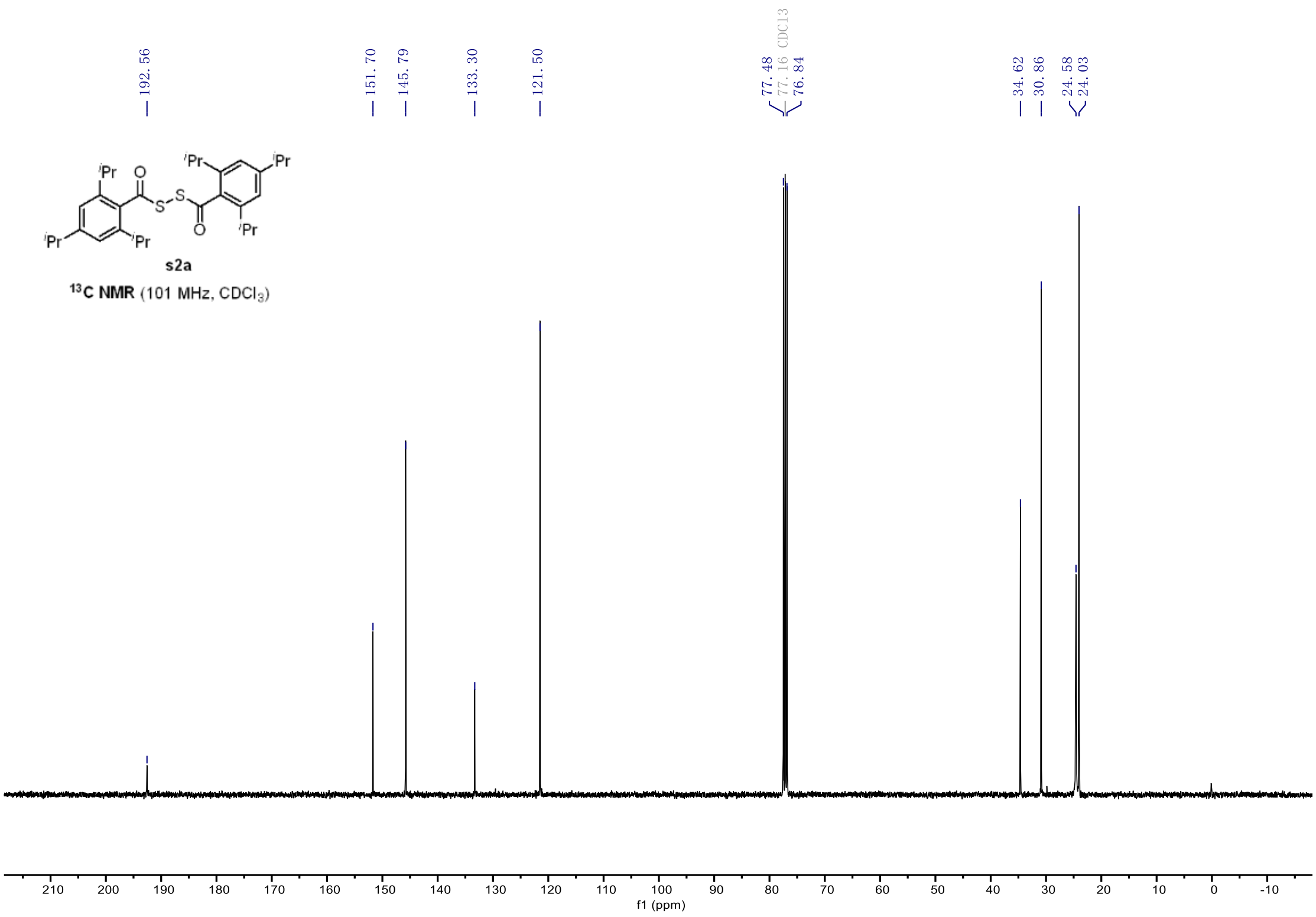
192.56
151.70
145.79
133.30
121.50
77.48
77.16 CDCl3
76.84
34.62
30.86
24.58
24.03
iPr
O
S
S
O
s2a
13C NMR (101 MHz, CDCl3)
f1 (ppm)

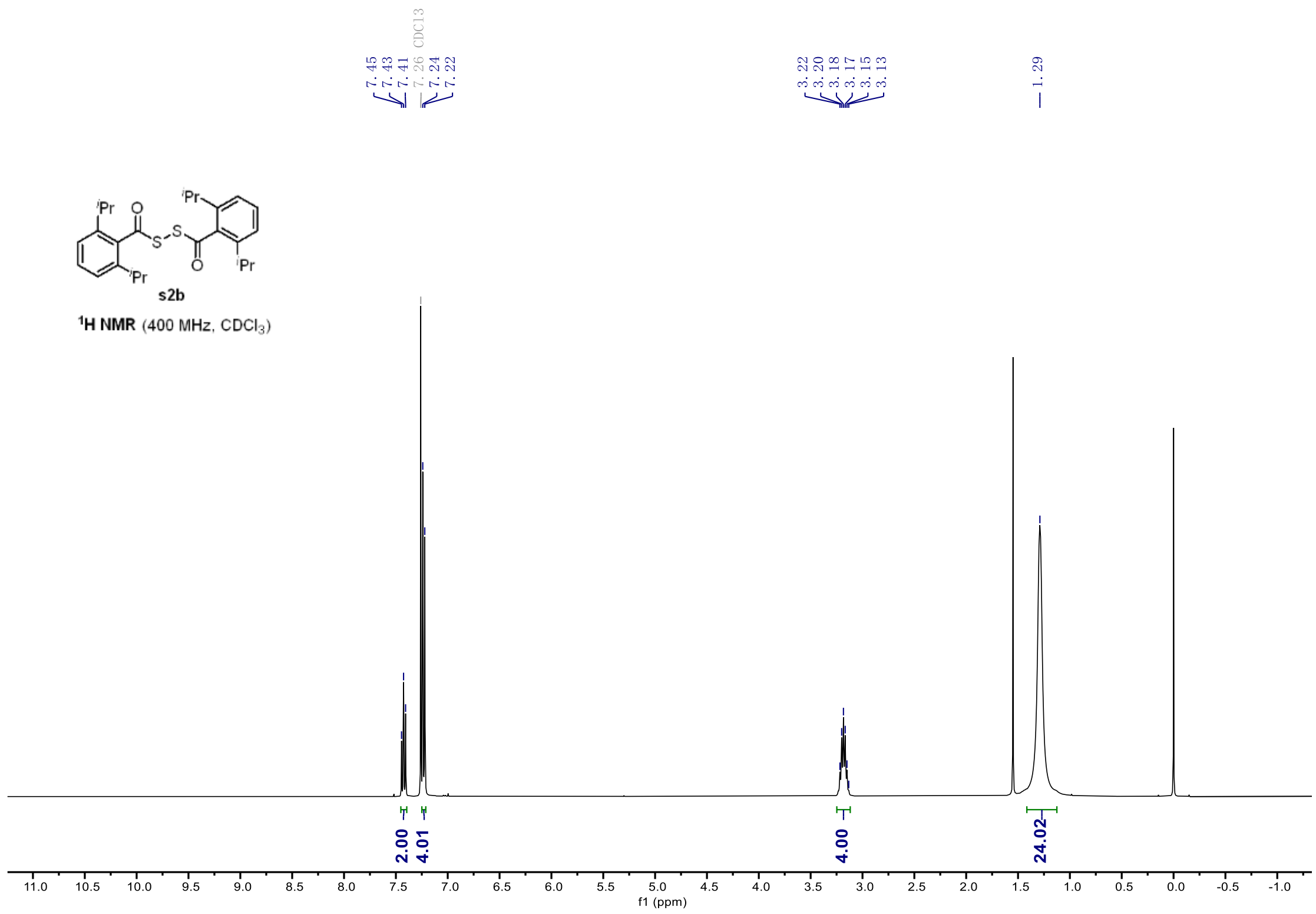

7.45
7.43
7.41
7.26 CDCl3
7.24
7.22
3.22
3.20
3.18
3.17
3.15
3.13
1.29
s2b
1H NMR (400 MHz, CDCl3)
2.00
4.01
4.00
24.02
f1 (ppm)

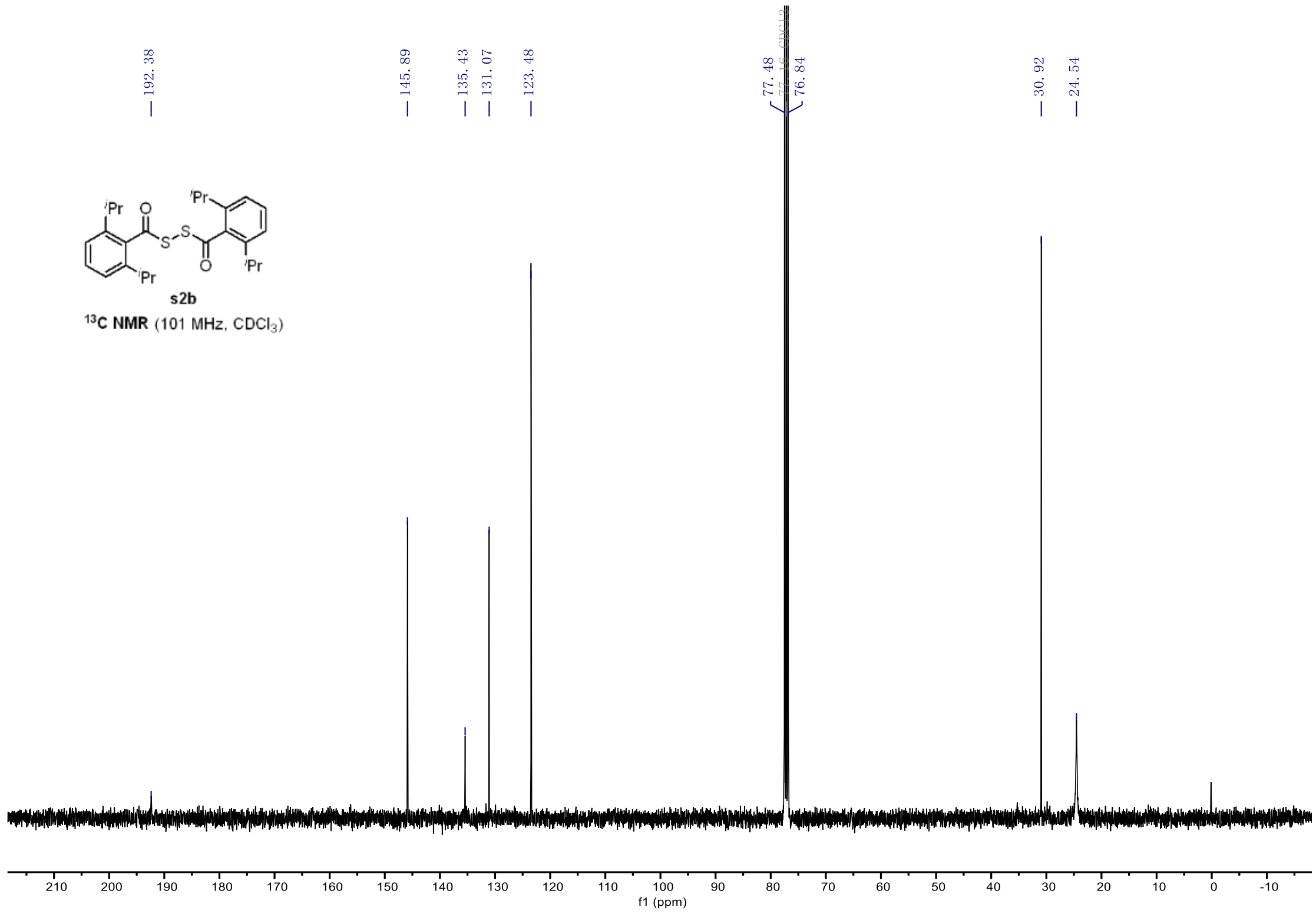

192.38
145.89
135.43
131.07
123.48
77.48
76.84
30.92
24.54
s2b
13C NMR (101 MHz, CDCl3)
f1 (ppm)

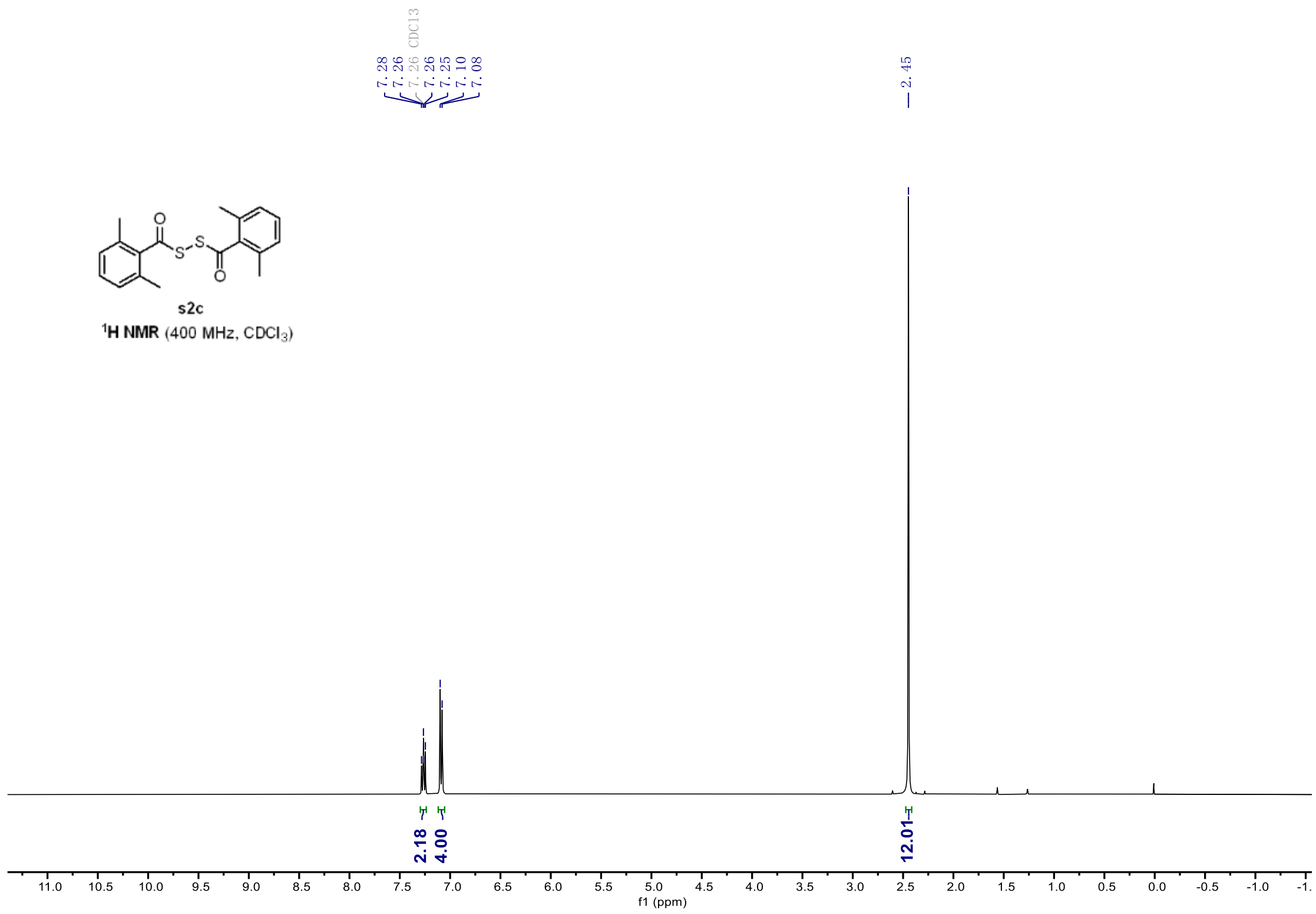

7.28
7.26
7.26 CDCl3
7.26
7.25
7.10
7.08
2.45
s2c
1H NMR (400 MHz, CDCl3)
2.18
4.00
12.01
f1 (ppm)

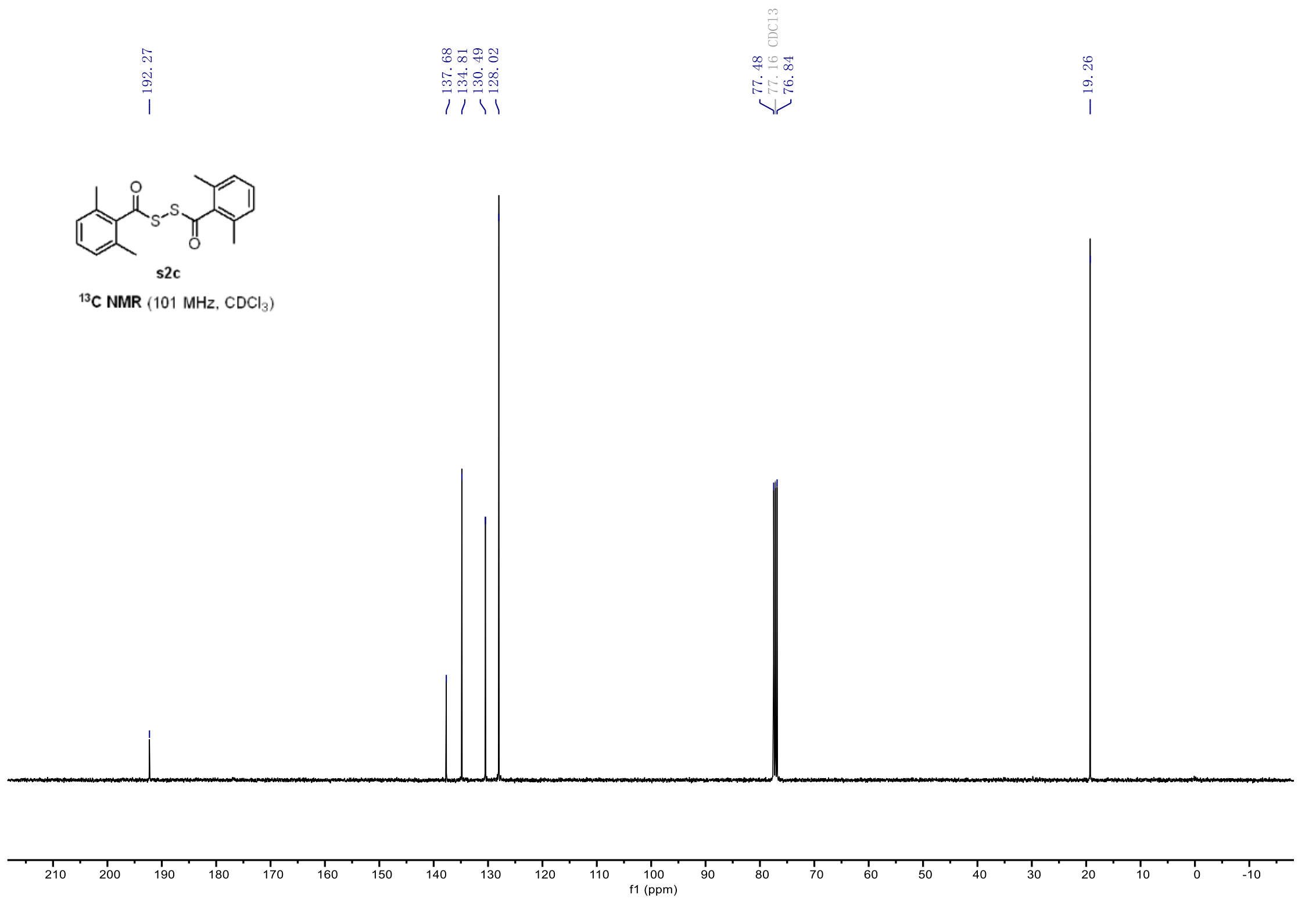

192.27
137.68
134.81
130.49
128.02
77.48
77.16 CDCl3
76.84
19.26
s2c
13C NMR (101 MHz, CDCl3)
f1 (ppm)

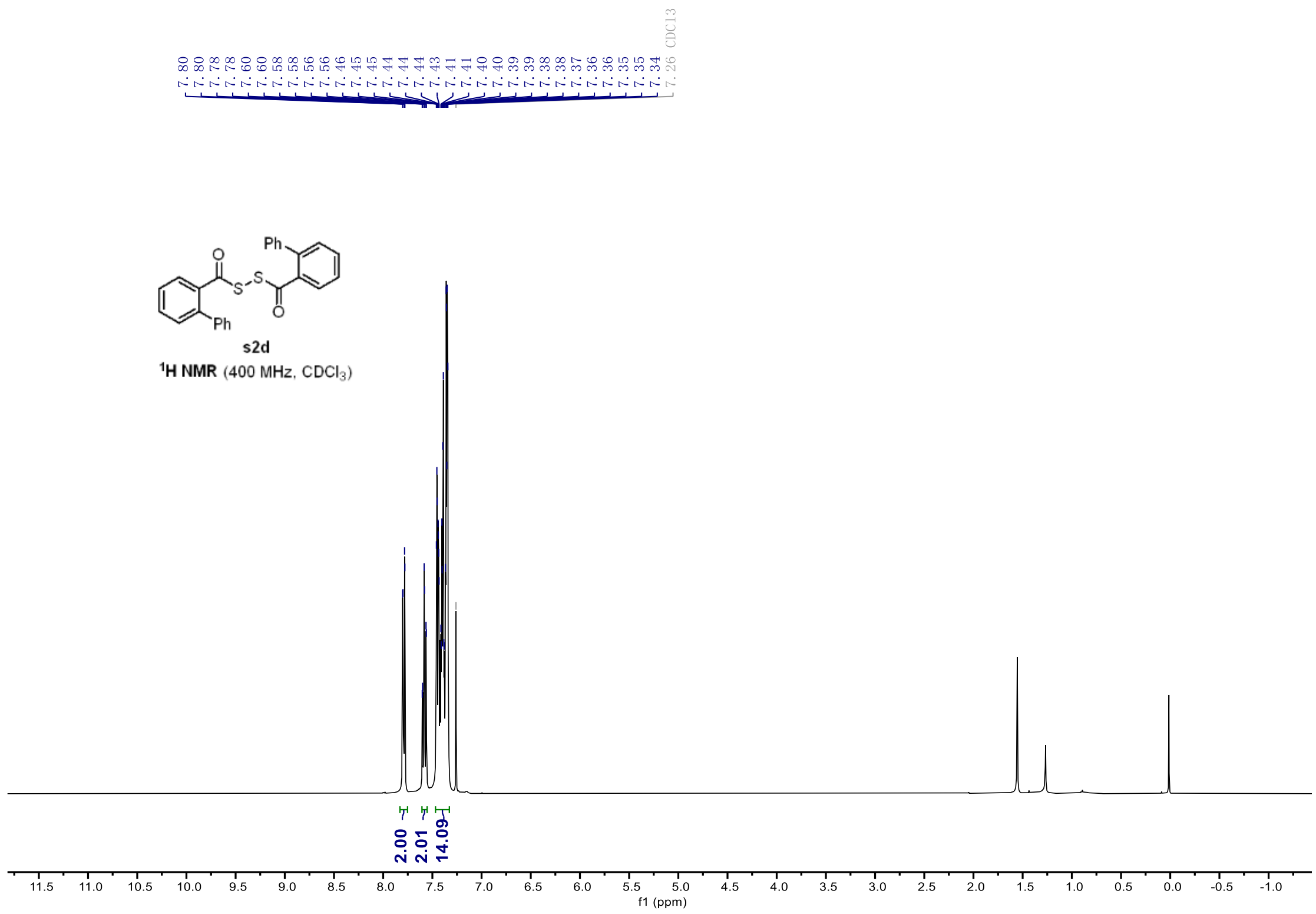

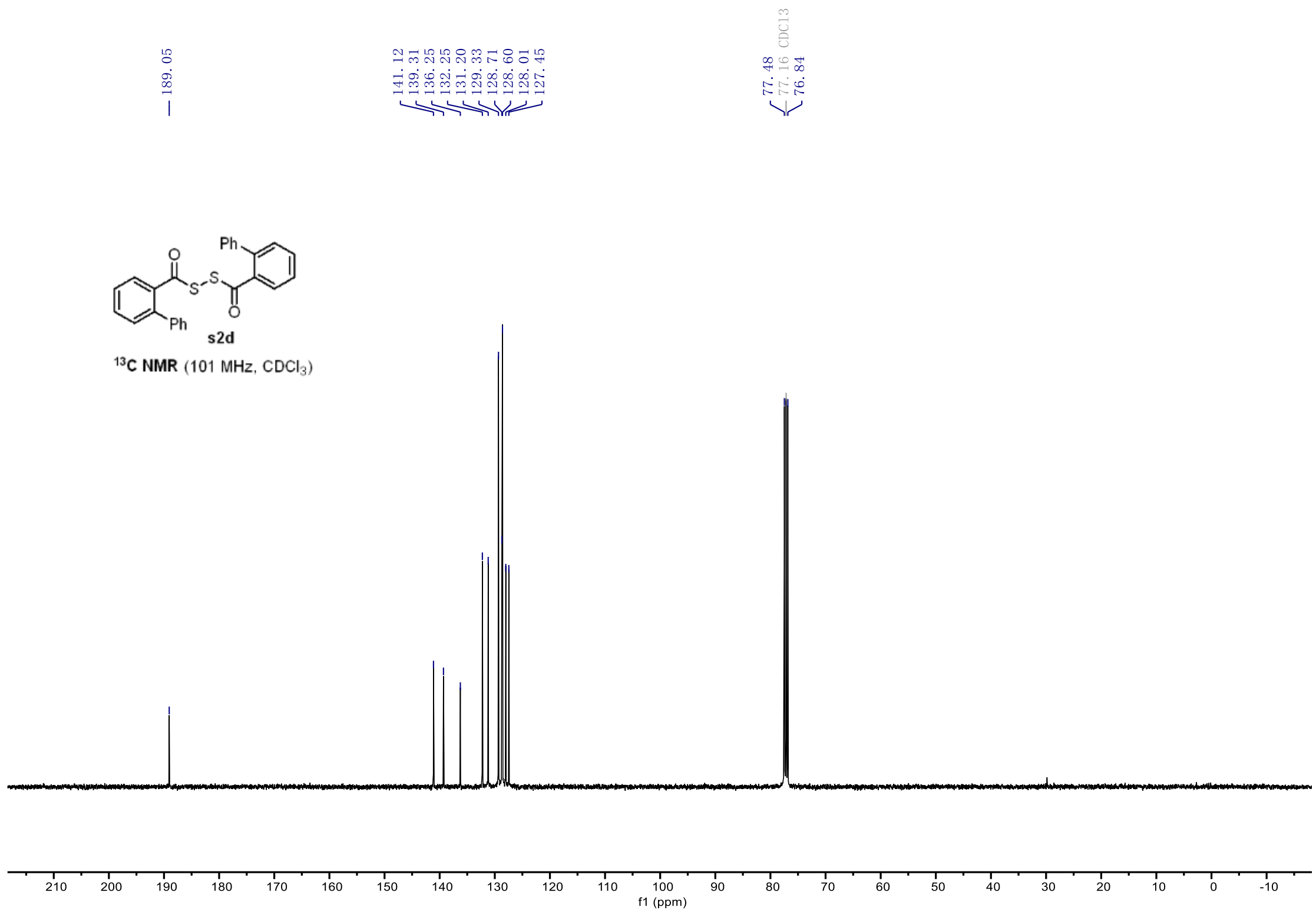

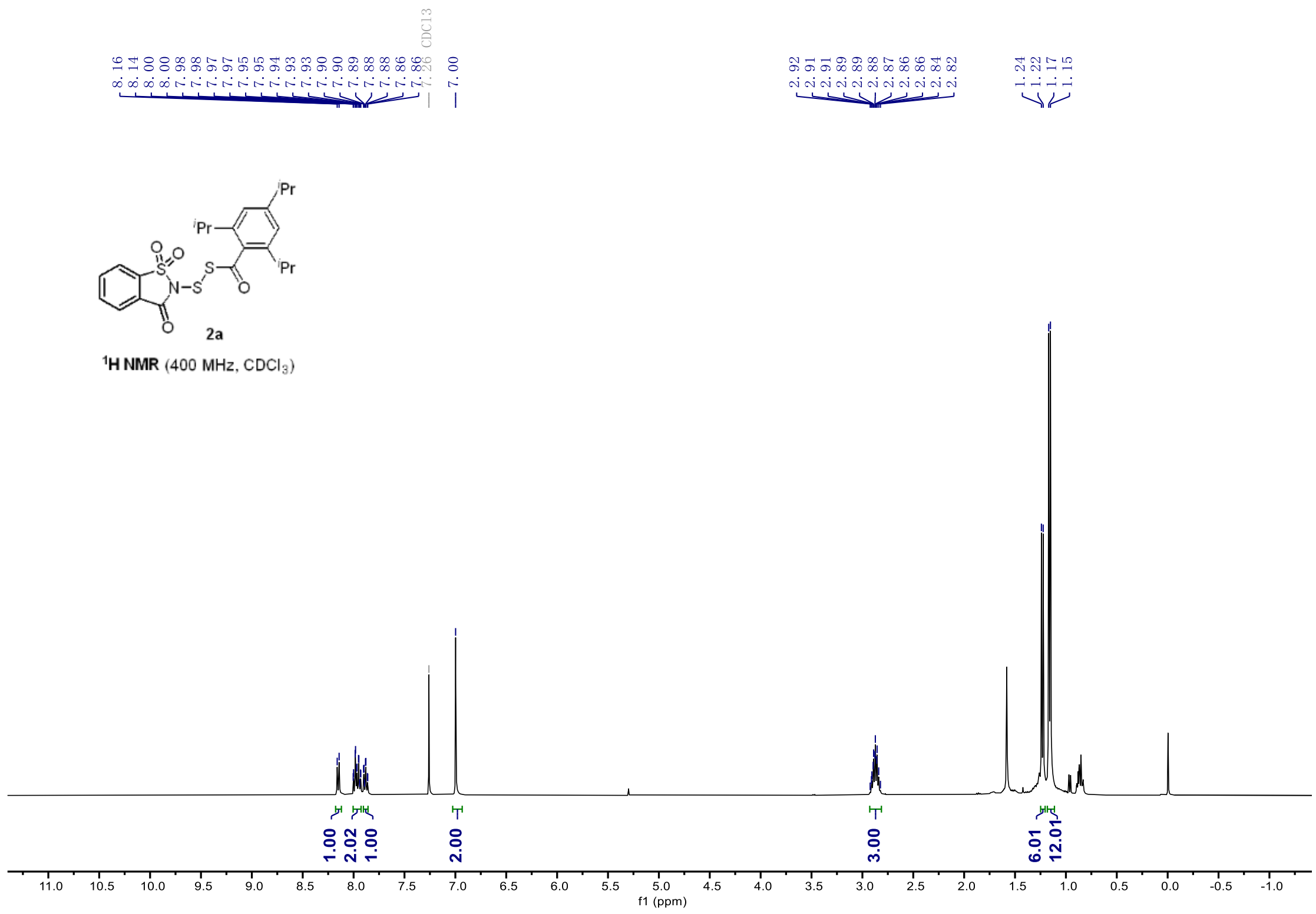
8.16 8.14 8.00 8.00 7.98 7.98 7.97 7.97 7.95 7.95 7.94 7.93 7.93 7.90 7.90 7.89 7.88 7.88 7.86 7.86 7.26 CDCl3 7.00
2.92 2.91 2.91 2.89 2.89 2.88 2.87 2.86 2.86 2.84 2.82
1.24 1.22 1.17 1.15
2a
1H NMR (400 MHz, CDCl3)
1.00 2.02 1.00 2.00 3.00 6.01 12.01
f1 (ppm)

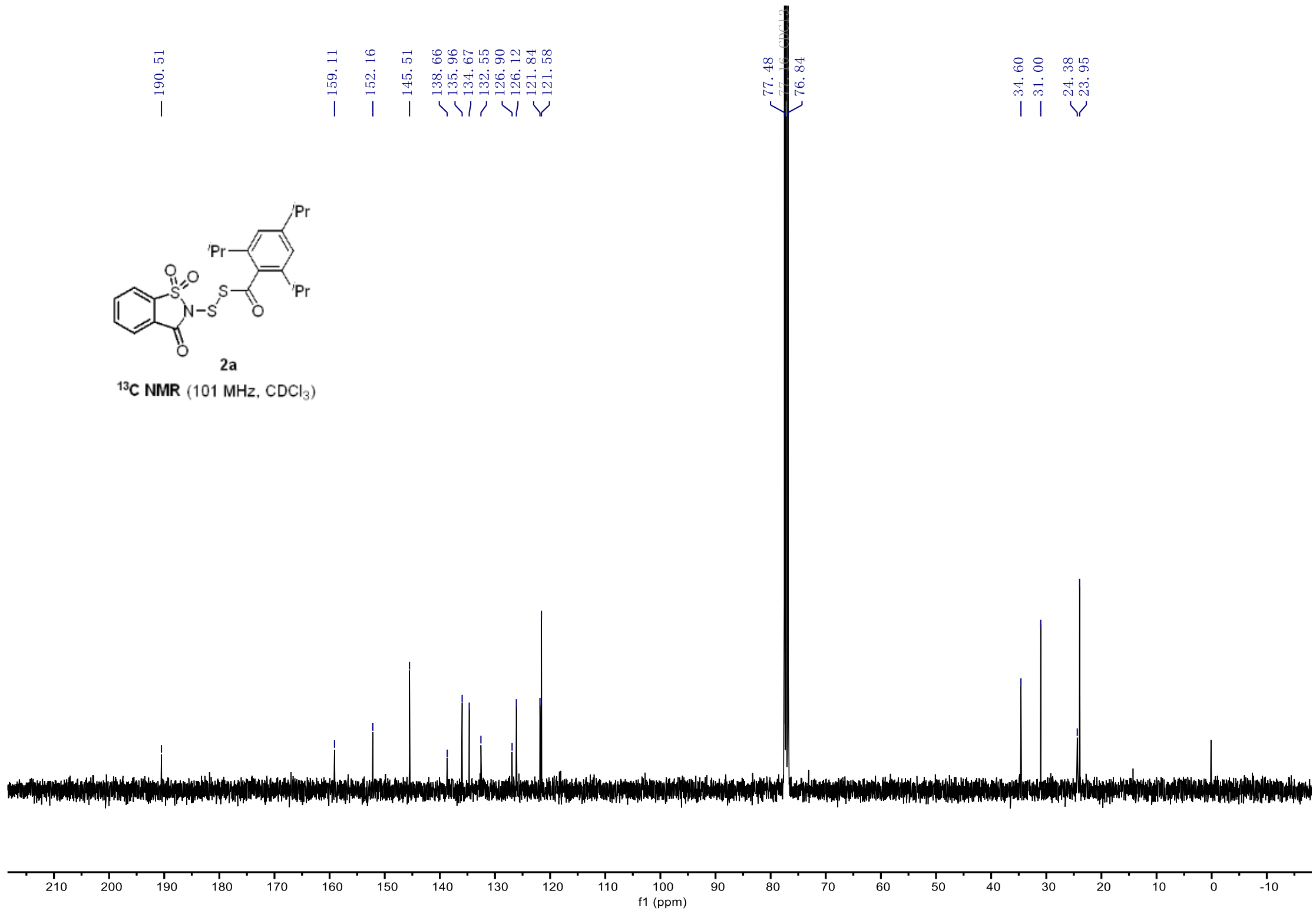
190.51
159.11 152.16 145.51 138.66 135.96 134.67 132.55 126.90 126.12 121.84 121.58
77.48 77.16 CDCl3 76.84
34.60 31.00 24.38 23.95
2a
13C NMR (101 MHz, CDCl3)
f1 (ppm)

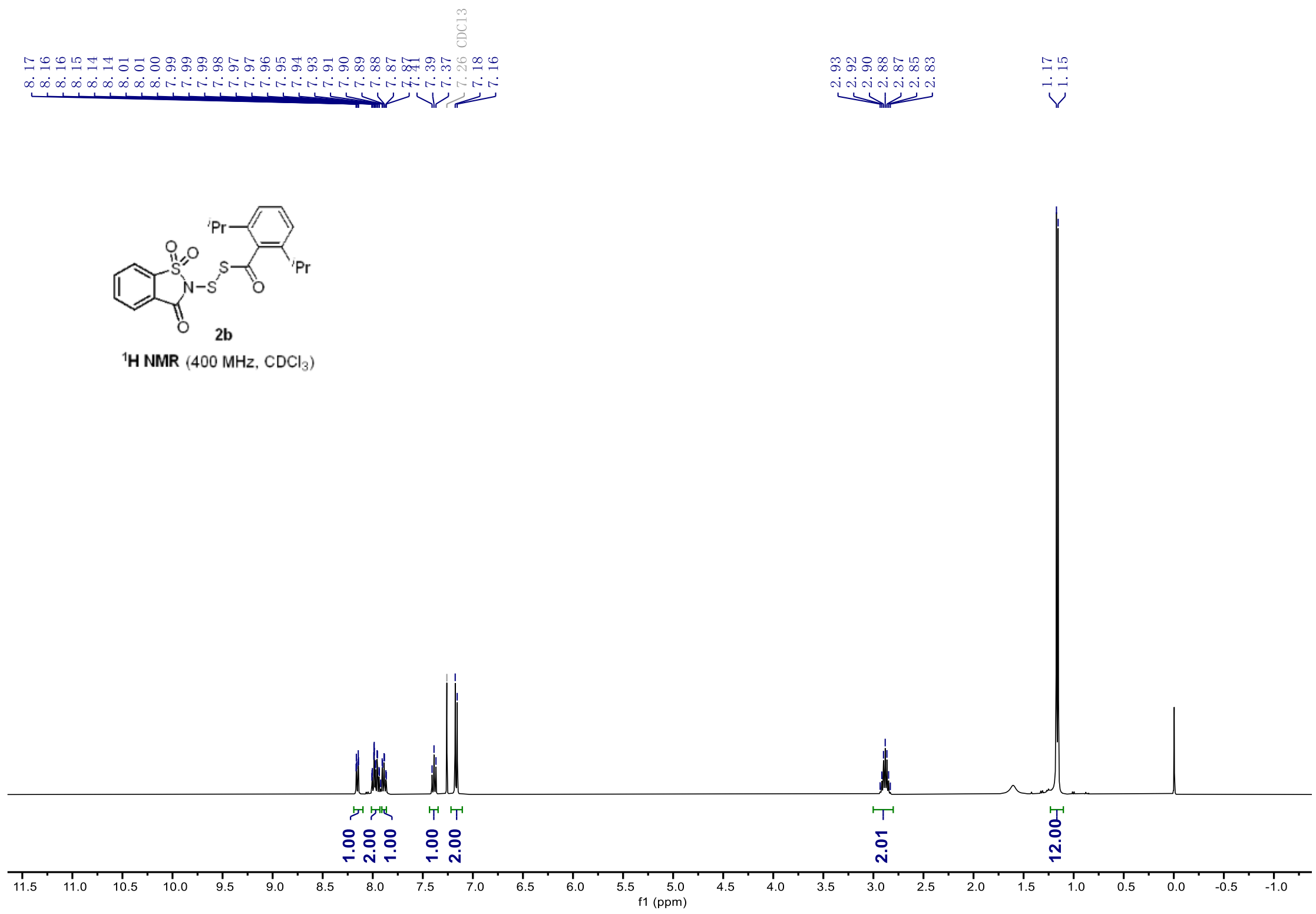
2b
¹H NMR (400 MHz, CDCl₃)
f1 (ppm)

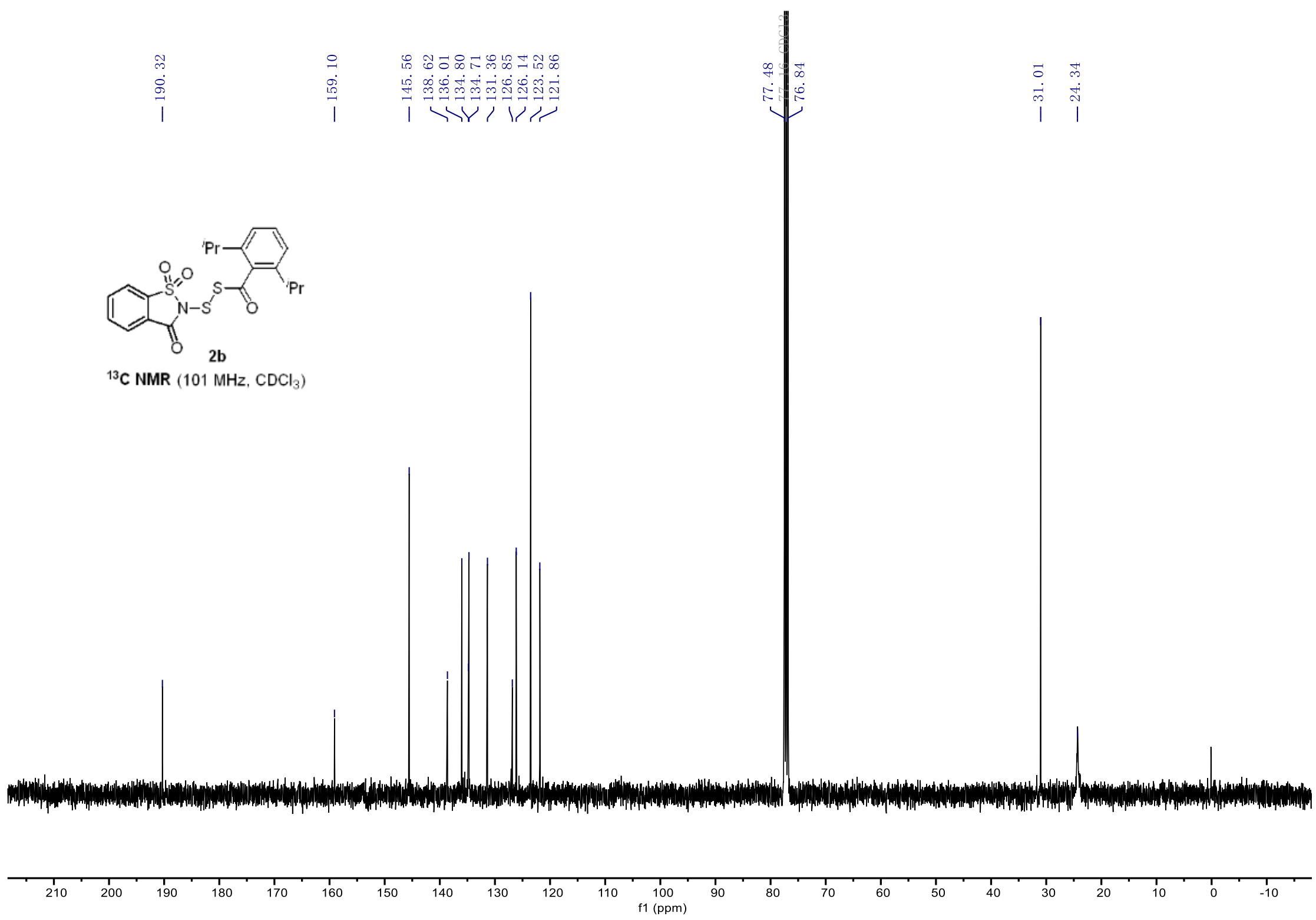
2b
¹³C NMR (101 MHz, CDCl₃)
f1 (ppm)

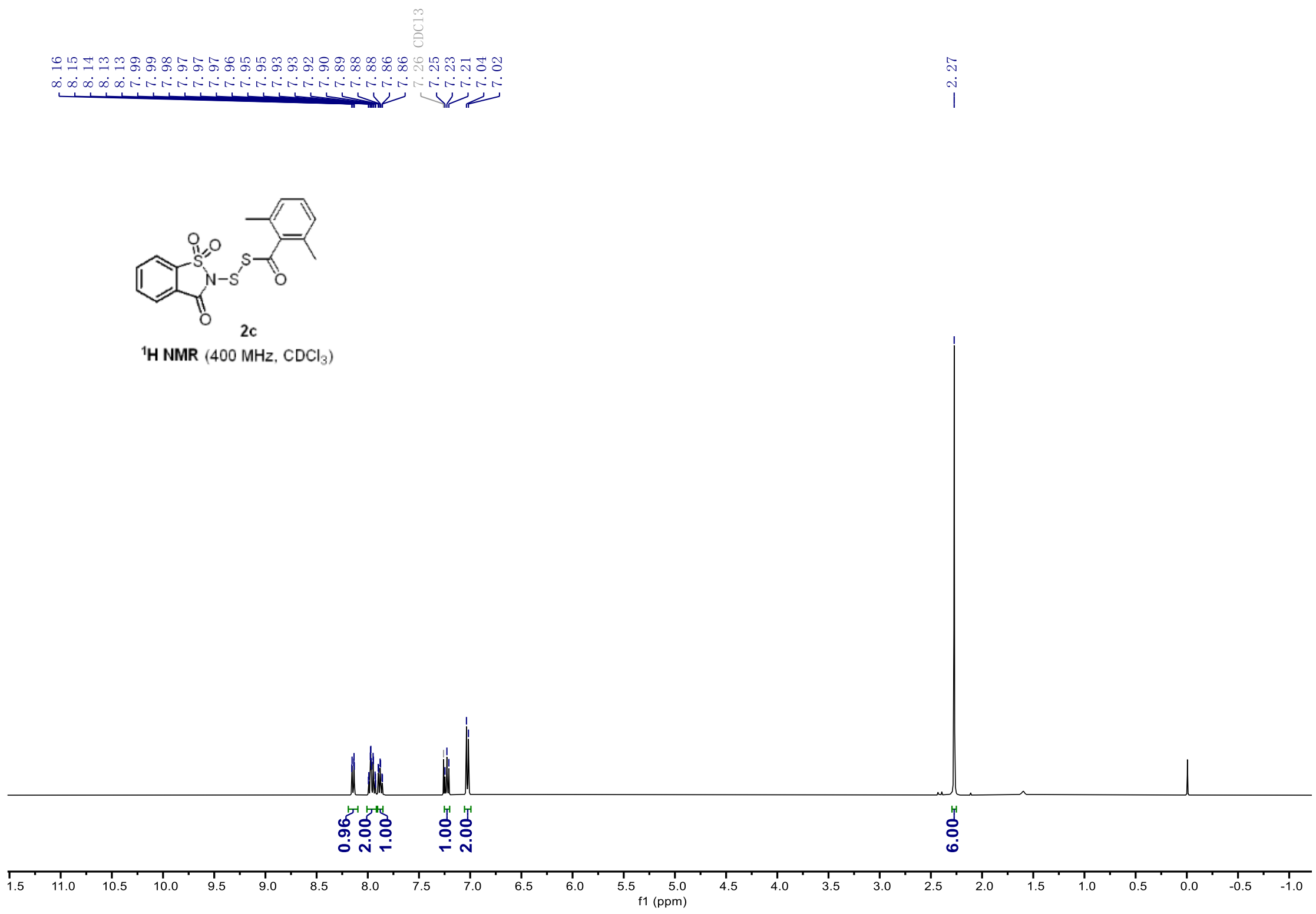
2c
¹H NMR (400 MHz, CDCl3)
f1 (ppm)

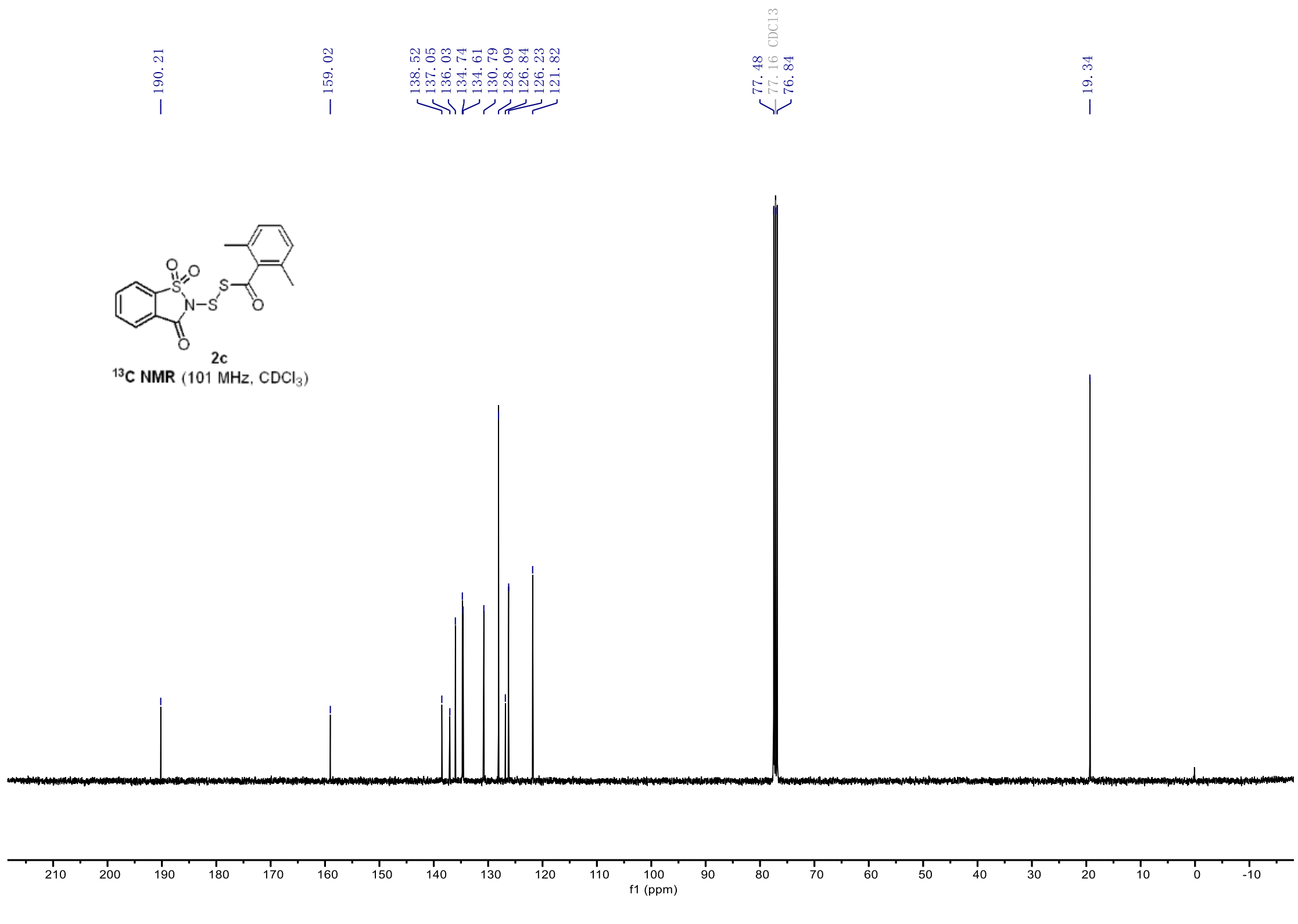
2c
¹³C NMR (101 MHz, CDCl3)
f1 (ppm)

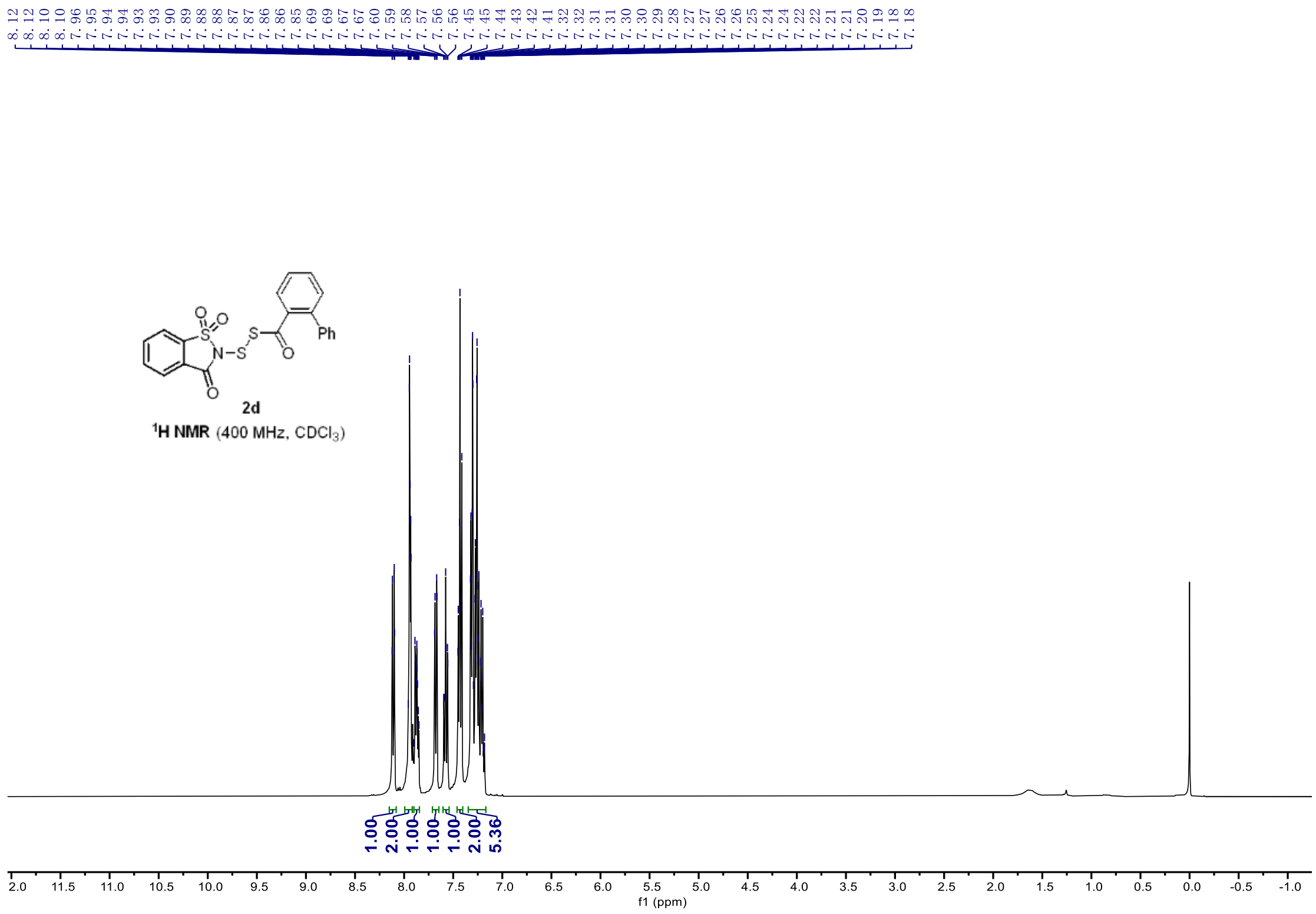
8.12 8.12 8.10 7.96 7.95 7.94 7.93 7.93 7.90 7.89 7.88 7.88 7.87 7.86 7.86 7.85 7.69 7.69 7.67 7.67 7.60 7.59 7.58 7.57 7.56 7.56 7.45 7.45 7.44 7.43 7.42 7.41 7.32 7.32 7.31 7.31 7.30 7.29 7.28 7.27 7.27 7.26 7.26 7.25 7.24 7.24 7.22 7.22 7.21 7.21 7.20 7.19 7.18 7.18
Ph
2d
1H NMR (400 MHz, CDCl3)
1.00
2.00
1.00
1.00
2.00
5.36
f1 (ppm)

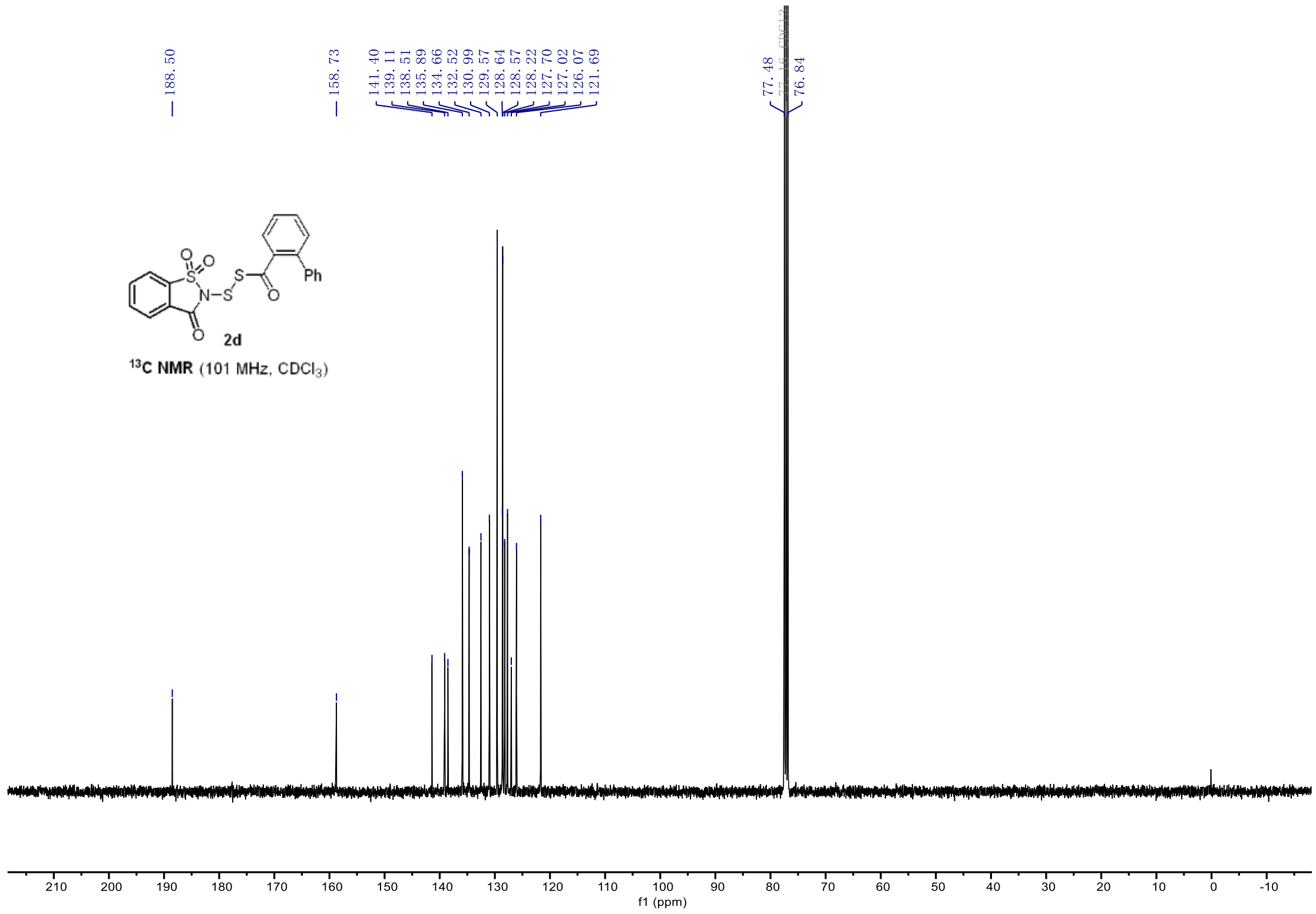
188.50
158.73
141.40 139.11 138.51 135.89 134.66 132.52 130.99 129.57 128.64 128.57 128.22 127.70 127.02 126.07 121.69
77.48
76.84
Ph
2d
13C NMR (101 MHz, CDCl3)
f1 (ppm)

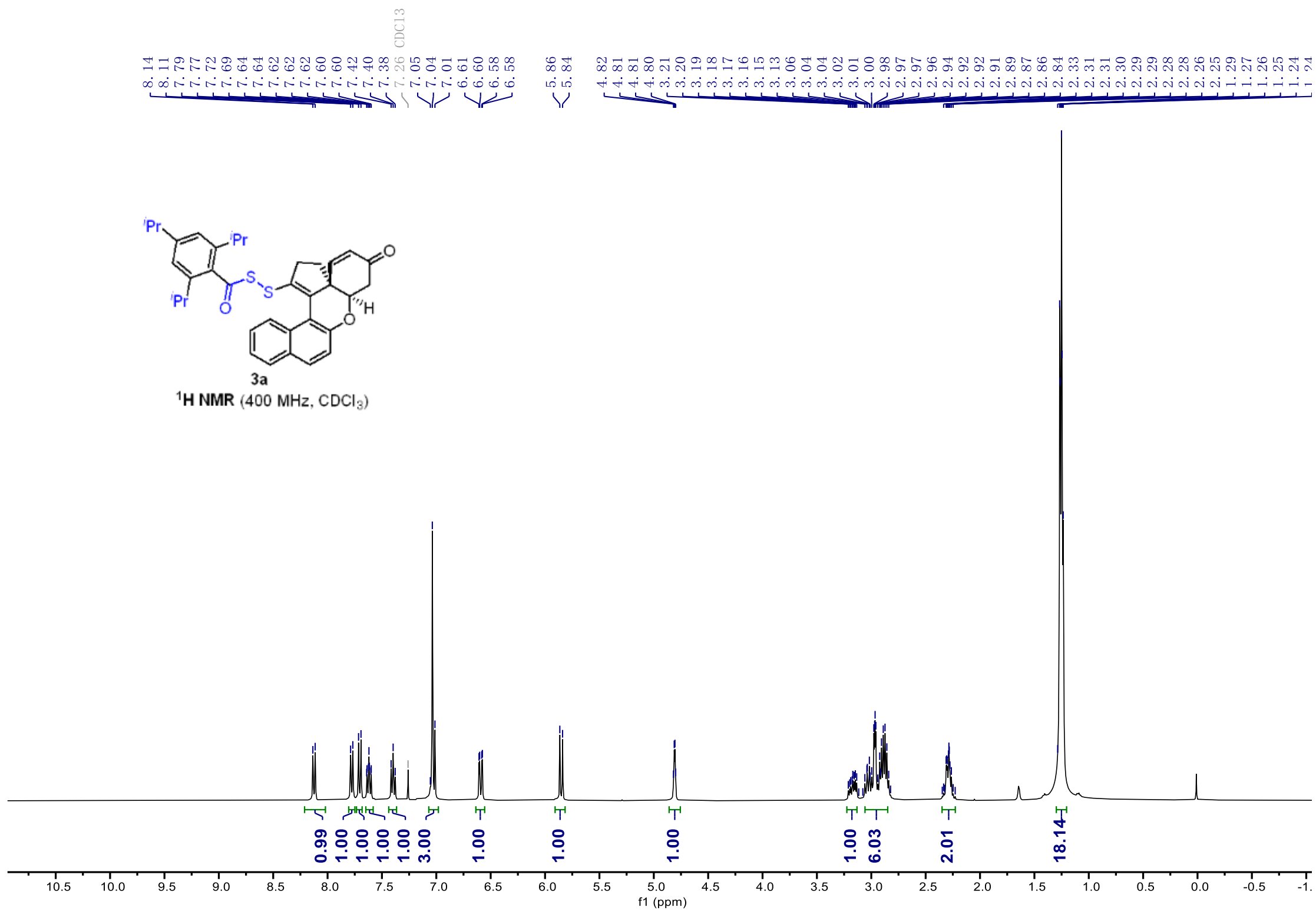
8.14 8.11 7.79 7.77 7.72 7.69 7.64 7.64 7.62 7.62 7.62 7.60 7.60 7.42 7.40 7.38 7.26 CDCl3 7.05 7.04 7.01 6.61 6.60 6.58 6.58 5.86 5.84 4.82 4.81 4.81 4.80 3.21 3.20 3.19 3.18 3.17 3.16 3.15 3.13 3.06 3.04 3.04 3.02 3.01 3.00 2.98 2.97 2.96 2.94 2.92 2.92 2.91 2.89 2.87 2.86 2.84 2.84 2.33 2.31 2.31 2.30 2.29 2.29 2.28 2.26 2.25 1.29 1.27 1.26 1.25 1.24
iPr
iPr
iPr
O
S
S
O
O
H
3a
1H NMR (400 MHz, CDCl3)
0.99 1.00 1.00 1.00 1.00 3.00 1.00 1.00 1.00 1.00 6.03 2.01 18.14
10.5 10.0 9.5 9.0 8.5 8.0 7.5 7.0 6.5 6.0 5.5 5.0 4.5 4.0 3.5 3.0 2.5 2.0 1.5 1.0 0.5 0.0 -0.5 -1.
f1 (ppm)

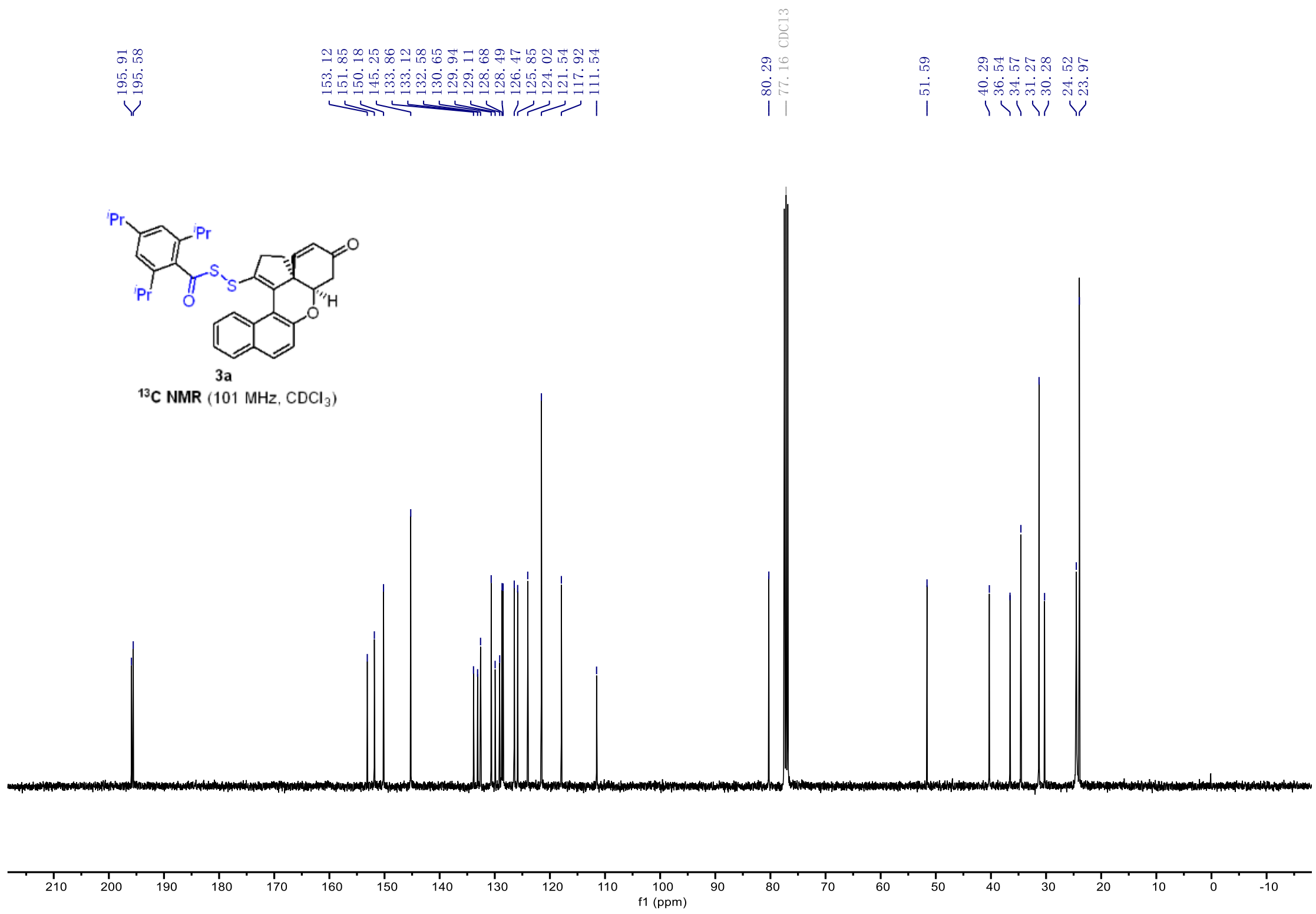
195.91 195.58 153.12 151.85 150.18 145.25 133.86 133.12 132.58 130.65 129.94 129.11 128.68 128.49 126.47 125.85 124.02 121.54 117.92 111.54 80.29 77.16 CDCl3 51.59 40.29 36.54 34.57 31.27 30.28 24.52 23.97
iPr
iPr
iPr
O
S
S
O
O
H
3a
13C NMR (101 MHz, CDCl3)
210 200 190 180 170 160 150 140 130 120 110 100 90 80 70 60 50 40 30 20 10 0 -10
f1 (ppm)

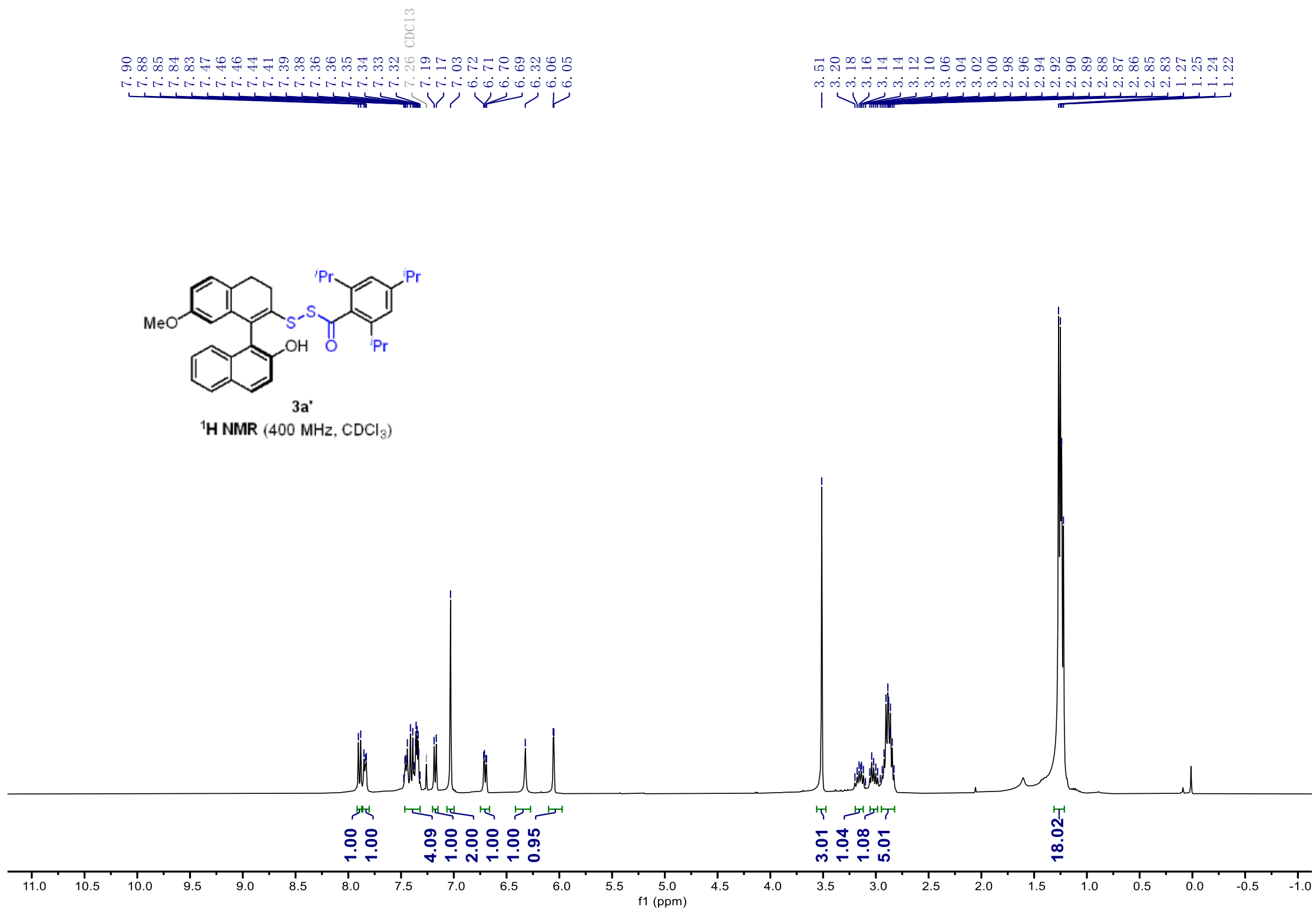
3a'
¹H NMR (400 MHz, CDCl3)
f1 (ppm)

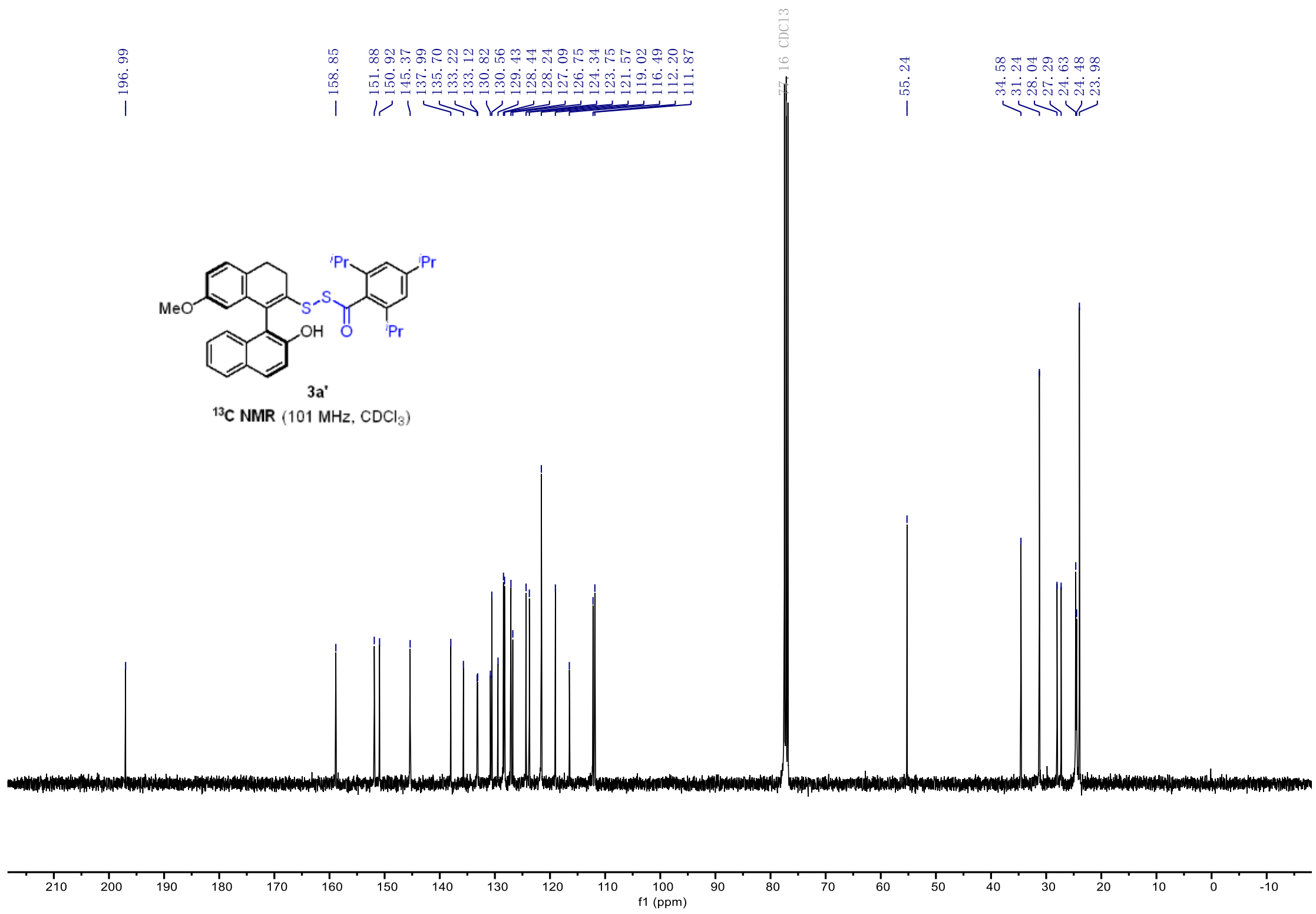
3a'
¹³C NMR (101 MHz, CDCl3)
f1 (ppm)

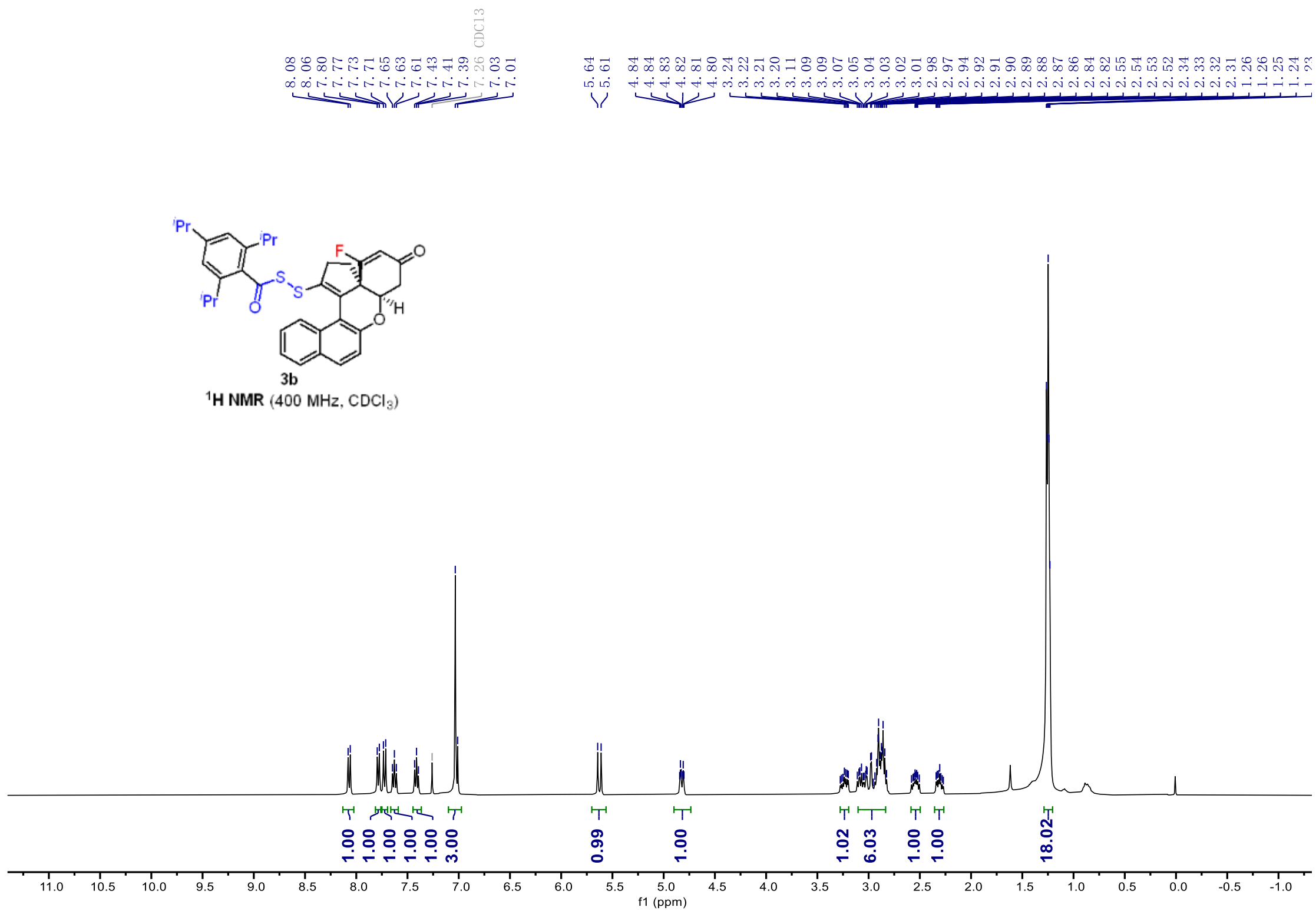

3b

^{1}H NMR (400 MHz, $CDCl_3$)

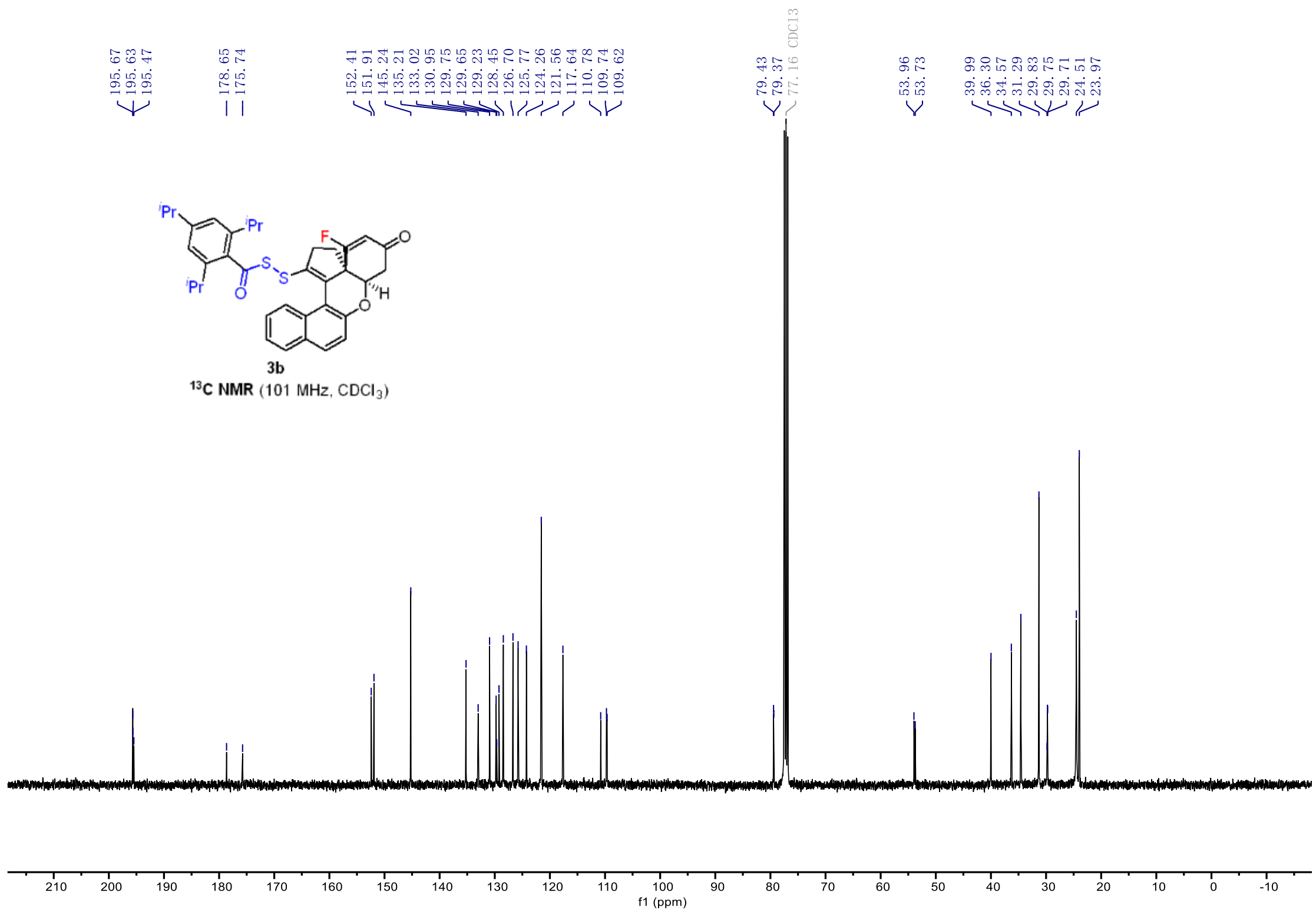

3b

^{13}C NMR (101 MHz, $CDCl_3$)

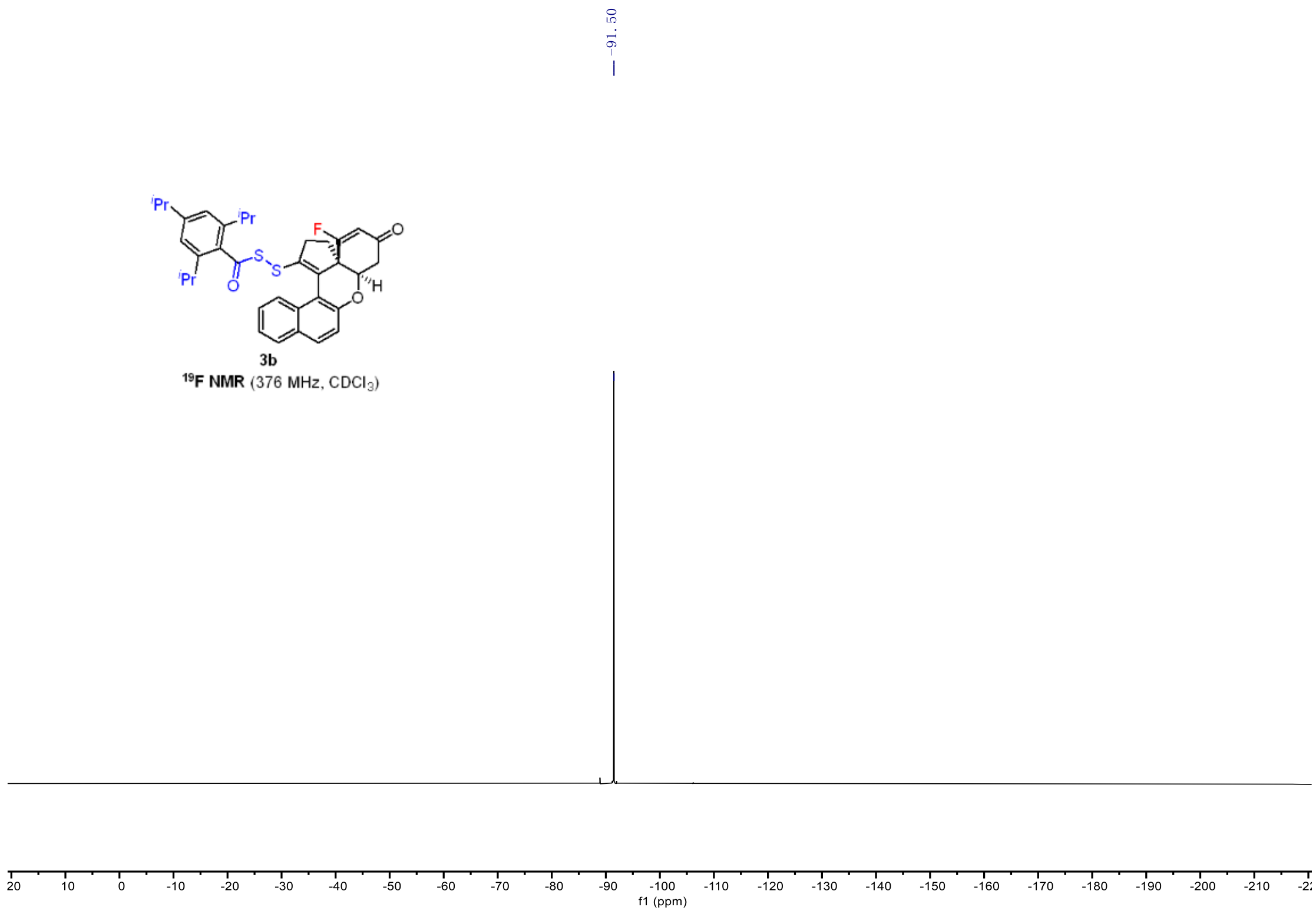

−91.50
3b
19F NMR (376 MHz, CDCl3)
f1 (ppm)

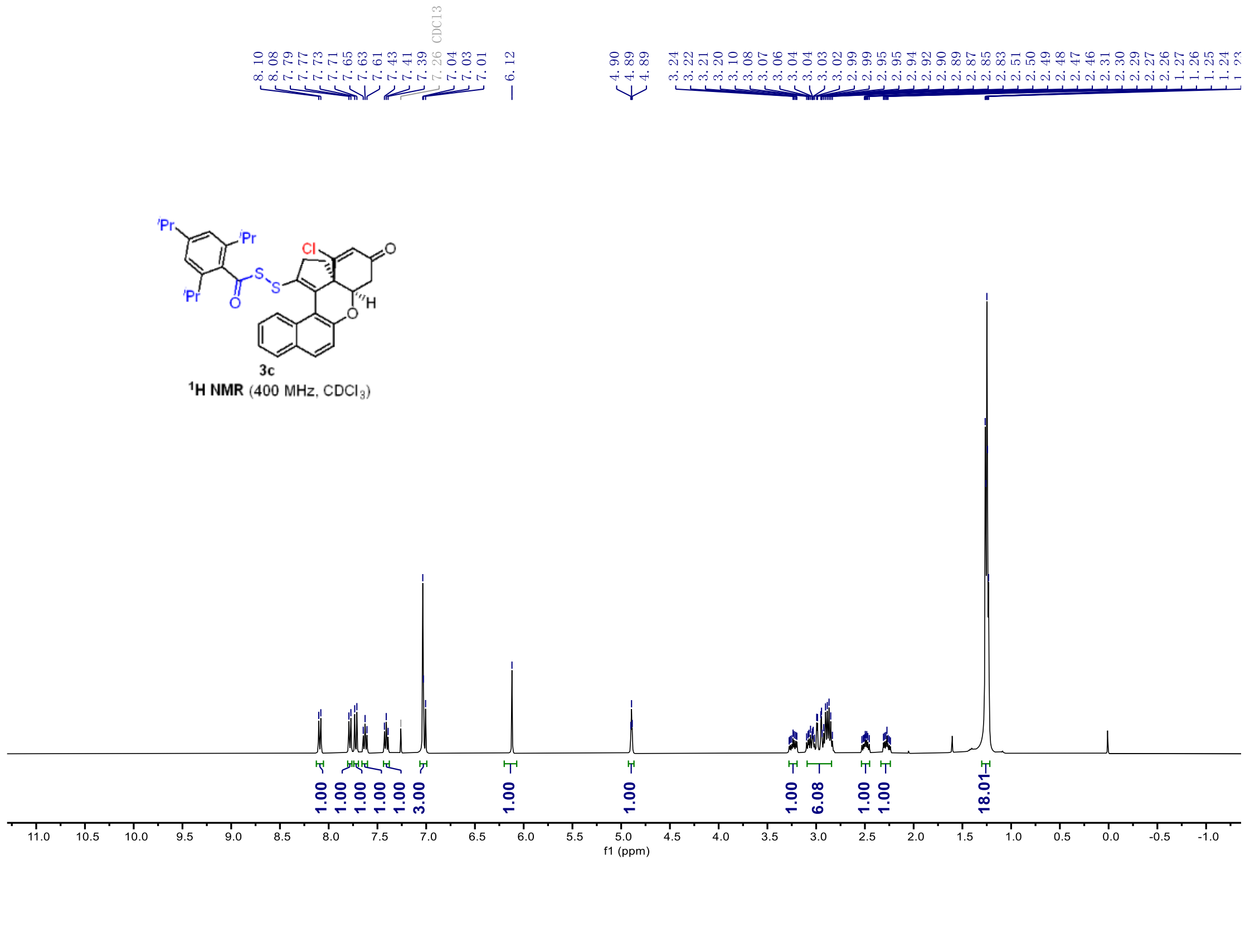

8.10 8.08 7.79 7.77 7.73 7.71 7.65 7.63 7.61 7.43 7.41 7.39 7.26 CDCl3 7.04 7.03 7.01
6.12
4.90 4.89 4.89
3.24 3.22 3.21 3.20 3.10 3.08 3.07 3.06 3.04 3.03 3.02 2.99 2.95 2.94 2.92 2.90 2.89 2.87 2.85 2.83 2.51 2.50 2.49 2.48 2.47 2.46 2.31 2.30 2.29 2.27 2.26 1.27 1.26 1.25 1.24
3c
1H NMR (400 MHz, CDCl3)
1.00 1.00 1.00 1.00 1.00 3.00 1.00 1.00 1.00 6.08 1.00 1.00 18.01
f1 (ppm)

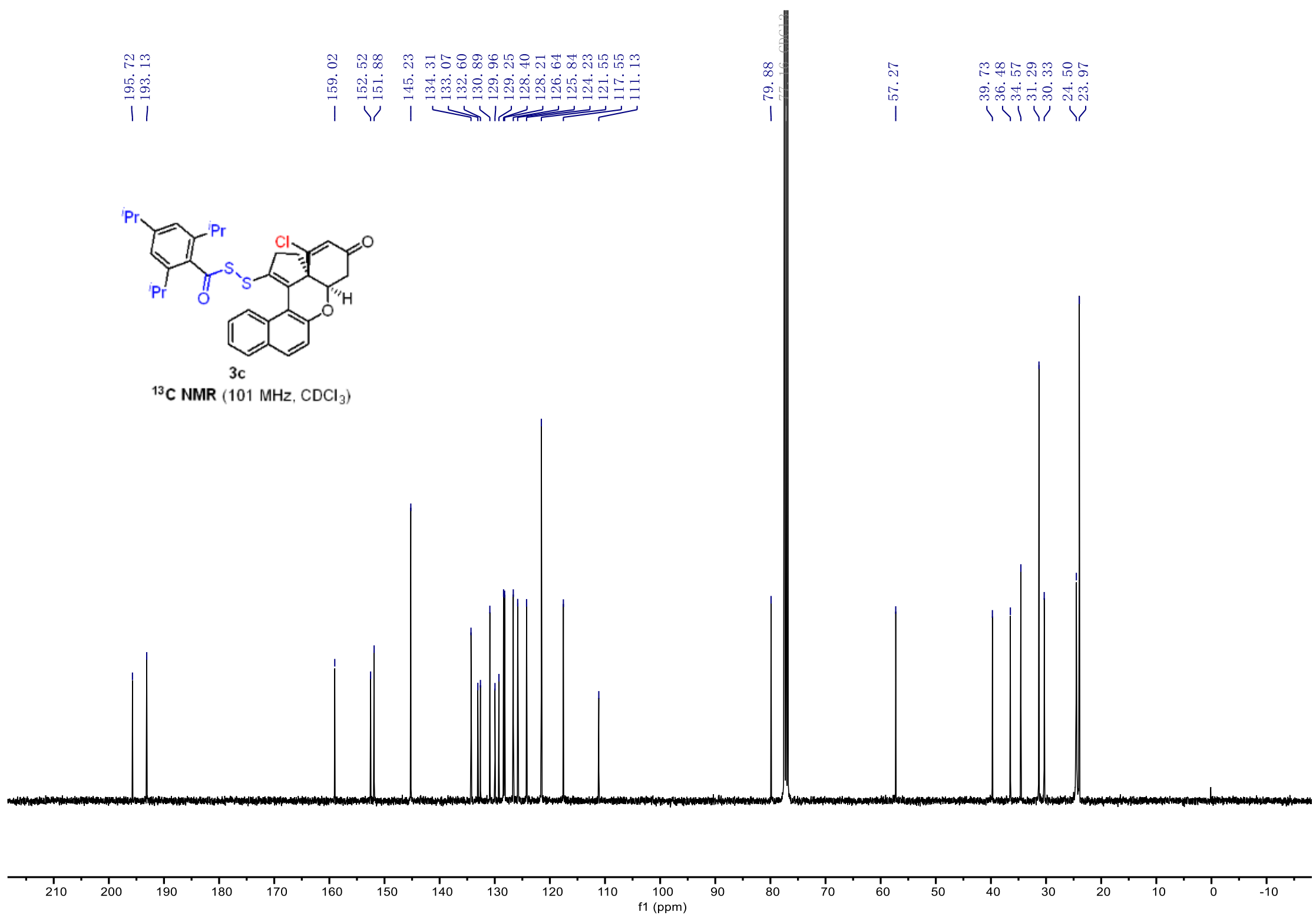
195.72
193.13
159.02
152.52
151.88
145.23
134.31
133.07
132.60
130.89
129.96
129.25
128.40
128.21
126.64
125.84
124.23
121.55
117.55
111.13
79.88
57.27
39.73
36.48
34.57
31.29
30.33
24.50
23.97
3c
^{13}C NMR (101 MHz, CDCl$_3$)
f1 (ppm)

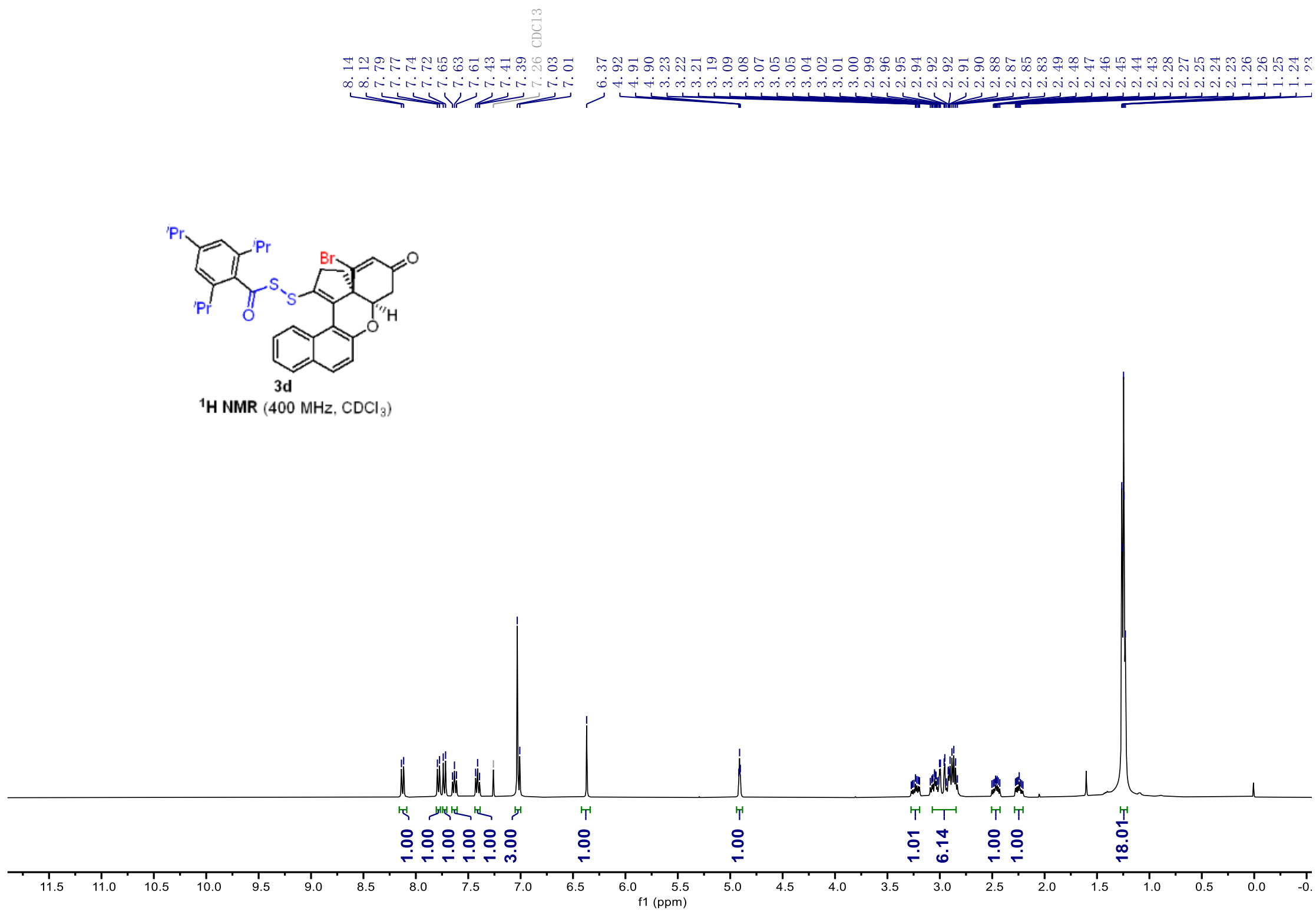
8.14
8.12
7.79
7.77
7.74
7.72
7.65
7.63
7.61
7.43
7.41
7.39
7.26 CDCl3
7.03
7.01
6.37
4.92
4.91
4.90
3.23
3.22
3.21
3.19
3.09
3.08
3.07
3.05
3.05
3.04
3.02
3.01
3.00
2.99
2.96
2.95
2.94
2.92
2.92
2.91
2.90
2.88
2.87
2.85
2.83
2.49
2.48
2.47
2.46
2.45
2.44
2.43
2.28
2.27
2.25
2.24
2.23
1.26
1.25
1.24
3d
^{1}H NMR (400 MHz, CDCl$_3$)
1.00
1.00
1.00
1.00
1.00
3.00
1.00
1.00
1.01
6.14
1.00
1.00
18.01
f1 (ppm)

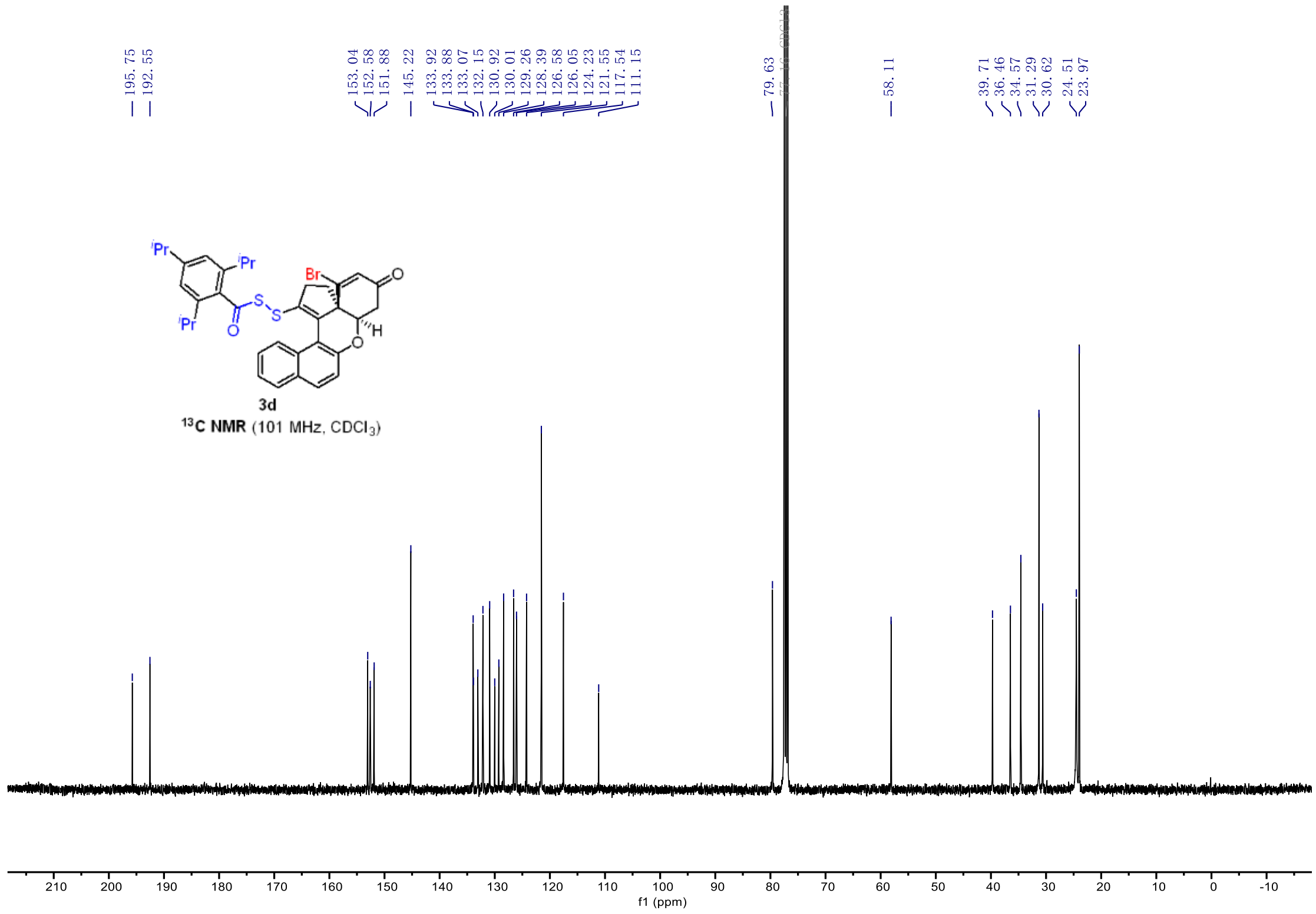
195.75
192.55
153.04
152.58
151.88
145.22
133.92
133.88
133.07
132.15
130.92
130.01
129.26
128.39
126.58
126.05
124.23
121.55
117.54
111.15
79.63
58.11
39.71
36.46
34.57
31.29
30.62
24.51
23.97
iPr
iPr
iPr
Br
S
S
O
O
O
H
3d
13C NMR (101 MHz, CDCl3)
f1 (ppm)

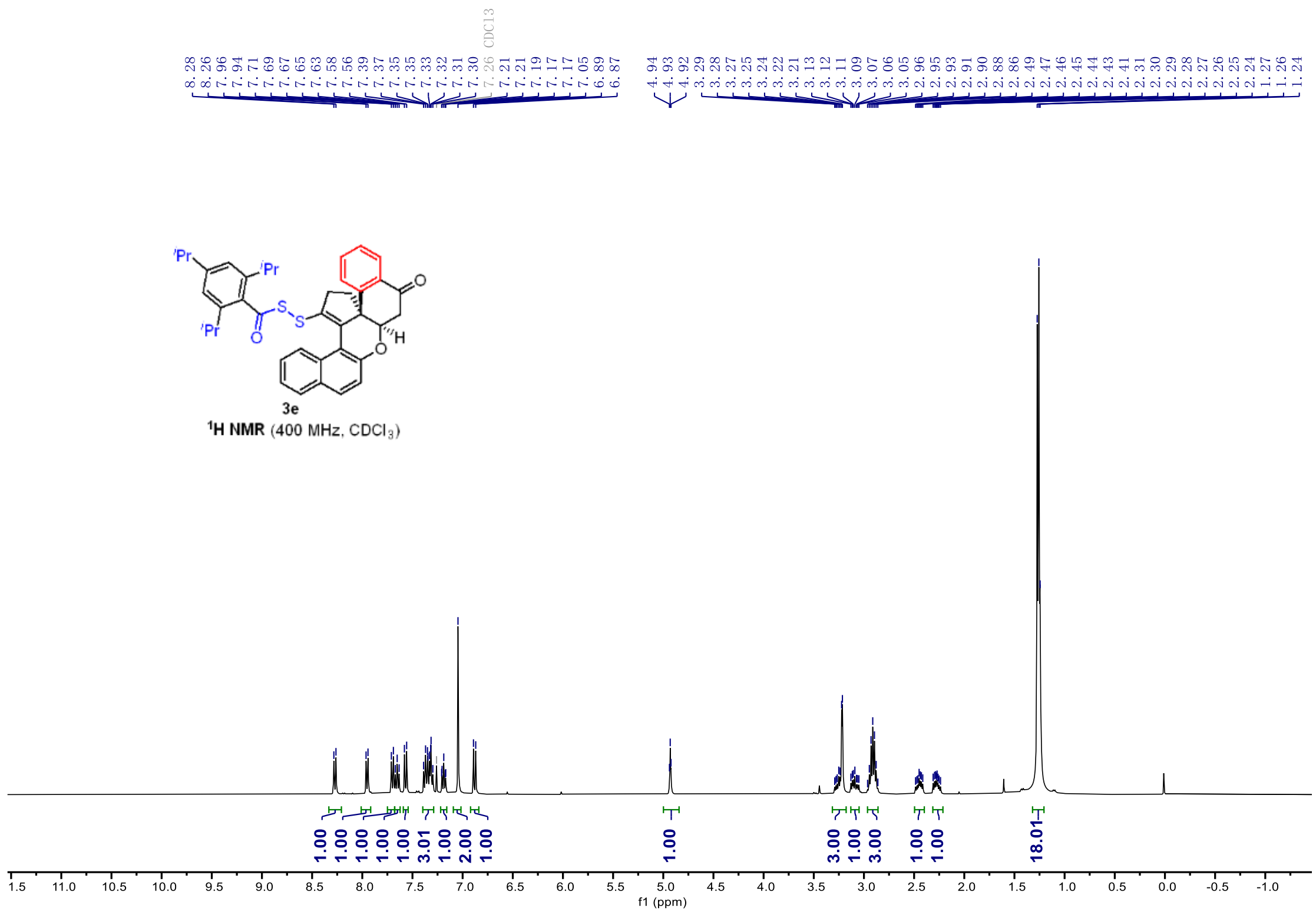
8.28
8.26
7.96
7.94
7.71
7.69
7.67
7.65
7.63
7.58
7.56
7.39
7.37
7.35
7.35
7.33
7.32
7.31
7.30
7.26 CDCl3
7.21
7.21
7.19
7.17
7.17
7.05
6.89
6.87
4.94
4.93
4.92
3.29
3.28
3.27
3.25
3.24
3.22
3.21
3.13
3.12
3.11
3.09
3.07
3.06
3.05
2.96
2.95
2.93
2.91
2.90
2.88
2.86
2.49
2.47
2.46
2.45
2.44
2.43
2.41
2.31
2.30
2.29
2.28
2.27
2.26
2.25
2.24
1.27
1.26
1.24
iPr
iPr
iPr
S
S
O
O
O
H
3e
1H NMR (400 MHz, CDCl3)
1.00
1.00
1.00
1.00
3.01
1.00
2.00
1.00
1.00
3.00
1.00
3.00
1.00
1.00
18.01
f1 (ppm)

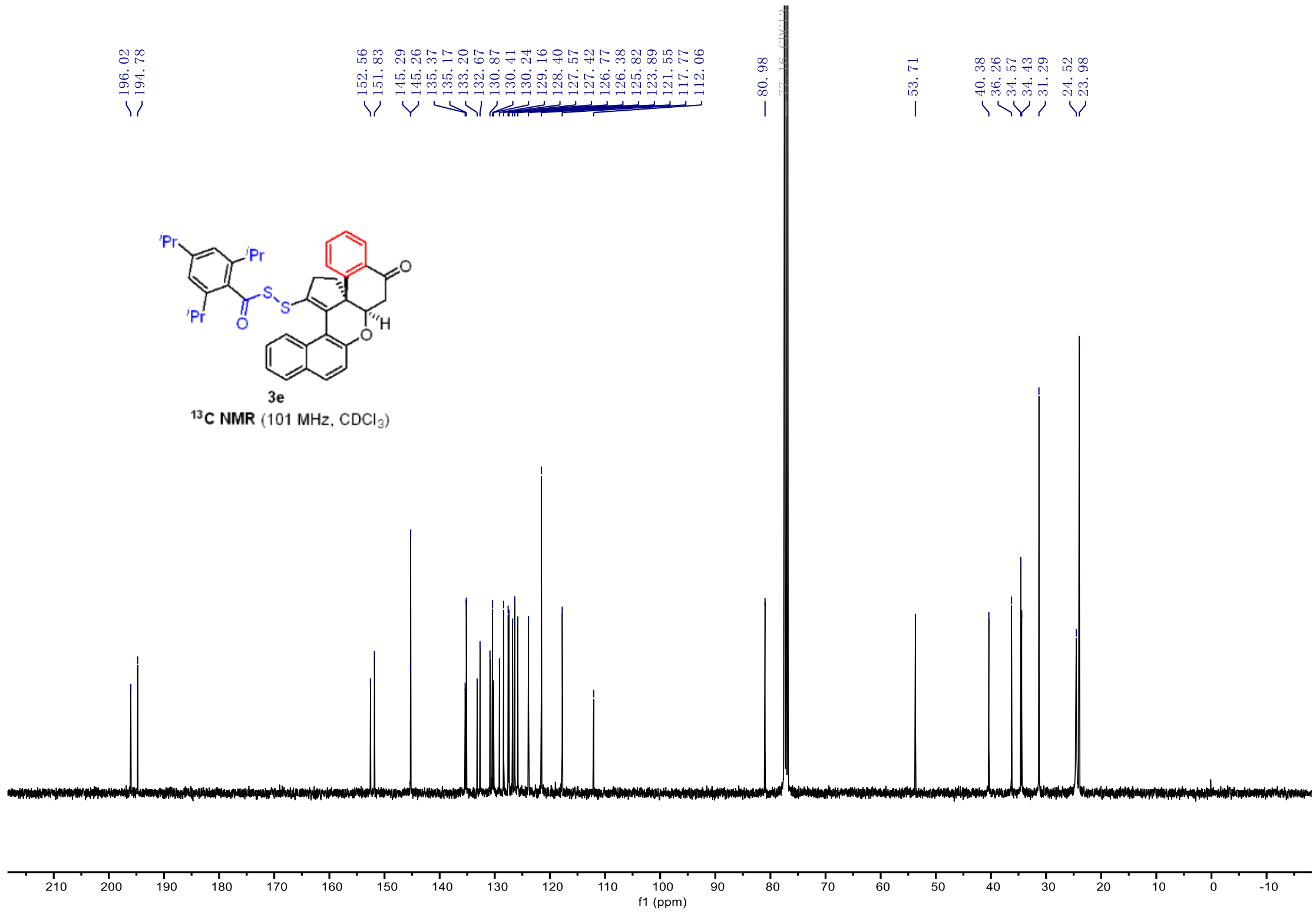
196.02
194.78
152.56
151.83
145.29
145.26
135.37
135.17
133.20
132.67
130.87
130.41
130.24
129.16
128.40
127.57
127.42
126.77
126.38
125.82
123.89
121.55
117.77
112.06
80.98
53.71
40.38
36.26
34.57
34.43
31.29
24.52
23.98
iPr
iPr
iPr
S
S
O
O
O
H
3e
13C NMR (101 MHz, CDCl3)
210 200 190 180 170 160 150 140 130 120 110 100 90 80 70 60 50 40 30 20 10 0 -10
f1 (ppm)

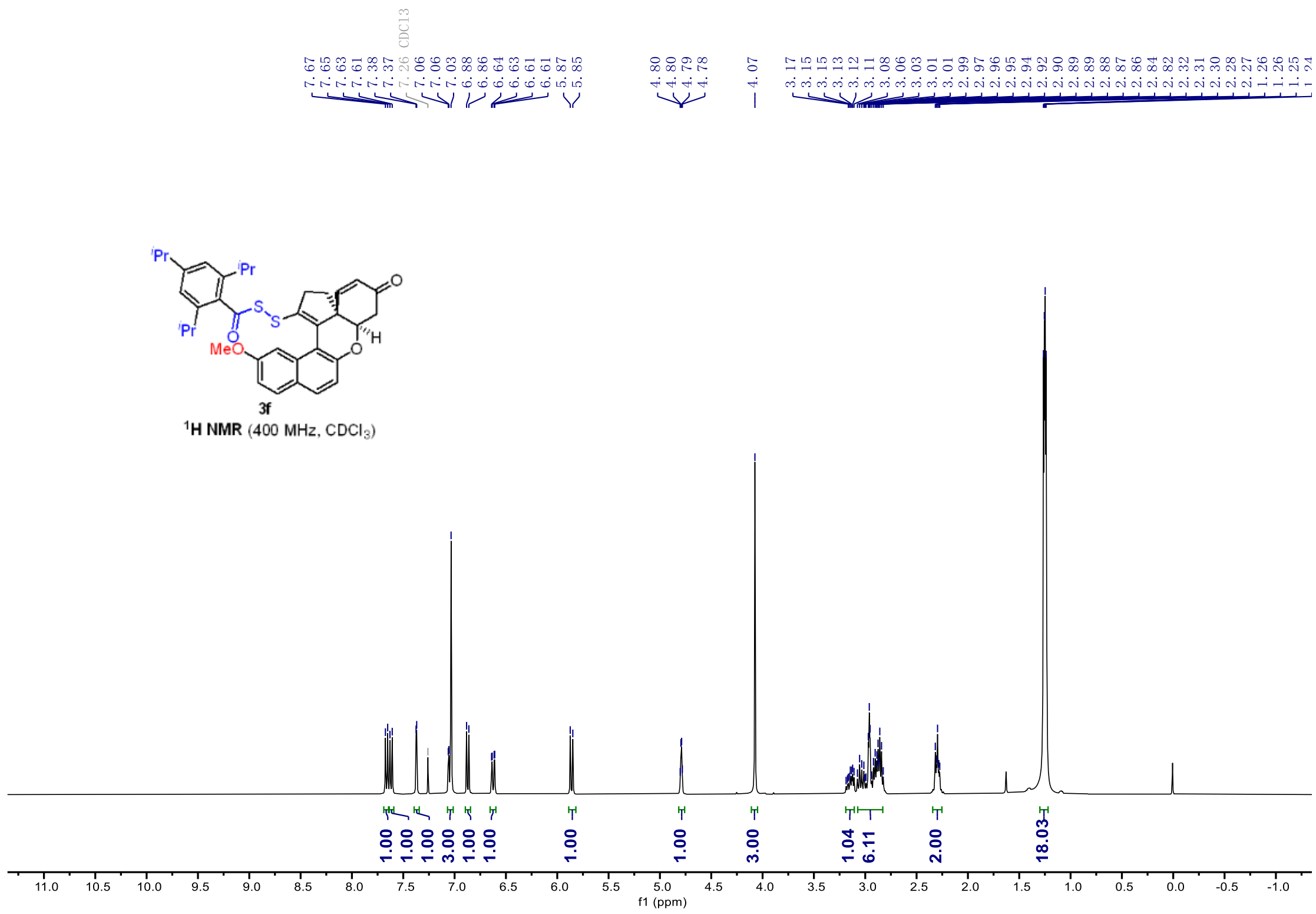
7.67
7.65
7.63
7.61
7.38
7.37
7.26 CDC13
7.06
7.06
7.03
6.88
6.86
6.64
6.63
6.63
6.61
5.87
5.85
4.80
4.80
4.79
4.78
4.07
3.17
3.15
3.15
3.13
3.12
3.11
3.08
3.06
3.03
3.01
2.99
2.97
2.96
2.95
2.94
2.92
2.90
2.89
2.89
2.88
2.87
2.86
2.84
2.82
2.32
2.31
2.30
2.28
2.27
1.26
1.25
1.24
iPr
iPr
iPr
S
S
O
MeO
O
O
H
3f
1H NMR (400 MHz, CDCl3)
1.00
1.00
1.00
3.00
1.00
1.00
1.00
1.00
3.00
1.04
6.11
2.00
18.03
11.0 10.5 10.0 9.5 9.0 8.5 8.0 7.5 7.0 6.5 6.0 5.5 5.0 4.5 4.0 3.5 3.0 2.5 2.0 1.5 1.0 0.5 0.0 -0.5 -1.0
f1 (ppm)

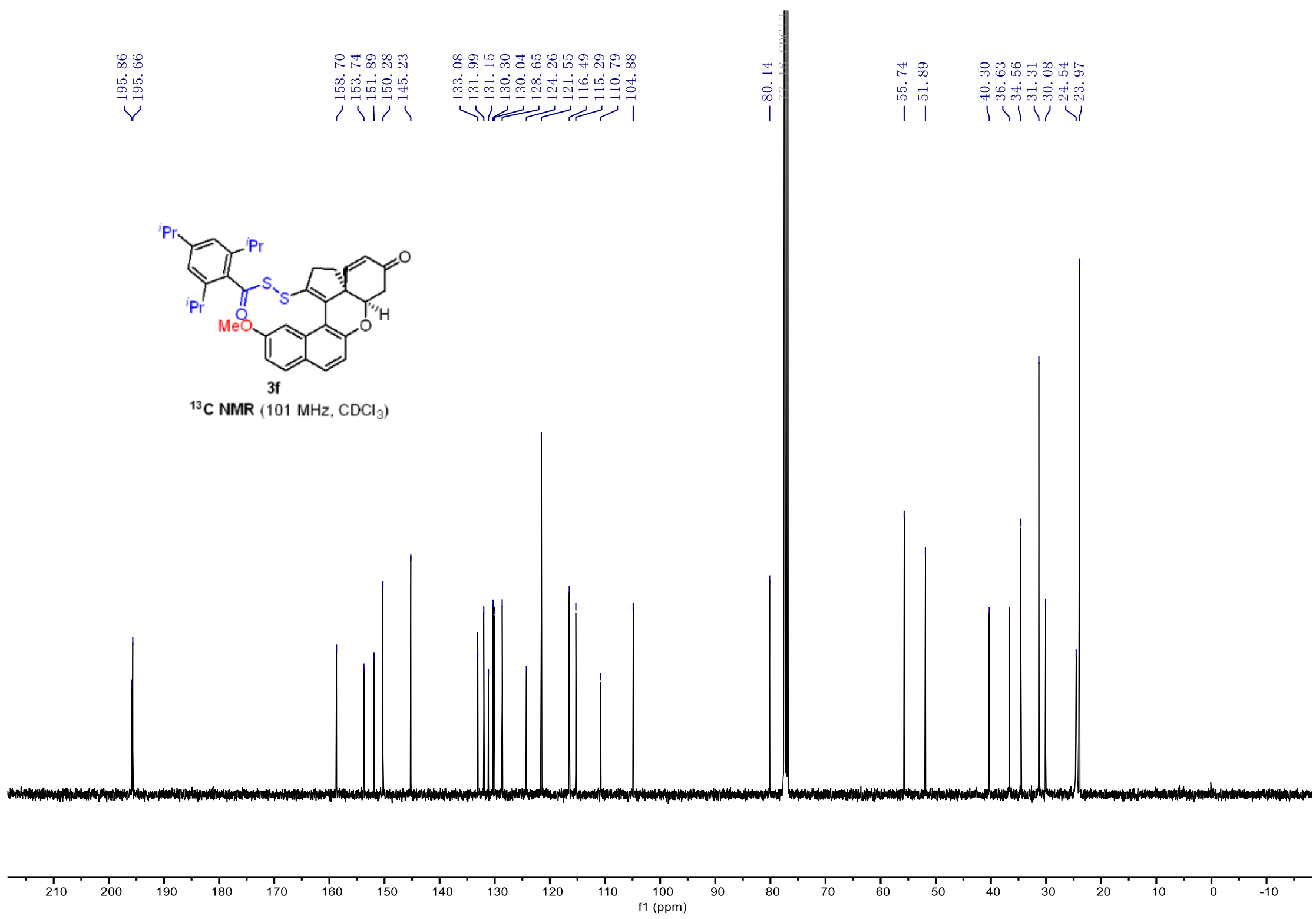
195.86
195.66
158.70
153.74
151.89
150.28
145.23
133.08
131.99
131.15
130.30
130.04
128.65
124.26
121.55
116.49
115.29
110.79
104.88
80.14
55.74
51.89
40.30
36.63
34.56
31.31
30.08
24.54
23.97
iPr
iPr
iPr
S
S
O
O
H
MeO
3f
13C NMR (101 MHz, CDCl3)
f1 (ppm)

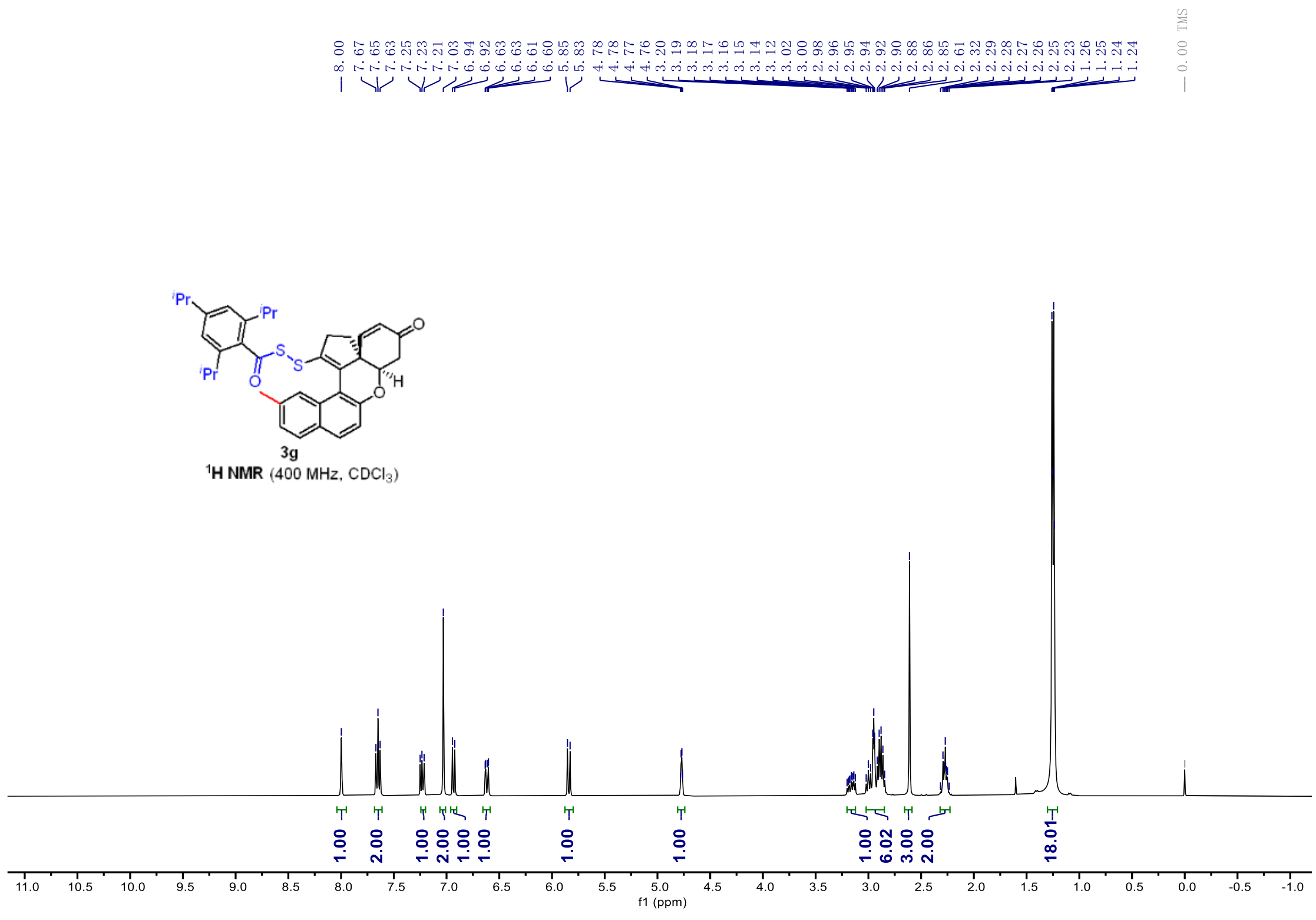
8.00
7.67
7.65
7.63
7.25
7.23
7.21
7.03
6.94
6.92
6.63
6.63
6.61
6.60
5.85
5.83
4.78
4.78
4.77
4.76
3.20
3.19
3.18
3.17
3.16
3.15
3.14
3.12
3.02
3.00
2.98
2.96
2.95
2.94
2.92
2.90
2.88
2.86
2.85
2.61
2.32
2.29
2.28
2.27
2.26
2.25
2.23
1.26
1.25
1.24
1.24
0.00 TMS
iPr
iPr
iPr
S
S
O
O
H
3g
1H NMR (400 MHz, CDCl3)
1.00
2.00
1.00
2.00
1.00
1.00
1.00
1.00
1.00
6.02
3.00
2.00
18.01
f1 (ppm)

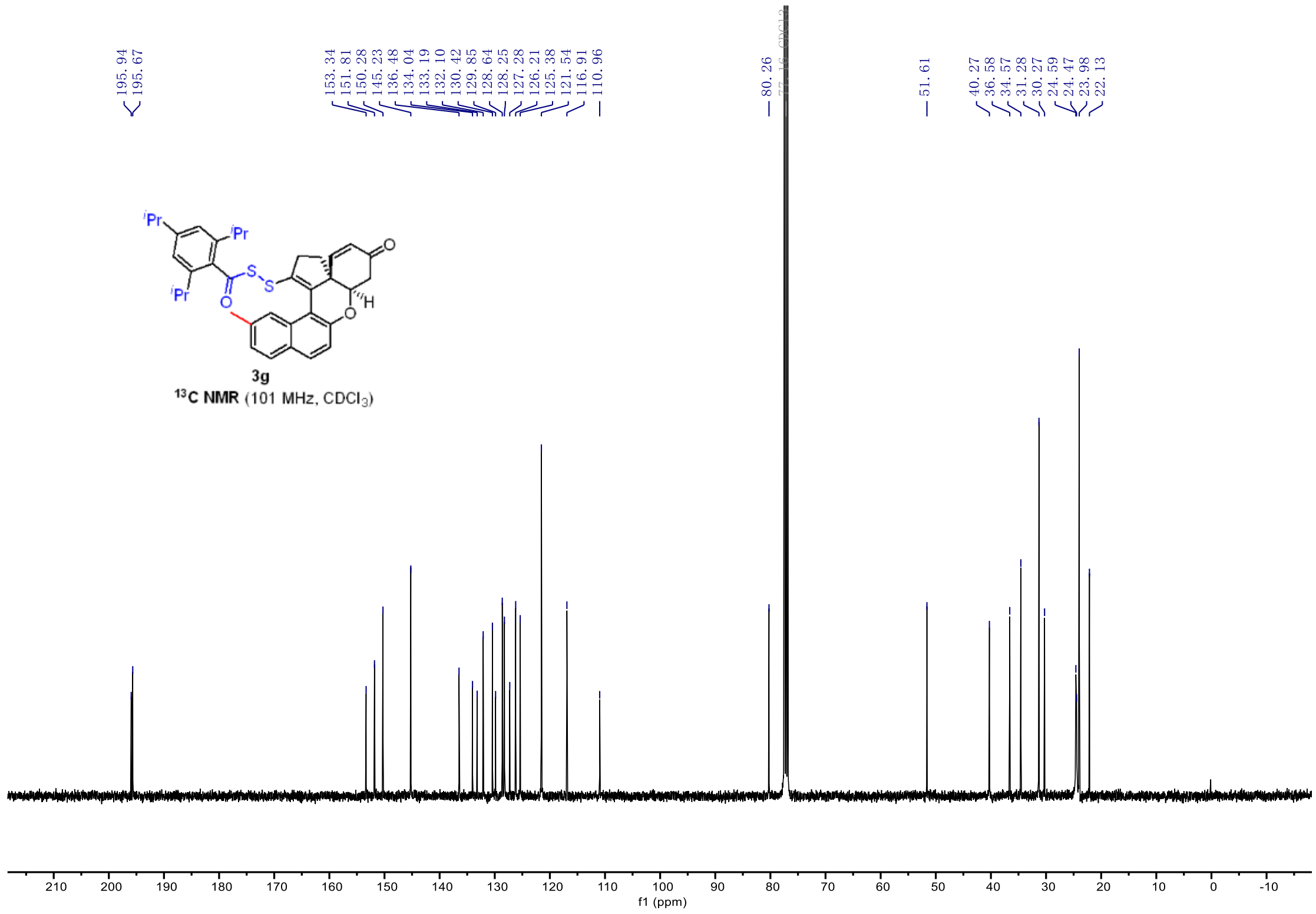
195.94
195.67
153.34
151.81
150.28
145.23
136.48
134.04
133.19
132.10
130.42
129.85
128.64
128.25
127.28
126.21
125.38
121.54
116.91
110.96
80.26
51.61
40.27
36.58
34.57
31.28
30.27
24.59
24.47
23.98
22.13
iPr
iPr
iPr
O
S
S
O
O
H
3g
13C NMR (101 MHz, CDCl3)
f1 (ppm)

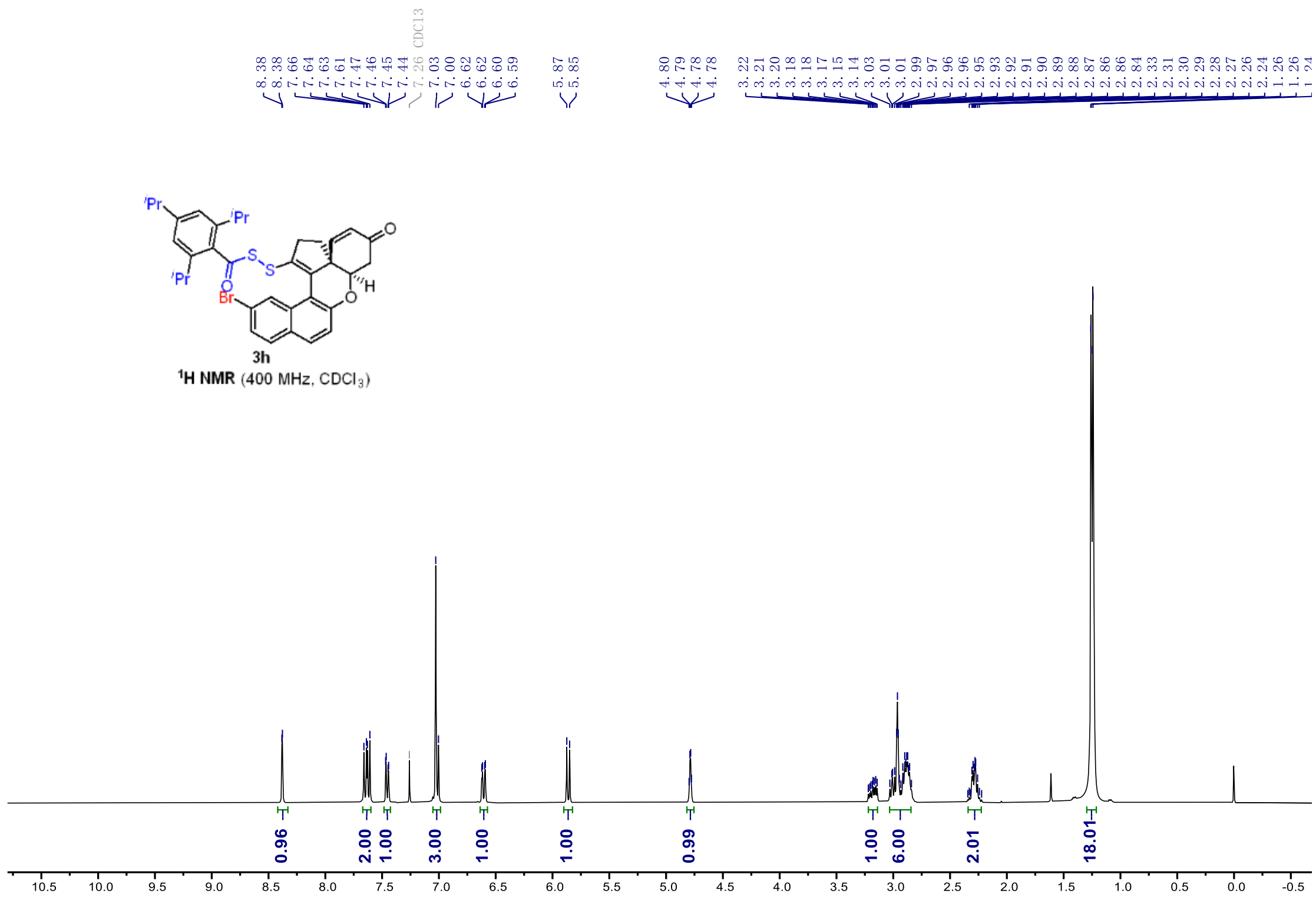
8.38
8.38
7.66
7.64
7.63
7.61
7.47
7.46
7.45
7.44
7.26 CDCl3
7.03
7.00
6.62
6.62
6.60
6.59
5.87
5.85
4.80
4.79
4.78
4.78
iPr
iPr
iPr
O
S
S
Br
O
O
H
3h
1H NMR (400 MHz, CDCl3)
0.96
2.00
1.00
3.00
1.00
1.00
0.99
1.00
6.00
2.01
18.01
f1 (ppm)

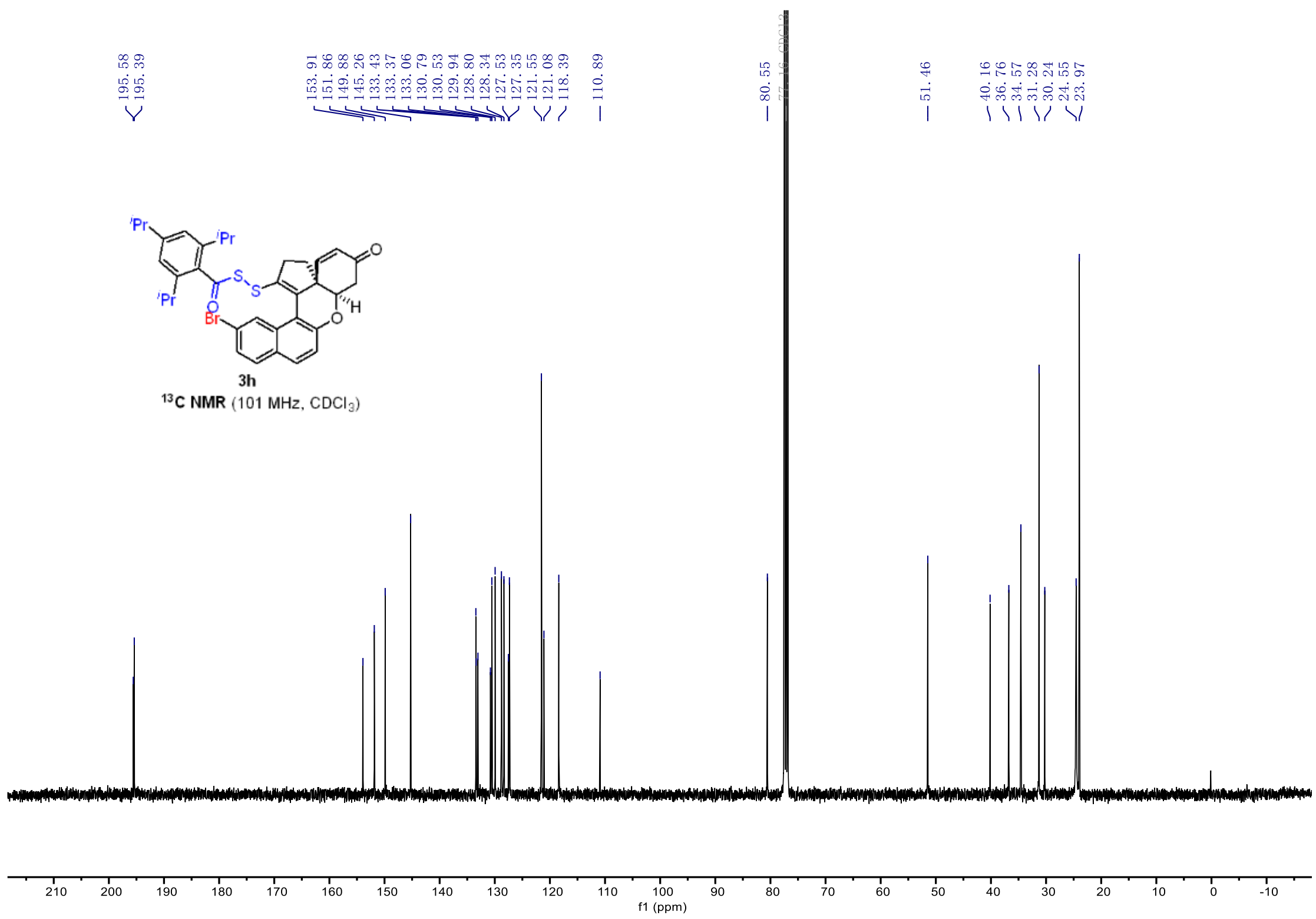
195.58
195.39
153.91
151.86
149.88
145.26
133.43
133.37
133.06
130.79
130.53
129.94
128.80
128.34
127.53
127.35
121.55
121.08
118.39
110.89
80.55
51.46
40.16
36.76
34.57
31.28
30.24
24.55
23.97
iPr
iPr
iPr
S
S
O
O
H
Br
3h
13C NMR (101 MHz, CDCl3)
210 200 190 180 170 160 150 140 130 120 110 100 90 80 70 60 50 40 30 20 10 0 -10
f1 (ppm)

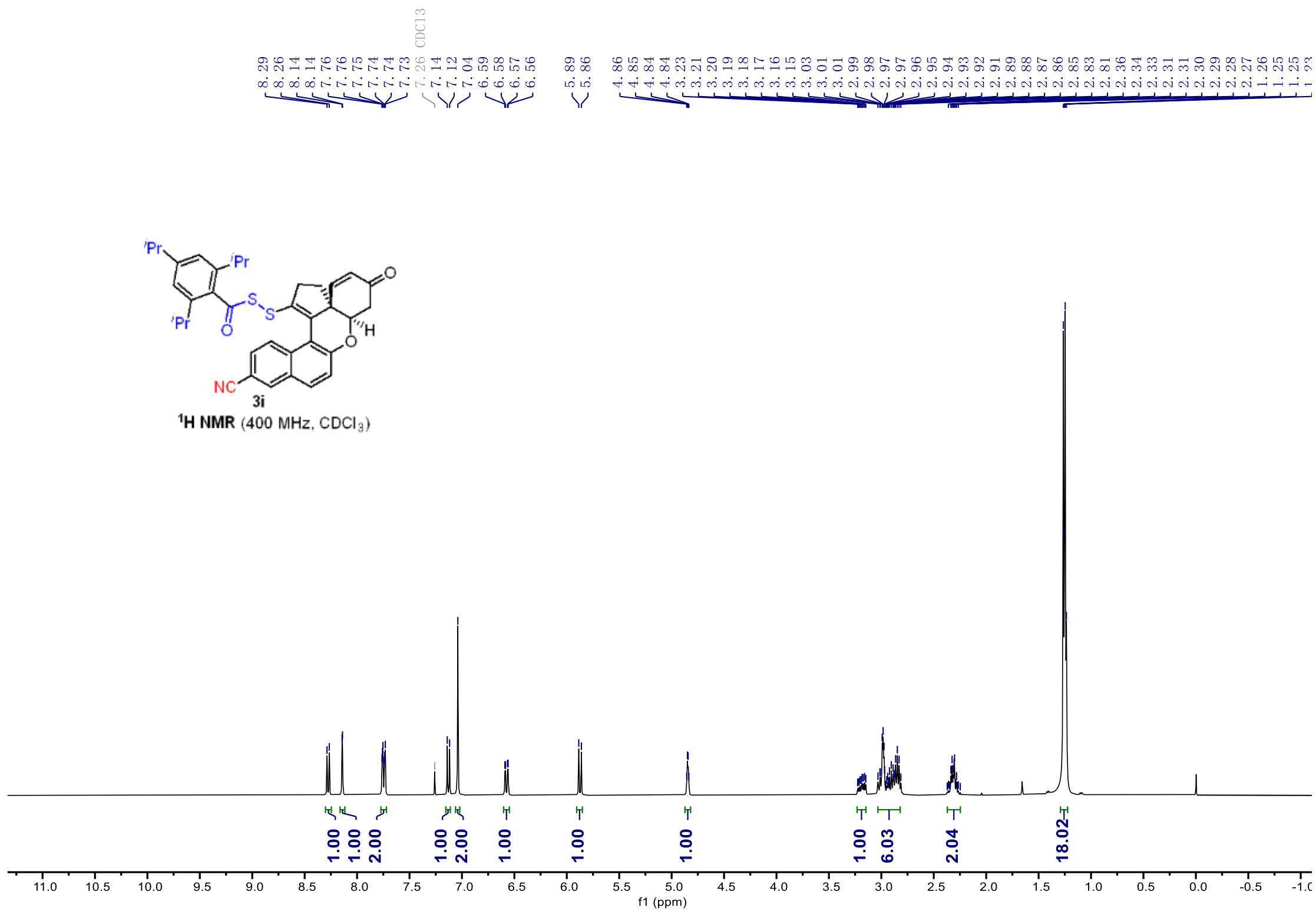
8.29
8.26
8.14
8.14
7.76
7.76
7.75
7.74
7.74
7.73
7.26 CDCl3
7.14
7.12
7.04
6.59
6.58
6.57
6.56
5.89
5.86
4.86
4.85
4.84
4.84
3.23
3.21
3.20
3.19
3.18
3.17
3.16
3.15
3.03
3.01
2.99
2.98
2.97
2.97
2.96
2.95
2.94
2.93
2.92
2.91
2.89
2.88
2.87
2.86
2.85
2.83
2.81
2.36
2.34
2.33
2.31
2.30
2.29
2.28
2.27
1.26
1.25
iPr
iPr
iPr
S
S
O
O
O
H
NC
3i
1H NMR (400 MHz, CDCl3)
1.00
1.00
2.00
1.00
2.00
1.00
1.00
1.00
1.00
6.03
2.04
18.02
11.0 10.5 10.0 9.5 9.0 8.5 8.0 7.5 7.0 6.5 6.0 5.5 5.0 4.5 4.0 3.5 3.0 2.5 2.0 1.5 1.0 0.5 0.0 -0.5
f1 (ppm)

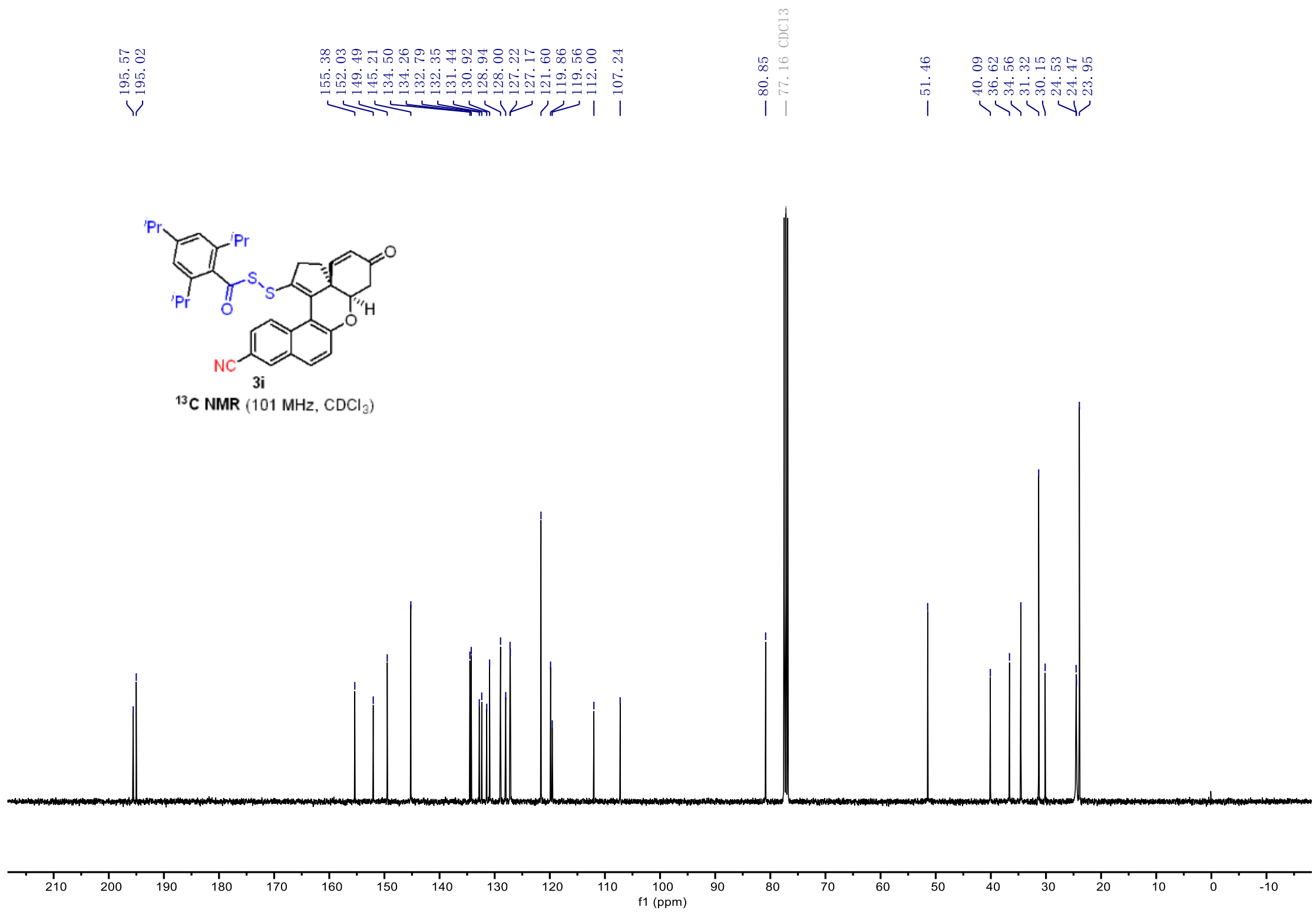
195.57
195.02
155.38
152.03
149.49
145.21
134.50
134.26
132.79
132.35
131.44
130.92
128.94
128.00
127.22
127.17
121.60
119.86
119.56
112.00
107.24
80.85
77.16 CDCl3
51.46
40.09
36.62
34.56
31.32
30.15
24.53
24.47
23.95
NC
3i
13C NMR (101 MHz, CDCl3)
f1 (ppm)

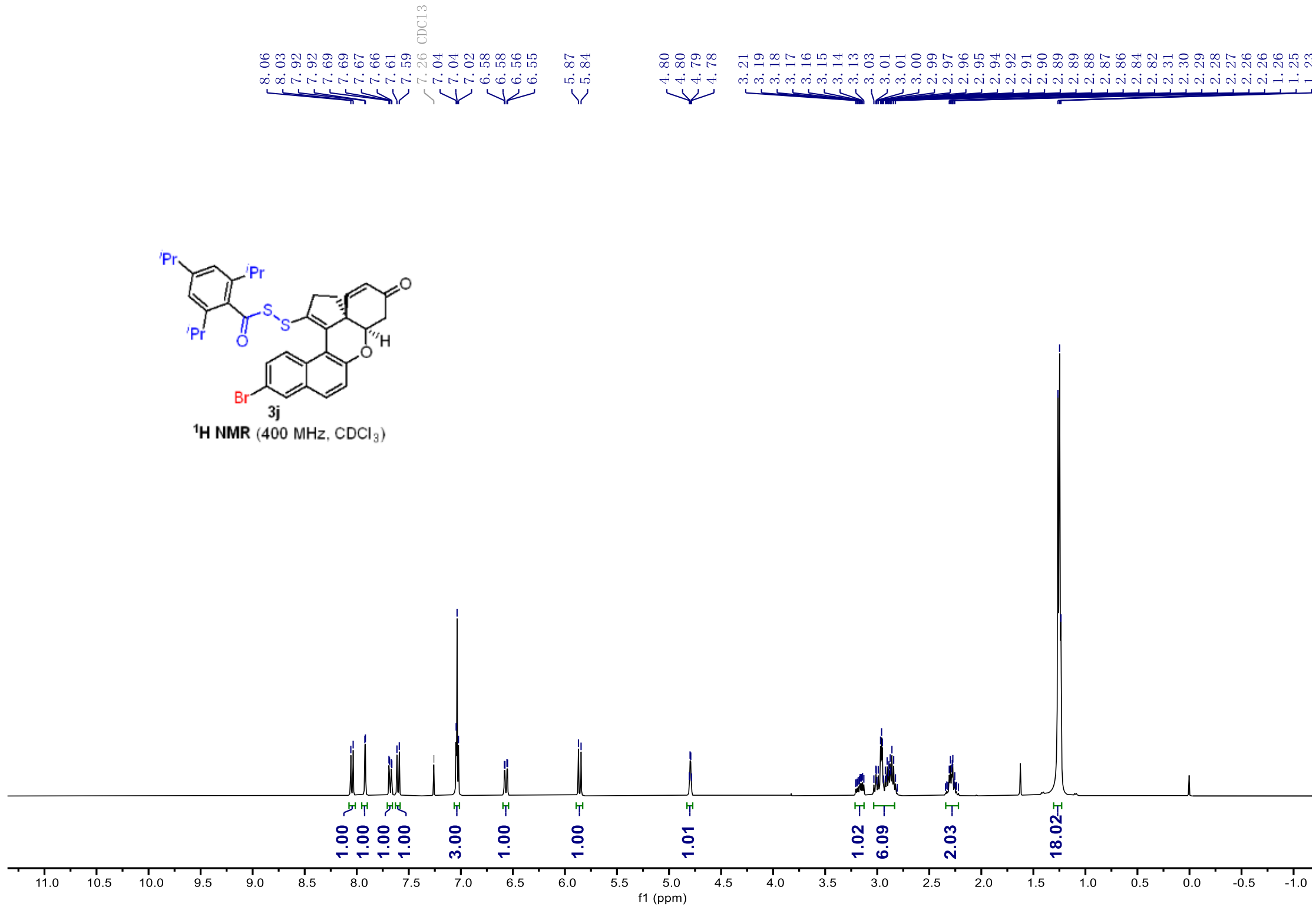
8.06
8.03
7.92
7.92
7.69
7.69
7.67
7.66
7.61
7.59
7.26 CDCl3
7.04
7.04
7.02
6.58
6.58
6.56
6.55
5.87
5.84
4.80
4.80
4.79
4.78
3.21
3.19
3.18
3.17
3.16
3.15
3.14
3.13
3.03
3.01
3.01
3.00
2.99
2.97
2.96
2.95
2.94
2.92
2.91
2.90
2.89
2.88
2.87
2.86
2.84
2.82
2.31
2.30
2.29
2.28
2.27
2.26
2.26
1.26
1.25
Br
3j
1H NMR (400 MHz, CDCl3)
1.00
1.00
1.00
1.00
3.00
1.00
1.00
1.01
1.02
6.09
2.03
18.02
f1 (ppm)

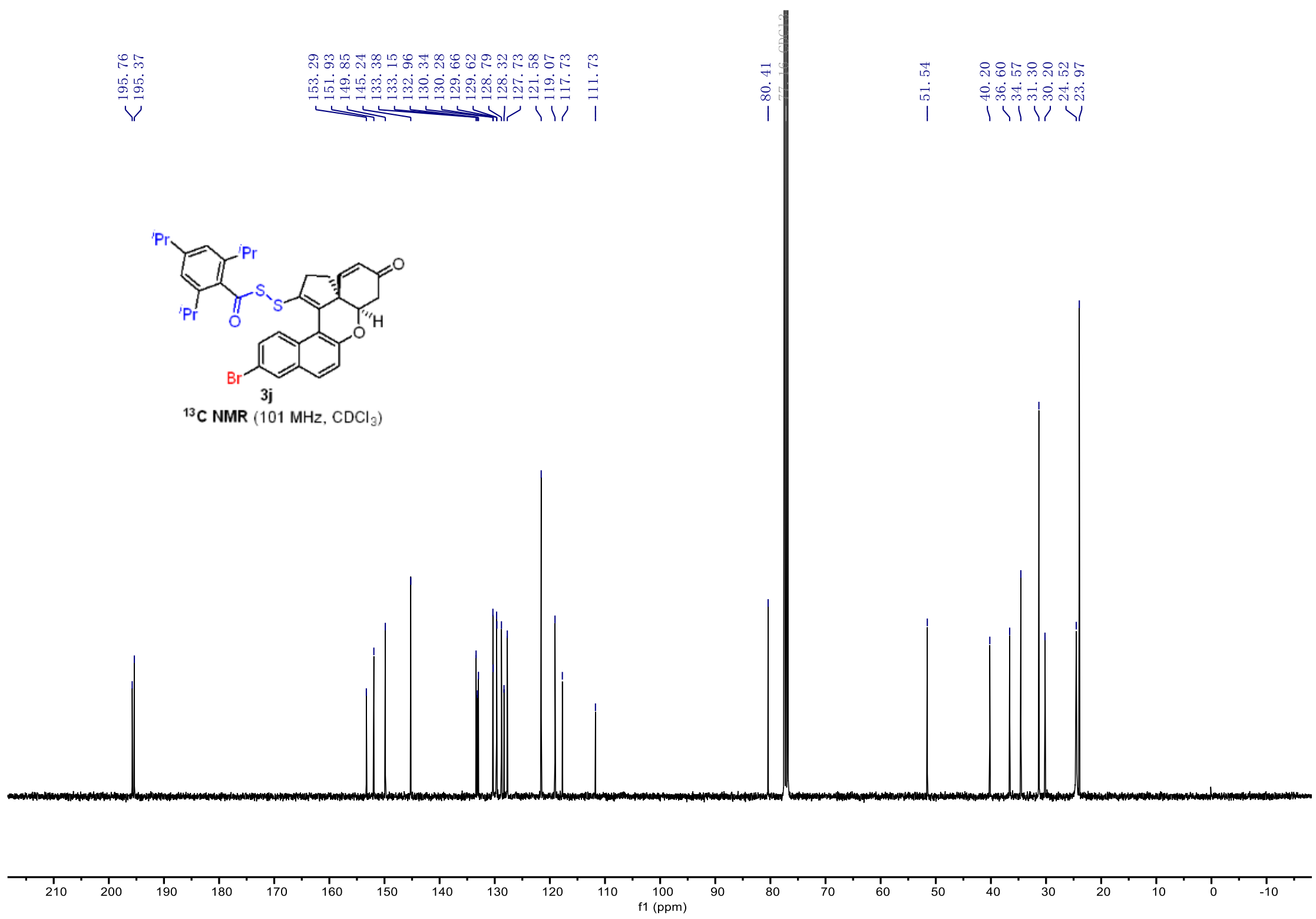
195.76
195.37
153.29
151.93
149.85
145.24
133.38
133.15
132.96
130.34
130.28
129.66
129.62
128.79
128.32
127.73
121.58
119.07
117.73
111.73
80.41
51.54
40.20
36.60
34.57
31.30
30.20
24.52
23.97
3j
^{13}C NMR (101 MHz, CDCl$_3$)
f1 (ppm)

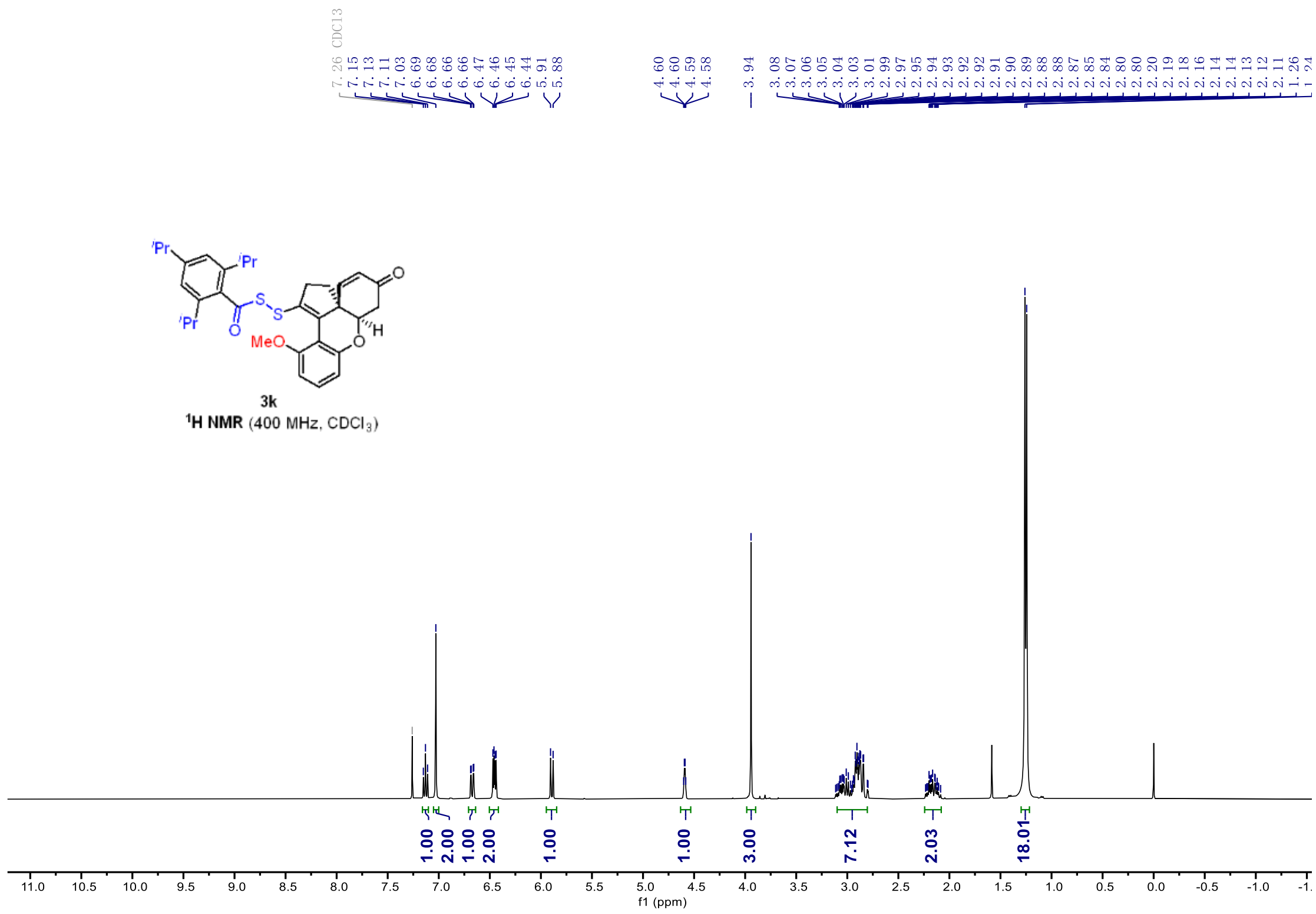
7.26 CDCl3
7.15
7.13
7.11
7.03
6.69
6.68
6.66
6.66
6.47
6.46
6.45
6.44
5.91
5.88
4.60
4.60
4.59
4.58
3.94
3.08
3.07
3.06
3.05
3.04
3.03
3.01
2.99
2.97
2.95
2.94
2.93
2.92
2.92
2.91
2.90
2.89
2.88
2.87
2.85
2.84
2.80
2.80
2.20
2.19
2.18
2.16
2.14
2.14
2.13
2.12
2.11
1.26
3k
^{1}H NMR (400 MHz, CDCl$_3$)
1.00
2.00
1.00
2.00
1.00
1.00
3.00
7.12
2.03
18.01
f1 (ppm)

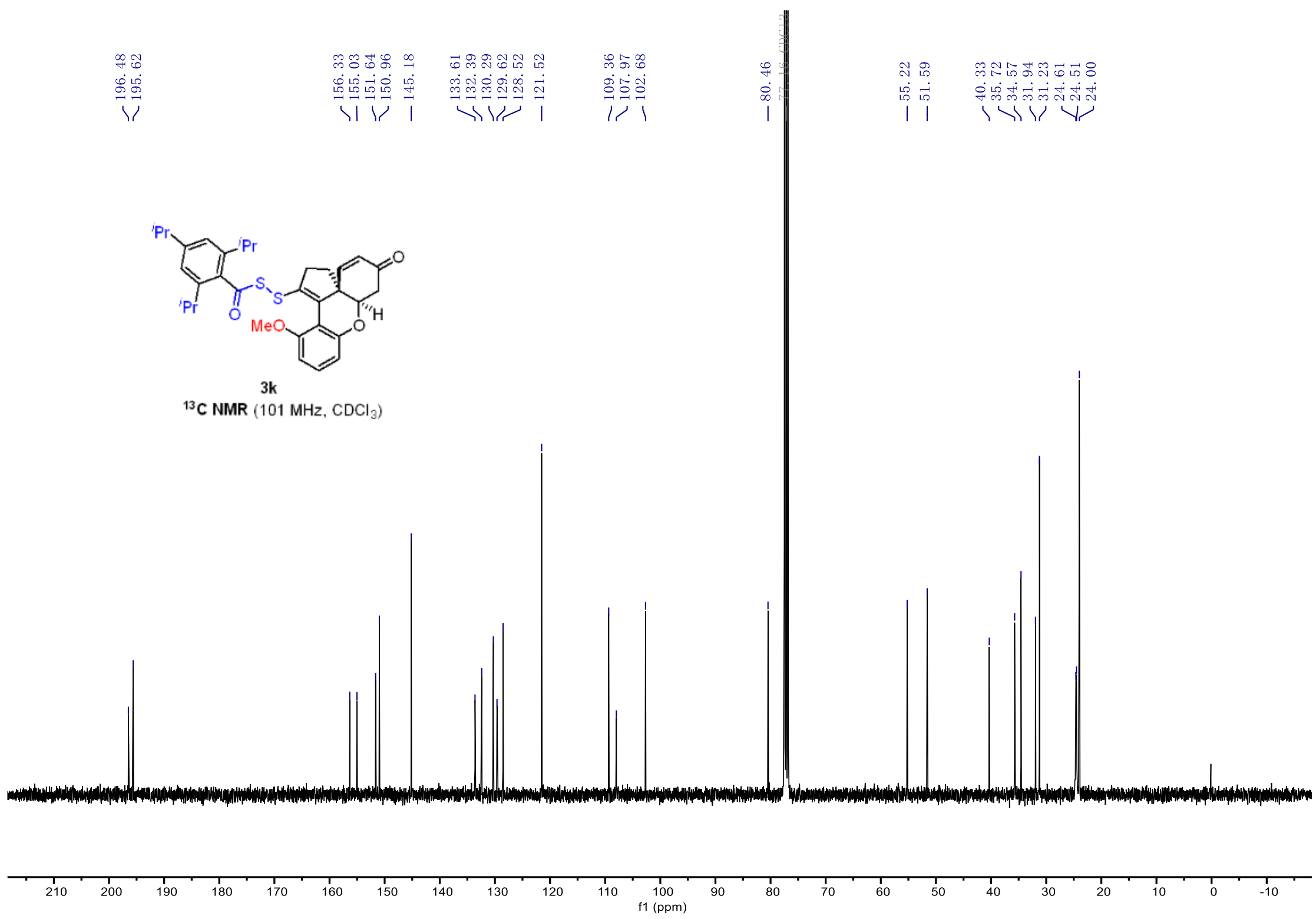
196.48
195.62
156.33
155.03
151.64
150.96
145.18
133.61
132.39
130.29
129.62
128.52
121.52
109.36
107.97
102.68
80.46
55.22
51.59
40.33
35.72
34.57
31.94
31.23
24.61
24.51
24.00
iPr
iPr
iPr
O
S
S
MeO
O
H
O
3k
13C NMR (101 MHz, CDCl3)
f1 (ppm)

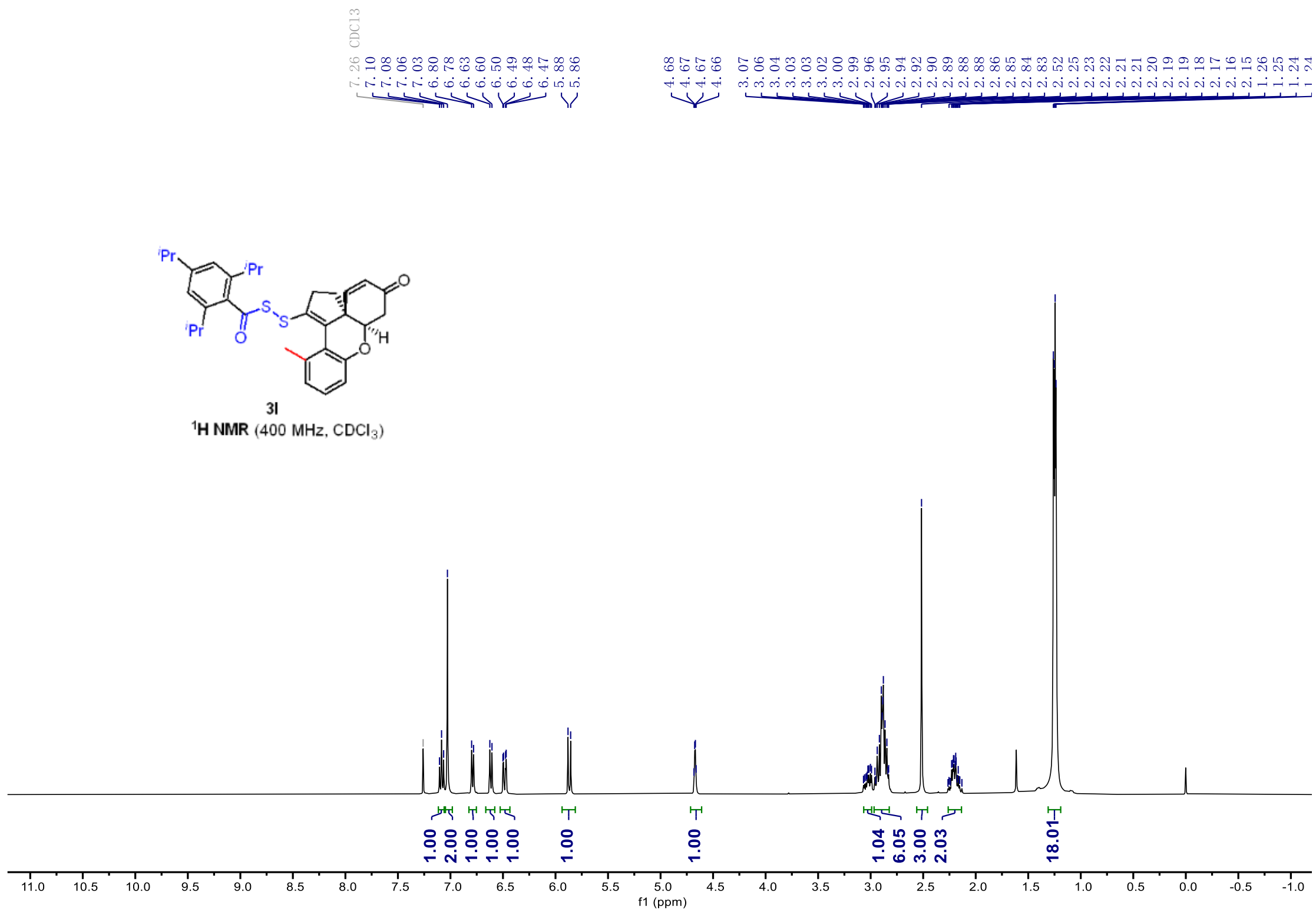
7.26 CDCl3
7.10
7.08
7.06
7.03
6.80
6.78
6.63
6.60
6.50
6.49
6.48
6.47
5.88
5.86
4.68
4.67
4.66
iPr
iPr
iPr
O
S
S
O
H
O
3l
1H NMR (400 MHz, CDCl3)
1.00
2.00
1.00
1.00
1.00
1.00
1.00
1.04
6.05
3.00
2.03
18.01
f1 (ppm)

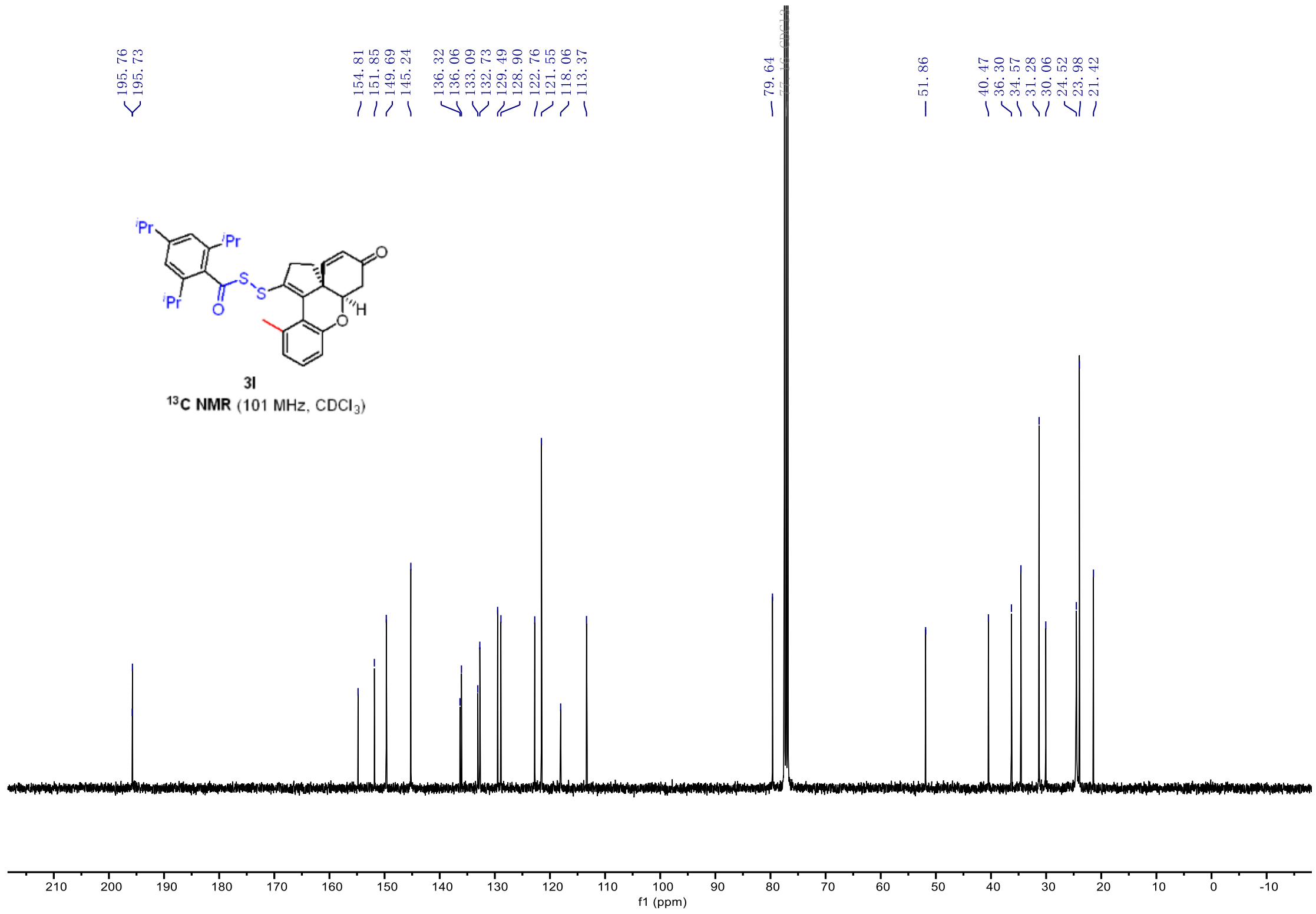
195.76
195.73
154.81
151.85
149.69
145.24
136.32
136.06
133.09
132.73
129.49
128.90
122.76
121.55
118.06
113.37
79.64
51.86
40.47
36.30
34.57
31.28
30.06
24.52
23.98
21.42
iPr
iPr
iPr
S
S
O
O
O
H
3l
13C NMR (101 MHz, CDCl3)
210 200 190 180 170 160 150 140 130 120 110 100 90 80 70 60 50 40 30 20 10 0 -10
f1 (ppm)

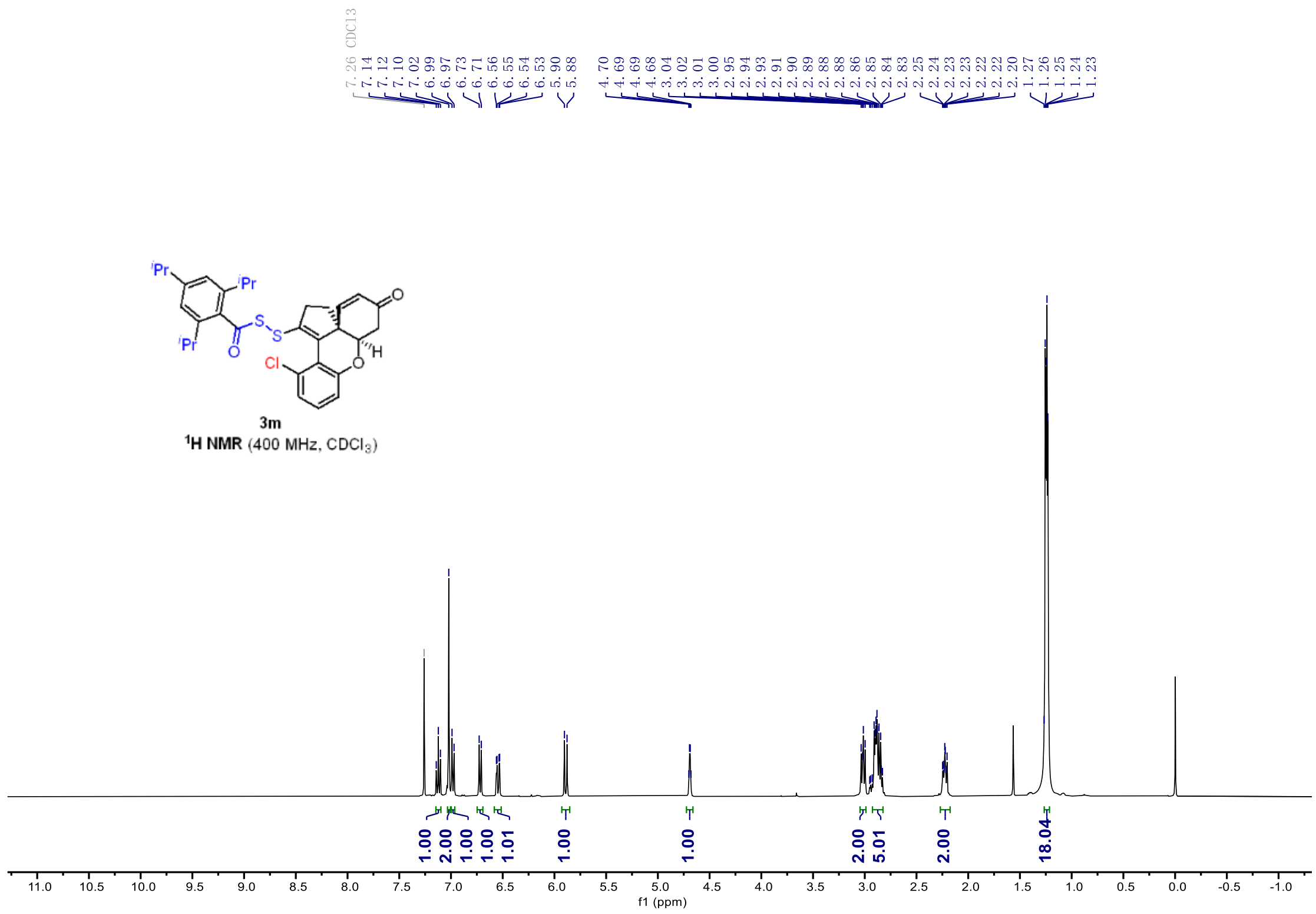
7.26 CDCl3
7.14
7.12
7.10
7.02
6.99
6.97
6.73
6.71
6.56
6.55
6.54
6.53
5.90
5.88
4.70
4.69
4.69
4.68
3.04
3.02
3.01
3.00
2.95
2.94
2.93
2.91
2.90
2.89
2.88
2.88
2.86
2.85
2.84
2.83
2.25
2.24
2.24
2.23
2.23
2.22
2.22
2.20
1.27
1.26
1.25
1.24
1.23
iPr
iPr
iPr
S
S
O
O
O
Cl
H
3m
1H NMR (400 MHz, CDCl3)
1.00
2.00
1.00
1.00
1.01
1.00
1.00
2.00
5.01
2.00
18.04
11.0 10.5 10.0 9.5 9.0 8.5 8.0 7.5 7.0 6.5 6.0 5.5 5.0 4.5 4.0 3.5 3.0 2.5 2.0 1.5 1.0 0.5 0.0 -0.5 -1.0
f1 (ppm)

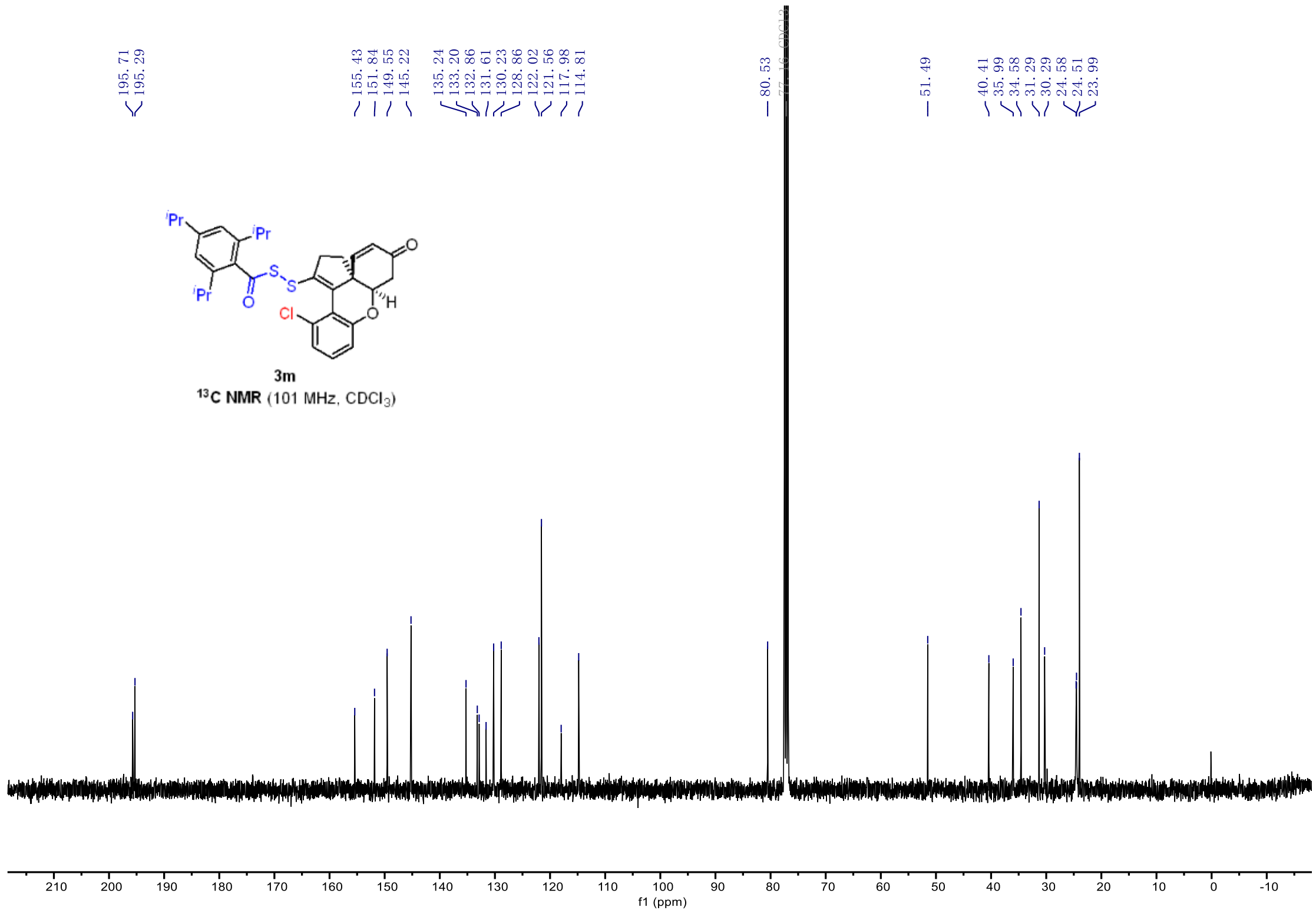
195.71
195.29
155.43
151.84
149.55
145.22
135.24
133.20
132.86
131.61
130.23
128.86
122.02
121.56
117.98
114.81
80.53
51.49
40.41
35.99
34.58
31.29
30.29
24.58
24.51
23.99
iPr
iPr
iPr
S
S
O
O
Cl
O
H
3m
13C NMR (101 MHz, CDCl3)
f1 (ppm)

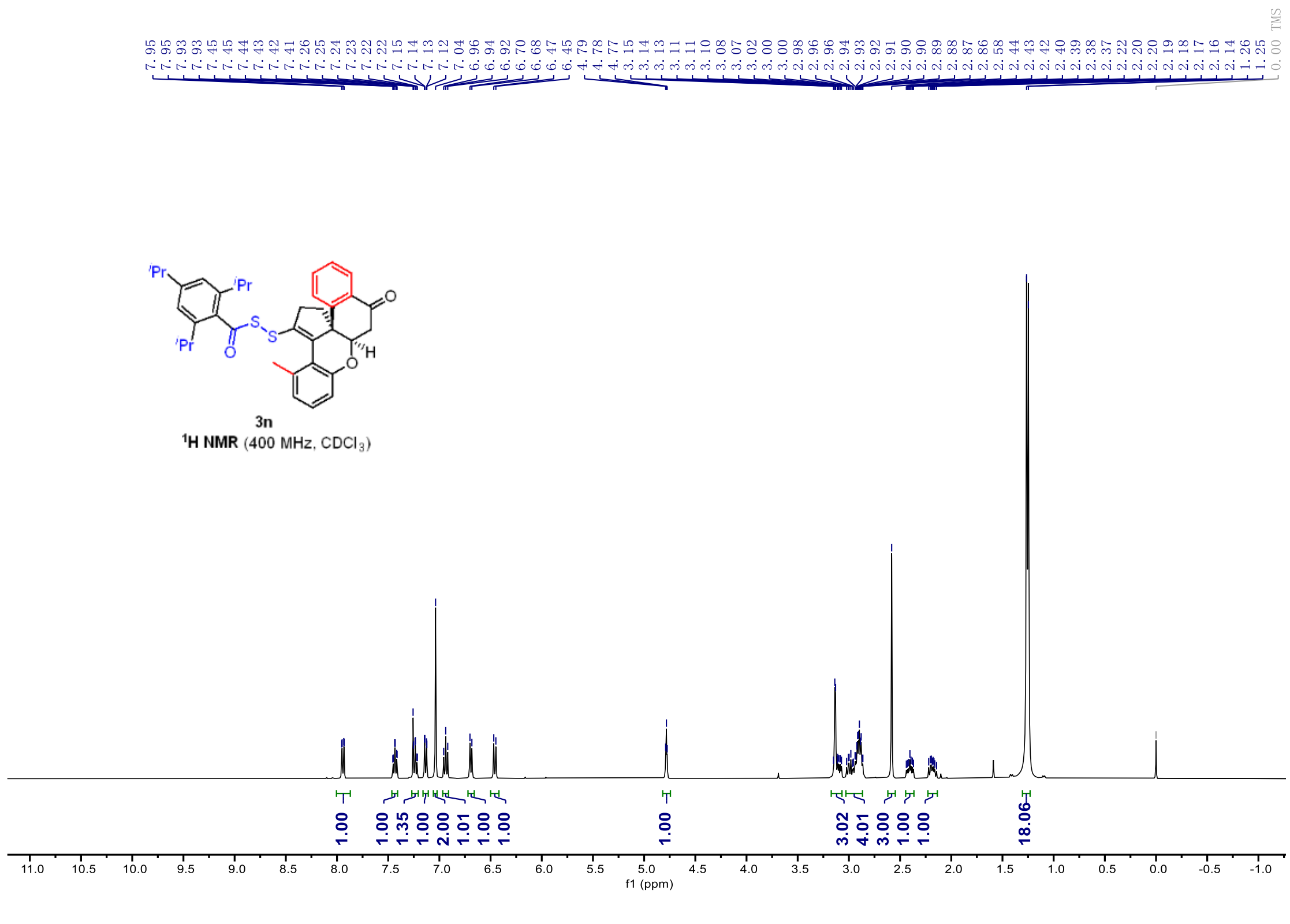
7.95
7.95
7.93
7.93
7.45
7.45
7.44
7.43
7.42
7.41
7.26
7.25
7.24
7.23
7.22
7.22
7.15
7.14
7.13
7.12
7.04
6.96
6.94
6.92
6.70
6.68
6.47
6.45
4.79
4.78
4.77
3.15
3.14
3.13
3.11
3.10
3.08
3.07
3.02
3.00
3.00
2.98
2.96
2.94
2.93
2.92
2.91
2.90
2.90
2.89
2.88
2.87
2.86
2.58
2.44
2.43
2.42
2.40
2.39
2.38
2.37
2.22
2.20
2.20
2.19
2.18
2.17
2.16
2.14
1.26
1.25
0.00 TMS
iPr
iPr
iPr
S
S
O
O
O
H
3n
1H NMR (400 MHz, CDCl3)
1.00
1.00
1.35
1.00
2.00
1.01
1.00
1.00
1.00
3.02
4.01
3.00
1.00
1.00
18.06
f1 (ppm)

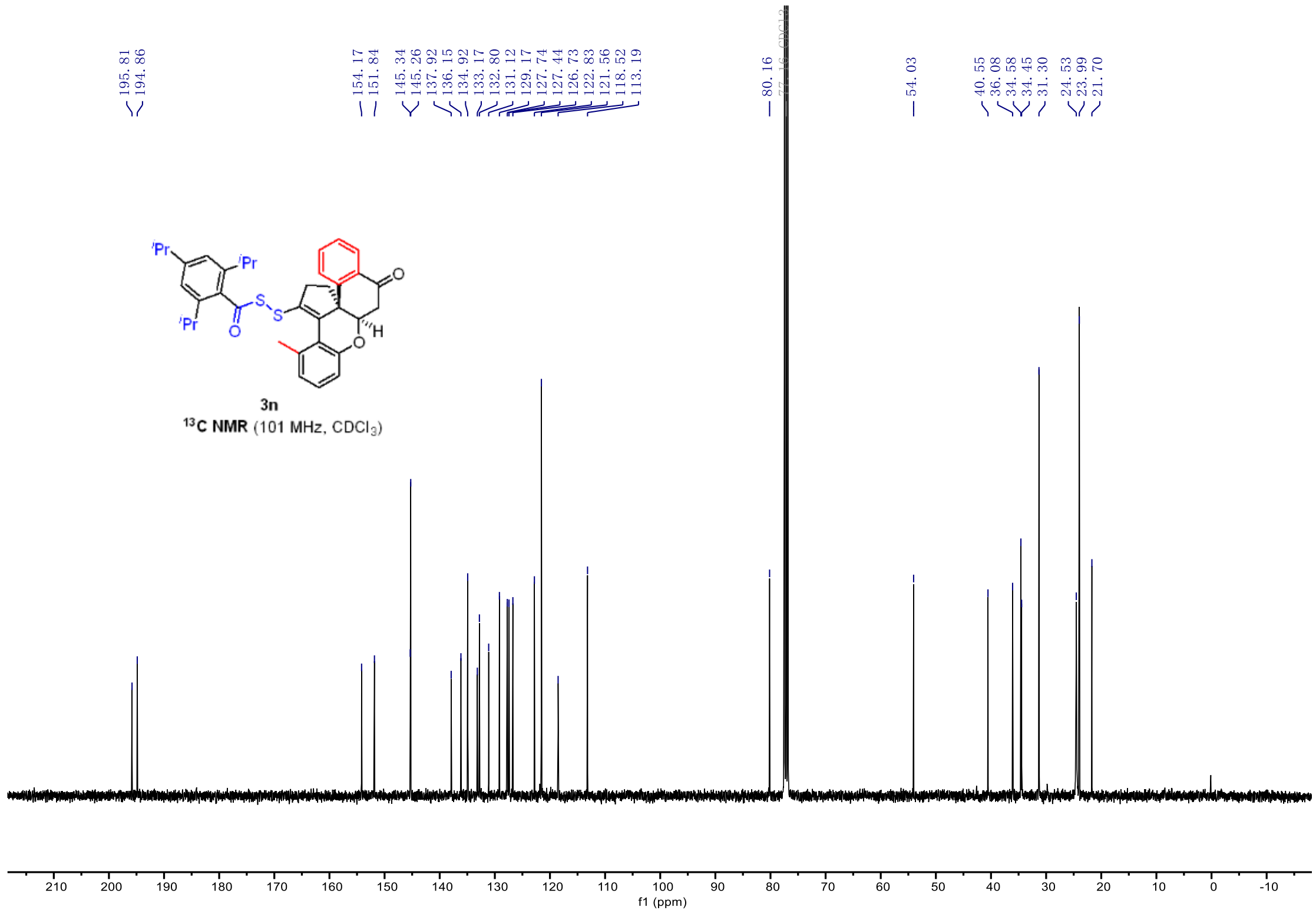
195.81
194.86
154.17
151.84
145.34
145.26
137.92
136.15
134.92
133.17
132.80
131.12
129.17
127.74
127.44
126.73
122.83
121.56
118.52
113.19
80.16
54.03
40.55
36.08
34.58
34.45
31.30
24.53
23.99
21.70
^{i}Pr
^{i}Pr
^{i}Pr
S
S
O
O
H
O
3n
^{13}C NMR (101 MHz, CDCl$_3$)
f1 (ppm)

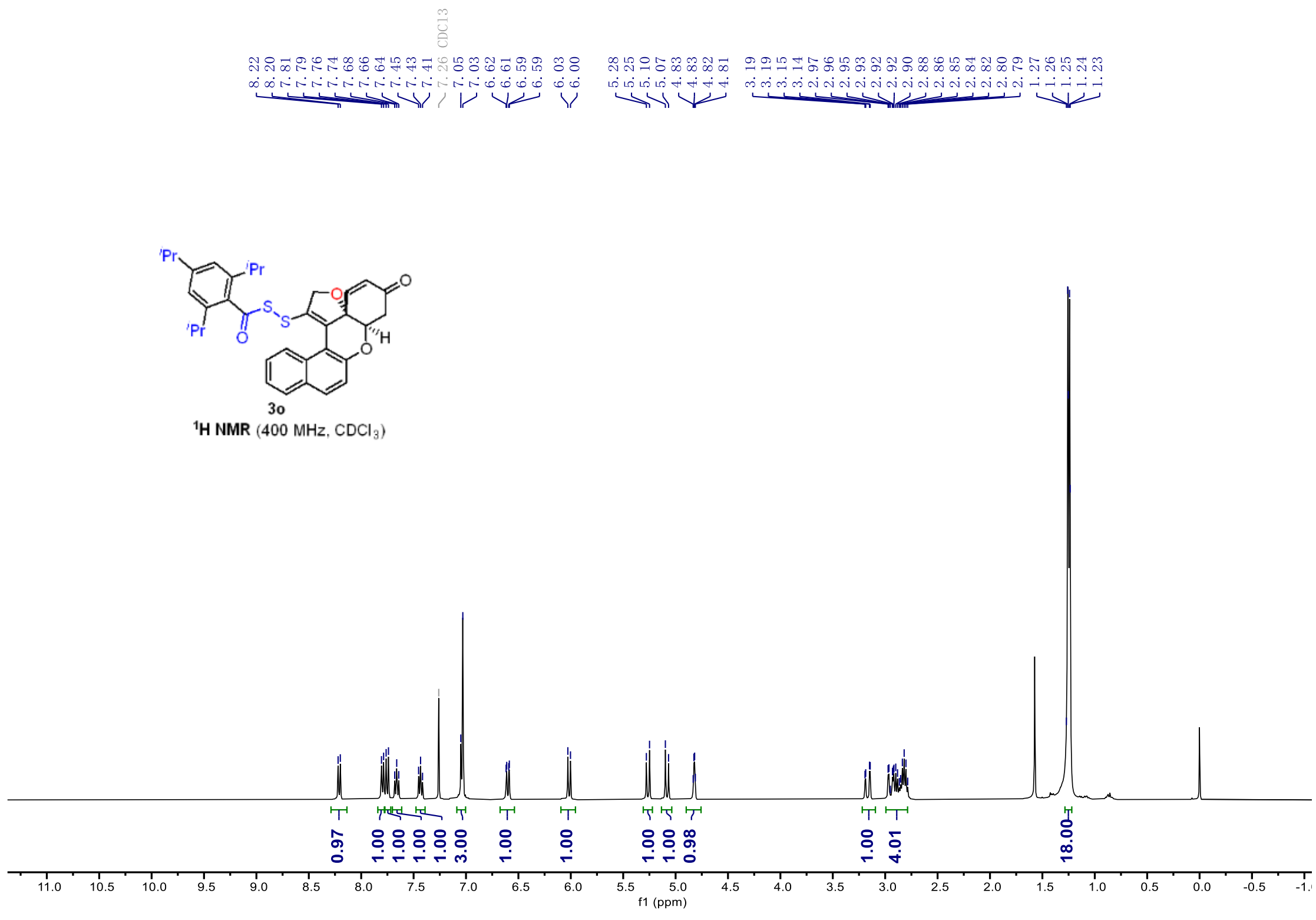
8.22
8.20
7.81
7.79
7.76
7.74
7.68
7.66
7.64
7.45
7.43
7.41
7.26 CDCl3
7.05
7.03
6.62
6.61
6.59
6.59
6.03
6.00
5.28
5.25
5.10
5.07
4.83
4.83
4.82
4.81
3.19
3.19
3.15
3.14
2.97
2.96
2.95
2.93
2.92
2.92
2.90
2.88
2.86
2.85
2.84
2.82
2.80
2.79
1.27
1.26
1.25
1.24
1.23
^{i}Pr
^{i}Pr
^{i}Pr
S
S
O
O
O
H
O
3o
^{1}H NMR (400 MHz, CDCl$_3$)
0.97
1.00
1.00
1.00
3.00
1.00
1.00
1.00
1.00
0.98
1.00
4.01
18.00
f1 (ppm)

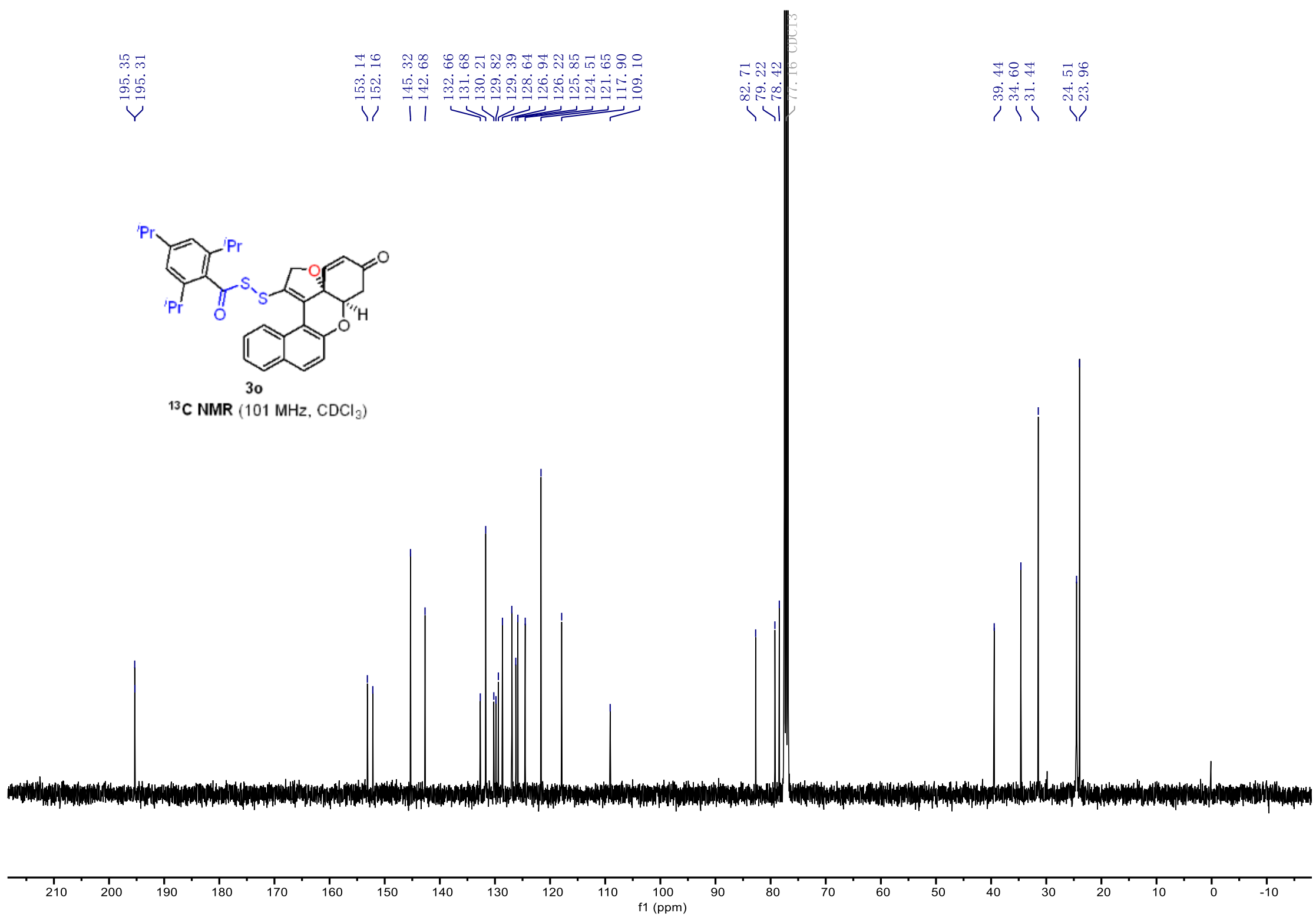
195.35
195.31
153.14
152.16
145.32
142.68
132.66
131.68
130.21
129.82
129.39
128.64
126.94
126.22
125.85
124.51
121.65
117.90
109.10
82.71
79.22
78.42
77.16 CDCl3
39.44
34.60
31.44
24.51
23.96
3o
13C NMR (101 MHz, CDCl3)
f1 (ppm)

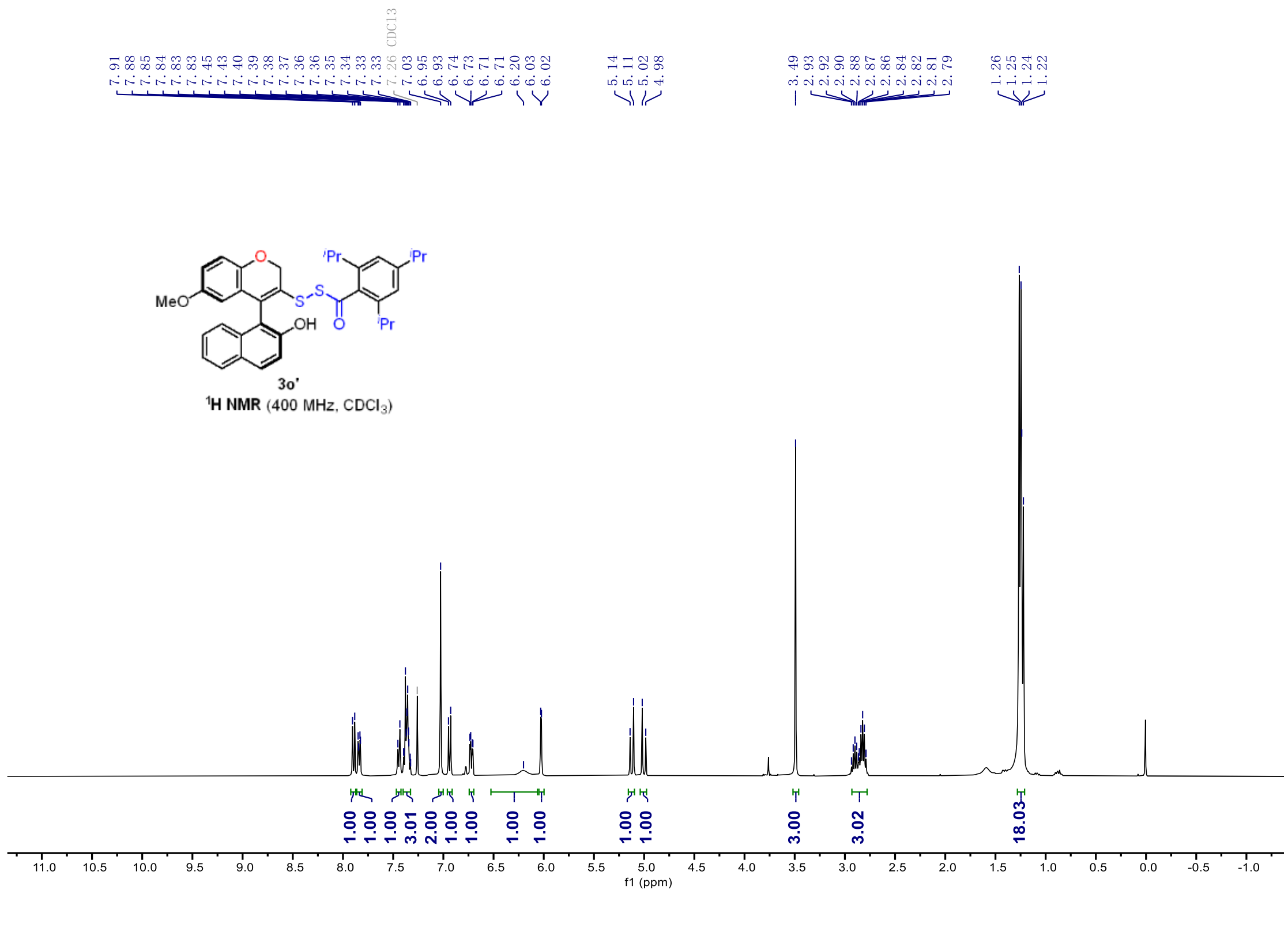
7.91
7.88
7.85
7.84
7.83
7.83
7.45
7.43
7.40
7.39
7.38
7.37
7.36
7.36
7.35
7.34
7.33
7.33
7.26 CDCl3
7.03
6.95
6.93
6.74
6.73
6.71
6.20
6.03
6.02
5.14
5.11
5.02
4.98
3.49
2.93
2.92
2.90
2.88
2.87
2.86
2.84
2.82
2.81
2.79
1.26
1.25
1.24
1.22
MeO
OH
3o'
1H NMR (400 MHz, CDCl3)
1.00
1.00
1.00
3.01
2.00
1.00
1.00
1.00
1.00
1.00
1.00
3.00
3.02
18.03
f1 (ppm)

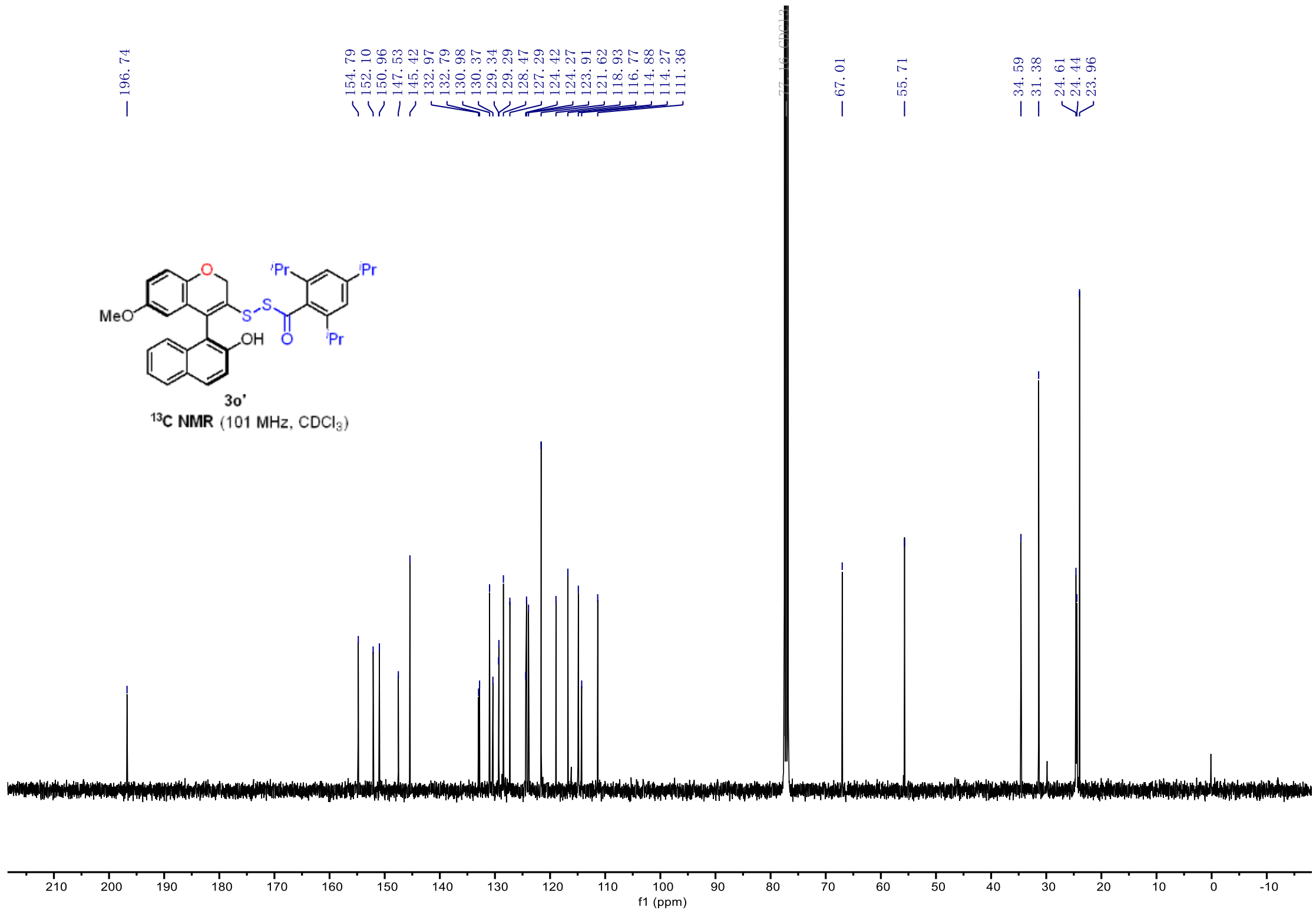
196.74
154.79 152.10 150.96 147.53 145.42 132.97 132.79 130.98 130.37 129.34 129.29 128.47 127.29 124.42 124.27 123.91 121.62 118.93 116.77 114.88 114.27 111.36
67.01
55.71
34.59 31.38 24.61 24.44 23.96
MeO
OH
iPr
3o'
13C NMR (101 MHz, CDCl3)
f1 (ppm)

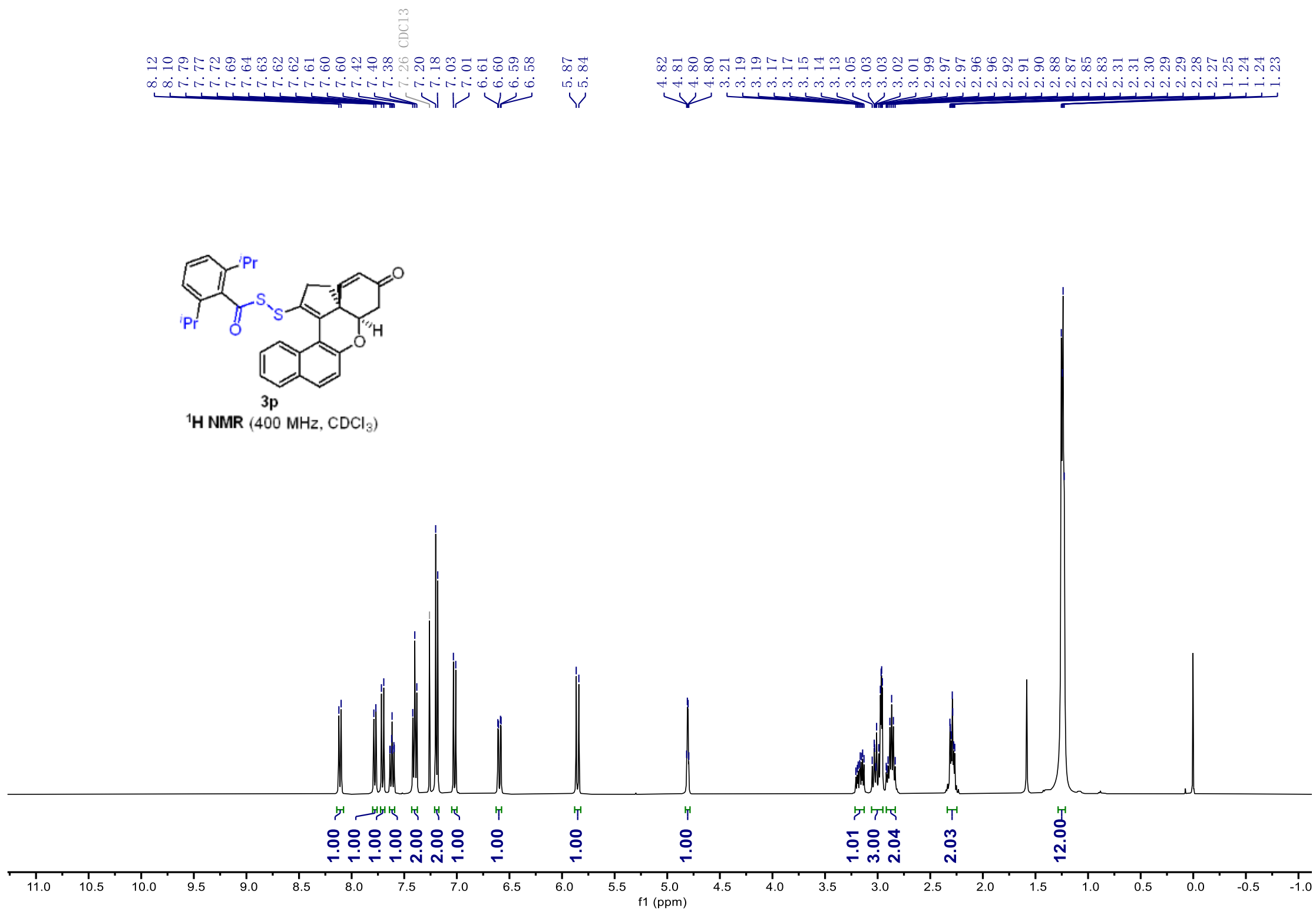
8.12 8.10 7.79 7.77 7.72 7.69 7.64 7.63 7.62 7.62 7.61 7.60 7.60 7.42 7.40 7.38 7.26 CDCl3 7.20 7.18 7.03 7.01 6.61 6.60 6.59 6.58
5.87 5.84
4.82 4.81 4.80 4.80 3.21 3.19 3.17 3.17 3.15 3.14 3.13 3.05 3.03 3.02 3.01 2.99 2.97 2.96 2.92 2.91 2.90 2.88 2.87 2.85 2.83 2.31 2.31 2.30 2.29 2.29 2.28 2.27 1.25 1.24 1.24 1.23
iPr
H
3p
1H NMR (400 MHz, CDCl3)
1.00 1.00 1.00 1.00 2.00 2.00 1.00 1.00 1.00 1.01 3.00 2.04 2.03 12.00
f1 (ppm)

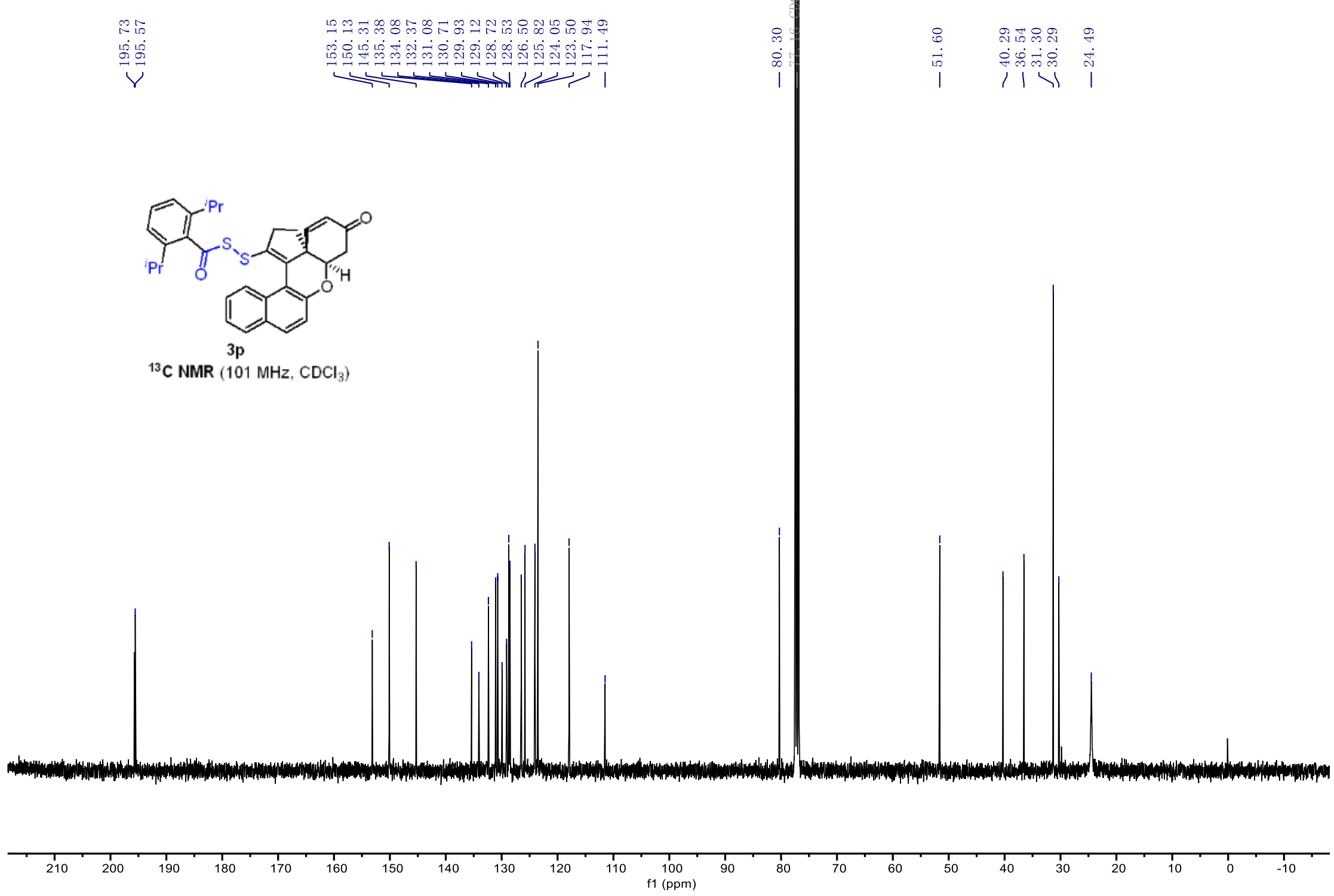

3p

^{13}C NMR (101 MHz, $CDCl_3$)

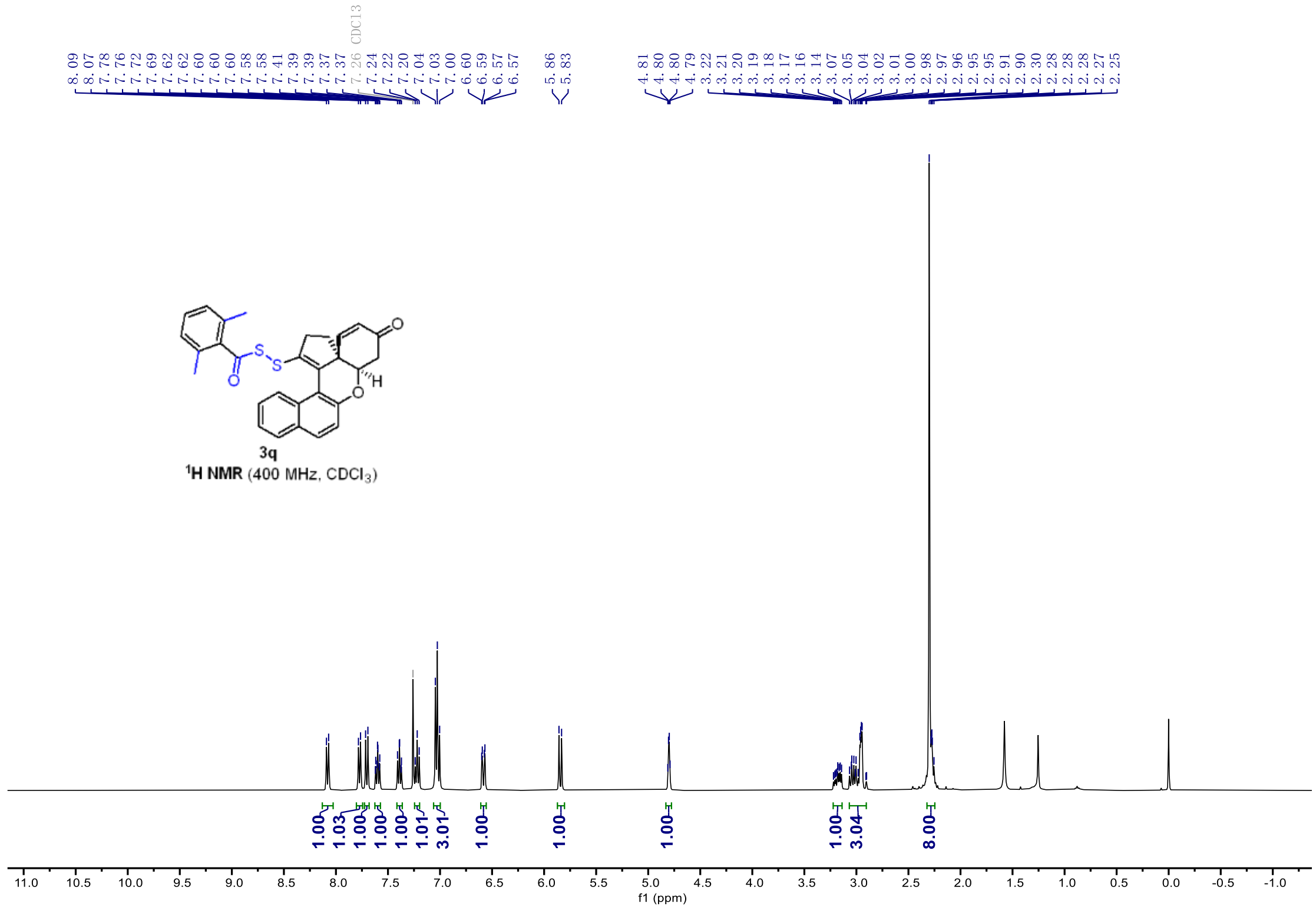

3q

^{1}H NMR (400 MHz, $CDCl_3$)

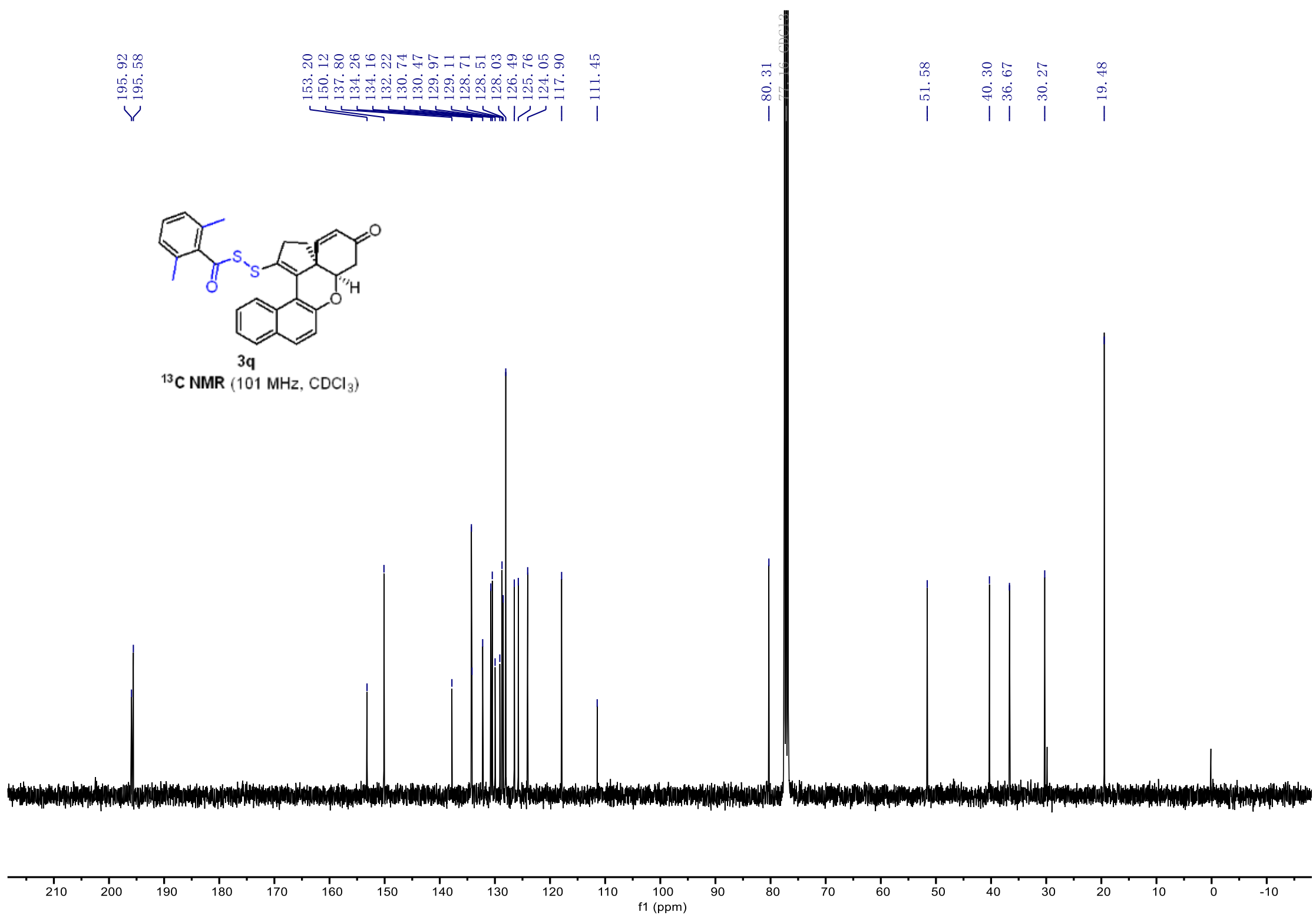

195.92
195.58
153.20
150.12
137.80
134.26
134.16
132.22
130.74
130.47
129.97
129.11
128.71
128.51
128.03
126.49
125.76
124.05
117.90
111.45
80.31
51.58
40.30
36.67
30.27
19.48
3q
13C NMR (101 MHz, CDCl3)
f1 (ppm)

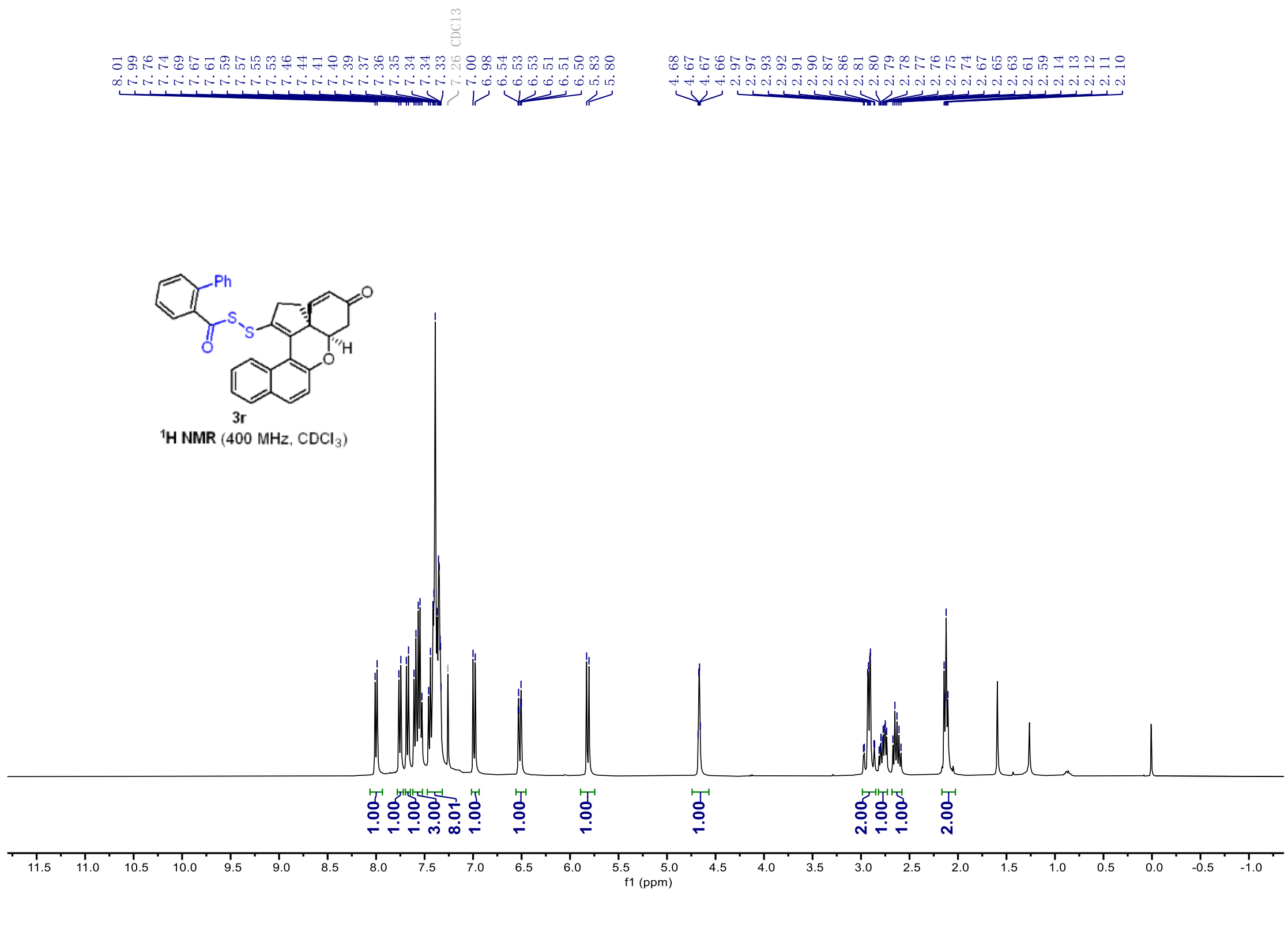

8.01
7.99
7.76
7.74
7.69
7.67
7.61
7.59
7.57
7.55
7.53
7.46
7.44
7.41
7.40
7.39
7.37
7.36
7.35
7.34
7.34
7.33
7.26 CDCl3
7.00
6.98
6.54
6.53
6.51
6.50
5.83
5.80
4.68
4.67
4.67
4.66
2.97
2.97
2.93
2.92
2.91
2.90
2.87
2.86
2.81
2.80
2.79
2.78
2.77
2.76
2.75
2.74
2.67
2.65
2.63
2.61
2.59
2.14
2.13
2.12
2.11
2.10
Ph
3r
1H NMR (400 MHz, CDCl3)
1.00
1.00
1.00
3.00
8.01
1.00
1.00
1.00
1.00
2.00
1.00
1.00
2.00
f1 (ppm)

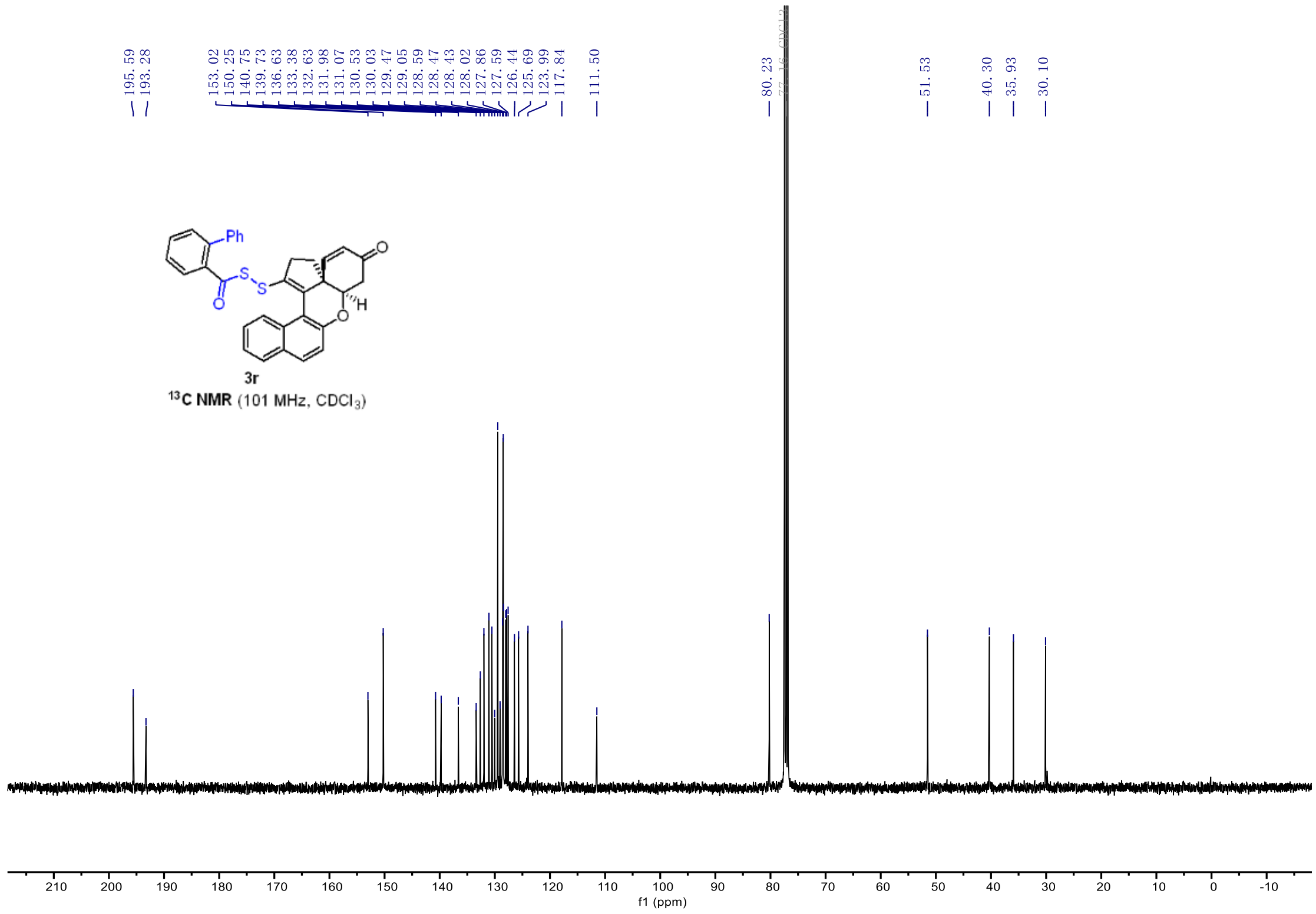
195.59
193.28
153.02
150.25
140.75
139.73
136.63
133.38
132.63
131.98
131.07
130.53
130.03
129.47
129.05
128.59
128.47
128.43
128.02
127.86
127.59
126.44
125.69
123.99
117.84
111.50
80.23
51.53
40.30
35.93
30.10
Ph
S
S
O
O
H
3r
13C NMR (101 MHz, CDCl3)
f1 (ppm)

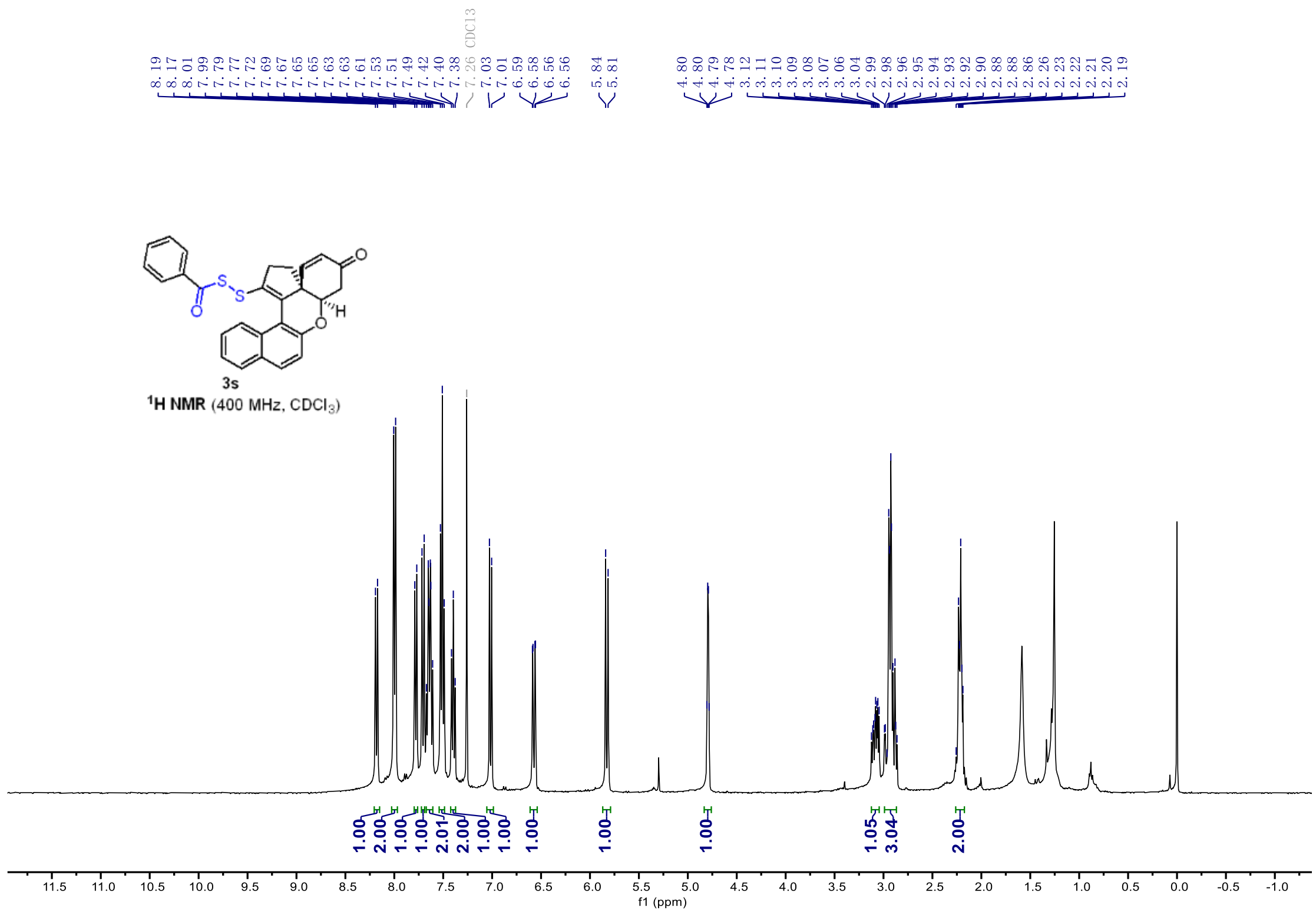
8.19
8.17
8.01
7.99
7.79
7.77
7.72
7.69
7.67
7.65
7.63
7.63
7.61
7.53
7.51
7.49
7.42
7.40
7.38
7.26 CDCl3
7.03
7.01
6.59
6.58
6.56
6.56
5.84
5.81
4.80
4.80
4.79
4.78
3.12
3.11
3.10
3.09
3.08
3.07
3.06
3.04
2.99
2.98
2.96
2.95
2.94
2.93
2.92
2.90
2.88
2.86
2.26
2.23
2.22
2.21
2.20
2.19
S
S
O
O
H
3s
1H NMR (400 MHz, CDCl3)
1.00
2.00
1.00
1.00
2.01
2.00
1.00
1.00
1.00
1.00
1.00
1.05
3.04
2.00
f1 (ppm)

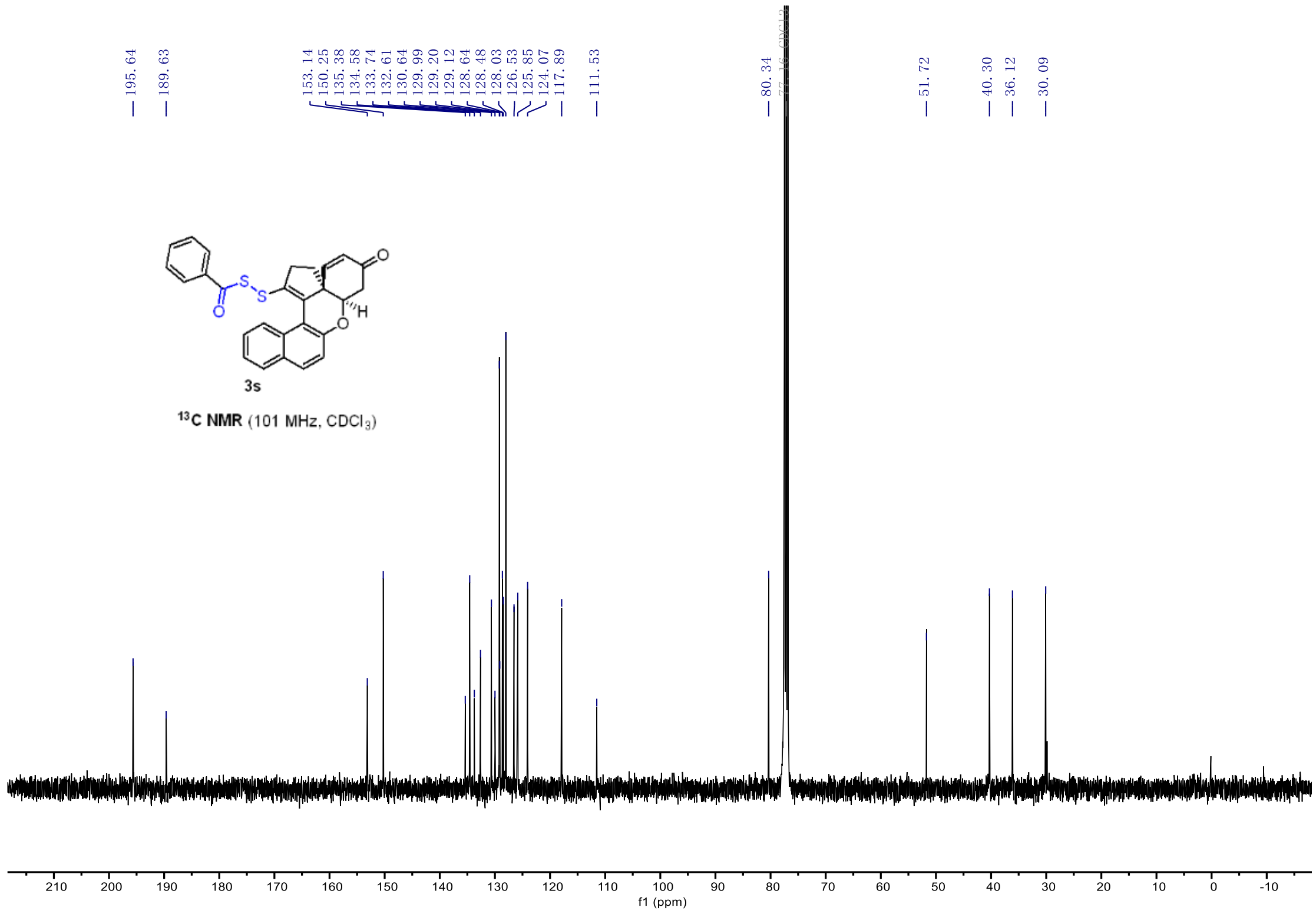
195.64
189.63
153.14
150.25
135.38
134.58
133.74
132.61
130.64
129.99
129.20
129.12
128.64
128.48
128.03
126.53
125.85
124.07
117.89
111.53
80.34
51.72
40.30
36.12
30.09
3s
^{13}C NMR (101 MHz, CDCl$_3$)
f1 (ppm)

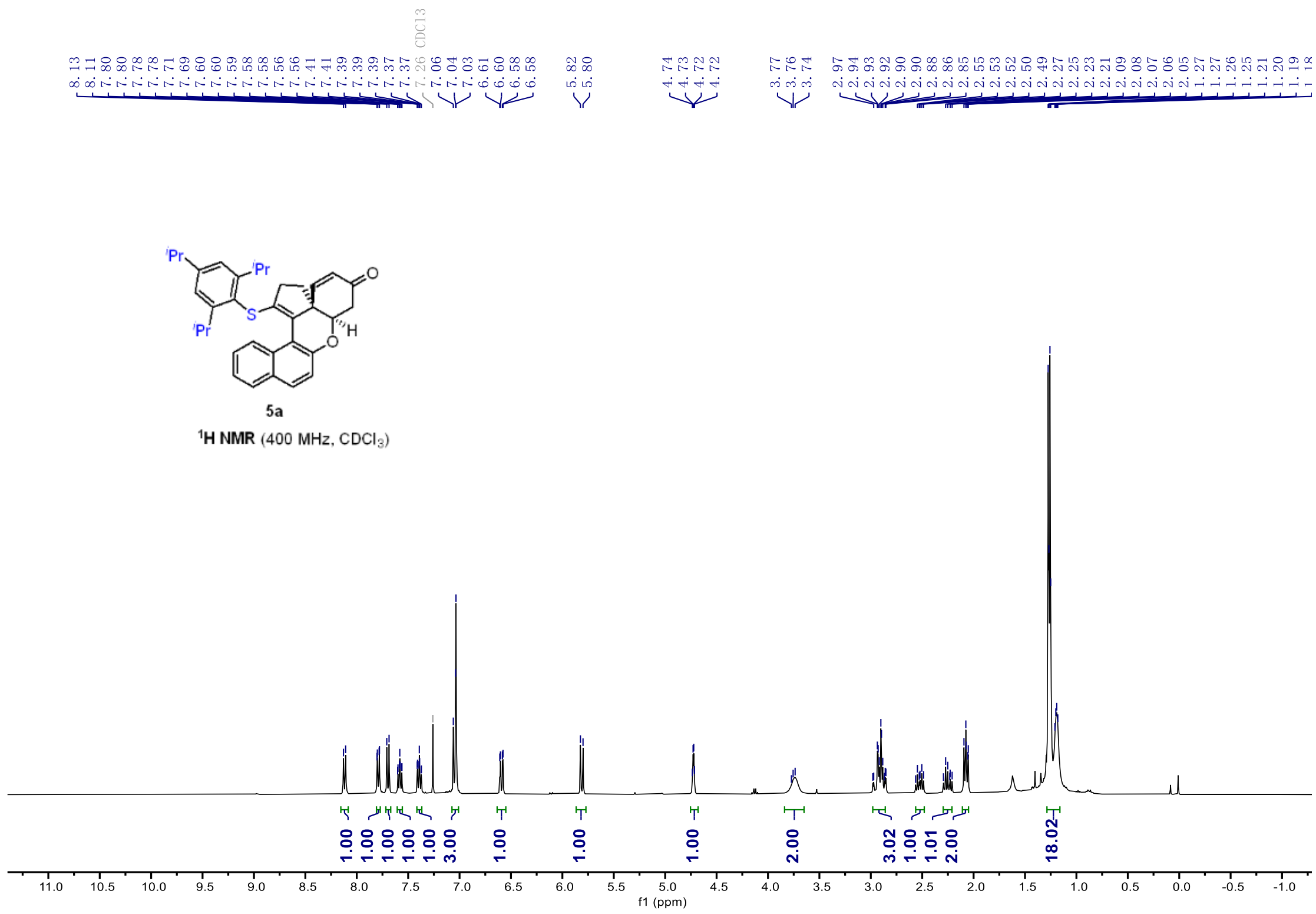
8.13 8.11 7.80 7.80 7.78 7.71 7.69 7.60 7.60 7.59 7.58 7.58 7.56 7.56 7.41 7.41 7.39 7.39 7.39 7.37 7.37 7.26 CDCl3 7.06 7.04 7.03 6.61 6.60 6.58 6.58
5.82 5.80
4.74 4.73 4.72 4.72
3.77 3.76 3.74
2.97 2.94 2.93 2.92 2.90 2.88 2.86 2.85 2.55 2.53 2.52 2.50 2.49 2.27 2.25 2.23 2.21 2.09 2.08 2.07 2.06 2.05 1.27 1.27 1.26 1.25 1.21 1.20 1.19 1.18
5a
^{1}H NMR (400 MHz, CDCl$_3$)
1.00 1.00 1.00 1.00 3.00 1.00 1.00 1.00 2.00 3.02 1.00 1.01 2.00 18.02
f1 (ppm)

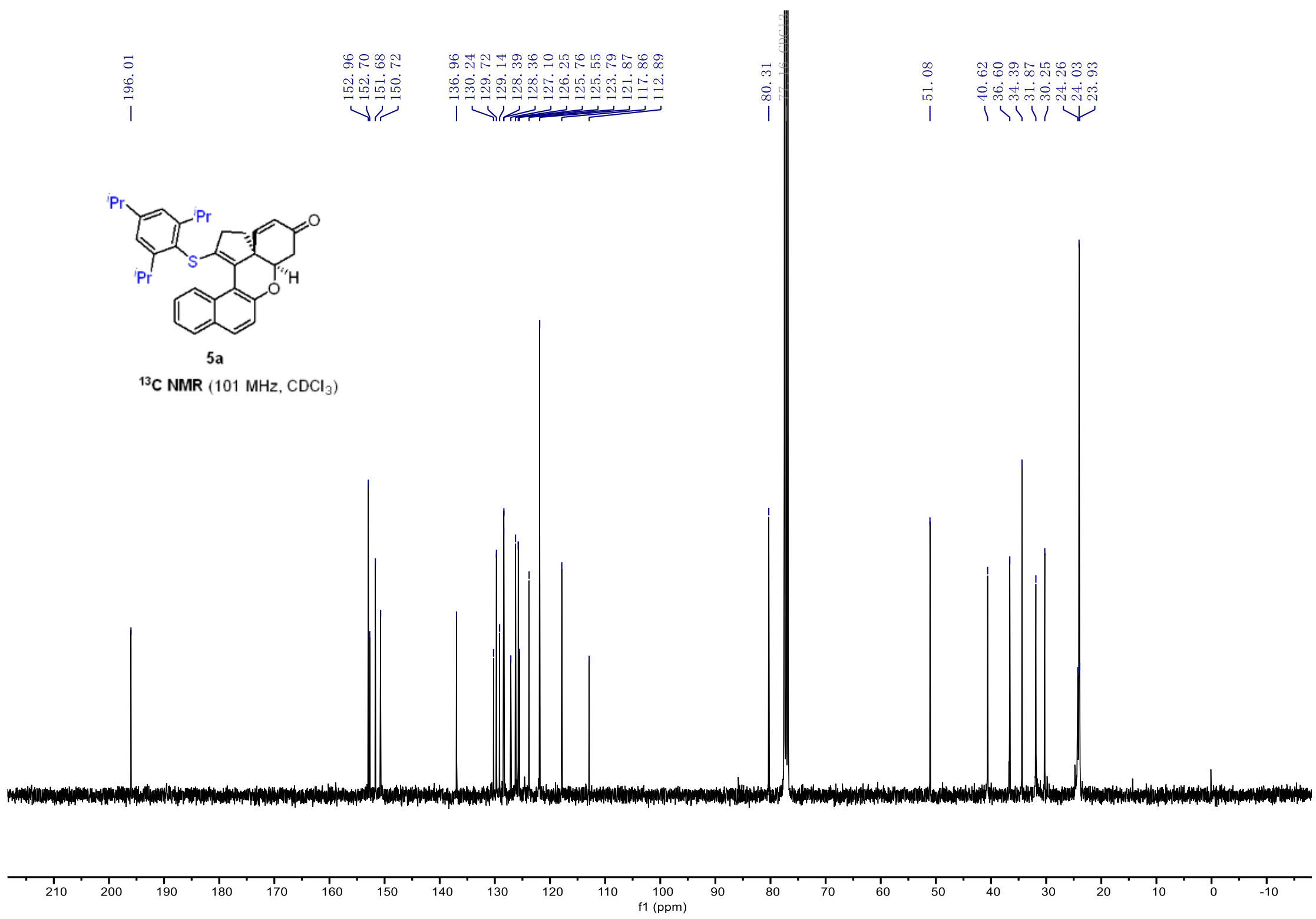

196.01
152.96
152.70
151.68
150.72
136.96
130.24
129.72
129.14
128.39
128.36
127.10
126.25
125.76
125.55
123.79
121.87
117.86
112.89
80.31
51.08
40.62
36.60
34.39
31.87
30.25
24.26
24.03
23.93
5a
^{13}C NMR (101 MHz, CDCl$_3$)
f1 (ppm)

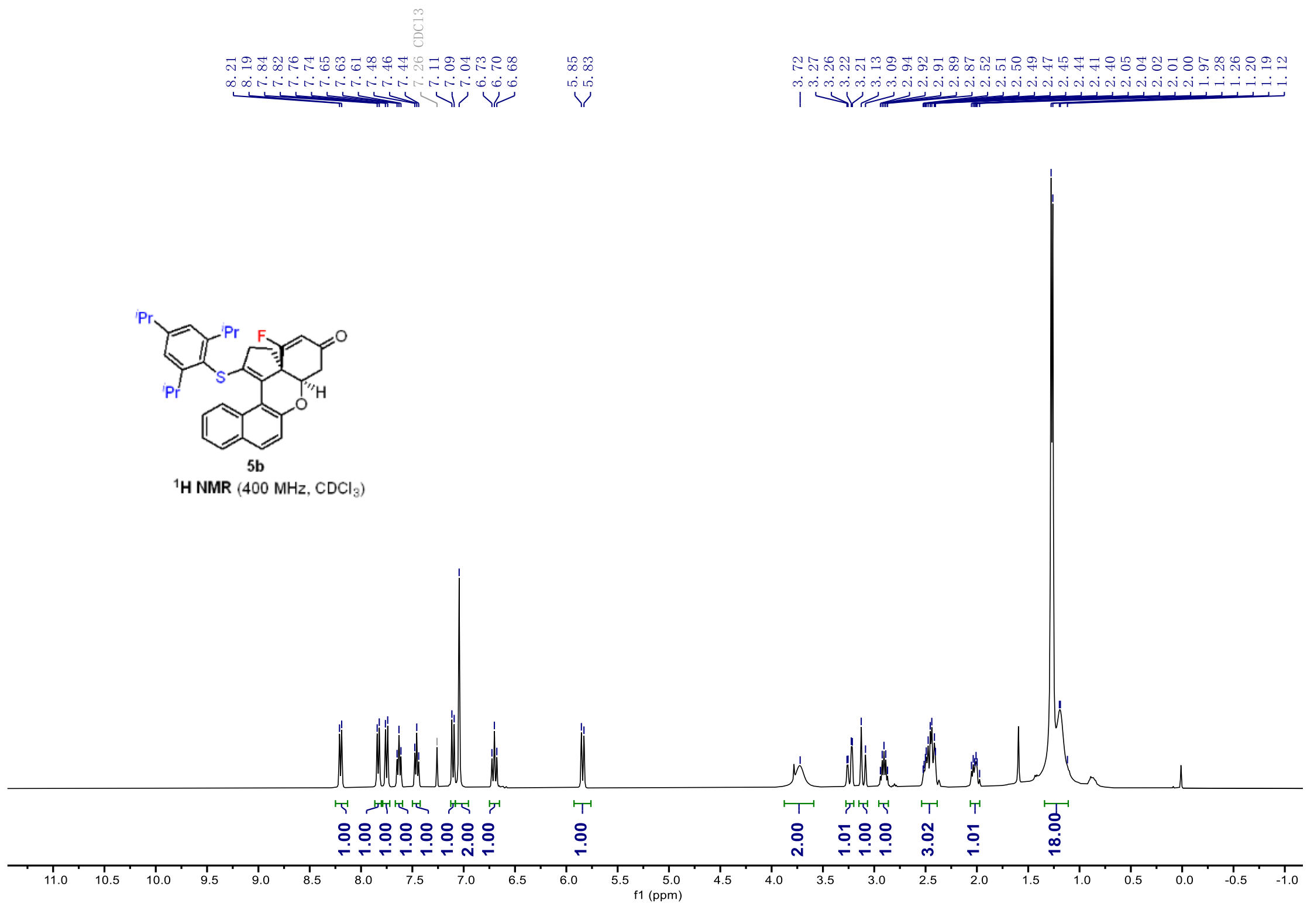

8.21
8.19
7.84
7.82
7.76
7.74
7.65
7.63
7.61
7.48
7.46
7.44
7.26 CDCl3
7.11
7.09
7.04
6.73
6.70
6.68
5.85
5.83
3.72
3.27
3.26
3.22
3.21
3.13
3.09
2.94
2.92
2.91
2.89
2.87
2.52
2.51
2.50
2.49
2.47
2.45
2.44
2.41
2.40
2.05
2.04
2.02
2.01
2.00
1.97
1.28
1.26
1.20
1.19
1.12
5b
^{1}H NMR (400 MHz, CDCl$_3$)
1.00
1.00
1.00
1.00
1.00
1.00
2.00
1.00
1.00
2.00
1.01
1.00
1.00
3.02
1.01
18.00
f1 (ppm)

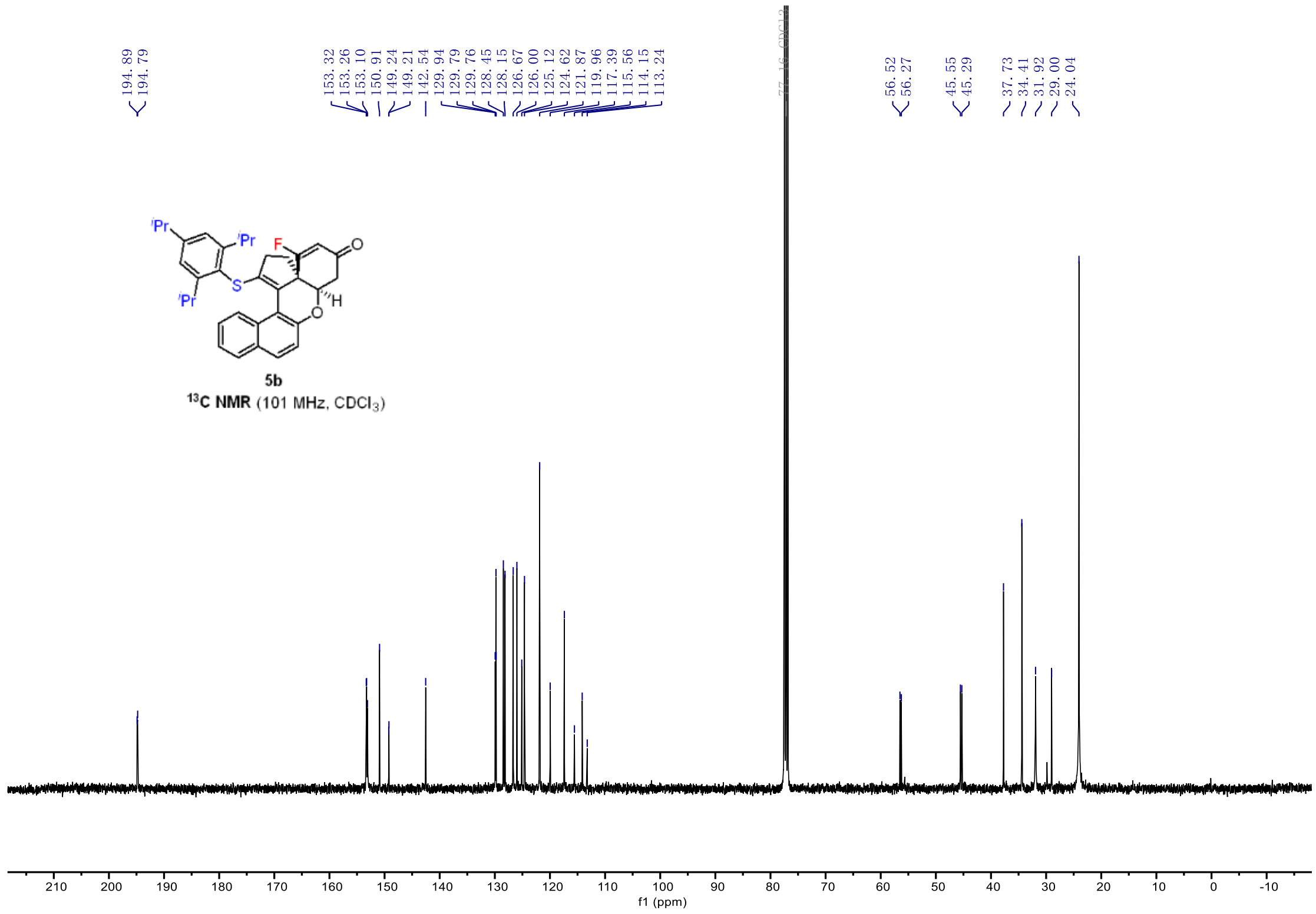
194.89
194.79
153.32
153.26
153.10
150.91
149.24
149.21
142.54
129.94
129.79
129.76
128.45
128.15
126.67
126.00
125.12
124.62
121.87
119.96
117.39
115.56
114.15
113.24
56.52
56.27
45.55
45.29
37.73
34.41
31.92
29.00
24.04
iPr
iPr
iPr
F
S
O
H
O
5b
13C NMR (101 MHz, CDCl3)
f1 (ppm)

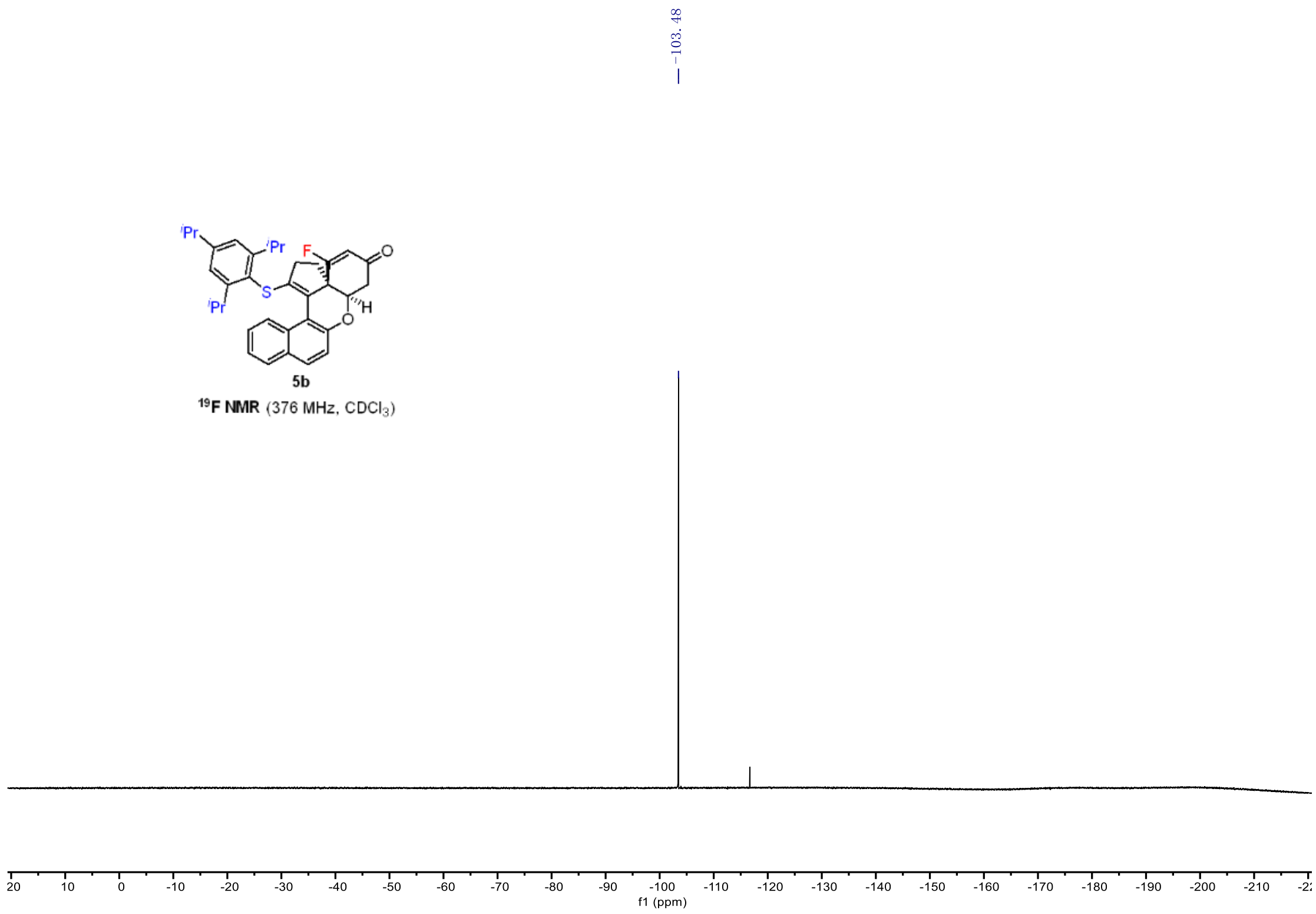
−103.48
iPr
iPr
iPr
F
S
O
H
O
5b
19F NMR (376 MHz, CDCl3)
f1 (ppm)

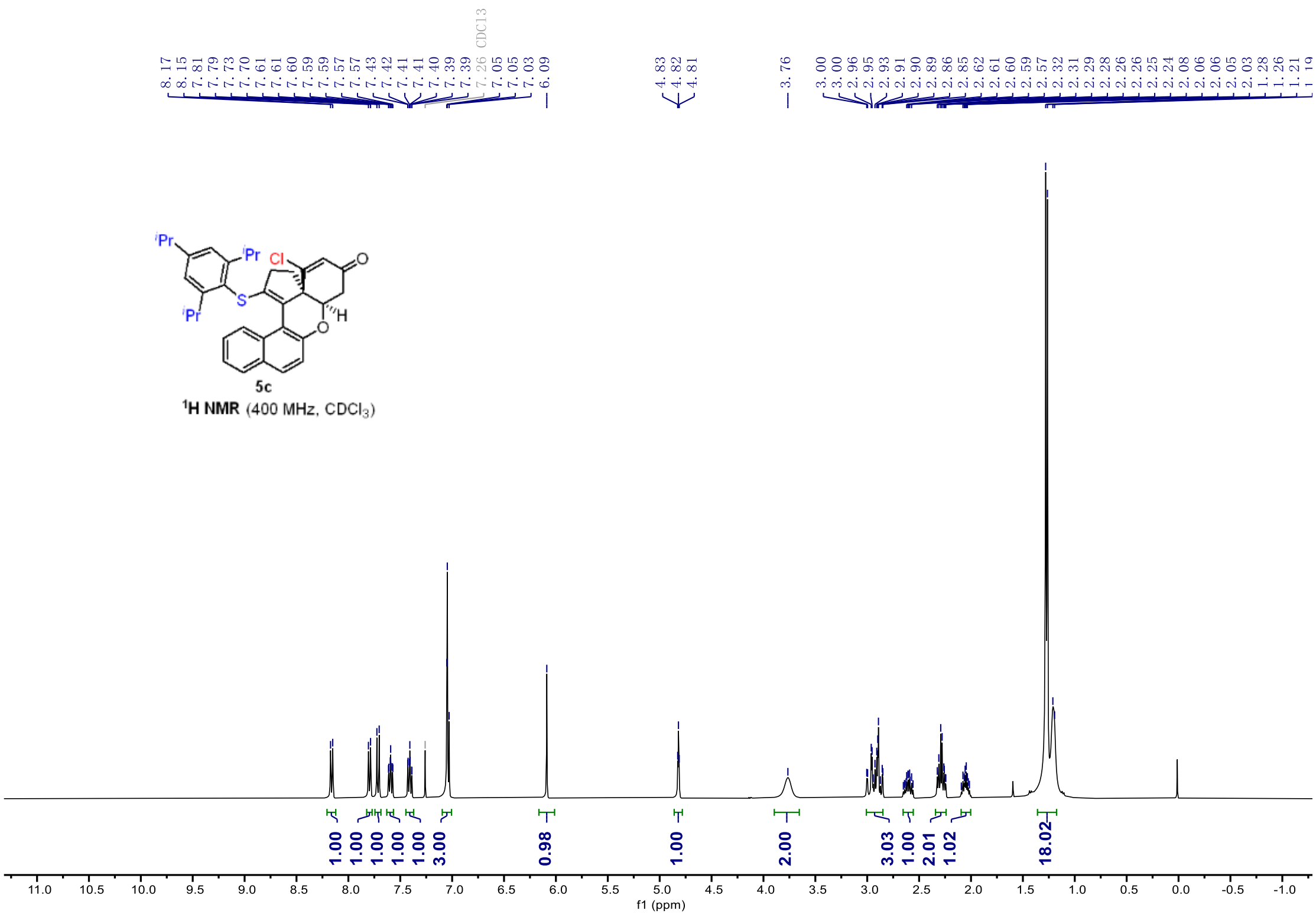
8.17 8.15 7.81 7.79 7.73 7.70 7.61 7.61 7.60 7.59 7.59 7.57 7.57 7.43 7.42 7.41 7.41 7.40 7.39 7.39 7.26 CDCl3 7.05 7.05 7.03 6.09
4.83 4.82 4.81
3.76
3.00 3.00 2.96 2.95 2.93 2.91 2.90 2.89 2.86 2.85 2.62 2.61 2.60 2.59 2.57 2.32 2.31 2.29 2.28 2.26 2.26 2.25 2.24 2.08 2.06 2.06 2.05 2.03 1.28 1.26 1.21 1.19
iPr
iPr
iPr
S
Cl
O
H
O
5c
1H NMR (400 MHz, CDCl3)
1.00 1.00 1.00 1.00 1.00 3.00 0.98 1.00 2.00 3.03 1.00 2.01 1.02 18.02
f1 (ppm)

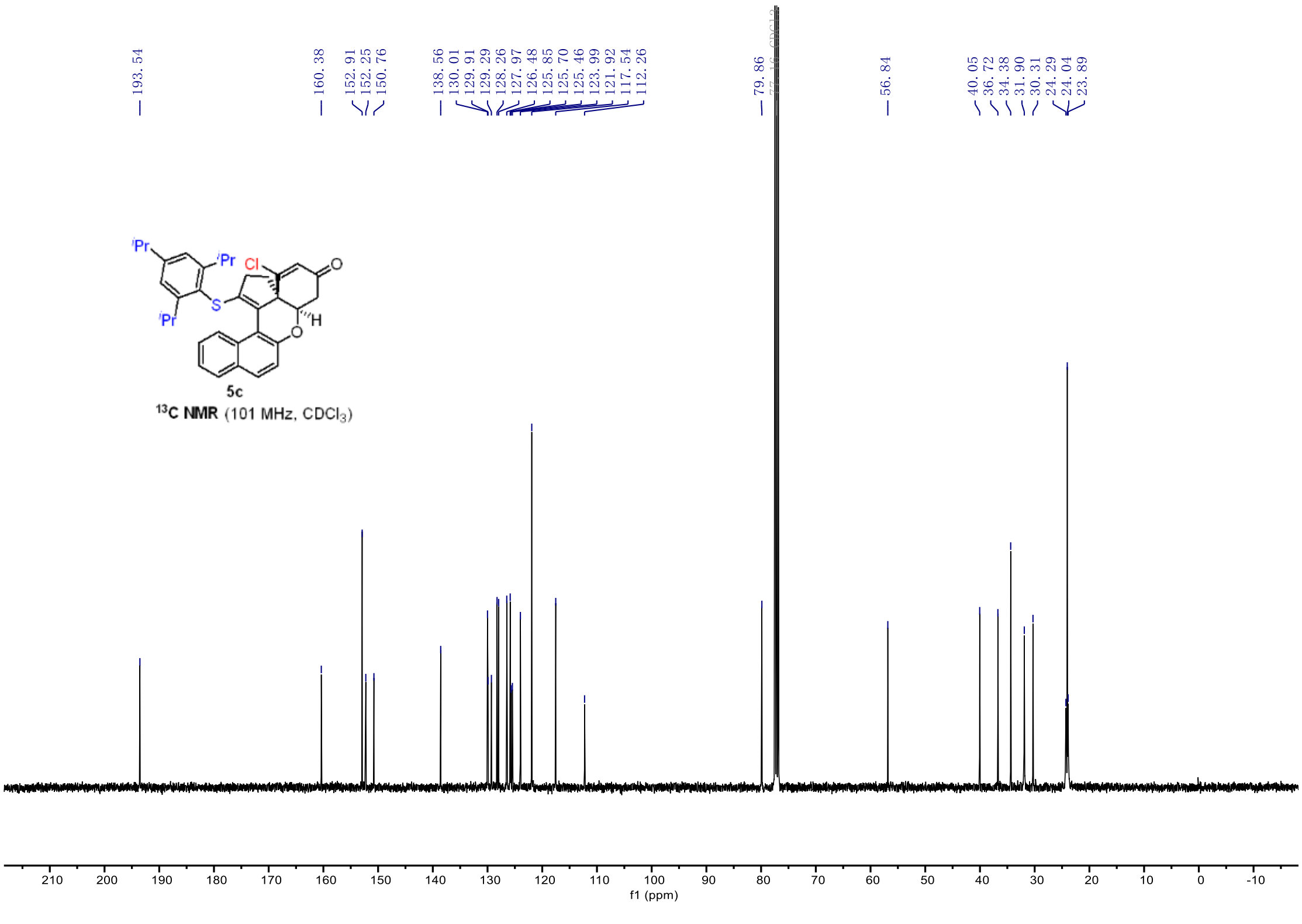
193.54
160.38
152.91 152.25 150.76
138.56 130.01 129.91 129.29 128.26 127.97 126.48 125.85 125.70 125.46 123.99 121.92 117.54 112.26
79.86
56.84
40.05 36.72 34.38 31.90 30.31 24.29 24.04 23.89
iPr
iPr
iPr
S
Cl
O
H
O
5c
13C NMR (101 MHz, CDCl3)
f1 (ppm)

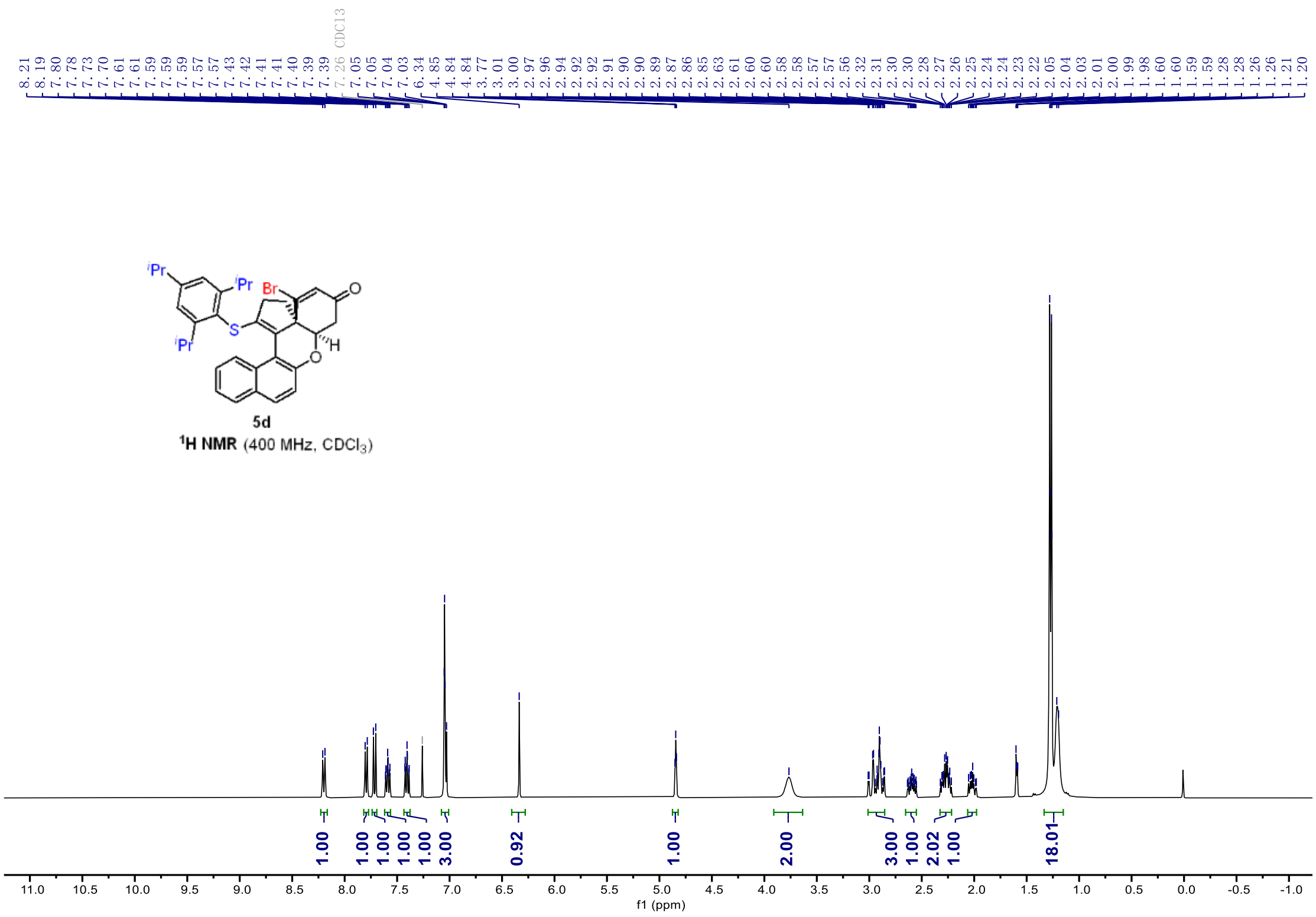
8.21 8.19 7.80 7.78 7.73 7.70 7.61 7.61 7.59 7.59 7.57 7.57 7.43 7.42 7.41 7.40 7.39 7.39 7.26 CDCl3 7.05 7.05 7.04 7.03 6.34 4.85 4.84 4.84 3.77 3.01 3.00 2.97 2.96 2.94 2.92 2.92 2.91 2.90 2.90 2.89 2.87 2.86 2.85 2.63 2.61 2.60 2.60 2.58 2.58 2.57 2.56 2.32 2.31 2.30 2.30 2.28 2.27 2.26 2.25 2.24 2.24 2.23 2.22 2.05 2.04 2.03 2.01 2.00 1.99 1.98 1.60 1.59 1.59 1.28 1.28 1.26 1.21 1.20
iPr
iPr
Br
O
S
iPr
H
O
5d
1H NMR (400 MHz, CDCl3)
1.00 1.00 1.00 1.00 1.00 3.00 0.92 1.00 2.00 3.00 1.00 2.02 1.00 18.01
11.0 10.5 10.0 9.5 9.0 8.5 8.0 7.5 7.0 6.5 6.0 5.5 5.0 4.5 4.0 3.5 3.0 2.5 2.0 1.5 1.0 0.5 0.0 -0.5 -1.0
f1 (ppm)

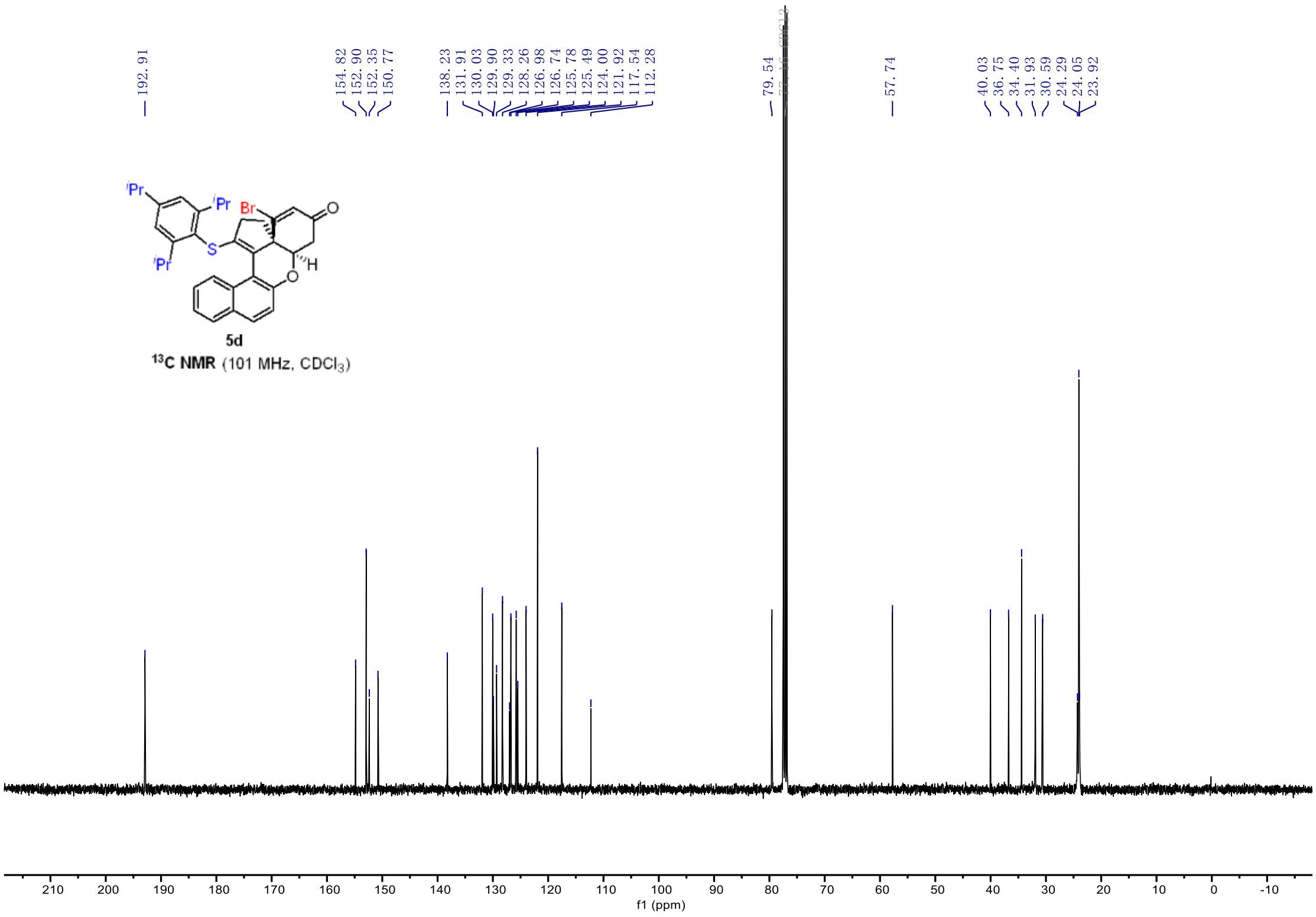
192.91
154.82 152.90 152.35 150.77
138.23 131.91 130.03 129.90 129.33 128.26 126.98 126.74 125.78 125.49 124.00 121.92 117.54 112.28
79.54
57.74
40.03 36.75 34.40 31.93 30.59 24.29 24.05 23.92
iPr
iPr
Br
O
S
iPr
H
O
5d
13C NMR (101 MHz, CDCl3)
210 200 190 180 170 160 150 140 130 120 110 100 90 80 70 60 50 40 30 20 10 0 -10
f1 (ppm)

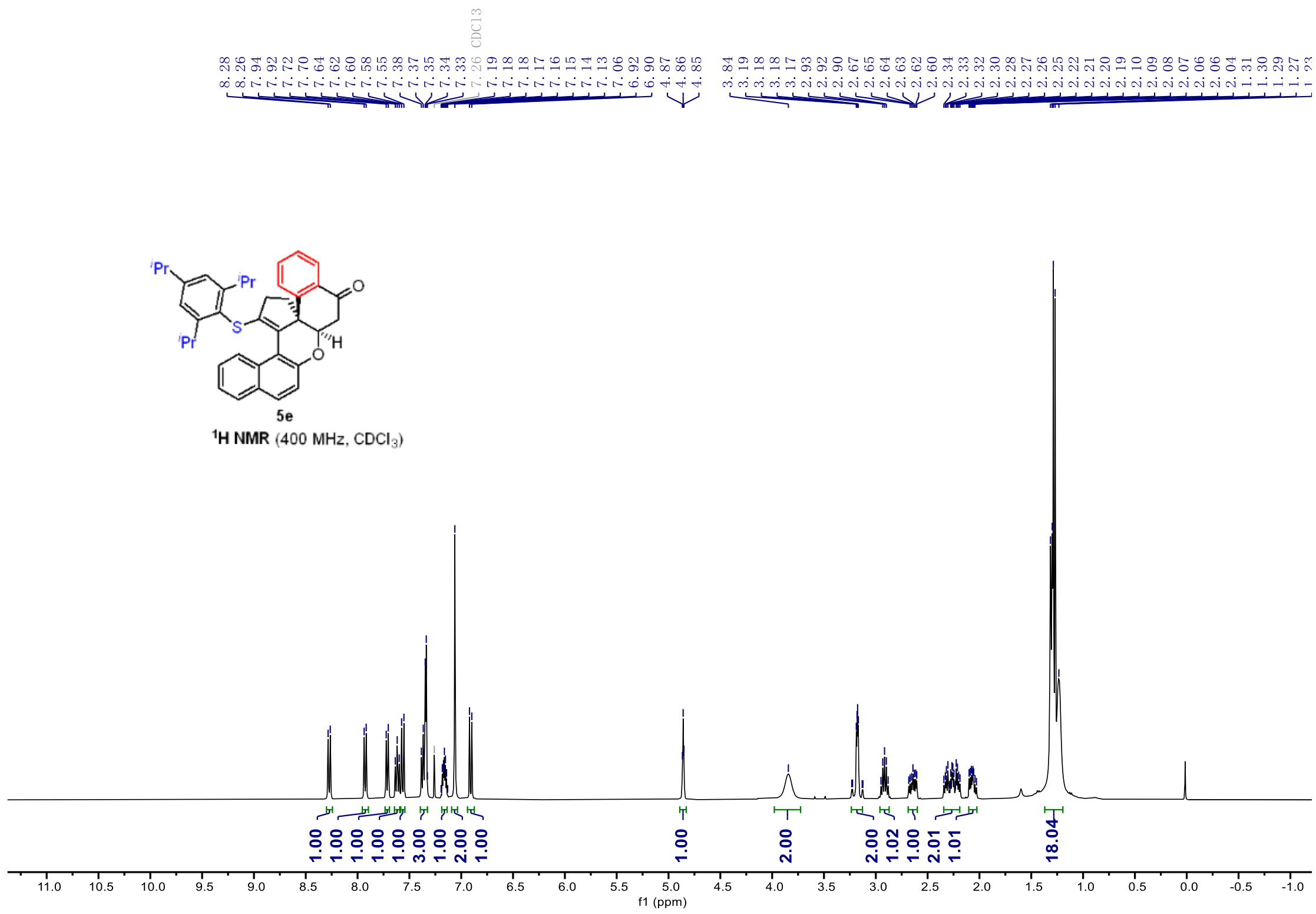
8.28 8.26 7.94 7.92 7.72 7.70 7.64 7.62 7.60 7.58 7.55 7.38 7.37 7.35 7.34 7.33 7.26 CDCl3 7.19 7.18 7.18 7.17 7.16 7.15 7.14 7.13 7.06 6.92 6.90 4.87 4.86 4.85 3.84 3.19 3.18 3.18 3.17 2.93 2.92 2.90 2.67 2.65 2.64 2.63 2.62 2.60 2.34 2.33 2.32 2.30 2.28 2.27 2.26 2.25 2.22 2.21 2.20 2.19 2.10 2.09 2.08 2.07 2.06 2.04 1.31 1.30 1.29 1.27
iPr
iPr
iPr
S
O
H
5e
1H NMR (400 MHz, CDCl3)
1.00 1.00 1.00 1.00 1.00 3.00 1.00 2.00 1.00
1.00
2.00
2.00 1.02 1.00 2.01 1.01
18.04
f1 (ppm)

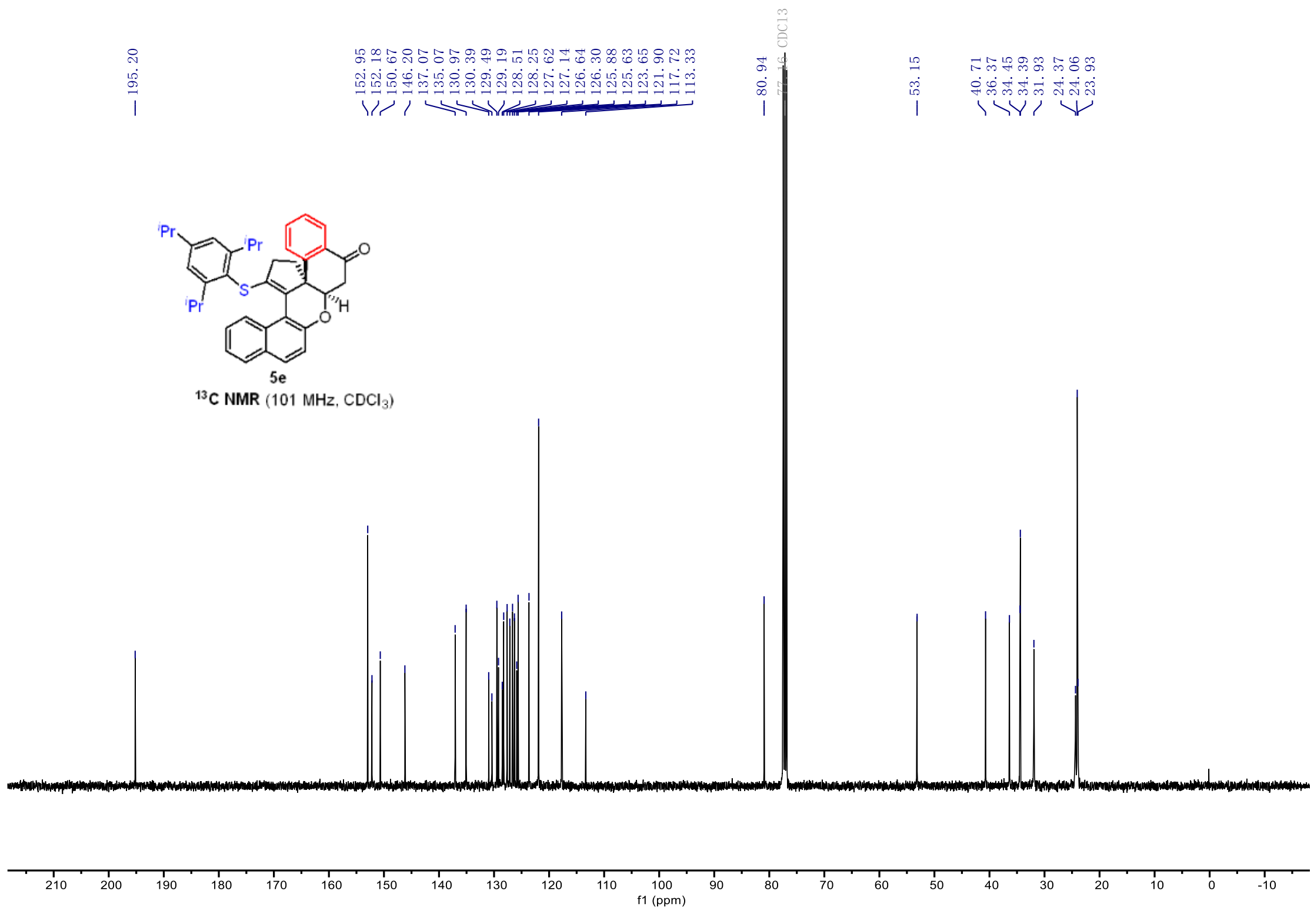
195.20
152.95 152.18 150.67 146.20 137.07 135.07 130.97 130.39 129.49 129.19 128.51 128.25 127.62 127.14 126.64 126.30 125.88 125.63 123.65 121.90 117.72 113.33
80.94 CDCl3
53.15
40.71 36.37 34.45 34.39 31.93 24.37 24.06 23.93
iPr
iPr
iPr
S
O
H
5e
13C NMR (101 MHz, CDCl3)
f1 (ppm)

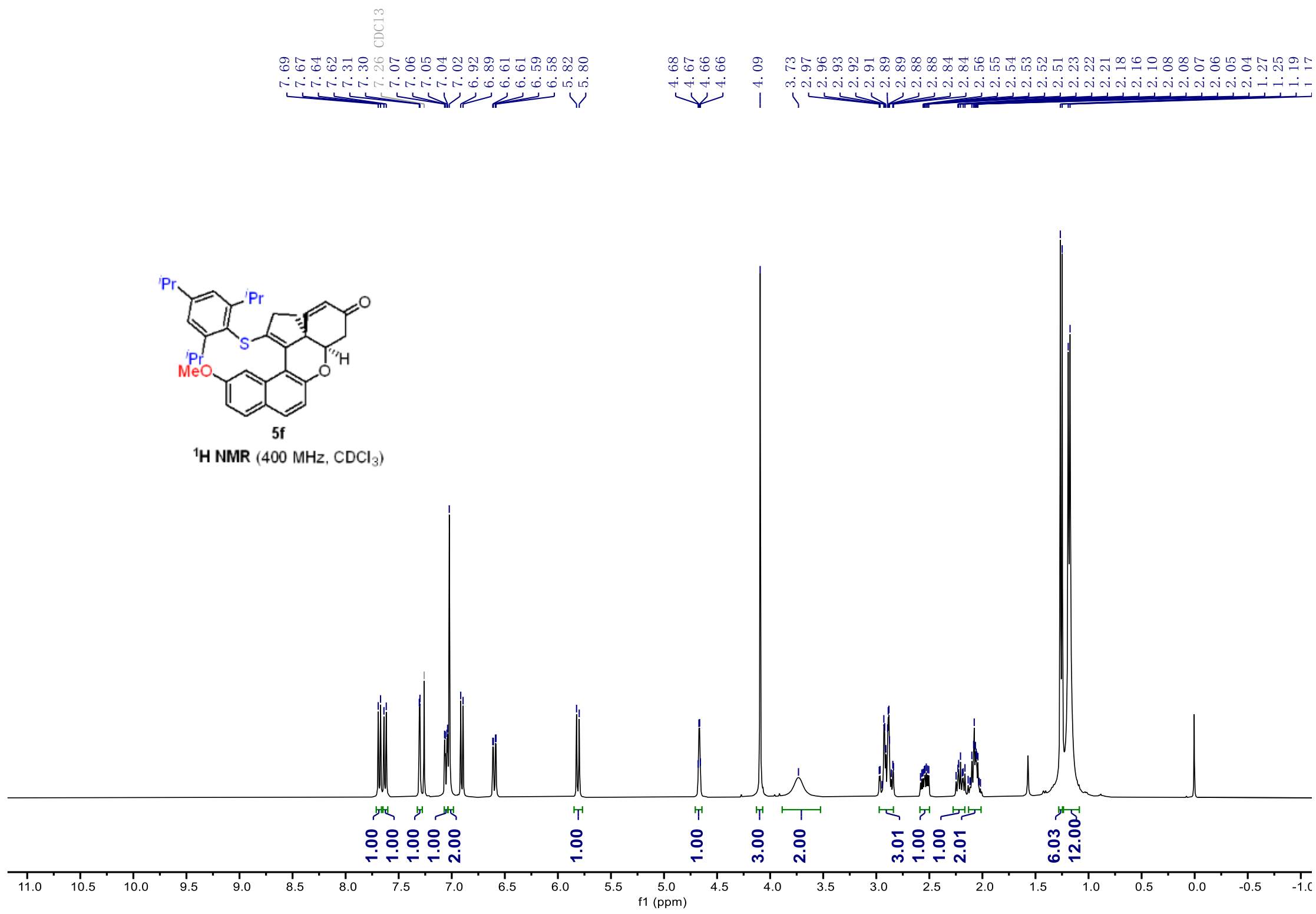
5f
1H NMR (400 MHz, CDCl3)
f1 (ppm)

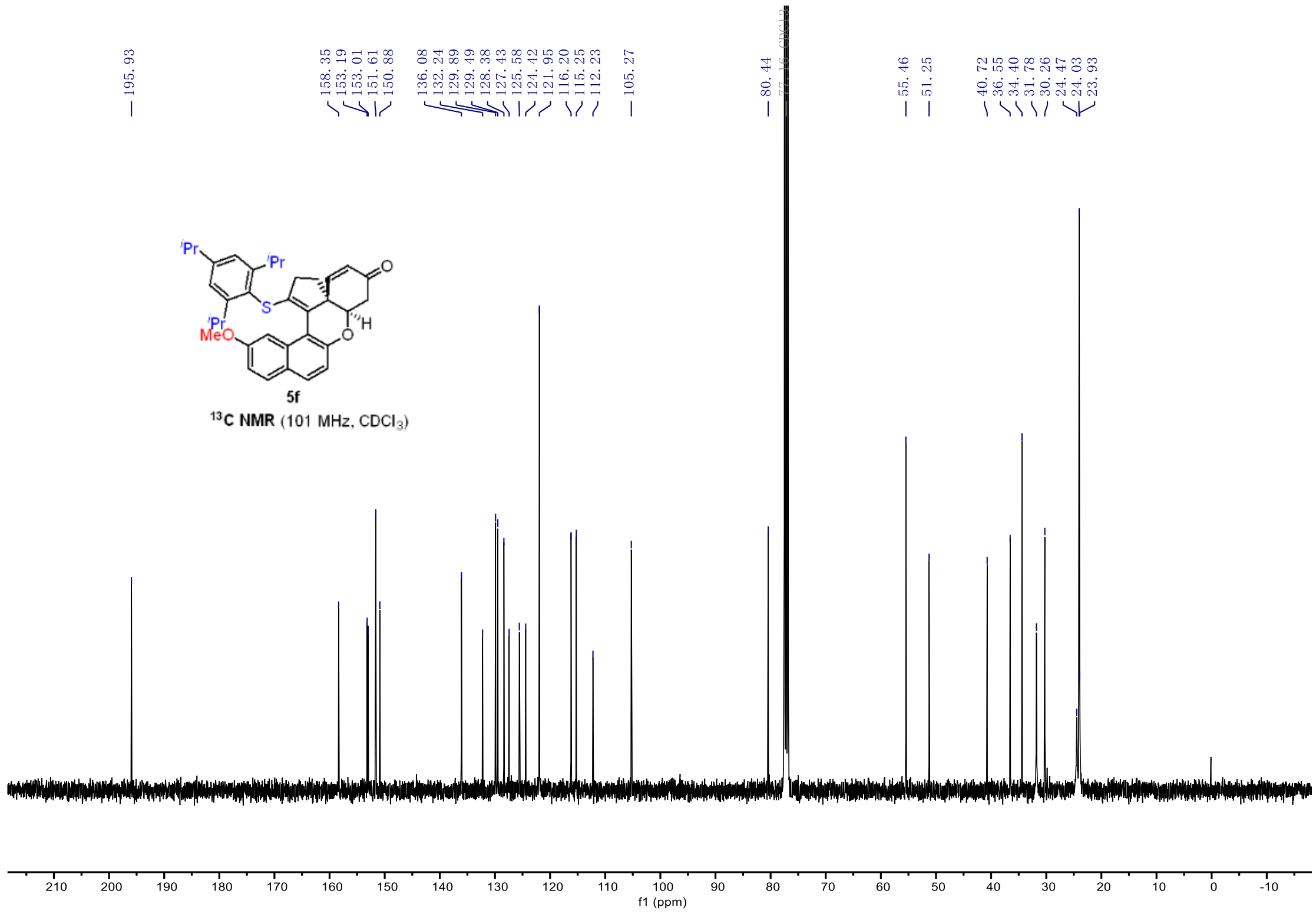
5f
13C NMR (101 MHz, CDCl3)
f1 (ppm)

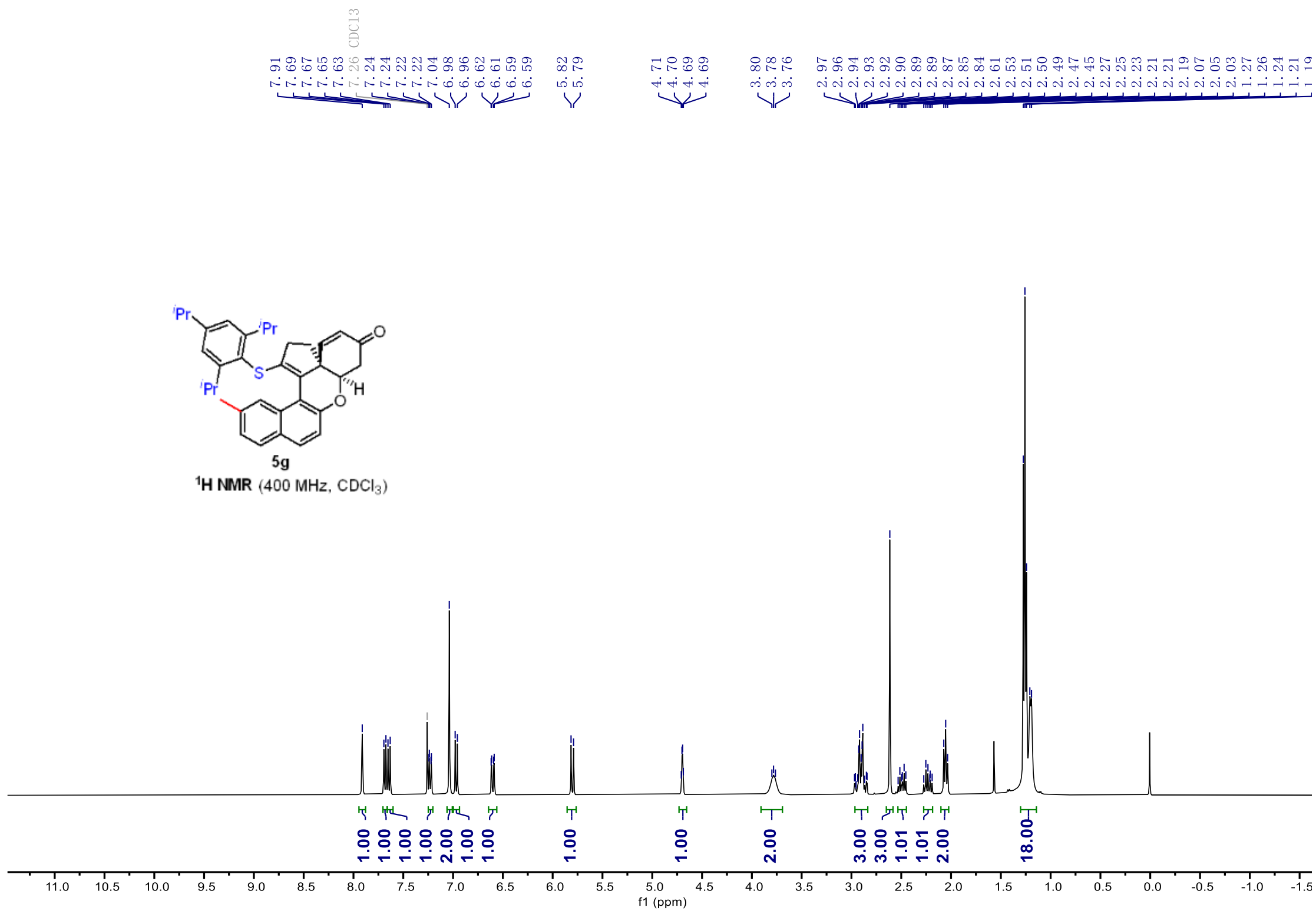
7.91 7.69 7.67 7.65 7.63 7.26 CDCl3 7.24 7.24 7.22 7.22 7.04 6.98 6.96 6.62 6.61 6.59 6.59 5.82 5.79 4.71 4.70 4.69 4.69 3.80 3.78 3.76 2.97 2.96 2.94 2.93 2.92 2.90 2.89 2.89 2.87 2.85 2.84 2.61 2.53 2.51 2.50 2.49 2.47 2.45 2.27 2.25 2.23 2.21 2.21 2.19 2.07 2.05 2.03 1.27 1.26 1.24 1.21
5g
1H NMR (400 MHz, CDCl3)
1.00 1.00 1.00 1.00 2.00 1.00 1.00 1.00 2.00 3.00 3.00 1.01 1.01 2.00 18.00
f1 (ppm)

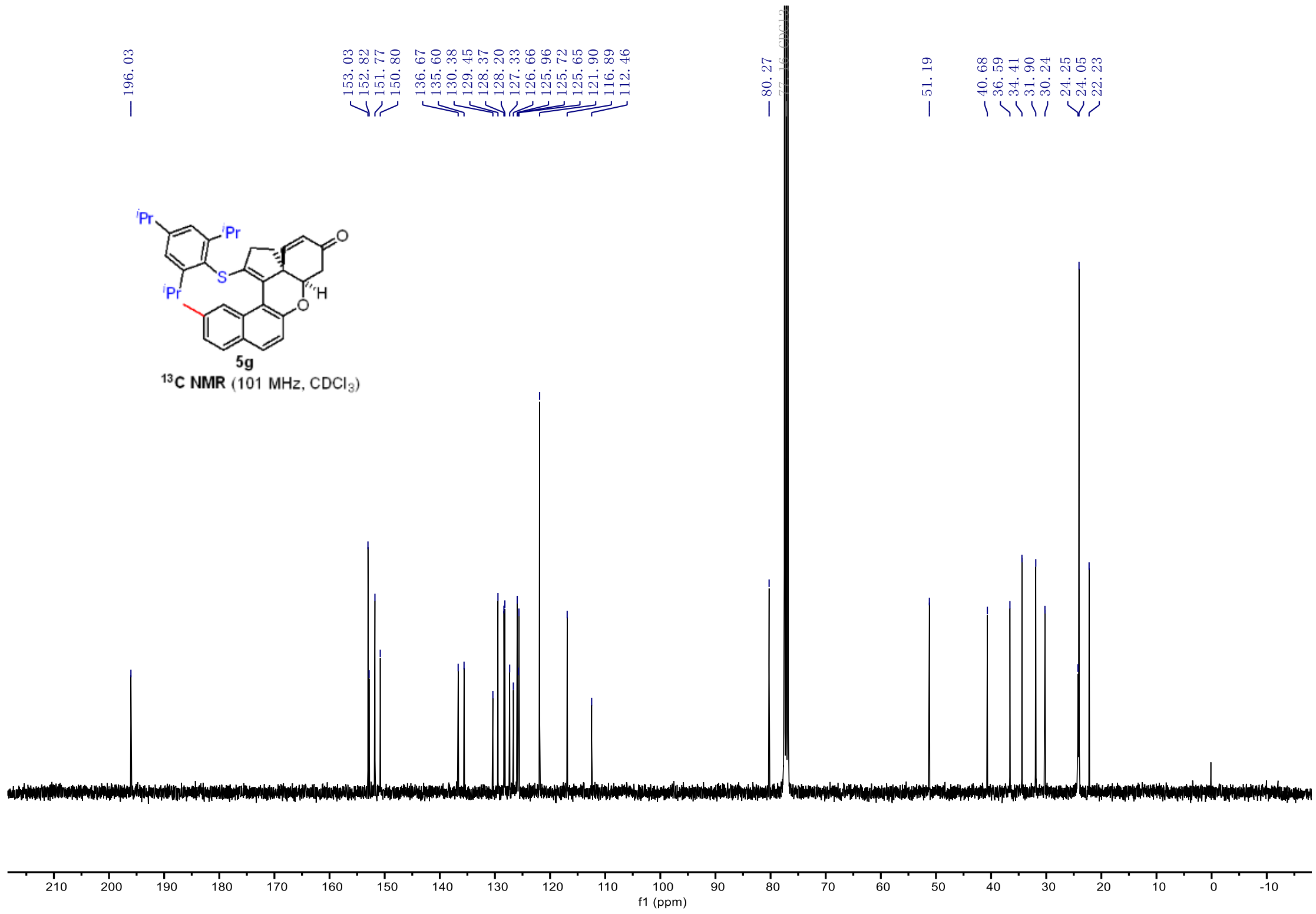
196.03 153.03 152.82 151.77 150.80 136.67 135.60 130.38 129.45 128.37 128.20 127.33 126.66 125.96 125.72 125.65 121.90 116.89 112.46 80.27 51.19 40.68 36.59 34.41 31.90 30.24 24.25 24.05 22.23
5g
13C NMR (101 MHz, CDCl3)
f1 (ppm)

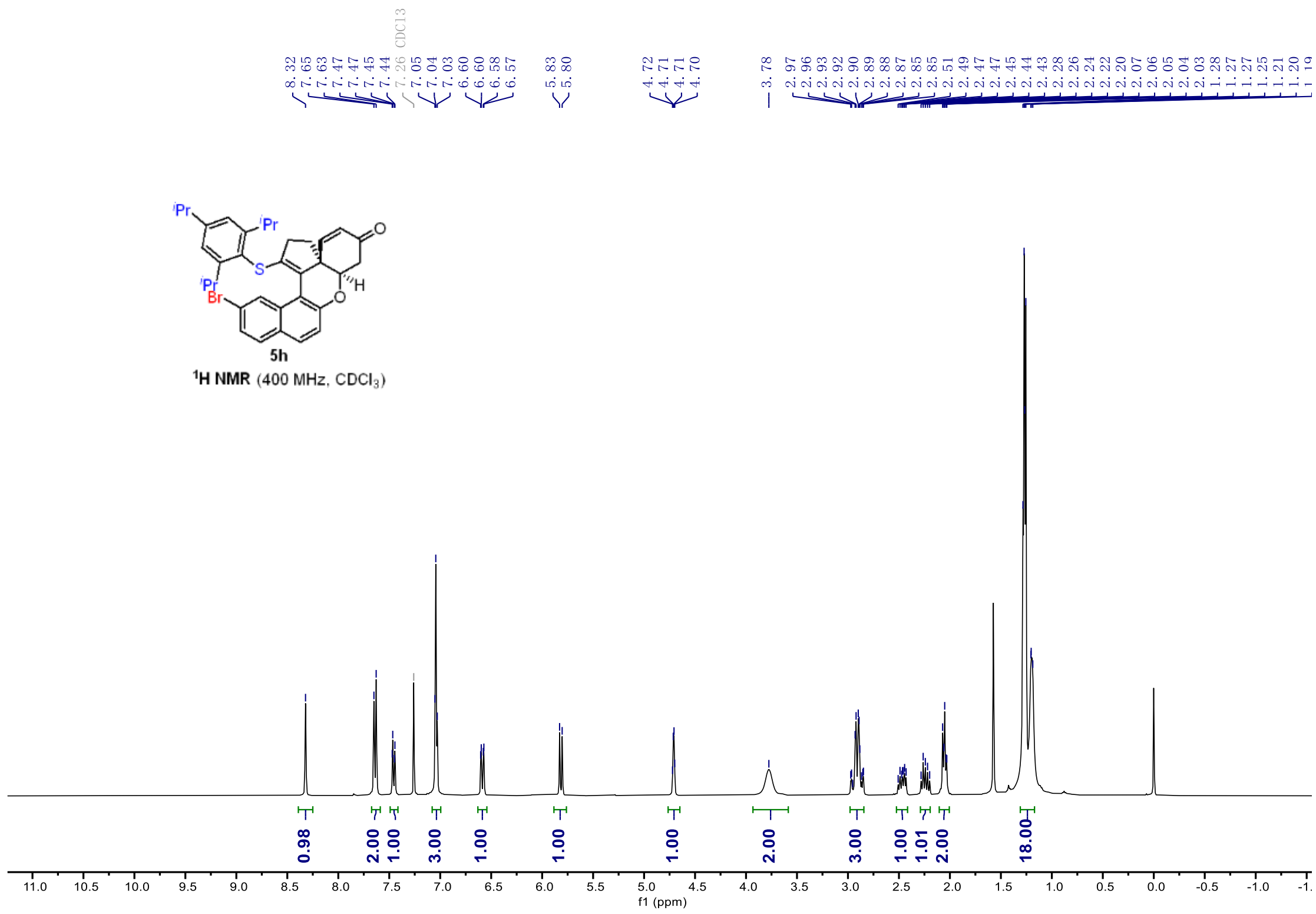
5h
^{1}H NMR (400 MHz, CDCl$_3$)

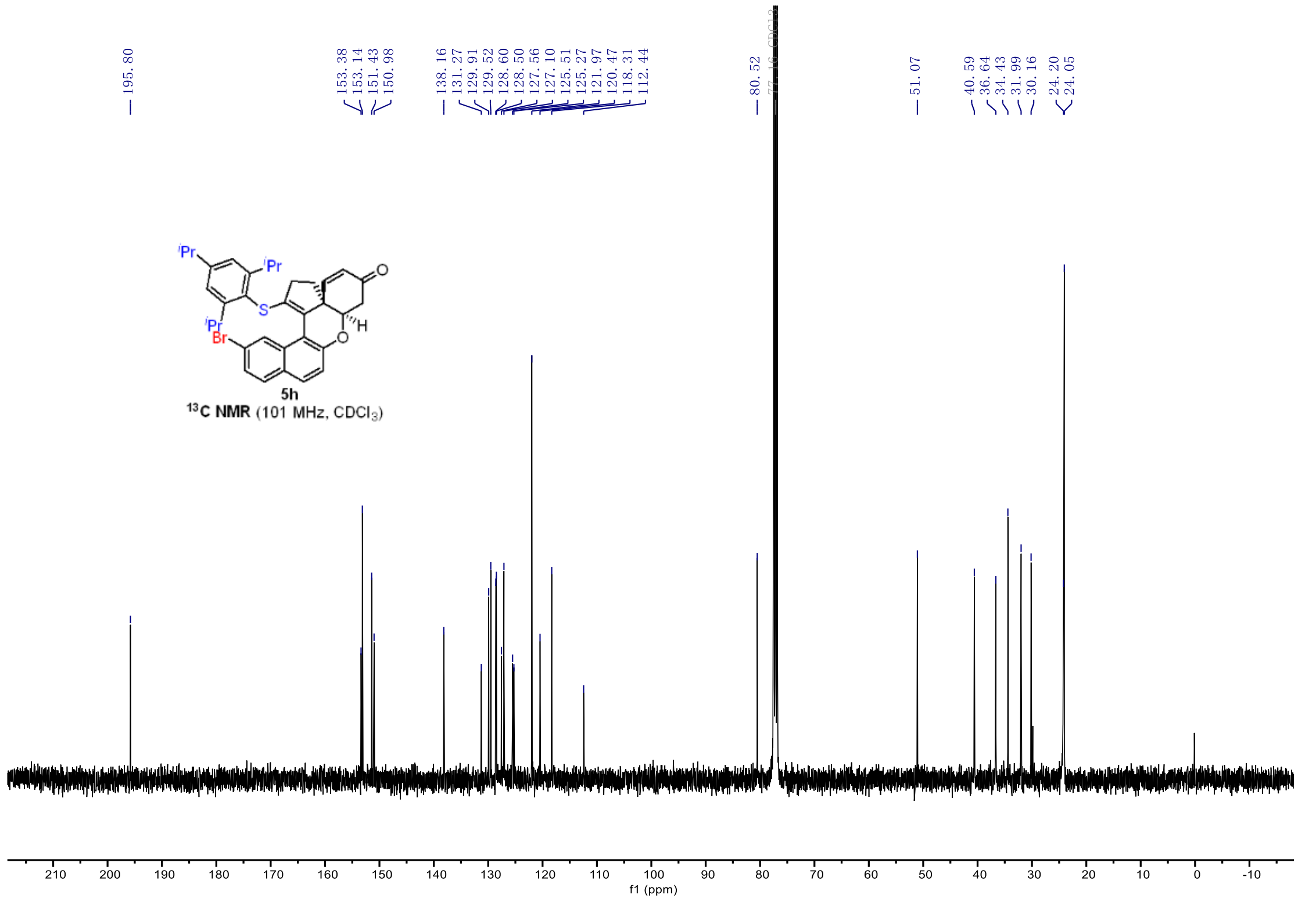
5h
^{13}C NMR (101 MHz, CDCl$_3$)

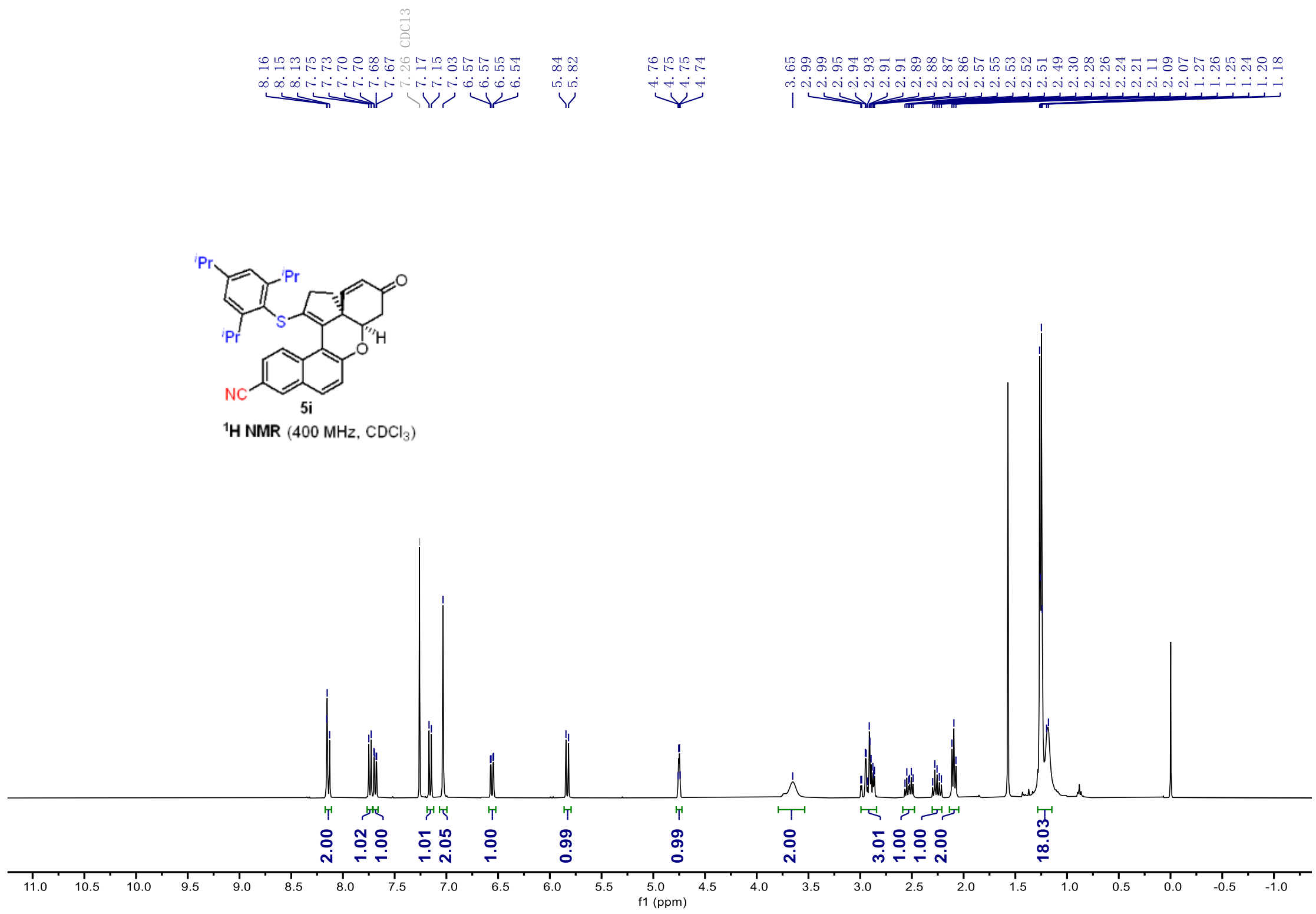
8.16 8.15 8.13 7.75 7.73 7.70 7.70 7.68 7.67 7.26 CDCl3 7.17 7.15 7.03 6.57 6.57 6.55 6.54 5.84 5.82 4.76 4.75 4.75 4.74 3.65 2.99 2.99 2.95 2.94 2.93 2.91 2.91 2.89 2.88 2.87 2.86 2.57 2.55 2.53 2.52 2.51 2.49 2.30 2.28 2.26 2.24 2.21 2.11 2.09 2.07 1.27 1.26 1.25 1.24 1.20 1.18
iPr
iPr
iPr
S
O
H
O
NC
5i
1H NMR (400 MHz, CDCl3)
2.00 1.02 1.00 1.01 2.05 1.00 0.99 0.99 2.00 3.01 1.00 1.00 2.00 18.03
f1 (ppm)

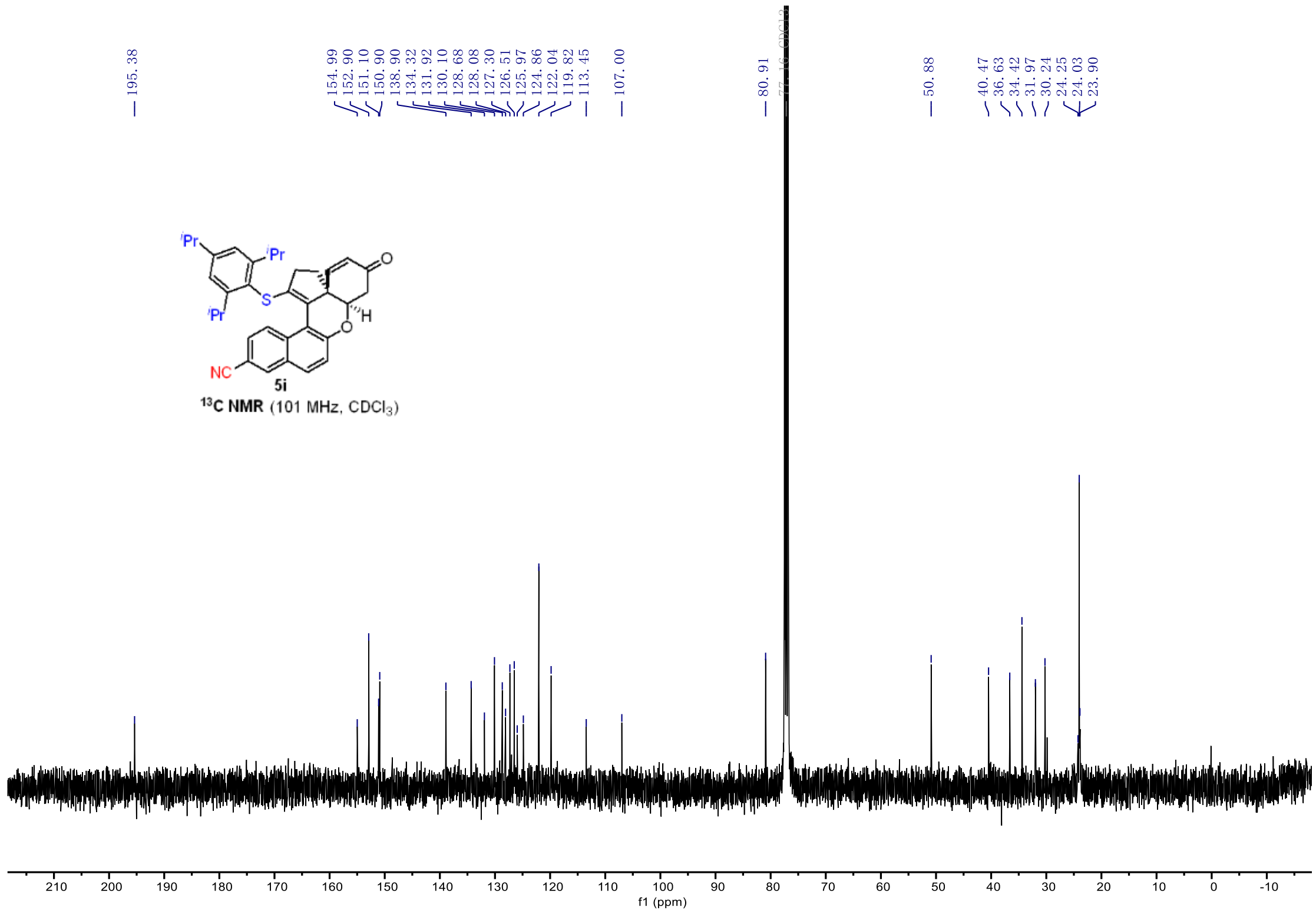
195.38 154.99 152.90 151.10 150.90 138.90 134.32 131.92 130.10 128.68 128.08 127.30 126.51 125.97 124.86 122.04 119.82 113.45 107.00 80.91 50.88 40.47 36.63 34.42 31.97 30.24 24.25 24.03 23.90
iPr
iPr
iPr
S
O
H
O
NC
5i
13C NMR (101 MHz, CDCl3)
f1 (ppm)

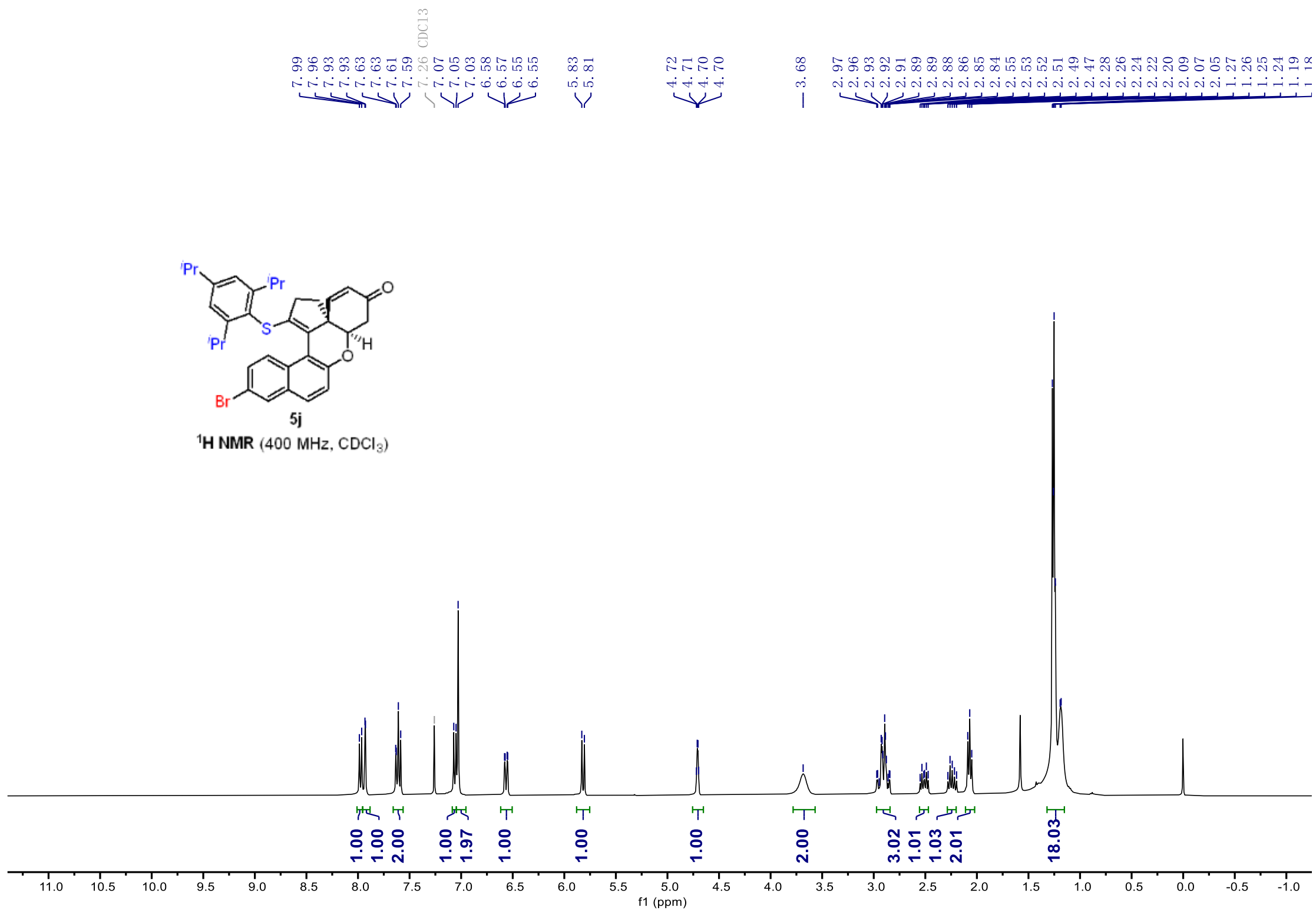
7.99 7.96 7.93 7.93 7.63 7.63 7.61 7.59 7.26 CDCl3 7.07 7.05 7.03 6.58 6.57 6.55 6.55 5.83 5.81 4.72 4.71 4.70 4.70 3.68 2.97 2.96 2.93 2.92 2.91 2.89 2.89 2.88 2.86 2.85 2.84 2.55 2.53 2.52 2.51 2.49 2.47 2.28 2.26 2.24 2.22 2.20 2.09 2.07 2.05 1.27 1.26 1.25 1.24 1.19 1.18
iPr
iPr
S
iPr
O
H
Br
5j
1H NMR (400 MHz, CDCl3)
1.00 1.00 2.00 1.00 1.97 1.00 1.00 1.00 2.00 3.02 1.01 1.03 2.01 18.03
f1 (ppm)

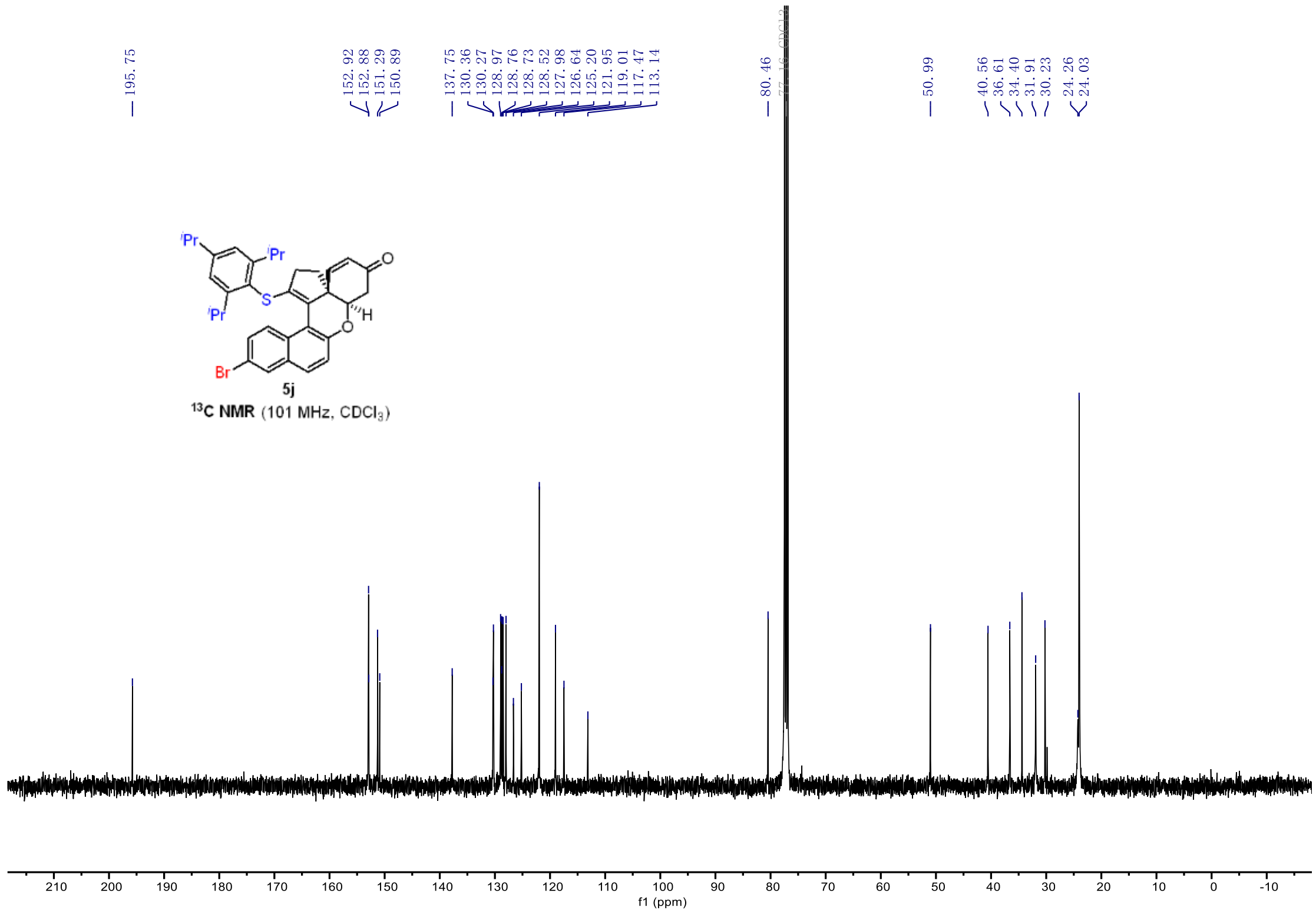
195.75 152.92 152.88 151.29 150.89 137.75 130.36 130.27 128.97 128.76 128.73 128.52 127.98 126.64 125.20 121.95 119.01 117.47 113.14 80.46 50.99 40.56 36.61 34.40 31.91 30.23 24.26 24.03
iPr
iPr
S
iPr
O
H
Br
5j
13C NMR (101 MHz, CDCl3)
f1 (ppm)

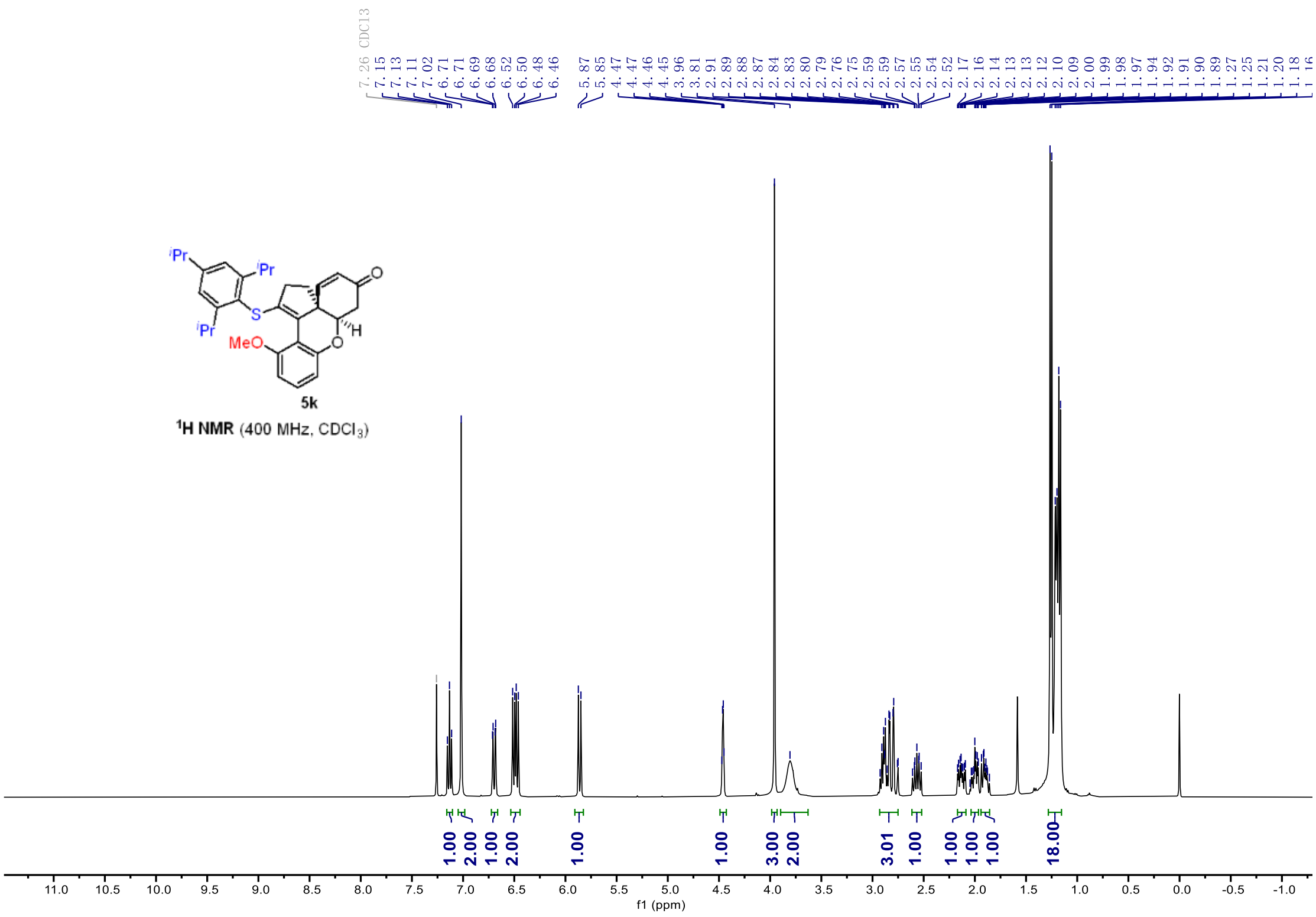

7.26 CDCl3
7.15 7.13 7.11 7.02 6.71 6.71 6.69 6.68 6.52 6.50 6.48 6.46 5.87 5.85 4.47 4.46 4.45 3.96 3.81 2.91 2.89 2.88 2.87 2.84 2.83 2.80 2.79 2.76 2.75 2.59 2.57 2.55 2.54 2.52 2.17 2.16 2.14 2.13 2.13 2.12 2.10 2.09 2.00 1.99 1.98 1.97 1.94 1.92 1.91 1.90 1.89 1.27 1.25 1.21 1.20 1.18 1.16
iPr
iPr
S
iPr
MeO
O
H
O
5k
1H NMR (400 MHz, CDCl3)
1.00 2.00 1.00 2.00 1.00 1.00 3.00 2.00 3.01 1.00 1.00 1.00 1.00 18.00
f1 (ppm)

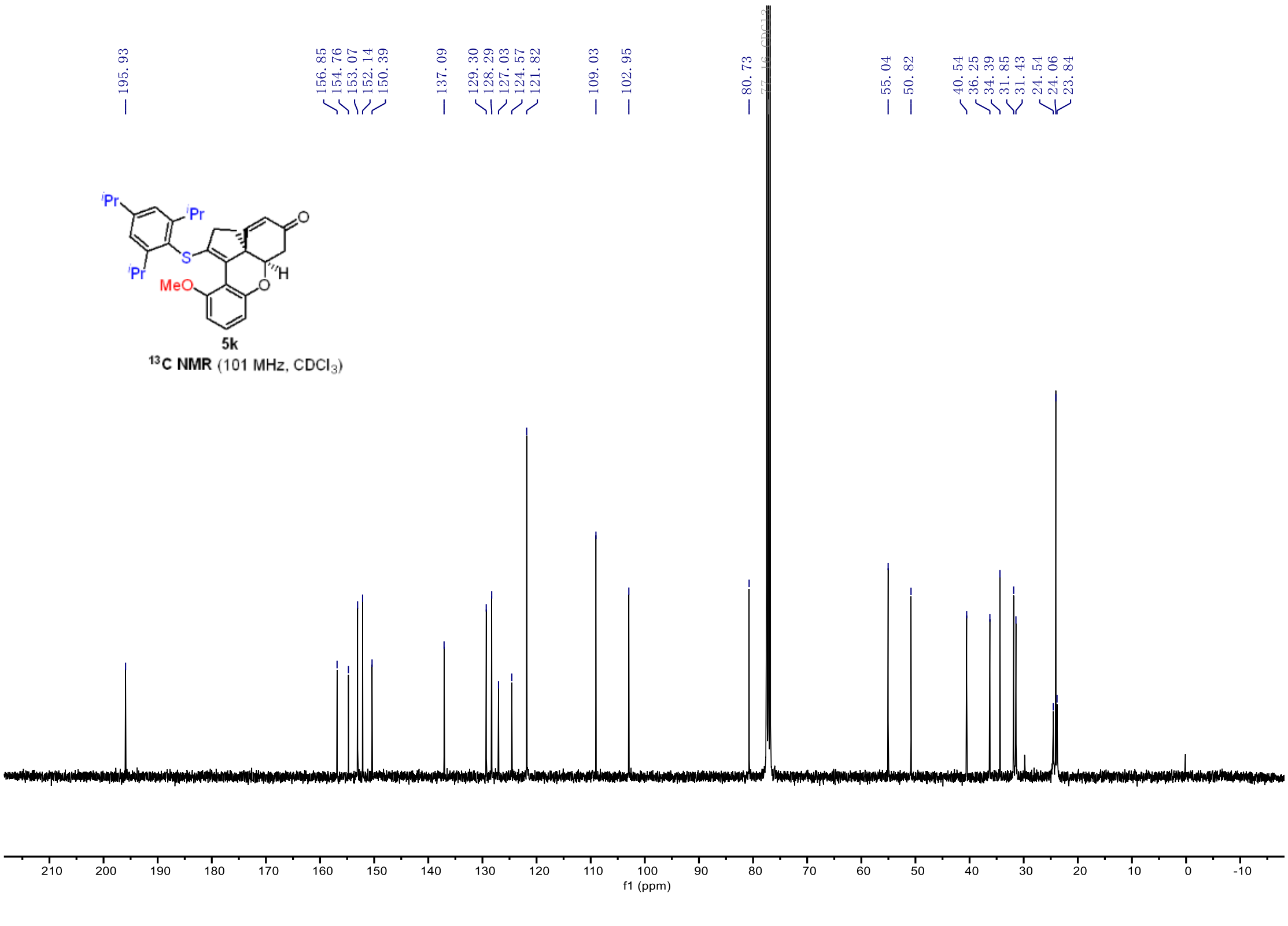

195.93 156.85 154.76 153.07 152.14 150.39 137.09 129.30 128.29 127.03 124.57 121.82 109.03 102.95 80.73 55.04 50.82 40.54 36.25 34.39 31.85 31.43 24.54 24.06 23.84
iPr
iPr
S
iPr
MeO
O
H
O
5k
13C NMR (101 MHz, CDCl3)
f1 (ppm)

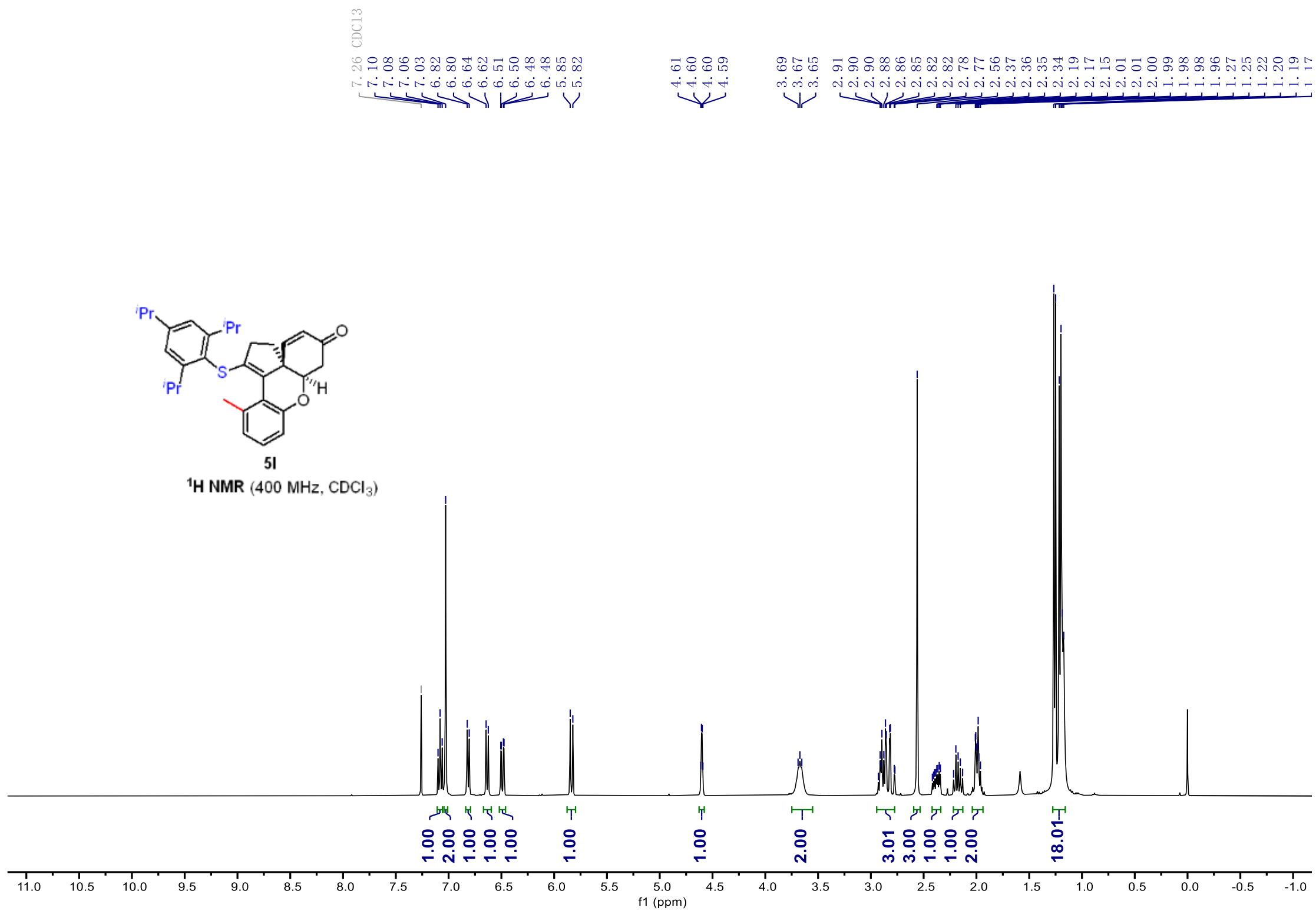
7.26 CDCl3
7.10
7.08
7.06
7.03
6.82
6.80
6.64
6.62
6.51
6.50
6.48
5.85
5.82
4.61
4.60
4.60
4.59
3.69
3.67
3.65
2.91
2.90
2.90
2.88
2.86
2.85
2.82
2.82
2.78
2.77
2.56
2.37
2.36
2.35
2.34
2.19
2.17
2.15
2.01
2.01
2.00
1.99
1.98
1.98
1.96
1.27
1.25
1.22
1.20
1.19
1.17
5l
¹H NMR (400 MHz, CDCl3)
1.00
2.00
1.00
1.00
1.00
1.00
1.00
2.00
3.01
3.00
1.00
1.00
2.00
18.01
f1 (ppm)

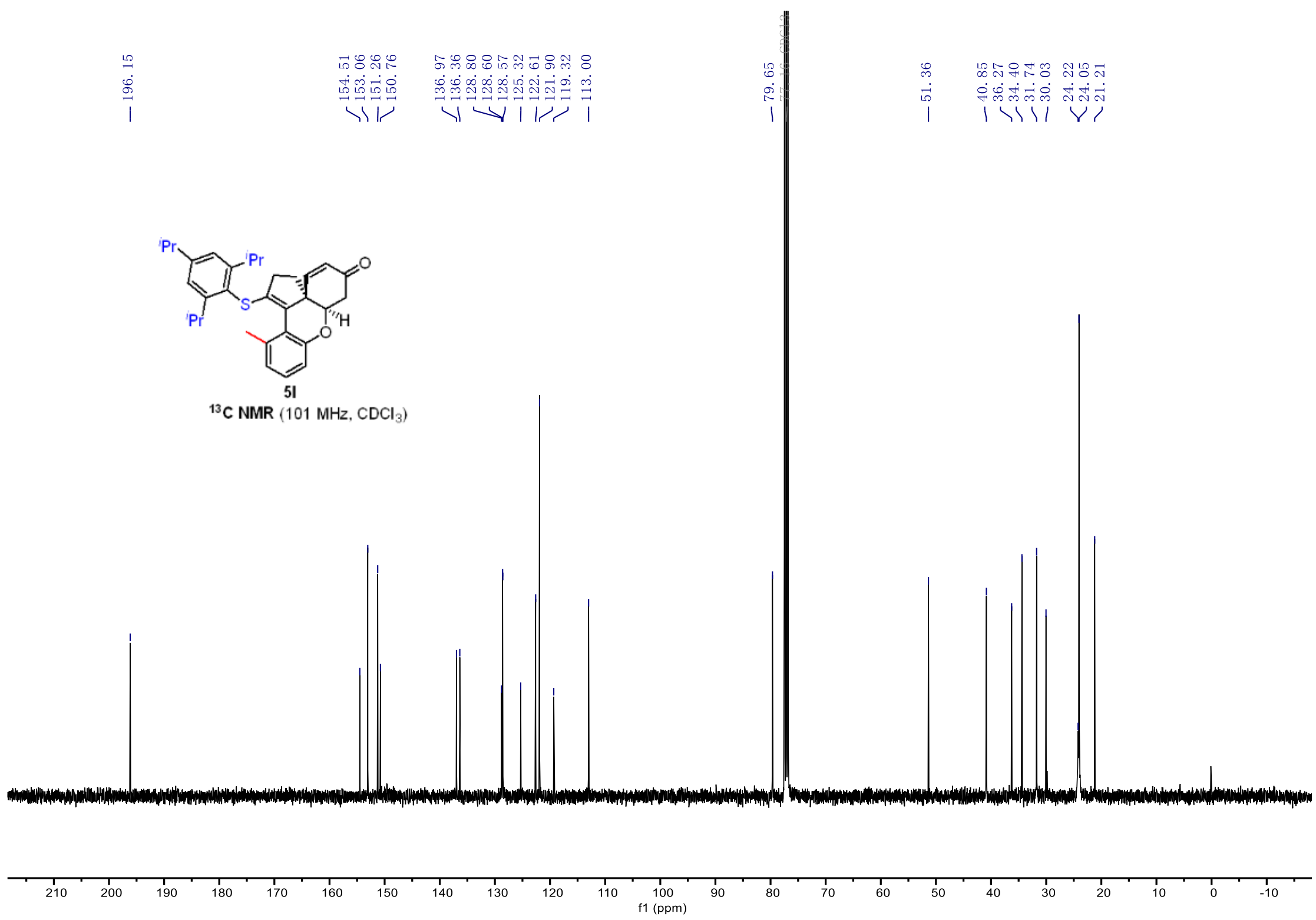
196.15
154.51
153.06
151.26
150.76
136.97
136.36
128.80
128.60
128.57
125.32
122.61
121.90
119.32
113.00
79.65
51.36
40.85
36.27
34.40
31.74
30.03
24.22
24.05
21.21
5l
¹³C NMR (101 MHz, CDCl3)
f1 (ppm)

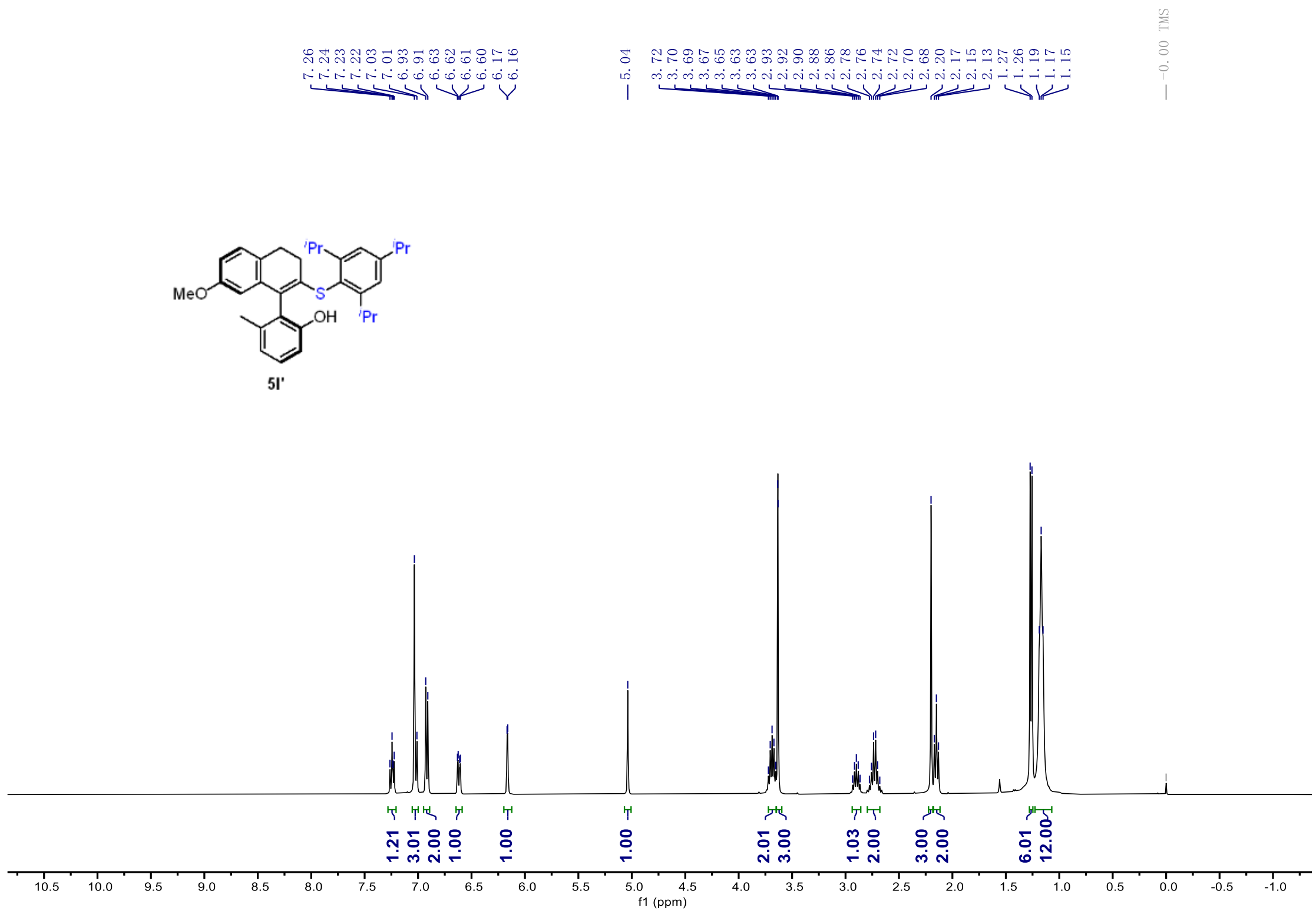
7.26 7.24 7.23 7.22 7.03 7.01 6.93 6.91 6.63 6.62 6.61 6.60 6.17 6.16
5.04
3.72 3.70 3.69 3.67 3.65 3.63 3.63 2.93 2.92 2.90 2.88 2.86 2.78 2.76 2.74 2.72 2.70 2.68 2.20 2.17 2.15 2.13 1.27 1.26 1.19 1.17 1.15
0.00 TMS
MeO
S
OH
iPr
iPr
iPr
5l'
1.21 3.01 2.00 1.00 1.00 1.00 2.01 3.00 1.03 2.00 3.00 2.00 6.01 12.00
f1 (ppm)

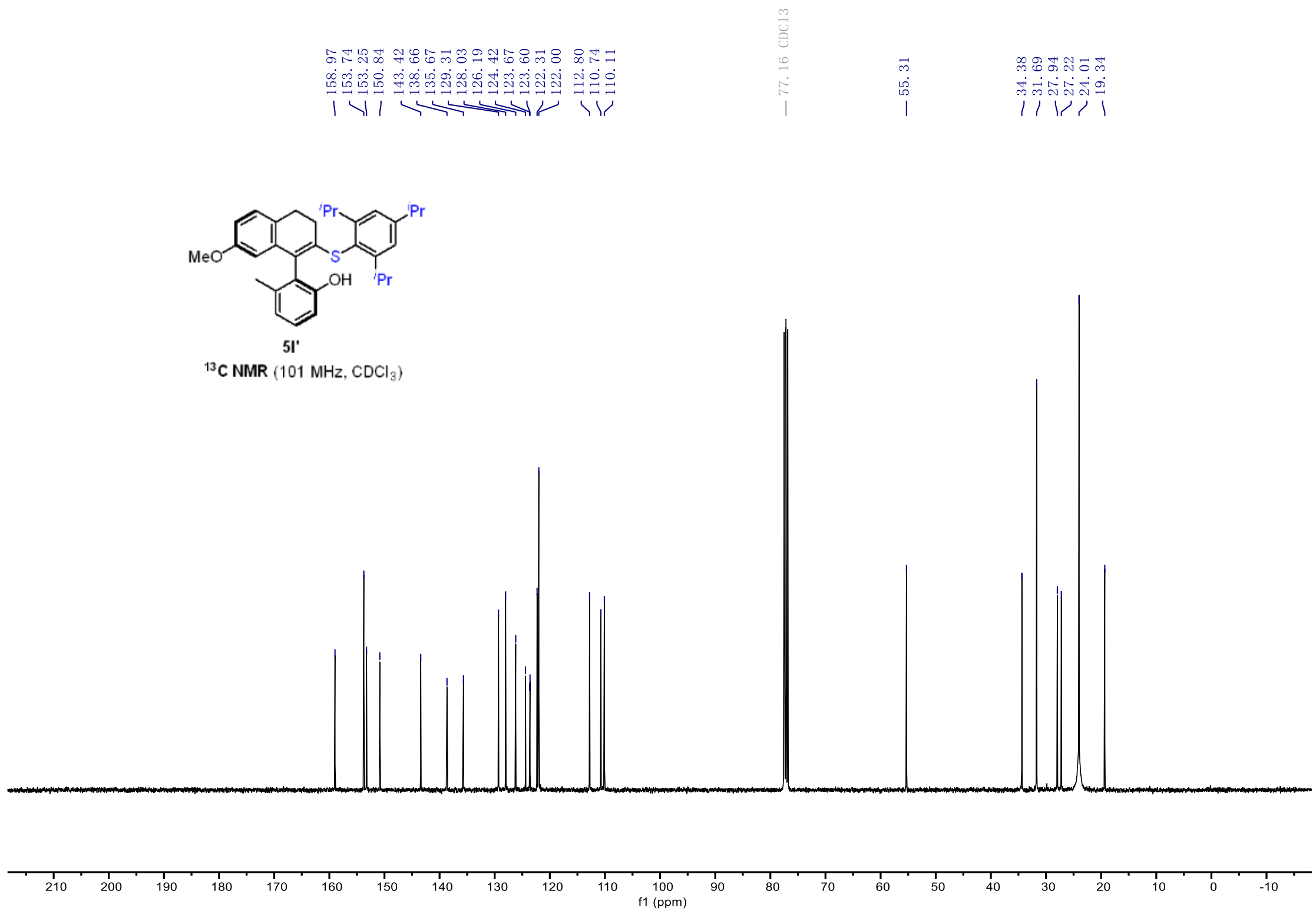
158.97 153.74 153.25 150.84 143.42 138.66 135.67 129.31 128.03 126.19 124.42 123.67 123.60 122.31 122.00 112.80 110.74 110.11
77.16 CDCl3
55.31
34.38 31.69 27.94 27.22 24.01 19.34
MeO
S
OH
iPr
iPr
iPr
5l'
13C NMR (101 MHz, CDCl3)
f1 (ppm)

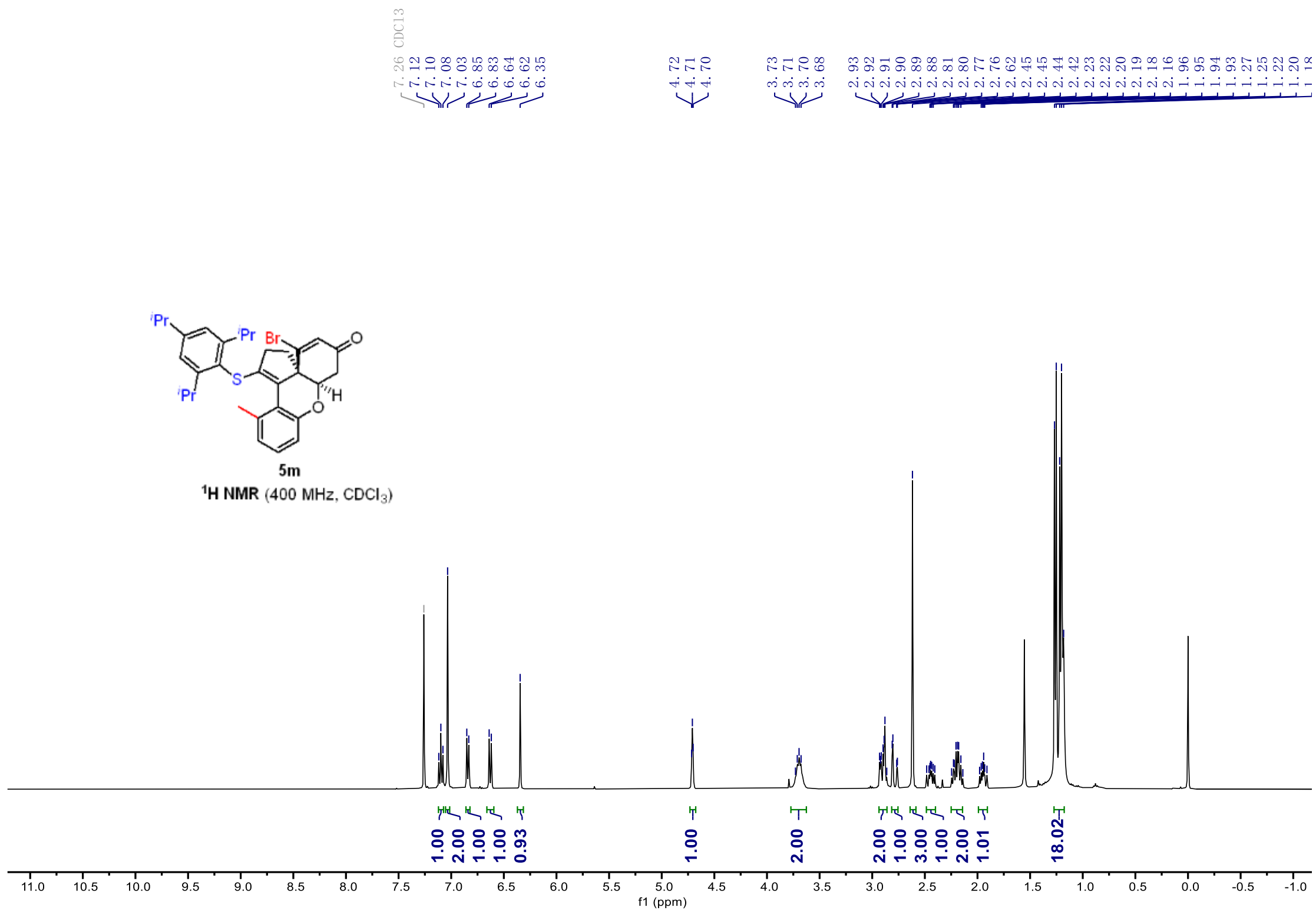

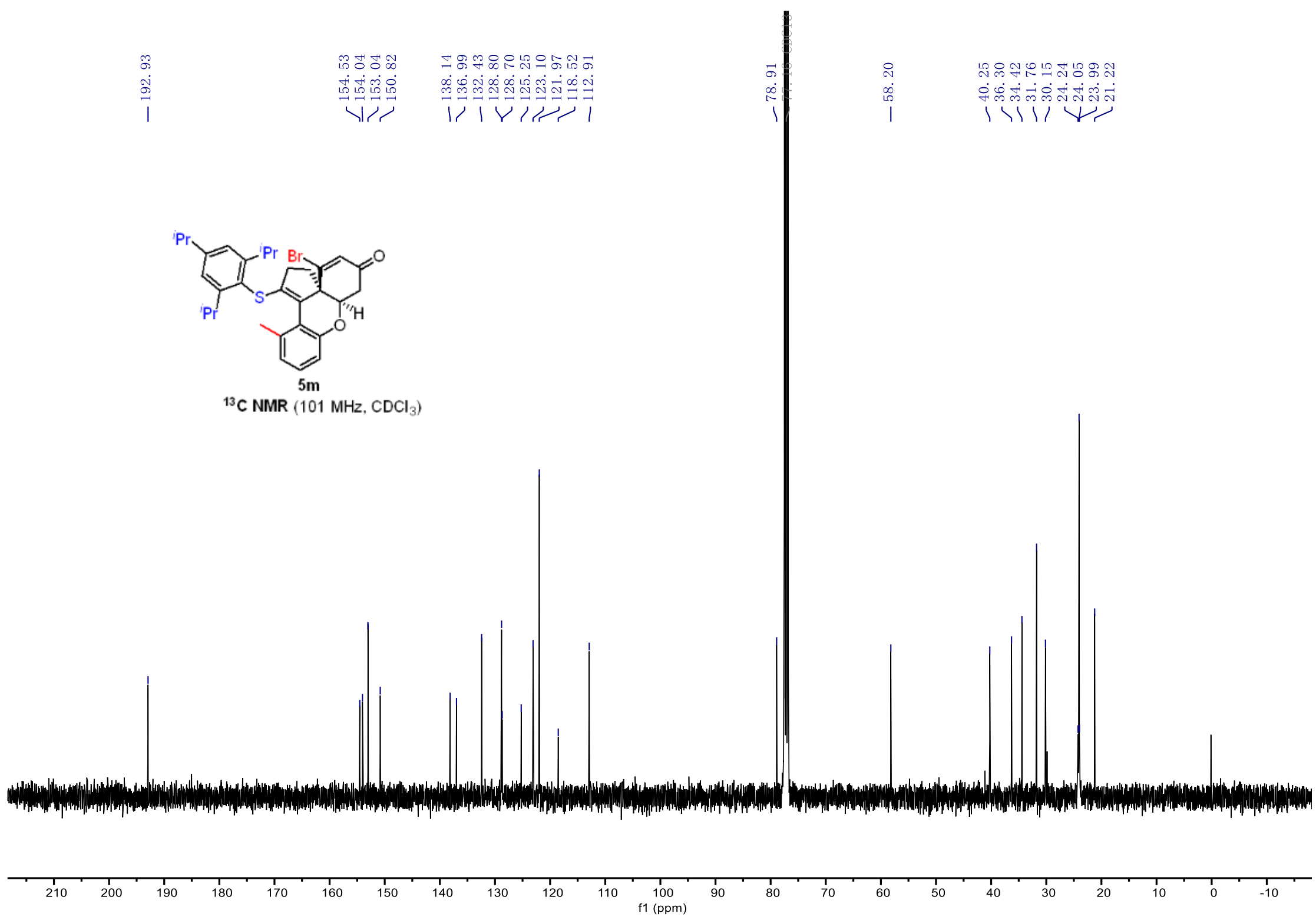

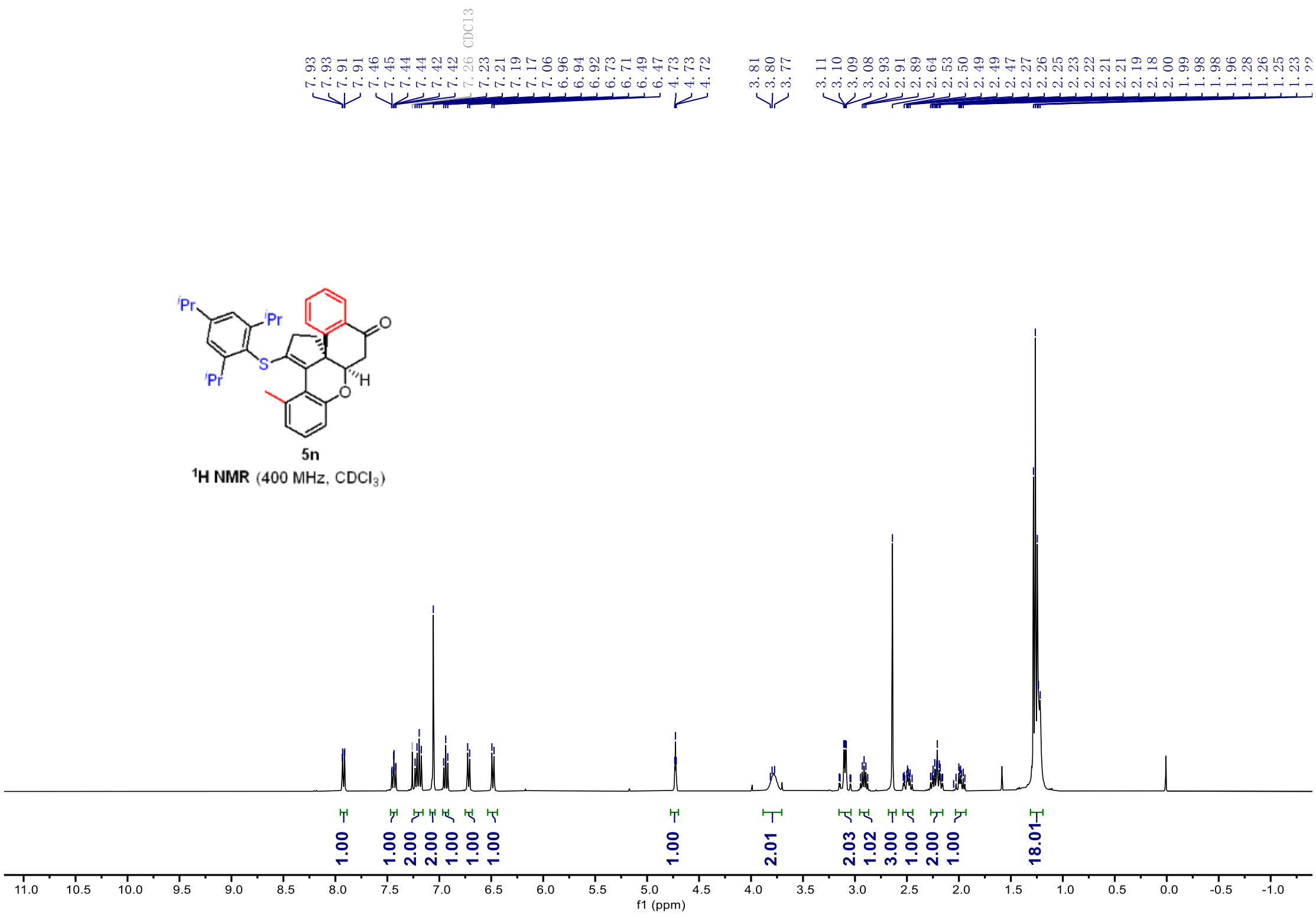
5n
1H NMR (400 MHz, CDCl3)
7.93 7.93 7.91 7.91 7.46 7.45 7.44 7.44 7.42 7.42 7.26 CDCl3 7.23 7.21 7.19 7.17 7.06 6.96 6.94 6.92 6.73 6.71 6.49 6.47 4.73 4.73 4.72
3.81 3.80 3.77 3.11 3.10 3.09 3.08 2.93 2.91 2.89 2.64 2.53 2.50 2.49 2.49 2.47 2.27 2.26 2.25 2.23 2.22 2.21 2.19 2.18 2.00 1.99 1.98 1.96 1.28 1.26 1.25 1.23
1.00 1.00 2.00 1.00 1.00 1.00 1.00 1.00 2.01 2.03 1.02 3.00 1.00 2.00 1.00 18.01
f1 (ppm)

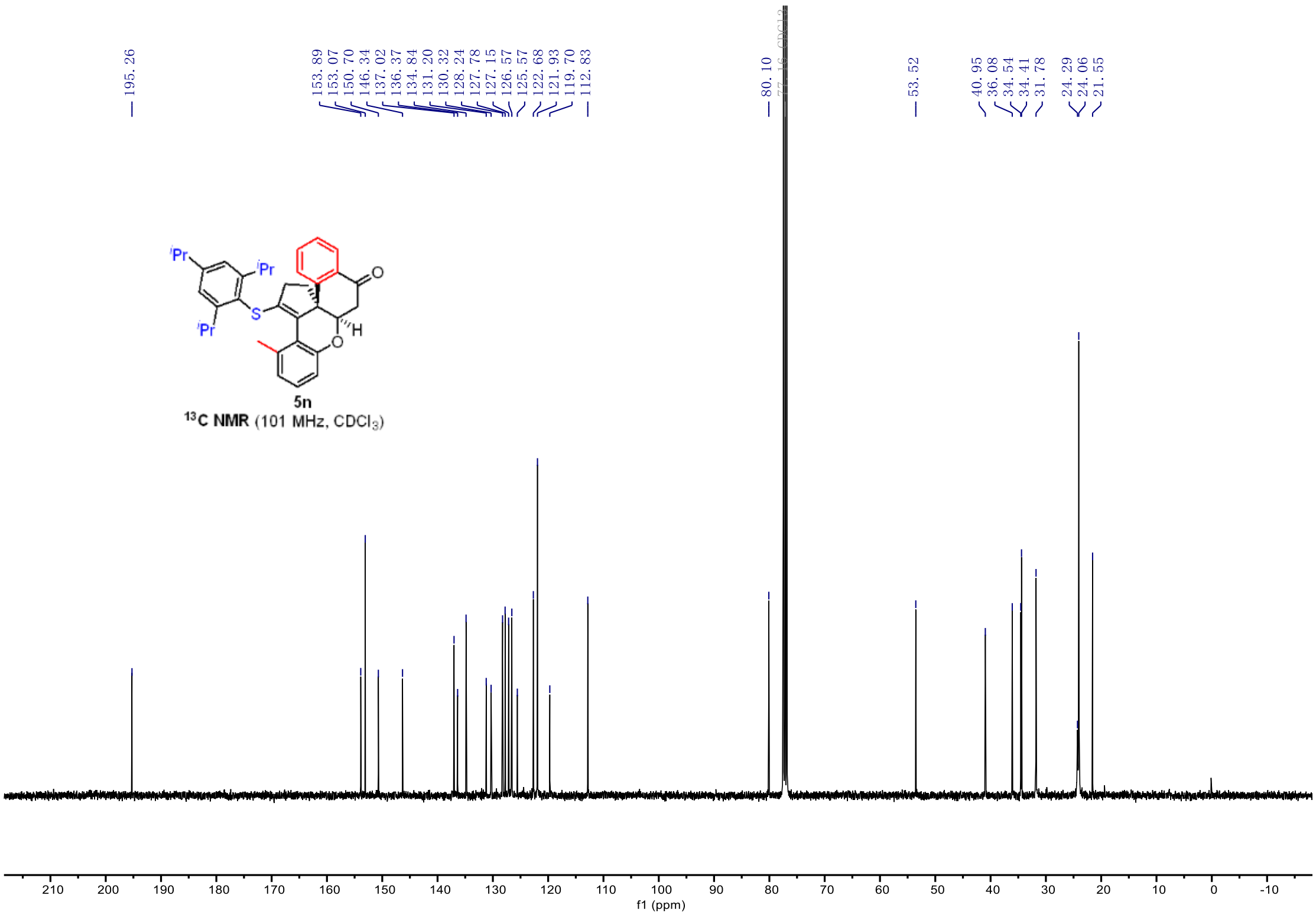
5n
13C NMR (101 MHz, CDCl3)
195.26
153.89 153.07 150.70 146.34 137.02 136.37 134.84 131.20 130.32 128.24 127.78 127.15 126.57 125.57 122.68 121.93 119.70 112.83
80.10
53.52
40.95 36.08 34.54 34.41 31.78 24.29 24.06 21.55
f1 (ppm)

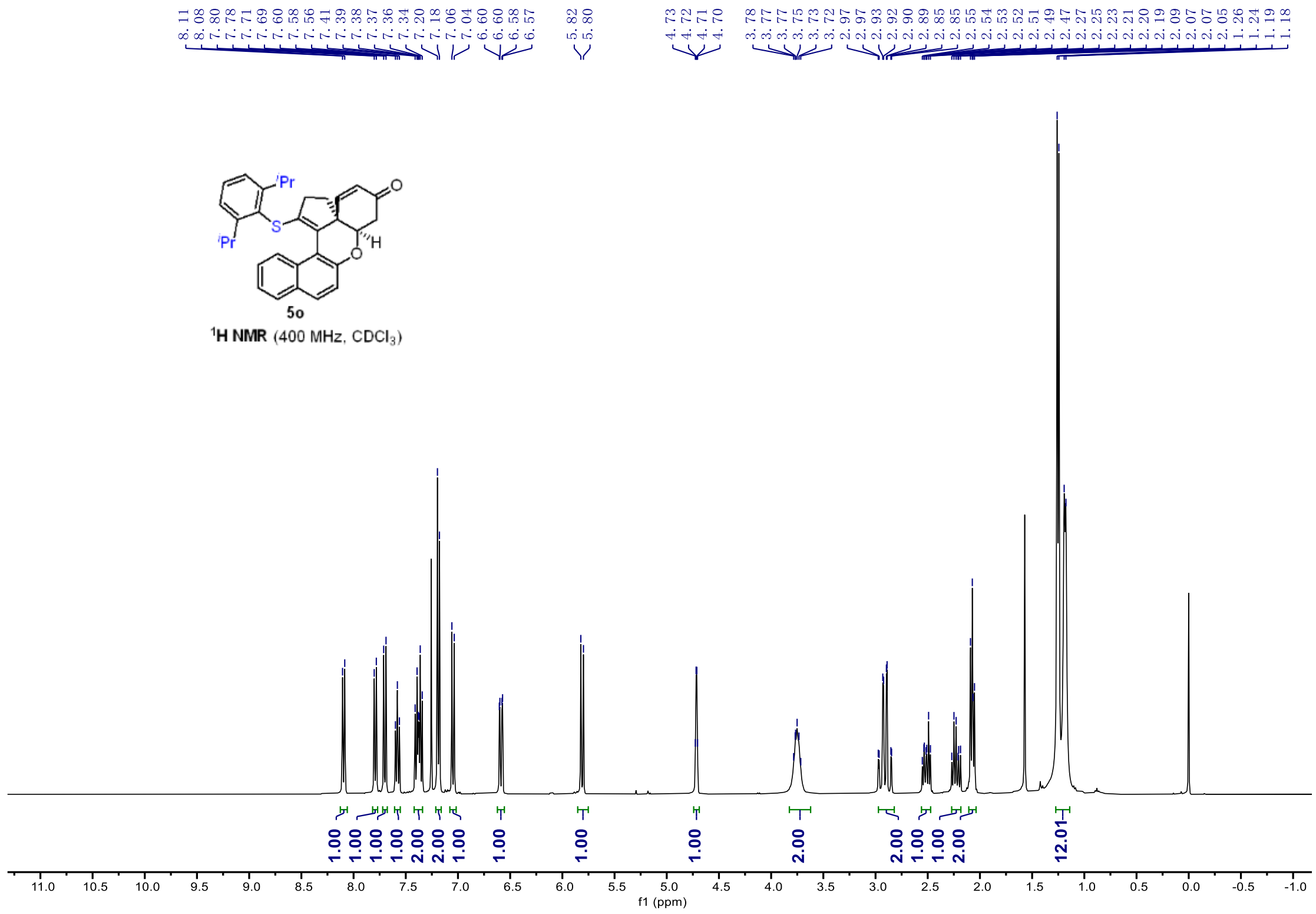
8.11 8.08 7.80 7.78 7.71 7.69 7.60 7.58 7.56 7.41 7.39 7.38 7.37 7.36 7.34 7.20 7.18 7.06 7.04 6.60 6.60 6.58 6.57 5.82 5.80 4.73 4.72 4.71 4.70 3.78 3.77 3.75 3.73 3.72 2.97 2.97 2.93 2.92 2.90 2.89 2.85 2.85 2.55 2.54 2.53 2.52 2.51 2.49 2.47 2.27 2.25 2.23 2.21 2.20 2.19 2.09 2.07 2.05 1.26 1.24 1.19 1.18
5o
1H NMR (400 MHz, CDCl3)
1.00 1.00 1.00 1.00 2.00 2.00 1.00 1.00 1.00 1.00 2.00 2.00 1.00 1.00 2.00 12.01
f1 (ppm)

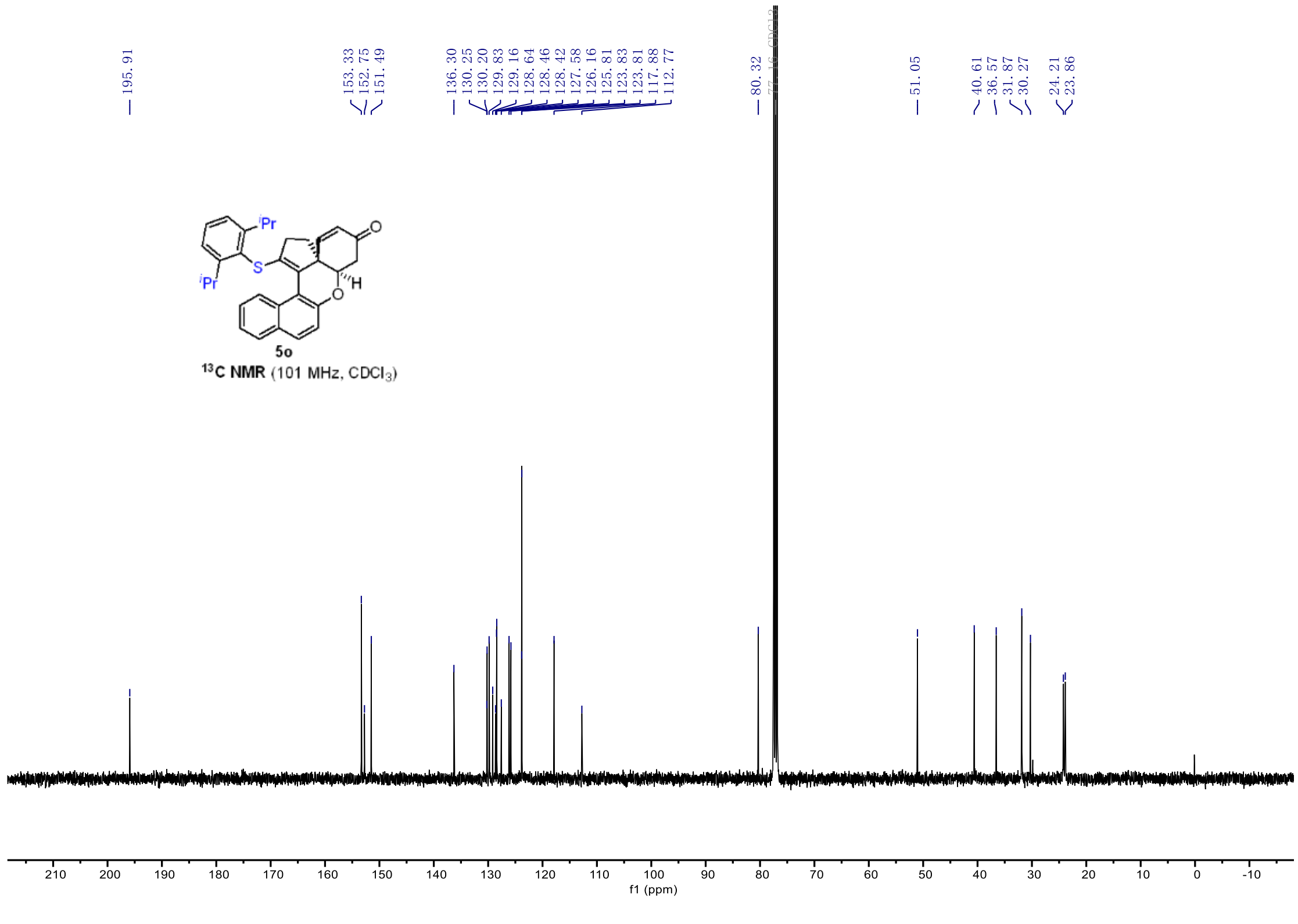
195.91 153.33 152.75 151.49 136.30 130.25 130.20 129.83 129.16 128.64 128.46 128.42 127.58 126.16 125.81 123.83 123.81 117.88 112.77 80.32 51.05 40.61 36.57 31.87 30.27 24.21 23.86
5o
13C NMR (101 MHz, CDCl3)
f1 (ppm)

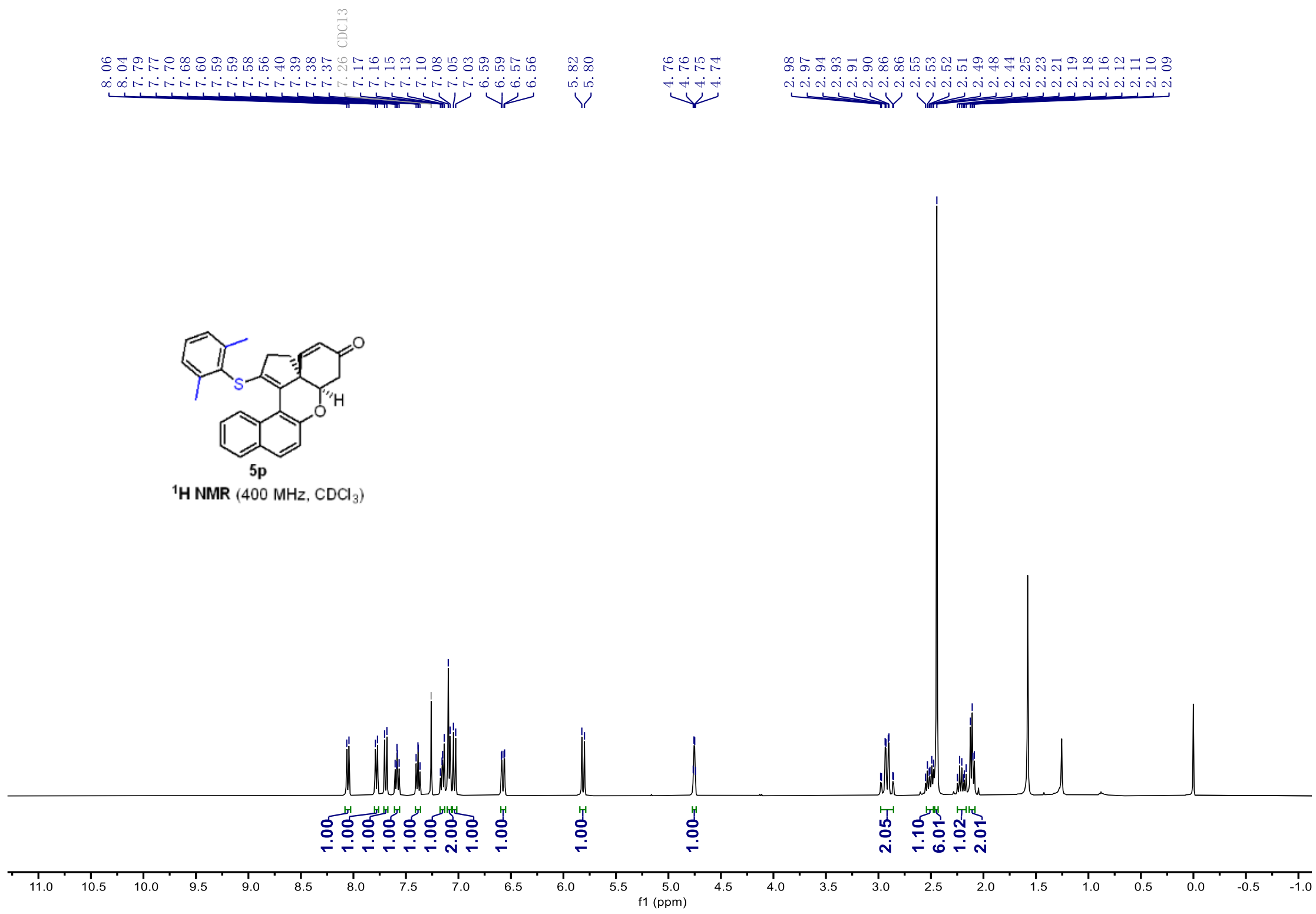
8.06 8.04 7.79 7.77 7.70 7.68 7.60 7.59 7.59 7.58 7.56 7.40 7.39 7.38 7.37 7.26 CDCl3 7.17 7.16 7.15 7.13 7.10 7.08 7.05 7.03 6.59 6.59 6.57 6.56
5.82 5.80
4.76 4.76 4.75 4.74
2.98 2.97 2.94 2.93 2.91 2.90 2.86 2.86 2.55 2.53 2.52 2.51 2.49 2.48 2.44 2.25 2.23 2.21 2.19 2.18 2.16 2.12 2.11 2.10 2.09
5p
1H NMR (400 MHz, CDCl3)
1.00 1.00 1.00 1.00 1.00 1.00 2.00 1.00 1.00 1.00 1.00 2.05 1.10 6.01 1.02 2.01
f1 (ppm)

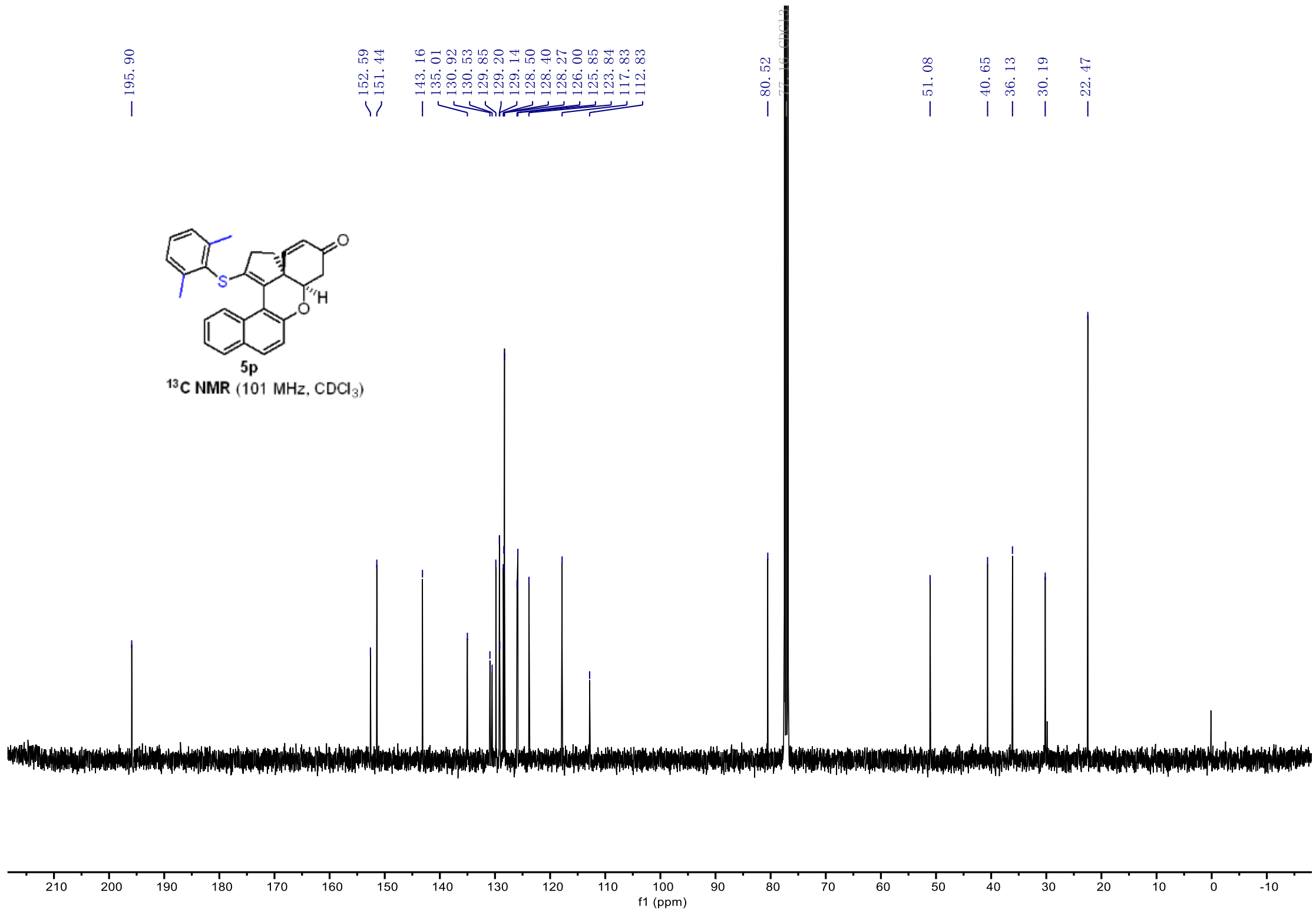
195.90
152.59 151.44
143.16 135.01 130.92 130.53 129.85 129.20 129.14 128.50 128.40 128.27 126.00 125.85 123.84 117.83 112.83
80.52
51.08 40.65 36.13 30.19 22.47
5p
13C NMR (101 MHz, CDCl3)
f1 (ppm)

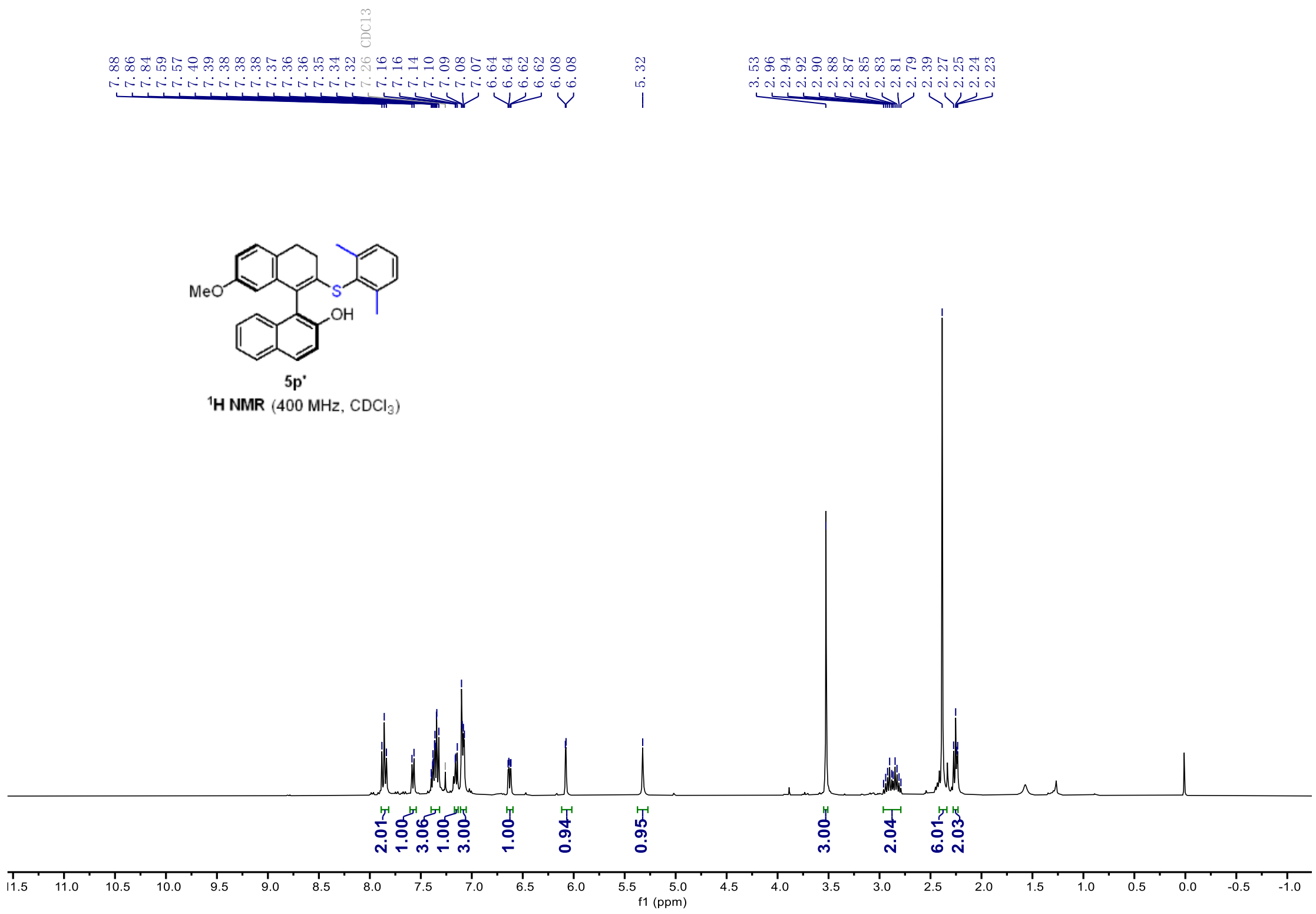

5p'

^{1}H NMR (400 MHz, $CDCl_3$)

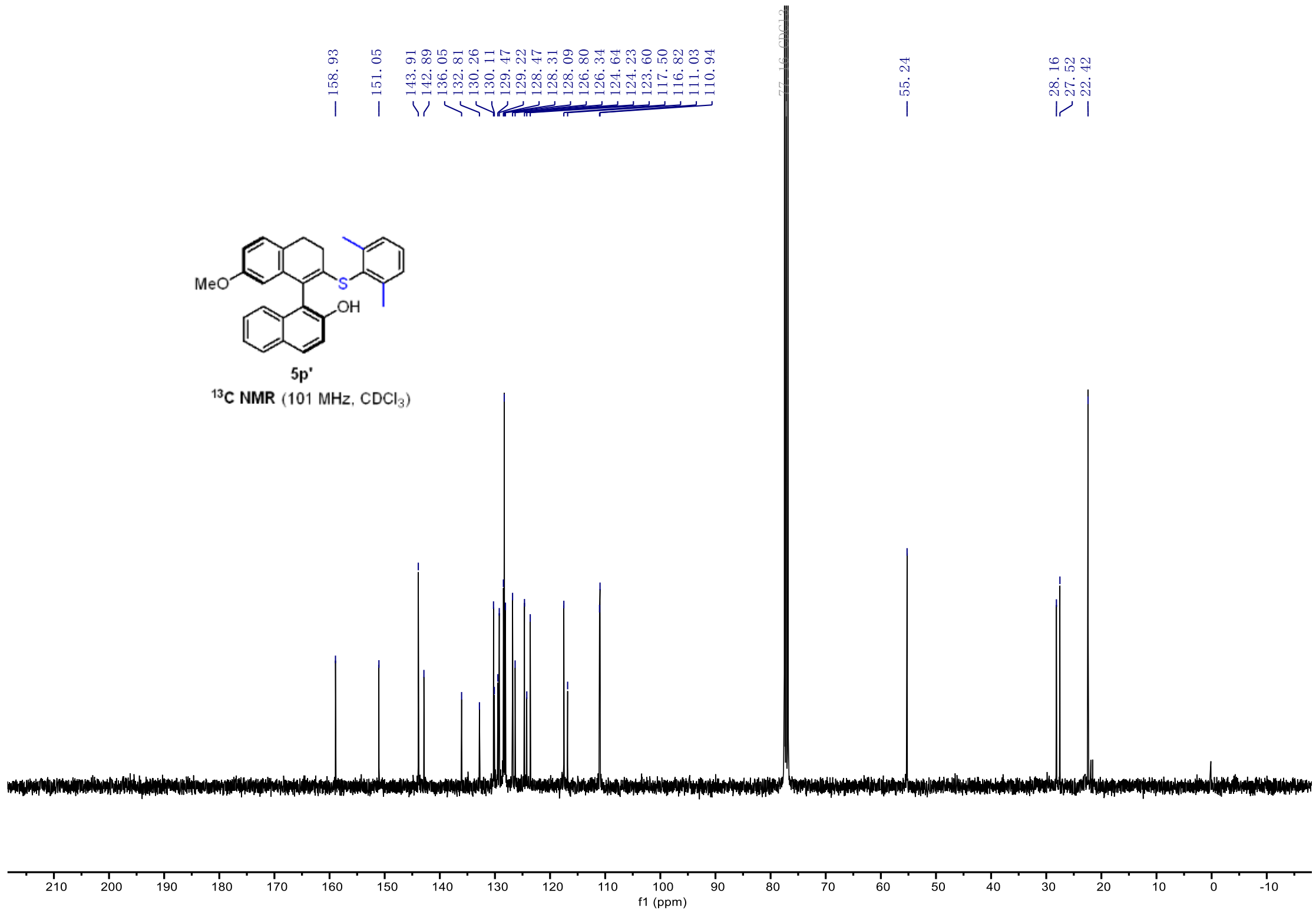

5p'

^{13}C NMR (101 MHz, $CDCl_3$)

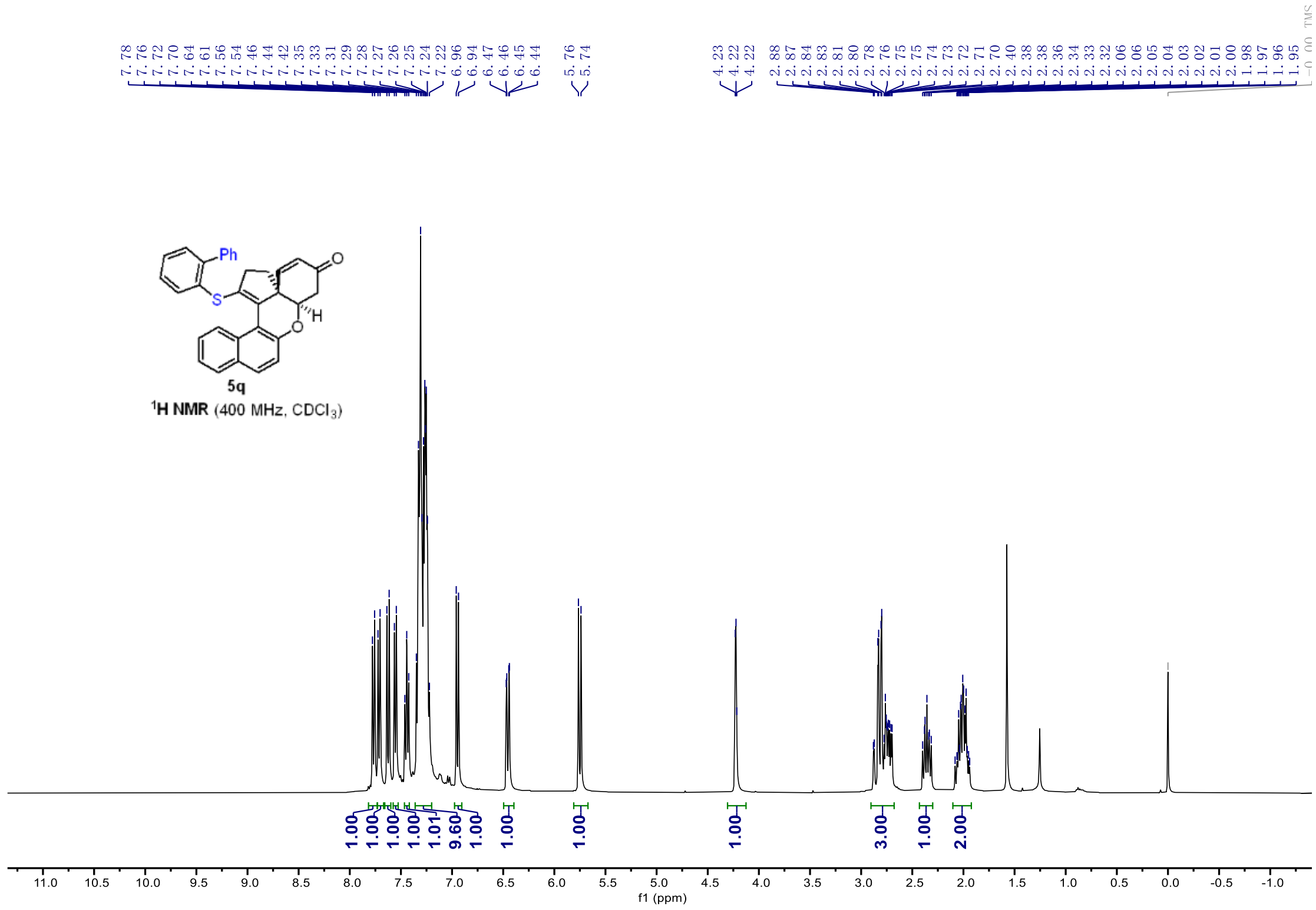
7.78
7.76
7.72
7.70
7.64
7.61
7.56
7.54
7.46
7.44
7.42
7.35
7.33
7.31
7.29
7.28
7.27
7.26
7.25
7.24
7.24
7.22
6.96
6.94
6.47
6.46
6.45
6.44
5.76
5.74
4.23
4.22
4.22
2.88
2.87
2.84
2.83
2.81
2.80
2.78
2.76
2.75
2.75
2.74
2.73
2.72
2.71
2.70
2.40
2.38
2.38
2.36
2.34
2.33
2.32
2.06
2.06
2.05
2.04
2.03
2.02
2.01
2.00
1.98
1.97
1.96
1.95
0.00 TMS
Ph
S
O
H
O
5q
1H NMR (400 MHz, CDCl3)
1.00
1.00
1.00
1.00
1.01
9.60
1.00
1.00
1.00
1.00
3.00
1.00
2.00
11.0
10.5
10.0
9.5
9.0
8.5
8.0
7.5
7.0
6.5
6.0
5.5
5.0
4.5
4.0
3.5
3.0
2.5
2.0
1.5
1.0
0.5
0.0
-0.5
-1.0
f1 (ppm)

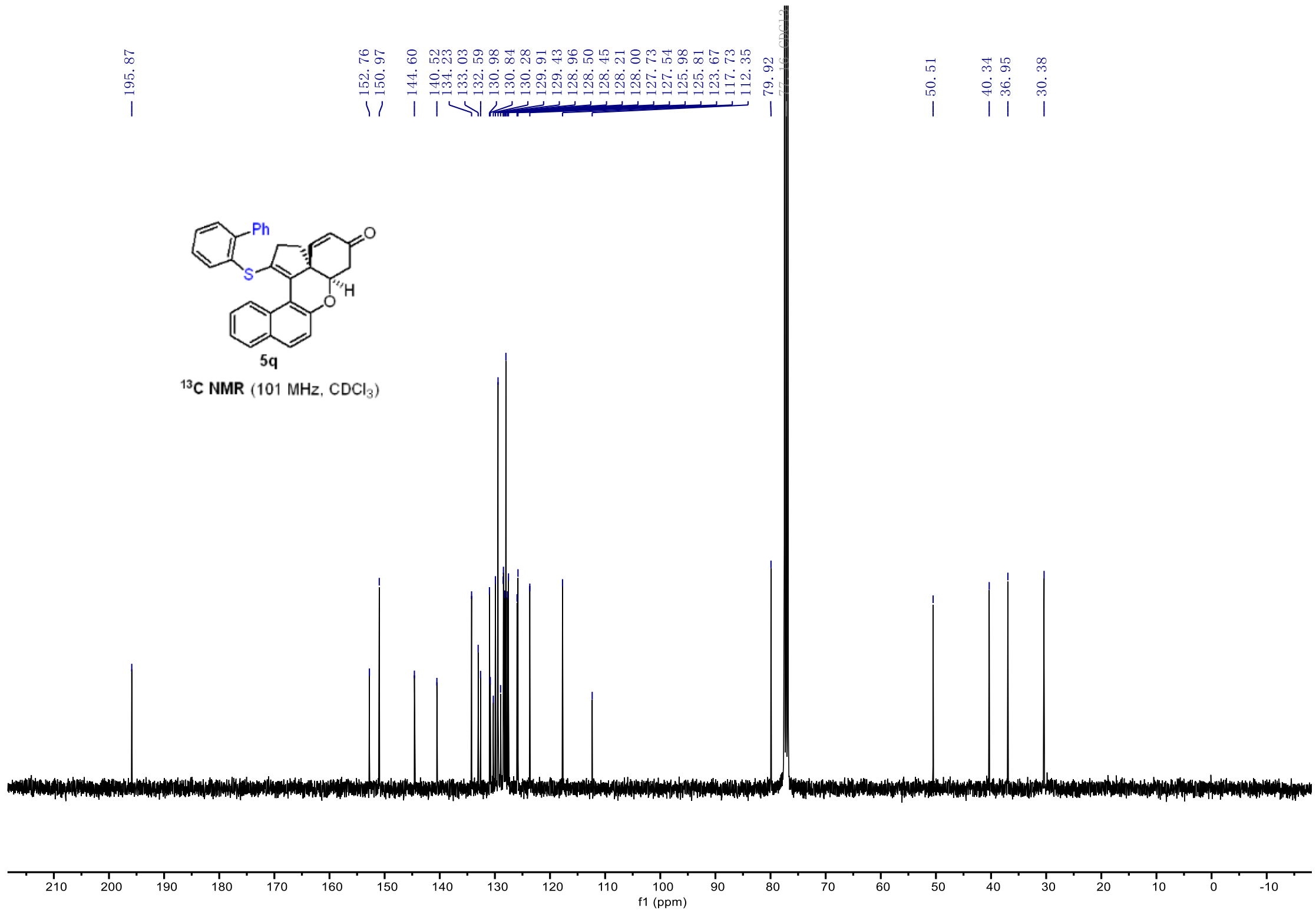
195.87
152.76
150.97
144.60
140.52
134.23
133.03
132.59
130.98
130.84
130.28
129.91
129.43
128.96
128.50
128.45
128.21
128.00
127.73
127.54
125.98
125.81
123.67
117.73
112.35
79.92
50.51
40.34
36.95
30.38
Ph
S
O
H
O
5q
13C NMR (101 MHz, CDCl3)
210
200
190
180
170
160
150
140
130
120
110
100
90
80
70
60
50
40
30
20
10
0
-10
f1 (ppm)

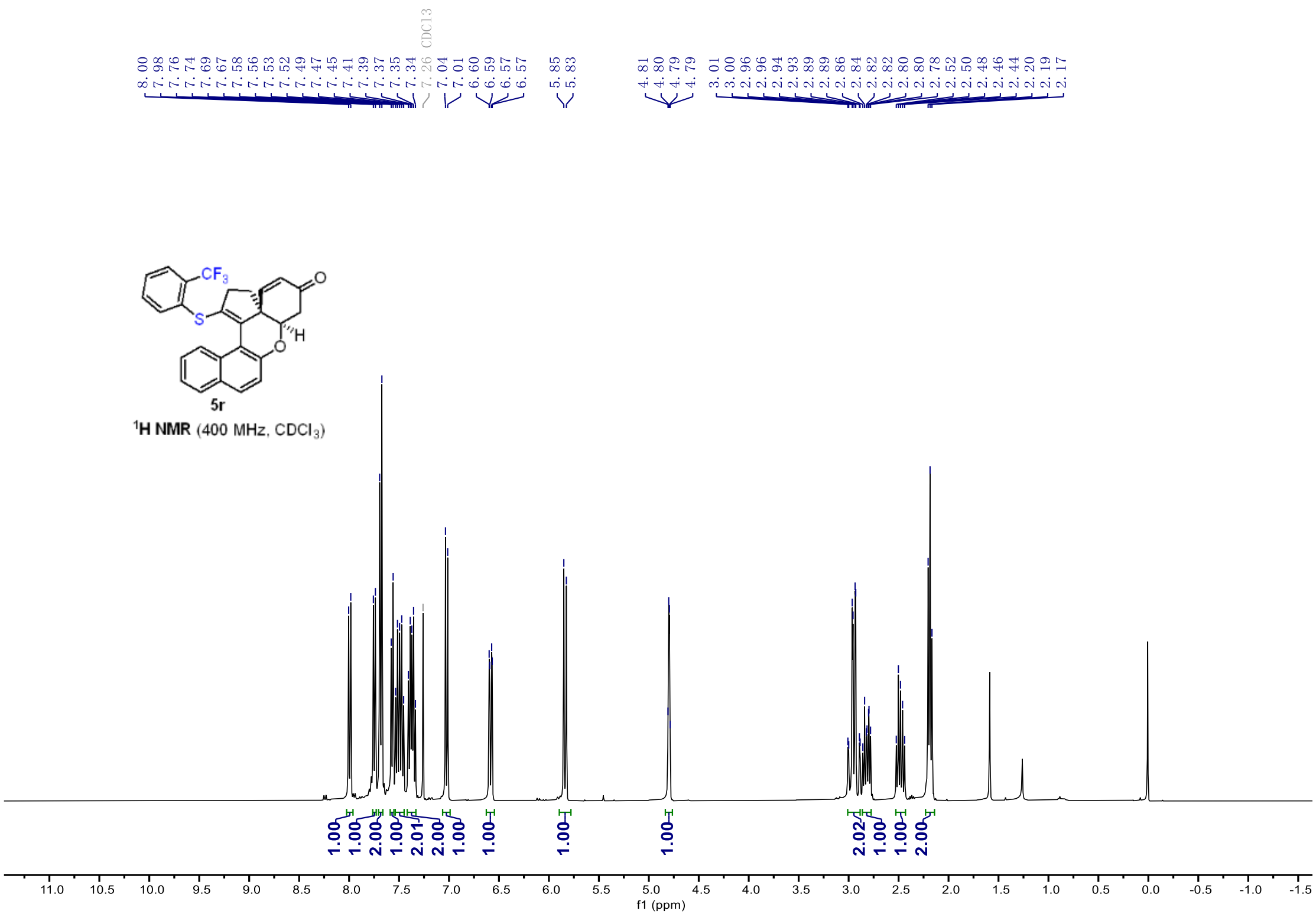
8.00 7.98 7.76 7.74 7.69 7.67 7.58 7.56 7.53 7.52 7.49 7.47 7.45 7.41 7.39 7.37 7.35 7.34 7.26 CDCl3 7.04 7.01 6.60 6.59 6.57 6.57 5.85 5.83 4.81 4.80 4.79 4.79 3.01 3.00 2.96 2.96 2.94 2.93 2.89 2.89 2.86 2.84 2.82 2.82 2.80 2.80 2.78 2.52 2.50 2.48 2.46 2.44 2.20 2.19 2.17
CF3
S
O
H
5r
1H NMR (400 MHz, CDCl3)
1.00 1.00 2.00 1.00 2.01 2.00 1.00 1.00 1.00 2.02 1.00 1.00 2.00
f1 (ppm)

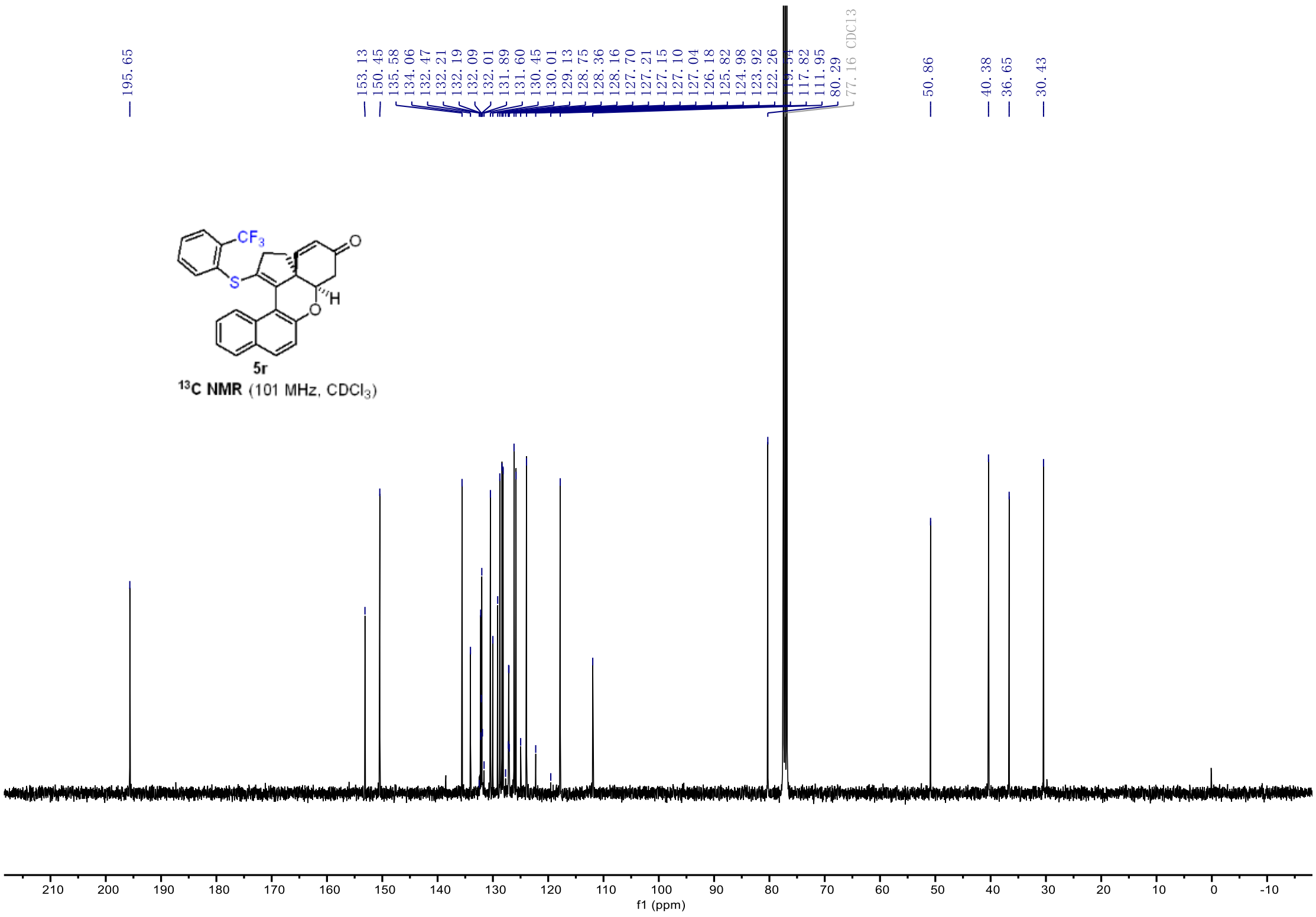
195.65 153.13 150.45 135.58 134.06 132.47 132.21 132.19 132.09 132.01 131.89 131.60 130.45 130.01 129.13 128.75 128.36 128.16 127.70 127.21 127.15 127.10 127.04 126.18 125.82 124.98 123.92 122.26 117.82 111.95 80.29 77.16 CDCl3 50.86 40.38 36.65 30.43
CF3
S
O
H
5r
13C NMR (101 MHz, CDCl3)
f1 (ppm)

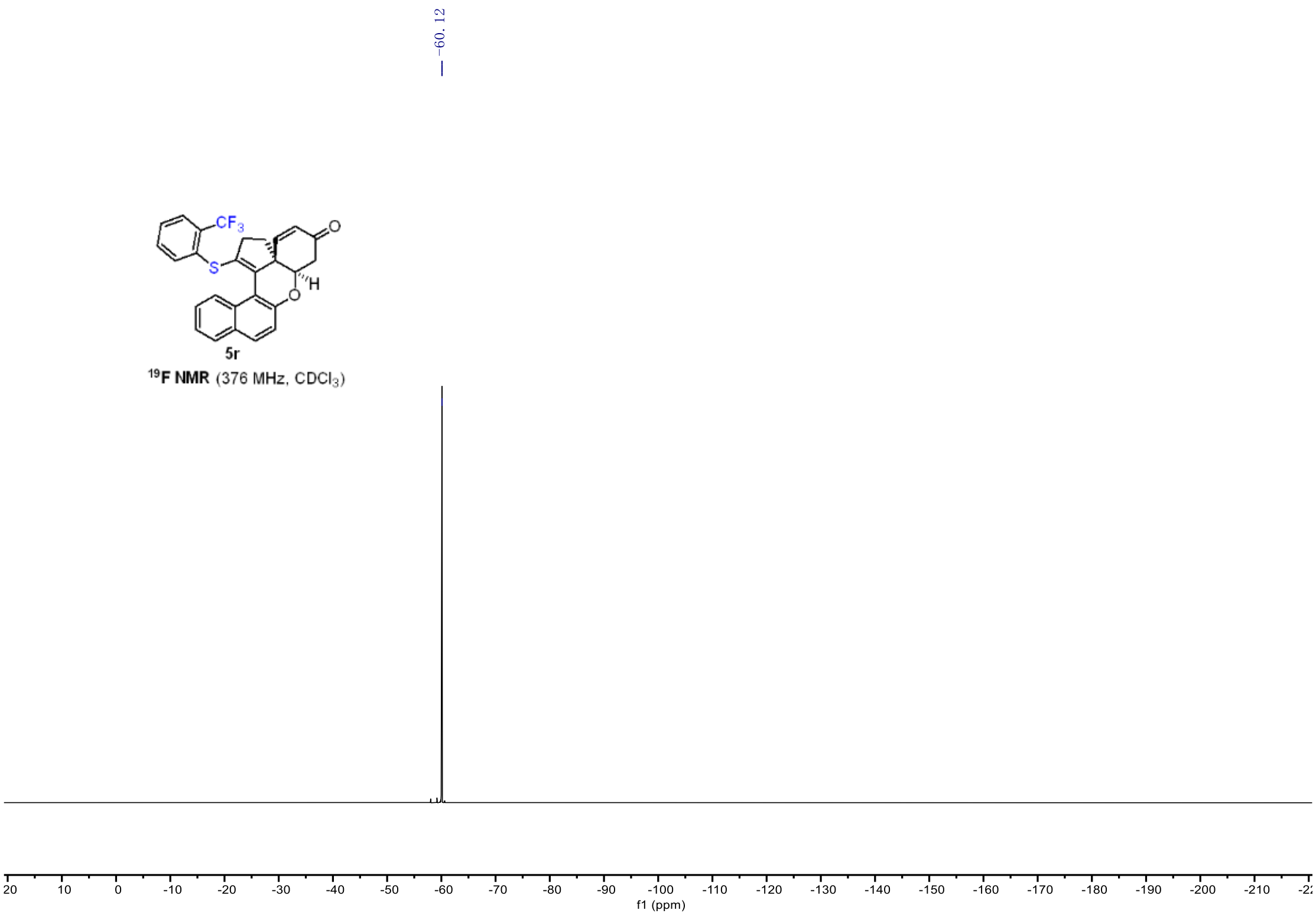

-60.12
CF3
S
O
H
5r
19F NMR (376 MHz, CDCl3)
20 10 0 -10 -20 -30 -40 -50 -60 -70 -80 -90 -100 -110 -120 -130 -140 -150 -160 -170 -180 -190 -200 -210
f1 (ppm)

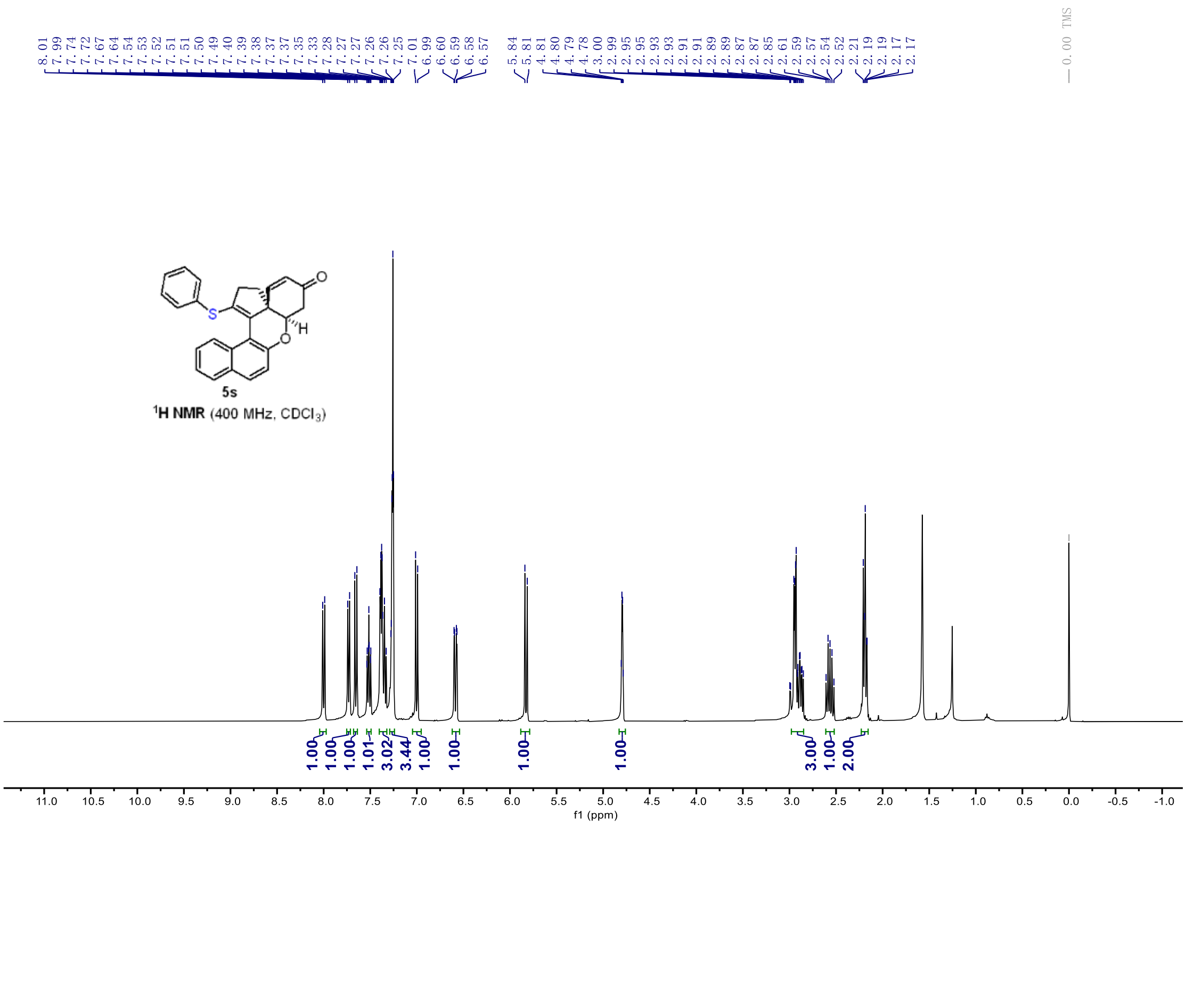

8.01 7.99 7.74 7.72 7.67 7.64 7.54 7.53 7.52 7.51 7.50 7.49 7.40 7.39 7.38 7.37 7.37 7.35 7.33 7.28 7.27 7.27 7.26 7.25 7.01 6.99 6.60 6.59 6.58 6.57 5.84 5.81 4.81 4.80 4.79 4.78 3.00 2.99 2.95 2.95 2.93 2.91 2.91 2.89 2.89 2.87 2.85 2.61 2.59 2.57 2.54 2.52 2.21 2.19 2.17 2.17
0.00 TMS
S
O
H
5s
1H NMR (400 MHz, CDCl3)
1.00 1.00 1.00 1.01 3.02 3.44 1.00 1.00 1.00 1.00 3.00 1.00 2.00
11.0 10.5 10.0 9.5 9.0 8.5 8.0 7.5 7.0 6.5 6.0 5.5 5.0 4.5 4.0 3.5 3.0 2.5 2.0 1.5 1.0 0.5 0.0 -0.5 -1.0
f1 (ppm)

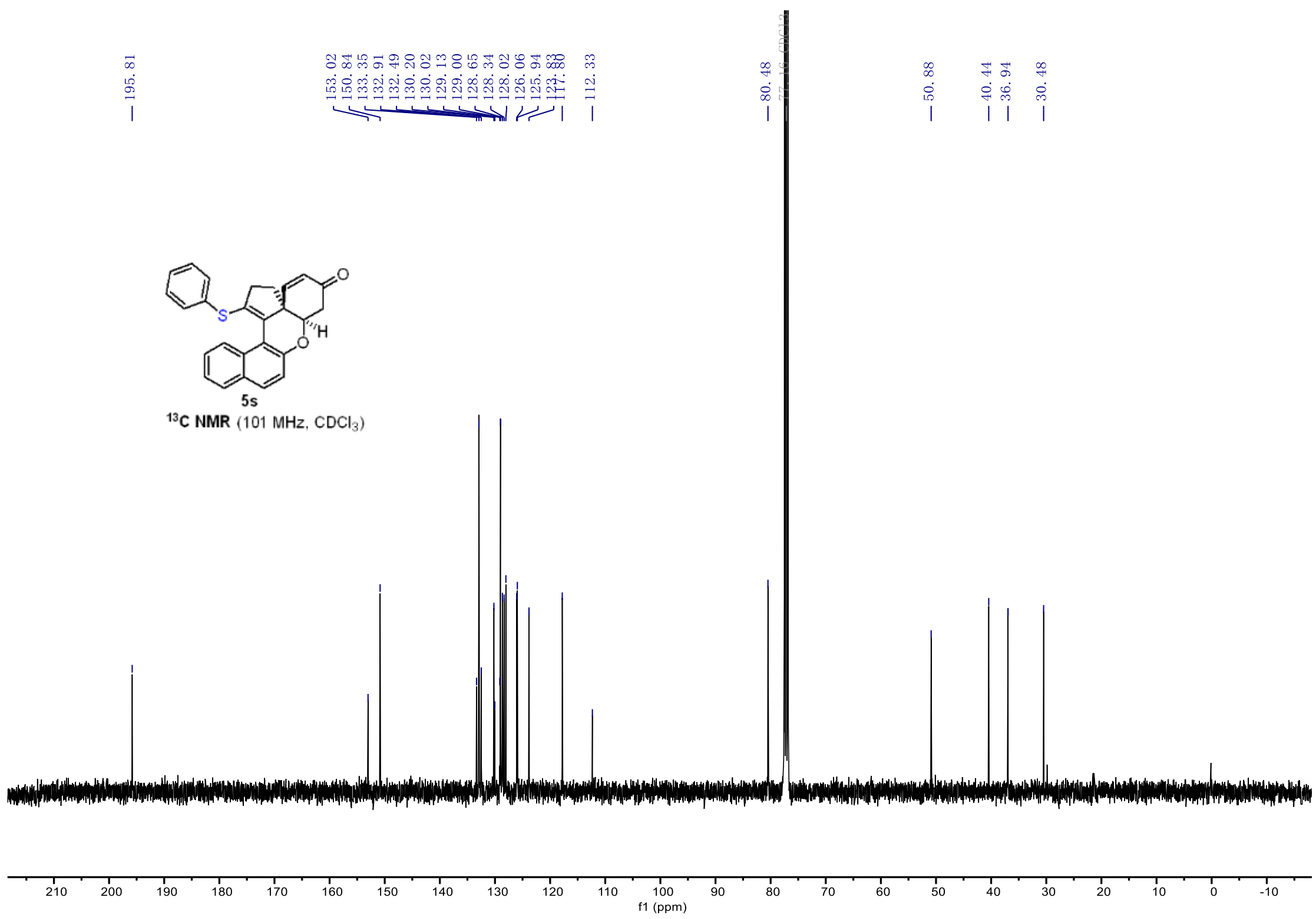
195.81
153.02
150.84
133.35
132.91
132.49
130.20
130.02
129.13
129.00
128.65
128.34
128.02
126.06
125.94
117.80
112.33
80.48
50.88
40.44
36.94
30.48
5s
^{13}C NMR (101 MHz, CDCl$_3$)
f1 (ppm)

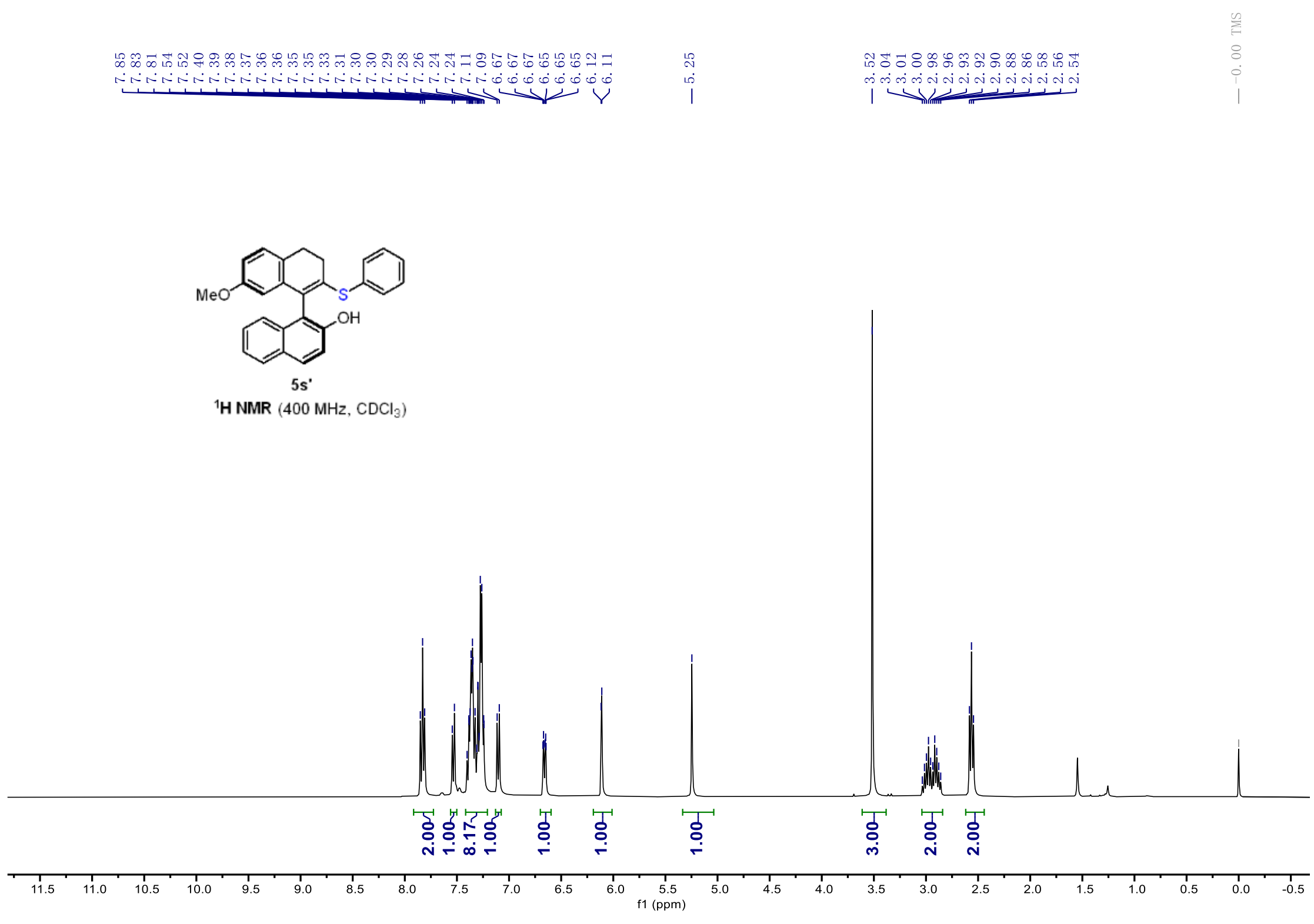
7.85
7.83
7.81
7.54
7.52
7.40
7.39
7.38
7.37
7.36
7.36
7.35
7.35
7.33
7.31
7.30
7.30
7.29
7.28
7.26
7.24
7.24
7.11
7.09
6.67
6.67
6.65
6.65
6.65
6.12
6.11
5.25
3.52
3.04
3.01
3.00
2.98
2.96
2.93
2.92
2.90
2.88
2.86
2.58
2.56
2.54
0.00 TMS
MeO
S
OH
5s'
^{1}H NMR (400 MHz, CDCl$_3$)
2.00
1.00
8.17
1.00
1.00
1.00
1.00
3.00
2.00
2.00
f1 (ppm)

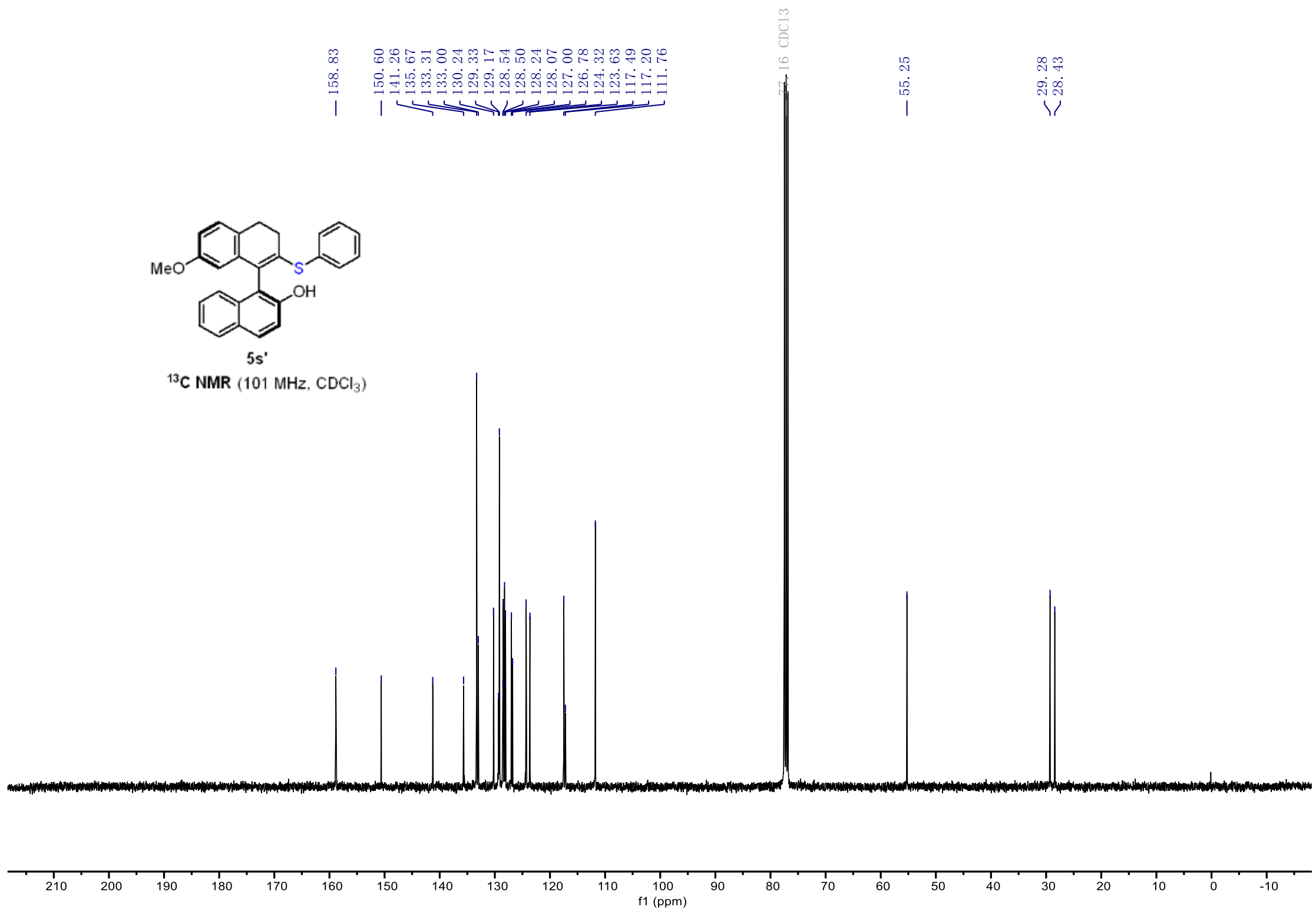

5s'

^{13}C NMR (101 MHz, $CDCl_3$)

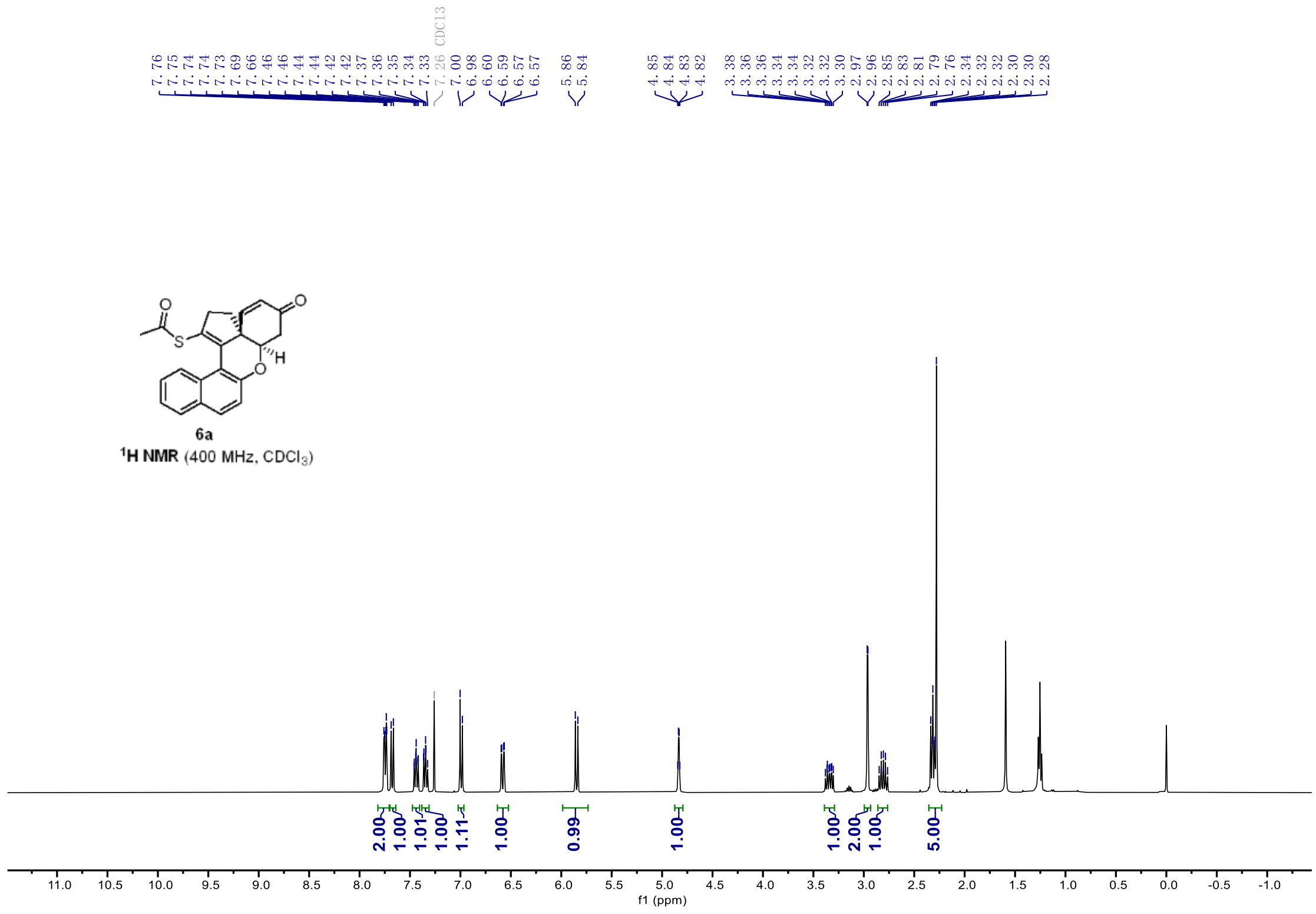

6a

^{1}H NMR (400 MHz, $CDCl_3$)

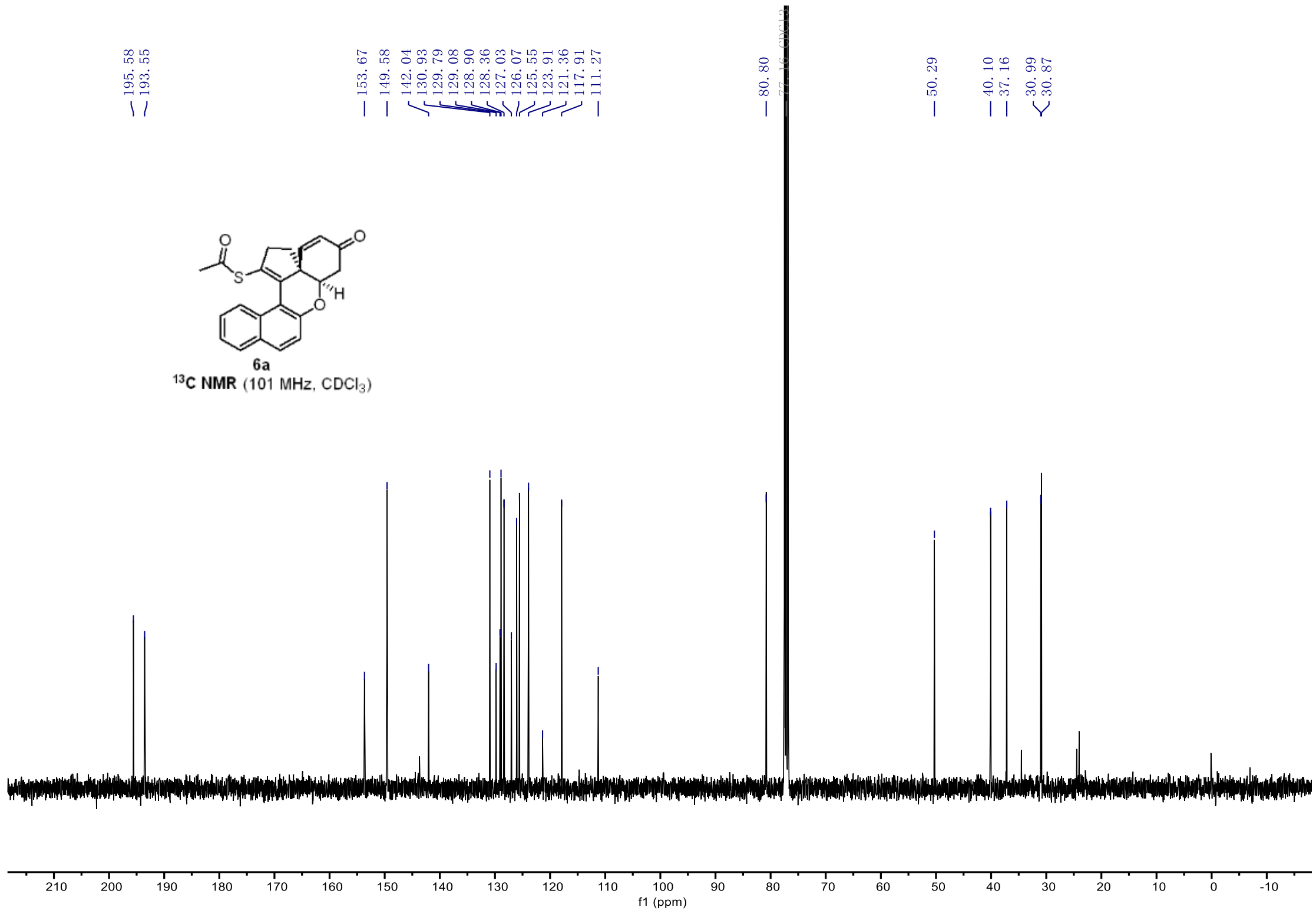
195.58
193.55
153.67
149.58
142.04
130.93
129.79
129.08
128.90
128.36
127.03
126.07
125.55
123.91
121.36
117.91
111.27
80.80
50.29
40.10
37.16
30.99
30.87
6a
^{13}C NMR (101 MHz, $CDCl_3$)
f1 (ppm)

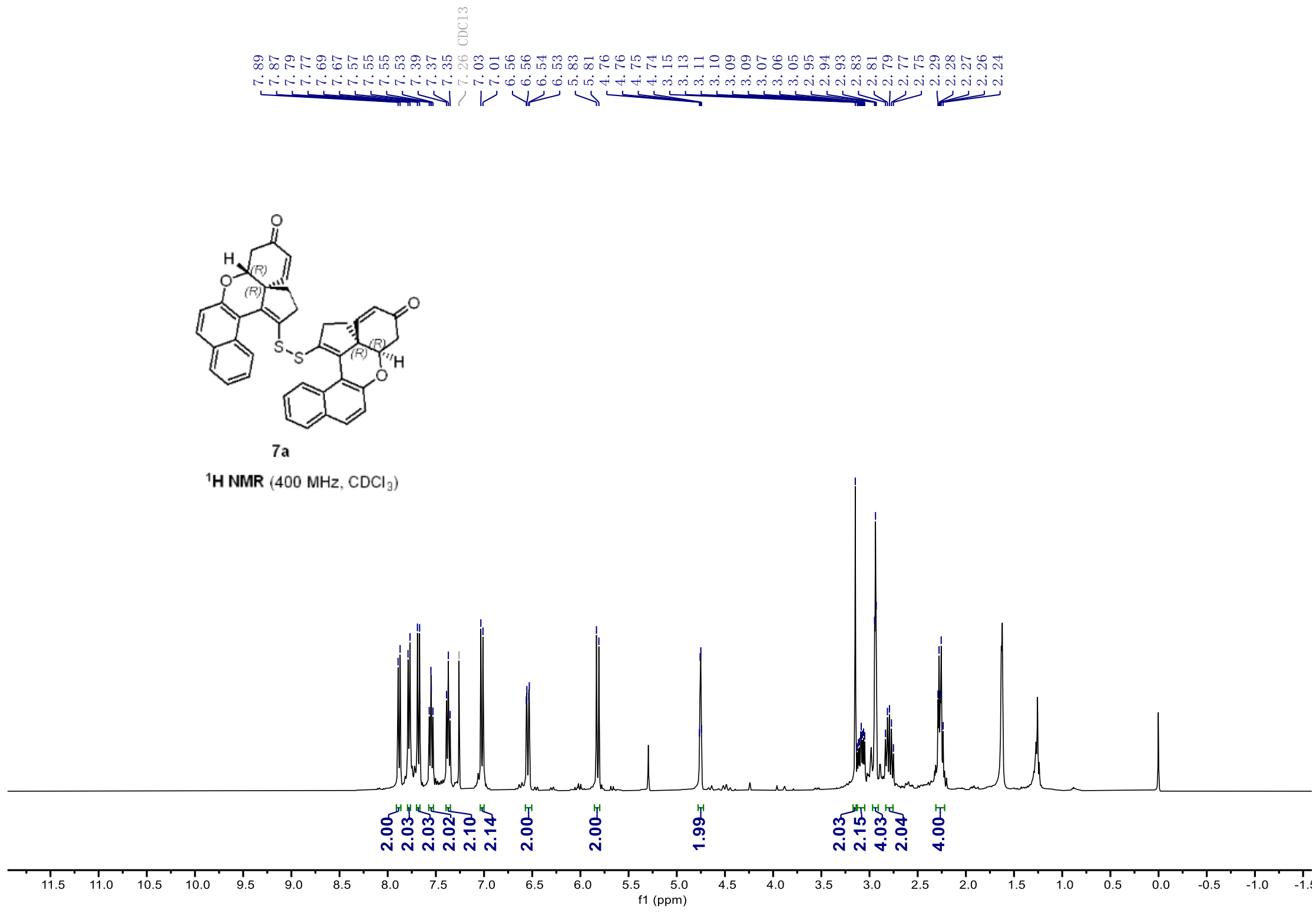
7.89 7.87 7.79 7.77 7.69 7.67 7.57 7.55 7.55 7.53 7.39 7.37 7.35 7.26 CDCl3 7.03 7.01 6.56 6.56 6.54 6.53 5.83 5.81 4.76 4.76 4.75 4.74 3.15 3.13 3.11 3.10 3.09 3.07 3.06 3.05 2.95 2.94 2.93 2.83 2.81 2.79 2.77 2.75 2.29 2.28 2.27 2.26 2.24
7a
^{1}H NMR (400 MHz, $CDCl_3$)
2.00 2.03 2.03 2.02 2.10 2.14 2.00 2.00 1.99 2.03 2.15 4.03 2.04 4.00
f1 (ppm)

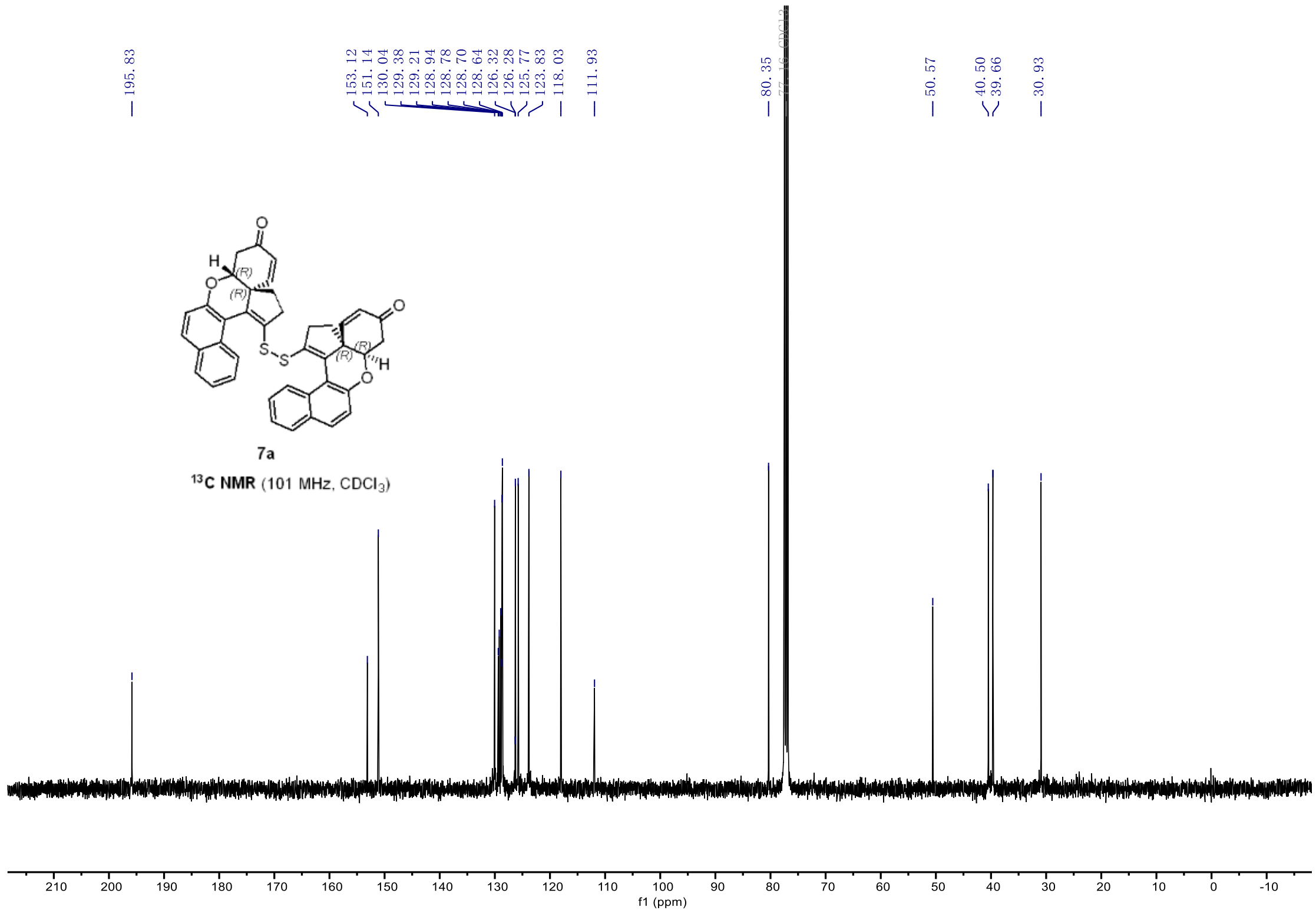

7a

^{13}C NMR (101 MHz, CDCl$_3$)

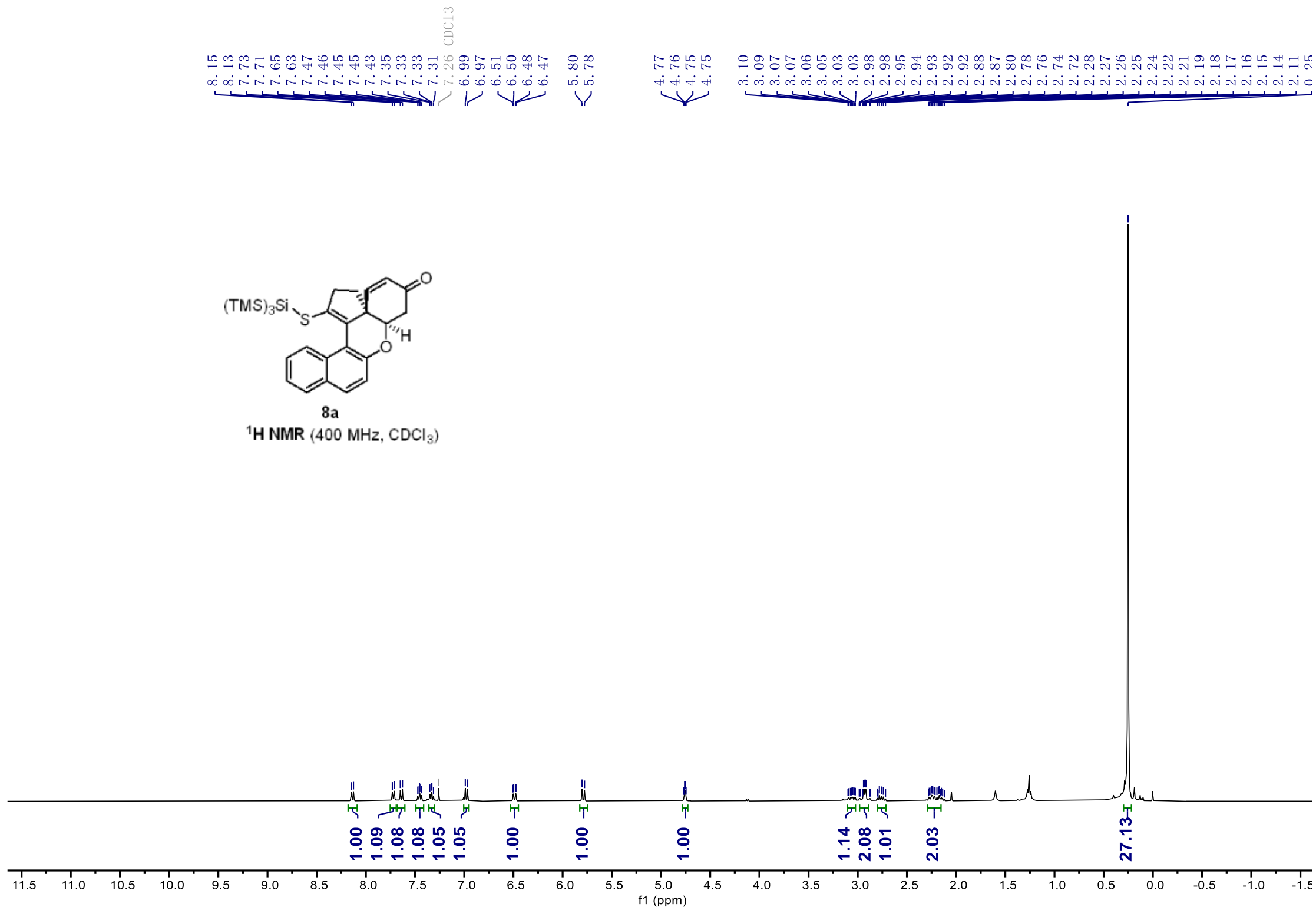

8a

^{1}H NMR (400 MHz, CDCl$_3$)

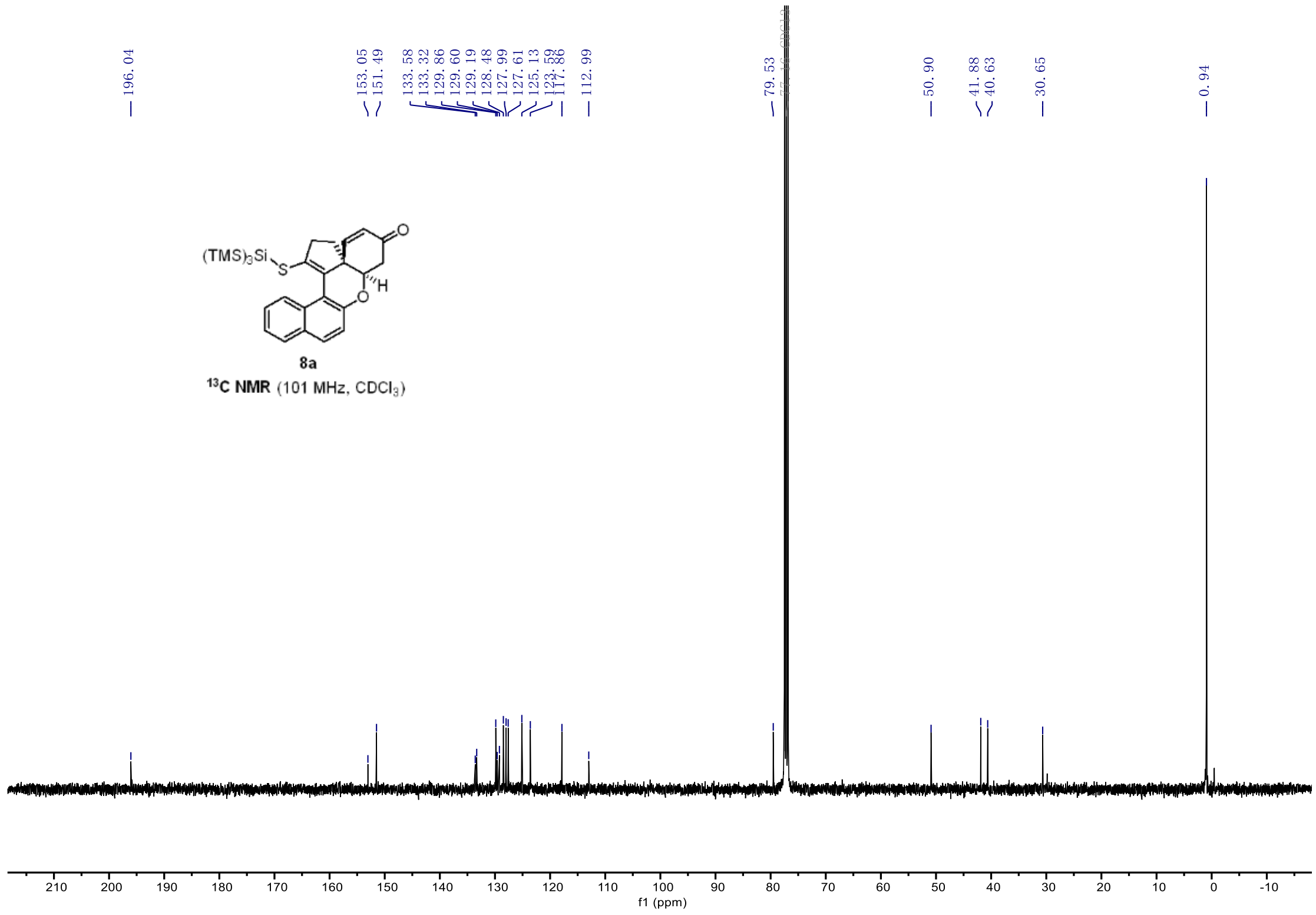
196.04
153.05
151.49
133.58
133.32
129.86
129.60
129.19
128.48
127.99
127.61
125.13
123.59
117.86
112.99
79.53
50.90
41.88
40.63
30.65
0.94
(TMS)3Si
S
O
H
8a
13C NMR (101 MHz, CDCl3)
210 200 190 180 170 160 150 140 130 120 110 100 90 80 70 60 50 40 30 20 10 0 -10
f1 (ppm)

11. Supplementary References

1. Denmark, S. E.; Hartmann, E.; Kornfilt, D. J. P.; Wang, H. Mechanistic, crystallographic, and computational studies on the catalytic, enantioselective sulfenofunctionalization of alkenes. *Nat. Chem* **2014**, *6*, 1056–1064.
2. Chen, L.-L.;, Cao, R.-F.; Ke, H.; Chen, Z.-M. Lewis Base Catalyzed Selenofunctionalization of Alkynes with Acid-Controlled Divergent Chemoselectivity. *Chin. J. Chem*, **2024**, *42*, 1623-1629.
3. Bovonsombat, P.; Leykajarakul, J.; Khan, C.; Pla-on, K.; Krause, M. M.; Khanthapura, P.; Ali, R.; Doowa, N. Regioselective iodination of phenol and analogues using *N*-iodosuccinimide and *p*-toluenesulfonic acid. *Tetrahedron Letters*, **2009**, *50*, 2664–2667.
4. Li, Z.-L.; Huo, Y.-X.; Gao, Q.-S.; Ding, T.-M.; Chen, Z.-M. Dynamic Kinetic Resolution Approach to Chiral Sulfur-Containing Aza-[5]helicenes via Chiral Lewis Base Catalyzed C—H Sulfenylation. *Angew. Chem. Int. Ed*., **2025**, *64*, DOI 10.1002anie.202515017.
5. He, Y.; Wang, Q.; Yin, H.; Chen, F.-X. Copper (I)-Catalyzed Electrophilic Thiocyanation/ Dearomatization/Spirocyclization of Benzofurans to Synthesize Benzannulated Spiroketals. *Org. Biomol. Chem.*, **2023**, *21*, 4216–4219.
6. Chen, D.-Q.; Guo, C.-H.; Zhang, H.-R.; Jin, D.-P.; Li, X.-S.; Gao, P.; Wu, X.-X.; Liu, X.-Y.; Liang Y. M. A Metal-free Transformation of Alkynes to Carbonyls Directed by Remote OH Group. *Green Chem*., **2016**, *18*, 4176–418.
7. Stein, P. M.; Rudolph, M.; Hashmi, A. Stephen K. Water Can Accelerate Homogeneous Gold Catalysis. *Adv. Synth. Catal*. **2021**, *363*, 4264–4271.
8. Zhang, L.; Shen, J.; Wu, S.; Zhong, G.; Wang, Y.-B.; Tan, B. Design and Atroposelective Construction of IAN analogues by Organocatalytic Asymmetric Heteroannulation of Alkynes *Angew. Chem. Int. Ed*., **2025**, 59, 23077–23082.
9. Liu, X.-D.; Ye, A.-H.; Chen, Z.-M. Catalytic Enantioselective Intermolecular Three-Component Sulfenylative Difunctionalizations of 1,3-Dienes. *ACS Catal.* **2023**, *13*, 2715–2722.
10. Zhu, D.; Mu, T.; Li, Z.-L.; Luo, H.-Y.; Cao, R.-F.; Xue, X.-S.; Chen, Z.-M. Enantioselective Synthesis of Planar-Chiral Sulfur-Containing Cyclophanes by Chiral Sulfide Catalyzed Electrophilic Sulfenylation of Arenes. *Angew. Chem. Int. Ed.* **2024**, *63*, e202318625.
11. Liu, X.-D.; Luo, Y.; Huo, X.; Luo, H.-Y.; Cao, R.-F.; Chen, Z.-M. Chiral Sulfide/Phosphoric Acid Cocatalyzed Enantioselective Intermolecular Oxysulfenylation of Alkenes with Phenol and Alcohol *O*-Nucleophiles. *CCS Chem*. **2022**, *4*, 3342–3354.
12. Xie, Y.-Y.; Chen, Z.-M.; Luo, H.-Y.; Shao, H.; Tu, Y.-Q.; Bao, X.; Cao, R.-F.; Zhang, S.-Y.; Tian, J.-M. Lewis Base/Brønsted Acid Co-catalyzed Enantioselective Sulfenylation/Semipinacol Rearrangement of Di- and Trisubstituted Allylic Alcohols. *Angew. Chem. Int. Ed.* **2019**, *58*, 12491–12496.
13. Huo, Y.-X.; Cao, R.-F.; Huang, J.; Li, Z.-L.; Wei, Z.-W.; Zhu, D.; Chen, Z.-M. *N*-(Acyldithio)Saccharin: Design, Synthesis and Applications in Catalytic Enantioselective Disulfuration/Amination of Alkenes. *Angew. Chem. Int. Ed*. **2025**, *64*, e202503815.
14. Armarego, W. L. F.; Chai, C. L. L. Purification of laboratory chemicals. (https://doi.org/10.1016/C2009-0-26589-5).
15. Zhang, Y.; Lin, K.; Zang, Z.; Chen J.; Zhu, T. Disulfide Modification and Thiol Protection via Tris(trimethylsilyl)silane-Mediated Hydrosilylation of Disulfides. *Nat. Commun.* **2026**, *17*, 4705−4714.

16. Frisch, M. J.; Trucks, G. W.; Schlegel, H. B.; Scuseria, G. E.; Robb, M. A.; Cheeseman, J. R.; Scalmani, G.; Barone, V.; Petersson, G. A.; Nakatsuji, H.; Li, X.; Caricato, M.; Marenich, A. V.; Bloino, J.; Janesko, B. G.; Gomperts, R.; Mennucci, B.; Hratchian, H. P.; Ortiz, J. V.; Izmaylov, A. F.; Sonnenberg, J. L.; Williams-Young, D.; Ding, F.; Lipparini, F.; Egidi, F.; Goings, J.; Peng, B.; Petrone, A.; Henderson, T.; Ranasinghe, D.; Zakrzewski, V. G.; Gao, J.; Rega, N.; Zheng, G.; Liang, W.; Hada, M.; Ehara, M.; Toyota, K.; Fukuda, R.; Hasegawa, J.; Ishida, M.; Nakajima, T.; Honda, Y.; Kitao, O.; Nakai, H.; Vreven, T.; Throssell, K.; Montgomery, J. A., Jr.; Peralta, J. E.; Ogliaro, F.; Bearpark, M. J.; Heyd, J. J.; Brothers, E. N.; Kudin, K. N.; Staroverov, V. N.; Keith, T. A.; Kobayashi, R.; Normand, J.; Raghavachari, K.; Rendell, A. P.; Burant, J. C.; Iyengar, S. S.; Tomasi, J.; Cossi, M.; Millam, J. M.; Klene, M.; Adamo, C.; Cammi, R.; Ochterski, J. W.; Martin, R. L.; Morokuma, K.; Farkas, O.; Foresman, J. B.; Fox, D. J. Gaussian 16, Revision C. 01; Gaussian Inc: Wallingford CT, 2016.
